# Synthesis of Quinolizidine-Based 1,4-Azaphosphinines via Cyclization of Heteroarylmethyl(alkynyl)phosphinates

**DOI:** 10.1021/acs.joc.5c02870

**Published:** 2025-12-22

**Authors:** Martin Kos, Tomáš Beránek, Jaroslav Žádný, Natálie Kochová, Karolína Václavíková, Illia Panov, Jan Storch, Ivana Císařová, Jan Sýkora, Vladimír Církva

**Affiliations:** † Department of Materials Chemistry, Research Group of Advanced Materials and Organic Synthesis, 86876 Institute of Chemical Process Fundamentals of the Czech Academy of Sciences, v. v. i., Rozvojová 135, 165 00 Prague 6, Czech Republic; ‡ Department of Inorganic Chemistry, Faculty of Science, Charles University in Prague, Hlavova 2030, 128 40 Prague 2, Czech Republic; § Department of Analytical Chemistry, Faculty of Chemical Engineering, University of Chemistry and Technology, Prague, Technická 5, 166 28 Prague 6, Czech Republic

## Abstract

Intramolecular hydroarylation of (phenylethynyl)­phosphinates represents a powerful strategy for constructing six-membered phosphorus heterocycles. In this study, we report a silver-catalyzed cyclization protocol that enables the efficient synthesis of 1,4-aza-phosphorus heterocycles under mild conditions. The approach demonstrates broad substrate tolerance, affording full conversion across more than 30 derivatives. Dearomatization of pyridine leads to the formation of previously unreported quinolizidine-based 1,4-azaphosphinine scaffolds. These novel phosphorus heterocycles expand the structural diversity of phosphorus-containing frameworks and open new opportunities in the chemistry of functional heterocycles.

## Graphical abstract

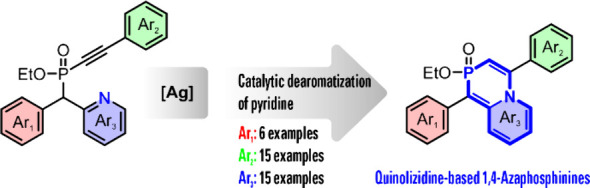

## Introduction

Phosphorus-containing compounds play a pivotal role in modern chemistry due to their versatile applications in catalysis,^1–5^ materials science,^6–9^ and drug discovery.^10–12^ Among these, phosphorus hexacycles stand out as a unique class of compounds, characterized by their ambipolar redox properties,^13,14^ luminescence properties,^15–17^ and potential for postfunctionalization.^7,18^ Therefore, the synthesis of phosphorus-containing heterocycles has evolved considerably over the past several years.^19^ Recent advancements in organophosphorus chemistry have introduced innovative strategies for constructing phosphorus-containing rings, including intra- or intermolecular cyclization of alkynes with phosphonates,^20^ phosphine oxides,^21^ phosphines,^22,23^ and phosphonium salts,^24^ cyclization of ylides,^25^ or [2+2+2] cycloaddition of diynes with phosphaalkynes.^26–28^ Recently, we reported the intramolecular acid-assisted cyclization of phosphinates bearing an acetylene moiety.^29,30^ This method demonstrated the efficient conversion of diphenyl phosphinate **I** into cyclic product **II** in trifluoroacetic acid (Figure 1). Encouraged by these results, we pursued the preparation of the aza analogue of cyclic phosphinate **5p**, which could potentially be transformed into bidentate pyridyl-phosphinine ligands.^31^ However, we observed that under these conditions, the reaction is extremely slow and ineffective (see for details). Inspired by the work of Lee et al.^20^ on similar transformations involving carbophilic activation,^32,33^ we explored the use of a gold/silver catalyst. Intriguingly, while phosphinate **1p** exhibited high reactivity under these conditions, the cyclization occurred not at the phenyl but rather at the pyridyl, leading to the dearomatization of pyridine and the formation of **2p** with an unprecedented 1,4-azaphosphinine scaffold. Although this transformation is documented for pyridines bearing an alkyne activated by a carbonyl group,^34–37^ its application in phosphorus chemistry has remained unexplored. Motivated by this observation, we decided to study the reaction in detail.

**Figure 1:**
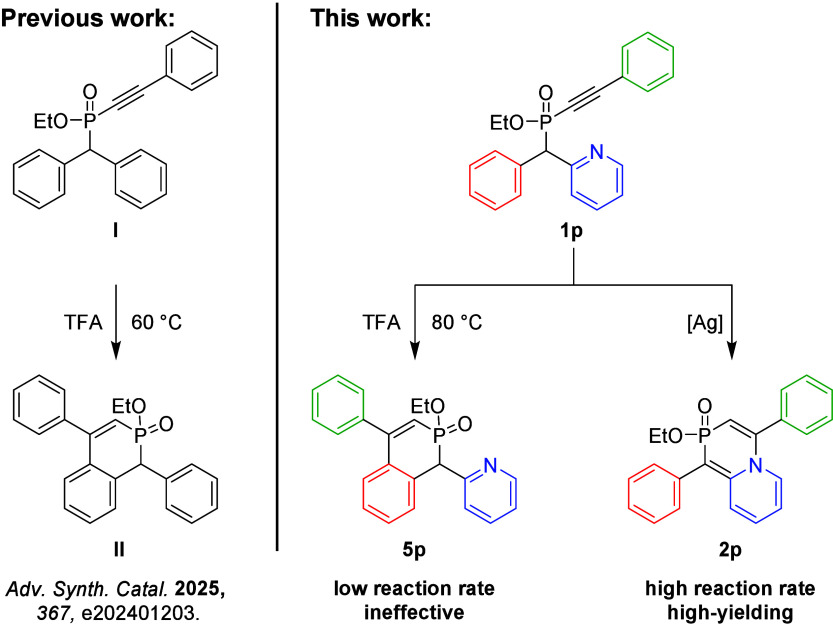


## Results and Discussion

We began our study with the preparation of phosphinate **1a** as a model compound via the Michaelis–Arbuzov reaction. The starting material, 2-bromomethylpyridine, is intrinsically unstable due to the presence of both electrophilic and nucleophilic moieties. Therefore, it is commercially available only as its hydrobromide. To obtain the free base, the salt was deprotonated by extraction between DCM and aqueous potassium carbonate. We observed that the resulting solution was relatively stable at room temperature but decomposed upon heating or solvent evaporation, as indicated by the change in color from transparent to deep red.^38^ Due to its instability, the solution of 2-bromomethylpyridine in DCM was reacted directly with phosphonite **6a**. Refluxing this mixture resulted in only negligible formation of phosphinate **1a**. To overcome this limitation, the reaction mixture was gradually heated to 100 °C, during which the solvent was distilled off while the starting material was converted almost completely. Nevertheless, this treatment inevitably caused decomposition of 2-bromomethylpyridine, resulting in a relatively low isolated yield of **1a**.

Having **1a** in hand, we proceeded to screen various π-acidic transition metal-based catalysts to optimize the reaction conditions (Table 1). Copper­(I) and copper­(II) catalysts, as well as palladium­(II) acetate, showed no conversion (entries 1–3). A gold­(I) catalyst, Ph_3_PAuNTf_2_, exhibited low catalytic activity, providing a conversion of 12% (entry 4). Silver­(I) salts demonstrated more promising results, with AgNO_3_ yielding 36% (entry 5), AgF 44% (entry 6), and AgBF_4_ 54% conversion (entry 7). Remarkably, CH_3_COOAg and CF_3_COOAg both achieved complete conversion of **1a** with excellent isolated yields (entries 8 and 9, respectively). Reducing the loading of CF_3_COOAg from 0.1 to 0.02 equiv maintained full conversion (entry 10). Additionally, the reaction also proceeded with complete conversion in other chlorinated (CHCl_3_ and DCE) and nonchlorinated (DMF, THF, and MeOH) solvents.

**Table 1:**
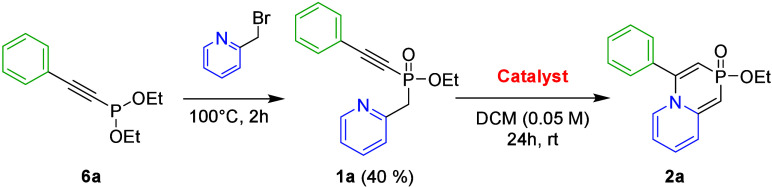
Catalyst Screening

entry^a^	catalyst (equiv)	conversion^b^ (%)
1	[(MeCN)_4_Cu]PF_6_ (0.1)	0
2	Cu(OTf)_2_ (0.1)	0
3	Pd(OAc)_2_ (0.1)	0
4	Ph_3_PAuNTf_2_ (0.1)	12
5	AgNO_3_ (0.1)	36
6	AgF (0.1)	44
7	AgBF_4_ (0.1)	54
8	CH_3_COOAg (0.1)	100 (80)^c^
9	CF_3_COOAg (0.1)	100 (87)^c^
10	CF_3_COOAg (0.02)	100

a Reactions were performed using 0.1 mmol of **1a**.

b The conversion was calculated by measuring the ratio of starting material **1a** to product **2a** in the ^31^P NMR spectrum of the crude reaction mixture.

c Isolated yield.

The proposed mechanism is depicted in Scheme 1.^36^ Initially, the triple bond in **1a** is activated by the π-acidic silver­(I) catalyst, which promotes nucleophilic attack by the pyridine lone pair, leading to the formation of a pyridinium species. Deprotonation of the pyridinium intermediate at the acidic α-position of phosphinate then generates a vinyl silver species. Subsequent protodemetalation of this intermediate affords final 1,4-azaphosphinine product **2a** and regenerates the silver­(I) catalyst.

**Scheme 1:**
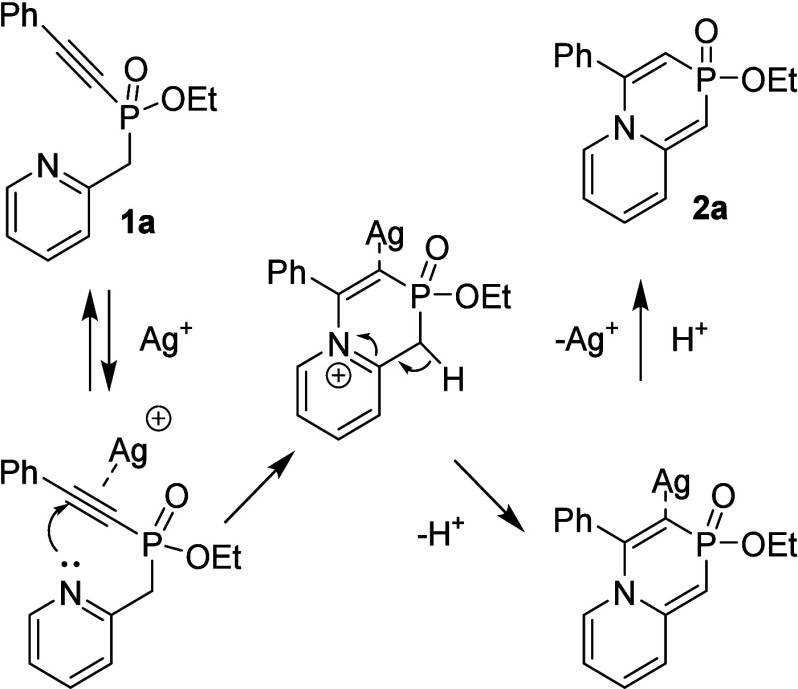
Plausible Reaction Mechanism for Ag­(I)-Catalyzed Pyridine Dearomatization

The scope of this transformation was examined by first evaluating the influence of the electronic and steric properties of the aryl group attached to the acetylene moiety (Scheme 2, colored green). For this purpose, a series of phosphinates **1a**–**o** were synthesized from phosphonites **6a–o**, respectively, which were in turn prepared from commercially available substituted phenylacetylenes (for details, see ). Due to the low stability of intermediates **6a–o**, they were used in the transformation into **1a**–**o**, respectively, without any purification. This two-step procedure afforded **1a–h** in 30–40% yields. For substrates bearing strong electron-withdrawing substituents (**1i–o**), the yields decreased significantly, reaching 6% in the case of 2-trifluoromethyl-substituted phosphinate **1o**. Cyclization of **1a**–**o** proceeded successfully; all substituents were well tolerated, providing the desired products **2a–o**, respectively, in good to excellent yields. Notably, methoxy- and dimethoxy-substituted phosphinates **1b–e** exhibited exceptional reactivity, affording the corresponding products in excellent yields ranging from 90% to 96%. In the case of dimethoxy phosphinate **1d**, the yield can be ascribed to its high reactivity, which even led to spontaneous cyclization of **1d** to **2d** during the purification. After the silica gel column, a few percent of cyclized product **2d** was observed in the NMR spectra (see ). Similar behavior was also observed for other phosphinates in this study (e.g., isoquinoline derivative **3j** (see )). On the other hand, substrates bearing electron-withdrawing groups such as fluoro, difluoro, cyano, or trifluoromethyl (**2j–o**) also exhibited good reactivity, although the yields were slightly lower than those of phosphinates with ED groups. The influence of sterically demanding *ortho* substituents was found to be negligible when comparing the cyclization of *ortho*-substituted (**1c** and **1o**) and *para*-substituted (**1b** and **1n**) phosphinates. However, the presence of *ortho* substitutions in products **2c** and **2o** resulted in hindered rotation and, consequently, in the formation of axial chirality, giving rise to two diastereomers observed in the NMR spectra (see ).

**Scheme 2:**
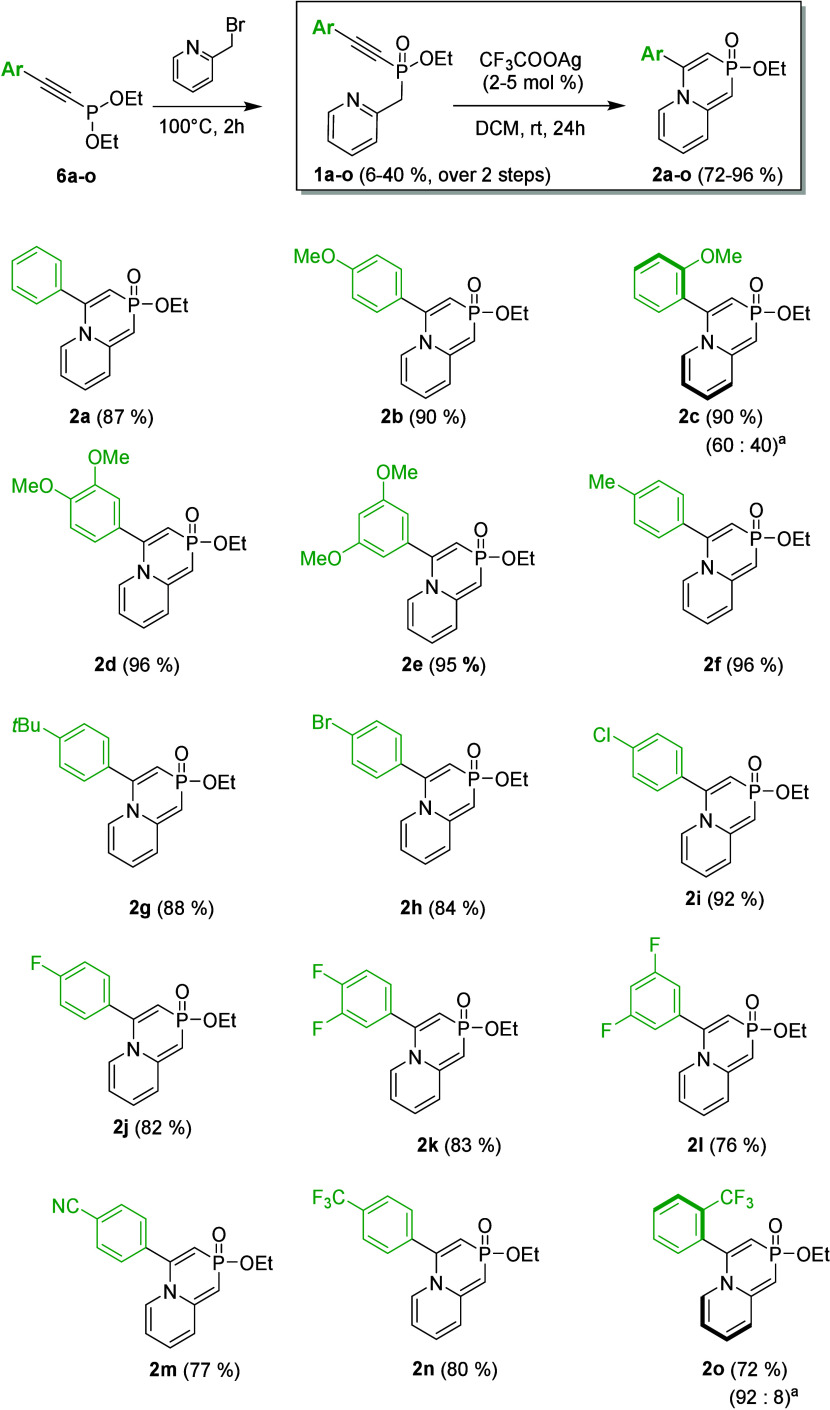
Reactivity of Phosphinates **1a**–**o** Bearing Substituted Arylacetylenes

a Diastereomeric ratios (dr).

Next, the influence of an additional aromatic ring attached to the benzylic position was evaluated (Scheme 3, colored red). To investigate this effect, diarylmethyl bromides **8p–u** were synthesized by the reaction of 2-lithiopyridine with the corresponding aldehydes, followed by bromination of resulting alcohols **7p–u**, respectively, using the Appel reaction (for details, see ). Notably, the electronic properties of the attached aryl rings played a crucial role in determining the stability of bromomethylpyridines **8p–u**. For instance, trifluoromethyl-substituted derivative **8r** exhibited relatively high stability, allowing for successful isolation via column chromatography. In contrast, the presence of an electron-donating methoxy group significantly reduced the stability, causing the chromatographic isolation of **8q** to be impossible. Thus, methoxy derivative **8q** was obtained via bromination with PBr_3_ and used directly in the next step without further purification. All phosphinates **1p–u** were obtained as inseparable mixtures of diastereomers in yields of approximately 30%, except for methoxy-substituted **1q**, which was isolated in only 8% yield due to the previously mentioned low stability of bromide **8q**. The cyclization of all starting materials **1p–u** proceeded smoothly, affording products **2p–u**, respectively, in excellent yields (85–98%). No significant difference in reactivity was observed between electron-donating methoxy phosphinate **1q** and electron-withdrawing trifluoromethyl phosphinate **1r**, suggesting that the electronic effects of the attached aryl ring had minimal influence on the efficiency of the cyclization under the applied conditions. Moreover, *ortho* substitution in phosphinates **1s** and **1u** did not affect their reactivity.

**Scheme 3:**
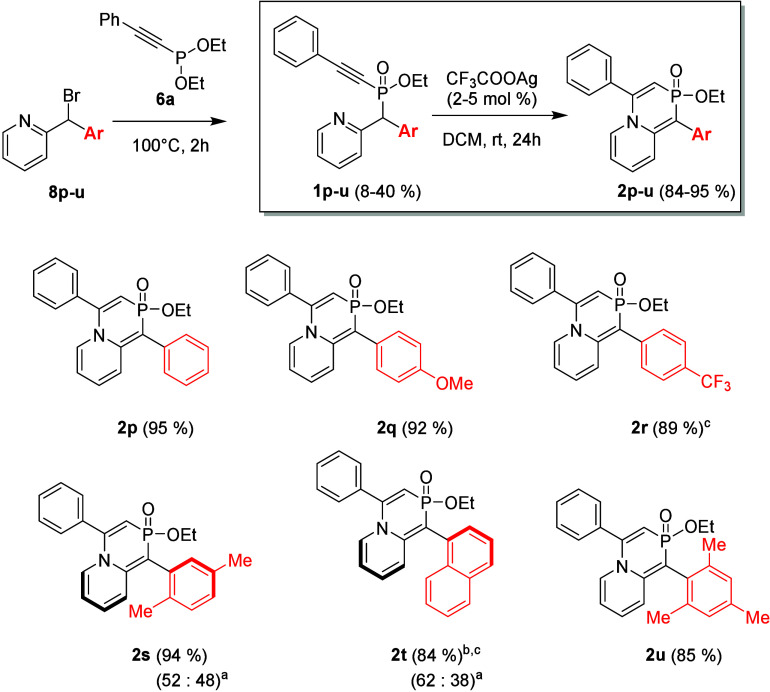
Reactivity of Phosphinates **1p**–**u** Bearing an Additional Aromatic Ring

a Diastereomeric ratios (dr).
b Overall yield.
c CCDC registration numbers are 2489828 for **2r**, 2489827 for (*S*_*a*_ *,R*)**-2t**, and 2489829 for (*R*_*a*_ *,R*)**-2t**.

The reaction of 1-naphthyl phosphinate **1t** led to the formation of a binaphthyl scaffold, resulting in the generation of two pairs of diastereomers: (*S*_*a*_,*R*)/(*R*_*a*_,*S*)-**2t** and (*R*_*a*_,*R*)/(*S*_*a*_ *,S*)-**2t**. These diastereomers were successfully separated by column chromatography. Furthermore, single crystals of both diastereomeric pairs, suitable for X-ray crystallographic analysis, were obtained by slow cooling of their EtOAc solution, enabling the assignment of the absolute configuration of both isomers (Figure 2A). The aromatic character of the pyridine ring in **2t** was investigated using DFT calculations at the B3LYP/6-311++G­(d,p) level of theory (Figure 2B). The N-heterocycle displayed a NICS(0) value of −0.66, which is consistent with the proposed dearomatization reaction. This finding is further supported by the alternation of bond lengths observed in the X-ray crystal structure, which reveals the 1,4-diene character of the pyridine moiety.

**Figure 2:**
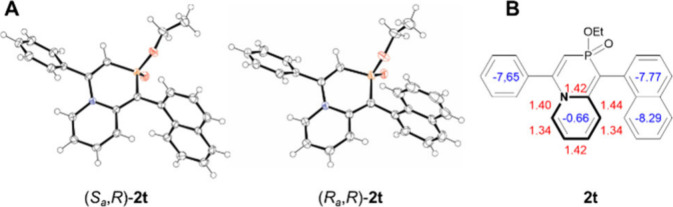
(A) ORTEP projection of the crystal structure of diastereomers of **2t**. Hydrogen atoms have been omitted for the sake of clarity. Thermal ellipsoids are shown with 50% probability. (B) NICS(0) values (blue) and bond lengths (angstroms, red).

Finally, the reactivity of phosphinates **3a–m** bearing various nitrogen-containing heterocycles was evaluated (Scheme 4, colored blue). This investigation aimed to explore the influence of different heterocyclic frameworks on the cyclization process and the stability of the resulting products. Pyrazine (**3a** and **3b**) and pyrimidine (**3c**) phosphinates, containing two nitrogen atoms, exhibited reactivity comparable to that of parent pyridine derivative **1a**, affording 1,4-azaphosphinines **4a**–**c**, respectively, in excellent yields (86–95%). A more intriguing difference was observed between the reactivities of quinoline **3h** and isoquinoline **3j** derivatives. While isoquinoline **3j** underwent cyclization smoothly, yielding **4j** overnight, the reaction of quinoline **3h** proceeded much more slowly. Full conversion was eventually achieved after 13 days. Even lower reactivity was observed for quinoxaline derivative **3i**, where only approximately 50% conversion was reached after 18 days. Furthermore, no reaction was observed in the case of even more sterically hindered dibenzo­[*f*,*h*]­quinoxaline derivative **3k**. This trend suggested that substitution at the *ortho* position to nitrogen significantly diminishes reactivity. To verify this hypothesis, we synthesized and evaluated a series of phosphinates **3d–g** bearing various substituents at the *ortho*, *meta*, and *para* positions to nitrogen. Indeed, derivatives **3d–g**_*ortho*_ exhibited no reactivity, regardless of the electronic nature of the substituents, including both the electron-donating (**3f**_*ortho*_) and the electron-withdrawing (**3g**_*ortho*_) groups. In some cases (**4d** *_meta_* and **4f**_*meta*_), substitution at the *meta* position resulted in only slightly reduced reaction rates, in comparison with that of unsubstituted **1a**, with no clear dependence on the electronic properties of the substituents. Finally, all *para*-substituted derivatives **3d–g**_*para*_ underwent cyclization smoothly. These findings confirm that steric hindrance at the *ortho* position to nitrogen (see **4d–g**_*ortho*_ and **4h**, **4i**, **4k**, and **4m** in Scheme 4) plays a crucial role in suppressing reactivity, while electronic effects have a negligible influence on the cyclization outcome. Finally, the potential for double cyclization was examined using bis-phosphinates **3l** and **3m**. While pyrazine-based derivative **3l** cyclized smoothly to afford the desired product **4l**, no reaction was observed for quinoline derivative **3m**, further emphasizing the sensitivity of the transformation to steric factors.

**Scheme 4:**
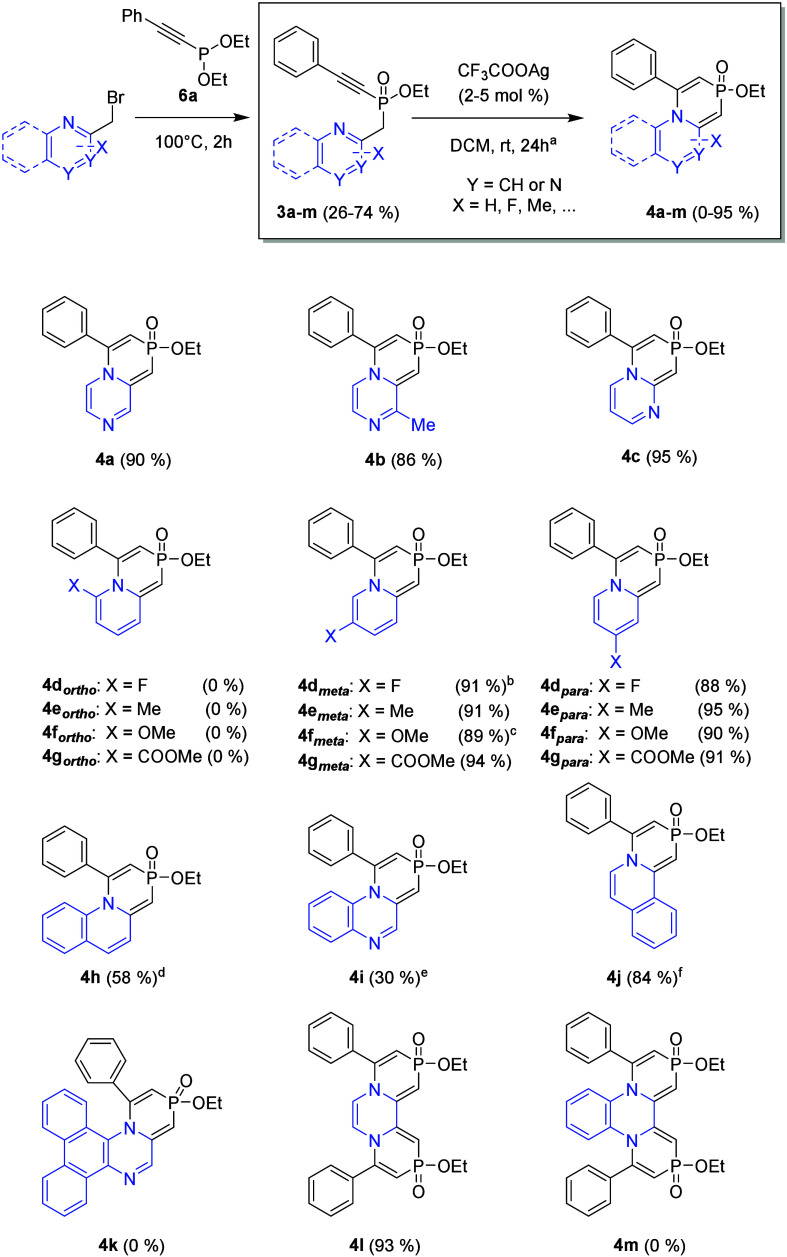
Reactivity of Phosphinates **3a–m** Bearing Substituted Pyridines and Other Nitrogen Heterocycles

a Unless otherwise stated.
b Full conversion was achieved after 3 days.
c Full conversion was achieved after 5 days.
d Full conversion was achieved after 13 days.
e Approximately 50% conversion was reached after 18 days.
f CCDC registration number 2489828.

For comparison of 1,4-azaphosphinine **2a** and its derivative **II** containing a carbon atom in place of nitrogen, the electrostatic potentials, HOMO/LUMO energies, and band gaps (*E*_g_) were calculated (using DFT calculations at the B3LYP/6-311G++(d,p) level of theory (Figure 3A,B)). The relative energies of the frontier MOs are shifted toward lower values in both the HOMO and the LUMO. The HOMO of derivative **2a** is decreased by more than 0.89 eV in comparison to that of **II**. The changes in energy levels of the FMOs together contribute to the decrease in the energy gap (*E*_g_) by almost 0.78 eV. More detailed analyses of the MOs are shown in . The attachment of additional π-systems, by annulation of extra benzene rings (**4h**–**j** and **4l**), leads to a decrease in HOMO energy by up to 0.3 eV, while the LUMO energy remains practically unchanged.

**Figure 3:**
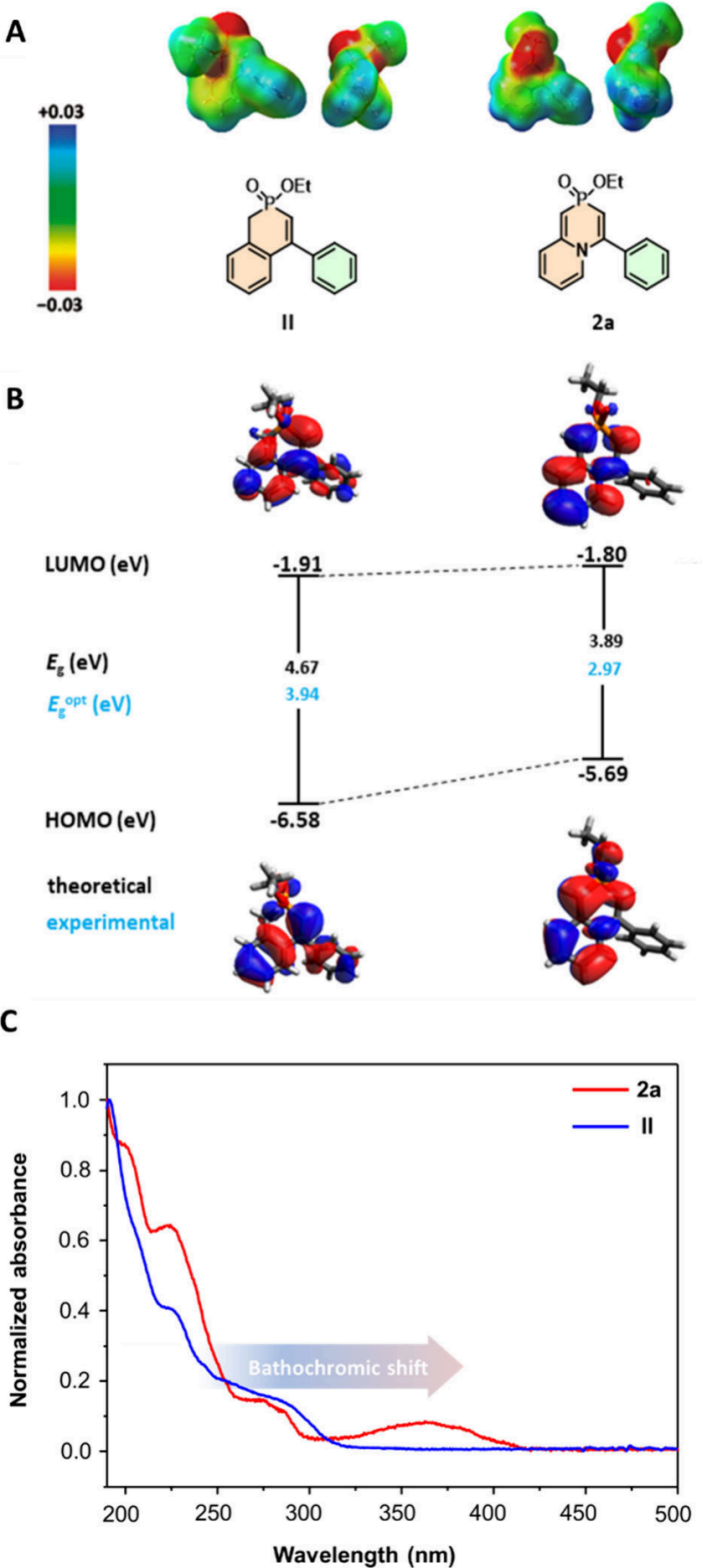
Illustration of the differences in the properties of carbo (**II**) and hetero (**2a**) analogues. (A) Electrostatic potentials mapped onto the electron density surface in the range from −0.03 (red) to 0.03 (blue). (B) Calculated HOMO/LUMO orbitals and energy gaps (*E*_g_) (DFT, B3LYP/6-311G++(d,p)) and experimental values of optical gap energies (blue). (C) Normalized UV–vis spectra of compounds **2a** and **II**.

The UV–vis absorption spectra of 1,4-azaphosphinine **2a** showed a significant bathochromic shift (ca. 60–120 nm) of the 0–0 transition compared with that of derivative **II** (Figure 3C), resulting in notably lower Δ*E* ^opt^ values of up to 0.97 eV. The observed red-shift and experimentally given values of the optical band gap are consistent with the DFT-calculated HOMO–LUMO energy gap as well as the simulated absorption spectra (UV–vis spectra and theoretical absorption spectra are provided in the , respectively). summarizes the main spectral features of the selected compounds in comparison with those of their carbo analogue **II**.

It is noteworthy that in contrast with recently reported π-extended azaphosphinine-based TADF-active systems,^17^ the present 1,4-azaphosphinines did not exhibit any detectable emission. The absence of emission suggests that the molecules undergo rapid nonradiative decay, which is further supported by the absence of measurable excitation spectra (not shown). The only exception is bis-phospha derivative **4l**, which likely benefits from conjugation involving both phosphinate moieties, partially restricting nonradiative relaxation. The excitation and emission spectra of **4l** are provided in .

## Conclusions

In summary, a series of intramolecular hydroarylation reactions of pyridyl phosphinates were developed, providing an efficient route to 1,4-azaphosphinines via catalytic dearomatization. Catalytic screening revealed that Ag­(I) salts, particularly CF_3_COOAg, are highly effective, promoting full conversion even at low catalyst loadings. The transformation proceeded smoothly across a wide range of substrates, demonstrating excellent functional group tolerance. Both electron-donating and electron-withdrawing substituents on the arylacetylene moiety were well tolerated, furnishing **2a**–**o** in excellent to very good yields. The reaction also performed well with additional aromatic rings (providing derivatives **2p**–**u**) and various nitrogen-containing heterocycles (**4a**–**m**). However, steric hindrance at the *ortho* position to nitrogen significantly reduced or even completely suppressed reactivity (**4h**, **4i**, **4k**, **4m**, and **4d–g**_*ortho*_). Double cyclization was achieved for pyrazine-based bis-phosphinate **4l**. Overall, this work provides a versatile and mild approach to so far unreported phosphorus heterocyclic frameworks, highlighting the crucial role of steric effects in controlling reactivity.

## Experimental Section

### Materials and Methods

^1^H, ^13^C­{^1^H}, ^19^F, ^31^P, and ^31^P­{^1^H} NMR spectra were recorded using a Bruker Avance 400 MHz instrument. Chemical shifts are reported in parts per million (δ) relative to TMS, CFCl_3_, and PPh_3_ (−6 ppm) or referenced to residuals of CDCl_3_ (δ = 7.26 and 77.16 ppm) or CD_2_Cl_2_ (δ = 5.30 and 54.00 ppm). The coupling constants (*J*) are given in hertz. The HMBC experiments were set up for *J*_C–H_ = 5 Hz. For the correct assignment of the ^1^H and ^13^C NMR spectra of key compounds, COSY, HSQC, and HMBC experiments were performed. GC–MS analyses were performed on an Agilent 6890 gas chromatograph coupled to an Agilent 5973 mass spectrometer operating in 70 eV ionization mode. A DB-5MS column (30 m × 0.25 mm × 0.25 μm) was used with He as a carrier gas at a flow rate of 1.0 mL/min. The initial temperature of 50 °C was held for 3 min and increased at a rate of 10 °C/min to 290 or 310 °C. The injection port was set at 250, 300, or 310 °C, depending on the volatility of the sample, and the *m*/*z* values are given along with their relative intensities (percent). For exact mass measurement, the spectra were internally calibrated using Na formate or tuning mix APCI-TOF. ESI and APCI high-resolution mass spectra were measured in positive mode by a micrOTOF QIII mass spectrometer (Bruker) and determined by Compass Data Analysis software. TLC was performed on silica gel 60 F254-coated aluminum sheets, and compounds were visualized with UV light (254 and 366 nm). Column chromatography was performed on an HPFC Biotage Isolera One system with prepacked flash silica gel columns with KP-Sil silica cartridges (0.040–0.063 mm). Absorption spectra were recorded using a DSM172 spectrophotometer (Online Instrument Systems, Inc.) in the wavelength range of 190–650 nm. Data were acquired at 20 °C (using a Peltier cell), with a data interval of 0.5 nm, a bandwidth of 1 nm, and a DIT of 5 ms. Fluorescence spectra of **4l** in excitation and emission modes were recorded in a quartz cuvette with a 1 cm optical path using a FP-8300 spectrofluorometer (JASCO) controlled by Spectra Manager II. Data were acquired at room temperature under a nitrogen (5.0) atmosphere using the following measurement conditions: excitation and emission bandwidths of 5 nm, scanning speed of 100 nm/min, and a data interval of 0.5 nm. The standard Schlenk technique was used for all reactions. Dichloromethane was freshly distilled from calcium hydride under an argon atmosphere. Other solvents were of HPLC quality. Commercially available reagents were purchased from Merck or Fluorochem and used as received.

### General Procedure for the Preparation of Phosphonites **6a–o** (GP1_A_)

The corresponding phenylacetylene (1 equiv) was dissolved in tetrahydrofuran (0.17 M) under an argon atmosphere and cooled to −78 °C. A 2.5 M solution of *n*-butyllithium in hexane (1 equiv) was then added (LiHMDS was used as a base instead of *n*-BuLi in the synthesis of bromo- and cyano-substituted derivatives **6h** and **6i**, respectively), and the reaction mixture was stirred at −78 °C for 15 min. Subsequently, diethyl chlorophosphite (1 equiv) was introduced, and the reaction was allowed to proceed at 60 °C for 1 h. Following the completion of the reaction, tetrahydrofuran was evaporated, and the residue was dissolved in dichloromethane (10 mL). The resulting mixture was filtered to remove insoluble salts, and the solvent was evaporated to afford phosphonites **6a–o** as a brown liquid. These crude products were obtained in approximately 80% purity, as determined by GC-MS analysis, and used in subsequent reactions without further purification.

### General Procedure for the Preparation of Phosphinates **1a–o** (GP2_A_)

2-Bromomethylpyridine hydrobromide (1 equiv) was treated with a 10% aqueous solution of K_2_CO_3_ to achieve a basic pH. The resulting aqueous phase was extracted with dichloromethane (3 × 10 mL/mmol), and the combined organic layers were dried over anhydrous MgSO_4_, filtered, and then mixed with phosphonites **6a–o** (1 equiv). The solvent was subsequently distilled off at atmospheric pressure, and the resulting oily residue was heated at 100 °C in an oil bath for approximately 2 h. Upon consumption of the starting materials, as monitored by GC-MS, the crude mixture was purified by column chromatography to afford the desired phosphinates **1a–o**.

### General Procedure for the Preparation of 1,4-Azaphosphinines **2a–o** (GP3_A_)

A Schlenk flask was charged with phosphinate **1a**–**o** (1 equiv), silver­(I) trifluoroacetate (0.02–0.05 equiv), and dry DCM (0.05–0.15 M) under an inert atmosphere. The resulting mixture was stirred overnight. Upon consumption of the starting material, as monitored by GC-MS or TLC, the crude product was purified by column chromatography to afford the desired 1,4-azaphosphinines **2a–o** as an oil, which typically solidified over time or upon treatment with EtOAc.

#### Ethyl (Phenylethynyl)­(pyridin-2-ylmethyl)­phosphinate (**1a**)

First, GP1_A_ was followed to generate crude phosphonite **6a** by reacting ethynylbenzene (1.24 mL, 11.25 mmol) with *n*-butyllithium (4.50 mL, 2.5 M in *n*-hexane, 11.25 mmol), followed by the addition of diethyl chlorophosphite (1.62 mL, 11.25 mmol). In the second step, GP2_A_ was employed using crude **6a** and 2-(bromomethyl)­pyridine hydrobromide (2.85 g, 11.25 mmol). The resulting product was purified by column chromatography (CHCl_3_/EtOAc/MeOH, gradient from 50:50:0 to 48:48:4), yielding 1.284 g of phosphinate **1a** (4.50 mmol, 40% over two steps) as a brown oil: ^1^H NMR (400 MHz, CDCl_3_) δ 8.62–8.53 (m, 1H), 7.66 (tdd, *J* = 7.6, 1.9, 0.8 Hz, 1H), 7.51–7.39 (m, 4H), 7.39–7.30 (m, 2H), 7.23–7.15 (m, 1H), 4.36–4.15 (m, 2H), 3.62 (d, *J* = 20.5 Hz, 2H), 1.36 (t, *J* = 7.0 Hz, 3H); ^13^C­{^1^H} NMR (101 MHz, CDCl_3_) δ 151.9 (d, *J* = 8.2 Hz), 149.7 (d, *J* = 2.9 Hz), 136.7 (d, *J* = 3.0 Hz), 132.7 (d, *J* = 2.1 Hz, 2C), 130.8, 128.6 (2C), 124.9 (d, *J* = 4.8 Hz), 122.2 (d, *J* = 3.3 Hz), 119.7 (d, *J* = 4.2 Hz), 101.7 (d, *J* = 37.1 Hz), 80.8 (d, *J* = 205.6 Hz), 62.7 (d, *J* = 7.4 Hz), 41.9 (d, *J* = 114.2 Hz), 16.4 (d, *J* = 6.6 Hz); ^31^P­{^1^H} NMR (162 MHz, CDCl_3_) δ 16.84; *R_f_* = 0.45 (93:7 CHCl_3_/MeOH); EI MS 285 (5%, M^+^), 284 (16%), 241 (56%), 192 (100%), 165 (57%), 156 (30%), 123 (17%), 102 (34%), 93 (60%), 65 (23%); HRMS (ESI/QTOF) *m*/*z* [M + H]^+^ calcd for [C_16_H_17_NO_2_P]^+^ 286.0991, found 286.0991 (100%).

#### Ethyl ((4-Methoxyphenyl)­ethynyl)­(pyridin-2-ylmethyl)­phosphinate (**1b**)

First, GP1_A_ was followed to generate crude phosphonite **6b** by reacting 1-ethynyl-4-methoxybenzene (0.49 mL, 3.79 mmol) with *n*-butyllithium (1.51 mL, 2.5 M in *n*-hexane, 3.79 mmol), followed by the addition of diethyl chlorophosphite (0.545 mL, 3.79 mmol). In the second step, GP2_A_ was employed using crude **6b** and 2-(bromomethyl)­pyridine hydrobromide (0.958 g, 3.79 mmol). The resulting product was purified by column chromatography (CHCl_3_/EtOAc/MeOH, gradient from 50:50:0 to 48:48:4), yielding 0.335 g of phosphinate **1b** (1.06 mmol, 28% over two steps) as a yellow oil: ^1^H NMR (400 MHz, CDCl_3_) δ 8.61–8.54 (m, 1H), 7.65 (td, *J* = 7.5, 1.5 Hz, 1H), 7.48–7.38 (m, 3H), 7.19 (dddd, *J* = 7.4, 4.3, 2.2, 1.1 Hz, 1H), 6.85 (d, *J* = 8.8 Hz, 2H), 4.32–4.14 (m, 2H), 3.83 (s, 3H), 3.61 (d, *J*_P–H_ = 20.4 Hz, 2H), 1.35 (t, *J* = 7.0 Hz, 3H); ^13^C­{^1^H} NMR (101 MHz, CDCl_3_) δ 161.4, 152.0 (d, *J* = 8.3 Hz), 149.6 (d, *J* = 2.7 Hz), 136.4 (d, *J* = 3.1 Hz), 134.3 (d, *J* = 2.2 Hz), 124.7 (d, *J* = 4.9 Hz), 122.0 (d, *J* = 3.4 Hz), 114.2, 111.5 (d, *J* = 4.7 Hz), 102.3 (d, *J* = 37.9 Hz), 79.7 (d, *J* = 208.7 Hz), 62.4 (d, *J* = 7.2 Hz), 55.4, 41.9 (d, *J* = 113.3 Hz), 16.3 (d, *J* = 6.8 Hz); ^31^P­{^1^H} NMR (162 MHz, CDCl_3_) δ 17.08; *R_f_* = 0.13 (EtOAc); EI MS 315 (10%, M^+^), 224 (15%), 200 (100%), 195 (20%), 132 (50%), 123 (40%), 93 (40%); HRMS (ESI/QTOF) *m*/*z* [M + H]^+^ calcd for [C_17_H_19_NO_3_P]^+^ 316.1097, found 316.1091 (100%).

#### Ethyl ((2-Methoxyphenyl)­ethynyl)­(pyridin-2-ylmethyl)­phosphinate (**1c**)

First, GP1_A_ was followed to generate crude phosphonite **6c** by reacting 1-ethynyl-2-methoxybenzene (1.00 mL, 7.73 mmol) with *n*-butyllithium (3.09 mL, 2.5 M in *n*-hexane, 7.73 mmol), followed by the addition of diethyl chlorophosphite (1.11 mL, 7.73 mmol). In the second step, GP2_A_ was employed using crude **6c** and 2-(bromomethyl)­pyridine hydrobromide (1.96 g, 7.73 mmol). The resulting product was purified by column chromatography (CHCl_3_/EtOAc/MeOH, gradient from 50:50:0 to 48:48:4), yielding 0.850 g of phosphinate **1c** (2.70 mmol, 35% over two steps) as a brown oil: ^1^H NMR (400 MHz, CDCl_3_) δ 8.56 (dt, *J* = 5.1, 1.3 Hz, 1H), 7.64 (td, *J* = 7.7, 1.9 Hz, 1H), 7.49 (ddd, *J* = 7.8, 2.6, 1.3 Hz, 1H), 7.44–7.33 (m, 2H), 7.22–7.14 (m, 1H), 6.96–6.79 (m, 2H), 4.24 (dtt, *J* = 13.3, 9.5, 6.3 Hz, 2H), 3.85 (s, 3H), 3.63 (d, *J* = 20.4 Hz, 2H), 1.35 (t, *J* = 7.1 Hz, 3H); ^13^C­{^1^H} NMR (101 MHz, CDCl_3_) δ 161.5 (d, *J* = 1.5 Hz), 151.9 (d, *J* = 8.4 Hz), 149.6 (d, *J* = 2.6 Hz), 136.5 (d, *J* = 2.9 Hz), 134.4 (d, *J* = 2.2 Hz), 132.3, 124.8 (d, *J* = 4.7 Hz), 122.1 (d, *J* = 3.4 Hz), 120.5, 110.9, 109.0 (d, *J* = 4.5 Hz), 98.8 (d, *J* = 38.2 Hz), 84.6 (d, *J* = 207.3 Hz), 62.6 (d, *J* = 7.3 Hz), 55.8, 41.8 (d, *J* = 113.4 Hz), 16.2 (d, *J* = 6.6 Hz); ^31^P­{^1^H} NMR (162 MHz, CDCl_3_) δ 16.68; *R_f_* = 0.50 (92:8 CHCl_3_/MeOH); EI MS 315 (4%, M^+^), 286 (45%), 222 (41%), 156 (30%), 131 (100%), 93 (66%), 65 (21%); HRMS (ESI/QTOF) *m*/*z* [M + H]^+^ calcd for [C_17_H_19_NO_3_P]^+^ 316.1097, found 316.1099 (100%).

#### Ethyl ((3,4-Dimethoxyphenyl)­ethynyl)­(pyridin-2-ylmethyl)­phosphinate (**1d**)

First, GP1_A_ was followed to generate crude phosphonite **6d** by reacting 4-ethynyl-1,2-dimethoxybenzene (500 mg, 3.08 mmol) with *n*-butyllithium (1.23 mL, 2.5 M in *n*-hexane, 3.08 mmol), followed by the addition of diethyl chlorophosphite (0.443 mL, 3.08 mmol). In the second step, GP2_A_ was employed using crude **6d** and 2-(bromomethyl)­pyridine hydrobromide (731 mg, 3.08 mmol). The resulting product was purified by column chromatography (CHCl_3_/EtOAc/MeOH, gradient from 50:50:0 to 48:48:4), yielding 510 mg of phosphinate **1d** (1.48 mmol, 48% over two steps) as a brown oil: ^1^H NMR (400 MHz, CDCl_3_) δ 8.60–8.51 (m, 1H), 7.65 (td, *J* = 7.7, 1.8 Hz, 1H), 7.49–7.40 (m, 1H), 7.18 (dddd, *J* = 7.4, 5.0, 2.3, 1.2 Hz, 1H), 7.09 (dd, *J* = 8.3, 1.9 Hz, 1H), 6.94 (d, *J* = 1.9 Hz, 1H), 6.80 (d, *J* = 8.3 Hz, 1H), 4.33–4.13 (m, 2H), 3.89 (s, 3H), 3.86 (s, 3H), 3.61 (d, *J* = 20.4 Hz, 2H), 1.35 (t, *J* = 7.0 Hz, 3H); ^13^C­{^1^H} NMR (101 MHz, CDCl_3_) δ 151.8 (d, *J* = 8.4 Hz), 151.3, 149.4 (d, *J* = 2.6 Hz), 148.5, 136.3 (d, *J* = 3.0 Hz), 126.4 (d, *J* = 2.1 Hz), 124.6 (d, *J* = 4.8 Hz), 121.9 (d, *J* = 3.4 Hz), 114.6 (d, *J* = 2.0 Hz), 111.3 (d, *J* = 4.5 Hz), 110.8, 102.2 (d, *J* = 37.9 Hz), 79.3 (d, *J* = 207.7 Hz), 62.3 (d, *J* = 7.3 Hz), 55.8, 55.8, 41.8 (d, *J* = 113.2 Hz), 16.1 (d, *J* = 6.8 Hz); ^31^P­{^1^H} NMR (162 MHz, CDCl_3_) δ 17.04; *R_f_* = 0.43 (93:7 CHCl_3_/MeOH); EI MS 345 (24%, M^+^), 302 (37%), 254 (26%), 238 (31%), 225 (39%), 162 (46%), 154 (37%), 123 (67%), 93 (100%); HRMS (ESI/QTOF) *m*/*z* [M + H]^+^ calcd for [C_18_H_21_NO_4_P]^+^ 346.1203, found 346.1204 (100%).

#### Ethyl ((3,5-Dimethoxyphenyl)­ethynyl)­(pyridin-2-ylmethyl)­phosphinate (**1e**)

First, GP1_A_ was followed to generate crude phosphonite **6e** by reacting 1-ethynyl-3,5-dimethoxybenzene (2.00 g, 12.33 mmol) with *n*-butyllithium (4.93 mL, 2.5 M in *n*-hexane, 12.33 mmol), followed by the addition of diethyl chlorophosphite (1.77 mL, 12.33 mmol). In the second step, GP2_A_ was employed using crude **6e** and 2-(bromomethyl)­pyridine hydrobromide (3.12 g, 12.33 mmol). The resulting product was purified by column chromatography (CHCl_3_/EtOAc/MeOH, gradient from 50:50:0 to 48:48:4), yielding 1.60 g of phosphinate **1e** (4.63 mmol, 38% over two steps) as a brown oil: ^1^H NMR (400 MHz, CDCl_3_) δ 8.57 (d, *J* = 4.0 Hz, 1H), 7.73–7.62 (m, 1H), 7.52–7.38 (m, 1H), 7.21–7.02 (m, 1H), 6.60 (d, *J* = 2.3 Hz, 2H), 6.52 (t, *J* = 2.3 Hz, 1H), 4.39–4.09 (m, 2H), 3.77 (s, 6H), 3.61 (d, *J* = 20.6 Hz, 2H), 1.36 (t, *J* = 7.1 Hz, 3H); ^13^C­{^1^H} NMR (101 MHz, CDCl_3_) δ 160.5, 151.8 (d, *J* = 8.3 Hz), 149.6 (d, *J* = 2.7 Hz), 136.5 (d, *J* = 3.0 Hz), 124.7 (d, *J* = 4.8 Hz), 122.1 (d, *J* = 3.4 Hz), 120.8 (d, *J* = 4.4 Hz), 110.2 (d, *J* = 2.1 Hz, 2C), 104.0, 101.5 (d, *J* = 37.0 Hz), 80.2 (d, *J* = 204.7 Hz), 62.6 (d, *J* = 7.3 Hz), 55.5 (2C), 41.8 (d, *J* = 113.3 Hz), 16.2 (d, *J* = 6.7 Hz) (the signals corresponding to the two equivalent quaternary carbons could not be assigned with certainty); ^31^P­{^1^H} NMR (162 MHz, CDCl_3_) δ 16.86; *R_f_* = 0.43 (93:7 CHCl_3_/MeOH); EI MS 435 (44%, M^+^), 344 (100%), 301 (53%), 282 (39%), 254 (99%), 225 (70%), 210 (60%), 195 (37%), 156 (67%), 123 (54%), 93 (96%), 65 (34%); HRMS (ESI/QTOF) *m*/*z* [M + H]^+^ calcd for [C_18_H_21_NO_4_P]^+^ 346.1203, found 346.1204 (100%).

#### Ethyl ((4-Methylphenyl)­ethynyl)­(pyridin-2-ylmethyl)­phosphinate (**1f**)

First, GP1_A_ was followed to generate crude phosphonite **6f** by reacting 1-ethynyl-4-methylbenzene (230 mg, 1.98 mmol) with *n*-butyllithium (0.791 mL, 2.5 M in *n*-hexane, 1.98 mmol), followed by the addition of diethyl chlorophosphite (0.284 mL, 1.98 mmol). In the second step, GP2_A_ was employed using crude **6f** and 2-(bromomethyl)­pyridine hydrobromide (500 mg, 1.98 mmol). The resulting product was purified by column chromatography (CHCl_3_/EtOAc/MeOH, gradient from 50:50:0 to 48:48:4), yielding 229 mg of phosphinate **1f** (0.765 mmol, 39% over two steps) as a brown oil: ^1^H NMR (400 MHz, CDCl_3_) δ 8.59–8.53 (m, 1H), 7.65 (td, *J* = 7.6, 1.2 Hz, 1H), 7.48–7.41 (m, 1H), 7.35 (d, *J* = 8.2 Hz, 2H), 7.23–7.16 (m, 1H), 7.14 (d, *J* = 8.6 Hz, 2H), 4.31–4.13 (m, 2H), 3.61 (d, *J* = 20.4 Hz, 2H), 2.36 (s, 3H), 1.35 (t, *J* = 7.1 Hz, 3H); ^13^C­{^1^H} NMR (101 MHz, CDCl_3_) δ 151.7 (d, *J* = 8.3 Hz), 149.4 (d, *J* = 2.6 Hz), 141.2, 136.5 (d, *J* = 3.0 Hz), 132.4 (d, *J* = 2.0 Hz, 2C), 129.2 (2C), 124.7 (d, *J* = 4.8 Hz), 122.1 (d, *J* = 3.4 Hz), 116.4 (d, *J* = 4.5 Hz), 102.2 (d, *J* = 37.6 Hz), 80.1 (d, *J* = 207.3 Hz), 62.5 (d, *J* = 7.3 Hz), 41.7 (d, *J* = 113.2 Hz), 21.6, 16.2 (d, *J* = 6.7 Hz); ^31^P­{^1^H} NMR (162 MHz, CDCl_3_) δ 16.90; *R_f_* = 0.69 (93:7 CHCl_3_/MeOH); EI MS 299 (10%, M^+^) 255 (37%), 207 (100%), 179 (49%), 156 (31%), 115 (46%), 93 (58%), 65 (24%); HRMS (ESI/QTOF) *m*/*z* [M + H]^+^ calcd for [C_17_H_19_NO_2_P]^+^ 300.1148, found 300.1150 (100%).

#### Ethyl ((4-(*tert*-Butyl)­phenyl)­ethynyl)­(pyridin-2-ylmethyl)­phosphinate (**1g**)

First, GP1_A_ was followed to generate crude phosphonite **6g** by reacting 1-(*tert*-butyl)-4-ethynylbenzene (313 mg, 1.98 mmol) with *n*-butyllithium (0.791 mL, 2.5 M in *n*-hexane, 1.98 mmol), followed by the addition of diethyl chlorophosphite (0.284 mL, 1.98 mmol). In the second step, GP2_A_ was employed using crude **6g** and 2-(bromomethyl)­pyridine hydrobromide (500 mg, 1.98 mmol). The resulting product was purified by column chromatography (CHCl_3_/EtOAc/MeOH, gradient from 50:50:0 to 48:48:4), yielding 236 mg of phosphinate **1g** (0.692 mmol, 35% over two steps) as a brown oil: ^1^H NMR (400 MHz, CDCl_3_) δ 8.61–8.52 (m, 1H), 7.65 (tdd, *J* = 7.6, 1.9, 0.8 Hz, 1H), 7.46–7.34 (m, 5H), 7.22–7.15 (m, 1H), 4.31–4.13 (m, 2H), 3.61 (d, *J* = 20.5 Hz, 2H), 1.35 (t, *J* = 7.0 Hz, 3H), 1.30 (s, 9H); ^13^C­{^1^H} NMR (101 MHz, CDCl_3_) δ 154.4, 152.0 (d, *J* = 8.4 Hz), 149.7 (d, *J* = 2.6 Hz), 136.5 (d, *J* = 3.1 Hz), 132.4 (d, *J* = 1.9 Hz, 2C), 125.6 (2C), 124.8 (d, *J* = 4.8 Hz), 122.1 (d, *J* = 3.4 Hz), 116.6 (d, *J* = 4.4 Hz), 102.1 (d, *J* = 37.6 Hz), 80.2 (d, *J* = 206.9 Hz), 62.6 (d, *J* = 7.3 Hz), 41.9 (d, *J* = 113.3 Hz), 35.1, 31.1 (3C), 16.3 (d, *J* = 6.7 Hz); ^31^P­{^1^H} NMR (162 MHz, CDCl_3_) δ 16.99; *R_f_* = 0.52 (93:7 CHCl_3_/MeOH); EI MS 341 (16%, M^+^), 297 (26%), 250 (42%), 234 (100%), 221 (24%), 156 (51%), 123 (44%), 93 (81%); HRMS (ESI/QTOF) *m*/*z* [M + H]^+^ calcd for [C_20_H_25_NO_2_P]^+^ 342.1617, found 342.1618 (100%).

#### Ethyl ((4-Bromophenyl)­ethynyl)­(pyridin-2-ylmethyl)­phosphinate (**1h**)

First, GP1_A_ was followed to generate crude phosphonite **6h** by reacting 1-bromo-4-ethynylbenzene (1.00 g, 5.52 mmol) with LiHMDS (5.52 mL, 1 M in THF, 5.52 mmol), followed by the addition of diethyl chlorophosphite (0.794 mL, 5.52 mmol). In the second step, GP2_A_ was employed using crude **6h** and 2-(bromomethyl)­pyridine hydrobromide (1397 mg, 5.52 mmol). The resulting product was purified by column chromatography (CHCl_3_/EtOAc/MeOH, gradient from 50:50:0 to 48:48:4), yielding 600 mg of phosphinate **1h** (1.65 mmol, 30% over two steps) as a brown oil: ^1^H NMR (400 MHz, CDCl_3_) δ 8.62–8.47 (m, 1H), 7.66 (td, *J* = 7.7, 1.9 Hz, 1H), 7.49 (d, *J* = 8.5 Hz, 2H), 7.45–7.38 (m, 1H), 7.31 (d, *J* = 8.5 Hz, 2H), 7.19 (dddd, *J* = 7.4, 4.9, 2.4, 1.2 Hz, 1H), 4.33–4.12 (m, 2H), 3.61 (d, *J* = 20.6 Hz, 2H), 1.35 (t, *J* = 7.1 Hz, 3H); ^13^C­{^1^H} NMR (101 MHz, CDCl_3_) δ 151.7 (d, *J* = 8.4 Hz), 149.7 (d, *J* = 2.7 Hz), 136.6 (d, *J* = 3.0 Hz), 133.9 (d, *J* = 2.0 Hz, 2C), 132.0 (2C), 125.5, 124.8 (d, *J* = 4.9 Hz), 122.2 (d, *J* = 3.4 Hz), 118.6 (d, *J* = 4.5 Hz), 100.3 (d, *J* = 36.8 Hz), 82.0 (d, *J* = 203.2 Hz), 62.7 (d, *J* = 7.4 Hz), 41.8 (d, *J* = 113.5 Hz), 16.3 (d, *J* = 6.7 Hz); ^31^P­{^1^H} NMR (162 MHz, CDCl_3_) δ 16.79; *R_f_* = 0.62 (92:8 CHCl_3_/MeOH); EI MS 363 (8%, M^+^) 319 (62%), 272 (81%), 245 (37%), 191 (100%), 180 (34%), 156 (44%), 123 (30%), 93 (99%), 65 (61%); HRMS (APCI/QTOF) *m*/*z* [M + H]^+^ calcd for [C_16_H_16_^79^BrNO_2_P]^+^ 364.0097, found 364.0095 (100%).

#### Ethyl ((4-Chlorophenyl)­ethynyl)­(pyridin-2-ylmethyl)­phosphinate (**1i**)

First, GP1_A_ was followed to generate crude phosphonite **6i** by reacting 1-chloro-4-ethynylbenzene (1.50 g, 10.9 mmol) with *n*-butyllithium (4.39 mL, 2.5 M in *n*-hexane, 10.9 mmol), followed by the addition of diethyl chlorophosphite (1.58 mL, 10.9 mmol). In the second step, GP2_A_ was employed using crude **6i** and 2-(bromomethyl)­pyridine hydrobromide (2.78 g, 10.9 mmol). The resulting product was purified by column chromatography (CHCl_3_/EtOAc/MeOH, gradient from 50:50:0 to 48:48:4), yielding 960 mg of phosphinate **1i** (3.00 mmol, 27% over two steps) as a brown oil: ^1^H NMR (400 MHz, CDCl_3_) δ 8.62–8.50 (m, 1H), 7.66 (tdd, *J* = 7.7, 1.9, 0.8 Hz, 1H), 7.42 (ddd, *J* = 7.9, 2.5, 1.2 Hz, 1H), 7.39 (d, *J* = 8.6 Hz, 2H), 7.33 (d, *J* = 8.7 Hz, 2H), 7.19 (dddd, *J* = 7.4, 4.9, 2.3, 1.1 Hz, 1H), 4.30–4.14 (m, 2H), 3.61 (d, *J* = 20.6 Hz, 2H), 1.36 (t, *J* = 7.0 Hz, 3H); ^13^C­{^1^H} NMR (101 MHz, CDCl_3_) δ 151.6 (d, *J* = 8.3 Hz), 149.6 (d, *J* = 2.8 Hz), 136.9, 136.4 (d, *J* = 3.0 Hz), 133.6 (d, *J* = 2.1 Hz, 2C), 128.9 (2C), 124.6 (d, *J* = 4.9 Hz), 122.1 (d, *J* = 3.4 Hz), 118.0 (d, *J* = 4.6 Hz), 100.1 (d, *J* = 36.7 Hz), 81.8 (d, *J* = 203.2 Hz), 62.5 (d, *J* = 7.4 Hz), 41.7 (d, *J* = 113.4 Hz), 16.2 (d, *J* = 6.7 Hz); ^31^P­{^1^H} NMR (162 MHz, CDCl_3_) δ 16.78; *R_f_* = 0.65 (93:7 CHCl_3_/MeOH); EI MS 319 (7%, M^+^), 275 (53%), 227 (100%), 191 (58%), 156 (38%), 136 (57%), 123 (37%), 93 (88%), 65 (40%); HRMS (ESI/QTOF) *m*/*z* [M + H]^+^ calcd for [C_16_H_16_ClNO_2_P]^+^ 320.0602, found 320.0603 (100%).

#### Ethyl ((4-Fluorophenyl)­ethynyl)­(pyridin-2-ylmethyl)­phosphinate (**1j**)

First, GP1_A_ was followed to generate crude phosphonite **6j** by reacting 1-ethynyl-4-fluorobenzene (0.48 mL, 4.16 mmol) with *n*-butyllithium (1.67 mL, 2.5 M in *n*-hexane, 4.16 mmol), followed by the addition of diethyl chlorophosphite (0.60 mL, 4.16 mmol). In the second step, GP2_A_ was employed using crude **6j** and 2-(bromomethyl)­pyridine hydrobromide (1.053 g, 4.16 mmol). The resulting product was purified by column chromatography (CHCl_3_/EtOAc/MeOH, gradient from 50:50:0 to 48:48:4), yielding 353 mg of phosphinate **1j** (1.17 mmol, 28% over two steps) as a yellow oil: ^1^H NMR (400 MHz, CDCl_3_) δ 8.55 (dd, *J* = 4.9, 1.0 Hz, 1H), 7.65 (td, *J* = 7.7, 1.1 Hz, 1H), 7.50–7.39 (m, 3H), 7.22–7.15 (m, 1H), 7.08–6.99 (m, 2H), 4.31–4.11 (m, 2H), 3.60 (d, *J*_P–H_ = 20.6 Hz, 2H), 1.35 (t, *J* = 7.0 Hz, 3H); ^13^C­{^1^H} NMR (101 MHz, CDCl_3_) δ 164.0 (d, *J* = 253.7 Hz), 151.8 (d, *J* = 8.3 Hz), 149.7 (d, *J* = 2.7 Hz), 136.7 (d, *J* = 3.0 Hz), 134.9 (dd, *J* = 8.9, 2.1 Hz), 124.9 (d, *J* = 4.8 Hz), 122.3 (d, *J* = 3.4 Hz), 116.2 (d, *J* = 22.4 Hz), 115.9–115.8 (m), 100.6 (d, *J* = 37.3 Hz), 80.8 (dd, *J* = 205.0, 1.6 Hz), 62.7 (d, *J* = 7.4 Hz), 41.9 (d, *J* = 113.5 Hz), 16.4 (d, *J* = 6.7 Hz); ^31^P­{^1^H} NMR (162 MHz, CDCl_3_) δ 16.86; ^19^F NMR (376 MHz, CDCl_3_) δ −106.21; *R_f_* = 0.15 (EtOAc); EI MS 303 (2%, M^+^), 302 (5%), 259 (50%), 211 (100%), 183 (50%), 156 (20%), 120 (45%), 93 (60%), 65 (40%); HRMS (ESI/QTOF) *m*/*z* [M + H]^+^ calcd for [C_16_H_16_FNO_2_P]^+^ 304.0897, found 304.0896 (100%).

#### Ethyl ((3,4-Difluorophenyl)­ethynyl)­(pyridin-2-ylmethyl)­phosphinate (**1k**)

First, GP1_A_ was followed to generate crude phosphonite **6k** by reacting 4-ethynyl-1,2-difluorobenzene (1.00 mL, 8.25 mmol) with *n*-butyllithium (3.30 mL, 2.5 M in *n*-hexane, 8.25 mmol), followed by the addition of diethyl chlorophosphite (1.19 mL, 8.25 mmol). In the second step, GP2_A_ was employed using crude **6k** and 2-(bromomethyl)­pyridine hydrobromide (2.09 g, 8.25 mmol). The resulting product was purified by column chromatography (CHCl_3_/EtOAc/MeOH, gradient from 50:50:0 to 48:48:4), yielding 567 mg of phosphinate **1k** (1.77 mmol, 21% over two steps) as a brown oil: ^1^H NMR (400 MHz, CDCl_3_) 8.57 (d, *J* = 4.2 Hz, 1H), 7.66 (td, *J* = 7.7, 2.4 Hz, 1H), 7.41 (dd, *J* = 7.9, 2.8 Hz, 1H), 7.30–7.10 (m, 4H), 4.34–4.10 (m, 2H), 3.60 (d, *J* = 20.7 Hz, 2H), 1.36 (t, *J* = 7.1 Hz, 3H); ^13^C­{^1^H} NMR (101 MHz, CDCl_3_) δ 152.4 (dd, *J* = 212.6, 13.0 Hz), 151.7 (d, *J* = 8.2 Hz), 149.9 (dd, *J* = 208.1, 13.0 Hz), 149.8 (d, *J* = 2.8 Hz), 136.7 (d, *J* = 3.0 Hz), 130.4–129.1 (m), 124.9 (d, *J* = 5.1 Hz), 122.4 (d, *J* = 3.6 Hz), 121.7 (d, *J* = 19.1 Hz), 118.1 (d, *J* = 18.3 Hz), 116.6 (d, *J* = 7.5 Hz), 99.0 (d, *J* = 36.5 Hz), 81.6 (d, *J* = 202.1 Hz), 62.8 (d, *J* = 7.4 Hz), 41.9 (d, *J* = 113.5 Hz), 16.4 (d, *J* = 6.7 Hz); ^31^P­{^1^H} NMR (162 MHz, CDCl_3_) δ 16.69; ^19^F­{^1^H} NMR (376 MHz, CDCl_3_) δ −131.17 (d, *J* = 21.3 Hz), −135.66 (d, *J* = 21.2 Hz); *R_f_* = 0.56 (92:8 CHCl_3_/MeOH); EI MS 321 (2%, M^+^), 320 (8%), 277 (53%), 229 (100%), 201 (42%), 156 (21%), 138 (37%), 93 (58%), 65 (30%); HRMS (ESI/QTOF) *m*/*z* [M + H]^+^ calcd for [C_16_H_15_F_2_NO_2_P]^+^ 322.0803, found 322.0803 (100%).

#### Ethyl ((3,5-Difluorophenyl)­ethynyl)­(pyridin-2-ylmethyl)­phosphinate (**1l**)

First, GP1_A_ was followed to generate crude phosphonite **6l** by reacting 1-ethynyl-3,5-difluorobenzene (1.00 mL, 8.42 mmol) with *n*-butyllithium (3.37 mL, 2.5 M in *n*-hexane, 8.42 mmol), followed by the addition of diethyl chlorophosphite (1.21 mL, 8.42 mmol). In the second step, GP2_A_ was employed using crude **6l** and 2-(bromomethyl)­pyridine hydrobromide (2.13 g, 8.42 mmol). The resulting product was purified by column chromatography (CHCl_3_/EtOAc/MeOH, gradient from 50:50:0 to 48:48:4), yielding 435 mg of phosphinate **1l** (1.35 mmol, 16% over two steps) as a brown oil: ^1^H NMR (400 MHz, CDCl_3_) δ 8.58 (dd, *J* = 4.0, 0.9 Hz, 1H), 7.67 (tdd, *J* = 7.7, 1.9, 0.8 Hz, 1H), 7.47–7.36 (m, 1H), 7.25–7.16 (m, 1H), 7.01–6.94 (m, 2H), 6.90 (tt, *J* = 8.8, 2.3 Hz, 1H), 4.36–4.13 (m, 2H), 3.61 (d, *J* = 20.8 Hz, 2H), 1.37 (t, *J* = 7.0 Hz, 3H); ^13^C­{^1^H} NMR (101 MHz, CDCl_3_) δ 162.5 (dd, *J* = 250.8, 13.0 Hz, 2C), 151.4 (d, *J* = 8.4 Hz), 149.6 (d, *J* = 2.7 Hz), 136.7 (d, *J* = 3.1 Hz), 124.8 (d, *J* = 5.0 Hz), 122.3 (d, *J* = 3.5 Hz), 115.5 (dd, *J* = 11.5, 2.1 Hz), 115.5 (dd, *J* = 27.5, 2.1 Hz, 2C), 107.0 (t, *J* = 25.2 Hz), 98.1 (d, *J* = 35.9 Hz), 82.6 (d, *J* = 199.5 Hz), 62.8 (d, *J* = 7.4 Hz), 41.6 (d, *J* = 113.7 Hz), 16.2 (d, *J* = 6.6 Hz); ^31^P­{^1^H} NMR (162 MHz, CDCl_3_) δ 16.54; ^19^F NMR (376 MHz, CDCl_3_) δ −108.01; *R_f_* = 0.58 (92:8 CHCl_3_/MeOH); EI MS 321 (3%, M^+^), 320 (11%), 277 (59%), 229 (100%), 201 (43%), 156 (30%), 138 (34%), 93 (78%), 65 (40%); HRMS (ESI/QTOF) *m*/*z* [M + H]^+^ calcd for [C_16_H_15_F_2_NO_2_P]^+^ 322.0803, found 322.0802 (100%).

#### Ethyl ((4-Cyanophenyl)­ethynyl)­(pyridin-2-ylmethyl)­phosphinate (**1m**)

First, GP1_A_ was followed to generate crude phosphonite **6m** by reacting 4-ethynylbenzonitrile (1.50 g, 11.8 mmol) with LiHMDS (11.8 mL, 1 M in THF, 11.8 mmol), followed by the addition of diethyl chlorophosphite (1.70 mL, 11.8 mmol). In the second step, GP2_A_ was employed using crude **6m** and 2-(bromomethyl)­pyridine hydrobromide (2.98 g, 11.8 mmol). The resulting product was purified by column chromatography (CHCl_3_/EtOAc/MeOH, gradient from 50:50:0 to 48:48:4), yielding 725 mg of phosphinate **1m** (2.34 mmol, 20%) as a brown oil: ^1^H NMR (400 MHz, CDCl_3_) δ 8.62–8.50 (m, 1H), 7.71–7.60 (m, 3H), 7.55 (d, *J* = 8.4 Hz, 2H), 7.41 (dd, *J* = 7.7, 2.5 Hz, 1H), 7.25–7.16 (m, 1H), 4.40–4.05 (m, 2H), 3.62 (d, *J* = 20.7 Hz, 2H), 1.36 (t, *J* = 7.1 Hz, 3H); ^13^C­{^1^H} NMR (101 MHz, CDCl_3_) δ 151.5 (d, *J* = 8.3 Hz), 149.8 (d, *J* = 2.7 Hz), 136.8 (d, *J* = 3.0 Hz), 133.1 (d, *J* = 2.1 Hz, 2C), 132.3 (2C), 124.9 (d, *J* = 5.0 Hz), 125.0 (d, *J* = 4.3 Hz), 122.4 (d, *J* = 3.6 Hz), 117.9, 114.2, 98.7 (d, *J* = 35.6 Hz), 84.8 (d, *J* = 198.5 Hz), 63.0 (d, *J* = 7.4 Hz), 41.8 (d, *J* = 113.9 Hz), 16.4 (d, *J* = 6.7 Hz); ^31^P­{^1^H} NMR (162 MHz, CDCl_3_) δ 16.52; *R_f_* = 0.75 (93:7 CHCl_3_/MeOH); EI MS 310 (5%, M^+^), 309 (8%), 266 (64%), 247 (16%), 218 (100%), 190 (40%), 127 (26%), 93 (55%), 65 (32%); HRMS (ESI/QTOF) *m*/*z* [M + H]^+^ calcd for [C_17_H_16_N_2_O_2_P]^+^ 311.0944, found 311.0949 (100%).

#### Ethyl (Pyridin-2-ylmethyl)­((4-(trifluoromethyl)­phenyl)­ethynyl)­phosphinate (**1n**)

First, GP1_A_ was followed to generate crude phosphonite **6n** by reacting 1-ethynyl-4-(trifluoromethyl)­benzene (500 mg, 2.94 mmol) with *n*-butyllithium (1.17 mL, 2.5 M in *n*-hexane, 2.94 mmol), followed by the addition of diethyl chlorophosphite (0.422 mL, 2.94 mmol). In the second step, GP2_A_ was employed using crude **6n** and 2-(bromomethyl)­pyridine hydrobromide (743 mg, 2.94 mmol). The resulting product was purified by column chromatography (CHCl_3_/EtOAc/MeOH, gradient from 50:50:0 to 48:48:4), yielding 85.0 mg of phosphinate **1n** (0.241 mmol, 8% over two steps) as a brown oil: ^1^H NMR (400 MHz, CDCl_3_) δ 8.56 (d, *J* = 4.3 Hz, 1H), 7.66 (td, *J* = 7.8, 1.9 Hz, 1H), 7.60 (d, *J* = 8.4 Hz, 2H), 7.56 (d, *J* = 8.3 Hz, 2H), 7.42 (ddd, *J* = 7.9, 2.6, 1.2 Hz, 1H), 7.24–7.15 (m, 1H), 4.33–4.14 (m, 2H), 3.62 (d, *J* = 20.7 Hz, 2H), 1.36 (t, *J* = 7.1 Hz, 3H); ^13^C­{^1^H} NMR (101 MHz, CDCl_3_) δ 151.6 (d, *J* = 8.3 Hz), 149.8 (d, *J* = 2.8 Hz), 136.7 (d, *J* = 3.1 Hz), 132.9 (d, *J* = 2.1 Hz, 2C), 132.3 (d, *J* = 33.0 Hz), 125.6 (q, *J* = 3.8 Hz, 2C), 124.9 (d, *J* = 5.0 Hz), 123.5, 122.4 (d, *J* = 3.4 Hz), 99.4 (d, *J* = 35.9 Hz), 83.1 (d, *J* = 200.6 Hz), 62.9 (d, *J* = 7.4 Hz), 41.8 (d, *J* = 113.6 Hz), 16.4 (d, *J* = 6.6 Hz) (the signal of the CF_3_ group could not be unambiguously assigned due to its low intensity and overlap with other signals); ^31^P­{^1^H} NMR (162 MHz, CDCl_3_) δ 16.84; ^19^F­{^1^H} NMR (376 MHz, CDCl_3_) δ −63.19; *R_f_* = 0.55 (93:7 CHCl_3_/MeOH); EI MS 353 (7%, M^+^), 352 (14%), 334 (13%), 309 (70%), 290 (21%), 261 (100%), 233 (31%), 191 (19%), 170 (19%), 156 (19%), 93 (57%), 65 (23%); HRMS (ESI/QTOF) *m*/*z* [M + H]^+^ calcd for [C_17_H_16_F_3_NO_2_P]^+^ 354.0865, found 354.0866 (100%).

#### Ethyl (Pyridin-2-ylmethyl)­((2-(trifluoromethyl)­phenyl)­ethynyl)­phosphinate (**1o**)

First, GP1_A_ was followed to generate crude phosphonite **6o** by reacting 1-ethynyl-2-(trifluoromethyl)­benzene (500 mg, 2.94 mmol) with *n*-butyllithium (1.17 mL, 2.5 M in *n*-hexane, 2.94 mmol), followed by the addition of diethyl chlorophosphite (0.422 mL, 2.94 mmol). In the second step, GP2_A_ was employed using crude **6o** and 2-(bromomethyl)­pyridine hydrobromide (743 mg, 2.94 mmol). The resulting product was purified by column chromatography (CHCl_3_/EtOAc/MeOH, gradient from 50:50:0 to 48:48:4), yielding 63.0 mg of phosphinate **1o** (0.178 mmol, 6% over two steps) as a brown oil: ^1^H NMR (400 MHz, CDCl_3_) δ 8.61–8.52 (m, 1H), 7.73–7.61 (m, 3H), 7.59–7.51 (m, 2H), 7.47–7.41 (m, 1H), 7.19 (dddd, *J* = 7.4, 5.0, 2.3, 1.1 Hz, 1H), 4.33–4.18 (m, 2H), 3.63 (d, *J* = 20.5 Hz, 2H), 1.36 (t, *J* = 7.0 Hz, 3H); ^13^C­{^1^H} NMR (101 MHz, CDCl_3_) δ 151.4 (d, *J* = 8.4 Hz), 149.7 (d, *J* = 2.6 Hz), 136.6 (d, *J* = 2.9 Hz), 135.2 (d, *J* = 2.2 Hz), 132.5 (q, *J* = 31.3 Hz), 131.7, 130.5, 126.1 (q, *J* = 5.0 Hz), 124.8 (d, *J* = 5.0 Hz), 123.0 (q, *J* = 273.6 Hz) 122.2 (d, *J* = 3.3 Hz), 117.9–117.7 (m), 96.4 (d, *J* = 36.0 Hz), 86.2 (d, *J* = 199.1 Hz), 62.9 (d, *J* = 7.5 Hz), 41.6 (d, *J* = 114.2 Hz), 16.2 (d, *J* = 6.8 Hz); ^31^P­{^1^H} NMR (162 MHz, CDCl_3_) δ 16.22; ^19^F­{^1^H} NMR (376 MHz, CDCl_3_) δ −61.90; *R_f_* = 0.51 (92:8 CHCl_3_/MeOH); EI MS 353 (8%, M^+^), 309 (68%), 290 (23%), 261 (100%), 240 (65%), 222 (43%), 185 (40%), 157 (15%), 93 (71%), 65 (26%); HRMS (ESI/QTOF) *m*/*z* [M + H]^+^ calcd for [C_17_H_16_F_3_NO_2_P]^+^ 354.0865, found 354.0869 (100%).

#### 2-Ethoxy-4-phenylpyrido­[1,2-*a*]­[1,4]­azaphosphinine 2-Oxide (**2a**)

GP3_A_ was followed with **1a** (2.00 g, 7.01 mmol), CF_3_COOAg (31.0 mg, 0.140 mmol), and 35 mL of DCM. The resulting product was purified by column chromatography (CHCl_3_/MeOH, gradient from 100:0 to 96:4), yielding 1.74 g of 1,4-azaphosphinine **2a** (6.10 mmol, 87%) as a pale-yellow solid: ^1^H NMR (400 MHz, CDCl_3_) δ 7.55–7.41 (m, 3H), 7.41–7.29 (m, 2H), 7.01 (d, *J* = 7.6 Hz, 1H), 6.73 (d, *J* = 9.2 Hz, 1H), 6.64–6.43 (m, 1H), 5.82 (ddd, *J* = 7.8, 6.1, 1.6 Hz, 1H), 5.77–5.68 (m, 1H), 5.21 (d, *J* = 4.2 Hz, 1H), 3.98 (p, *J* = 7.4 Hz, 2H), 1.31 (t, *J* = 7.0 Hz, 3H); ^13^C­{^1^H} NMR (101 MHz, CDCl_3_) δ 151.2 (d, *J* = 2.0 Hz), 148.8 (d, *J* = 4.0 Hz), 136.8 (d, *J* = 12.2 Hz), 131.2, 129.7, 129.4 (2C), 128.7 (2C), 127.0 (d, *J* = 2.5 Hz), 126.8, 107.4, 105.9 (d, *J* = 124.3 Hz), 86.9 (d, *J* = 145.9 Hz), 61.7 (d, *J* = 6.1 Hz), 17.0 (d, *J* = 6.4 Hz); ^31^P­{^1^H} NMR (162 MHz, CDCl_3_) δ 17.57; *R_f_* = 0.41 (93:7 CHCl_3_/MeOH); EI MS 285 (<3%, M^+^), 193 (100%); HRMS (ESI/QTOF) *m*/*z* [M + H]^+^ calcd for [C_16_H_17_NO_2_P]^+^ 286.0991, found 286.0990 (100%); mp 67.2–73.6 °C (Et_2_O).

#### 2-Ethoxy-4-(4-methoxyphenyl)­pyrido­[1,2-*a*]­[1,4]­azaphosphinine 2-Oxide (**2b**)

GP3_A_ was followed with **1b** (82 mg, 0.26 mmol), CF_3_COOAg (3.0 mg, 15 μmol), and 2 mL of DCM. The resulting product was purified by column chromatography (CHCl_3_/MeOH, gradient from 100:0 to 96:4), yielding 74 mg of 1,4-azaphosphinine **2b** (0.23 mmol, 90%) as a yellow amorphous solid: ^1^H NMR (400 MHz, CDCl_3_) δ 7.28 (d, *J* = 8.6 Hz, 2H), 7.10 (dq, *J* = 7.6, 1.0 Hz, 1H), 7.02–6.95 (m, 2H), 6.77–6.70 (m, 1H), 6.64–6.56 (m, 1H), 5.83 (ddd, *J* = 7.7, 6.2, 1.6 Hz, 1H), 5.73 (dd, *J* = 4.2, 1.5 Hz, 1H), 5.21 (dd, *J* = 4.3, 1.8 Hz, 1H), 4.04–3.94 (m, 2H), 3.86 (s, 3H), 1.32 (t, *J* = 7.1 Hz, 3H); ^13^C­{^1^H} NMR (101 MHz, CDCl_3_) δ 160.5, 151.0 (d, *J* = 2.2 Hz), 148.8 (d, *J* = 4.0 Hz), 131.1, 130.0, 128.9 (d, *J* = 12.4 Hz), 126.8 (d, *J* = 1.3 Hz), 126.7 (d, *J* = 14.6 Hz), 114.6, 107.2, 105.6 (d, *J* = 124.1 Hz), 86.7 (d, *J* = 145.8 Hz), 61.6 (d, *J* = 6.0 Hz), 55.4, 16.9 (d, *J* = 6.3 Hz); ^31^P­{^1^H} NMR (162 MHz, CDCl_3_) δ 17.98; *R_f_* = 0.50 (93:7 CHCl_3_/MeOH); EI MS 315 (<1%, M^+^), 223 (100%), 208 (80%); HRMS (ESI/QTOF) *m*/*z* [M + H]^+^ calcd for [C_17_H_18_NO_3_P]^+^ 316.1097, found 316.1098 (100%).

#### 2-Ethoxy-4-(2-methoxyphenyl)­pyrido­[1,2-*a*]­[1,4]­azaphosphinine 2-Oxide (**2c**)

GP3_A_ was followed with **1c** (200 mg, 0.634 mmol), CF_3_COOAg (2.8 mg, 0.013 mmol), and 2.3 mL of DCM. The resulting product was purified by column chromatography (CHCl_3_/MeOH, gradient from 100:0 to 96:4), yielding 180 mg of a 60:40 diastereomeric mixture of 1,4-azaphosphinines **2c** (0.571 mmol, 90%) as a yellow amorphous solid: ^1^H NMR (400 MHz, CDCl_3_) δ 7.44–7.34 (m, 1H), 7.20 (ddd, *J* = 15.5, 7.5, 1.7 Hz, 1H), 6.98 (tdd, *J* = 7.5, 3.0, 1.0 Hz, 1H), 6.93–6.86 (m, 1H), 6.83 (ddd, *J* = 7.7, 3.0, 1.1 Hz, 1H), 6.65 (ddd, *J* = 9.6, 4.8, 1.4 Hz, 1H), 6.54 (ddd, *J* = 8.8, 5.9, 3.5 Hz, 1H), 5.75 (ddd, *J* = 7.7, 6.3, 1.5 Hz, 1H), 5.62 (ddd, *J* = 6.1, 4.2, 1.8 Hz, 1H), 5.08 (dd, *J* = 4.5, 2.0 Hz, 1H), 3.88–3.73 (m, 2H), 3.66 (s, 3H, OCH_3_ of the major diastereomer), 3.63 (s, 3H, OCH_3_ of the minor diastereomer), 1.24 (t, *J* = 7.1 Hz, 3H, CH_2_–CH_3_ of the minor diastereomer), 1.20 (t, *J* = 7.0 Hz, 3H, CH_2_–CH_3_ of the major diastereomer) (in the ^1^H NMR spectrum, the signals of both diastereomers overlap significantly, except for those corresponding to the CH_3_ groups, which appear as distinct resonances); ^13^C­{^1^H} NMR (101 MHz, CDCl_3_) δ 156.0, 155.9, 149.1 (d, *J* = 2.3 Hz), 148.9 (d, *J* = 2.4 Hz), 148.6 (d, *J* = 4.2 Hz), 148.5 (d, *J* = 4.1 Hz), 131.5, 131.4, 130.8, 130.7, 130.5, 130.3, 126.9 (d, *J* = 3.0 Hz), 126.5, 126.5, 126.4, 126.4, 125.3 (d, *J* = 12.2 Hz), 121.4, 121.1, 110.9, 110.8, 107.2, 107.1, 105.4 (d, *J* = 122.5 Hz), 105.4 (d, *J* = 123.2 Hz), 85.5 (d, *J* = 145.9 Hz), 85.4 (d, *J* = 144.9 Hz), 61.7 (d, *J* = 5.9 Hz), 61.2 (d, *J* = 6.1 Hz), 55.5, 55.4, 16.8 (d, *J* = 6.4 Hz), 16.5 (d, *J* = 6.5 Hz); ^31^P­{^1^H} NMR (162 MHz, CDCl_3_) δ 18.62, 18.48; *R_f_* = 0.50 (92:8 CHCl_3_/MeOH); EI MS 315 (4%, M^+^), 286 (45%), 222 (41%), 156 (30%), 131 (100%), 93 (66%), 65 (21%); HRMS (ESI/QTOF) *m*/*z* [M + H]^+^ calcd for [C_17_H_19_NO_3_P]^+^ 316.1097, found 316.1099 (100%).

#### 4-(3,4-Dimethoxyphenyl)-2-ethoxypyrido­[1,2-*a*]­[1,4]­azaphosphinine 2-Oxide (**2d**)

GP3_A_ was followed with **1d** (190 mg, 0.550 mmol), CF_3_COOAg (2.43 mg, 0.011 mmol), and 11 mL of DCM. The resulting product was purified by column chromatography (CHCl_3_/MeOH, gradient from 100:0 to 96:4), yielding 183 mg of 1,4-azaphosphinine **2d** (0.530 mmol, 96%) as an orange solid: ^1^H NMR (400 MHz, CDCl_3_) δ 7.09 (d, *J* = 7.6 Hz, 1H), 7.00–6.89 (m, 2H), 6.83 (s, 1H), 6.73 (d, *J* = 8.1 Hz, 1H), 6.64–6.56 (m, 1H), 5.83 (ddd, *J* = 7.7, 6.2, 1.6 Hz, 1H), 5.77 (dd, *J* = 4.2, 1.5 Hz, 1H), 5.22 (dd, *J* = 4.3, 1.8 Hz, 1H), 4.06–3.95 (m, 2H), 3.93 (s, 3H), 3.87 (s, 3H), 1.33 (t, *J* = 7.1 Hz, 3H); ^13^C­{^1^H} NMR (101 MHz, CDCl_3_) δ 150.9 (d, *J* = 2.1 Hz), 149.9, 149.3, 148.6 (d, *J* = 4.1 Hz), 131.0, 128.9 (d, *J* = 12.5 Hz), 126.8 (d, *J* = 3.1 Hz), 126.6, 126.5, 121.2, 111.4, 107.2, 105.4 (d, *J* = 123.8 Hz), 86.6 (d, *J* = 145.6 Hz), 61.5 (d, *J* = 6.0 Hz), 56.0, 55.9, 16.8 (d, *J* = 6.4 Hz); ^31^P­{^1^H} NMR (162 MHz, CDCl_3_) δ 17.87; *R_f_* = 0.43 (93:7 CHCl_3_/MeOH); EI MS 345 (5%, M^+^), 253 (100%), 238 (41%); HRMS (ESI/QTOF) *m*/*z* [M + H]^+^ calcd for [C_18_H_21_NO_4_P]^+^ 346.1203, found 346.1213 (100%); mp 156.0–158.0 °C (EtOAc).

#### 4-(3,5-Dimethoxyphenyl)-2-ethoxypyrido­[1,2-*a*]­[1,4]­azaphosphinine 2-Oxide (**2e**)

GP3_A_ was followed with **1e** (800 mg, 2.32 mmol), CF_3_COOAg (10 mg, 0.046 mmol), and 15 mL of DCM. The resulting product was purified by column chromatography (CHCl_3_/MeOH, gradient from 100:0 to 96:4), yielding 760 mg of 1,4-azaphosphinine **2e** (2.20 mmol, 95%) as a yellow-orange amorphous solid: ^1^H NMR (400 MHz, CDCl_3_) δ 7.06 (dd, *J* = 7.6, 1.1 Hz, 1H), 6.72 (dd, *J* = 9.6, 1.4 Hz, 1H), 6.65–6.56 (m, 1H), 6.56–6.37 (m, 3H), 5.83 (ddd, *J* = 7.7, 6.2, 1.6 Hz, 1H), 5.77 (dd, *J* = 4.2, 1.6 Hz, 1H), 5.21 (dd, *J* = 4.4, 1.9 Hz, 1H), 4.09–3.90 (m, 2H), 3.79 (s, 6H), 1.32 (t, *J* = 7.0 Hz, 3H); ^13^C­{^1^H} NMR (101 MHz, CDCl_3_) δ 161.0, 150.7 (d, *J* = 1.9 Hz), 148.4 (d, *J* = 4.0 Hz), 138.0 (d, *J* = 12.2 Hz), 130.8, 126.7 (d, *J* = 3.0 Hz), 126.3 (d, *J* = 16.1 Hz), 107.2, 106.2 (2C), 104.8 (d, *J* = 123.7 Hz), 101.2, 86.3 (d, *J* = 145.7 Hz), 61.4 (d, *J* = 6.1 Hz), 55.3 (2C), 16.6 (d, *J* = 6.4 Hz) (the signals corresponding to the two equivalent quaternary carbons could not be assigned with certainty); ^31^P­{^1^H} NMR (162 MHz, CDCl_3_) δ 17.83; *R_f_* = 0.43 (93:7 CHCl_3_/MeOH); EI MS 345 (<5%, M^+^), 253 (100%), 238 (41%); HRMS (ESI/QTOF) *m*/*z* [M + H]^+^ calcd for [C_18_H_21_NO_4_P]^+^ 346.1203, found 346.1205 (100%).

#### 2-Ethoxy-4-(*p*-tolyl)­pyrido­[1,2-*a*]­[1,4]­azaphosphinine 2-Oxide (**2f**)

GP3_A_ was followed with **1f** (135 mg, 0.451 mmol), CF_3_COOAg (1.9 mg, 9.0 μmol), and 3 mL of DCM. The resulting product was purified by column chromatography (CHCl_3_/MeOH, gradient from 100:0 to 96:4), yielding 130 mg of 1,4-azaphosphinine **2f** (0.434 mmol, 96%) as a yellow solid: ^1^H NMR (400 MHz, CDCl_3_) δ 7.31–7.20 (m, 4H), 7.06 (dd, *J* = 7.6, 1.1 Hz, 1H), 6.73 (d, *J* = 10.0 Hz, 1H), 6.63–6.54 (m, 1H), 5.81 (ddd, *J* = 7.7, 6.2, 1.6 Hz, 1H), 5.73 (dd, *J* = 4.2, 1.5 Hz, 1H), 5.21 (dd, *J* = 4.4, 1.9 Hz, 1H), 4.08–3.85 (m, 2H), 2.41 (s, 3H), 1.32 (t, *J* = 7.0 Hz, 3H); ^13^C­{^1^H} NMR (101 MHz, CDCl_3_) δ 151.3 (d, *J* = 2.0 Hz), 148.9 (d, *J* = 4.1 Hz), 139.9, 133.9 (d, *J* = 12.2 Hz), 131.2, 130.0 (2C), 128.6 (2C), 126.9, 126.9, 126.7, 107.3, 105.7 (d, *J* = 124.1 Hz), 86.8 (d, *J* = 145.8 Hz), 61.7 (d, *J* = 6.1 Hz), 21.4, 17.0 (d, *J* = 6.3 Hz); ^31^P­{^1^H} NMR (162 MHz, CDCl_3_) δ 17.54; *R_f_* = 0.69 (93:7 CHCl_3_/MeOH); EI MS 299 (<1%, M^+^), 207 (100%); HRMS (ESI/QTOF) *m*/*z* [M + H]^+^ calcd for [C_17_H_19_NO_2_P]^+^ 300.1148, found 300.1148 (100%); mp 158.6–160.9 °C (EtOAc).

#### 4-(4-(*tert*-Butyl)­phenyl)-2-ethoxypyrido­[1,2-*a*]­[1,4]­azaphosphinine 2-Oxide (**2g**)

GP3_A_ was followed with **1g** (60.0 mg, 0.176 mmol), CF_3_COOAg (1.94 mg, 8.79 μmol), and 4 mL of DCM. The resulting product was purified by column chromatography (CHCl_3_/MeOH, gradient from 100:0 to 96:4), yielding 53.0 mg of 1,4-azaphosphinine **2g** (0.155 mmol, 88%) as a yellow amorphous solid: ^1^H NMR (400 MHz, CDCl_3_) δ 7.47 (d, *J* = 8.7 Hz, 2H), 7.31–7.21 (m, 2H), 7.09 (dt, *J* = 7.6, 1.0 Hz, 1H), 6.73 (d, *J* = 9.2 Hz, 1H), 6.64–6.50 (m, 1H), 5.83 (ddd, *J* = 7.7, 6.2, 1.6 Hz, 1H), 5.74 (dd, *J* = 4.2, 1.6 Hz, 1H), 5.20 (dd, *J* = 4.4, 1.9 Hz, 1H), 4.05–3.89 (m, 2H), 1.35 (s, 9H), 1.31 (t, *J* = 7.0 Hz, 3H); ^13^C­{^1^H} NMR (101 MHz, CDCl_3_) δ 153.0, 151.4 (d, *J* = 1.9 Hz), 148.9 (d, *J* = 4.1 Hz), 133.7 (d, *J* = 12.2 Hz), 131.3, 128.3 (2C), 127.0 (d, *J* = 3.2 Hz), 126.8, 126.7, 126.2 (2C), 107.3, 105.6 (d, *J* = 124.1 Hz), 86.6 (d, *J* = 145.8 Hz), 61.7 (d, *J* = 6.1 Hz), 34.9, 31.3, 16.9 (d, *J* = 6.5 Hz); ^31^P­{^1^H} NMR (162 MHz, CDCl_3_) δ 17.88; *R_f_* = 0.52 (93:7 CHCl_3_/MeOH); EI MS 341 (1%, M^+^), 249 (100%), 234 (77%), 219 (23%), 103 (17%); HRMS (ESI/QTOF) *m*/*z* [M + H]^+^ calcd for [C_20_H_25_NO_2_P]^+^ 342.1617, found 342.1615 (100%).

#### 4-(4-Bromophenyl)-2-ethoxypyrido­[1,2-*a*]­[1,4]­azaphosphinine 2-Oxide (**2h**)

GP3_A_ was followed with **1h** (200 mg, 0.549 mmol), CF_3_COOAg (2.42 mg, 10.9 μmol), and 3.6 mL of DCM. The resulting product was purified by column chromatography (CHCl_3_/MeOH, gradient from 100:0 to 96:4), yielding 168 mg of 1,4-azaphosphinine **2h** (0.461 mmol, 84%) as a pale-yellow amorphous solid: ^1^H NMR (400 MHz, CDCl_3_) δ 7.62 (d, *J* = 8.6 Hz, 2H), 7.24 (d, *J* = 8.1 Hz, 2H), 6.97 (dd, *J* = 7.6, 1.1 Hz, 1H), 6.74 (d, *J* = 9.2 Hz, 1H), 6.61 (ddt, *J* = 10.0, 6.7, 1.9 Hz, 1H), 5.85 (ddd, *J* = 7.7, 6.2, 1.6 Hz, 1H), 5.73 (dd, *J* = 4.2, 1.8 Hz, 1H), 5.23 (dd, *J* = 4.4, 1.9 Hz, 1H), 4.01 (dq, *J* = 9.1, 7.0 Hz, 2H), 1.32 (t, *J* = 7.0 Hz, 3H); ^13^C­{^1^H} NMR δ 149.9 (d, *J* = 2.2 Hz), 148.8 (d, *J* = 4.0 Hz), 135.6 (d, *J* = 12.4 Hz), 132.7, 130.9, 130.3, 127.0 (d, *J* = 13.3 Hz), 127.0, 124.3, 107.8, 106.3 (d, *J* = 124.0 Hz), 87.3 (d, *J* = 146.5 Hz), 61.9 (d, *J* = 6.2 Hz), 17.0 (d, *J* = 6.3 Hz); ^31^P­{^1^H} NMR (162 MHz, CDCl_3_) δ 17.13; *R_f_* = 0.57 (92:8 CHCl_3_/MeOH); EI MS 363 (<2%, M^+^), 273 (97%) 271 (100%), 191 (42%); HRMS (ESI/QTOF) *m*/*z* [M + H]^+^ calcd for [C_16_H_16_^79^BrNO_2_P]^+^ 364.0097, found 364.0099 (100%).

#### 4-(4-Chlorophenyl)-2-ethoxypyrido­[1,2-*a*]­[1,4]­azaphosphinine 2-Oxide (**2i**)

GP3_A_ was followed with **1i** (600 mg, 1.88 mmol), CF_3_COOAg (8.3 mg, 0.038 mmol), and 12.5 mL of DCM. The resulting product was purified by column chromatography (CHCl_3_/MeOH, gradient from 100:0 to 96:4), yielding 553 mg of 1,4-azaphosphinine **2i** (1.73 mmol, 92%) as a yellow solid: ^1^H NMR (400 MHz, CDCl_3_) δ 7.46 (d, *J* = 8.8 Hz, 2H), 7.30 (d, *J* = 7.8 Hz, 2H), 6.97 (dd, *J* = 7.6, 1.0 Hz, 1H), 6.77–6.69 (m, 1H), 6.65–6.54 (m, 1H), 5.84 (ddd, *J* = 7.7, 6.2, 1.6 Hz, 1H), 5.72 (dd, *J* = 4.2, 1.7 Hz, 1H), 5.27–5.18 (m, 1H), 4.00 (dq, *J* = 9.2, 7.1 Hz, 2H), 1.32 (t, *J* = 7.0 Hz, 3H); ^13^C­{^1^H} NMR δ 149.7 (d, *J* = 2.2 Hz), 148.6 (d, *J* = 4.0 Hz), 135.9, 135.0 (d, *J* = 12.4 Hz), 130.7, 129.9 (2C), 129.6 (2C), 126.9 (d, *J* = 1.7 Hz), 126.8 (d, *J* = 11.5 Hz), 107.6, 106.1 (d, *J* = 123.6 Hz), 87.1 (d, *J* = 146.1 Hz), 61.6 (d, *J* = 6.0 Hz), 16.9 (d, *J* = 6.3 Hz); ^31^P­{^1^H} NMR (162 MHz, CDCl_3_) δ 17.07; *R_f_* = 0.65 (93:7 CHCl_3_/MeOH); EI MS 319 (<2%, M^+^), 229 (35%), 227 (100%), 191 (18%); HRMS (ESI/QTOF) *m*/*z* [M + H]^+^ calcd for [C_16_H_16_ClNO_2_P]^+^ 320.0602, found 320.0601 (100%); mp 157.1–161.3 °C (EtOAc).

#### 2-Ethoxy-4-(4-fluorophenyl)­pyrido­[1,2-*a*]­[1,4]­azaphosphinine 2-Oxide (**2j**)

GP3_A_ was followed with **1j** (0.105 g, 0.35 mmol), CF_3_COOAg (4 mg, 0.02 mmol), and 3 mL of DCM. The resulting product was purified by column chromatography (CHCl_3_/MeOH, gradient from 100:0 to 96:4), yielding 0.86 g of 1,4-azaphosphinine **2j** (0.28 mmol, 82%) as an orange amorphous solid: ^1^H NMR (400 MHz, CDCl_3_) δ 7.40–7.32 (m, 2H), 7.20–7.15 (m, 2H), 6.99 (dd, *J* = 7.7, 1.1 Hz, 1H), 6.74 (d, *J* = 9.2 Hz, 1H), 6.65–6.56 (m, 1H), 5.85 (ddd, *J* = 7.7, 6.2, 1.6 Hz, 1H), 5.74 (dd, *J* = 4.2, 1.7 Hz, 1H), 5.24 (dd, *J* = 4.5, 1.9 Hz, 1H), 4.02 (dq, *J* = 9.1, 7.1 Hz, 2H), 1.33 (t, *J* = 7.0 Hz, 3H); ^13^C­{^1^H} NMR δ 163.2 (d, *J* = 250.8 Hz), 149.8 (d, *J* = 2.2 Hz), 148.6 (d, *J* = 4.0 Hz), 132.7 (dd, *J* = 12.5, 3.7 Hz), 130.8, 126.9, 126.78 (d, *J* = 1 Hz), 126.76, 116.5 (d, *J* = 21.7 Hz), 107.5, 106.3 (d, *J* = 124.1 Hz), 87.2 (d, *J* = 146.2 Hz), 61.7 (d, *J* = 6.1 Hz), 16.9 (d, *J* = 6.3 Hz); ^19^F NMR (376 MHz, CDCl_3_) δ −110.34; ^31^P­{^1^H} NMR (162 MHz, CDCl_3_) δ 17.23; *R_f_* = 0.37 (93:7 CHCl_3_/MeOH); EI MS 303 (<1%, M^+^), 211 (100%); HRMS (ESI/QTOF) *m*/*z* [M + H]^+^ calcd for [C_16_H_16_FNO_2_P]^+^ 304.0897, found 304.0892 (100%).

#### 4-(3,4-Difluorophenyl)-2-ethoxypyrido­[1,2-*a*]­[1,4]­azaphosphinine 2-Oxide (**2k**)

GP3_A_ was followed with **1k** (460 mg, 1.43 mmol), CF_3_COOAg (6.3 mg, 0.029 mmol), and 9.5 mL of DCM. The resulting product was purified by column chromatography (CHCl_3_/MeOH, gradient from 100:0 to 96:4), yielding 382 mg of 1,4-azaphosphinine **2k** (1.19 mmol, 83%) as a yellow amorphous solid: ^1^H NMR (400 MHz, CDCl_3_) δ 7.29 (q, *J* = 8.7 Hz, 1H), 7.20 (t, *J* = 9.4 Hz, 1H), 7.12 (d, *J* = 8.6 Hz, 1H), 6.94 (dd, *J* = 7.6, 1.1 Hz, 1H), 6.74 (d, *J* = 9.2 Hz, 1H), 6.66–6.52 (m, 1H), 5.87 (ddd, *J* = 7.7, 6.2, 1.6 Hz, 1H), 5.73 (dd, *J* = 4.1, 1.9 Hz, 1H), 5.24 (dd, *J* = 4.4, 1.9 Hz, 1H), 4.01 (dq, *J* = 9.1, 7.0 Hz, 2H), 1.32 (t, *J* = 7.1 Hz, 3H); ^13^C­{^1^H} NMR (101 MHz, CHCl_3_) δ 150.9 (dd, *J* = 240.6, 14.5 Hz), 150.3 (dd, *J* = 239.2, 14.6 Hz), 148.6–148.3 (m), 133.1 (dt, *J* = 12.5, 5.1 Hz), 130.3, 126.8, 126.8, 126.6, 125.1, 118.4 (d, *J* = 17.5 Hz), 117.9 (d, *J* = 17.7 Hz), 107.8, 106.3 (d, *J* = 123.6 Hz), 87.0 (d, *J* = 146.3 Hz), 61.6 (d, *J* = 6.1 Hz), 16.6 (d, *J* = 6.4 Hz); ^31^P­{^1^H} NMR (162 MHz, CDCl_3_) δ 16.74; ^19^F NMR (376 MHz, CDCl_3_) δ −134.85, −134.90; *R_f_* = 0.49 (92:8 CHCl_3_/MeOH); EI MS 321 (<3%, M^+^), 229 (100%); HRMS (ESI/QTOF) *m*/*z* [M + H]^+^ calcd for [C_16_H_15_F_2_NO_2_P]^+^ 322.0803, found 322.0804 (100%).

#### 4-(3,5-Difluorophenyl)-2-ethoxypyrido­[1,2-*a*]­[1,4]­azaphosphinine 2-Oxide (**2l**)

GP3_A_ was followed with **1l** (80 mg, 0.25 mmol), CF_3_COOAg (2.7 mg, 12 μmol), and 2.5 mL of DCM. The resulting product was purified by column chromatography (CHCl_3_/MeOH, gradient from 100:0 to 96:4), yielding 60 mg of 1,4-azaphosphinine **2l** (0.19 mmol, 76%) as a yellow-orange amorphous solid: ^1^H NMR (400 MHz, CDCl_3_) δ 7.00–6.90 (m, 4H), 6.75 (ddd, *J* = 9.3, 1.5, 0.8 Hz, 1H), 6.62 (dddd, *J* = 10.8, 6.3, 2.4, 0.9 Hz, 1H), 5.90 (ddd, *J* = 7.7, 6.2, 1.6 Hz, 1H), 5.76 (dd, *J* = 4.1, 2.1 Hz, 1H), 5.32–5.10 (m, 1H), 4.03 (dq, *J* = 9.1, 7.0 Hz, 2H), 1.33 (t, *J* = 7.0 Hz, 3H); ^13^C­{^1^H} NMR δ 163.3 (dd, *J* = 252.0, 12.1 Hz), 148.6 (d, *J* = 4.0 Hz), 148.3 (q, *J* = 2.4 Hz), 139.3 (dt, *J* = 12.6, 9.6 Hz), 130.4, 127.0 (d, *J* = 9.3 Hz), 126.9 (d, *J* = 3.7 Hz), 112.1 (d, *J* = 22.8 Hz, 2C), 108.1, 106.4 (d, *J* = 123.9 Hz), 105.5, 87.4 (d, *J* = 146.9 Hz), 61.8 (d, *J* = 6.2 Hz), 16.9 (d, *J* = 6.4 Hz); ^31^P­{^1^H} NMR (162 MHz, CDCl_3_) δ 16.50; ^19^F NMR (376 MHz, CDCl_3_) δ −106.37, −106.91; *R_f_* = 0.58 (92:8 CHCl_3_/MeOH); EI MS 321 (2%, M^+^), 229 (100%); HRMS (ESI/QTOF) *m*/*z* [M + H]^+^ calcd for [C_16_H_15_F_2_NO_2_P]^+^ 322.0803, found 322.0803 (100%).

#### 4-(2-Ethoxy-2-oxidopyrido­[1,2-*a*]­[1,4]­azaphosphinin-4-yl)­benzonitrile (**2m**)

GP3_A_ was followed with **1m** (150.0 mg, 0.483 mmol), CF_3_COOAg (4.3 mg, 19 μmol), and 3.2 mL of DCM. The resulting product was purified by column chromatography (CHCl_3_/MeOH, gradient from 100:0 to 96:4), yielding 115.0 mg of 1,4-azaphosphinine **2m** (0.371 mmol, 77%) as a pale-yellow solid: ^1^H NMR (400 MHz, CDCl_3_) δ 7.79 (d, *J* = 8.5 Hz, 2H), 7.51 (d, *J* = 7.7 Hz, 2H), 6.84 (dd, *J* = 7.6, 1.1 Hz, 1H), 6.76 (dd, *J* = 9.1, 1.3 Hz, 1H), 6.62 (ddt, *J* = 10.2, 6.8, 1.7 Hz, 1H), 5.87 (ddd, *J* = 7.7, 6.2, 1.6 Hz, 1H), 5.73 (dd, *J* = 4.2, 2.1 Hz, 1H), 5.27 (dd, *J* = 4.3, 1.9 Hz, 1H), 4.03 (dq, *J* = 9.1, 7.0 Hz, 2H), 1.32 (t, *J* = 7.1 Hz, 3H); ^13^C­{^1^H} NMR (101 MHz, CDCl_3_) δ 148.8 (d, *J* = 2.0 Hz), 148.6 (d, *J* = 3.8 Hz), 141.0 (d, *J* = 12.4 Hz), 133.3 (2C), 130.5, 129.6 (2C), 127.2 (d, *J* = 16.1 Hz), 127.0 (d, *J* = 3.1 Hz), 117.9, 114.0, 108.2, 107.0 (d, *J* = 123.9 Hz), 87.8 (d, *J* = 146.9 Hz), 62.0 (d, *J* = 6.0 Hz), 17.0 (d, *J* = 6.3 Hz); ^31^P­{^1^H} NMR (162 MHz, CDCl_3_) δ 16.33; *R_f_* = 0.72 (93:7 CHCl_3_/MeOH); EI MS 310 (<3%, M^+^), 218 (100%); HRMS (ESI/QTOF) *m*/*z* [M + H]^+^ calcd for [C_17_H_16_N_2_O_2_P]^+^ 311.0944, found 311.0940 (100%); mp 149.5–152.0 °C (EtOAc).

#### 2-Ethoxy-4-(4-(trifluoromethyl)­phenyl)­pyrido­[1,2-*a*]­[1,4]­azaphosphinine 2-Oxide (**2n**)

GP3_A_ was followed with **1n** (40.0 mg, 0.113 mmol), CF_3_COOAg (1.2 mg, 5.6 μmol), and 2 mL of DCM. The resulting product was purified by column chromatography (CHCl_3_/MeOH, gradient from 100:0 to 96:4), yielding 32.0 mg of 1,4-azaphosphinine **2n** (0.091 mmol, 80%) as a pale-yellow solid: ^1^H NMR (400 MHz, CDCl_3_) δ 7.76 (d, *J* = 8.0 Hz, 2H), 7.52 (d, *J* = 7.8 Hz, 2H), 6.90 (dd, *J* = 7.6, 1.0 Hz, 1H), 6.81–6.71 (m, 1H), 6.68–6.58 (m, 1H), 5.87 (ddd, *J* = 7.7, 6.2, 1.5 Hz, 1H), 5.76 (dd, *J* = 4.1, 2.0 Hz, 1H), 5.27 (dd, *J* = 4.4, 1.9 Hz, 1H), 4.03 (dq, *J* = 9.1, 7.0 Hz, 2H), 1.33 (t, *J* = 7.1 Hz, 3H); ^13^C­{^1^H} NMR (101 MHz, CDCl_3_) δ 149.4 (d, *J* = 2.1 Hz), 148.7 (d, *J* = 4.0 Hz), 140.2 (d, *J* = 12.6 Hz), 132.0 (q, *J* = 33.0 Hz), 130.7, 129.3 (2C), 127.2, 127.0, 126.5 (2C), 123.7 (q, *J* = 272.7 Hz), 108.0, 106.6 (d, *J* = 124.0 Hz), 87.5 (d, *J* = 146.7 Hz), 61.9 (d, *J* = 6.1 Hz), 17.0 (d, *J* = 6.3 Hz); ^31^P­{^1^H} NMR (162 MHz, CDCl_3_) δ 16.82; ^19^F­{^1^H} NMR (376 MHz, CDCl_3_) δ −62.92; *R_f_* = 0.55 (93:7 CHCl_3_/MeOH); EI MS 353 (<2%, M^+^), 261 (100%), 191 (7%); HRMS (ESI/QTOF) *m*/*z* [M + H]^+^ calcd for [C_17_H_16_F_3_NO_2_P]^+^ 354.0865, found 354.0868 (100%); mp 192.8–195.0 °C (EtOAc).

#### 2-Ethoxy-4-(2-(trifluoromethyl)­phenyl)­pyrido­[1,2-*a*]­[1,4]­azaphosphinine 2-Oxide (**2o**)

GP3_A_ was followed with **1o** (50.0 mg, 0.142 mmol), CF_3_COOAg (1.5 mg, 7.1 μmol), and 2.8 mL of DCM. The resulting product was purified by column chromatography (CHCl_3_/MeOH, gradient from 100:0 to 96:4), yielding 36.0 mg of a 92:8 diastereomeric mixture of 1,4-azaphosphinines **2o** (0.102 mmol, 72%) as a yellow amorphous solid: ^1^H NMR (400 MHz, CDCl_3_) δ 7.81 (dd, *J* = 7.8, 1.5 Hz, 1H), 7.68 (td, *J* = 7.6, 1.5 Hz, 1H), 7.63 (td, *J* = 8.0, 1.5 Hz, 1H), 7.39 (d, *J* = 8.0 Hz, 1H), 6.81–6.66 (m, 1H), 6.66–6.52 (m, 2H), 5.82 (ddd, *J* = 7.7, 6.2, 1.6 Hz, 1H), 5.69 (dd, *J* = 4.2, 2.3 Hz, 1H), 5.19 (dd, *J* = 4.3, 2.1 Hz, 1H), 3.88–3.71 (m, 2H), 1.26 (t, *J* = 7.1 Hz, 3H); ^13^C­{^1^H} NMR (101 MHz, CDCl_3_) δ 148.8 (d, *J* = 3.8 Hz), 147.4 (d, *J* = 2.1 Hz), 134.2 (dd, *J* = 12.3, 2.1 Hz), 133.1, 131.1, 130.6, 130.2, 128.5 (q, *J* = 30.8 Hz), 127.2–127.1 (m), 127.0, 126.9–126.8 (m), 123.5 (q, *J* = 274.0 Hz), 107.7, 106.7 (d, *J* = 120.3 Hz), 86.4 (d, *J* = 145.0 Hz), 62.0 (d, *J* = 6.2 Hz), 16.6 (d, *J* = 7.0 Hz) (the signals corresponding to the minor diastereomer in the ^13^C and ^1^H NMR spectra were not assigned); ^31^P­{^1^H} NMR (162 MHz, CDCl_3_) δ 16.51, 16.10; ^19^F­{^1^H} NMR (376 MHz, CDCl_3_) δ −59.65, −59.71; *R_f_* = 0.45 (92:8 CHCl_3_/MeOH); EI MS 353 (5%, M^+^), 261 (100%), 240 (10%), 222 (5%); HRMS (ESI/QTOF) *m*/*z* [M + H]^+^ calcd for [C_17_H_16_F_3_NO_2_P]^+^ 354.0865, found 354.0862 (100%).

### General Procedure for the Preparation of Alcohols **7p–u** (GP1_B_)

A Schlenk flask was charged with the appropriate 2-bromopyridine (1 equiv) and dry tetrahydrofuran (THF) to achieve a 0.2 M solution under an inert atmosphere. The reaction mixture was cooled to −78 °C, and a solution of *n*-butyllithium (1 equiv, 2.5 M in *n*-hexane) was added dropwise. After the mixture had been stirred for 15 min, the appropriate aldehyde (1 equiv) was added dropwise. The mixture was stirred for 1 h at −78 °C and then slowly warmed to room temperature. A saturated aqueous solution of ammonium chloride (NH_4_Cl) was added, and the mixture was extracted with diethyl ether (Et_2_O, 3 × 20 mL). The combined organic layers were dried over anhydrous magnesium sulfate (MgSO_4_). After solvent evaporation, the residue was purified by column chromatography.

### General Procedure for the Preparation of Bromides **8p** and **8r–u** (GP2_B_)

A Schlenk flask was charged with the appropriate alcohol **7p** or **7r–u** (1 equiv), triphenylphosphine (1.8 equiv), tetrabromomethane (1.5 equiv), and dry tetrahydrofuran (THF) to achieve a 0.15 M solution under an inert atmosphere. After the mixture had been stirred at room temperature for 1 h, the precipitate was filtered through a pad of Celite, and the solid was washed with cold diethyl ether. The combined filtrate was collected. After removal of the solvents under reduced pressure, the residue was purified by column chromatography.

### General Procedure for the Preparation of Phosphinates **1p** and **1r–u** (GP3_B_)

A round-bottom flask was charged with the appropriate bromomethyl azaaromatic **8p** or **8r–u** (1 equiv) and phosphonite **6a** (1 equiv). The resulting reaction mixture was heated at 100 °C in an oil bath for approximately 2 h. Upon consumption of the starting materials, as monitored by GC-MS, the crude mixture was purified by column chromatography to afford the desired phosphinates **1p** or **1r**–**u**.

### General Procedure for the Preparation of 1,4-Azaphosphinines **2p–u** (GP4_B_)

A Schlenk flask was charged with phosphinate **1p**–**u** (1 equiv), silver­(I) trifluoroacetate (0.02–0.05 equiv), and dry DCM (0.05–0.15 M) under an inert atmosphere. The resulting mixture was stirred overnight. Upon consumption of the starting material, as monitored by GC-MS or TLC, the crude product was purified by column chromatography to afford the desired 1,4-azaphosphinines **1p–u** as an oil, which typically solidified over time or upon treatment with EtOAc.

#### Phenyl­(pyridin-2-yl)­methanol (**7p**)

A round-bottom flask was charged with phenyl­(pyridin-2-yl)­methanone (1.00 g, 5.46 mmol), sodium borohydride (103 mg, 2.73 mmol), and 27 mL of MeOH. After the mixture had been stirred at room temperature for 1 h, the reaction was quenched with water. The mixture was concentrated under vacuum and extracted with dichloromethane (DCM, 3 × 15 mL). The combined organic layers were dried over anhydrous magnesium sulfate. After solvent evaporation, 0.988 g of alcohol **7p** (5.33 mmol, 98%) was obtained as a white solid: ^1^H NMR (400 MHz, CDCl_3_) δ 8.42 (d, *J* = 6.0 Hz, 2H), 7.36–7.28 (m, 7H), 5.77 (s, 3H). The NMR spectrum is in accordance with published literature data.^39^

#### (4-Methoxyphenyl)­(pyridin-2-yl)­methanol (**7q**)

GP1_B_ was followed with 2.00 mL of 2-bromopyridine (20.5 mmol), 103 mL of THF, 8.2 mL of a solution of *n*-BuLi (20.5 mmol, 2.5 M in *n*-hexane), and 2.49 mL of 4-methoxybenzaldehyde (20.5 mmol). Crystallization from ethanol provided 2.4 g of alcohol **7q** (11.2 mmol, 54%) as a white solid: ^1^H NMR (400 MHz, CDCl_3_) δ 8.65–8.48 (m, 1H), 7.61 (td, *J* = 7.7, 1.8 Hz, 1H), 7.28 (d, *J* = 8.7 Hz, 2H), 7.23–7.16 (m, 1H), 7.13 (dd, *J* = 7.9, 1.0 Hz, 1H), 6.87 (d, *J* = 8.7 Hz, 2H), 5.71 (d, *J* = 3.2 Hz, 1H), 5.26–5.10 (m, 1H), 3.79 (s, 3H). The NMR spectrum is in accordance with published literature data.^40^

#### (4-Trifluoromethylphenyl)­(pyridin-2-yl)­methanol (**7r**)

GP1_B_ was followed with 2.00 mL of 2-bromopyridine (20.5 mmol), 103 mL of THF, 8.2 mL of a solution of *n*-BuLi (20.5 mmol, 2.5 M in *n*-hexane), and 2.80 mL of 4-trifluoromethylbenzaldehyde (20.5 mmol). The resulting product was purified by column chromatography (90:10 PE/EtOAc), yielding 4.11 g of alcohol **7r** (16.2 mmol, 79%) as a white solid: ^1^H NMR (400 MHz, CDCl_3_) δ 8.58 (d, *J* = 5.1 Hz, 1H), 7.65 (td, *J* = 7.7, 1.8 Hz, 1H), 7.60 (d, *J* = 8.1 Hz, 2H), 7.52 (d, *J* = 8.1 Hz, 2H), 7.23 (ddd, *J* = 7.0, 4.6, 0.9 Hz, 1H), 7.14 (dd, *J* = 7.9, 0.9 Hz, 1H), 5.80 (s, 1H), 5.34 (s, 1H). The NMR spectrum is in accordance with published literature data.^41^

#### (2,5-Dimethylphenyl)­(pyridin-2-yl)­methanol (**7s**)

GP1_B_ was followed with 1.16 mL of 2-bromopyridine (11.9 mmol), 60 mL of THF, 4.8 mL of a solution of *n*-BuLi (11.9 mmol, 2.5 M in *n*-hexane), and 1.60 g of 2,5-dimethylbenzaldehyde (11.9 mmol). The resulting product was purified by column chromatography (PE/EtOAc, gradient from 100:0 to 75:25), yielding 2.1 g of alcohol **7s** (9.85 mmol, 83%) as a white solid: ^1^H NMR (400 MHz, CDCl_3_) δ 8.59 (d, *J* = 5.0 Hz, 1H), 7.60 (td, *J* = 7.7, 1.7 Hz, 1H), 7.24–7.17 (m, 1H), 7.09–6.98 (m, 4H), 5.94 (s, 1H), 5.14 (s, 1H), 2.30 (s, 3H), 2.27 (s, 3H); ^13^C­{^1^H} NMR (101 MHz, CDCl_3_) δ 161.2, 147.9, 140.5, 136.9, 135.7, 133.1, 130.7, 128.7, 128.6, 122.4, 121.3, 72.8, 21.1, 19.1; *R_f_* = 0.34 (75:25 PE/EtOAc); EI MS 213 (19%, M^+^), 194 (100%), 182 (14%), 93 (21%), 80 (25%); HRMS (ESI/QTOF) *m*/*z* [M + H]^+^ calcd for [C_14_H_16_NO]^+^ 214.1226, found 214.1218 (17%).

#### Naphthalen-1-yl­(pyridin-2-yl)­methanol (**7t**)

GP1_B_ was followed with 1.00 mL of 2-bromopyridine (10.3 mmol), 51 mL of THF, 4.1 mL of a solution of *n*-BuLi (10.3 mmol, 2.5 M in *n*-hexane), and 1.39 mL of 1-naphthaldehyde (10.3 mmol). The resulting product was purified by column chromatography (PE/EtOAc, gradient from 75:25 to 55:45), yielding 1.63 g of alcohol **7t** (6.93 mmol, 67%) as a white solid: ^1^H NMR (400 MHz, CDCl_3_) δ 8.65 (d, *J* = 4.9 Hz, 1H), 8.14–8.09 (m, 1H), 7.89–7.80 (m, 2H), 7.55 (td, *J* = 7.7, 1.8 Hz, 1H), 7.51–7.40 (m, 4H), 7.24–7.18 (m, 1H), 7.05 (dd, *J* = 7.9, 1.0 Hz, 1H), 6.42 (s, 1H), 5.40 (s, 1H). The NMR spectrum is in accordance with published literature data.^42^

#### (Mesityl)­(pyridin-2-yl)­methanol (**7u**)

GP1_B_ was followed with 1.00 mL of 2-bromopyridine (10.3 mmol), 51 mL of THF, 4.1 mL of a solution of *n*-BuLi (10.3 mmol, 2.5 M in *n*-hexane), and 1.49 mL of 2,4,6-trimethylbenzaldehyde (10.3 mmol). The resulting product was purified by column chromatography (PE/EtOAc, gradient from 88:12 to 80:20), yielding 1.79 g of alcohol **7u** (7.88 mmol, 77%) as a white solid: ^1^H NMR (400 MHz, CDCl_3_) δ 8.59 (dt, *J* = 4.9, 1.4 Hz, 1H), 7.57 (td, *J* = 7.7, 1.7 Hz, 1H), 7.19 (ddt, *J* = 7.3, 5.0, 1.0 Hz, 1H), 6.90 (dd, *J* = 7.9, 1.1 Hz, 1H), 6.83 (s, 2H), 6.19 (s, 1H), 5.38 (d, *J* = 1.8 Hz, 1H), 2.26 (s, 3H), 2.18 (s, 6H). The NMR spectrum is in accordance with published literature data.^42^

#### 2-(Bromo­(phenyl)­methyl)­pyridine (**8p**)

GP2_B_ was followed with **7p** (560 mg, 3.02 mmol), triphenylphosphine, (1.43 g, 5.44 mmol), tetrabromomethane (1.50 g, 4.54 mmol), and 21 mL of THF. The resulting product was purified by column chromatography (PE/EtOAc, gradient from 88:12 to 80:20), yielding 570 mg of bromide **8p** (2.30 mmol, 76%) as a pink amorphous solid: ^1^H NMR (400 MHz, CDCl_3_) δ 8.60 (ddd, *J* = 4.8, 1.8, 0.9 Hz, 1H), 7.68 (td, *J* = 7.7, 1.8 Hz, 1H), 7.59–7.50 (m, 3H), 7.39–7.31 (m, 2H), 7.31–7.26 (m, 1H), 7.19 (ddd, *J* = 7.5, 4.8, 1.1 Hz, 1H), 6.26 (s, 1H); ^13^C­{^1^H} NMR (101 MHz, CDCl_3_) δ 159.5, 149.3, 139.8, 137.0, 128.7 (2C), 128.6 (2C), 128.4, 122.9, 122.8, 55.1; *R_f_* = 0.44 (83:17 PE/EtOAc); EI MS 168 (100%, [M – Br]^+^); HRMS (ESI) *m*/*z* [M + H]^+^ calcd for [C_12_H_11_^79^BrN]^+^ 248.0069, found 248.0071 (43%).

#### 2-(Bromo­(4-(trifluoromethyl)­phenyl)­methyl)­pyridine (**8r**)

GP2_B_ was followed with **7r** (750 mg, 3.52 mmol), triphenylphosphine (1.66 g, 6.33 mmol), tetrabromomethane (1.96 g, 5.92 mmol), and 26 mL of THF. The resulting product was purified by column chromatography (PE/EtOAc, gradient from 100:0 to 82:18), yielding 1.01 g of bromide **8r** (3.20 mmol, 81%) as a slightly pink oil: ^1^H NMR (400 MHz, CDCl_3_) δ 8.61 (ddd, *J* = 4.9, 1.8, 0.9 Hz, 1H), 7.75–7.65 (m, 3H), 7.60 (d, *J* = 8.2 Hz, 2H), 7.54 (dt, *J* = 7.9, 1.1 Hz, 1H), 7.22 (ddd, *J* = 7.6, 4.8, 1.1 Hz, 1H), 6.25 (s, 1H); ^13^C­{^1^H} NMR (101 MHz, CDCl_3_) δ 158.8, 149.7, 143.9 (d, *J* = 1.5 Hz), 137.4, 130.5 (q, *J* = 32.6 Hz), 129.2 (2C), 125.7 (q, *J* = 3.7 Hz, 2C), 12.0 (q, *J* = 272.3 Hz), 123.2, 123.0, 53.6; ^19^F­{^1^H} NMR (376 MHz, CDCl_3_) δ −62.71; *R_f_* = 0.67 (83:17 PE/EtOAc); EI MS 236 (100%, [M – Br]^+^), 167 (51%); HRMS (ESI/QTOF) *m*/*z* [M + H]^+^ calcd for [C_13_H_9_^79^BrF_3_N]^+^ 315.9945, found 315.9945 (100%).

#### 2-(Bromo­(2,5-dimethylphenyl)­methyl)­pyridine (**8s**)

GP2_B_ was followed with **7s** (1.00 g, 3.95 mmol), triphenylphosphine (1.86 g, 7.11 mmol), tetrabromomethane (1.75 g, 5.27 mmol), and 23 mL of THF. The resulting product was purified by column chromatography (PE/EtOAc, gradient from 100:0 to 90:10), yielding 670 mg of bromide **8s** (2.42 mmol, 69%) as a pink amorphous solid: ^1^H NMR (400 MHz, CDCl_3_) δ 8.60 (ddd, *J* = 4.9, 1.9, 0.9 Hz, 1H), 7.70 (td, *J* = 7.7, 1.8 Hz, 1H), 7.55 (dt, *J* = 7.9, 1.1 Hz, 1H), 7.39–7.32 (m, 1H), 7.20 (ddd, *J* = 7.5, 4.8, 1.2 Hz, 1H), 7.08–6.96 (m, 2H), 6.47 (s, 1H), 2.35 (s, 3H), 2.30 (s, 3H); ^13^C­{^1^H} NMR (101 MHz, CDCl_3_) δ 159.1, 148.8, 137.6, 137.5, 136.1, 132.8, 130.8, 130.0, 129.4, 123.6, 122.9, 52.3, 21.2, 19.2; *R_f_* = 0.8 (75:25 PE/EtOAc); EI MS 196 (100%, [M – Br]^+^), 181 (64%); HRMS (ESI/QTOF) *m*/*z* [M – Br]^+^ calcd for [C_14_H_14_N]^+^ 196.1121, found 196.1124 (100%).

#### 2-(Bromo­(naphthalen-1-yl)­methyl)­pyridine (**8t**)

GP2_B_ was followed with **7t** (1.60 g, 6.80 mmol), triphenylphosphine (3.21 g, 12.24 mmol), tetrabromomethane (3.38 g, 10.20 mmol), and 45 mL of THF. The resulting product was purified by column chromatography (PE/EtOAc, gradient from 100:0 to 75:25), yielding 1.61 g of bromide **8t** (5.41 mmol, 80%) as a pink amorphous solid: ^1^H NMR (400 MHz, CDCl_3_) δ 8.64 (ddd, *J* = 4.9, 1.8, 0.9 Hz, 1H), 8.16 (d, *J* = 9.0 Hz, 1H), 7.88 (dd, *J* = 8.0, 1.6 Hz, 1H), 7.84 (d, *J* = 8.2 Hz, 1H), 7.77 (dd, *J* = 7.2, 1.2 Hz, 1H), 7.68 (td, *J* = 7.7, 1.8 Hz, 1H), 7.59–7.42 (m, 4H), 7.22 (ddd, *J* = 7.5, 4.8, 1.1 Hz, 1H), 7.08 (s, 1H); ^13^C­{^1^H} NMR (101 MHz, CDCl_3_) δ 159.5, 149.3, 137.2, 135.1, 134.0, 130.5, 129.5, 129.0, 127.9, 126.7, 126.1, 125.5, 123.8, 123.5, 122.9, 52.9; *R_f_* = 0.82 (75:25 PE/EtOAc); EI MS 218 (100%, [M – Br]^+^), 189 (10%), 108 (16%); HRMS (ESI/QTOF) *m*/*z* [M – Br]^+^ calcd for [C_16_H_12_N]^+^ 218.0964, found 218.0966 (100%).

#### 2-(Bromo­(mesityl)­methyl)­pyridine (**8u**)

GP2_B_ was followed with **7u** (1.50 g, 6.60 mmol), triphenylphosphine (3.12 g, 11.88 mmol), tetrabromomethane (3.28 g, 9.90 mmol), and 44 mL of THF. The resulting product was purified by column chromatography (PE/EtOAc, gradient from 100:0 to 85:15), yielding 1.35 g of bromide **8u** (4.65 mmol, 71%) as a pink amorphous solid: ^1^H NMR (400 MHz, CDCl_3_) δ 8.64–8.52 (m, 1H), 7.63 (dtd, *J* = 15.5, 8.0, 1.5 Hz, 2H), 7.16 (ddt, *J* = 7.2, 4.9, 1.2 Hz, 1H), 6.86 (s, 2H), 6.79 (s, 1H), 2.27 (s, 3H), 2.19 (s, 6H); ^13^C­{^1^H} NMR (101 MHz, CDCl_3_) δ 158.6, 149.3, 138.4, 137.4, 136.7, 134.4, 130.4, 122.6, 121.9, 52.0, 21.1, 20.8; *R_f_* = 0.3 (91:9 PE/EtOAc); EI MS 210 (100%, [M – Br]^+^), 195 (86%), 180 (14%); HRMS (ESI/QTOF) *m*/*z* [M + H]^+^ calcd for [C_15_H_17_^79^BrN]^+^ 290.0539, found 290.0530 (5%).

#### Ethyl (Phenyl­(pyridin-2-yl)­methyl)­(phenylethynyl)­phosphinate (**1p**)

GP3_B_ was followed with phosphonite **6a** (385 mg, 1.73 mmol) and bromide **8p** (430 mg, 1.73 mmol). The resulting product was purified by column chromatography (CHCl_3_/EtOAc/MeOH, gradient from 50:50:0 to 48:48:4), yielding 249 mg of phosphinate **1p** (0.689 mmol, 40%) as a brown oil, obtained as a diastereomeric mixture: ^1^H NMR (400 MHz, CDCl_3_) δ 8.53 (dt, *J* = 4.9, 1.9 Hz, 1H), 7.71–7.53 (m, 4H), 7.37–7.21 (m, 8H), 7.12 (dddt, *J* = 6.4, 5.0, 3.0, 1.7 Hz, 1H), 4.78 (dd, *J* = 20.9, 3.0 Hz, 1H), 4.21–4.00 (m, 2H), 1.21 (td, *J* = 7.1, 1.1 Hz, 3H); ^13^C­{^1^H} NMR (101 MHz, CDCl_3_) δ 156.0, 149.5, 137.2–136.3 (m), 134.7 (dd, *J* = 20.5, 5.9 Hz), 132.3 (2C), 130.5, 130.04, 129.97, 128.5 (2C), 128.4 (2C), 127.5, 124.3 (dd, *J* = 5.2, 3.4 Hz), 122.2 (d, *J* = 2.0 Hz), 119.6 (d, *J* = 4.4 Hz), 101.8 (dd, *J* = 36.1, 18.1 Hz), 81.1 (dd, *J* = 206.9, 21.6 Hz), 62.8 (d, *J* = 7.7 Hz), 58.0 (dd, *J* = 113.4, 23.4 Hz), 16.2 (d, *J* = 6.4 Hz) (some proton and carbon signals were split due to phosphorus coupling and the presence of two diastereomers, hindering unambiguous spectral interpretation); ^31^P­{^1^H} NMR (162 MHz, CDCl_3_) δ 17.80, 17.13; *R_f_* = 0.81 (EtOAc); EI MS 361 (24%, M^+^), 298 (24%), 268 (76%), 167 (100%); HRMS (ESI/QTOF) *m*/*z* [M + H]^+^ calcd for [C_22_H_21_NO_2_P]^+^ 362.1304, found 362.1304 (100%).

#### Ethyl ((4-Methoxyphenyl)­(pyridin-2-yl)­methyl)­(phenylethynyl)­phosphinate (**1q**)

A Schlenk flask was charged with alcohol **7q** (1.00 g, 4.65 mmol), phosphorus tribromide (0.219 mL, 2.32 mmol), and 23 mL of dry DCM. The reaction mixture was stirred at room temperature for 1 h. Subsequently, the reaction was quenched by adding a 10% aqueous solution of K_2_CO_3_. The aqueous phase was extracted with DCM (3 × 10 mL), and the combined organic layers were dried over anhydrous MgSO_4_, filtered, and then mixed with phosphonite **6a** (1.03 g, 4.65 mmol). The solvent was subsequently distilled off at atmospheric pressure, and the resulting oily residue was heated at 100 °C in an oil bath for 2 h. The resulting product was purified by column chromatography (PE/EtOAc, gradient from 50:50 to 100:0), yielding 146 mg of phosphinate **1q** (0.373 mmol, 8%) as a brown oil, obtained as a diastereomeric mixture: ^1^H NMR (400 MHz, CDCl_3_) δ 8.65–8.45 (m, 1H), 7.71 (ddd, *J* = 8.9, 7.8, 1.5 Hz, 1H), 7.69–7.63 (m, 1H), 7.57 (ddd, *J* = 11.0, 8.8, 2.2 Hz, 2H), 7.45–7.27 (m, 5H), 7.22–7.14 (m, 1H), 6.88 (d, *J* = 8.2 Hz, 2H), 4.79 (dd, *J* = 20.9, 2.9 Hz, 1H), 4.33–4.12 (m, 2H), 3.78 (s, 3H), 1.32–1.24 (m, 3H); ^13^C­{^1^H} NMR (101 MHz, CDCl_3_) δ 159.2 (d, *J* = 2.7 Hz), 156.7–156.3 (m), 149.7, 136.8 (d, *J* = 5.8 Hz), 132.6 (2C), 131.3 (dd, *J* = 7.6, 1.5 Hz, 2C), 130.6, 128.6 (d, *J* = 2.2 Hz, 2C), 127.2–126.6 (m), 124.4 (dd, *J* = 5.2, 1.8 Hz), 122.3, 120.0 (dd, *J* = 4.3, 2.0 Hz), 114.2 (2C), 101.9 (dd, *J* = 35.6, 6.2 Hz), 81.4 (dd, *J* = 205.6, 19.0 Hz), 63.0 (d, *J* = 7.7 Hz), 57.3 (dd, *J* = 113.8, 24.1 Hz), 55.4, 16.4 (dd, *J* = 6.5, 2.3 Hz); ^31^P­{^1^H} NMR (162 MHz, CDCl_3_) δ 18.02, 17.38; *R_f_* = 0.56 (EtOAc); EI MS 391 (20%, M^+^), 299 (16%), 198 (100%), 182 (16%), 167 (34%), 154 (16%); HRMS (ESI/QTOF) *m*/*z* [M + H]^+^ calcd for [C_23_H_23_NO_3_P]^+^ 392.1410, found 392.1408 (100%).

#### Ethyl (Phenylethynyl)­(pyridin-2-yl­(4-(trifluoromethyl)­phenyl)­methyl)­phosphinate (**1r**)

GP3_B_ was followed with phosphonite **6a** (668 mg, 3.01 mmol) and bromide **8r** (950 mg, 3.01 mmol). The resulting product was purified by column chromatography (PE/EtOAc, gradient from 100:0 to 50:50), yielding 515 mg of phosphinate **1r** (1.20 mmol, 40%) as a brown oil, obtained as a diastereomeric mixture: ^1^H NMR (400 MHz, CDCl_3_) δ 8.62 (ddd, *J* = 4.8, 2.8, 1.3 Hz, 1H), 7.80 (ddd, *J* = 11.0, 8.2, 2.2 Hz, 2H), 7.73–7.63 (m, 2H), 7.60 (d, *J* = 8.2 Hz, 2H), 7.46–7.38 (m, 1H), 7.37–7.29 (m, 4H), 7.25–7.18 (m, 1H), 4.89 (dd, *J* = 21.0, 2.4 Hz, 1H), 4.20 (tt, *J* = 8.5, 6.4 Hz, 2H), 1.29 (td, *J* = 7.1, 2.5 Hz, 3H); ^13^C­{^1^H} NMR (101 MHz, CDCl_3_) δ 156.4–154.0 (m), 149.8, 139.3–138.6 (m), 137.0 (d, *J* = 5.0 Hz), 132.5 (t, *J* = 1.9 Hz, 2C), 130.8 (d, *J* = 3.4 Hz), 130.6 (dd, *J* = 7.2, 2.6 Hz, 2C), 130.1–129.5 (m), 128.6 (d, *J* = 3.1 Hz, 2C), 126.3–125.2 (m, 2C), 124.6 (d, *J* = 5.2 Hz), 122.7 (d, *J* = 1.9 Hz), 119.5 (dd, *J* = 4.5, 2.4 Hz), 102.5 (dd, *J* = 36.7, 18.7 Hz), 80.7 (dd, *J* = 209.5, 14.5 Hz), 63.1 (dd, *J* = 7.6, 5.3 Hz), 57.8 (dd, *J* = 113.3, 10.6 Hz), 16.2 (d, *J* = 6.5 Hz) (the signal of the CF_3_ group could not be unambiguously assigned due to its low intensity and overlap with other signals); ^31^P­{^1^H} NMR (162 MHz, CDCl_3_) δ 16.52, 16.02; ^19^F­{^1^H} NMR (376 MHz, CDCl_3_) δ −62.59, −62.59; *R_f_* = 0.22 (50:50 PE/EtOAc); EI MS 429 (25%, M^+^), 400 (18%), 385 (13%), 366 (37%), 336 (100%), 235 (75%), 216 (29%), 165 (96%), 102 (32%); HRMS (ESI/QTOF) *m*/*z* [M + H]^+^ calcd for [C_23_H_20_F_3_NO_2_P]^+^ 430.1178, found 430.1179 (100%).

#### Ethyl ((2,5-Dimethylphenyl)­(pyridin-2-yl)­methyl)­(phenylethynyl)­phosphinate (**1s**)

GP3_B_ was followed with phosphonite **6a** (483 mg, 2.17 mmol) and bromide **8s** (600 mg, 2.17 mmol). The resulting product was purified by column chromatography (PE/EtOAc, gradient from 50:50 to 25:75), yielding 306 mg of a 55:45 diastereomeric mixture of phosphinate **1s** (0.786 mmol, 36%) as a brown oil: ^1^H NMR (400 MHz, CDCl_3_) δ 8.58 (d, *J* = 4.7 Hz, 2H), 7.83 (d, *J* = 2.1 Hz, 1H), 7.75 (d, *J* = 2.2 Hz, 1H), 7.69–7.56 (m, 4H), 7.43–7.28 (m, 10H), 7.20–7.13 (m, 2H), 7.07 (d, *J* = 7.4 Hz, 2H), 6.98 (d, *J* = 7.7 Hz, 2H), 5.16 (d, *J* = 1.9 Hz, 1H), 5.11 (d, *J* = 1.6 Hz, 1H), 4.25–4.10 (m, 4H), 2.42 (s, 6H), 2.32 (s, 6H), 1.28 (t, *J* = 7.0 Hz, 6H); ^13^C­{^1^H} NMR (101 MHz, CDCl_3_) δ 156.7, 156.5 (d, *J* = 5.3 Hz), 149.5 (2C), 136.8 (d, *J* = 4.3 Hz, 2C), 135.8 (d, *J* = 2.4 Hz), 135.7 (d, *J* = 2.2 Hz), 134.2 (dd, *J* = 10.0, 6.7 Hz, 2C), 133.5 (d, *J* = 3.6 Hz), 133.4 (d, *J* = 5.9 Hz), 132.7–132.3 (m, 4C), 130.9–130.3 (m, 6C), 128.6 (d, *J* = 2.6 Hz, 4C), 128.5 (t, *J* = 2.5 Hz, 2C), 124.7 (dd, *J* = 5.4, 3.1 Hz, 2C), 122.3 (2C), 120.0 (d, *J* = 4.4 Hz, 2C), 101.4 (d, *J* = 36.2 Hz, 2C), 81.6 (d, *J* = 205.3 Hz, 2C), 62.9 (d, *J* = 3.5 Hz), 62.8 (d, *J* = 3.6 Hz), 53.4 (dd, *J* = 115.1, 20.0 Hz, 2C), 21.4 (2C), 20.0 (d, *J* = 3.6 Hz, 2C), 16.3 (d, *J* = 6.5 Hz, 2C); ^31^P­{^1^H} NMR (162 MHz, CDCl_3_) δ 18.07, 17.84; *R_f_* = 0.69 (EtOAc); EI MS 389 (27%, M^+^), 360 (15%), 297 (93%), 220 (32%), 206 (50%), 194 (82%), 181 (100%), 165 (27%), 102 (23%); HRMS (ESI/QTOF) *m*/*z* [M + H]^+^ calcd for [C_24_H_25_NO_2_P]^+^ 390.1617, found 390.1618 (100%).

#### Ethyl (Naphthalen-1-yl­(pyridin-2-yl)­methyl)­(phenylethynyl)­phosphinate (**1t**)

GP3_B_ was followed with phosphonite **6a** (1.19 g, 5.37 mmol) and bromide **8t** (1.60 g, 5.37 mmol). The resulting product was purified by column chromatography (PE/EtOAc, gradient from 75:25 to 30:70), yielding 740 mg of a 51:49 diastereomeric mixture of phosphinate **1t** (1.80 mmol, 34%) as a brown oil: ^1^H NMR (400 MHz, CDCl_3_) δ 8.66–8.56 (m, 2H), 8.41 (ddd, *J* = 7.3, 2.4, 1.2 Hz, 1H), 8.35 (ddd, *J* = 7.3, 2.5, 1.2 Hz, 1H), 8.31 (d, *J* = 8.4 Hz, 2H), 7.88–7.77 (m, 4H), 7.73–7.66 (m, 1H), 7.64–7.42 (m, 9H), 7.42–7.36 (m, 1H), 7.36–7.27 (m, 5H), 7.25–7.19 (m, 2H), 7.19–7.08 (m, 4H), 5.74 (dd, *J* = 21.8, 2.2 Hz, 2H), 4.20 (dqd, *J* = 8.8, 7.1, 5.7 Hz, 4H), 1.28 (t, *J* = 7.0 Hz, 3H), 1.22 (t, *J* = 7.1 Hz, 3H); ^13^C­{^1^H} NMR (101 MHz, CDCl_3_) δ 156.5 (d, *J* = 3.2 Hz), 156.3 (d, *J* = 6.2 Hz), 149.4, 149.4, 136.8 (d, *J* = 1.8 Hz), 136.8 (d, *J* = 1.8 Hz), 134.3, 132.6 (d, *J* = 2.1 Hz, 2C), 132.5 (d, *J* = 1.9 Hz, 2C), 132.4, 132.3, 131.6 (d, *J* = 2.6 Hz), 131.5 (d, *J* = 5.2 Hz), 130.5 (d, *J* = 9.3 Hz, 2C), 129.0 (2C), 128.5 (2C), 128.4 (d, *J* = 1.9 Hz, 2C), 128.4 (2C), 128.1 (d, *J* = 8.0 Hz), 128.0 (d, *J* = 7.4 Hz), 126.6 (2C), 125.7 (2C), 125.6 (d, *J* = 2.0 Hz), 125.6 (d, *J* = 1.9 Hz), 124.8 (d, *J* = 2.9 Hz), 124.8 (d, *J* = 2.5 Hz), 123.8 (2C), 122.3 (d, *J* = 2.4 Hz, 2C), 119.6 (d, *J* = 4.4 Hz), 119.7 (d, *J* = 4.4 Hz), 102.0 (d, *J* = 21.8 Hz), 101.6 (d, *J* = 21.8 Hz), 81.5 (dd, *J* = 207.7, 3.6 Hz, 2C), 63.0 (d, *J* = 7.6 Hz), 62.9 (d, *J* = 7.6 Hz), 52.9 (d, *J* = 115.3 Hz, 2C), 16.3 (d, *J* = 2.9 Hz), 16.3 (d, *J* = 3.1 Hz) (one signal could not be unambiguously assigned due to spectral overlap); ^31^P­{^1^H} NMR (162 MHz, CDCl_3_) δ 17.82, 17.42; *R_f_* = 0.19 (50:50 PE/EtOAc); HRMS (ESI/QTOF) *m*/*z* [M + H]^+^ calcd for [C_26_H_23_NO_2_P]^+^ 412.1461, found 412.1460 (100%).

#### Ethyl (Mesityl­(pyridin-2-yl)­methyl)­(phenylethynyl)­phosphinate (**1u**)

GP3_B_ was followed with phosphonite **6a** (766 mg, 3.45 mmol) and bromide **8u** (1.00 g, 3.45 mmol). The resulting product was purified by column chromatography (PE/EtOAc, gradient from 75:25 to 25:75), yielding 380 mg of a 54:46 diastereomeric mixture of phosphinate **1u** (0.942 mmol, 27%) as a brown oil: ^1^H NMR (400 MHz, CDCl_3_) δ 8.65–8.57 (m, 2H), 7.59–7.50 (m, 2H), 7.42–7.33 (m, 3H), 7.33–7.21 (m, 8H), 7.11 (dd, *J* = 7.5, 4.9 Hz, 2H), 6.99–6.76 (m, 4H), 5.41 (d, *J* = 25.7 Hz, 1H), 5.36 (d, *J* = 27.0 Hz, 1H), 4.56–4.28 (m, 2H), 4.24–4.01 (m, 3H), 2.52 (s, 6H), 2.24 (d, *J* = 2.2 Hz, 6H), 2.10 (s, 3H), 2.02 (s, 3H), 1.40 (t, *J* = 7.1 Hz, 3H), 1.23 (t, *J* = 7.0 Hz, 3H); ^13^C­{^1^H} NMR (101 MHz, CDCl_3_) δ 157.9 (d, *J* = 1.5 Hz), 157.5 (d, *J* = 3.8 Hz), 149.0, 148.8, 139.2 (2C), 138.3 (2C), 137.1 (d, *J* = 3.6 Hz), 136.9 (d, *J* = 3.3 Hz), 136.2, 136.1 (d, *J* = 1.4 Hz), 132.3 (d, *J* = 2.0 Hz, 2C), 132.2 (d, *J* = 2.0 Hz, 2C), 131.0 (2C), 130.2, 130.1, 129.7 (d, *J* = 4.4 Hz), 129.5 (d, *J* = 7.6 Hz), 129.1 (2C), 128.34 (2C), 128.29 (2C), 123.4 (d, *J* = 8.6 Hz), 123.3 (d, *J* = 7.8 Hz), 121.4 (2C), 120.2 (d, *J* = 4.2 Hz), 120.0 (d, *J* = 4.4 Hz), 99.8 (d, *J* = 35.0 Hz), 99.4 (d, *J* = 36.1 Hz), 83.9 (d, *J* = 105.0 Hz), 81.9 (d, *J* = 106.7 Hz), 62.6 (d, *J* = 7.4 Hz), 62.0 (d, *J* = 7.2 Hz), 52.7 (d, *J* = 120.4 Hz), 52.0 (d, *J* = 119.2 Hz), 22.0–21.4 (m, 5C), 20.8, 16.3 (d, *J* = 6.6 Hz), 16.2 (d, *J* = 6.9 Hz); ^31^P­{^1^H} NMR (162 MHz, CDCl_3_) δ 18.69, 17.54; *R_f_* = 0.43–0.47 (67:33 PE/EtOAc); EI MS 403 (22%, M^+^), 374 (19%), 311 (100%), 234 (20%), 220 (32%), 208 (65%), 195 (58%); HRMS (ESI/QTOF) *m*/*z* [M + H]^+^ calcd for [C_25_H_27_NO_2_P]^+^ 404.1774, found 404.1774 (100%).

#### 2-Ethoxy-1,4-diphenylpyrido­[1,2-*a*]­[1,4]­azaphosphinine 2-Oxide (**2p**)

GP4_B_ was followed with **1p** (240 mg, 0.664 mmol), CF_3_COOAg (2.9 mg, 13 μmol), and 35 mL of DCM. The resulting product was purified by column chromatography (CHCl_3_/MeOH, gradient from 100:0 to 96:4), yielding 228 mg of 1,4-azaphosphinine **2p** (0.631 mmol, 95%) as a yellow amorphous solid: ^1^H NMR (400 MHz, CDCl_3_) δ 7.74–7.30 (m, 10H), 7.09 (d, *J* = 7.6 Hz, 1H), 6.83–6.75 (m, 1H), 6.49 (dddd, *J* = 9.9, 6.2, 2.7, 1.3 Hz, 1H), 5.86–5.76 (m, 2H), 3.89 (ddq, *J* = 10.4, 9.2, 7.0 Hz, 1H), 3.82–3.65 (m, 1H), 1.02 (t, *J* = 7.0 Hz, 3H); ^13^C­{^1^H} NMR δ 150.9 (d, *J* = 2.3 Hz), 145.0 (d, *J* = 7.8 Hz), 137.1 (d, *J* = 12.4 Hz), 134.2, 131.7, 129.6, 129.2, 128.9, 128.5, 127.3 (d, *J* = 1.5 Hz), 126.5 (d, *J* = 1.6 Hz), 123.0 (d, *J* = 11.1 Hz), 106.9, 104.6 (d, *J* = 125.7 Hz), 102.7 (d, *J* = 139.3 Hz), 62.3 (d, *J* = 6.4 Hz), 16.5 (d, *J* = 6.2 Hz) (two signals were not observed, most likely due to broadening caused by phenyl group rotation); ^31^P­{^1^H} NMR (162 MHz, CDCl_3_) δ 14.94; *R_f_* = 0.18 (EtOAc); EI MS 361 (<3%, M^+^), 269 (100%); HRMS (ESI/QTOF) *m*/*z* [M + H]^+^ calcd for [C_22_H_21_NO_2_P]^+^ 362.1304, found 362.1303 (100%).

#### 2-Ethoxy-1-(4-methoxyphenyl)-4-phenylpyrido­[1,2-*a*]­[1,4]­azaphosphinine 2-Oxide (**2q**)

GP4_B_ was followed with **1q** (60.0 mg, 0.153 mmol), CF_3_COOAg (3.4 mg, 15 μmol), and 3 mL of DCM. The resulting product was purified by column chromatography (EtOAc/MeOH, gradient from 100:0 to 97:3), yielding 55.0 mg of 1,4-azaphosphinine **2q** (0.141 mmol, 92%) as a yellow amorphous solid: ^1^H NMR (400 MHz, CDCl_3_) δ 7.52–7.36 (m, 7H), 7.07 (d, *J* = 7.6 Hz, 1H), 6.98 (d, *J* = 9.1 Hz, 2H), 6.82–6.76 (m, 1H), 6.48 (dddd, *J* = 9.8, 6.2, 2.8, 1.3 Hz, 1H), 5.84–5.74 (m, 2H), 3.95–3.86 (m, 1H), 3.84 (s, 3H), 3.79–3.71 (m, 1H), 1.05 (t, *J* = 7.1 Hz, 3H); ^13^C­{^1^H} NMR δ 159.1 (d, *J* = 1.7 Hz), 151.2 (d, *J* = 2.5 Hz), 145.4 (d, *J* = 8.7 Hz), 137.3 (d, *J* = 12.5 Hz), 132.7 (2C), 131.8, 129.7, 129.4 (2C), 128.7 (2C), 126.5 (d, *J* = 1.7 Hz), 126.1, 123.2 (d, *J* = 11.1 Hz), 114.5 (2C), 107.0, 104.4 (d, *J* = 125.1 Hz), 102.1 (d, *J* = 140.5 Hz), 62.4 (d, *J* = 6.5 Hz), 55.4, 16.7 (d, *J* = 6.3 Hz); ^31^P­{^1^H} NMR (162 MHz, CDCl_3_) δ 15.18; *R_f_* = 0.23 (EtOAc); EI MS 391 (6%, M^+^), 299 (100%), 284 (57%); HRMS (ESI/QTOF) *m*/*z* [M + H]^+^ calcd for [C_23_H_23_NO_3_P]^+^ 392.1410, found 392.1412 (100%).

#### 2-Ethoxy-4-phenyl-1-(4-(trifluoromethyl)­phenyl)­pyrido­[1,2-*a*]­[1,4]­azaphosphinine 2-Oxide (**2r**)

GP4_B_ was followed with **1r** (100 mg, 0.233 mmol), CF_3_COOAg (2.6 mg, 12 μmol), and 1.5 mL of DCM. The resulting product was purified by column chromatography (EtOAc/MeOH, gradient from 100:0 to 96:4), yielding 89.0 mg of 1,4-azaphosphinine **2r** (0.207 mmol, 89%) as a yellow solid. Single crystals suitable for X-ray analysis were obtained by slowly cooling its EtOAc solution: ^1^H NMR (400 MHz, CDCl_3_) 7.88–7.46 (m, 7H), 7.46–7.35 (m, 2H), 7.13 (dt, *J* = 7.7, 1.2 Hz, 1H), 6.74 (dd, *J* = 9.8, 1.4 Hz, 1H), 6.60–6.51 (m, 1H), 5.92–5.80 (m, 2H), 3.90 (ddq, *J* = 10.4, 8.8, 7.1 Hz, 1H), 3.84–3.65 (m, 1H), 1.05 (t, *J* = 7.0 Hz, 3H); ^13^C­{^1^H} NMR (101 MHz, CDCl_3_) δ 151.2 (d, *J* = 2.2 Hz), 145.4 (d, *J* = 7.1 Hz), 138.6, 137.1 (d, *J* = 12.5 Hz), 132.1 (3C), 129.9, 129.5 (2C), 128.7 (2C), 127.5 (d, *J* = 1.7 Hz), 125.9 (2C), 124.4 (q, *J* = 271.9 Hz), 122.7 (d, *J* = 11.1 Hz), 107.3, 105.4 (d, *J* = 126.2 Hz), 101.2 (d, *J* = 139.4 Hz), 62.5 (d, *J* = 6.4 Hz), 16.7 (d, *J* = 6.1 Hz); ^31^P­{^1^H} NMR (162 MHz, CDCl_3_) δ 14.50; ^19^F­{^1^H} NMR (376 MHz, CDCl_3_) δ −62.47; *R_f_* = 0.33 (EtOAc); EI MS 429 (<2%, M^+^), 337 (100%); HRMS (ESI/QTOF) *m*/*z* [M + H]^+^ calcd for [C_23_H_20_F_3_NO_2_P]^+^ 430.1178, found 430.1176 (100%); mp 155.4–156.9 °C (EtOAc).

#### 1-(2,5-Dimethylphenyl)-2-ethoxy-4-phenylpyrido­[1,2-*a*]­[1,4]­azaphosphinine 2-Oxide (**2s**)

GP4_B_ was followed with **1s** (100 mg, 0.257 mmol), CF_3_COOAg (2.8 mg, 13 μmol), and 5 mL of DCM. The resulting product was purified by column chromatography (CHCl_3_/MeOH, gradient from 100:0 to 96:4), yielding 94.0 mg of a 1:1.1 diastereomeric mixture of 1,4-azaphosphinine **2s** (0.241 mmol, 94%) as a yellow amorphous solid: ^1^H NMR (400 MHz, CDCl_3_) δ 7.54–7.39 (m, 10H), 7.34 (d, *J* = 2.2 Hz, 1H), 7.18 (dd, *J* = 10.3, 7.6 Hz, 2H), 7.12–6.99 (m, 5H), 6.52–6.43 (m, 2H), 6.42–6.34 (m, 2H), 5.86–5.73 (m, 4H), 4.03–3.79 (m, 3H), 3.72 (ddq, *J* = 10.3, 8.8, 7.1 Hz, 1H), 2.36 (s, 3H), 2.34 (s, 3H), 2.33 (s, 3H), 2.20 (s, 3H), 1.16 (t, *J* = 7.0 Hz, 3H), 0.98 (t, *J* = 7.1 Hz, 3H); ^13^C­{^1^H} NMR (101 MHz, CDCl_3_) δ 150.9 (dd, *J* = 19.0, 2.6 Hz), 144.3 (dd, *J* = 34.0, 8.3 Hz), 137.3 (d, *J* = 5.7 Hz), 137.2 (d, *J* = 5.6 Hz), 137.0 (d, *J* = 3.6 Hz), 135.7 (d, *J* = 1.9 Hz), 135.3 (d, *J* = 1.8 Hz), 134.8 (d, *J* = 4.1 Hz), 133.8 (d, *J* = 3.0 Hz), 132.8, 132.4, 132.4 (d, *J* = 3.3 Hz), 131.8, 131.7, 130.5 (d, *J* = 1.6 Hz), 130.1 (d, *J* = 1.5 Hz), 129.6, 129.3, 128.7, 128.6, 126.6 (dd, *J* = 6.3, 1.7 Hz), 122.9 (t, *J* = 12.1 Hz), 106.6 (d, *J* = 2.2 Hz), 105.6 (d, *J* = 2.8 Hz), 104.4, 102.4 (d, *J* = 140.8 Hz), 102.3 (d, *J* = 140.8 Hz), 62.1 (d, *J* = 3.6 Hz), 62.1 (d, *J* = 3.6 Hz), 21.1, 20.9, 19.7, 19.3, 16.8 (d, *J* = 6.4 Hz), 16.6 (d, *J* = 5.6 Hz) (some proton and carbon signals were split due to phosphorus coupling and the presence of two diastereomers, hindering unambiguous spectral interpretation); ^31^P­{^1^H} NMR (162 MHz, CDCl_3_) δ 15.06, 14.41; *R_f_* = 0.38 (EtOAc); EI MS 389 (3%, M^+^), 297 (100%); HRMS (ESI/QTOF) *m*/*z* [M + H]^+^ calcd for [C_24_H_25_NO_2_P]^+^ 390.1617, found 390.1620 (100%).

#### 2-Ethoxy-1-(naphthalen-1-yl)-4-phenylpyrido­[1,2-*a*]­[1,4]­azaphosphinine 2-Oxide (**2t**)

GP4_B_ was followed with **1t** (250 mg, 0.608 mmol), CF_3_COOAg (2.7 mg, 12 μmol), and 4 mL of DCM. The resulting product was purified by column chromatography (EtOAc/MeOH, gradient from 100:0 to 97:3), yielding 80 mg of 1,4-azaphosphinine (*R*_*a*_,*R*)/(*S*_*a*_ *,S*)-**2t** (0.194 mmol, 32%) as a yellow solid and 130 mg of 1,4-azaphosphinine (*S*_*a*_,*R*)/(*R*_*a*_ *,S*)-**2t** (0.316 mmol, 52%) as a yellow amorphous solid. Single crystals of both diastereomers suitable for X-ray analysis were obtained by slowly cooling their EtOAc solution.

##### (*R_a_,R*)/(*S_a_,S*)-**2t**

^1^H NMR (400 MHz, CDCl_3_) δ 8.14 (dd, *J* = 6.2, 3.6 Hz, 1H), 7.87 (dd, *J* = 6.2, 3.5 Hz, 2H), 7.57–7.43 (m, 9H), 7.13 (d, *J* = 7.6 Hz, 1H), 6.36 (dddd, *J* = 8.8, 6.1, 2.7, 1.3 Hz, 1H), 6.22 (dt, *J* = 9.6, 1.2 Hz, 1H), 5.89 (d, *J* = 1.1 Hz, 1H), 5.79 (ddd, *J* = 7.6, 6.0, 1.6 Hz, 1H), 3.96 (ddq, *J* = 10.3, 8.5, 7.0 Hz, 1H), 3.81 (ddq, *J* = 10.3, 8.8, 7.1 Hz, 1H), 1.14 (t, *J* = 7.1 Hz, 3H); ^13^C­{^1^H} NMR (101 MHz, CDCl_3_) δ 151.0 (d, *J* = 2.2 Hz), 145.6 (d, *J* = 8.1 Hz), 137.1 (d, *J* = 12.5 Hz), 134.2, 134.2, 131.6, 131.5, 129.7, 129.3, 129.3, 128.7, 128.4 (d, *J* = 2.1 Hz), 128.1, 126.71, 126.67 (d, *J* = 1.8 Hz), 126.6, 126.2, 125.6 (d, *J* = 2.1 Hz), 123.2 (d, *J* = 11.4 Hz), 106.7, 105.2 (d, *J* = 125.1 Hz), 100.3 (d, *J* = 141.0 Hz), 62.1 (d, *J* = 6.3 Hz), 16.9 (d, *J* = 6.2 Hz) (two signals are missing due to overlapping); ^31^P­{^1^H} NMR (162 MHz, CDCl_3_) δ 14.31; *R_f_* = 0.28 (EtOAc); HRMS (ESI/QTOF) *m*/*z* [M + H]^+^ calcd for [C_26_H_23_NO_2_P]^+^ 412.1461, found 412.1462 (100%); mp 196.1–197.2 °C (EtOAc).

##### (*S_a_,R*)/(*R_a_,S*)-**2t**

^1^H NMR (400 MHz, CDCl_3_) δ 7.95–7.84 (m, 3H), 7.80 (ddd, *J* = 7.1, 2.2, 1.2 Hz, 1H), 7.64–7.40 (m, 8H), 7.15 (dt, *J* = 7.6, 1.1 Hz, 1H), 6.39 (dddd, *J* = 9.9, 6.1, 2.7, 1.3 Hz, 1H), 6.26 (dt, *J* = 9.5, 1.3 Hz, 1H), 5.91 (d, *J* = 1.1 Hz, 1H), 5.81 (ddd, *J* = 7.7, 6.1, 1.6 Hz, 1H), 3.71 (ddq, *J* = 10.3, 9.2, 7.0 Hz, 1H), 3.43 (ddq, *J* = 10.2, 8.7, 7.1 Hz, 1H), 0.62 (t, *J* = 7.0 Hz, 3H); ^13^C­{^1^H} NMR (101 MHz, CDCl_3_) δ 151.2 (d, *J* = 2.6 Hz), 145.2 (d, *J* = 8.0 Hz), 137.2 (d, *J* = 12.5 Hz), 134.2, 132.6 (d, *J* = 3.7 Hz), 131.8, 131.1 (d, *J* = 3.9 Hz), 130.4, 129.8, 129.4, 128.8, 128.6, 128.2 (d, *J* = 2.0 Hz), 126.8 (d, *J* = 1.8 Hz), 126.3 (d, *J* = 2.2 Hz), 126.2, 126.0, 125.8, 123.5 (d, *J* = 11.6 Hz), 106.9, 105.0 (d, *J* = 127.3 Hz), 100.2 (d, *J* = 140.2 Hz), 62.4 (d, *J* = 6.5 Hz), 16.3 (d, *J* = 5.8 Hz); ^31^P­{^1^H} NMR (162 MHz, CDCl_3_) δ 14.90; *R_f_* = 0.16 (EtOAc); HRMS (ESI/QTOF) *m*/*z* [M + H]^+^ calcd for [C_26_H_23_NO_2_P]^+^ 412.1461, found 412.1464 (100%).

#### 2-Ethoxy-1-mesityl-4-phenylpyrido­[1,2-*a*]­[1,4]­azaphosphinine 2-Oxide (**2u**)

GP4_B_ was followed with **1u** (130 mg, 0.322 mmol), CF_3_COOAg (3.6 mg, 16 μmol), and 6.4 mL of DCM. The resulting product was purified by column chromatography (CHCl_3_/MeOH, gradient from 100:0 to 96:4), yielding 110 mg of 1,4-azaphosphinine **2u** (0.273 mmol, 85%) as a yellow solid: ^1^H NMR (400 MHz, CDCl_3_) δ 7.52–7.39 (m, 5H), 7.08 (d, *J* = 7.5 Hz, 1H), 6.96 (d, *J* = 14.9 Hz, 2H), 6.46 (dddd, *J* = 10.2, 6.1, 2.8, 1.3 Hz, 1H), 6.35–6.27 (m, 1H), 5.83–5.71 (m, 2H), 3.96 (ddq, *J* = 10.3, 8.6, 7.1 Hz, 1H), 3.75 (ddq, *J* = 10.3, 8.4, 7.0 Hz, 1H), 2.38 (s, 3H), 2.31 (s, 3H), 2.21 (s, 3H), 1.07 (t, *J* = 7.1 Hz, 3H); ^13^C­{^1^H} NMR (101 MHz, CDCl_3_) δ 150.9 (d, *J* = 2.6 Hz), 144.1 (d, *J* = 8.7 Hz), 140.5 (d, *J* = 3.0 Hz), 138.5 (d, *J* = 3.6 Hz), 137.4 (d, *J* = 12.5 Hz), 137.2 (d, *J* = 2.1 Hz), 132.0, 129.7, 129.3 (2C), 129.1 (d, *J* = 1.8 Hz), 128.9 (2C), 128.6, 126.7, 122.4 (d, *J* = 11.9 Hz), 106.5, 105.2 (d, *J* = 125.8 Hz), 101.3 (d, *J* = 142.1 Hz), 62.0 (d, *J* = 6.4 Hz), 21.2, 21.0, 20.2, 16.8 (d, *J* = 5.8 Hz) (the signals corresponding to the two equivalent quaternary carbons could not be assigned with certainty); ^31^P­{^1^H} NMR (162 MHz, CDCl_3_) δ 14.89; *R_f_* = 0.33 (EtOAc); EI MS 403 (7%, M^+^), 311 (100%); HRMS (ESI/QTOF) *m*/*z* [M + H]^+^ calcd for [C_25_H_27_NO_2_P]^+^ 404.1774, found 404.1778 (100%); mp 145.1–150.3 °C (EtOAc).

### General Procedure for the Preparation of Phosphinates **3a–m** (GP2_c_)

A round-bottom flask was charged with the appropriate bromomethyl azaaromatic (1 equiv) and phosphonite **6a** (1 equiv). The resulting reaction mixture was heated at 100 °C in an oil bath for approximately 2 h. Upon consumption of the starting materials, as monitored by GC-MS, the crude mixture was purified by column chromatography to afford the desired phosphinates **3a**–**m**.

### General Procedure for the Preparation of 1,4-Azaphosphinines **4a–m** (GP3_c_)

A Schlenk flask was charged with phosphinates **3a**–**m** (1 equiv), silver­(I) trifluoroacetate (0.02–0.05 equiv), and dry DCM (0.05–0.15 M) under an inert atmosphere. The resulting mixture was stirred overnight. Upon consumption of the starting material, as monitored by GC-MS or TLC, the crude product was purified by column chromatography to afford the desired 1,4-azaphosphinines **4a–m** as an oil, which typically solidified over time or upon treatment with EtOAc.

#### Ethyl (Phenylethynyl)­(pyrazin-2-ylmethyl)­phosphinate (**3a**)

GP2_C_ was followed with 2-(bromomethyl)­pyrazine (150 mg, 0.867 mmol) and phosphonite **6a** (193 mg, 0.867 mmol). The resulting product was purified by column chromatography (CHCl_3_/EtOAc/MeOH, gradient from 50:50:0 to 48:48:4), yielding 86 mg of phosphinate **3a** (0.30 mmol, 35%) as a brown oil: ^1^H NMR (400 MHz, CDCl_3_) δ 8.73–8.65 (m, 1H), 8.55 (d, *J* = 2.2 Hz, 1H), 8.52–8.44 (m, 1H), 7.52–7.40 (m, 3H), 7.40–7.31 (m, 2H), 4.34–4.15 (m, 2H), 3.63 (dd, *J* = 20.8, 1.5 Hz, 2H), 1.37 (td, *J* = 7.1, 1.9 Hz, 3H); ^13^C­{^1^H} NMR (101 MHz, CDCl_3_) δ 148.5 (d, *J* = 8.7 Hz), 146.0 (d, *J* = 5.2 Hz), 144.5 (d, *J* = 2.9 Hz), 143.2 (d, *J* = 3.8 Hz), 132.7 (d, *J* = 2.1 Hz, 2C), 131.0, 128.7 (2C), 119.4 (d, *J* = 4.5 Hz), 102.4 (d, *J* = 37.9 Hz), 80.3 (d, *J* = 208.7 Hz), 63.0 (d, *J* = 7.2 Hz), 39.5 (d, *J* = 113.8 Hz), 16.4 (d, *J* = 6.8 Hz); ^31^P­{^1^H} NMR (162 MHz, CDCl_3_) δ 15.07; *R_f_* = 0.56 (93:7 CHCl_3_/MeOH); EI MS 286 (5%, M^+^), 194 (100%); HRMS (ESI/QTOF) *m*/*z* [M + H]^+^ calcd for [C_15_H_16_N_2_O_2_P]^+^ 287.0944, found 287.0943 (100%).

#### Ethyl ((3-Methylpyrazin-2-yl)­methyl)­(phenylethynyl)­phosphinate (**3b**)

GP2_C_ was followed with 2-(bromomethyl)-3-methylpyrazine (490 mg, 2.62 mmol) and phosphonite **6a** (582 mg, 2.62 mmol). The resulting product was purified by column chromatography (CHCl_3_/EtOAc/MeOH, gradient from 50:50:0 to 48:48:4), yielding 310 mg of phosphinate **3b** (1.03 mmol, 39%) as a brown oil: ^1^H NMR (400 MHz, CDCl_3_) δ 8.43–8.28 (m, 2H), 7.58–7.45 (m, 2H), 7.45–7.41 (m, 1H), 7.40–7.31 (m, 2H), 4.42–4.01 (m, 2H), 3.67 (d, *J* = 21.1 Hz, 3H), 2.72 (d, *J* = 1.6 Hz, 3H), 1.37 (t, *J* = 7.1 Hz, 2H); ^13^C­{^1^H} NMR (101 MHz, CDCl_3_) δ 153.9 (d, *J* = 5.7 Hz), 147.0 (d, *J* = 9.9 Hz), 142.4 (d, *J* = 4.1 Hz), 141.8 (d, *J* = 3.3 Hz), 132.6 (d, *J* = 2.1 Hz, 2C), 130.9, 128.6 (2C), 119.4 (d, *J* = 4.4 Hz), 101.9 (d, *J* = 37.6 Hz), 80.7 (d, *J* = 207.0 Hz), 62.8 (d, *J* = 7.3 Hz), 39.0 (d, *J* = 114.0 Hz), 22.4, 16.3 (d, *J* = 6.7 Hz); ^31^P­{^1^H} NMR (162 MHz, CDCl_3_) δ 15.57; *R_f_* = 0.67 (93:7 CHCl_3_/MeOH); EI MS 300 (8%, M^+^), 256 (43%), 207 (100%), 165 (53%), 108 (25%), 102 (34%); HRMS (ESI/QTOF) *m*/*z* [M + H]^+^ calcd for [C_16_H_18_N_2_O_2_P]^+^ 301.1100, found 301.1100 (100%).

#### Ethyl (Phenylethynyl)­(pyrimidin-2-ylmethyl)­phosphinate (**3c**)

GP2_C_ was followed with 2-(bromomethyl)­pyrimidine (1.00 g, 5.78 mmol) and phosphonite **6a** (1.28 g, 5.78 mmol). The resulting product was purified by column chromatography (CHCl_3_/EtOAc/MeOH, gradient from 50:50:0 to 48:48:4), yielding 873 mg of phosphinate **3c** (3.05 mmol, 53%) as a brown oil: ^1^H NMR (400 MHz, CDCl_3_) δ 8.74 (dd, *J* = 4.9, 0.8 Hz, 2H), 7.53–7.47 (m, 2H), 7.47–7.41 (m, 1H), 7.40–7.32 (m, 2H), 7.20 (td, *J* = 5.0, 2.1 Hz, 1H), 4.38–4.18 (m, 2H), 3.83 (d, *J* = 20.9 Hz, 2H), 1.38 (t, *J* = 7.1 Hz, 3H); ^13^C­{^1^H} NMR (101 MHz, CDCl_3_) δ 162.9 (d, *J* = 9.4 Hz), 157.5 (d, *J* = 2.5 Hz), 132.6 (2C), 130.7, 128.6 (2C), 119.7 (d, *J* = 4.4 Hz), 119.3 (d, *J* = 3.1 Hz), 101.5 (d, *J* = 38.5 Hz), 81.0 (d, *J* = 211.2 Hz), 62.7 (d, *J* = 7.1 Hz), 43.5 (d, *J* = 112.6 Hz), 16.3 (d, *J* = 6.6 Hz); ^31^P­{^1^H} NMR (162 MHz, CDCl_3_) 15.03; *R_f_* = 0.41 (92:8 CHCl_3_/MeOH); EI MS 286 (8%, M^+^), 285 (17%), 242 (49%), 194 (100%), 165 (81%), 154 (27%), 124 (54%), 94 (66%); HRMS (ESI/QTOF) *m*/*z* [M + H]^+^ calcd for [C_15_H_16_N_2_O_2_P]^+^ 287.0944, found 287.0940 (100%).

#### Ethyl ((6-Fluoropyridin-2-yl)­methyl)­(phenylethynyl)­phosphinate (**3d** *_ortho_*)

GP2_C_ was followed with 2-(bromomethyl)-6-fluoropyridine (500 mg, 2.63 mmol) and phosphonite **6a** (585 mg, 2.63 mmol). The resulting product was purified by column chromatography (CHCl_3_/EtOAc/MeOH, gradient from 50:50:0 to 48:48:4), yielding 425 mg of phosphinate **3d**_*ortho*_ (1.40 mmol, 53%) as a yellow oil: ^1^H NMR (400 MHz, CDCl_3_) δ 7.80–7.71 (m, 1H), 7.53–7.48 (m, 2H), 7.47–7.41 (m, 1H), 7.39–7.30 (m, 3H), 6.84 (dt, *J* = 8.2, 2.7 Hz, 1H), 4.33–4.16 (m, 2H), 3.55 (d, *J* = 20.8 Hz, 2H), 1.37 (t, *J* = 7.0 Hz, 3H); ^13^C­{^1^H} NMR (101 MHz, CDCl_3_) δ 150.7 (dd, *J* = 13.8, 8.2 Hz), 141.4 (dd, *J* = 7.6, 3.2 Hz), 132.6 (d, *J* = 2.1 Hz), 130.7, 128.5, 122.1 (d, *J* = 4.5 Hz), 122.1 (d, *J* = 4.5 Hz), 119.5 (d, *J* = 4.6 Hz), 107.8 (dd, *J* = 36.6, 3.7 Hz), 102.0 (d, *J* = 38.0 Hz), 80.4 (d, *J* = 208.3 Hz), 62.7 (d, *J* = 7.3 Hz), 41.1 (d, *J* = 113.5 Hz), 16.2 (d, *J* = 6.8 Hz); ^19^F NMR (376 MHz, CDCl_3_) δ −67.07; ^31^P­{^1^H} NMR (162 MHz, CDCl_3_) δ 15.78; *R_f_* = 0.46 (EtOAc); EI MS 303 (2%, M^+^), 302 (5%), 259 (50%), 211 (100%), 183 (50%), 156 (20%), 120 (45%), 93 (60%), 65 (40%); HRMS (ESI/QTOF) *m*/*z* [M + H]^+^ calcd for [C_16_H_16_FNO_2_P]^+^ 304.0897, found 304.0911 (100%).

#### Ethyl ((6-Methylpyridin-2-yl)­methyl)­(phenylethynyl)­phosphinate (**3e** *_ortho_*)

GP2_C_ was followed with 2-(bromomethyl)-6-methylpyridine (500 mg, 2.69 mmol) and phosphonite **6a** (597 mg, 2.69 mmol). The resulting product was purified by column chromatography (CHCl_3_/EtOAc/MeOH, gradient from 50:50:0 to 48:48:4), yielding 353 mg of phosphinate **3e**_*ortho*_ (1.18 mmol, 44%) as a yellow oil: ^1^H NMR (400 MHz, CDCl_3_) δ 7.54 (t, *J* = 7.7 Hz, 1H), 7.50–7.42 (m, 4H), 7.44–7.40 (m, 1H), 7.37–7.31 (m, 2H), 7.24 (dd, *J* = 7.9, 2.3 Hz 1H), 7.04 (dd, *J* = 7.6, 2.3 Hz, 1H), 4.31–4.15 (m, 2H), 3.58 (d, *J*_P–H_ = 20.5 Hz, 2H), 2.51 (s, 3H), 1.36 (t, *J* = 7.0 Hz, 3H); ^13^C­{^1^H} NMR (101 MHz, CDCl_3_) δ 158.3 (d, *J* = 2.6 Hz), 150.9 (d, *J* = 8.1 Hz), 136.7 (d, *J* = 3.1 Hz), 132.5 (d, *J* = 2.0 Hz), 130.6, 128.5, 121.64 (d, *J* = 2.0 Hz), 121.59, 119.7 (d, *J* = 4.4 Hz), 101.4 (d, *J* = 37.1 Hz), 80.9 (d, *J* = 205.3 Hz), 62.5 (d, *J* = 7.3 Hz), 41.8 (d, *J* = 113.3 Hz), 24.4, 16.2 (d, *J* = 6.8 Hz); ^31^P­{^1^H} NMR (162 MHz, CDCl_3_) δ 17.25; *R_f_* = 0.26 (EtOAc); EI MS 299 (1%, M^+^), 298 (5%), 207 (25%), 206 (100%); HRMS (ESI/QTOF) *m*/*z* [M + H]^+^ calcd for [C_17_H_19_NO_2_P]^+^ 300.1148, found 300.1154 (100%).

#### Ethyl ((6-Methoxypyridin-2-yl)­methyl)­(phenylethynyl)­phosphinate (**3f** *_ortho_*)

GP2_C_ was followed with 2-(bromomethyl)-6-methoxypyridine (399 mg, 1.98 mmol) and phosphonite **6a** (439 mg, 1.98 mmol). The resulting product was purified by column chromatography (CHCl_3_/EtOAc/MeOH, gradient from 50:50:0 to 48:48:4), yielding 396 mg of phosphinate **3f**_*ortho*_ (1.26 mmol, 64%) as an orange oil: ^1^H NMR (400 MHz, CDCl_3_) δ 7.52 (ddd, *J* = 8.3, 7.3, 0.9 Hz, 1H), 7.48–7.40 (m, 3H), 7.39–7.32 (m, 2H), 6.95 (dd, *J* = 7.3, 2.9 Hz, 1H), 6.63 (dd, *J* = 8.2, 2.5 Hz, 1H), 4.34–4.17 (m, 2H), 3.87 (s, 3H), 3.51 (d, *J* = 20.7 Hz, 2H), 1.37 (t, *J* = 7.0 Hz, 3H); ^13^C­{^1^H} NMR (101 MHz, CDCl_3_) δ 163.7 (d, *J* = 2.6 Hz), 149.2 (d, *J* = 8.7 Hz), 138.9 (d, *J* = 3.2 Hz), 132.4 (d, *J* = 1.9 Hz), 130.6, 128.5, 119.8 (d, *J* = 4.4 Hz), 117.3 (d, *J* = 6.2 Hz), 109.0 (d, *J* = 3.8 Hz), 101.2 (d, *J* = 37.0 Hz), 81.1 (d, *J* = 205.5 Hz), 62.3 (d, *J* = 7.3 Hz), 53.3, 41.3 (d, *J* = 114.1 Hz), 16.3 (d, *J* = 7.0 Hz); ^31^P­{^1^H} NMR (162 MHz, CDCl_3_) δ 17.54; *R_f_* = 0.50 (EtOAc); EI MS 315 (10%, M^+^), 222 (10%), 208 (40%), 165 (60%), 123 (20%), 102 (30%); HRMS (ESI/QTOF) *m*/*z* [M + H]^+^ calcd for [C_17_H_19_NO_3_P]^+^ 316.1097, found 316.1099 (100%).

#### Methyl 6-((Ethoxy­(phenylethynyl)­phosphoryl)­methyl)­picolinate (**3g** *_ortho_*)

GP2_C_ was followed with methyl 6-(bromomethyl)­picolinate (200 mg, 0.869 mmol) and phosphonite **6a** (193 mg, 0.869 mmol). The resulting product was purified by column chromatography (CHCl_3_/EtOAc/MeOH, gradient from 50:50:0 to 48:48:4), yielding 221 mg of phosphinate **3g**_*ortho*_ (0.644 mmol, 74%) as a yellow oil: ^1^H NMR (400 MHz, CDCl_3_) δ 8.04 (ddd, *J* = 7.7, 2.1, 1.1 Hz, 1H), 7.82 (td, *J* = 7.8, 0.7 Hz, 1H), 7.67 (ddd, *J* = 7.8, 2.4, 1.1 Hz, 1H), 7.50–7.40 (m, 3H), 7.37–7.30 (m, 2H), 4.33–4.15 (m, 2H), 3.94 (s, 3H), 3.74 (d, *J* = 20.7 Hz, 2H), 1.36 (t, *J* = 7.1 Hz, 3H); ^13^C­{^1^H} NMR (101 MHz, CDCl_3_) δ 165.6, 152.4 (d, *J* = 7.7 Hz), 147.9 (d, *J* = 2.4 Hz), 137.4 (d, *J* = 2.8 Hz), 132.6 (d, *J* = 2.2 Hz), 130.7, 128.5, 128.1 (d, *J* = 4.3 Hz), 123.7 (d, *J* = 3.1 Hz), 119.5 (d, *J* = 4.4 Hz), 102.0 (d, *J* = 37.8 Hz), 80.5 (d, *J* = 207.9 Hz), 62.7 (d, *J* = 7.3 Hz), 52.9, 41.7 (d, *J* = 113.1 Hz), 16.2 (d, *J* = 6.8 Hz); ^31^P­{^1^H} NMR (162 MHz, CDCl_3_) δ 16.18; *R_f_* = 0.30 (EtOAc); EI MS 343 (1%, M^+^), 328 (100%), 300 (90%), 165 (20%); HRMS (ESI/QTOF) *m*/*z* [M + H]^+^ calcd for [C_18_H_19_NO_4_P]^+^ 344.1046, found 344.1032 (100%).

#### Ethyl ((5-Fluoropyridin-2-yl)­methyl)­(phenylethynyl)­phosphinate (**3d** *_meta_*)

GP2_C_ was followed with 2-(bromomethyl)-5-fluoropyridine (747 mg, 3.93 mmol) and phosphonite **6a** (873 mg, 3.93 mmol). The resulting product was purified by column chromatography (CHCl_3_/EtOAc, gradient from 100:0 to 50:50), yielding 487 mg of phosphinate **3d**_*meta*_ (1.61 mmol, 41%) as a brown oil: ^1^H NMR (400 MHz, CDCl_3_) δ 8.42 (d, *J* = 2.9 Hz, 1H), 7.52–7.41 (m, 4H), 7.41–7.31 (m, 3H), 4.31–4.15 (m, 2H), 3.60 (d, *J* = 20.3 Hz, 2H), 1.36 (td, *J* = 7.1, 1.0 Hz, 3H); ^13^C­{^1^H} NMR (101 MHz, CDCl_3_) δ 158.8 (dd, *J* = 255.7, 3.8 Hz), 147.8 (dd, *J* = 8.4, 4.0 Hz), 137.8 (dd, *J* = 23.6, 2.6 Hz), 132.6 (d, *J* = 2.1 Hz), 130.8, 128.6, 125.6 (t, *J* = 4.5 Hz), 123.4 (dd, *J* = 18.6, 3.2 Hz), 119.6 (d, *J* = 4.4 Hz), 101.8 (d, *J* = 37.1 Hz), 80.7 (d, *J* = 205.7 Hz), 62.7 (d, *J* = 7.3 Hz), 41.0 (dd, *J* = 114.1, 1.5 Hz), 16.3 (d, *J* = 6.6 Hz); ^31^P­{^1^H} NMR (162 MHz, CDCl_3_) δ 16.35 (d, *J* = 5.9 Hz); ^19^F NMR (376 MHz, CDCl_3_) δ −129.46 (d, *J* = 6.1 Hz); *R_f_* = 0.64 (92:8 CHCl_3_/MeOH); EI MS 303 (9%, M^+^), 302 (21%), 259 (57%), 211 (82%), 174 (28%), 165 (100%), 141 (34%), 111 (51%), 102 (50%), 83 (26%); HRMS (ESI/QTOF) *m*/*z* [M + H]^+^ calcd for [C_16_H_16_FNO_2_P]^+^ 304.0897, found 304.0899 (100%).

#### Ethyl ((5-Methylpyridin-2-yl)­methyl)­(phenylethynyl)­phosphinate (**3e** *_meta_*)

GP2_C_ was followed with 2-(bromomethyl)-5-methylpyridine (755 mg, 4.06 mmol) and phosphonite **6a** (902 mg, 4.06 mmol). The resulting product was purified by column chromatography (CHCl_3_/EtOAc/MeOH, gradient from 50:50:0 to 48:48:4), yielding 310 mg of phosphinate **3e** *_meta_* (1.04 mmol, 26%) as a brown oil: ^1^H NMR (400 MHz, CDCl_3_) δ 8.39 (d, *J* = 1.3 Hz, 1H), 7.51–7.39 (m, 4H), 7.39–7.29 (m, 3H), 4.32–4.13 (m, 2H), 3.58 (d, *J* = 20.3 Hz, 2H), 2.31 (d, *J* = 2.4 Hz, 3H), 1.36 (t, *J* = 7.0 Hz, 3H); ^13^C­{^1^H} NMR (101 MHz, CDCl_3_) δ 150.1 (d, *J* = 2.6 Hz), 148.7 (d, *J* = 8.5 Hz), 137.2 (d, *J* = 3.2 Hz), 132.6 (d, *J* = 2.0 Hz, 2C), 131.7 (d, *J* = 3.7 Hz), 130.7, 128.6 (2C), 124.2 (d, *J* = 4.9 Hz), 119.8 (d, *J* = 4.4 Hz), 101.5 (d, *J* = 36.9 Hz), 81.0 (d, *J* = 204.2 Hz), 62.6 (d, *J* = 7.3 Hz), 41.40 (d, *J* = 113.7 Hz), 18.2, 16.4 (d, *J* = 6.7 Hz); ^31^P­{^1^H} NMR (162 MHz, CDCl_3_) δ 17.24; *R_f_* = 0.50 (92:8 CHCl_3_/MeOH); EI MS 299 (10%, M^+^), 298 (15%), 255 (47%), 236 (23%), 206 (100%), 191 (18%), 165 (43%), 137 (18%), 107 (72%), 77 (30%); HRMS (ESI/QTOF) *m*/*z* [M + H]^+^ calcd for [C_17_H_19_NO_2_P]^+^ 300.1148, found 300.1148 (100%).

#### Ethyl ((5-Methoxypyridin-2-yl)­methyl)­(phenylethynyl)­phosphinate (**3f** *_meta_*)

GP2_C_ was followed with 2-(bromomethyl)-5-methoxypyridine (290 mg, 1.437 mmol) and phosphonite **6a** (319 mg, 1.437 mmol). The resulting product was purified by column chromatography (CHCl_3_/EtOAc/MeOH, gradient from 50:50:0 to 48:48:4), yielding 171 mg of phosphinate **3f** *_meta_* (0.542 mmol, 38%) as a brown oil: ^1^H NMR (400 MHz, CDCl_3_) δ 8.22 (d, *J* = 3.0 Hz, 1H), 7.46–7.41 (m, 2H), 7.41–7.35 (m, 1H), 7.33–7.27 (m, 3H), 7.13 (ddd, *J* = 8.6, 3.0, 0.8 Hz, 1H), 4.28–4.06 (m, 2H), 3.79 (s, 3H), 3.52 (d, *J* = 20.0 Hz, 2H), 1.31 (t, *J* = 7.1 Hz, 3H); ^13^C­{^1^H} NMR (101 MHz, CDCl_3_) δ 154.7 (d, *J* = 3.2 Hz), 143.4 (d, *J* = 8.6 Hz), 137.1 (d, *J* = 2.5 Hz), 132.5 (d, *J* = 2.0 Hz, 2C), 130.6, 128.5 (2C), 124.9 (d, *J* = 4.8 Hz), 121.2 (d, *J* = 3.2 Hz), 119.7 (d, *J* = 4.4 Hz), 101.5 (d, *J* = 36.7 Hz), 80.9 (d, *J* = 204.0 Hz), 62.5 (d, *J* = 7.4 Hz), 55.6, 40.7 (d, *J* = 114.4 Hz), 16.27 (d, *J* = 6.6 Hz); ^31^P­{^1^H} NMR (162 MHz, CDCl_3_) δ 17.42; *R_f_* = 0.64 (92:8 CHCl_3_/MeOH); EI MS 315 (21%, M^+^), 271 (64%), 252 (31%), 224 (100%), 208 (58%), 186 (38%), 180 (68%), 165 (67%), 153 (22%), 123 (75%), 102 (48%), 80 (28%), 52 (25%); HRMS (ESI/QTOF) *m*/*z* [M + H]^+^ calcd for [C_17_H_19_NO_3_P]^+^ 316.1097, found 316.1096 (100%).

#### Methyl 6-((Ethoxy­(phenylethynyl)­phosphoryl)­methyl)­nicotinate (**3g** *_meta_*)

GP2_C_ was followed with methyl 6-(bromomethyl)­nicotinate (500 mg, 2.17 mmol) and phosphonite **6a** (500 mg, 2.17 mmol). The resulting product was purified by column chromatography (CHCl_3_/EtOAc, gradient from 100:0 to 50:50), yielding 284 mg of phosphinate **3g** *_meta_* (0.827 mmol, 38%) as a brown oil: ^1^H NMR (400 MHz, CDCl_3_) δ 9.21–9.11 (m, 1H), 8.26 (ddd, *J* = 8.1, 2.3, 0.7 Hz, 1H), 7.53 (ddd, *J* = 8.1, 2.5, 0.9 Hz, 1H), 7.51–7.41 (m, 3H), 7.39–7.31 (m, 2H), 4.35–4.16 (m, 2H), 3.95 (s, 3H), 3.69 (d, *J* = 21.0 Hz, 2H), 1.36 (t, *J* = 7.0 Hz, 3H); ^13^C­{^1^H} NMR (101 MHz, CDCl_3_) δ 165.6 (d, *J* = 1.6 Hz), 156.3 (d, *J* = 8.4 Hz), 150.7 (d, *J* = 2.9 Hz), 137.5 (d, *J* = 2.9 Hz), 132.5 (d, *J* = 2.0 Hz), 130.8, 128.6, 124.5 (d, *J* = 3.4 Hz), 124.3 (d, *J* = 4.7 Hz), 119.3 (d, *J* = 4.5 Hz), 101.9 (d, *J* = 37.8 Hz), 80.4 (d, *J* = 207.9 Hz), 62.7 (d, *J* = 7.2 Hz), 52.4, 42.1 (d, *J* = 112.6 Hz), 16.2 (d, *J* = 6.7 Hz); ^31^P­{^1^H} NMR (162 MHz, CDCl_3_) δ 15.38; *R_f_* = 0.65 (92:8 CHCl_3_/MeOH); EI MS 343 (15%, M^+^), 342 (25%), 299 (67%), 280 (28%), 252 (100%), 214 (43%), 191 (52%), 181 (30%), 165 (81%), 151 (68%), 120 (28%), 102 (43%); HRMS (ESI/QTOF) *m*/*z* [M + H]^+^ calcd for [C_18_H_19_NO_4_P]^+^ 344.1046, found 344.1047 (100%).

#### Ethyl ((4-Fluoropyridin-2-yl)­methyl)­(phenylethynyl)­phosphinate (**3d** *_para_*)

GP2_C_ was followed with 2-(bromomethyl)-4-fluoropyridine (306 mg, 1.61 mmol) and phosphonite **6a** (358 mg, 1.61 mmol). The resulting product was purified by column chromatography (CHCl_3_/EtOAc/MeOH, gradient from 50:50:0 to 48:48:4), yielding 220 mg of phosphinate **3d** *_para_* (0.725 mmol, 45%) as a brown oil: ^1^H NMR (400 MHz, CDCl_3_) δ 8.52 (dd, *J* = 8.7, 5.7 Hz, 1H), 7.50–7.39 (m, 3H), 7.38–7.31 (m, 2H), 7.19 (dt, *J* = 9.5, 2.5 Hz, 1H), 6.94 (ddt, *J* = 8.2, 5.7, 2.3 Hz, 1H), 4.34–4.15 (m, 2H), 3.61 (d, *J* = 20.6 Hz, 2H), 1.36 (t, *J* = 7.1 Hz, 3H); ^13^C­{^1^H} NMR (101 MHz, CDCl_3_) δ 168.8 (dd, *J* = 262.5, 3.4 Hz), 155.1 (t, *J* = 7.7 Hz), 151.9 (dd, *J* = 7.2, 2.7 Hz), 132.6 (d, *J* = 2.1 Hz, 2C), 130.9, 128.7 (2C), 119.6 (d, *J* = 4.6 Hz), 112.7 (dd, *J* = 17.5, 4.7 Hz), 110.4 (dd, *J* = 16.3, 3.2 Hz), 101.9 (d, *J* = 37.5 Hz), 80.6 (d, *J* = 207.3 Hz), 62.8 (d, *J* = 7.3 Hz), 41.9 (dd, *J* = 113.5, 3.2 Hz), 16.3 (d, *J* = 6.7 Hz); ^31^P­{^1^H} NMR (162 MHz, CDCl_3_) δ 15.69 (d, *J* = 3.2 Hz); ^19^F­{^1^H} NMR (376 MHz, CDCl_3_) δ −102.27; *R_f_* = 0.70 (92:8 CHCl_3_/MeOH); EI MS 303 (9%, M^+^), 302 (19%), 259 (36%), 240 (23%), 211 (100%), 174 (34%), 165 (83%), 141 (24%), 120 (36%), 111 (89%), 102 (59%), 83 (53%); HRMS (APCI/QTOF) *m*/*z* [M + H]^+^ calcd for [C_16_H_16_FNO_2_P]^+^ 304.0897, found 304.0900 (100%).

#### Ethyl ((4-Methylpyridin-2-yl)­methyl)­(phenylethynyl)­phosphinate (**3e** *_para_*)

GP2_C_ was followed with 2-(bromomethyl)-4-methylpyridine (452 mg, 2.43 mmol) and phosphonite **6a** (540 mg, 2.43 mmol). The resulting product was purified by column chromatography (CHCl_3_/EtOAc/MeOH, gradient from 50:50:0 to 48:48:4), yielding 196 mg of phosphinate **3e**_*para*_ (0.656 mmol, 27%) as a brown oil: ^1^H NMR (400 MHz, CDCl_3_) δ 8.41 (d, *J* = 5.1 Hz, 1H), 7.50–7.45 (m, 2H), 7.45–7.40 (m, 1H), 7.38–7.32 (m, 2H), 7.28–7.26 (m, 1H), 7.01 (dd, *J* = 4.9, 2.3 Hz, 1H), 4.33–4.17 (m, 2H), 3.57 (d, *J* = 20.5 Hz, 2H), 2.33 (d, *J* = 0.8 Hz, 3H), 1.36 (t, *J* = 7.0 Hz, 3H); ^13^C­{^1^H} NMR (101 MHz, CDCl_3_) δ 151.5 (d, *J* = 8.3 Hz), 149.3 (d, *J* = 2.6 Hz), 147.7 (d, *J* = 3.0 Hz), 132.6 (d, *J* = 2.0 Hz, 2C), 130.6, 128.5 (2C), 125.7 (d, *J* = 4.8 Hz), 123.2 (d, *J* = 3.4 Hz), 119.7 (d, *J* = 4.3 Hz), 101.5 (d, *J* = 37.0 Hz), 80.9 (d, *J* = 205.0 Hz), 62.6 (d, *J* = 7.4 Hz), 41.7 (d, *J* = 113.3 Hz), 21.0, 16.3 (d, *J* = 6.7 Hz); ^31^P­{^1^H} NMR (162 MHz, CDCl_3_) δ 17.18; *R_f_* = 0.55 (92:8 CHCl_3_/MeOH); EI MS 299 (6%, M^+^), 298 (11%), 255 (29%), 236 (23%), 206 (100%), 165 (23%), 107 (51%), 77 (20%); HRMS (ESI/QTOF) *m*/*z* [M + H]^+^ calcd for [C_17_H_19_NO_2_P]^+^ 300.1148, found 300.1152 (100%).

#### Ethyl ((4-Methoxypyridin-2-yl)­methyl)­(phenylethynyl)­phosphinate (**3f** *_para_*)

GP2_C_ was followed with 2-(bromomethyl)-4-methoxypyridine (1.40 g, 6.93 mmol) and phosphonite **6a** (1.54 g, 6.93 mmol). The resulting product was purified by column chromatography (CHCl_3_/EtOAc/MeOH, gradient from 50:50:0 to 48:48:4), yielding 250 mg of phosphinate **3f**_*para*_ (0.793 mmol, 11%) as a brown oil: ^1^H NMR (400 MHz, CDCl_3_) δ 8.39 (d, *J* = 5.8 Hz, 1H), 7.53–7.48 (m, 2H), 7.47–7.43 (m, 1H), 7.40–7.34 (m, 2H), 7.00 (t, *J* = 2.6 Hz, 1H), 6.75 (dt, *J* = 5.9, 2.3 Hz, 1H), 4.37–4.18 (m, 2H), 3.85 (s, 3H), 3.59 (d, *J* = 20.4 Hz, 2H), 1.40 (t, *J* = 7.1 Hz, 3H); ^13^C­{^1^H} NMR (101 MHz, CDCl_3_) δ 166.1 (d, *J* = 2.8 Hz), 153.3 (d, *J* = 8.0 Hz), 150.7 (d, *J* = 2.5 Hz), 132.6 (d, *J* = 2.1 Hz, 2C), 130.7, 128.6 (2C), 119.7 (d, *J* = 4.4 Hz), 110.5 (d, *J* = 4.8 Hz), 108.9 (d, *J* = 3.0 Hz), 101.6 (d, *J* = 37.3 Hz), 80.8 (d, *J* = 205.5 Hz), 62.6 (d, *J* = 7.3 Hz), 55.2, 41.9 (d, *J* = 113.3 Hz), 16.3 (d, *J* = 6.7 Hz); ^31^P­{^1^H} NMR (162 MHz, CDCl_3_) δ 16.96; *R_f_* = 0.50 (92:8 CHCl_3_/MeOH); EI MS 315 (11%, M^+^), 314 (17%), 271 (29%), 252 (44%), 224 (100%), 208 (66%), 180 (81%), 165 (31%), 123 (67%), 10 (36%); HRMS (ESI/QTOF) *m*/*z* [M + H]^+^ calcd for [C_17_H_19_NO_3_P]^+^ 316.1097, found 316.1100 (100%).

#### Methyl 6-((Ethoxy­(phenylethynyl)­phosphoryl)­methyl)­isonicotinate (**3g** *_para_*)

GP2_C_ was followed with methyl 6-(bromomethyl)­isonicotinate (413 mg, 1.795 mmol) and phosphonite **6a** (399 mg, 1.795 mmol). The resulting product was purified by column chromatography (CHCl_3_/EtOAc, gradient from 100:0 to 50:50), yielding 294 mg of phosphinate **3g**_*para*_ (0.856 mmol, 48%) as a brown oil: ^1^H NMR (400 MHz, CDCl_3_) δ 8.73 (d, *J* = 5.1 Hz, 1H), 8.05–7.94 (m, 1H), 7.75 (ddd, *J* = 5.1, 2.3, 1.6 Hz, 1H), 7.52–7.46 (m, 2H), 7.43 (d, *J* = 7.6 Hz, 1H), 7.39–7.31 (m, 2H), 4.34–4.17 (m, 2H), 3.92 (s, 3H), 3.69 (d, *J* = 20.8 Hz, 2H), 1.37 (t, *J* = 7.1 Hz, 3H); ^13^C­{^1^H} NMR (101 MHz, CDCl_3_) δ 165.5, 153.2 (d, *J* = 8.5 Hz), 150.5 (d, *J* = 2.9 Hz), 138.0 (d, *J* = 3.2 Hz), 132.7 (d, *J* = 2.0 Hz), 130.8, 128.6, 124.0 (d, *J* = 4.9 Hz), 121.4 (d, *J* = 3.5 Hz), 119.6 (d, *J* = 4.5 Hz), 102.1 (d, *J* = 37.5 Hz), 80.6 (d, *J* = 207.1 Hz), 62.8 (d, *J* = 7.4 Hz), 52.8, 41.9 (d, *J* = 113.1 Hz), 16.3 (d, *J* = 6.7 Hz); ^31^P­{^1^H} NMR (162 MHz, CDCl_3_) δ 16.06; *R_f_* = 0.70 (92:8 CHCl_3_/MeOH); EI MS 343 (13%, M^+^), 342 (20%), 299 (86%), 252 (100%), 214 (36%), 192 (48%), 181 (28%), 165 (86%), 151 (77%), 102 (54%), 64 (21%); HRMS (ESI/QTOF) *m*/*z* [M + H]^+^ calcd for [C_18_H_19_NO_4_P]^+^ 344.1046, found 344.1049 (100%).

#### Ethyl (Phenylethynyl)­(quinolin-2-ylmethyl)­phosphinate (**3h**)

GP2_C_ was followed with 2-(bromomethyl)­quinoline (750 mg, 3.38 mmol) and phosphonite **6a** (750 mg, 3.38 mmol). The resulting product was purified by column chromatography (CHCl_3_/EtOAc/MeOH, gradient from 50:50:0 to 48:48:4), yielding 330 mg of phosphinate **3h** (0.984 mmol, 29%) as a brown oil: ^1^H NMR (400 MHz, CDCl_3_) δ 8.14 (d, *J* = 8.5 Hz, 1H), 8.05 (dt, *J* = 8.4, 1.0 Hz, 1H), 7.82 (dd, *J* = 7.9, 1.2 Hz, 1H), 7.70 (ddd, *J* = 8.4, 6.9, 1.5 Hz, 1H), 7.58 (dd, *J* = 8.5, 1.6 Hz, 1H), 7.53 (ddt, *J* = 8.0, 6.9, 1.1 Hz, 2H), 7.42–7.37 (m, 3H), 7.35–7.26 (m, 3H), 4.34–4.16 (m, 2H), 3.82 (d, *J* = 20.7 Hz, 2H), 1.36 (t, *J* = 7.0 Hz, 3H); ^13^C­{^1^H} NMR (101 MHz, CDCl_3_) δ 152.4 (d, *J* = 8.1 Hz), 148.2 (d, *J* = 2.6 Hz), 136.6 (d, *J* = 2.4 Hz), 132.6 (d, *J* = 2.1 Hz), 130.7 (2C), 129.7, 129.1 (d, *J* = 1.2 Hz), 128.5 (2C), 127.7 (d, *J* = 1.7 Hz), 127.1 (d, *J* = 2.3 Hz), 126.5 (d, *J* = 1.7 Hz), 122.6 (d, *J* = 3.3 Hz), 119.6 (d, *J* = 4.5 Hz), 101.9 (d, *J* = 37.4 Hz), 80.9 (d, *J* = 206.6 Hz), 62.7 (d, *J* = 7.4 Hz), 42.9 (d, *J* = 112.1 Hz), 16.3 (d, *J* = 6.8 Hz); ^31^P­{^1^H} NMR (162 MHz, CDCl_3_) δ 16.58; *R_f_* = 0.71 (92:8 CHCl_3_/MeOH); EI MS 335 (<2%, M^+^), 243 (100%); HRMS (ESI/QTOF) *m*/*z* [M + H]^+^ calcd for [C_20_H_19_NO_2_P]^+^ 336.1148, found 336.1147 (100%).

#### Ethyl (Phenylethynyl)­(quinoxalin-2-ylmethyl)­phosphinate (**3i**)

GP2_C_ was followed with 2-(bromomethyl)­quinoxaline (240 mg, 1.07 mmol) and phosphonite **6a** (750 mg, 3.38 mmol). The resulting product was purified by column chromatography (CHCl_3_/EtOAc/MeOH, gradient from 50:50:0 to 48:48:4), yielding 185 mg of phosphinate **3i** (0.550 mmol, 51%) as a brown oil: ^1^H NMR (400 MHz, CDCl_3_) δ 8.96 (d, *J* = 1.4 Hz, 1H), 8.17–8.08 (m, 1H), 8.10–8.03 (m, 1H), 7.82–7.70 (m, 2H), 7.47–7.39 (m, 3H), 7.33 (dd, *J* = 8.1, 7.0 Hz, 2H), 4.45–4.17 (m, 2H), 3.85 (d, *J* = 21.0 Hz, 2H), 1.37 (t, *J* = 7.0 Hz, 3H); ^13^C­{^1^H} NMR (101 MHz, CDCl_3_) δ 147.9 (d, *J* = 8.7 Hz), 146.2 (d, *J* = 3.3 Hz), 142.5 (d, *J* = 2.9 Hz), 141.5 (d, *J* = 2.6 Hz), 132.7 (d, *J* = 2.1 Hz, 2C), 131.0, 130.4 (d, *J* = 1.4 Hz), 129.9 (d, *J* = 1.7 Hz), 129.4 (d, *J* = 1.8 Hz), 129.2 (d, *J* = 1.4 Hz), 128.7 (2C), 119.3 (d, *J* = 4.5 Hz), 102.7 (d, *J* = 38.1 Hz), 80.4 (d, *J* = 209.7 Hz), 63.0 (d, *J* = 7.3 Hz), 40.4 (d, *J* = 112.6 Hz), 16.3 (d, *J* = 6.8 Hz); ^31^P­{^1^H} NMR (162 MHz, CDCl_3_) δ 14.81; *R_f_* = 0.65 (93:7 CHCl_3_/MeOH); EI MS 336 (<3%, M^+^), 307 (7%), 244 (100%), 102 (9%); HRMS (ESI) *m*/*z* [M + H]^+^ calcd for [C_19_H_18_N_2_O_2_P]^+^ 337.1100, found 337.1102 (100%).

#### Ethyl (Isoquinolin-1-ylmethyl)­(phenylethynyl)­phosphinate (**3j**)

GP2_C_ was followed with 1-(bromomethyl)­isoquinoline (250 mg, 1.13 mmol) and phosphonite **6a** (250 mg, 1.13 mmol). The resulting product was purified by column chromatography (CHCl_3_/EtOAc/MeOH, gradient from 50:50:0 to 48:48:4), yielding 192 mg of phosphinate **3j** (0.573 mmol, 51%) as a brown oil: ^1^H NMR (400 MHz, CDCl_3_) δ 8.50 (dd, *J* = 5.6, 0.9 Hz, 1H), 8.32 (dd, *J* = 8.6, 1.3 Hz, 1H), 7.85–7.79 (m, 1H), 7.67 (ddd, *J* = 8.2, 6.9, 1.3 Hz, 1H), 7.64–7.56 (m, 2H), 7.42–7.37 (m, 1H), 7.34–7.28 (m, 4H), 4.30–4.11 (m, 4H), 1.31 (t, *J* = 7.1 Hz, 3H); ^13^C­{^1^H} NMR (101 MHz, CDCl_3_) δ 152.4 (d, *J* = 9.6 Hz), 142.2 (d, *J* = 3.9 Hz), 136.6 (d, *J* = 2.6 Hz), 132.6 (d, *J* = 2.1 Hz, 2C), 130.7, 130.3, 128.5 (2C), 128.0 (d, *J* = 4.0 Hz), 127.5, 127.3, 126.5 (d, *J* = 1.8 Hz), 120.5 (d, *J* = 3.9 Hz), 119.7 (d, *J* = 4.5 Hz), 101.9 (d, *J* = 37.4 Hz), 81.1 (d, *J* = 206.9 Hz), 62.8 (d, *J* = 7.4 Hz), 39.8 (d, *J* = 113.1 Hz), 16.3 (d, *J* = 6.8 Hz); ^31^P­{^1^H} NMR (162 MHz, CDCl_3_) δ 16.24; *R_f_* = 0.33 (95:5 CHCl_3_/MeOH); EI MS 335 (16%, M^+^), 291 (23%), 272 (31%), 243 (100%), 206 (36%), 165 (25%), 143 (70%), 115 (60%), 102 (29%); HRMS (ESI) *m*/*z* [M + H]^+^ calcd for [C_20_H_19_NO_2_P]^+^ 336.1148, found 336.1146 (100%).

#### Ethyl (Dibenzo­[*f*,*h*]­quinoxalin-2-ylmethyl)­(phenylethynyl)­phosphinate (**3k**)

GP2_C_ was followed with 2-(bromomethyl)­dibenzo­[*f*,*h*]­quinoxaline (170 mg, 0.526 mmol) and phosphonite **6a** (117 mg, 0.526 mmol). The resulting product was purified by column chromatography (CHCl_3_/EtOAc/MeOH, gradient from 50:50:0 to 48:48:4), yielding 150 mg of phosphinate **3k** (0.344 mmol, 65%) as a brown oil: ^1^H NMR (400 MHz, CDCl_3_) δ 9.24 (dd, *J* = 8.1, 1.4 Hz, 1H), 9.21 (dd, *J* = 7.9, 1.7 Hz, 1H), 8.96 (d, *J* = 2.0 Hz, 1H), 8.68–8.55 (m, 2H), 7.84–7.69 (m, 3H), 7.60 (ddd, *J* = 8.2, 7.0, 1.1 Hz, 1H), 7.45–7.33 (m, 3H), 7.30–7.18 (m, 2H), 4.51–4.15 (m, 2H), 3.92 (d, *J* = 20.9 Hz, 2H), 1.38 (t, *J* = 7.0 Hz, 3H); ^13^C­{^1^H} NMR (101 MHz, CDCl_3_) δ 146.7 (d, *J* = 9.0 Hz), 144.5 (d, *J* = 4.8 Hz), 140.8 (d, *J* = 2.8 Hz), 139.8 (d, *J* = 3.3 Hz), 132.8 (d, *J* = 2.1 Hz, 2C), 131.7, 131.4, 130.8, 129.9 (d, *J* = 1.6 Hz), 129.8, 129.7, 129.6, 128.6 (2C), 127.9, 127.6, 125.8, 125.5, 122.9, 122.8, 119.5 (d, *J* = 4.4 Hz), 102.6 (d, *J* = 37.9 Hz), 80.6 (d, *J* = 209.2 Hz), 62.9 (d, *J* = 7.3 Hz), 39.9 (d, *J* = 113.7 Hz), 16.4 (d, *J* = 6.9 Hz); ^31^P­{^1^H} NMR (162 MHz, CDCl_3_) δ 15.93; *R_f_* = 0.53 (50:50 CHCl_3_/EtOAc); EI MS 436 (48%, M^+^), 435 (92%), 407 (100%), 389 (18%), 343 (21%), 244 (26%); HRMS (ESI/QTOF) *m*/*z* [M + H]^+^ calcd for [C_27_H_22_N_2_O_2_P]^+^ 437.1413, found 437.1411 (100%).

#### Diethyl (Pyrazine-2,3-diylbis­(methylene))­bis­((phenylethynyl)­phosphinate) (**3l**)

GP2_C_ was followed with 2,3-bis­(bromomethyl)­pyrazine (120 mg, 0.451 mmol) and phosphonite **6a** (201 mg, 0.902 mmol). The resulting product was purified by column chromatography (CHCl_3_/EtOAc/MeOH, gradient from 50:50:0 to 48:48:4), yielding 95 mg of phosphinate **3l** (0.193 mmol, 43%) as a brown oil, obtained as a 1:1 mixture of two diastereomers: ^1^H NMR (400 MHz, CDCl_3_) δ 8.45 (s, 2H), 7.53–7.41 (m, 6H), 7.40–7.30 (m, 4H), 4.35–4.16 (m, 4H), 4.16–3.88 (m, 4H), 1.37 (td, *J* = 7.1, 4.7 Hz, 6H); ^13^C­{^1^H} NMR (101 MHz, CDCl_3_) δ 148.4 (2C), 142.8 (2C), 132.7 (4C), 130.9 (d, *J* = 1.8 Hz, 2C), 128.7 (4C), 119.5 (2C), 102.2 (d, *J* = 44.5 Hz, 2C), 80.7 (d, *J* = 207.3 Hz, 2C), 62.9 (2C), 39.0 (d, *J* = 112.8 Hz, 2C), 16.4 (2C) (signals of some carbons were split due to the presence of two diastereomers); ^31^P­{^1^H} NMR (162 MHz, CDCl_3_) δ 15.65, 15.50; *R_f_* = 0.68 (93:7 CHCl_3_/MeOH); HRMS (ESI/QTOF) *m*/*z* [M + H]^+^ calcd for [C_26_H_27_N_2_O_4_P_2_]^+^ 493.1441, found 493.1436 (100%).

#### Diethyl (Quinoxaline-2,3-diylbis­(methylene))­bis­((phenylethynyl)­phosphinate) (**3m**)

GP2_C_ was followed with 2,3-bis­(bromomethyl)­quinoxaline (250 mg, 0.791 mmol) and phosphonite **6a** (352 mg, 1.58 mmol). The resulting product was purified by column chromatography (CHCl_3_/EtOAc/MeOH, gradient from 50:50:0 to 48:48:4), yielding 290 mg of phosphinate **3m** (0.535 mmol, 68%) as a brown oil, identified as a 1:1 mixture of two diastereomers. Upon standing, the mixture solidified. The resulting solid was treated with diethyl ether and filtered. ^31^P NMR analysis indicated that this procedure afforded one diastereomer in approximately 90% purity: ^1^H NMR (400 MHz, CDCl_3_) δ 8.03 (dd, *J* = 6.3, 3.5 Hz, 2H), 7.74–7.68 (m, 2H), 7.48–7.38 (m, 6H), 7.33 (ddt, *J* = 8.7, 6.9, 1.0 Hz, 4H), 4.36–4.06 (m, 8H), 1.36 (t, *J* = 7.0 Hz, 6H); ^13^C­{^1^H} NMR (101 MHz, CDCl_3_) δ 149.2–147.1 (m, 2C), 141.4 (2C), 132.7 (4C), 130.9 (2C), 130.0 (2C), 128.9 (2C), 128.6 (4C), 120.2–118.2 (m, 2C), 102.4 (d, *J* = 38.5 Hz, 2C), 80.7 (d, *J* = 208.7 Hz, 2C), 64.5–61.7 (m, 2C), 40.0 (d, *J* = 111.2 Hz, 2C), 17.9–14.6 (m, 2C); ^31^P­{^1^H} NMR (162 MHz, CDCl_3_) δ 15.93, 15.66; *R_f_* = 0.53 (93:7 CHCl_3_/MeOH); HRMS (APCI/QTOF) *m*/*z* [M + H]^+^ calcd for [C_30_H_29_N_2_O_4_P_2_]^+^ 543.1597, found 543.1603 (100%).

#### 8-Ethoxy-6-phenylpyrazino­[1,2-*a*]­[1,4]­azaphosphinine 8-Oxide (**4a**)

GP3_A_ was followed with **3a** (50.0 mg, 0.175 mmol), CF_3_COOAg (1.93 mg, 8.73 μmol), and 3.5 mL of DCM. The resulting product was purified by column chromatography (CHCl_3_/MeOH, gradient from 100:0 to 96:4), yielding 45.0 mg of 1,4-azaphosphinine **4a** (0.157 mmol, 90%) as a yellow amorphous solid: ^1^H NMR (400 MHz, CDCl_3_) δ 8.20 (s, 1H), 7.69–7.47 (m, 3H), 7.42–7.30 (m, 2H), 6.81 (d, *J* = 5.4 Hz, 1H), 6.77 (d, *J* = 4.5 Hz, 0H), 5.95–5.79 (m, 1H), 5.61 (ddd, *J* = 4.2, 1.7, 0.8 Hz, 1H), 4.16–3.98 (m, 3H), 1.35 (td, *J* = 7.1, 0.7 Hz, 3H); ^13^C­{^1^H} NMR (101 MHz, CDCl_3_) δ 154.5 (d, *J* = 16.7 Hz), 150.1 (d, *J* = 1.6 Hz), 141.5 (d, *J* = 3.4 Hz), 134.8 (d, *J* = 11.9 Hz), 130.3, 129.5 (2C), 128.7 (2C), 123.0, 121.0, 106.6 (d, *J* = 126.8 Hz), 92.6 (d, *J* = 141.0 Hz), 62.1 (d, *J* = 6.0 Hz), 17.0 (d, *J* = 6.1 Hz); ^31^P­{^1^H} NMR (162 MHz, CDCl_3_) δ 16.15; *R_f_* = 0.49 (93:7 CHCl_3_/MeOH); EI MS 286 (5%, M^+^), 194 (100%); HRMS (ESI/QTOF) *m*/*z* [M + H]^+^ calcd for [C_15_H_16_N_2_O_2_P]^+^ 287.0944, found 287.0945 (100%).

#### 8-Ethoxy-1-methyl-6-phenylpyrazino­[1,2-*a*]­[1,4]­azaphosphinine 8-Oxide (**4b**)

GP3_A_ was followed with **3b** (85.0 mg, 0.0283 mmol), CF_3_COOAg (3.13 mg, 14.0 μmol), and 5.6 mL of DCM. The resulting product was purified by column chromatography (CHCl_3_/MeOH, gradient from 100:0 to 96:4), yielding 73.0 mg of 1,4-azaphosphinine **4b** (0.243 mmol, 86%) as a yellow amorphous solid: ^1^H NMR (400 MHz, CDCl_3_) δ 7.49 (dt, *J* = 5.4, 2.9 Hz, 3H), 7.40–7.32 (m, 2H), 6.81–6.73 (m, 2H), 5.84 (dd, *J* = 4.0, 1.0 Hz, 1H), 5.75–5.67 (m, 1H), 4.08 (dq, *J* = 9.2, 7.1 Hz, 2H), 2.52 (s, 3H), 1.36 (t, *J* = 7.1 Hz, 3H); ^13^C­{^1^H} NMR (101 MHz, CDCl_3_) ^13^C NMR δ 158.9 (d, *J* = 13.6 Hz), 150.9 (d, *J* = 1.9 Hz), 141.4 (d, *J* = 3.0 Hz), 135.8 (d, *J* = 12.2 Hz), 130.2, 129.5 (2C), 128.8 (2C), 121.6, 120.5, 106.5 (d, *J* = 126.1 Hz), 89.9 (d, *J* = 139.7 Hz), 62.1 (d, *J* = 6.1 Hz), 23.7, 17.1 (d, *J* = 6.0 Hz); ^31^P­{^1^H} NMR (162 MHz, CDCl_3_) δ 16.65; *R_f_* = 0.59 (93:7 CHCl_3_/MeOH); EI MS 300 (3%, M^+^), 208 (100%); HRMS (ESI/QTOF) *m*/*z* [M + H]^+^ calcd for [C_16_H_18_N_2_O_2_P]^+^ 301.1100, found 301.1105 (100%).

#### 8-Ethoxy-6-phenylpyrimido­[1,2-*a*]­[1,4]­azaphosphinine 8-Oxide (**4c**)

GP3_A_ was followed with **3c** (200 mg, 0.699 mmol), CF_3_COOAg (3.09 mg, 14.0 μmol), and 4.6 mL of DCM. The resulting product was purified by column chromatography (CHCl_3_/MeOH, gradient from 100:0 to 96:4), yielding 189 mg of 1,4-azaphosphinine **4c** (0.660 mmol, 95%) as an orange solid: ^1^H NMR (400 MHz, CDCl_3_) δ 8.08–7.98 (m, 1H), 7.49 (dd, *J* = 4.9, 1.9 Hz, 3H), 7.39–7.33 (m, 2H), 7.31 (ddd, *J* = 7.5, 2.0, 0.8 Hz, 1H), 5.87 (dd, *J* = 7.5, 3.4 Hz, 1H), 5.83 (dd, *J* = 3.9, 1.0 Hz, 1H), 5.65 (d, *J* = 3.9 Hz, 1H), 4.05 (dq, *J* = 9.2, 7.0 Hz, 2H), 1.33 (t, *J* = 7.1 Hz, 3H); ^13^C­{^1^H} NMR (101 MHz, CDCl_3_) δ 155.4 (d, *J* = 4.4 Hz), 152.0 (d, *J* = 12.1 Hz), 149.2 (d, *J* = 3.0 Hz), 138.9, 135.2 (d, *J* = 13.1 Hz), 130.3, 129.6 (2C), 128.7 (2C), 107.4 (d, *J* = 124.1 Hz), 103.7, 89.3 (d, *J* = 143.9 Hz), 61.9 (d, *J* = 6.2 Hz), 16.9 (d, *J* = 6.3 Hz); ^31^P­{^1^H} NMR (162 MHz, CDCl_3_) δ 19.39; *R_f_* = 0.36 (92:8 CHCl_3_/MeOH); EI MS 286 (<2%, M^+^), 194 (100%); HRMS (ESI/QTOF) *m*/*z* [M + H]^+^ calcd for [C_15_H_16_N_2_O_2_P]^+^ 287.0944, found 287.0947 (100%); mp 132.6–140.2 °C (EtOAc).

#### 2-Ethoxy-7-fluoro-4-phenylpyrido­[1,2-*a*]­[1,4]­azaphosphinine 2-Oxide (**4d** *_meta_*)

GP3_A_ was followed with **3d**_*meta*_ (230 mg, 0.758 mmol), CF_3_COOAg (8.4 mg, 38 μmol), and 5 mL of DCM. The reaction mixture was stirred at rt for 3 days. The resulting product was purified by column chromatography (CHCl_3_/MeOH, gradient from 100:0 to 96:4), yielding 210 mg of 1,4-azaphosphinine **4d**_*meta*_ (0.692 mmol, 91%) as a yellow amorphous solid: ^1^H NMR (400 MHz, CDCl_3_) 7.57–7.43 (m, 3H), 7.42–7.29 (m, 2H), 7.03–6.93 (m, 1H), 6.76 (dd, *J* = 10.1, 6.1 Hz, 1H), 6.68–6.55 (m, 1H), 5.76 (dd, *J* = 4.2, 1.7 Hz, 1H), 5.32 (dd, *J* = 4.4, 2.3 Hz, 1H), 4.01 (dq, *J* = 9.2, 7.1 Hz, 2H), 1.32 (t, *J* = 7.1 Hz, 3H); ^13^C­{^1^H} NMR (101 MHz, CDCl_3_) δ 150.8, 148.6 (d, *J* = 236.0 Hz), 146.7 (d, *J* = 4.3 Hz), 136.3 (d, *J* = 12.2 Hz), 130.0, 129.6 (2C), 128.6 (dd, *J* = 16.3, 7.3 Hz, 2C), 121.4 (d, *J* = 27.6 Hz), 121.4 (d, *J* = 27.5 Hz), 116.4 (d, *J* = 42.7 Hz), 105.5 (d, *J* = 125.4 Hz), 88.8 (d, *J* = 145.0 Hz), 61.9 (d, *J* = 6.1 Hz), 17.0 (d, *J* = 6.3 Hz); ^31^P­{^1^H} NMR (162 MHz, CDCl_3_) δ 17.30; ^19^F NMR (376 MHz, CDCl_3_) δ −144.87; *R_f_* = 0.54 (92:8 CHCl_3_/MeOH); EI MS 303 (3%, M^+^), 211 (100%); HRMS (ESI/QTOF) *m*/*z* [M + H]^+^ calcd for [C_16_H_16_FNO_2_P]^+^ 304.0897, found 304.0896 (100%).

#### 2-Ethoxy-7-methyl-4-phenylpyrido­[1,2-*a*]­[1,4]­azaphosphinine 2-Oxide (**4e** *_meta_*)

GP3_A_ was followed with **3e**_*meta*_ (130 mg, 0.434 mmol), CF_3_COOAg (4.8 mg, 22 μmol), and 2.9 mL of DCM. The resulting product was purified by column chromatography (CHCl_3_/MeOH, gradient from 100:0 to 96:4), yielding 118 mg of 1,4-azaphosphinine **4e**_*meta*_ (0.394 mmol, 91%) as a yellow amorphous solid: ^1^H NMR (400 MHz, CDCl_3_) δ 7.55–7.41 (m, 3H), 7.41–7.29 (m, 2H), 6.79 (s, 1H), 6.71 (d, *J* = 9.3 Hz, 1H), 6.49 (dt, *J* = 9.3, 2.0 Hz, 1H), 5.68 (dd, *J* = 4.2, 1.7 Hz, 1H), 5.19 (dd, *J* = 4.3, 1.9 Hz, 1H), 4.03–3.87 (m, 2H), 1.82 (s, 3H), 1.31 (t, *J* = 7.1 Hz, 3H); ^13^C­{^1^H} NMR (101 MHz, CDCl_3_) δ 151.0 (d, *J* = 2.0 Hz), 147.9 (d, *J* = 4.1 Hz), 137.0 (d, *J* = 12.2 Hz), 130.4 (d, *J* = 3.0 Hz), 129.6, 129.3 (2C), 128.6 (2C), 127.5, 126.5 (d, *J* = 16.2 Hz), 116.3, 105.1 (d, *J* = 124.5 Hz), 86.1 (d, *J* = 146.0 Hz), 61.6 (d, *J* = 6.1 Hz), 17.8, 16.9 (d, *J* = 6.5 Hz); ^31^P­{^1^H} NMR (162 MHz, CDCl_3_) δ 18.46; *R_f_* = 0.50 (92:8 CHCl_3_/MeOH); EI MS 299 (3%, M^+^), 207 (100%); HRMS (ESI/QTOF) *m*/*z* [M + H]^+^ calcd for [C_17_H_19_NO_2_P]^+^ 300.1148, found 300.1149 (100%).

#### 2-Ethoxy-7-methoxy-4-phenylpyrido­[1,2-*a*]­[1,4]­azaphosphinine 2-Oxide (**4f** *_meta_*)

GP3_A_ was followed with **3f**_*meta*_ (80.0 mg, 0.254 mmol), CF_3_COOAg (2.8 mg, 13 μmol), and 1.7 mL of DCM. The reaction mixture was stirred at rt for 5 days. The resulting product was purified by column chromatography (CHCl_3_/MeOH, gradient from 100:0 to 96:4), yielding 71.0 mg of 1,4-azaphosphinine **4f**_*meta*_ (0.225 mmol, 89%) as a yellow amorphous solid: ^1^H NMR (400 MHz, CDCl_3_) δ 7.54–7.44 (m, 3H), 7.43–7.31 (m, 2H), 6.72 (d, *J* = 9.9 Hz, 1H), 6.53 (dtd, *J* = 9.8, 2.3, 0.6 Hz, 1H), 6.50–6.43 (m, 1H), 5.69 (dd, *J* = 4.2, 1.7 Hz, 1H), 5.23 (dd, *J* = 4.5, 1.9 Hz, 1H), 3.98 (dq, *J* = 9.2, 7.0 Hz, 2H), 3.31 (s, 3H), 1.31 (t, *J* = 7.0 Hz, 3H); ^13^C­{^1^H} NMR (101 MHz, CDCl_3_) δ 151.0 (d, *J* = 2.1 Hz), 146.5 (d, *J* = 4.1 Hz), 143.9, 136.9 (d, *J* = 12.3 Hz), 129.7, 129.2 (2C), 128.5 (2C), 127.8 (d, *J* = 16.2 Hz), 124.3 (d, *J* = 2.9 Hz), 111.1, 104.2 (d, *J* = 125.3 Hz), 87.0 (d, *J* = 145.3 Hz), 61.6 (d, *J* = 6.2 Hz), 55.3, 16.9 (d, *J* = 6.3 Hz); ^31^P­{^1^H} NMR (162 MHz, CDCl_3_) δ 18.65; *R_f_* = 0.58 (92:8 CHCl_3_/MeOH); EI MS 315 (7%, M^+^), 223 (100%), 208 (22%), 180 (18%); HRMS (ESI/QTOF) *m*/*z* [M + H]^+^ calcd for [C_17_H_19_NO_3_P]^+^ 316.1097, found 316.1095 (100%).

#### Methyl 2-Ethoxy-4-phenylpyrido­[1,2-*a*]­[1,4]­azaphosphinine-7-carboxylate 2-Oxide (**4g** *_meta_*)

GP3_A_ was followed with **3g**_*meta*_ (210 mg, 0.612 mmol), CF_3_COOAg (6.7 mg, 31 μmol), and 4 mL of DCM. The resulting product was purified by column chromatography (CHCl_3_/MeOH, gradient from 100:0 to 96:4), yielding 198 mg of 1,4-azaphosphinine **4g**_*meta*_ (0.577 mmol, 94%) as a yellow solid: ^1^H NMR (400 MHz, CDCl_3_) δ 7.98–7.90 (m, 1H), 7.55–7.48 (m, 3H), 7.40–7.33 (m, 2H), 7.10–7.03 (m, 1H), 6.72 (d, *J* = 9.6 Hz, 1H), 5.88 (dd, *J* = 4.1, 1.7 Hz, 1H), 5.36 (dd, *J* = 4.4, 2.0 Hz, 1H), 4.05 (dq, *J* = 9.1, 7.0 Hz, 2H), 3.71 (s, 3H), 1.34 (t, *J* = 7.1 Hz, 3H); ^13^C­{^1^H} NMR (101 MHz, CDCl_3_) δ 164.6, 151.4 (d, *J* = 1.7 Hz), 148.0 (d, *J* = 3.4 Hz), 137.2, 135.9 (d, *J* = 12.0 Hz), 130.3, 129.6 (2C), 128.7 (2C), 126.2 (d, *J* = 16.1 Hz), 125.3 (d, *J* = 2.8 Hz), 111.5, 108.0 (d, *J* = 124.2 Hz), 90.1 (d, *J* = 144.5 Hz), 61.9 (d, *J* = 6.2 Hz), 52.3, 17.0 (d, *J* = 6.3 Hz); ^31^P­{^1^H} NMR (162 MHz, CDCl_3_) δ 15.23; *R_f_* = 0.56 (92:8 CHCl_3_/MeOH); EI MS 343 (2%, M^+^), 251 (100%), 191 (21%); HRMS (ESI/QTOF) *m*/*z* [M + H]^+^ calcd for [C_18_H_19_NO_4_P]^+^ 344.1046, found 344.1044 (100%); mp 167.6–169.3 °C (EtOAc).

#### 2-Ethoxy-8-fluoro-4-phenylpyrido­[1,2-*a*]­[1,4]­azaphosphinine 2-Oxide (**4d** *_para_*)

GP3_A_ was followed with **3d**_*para*_ (100 mg, 0.33 mmol), CF_3_COOAg (3.6 mg, 16 μmol), and 6.5 mL of DCM. The resulting product was purified by column chromatography (CHCl_3_/MeOH, gradient from 100:0 to 96:4), yielding 88.0 mg of 1,4-azaphosphinine **4d**_*para*_ (0.290 mmol, 88%) as a yellow amorphous solid: ^1^H NMR (400 MHz, CDCl_3_) δ 7.55–7.44 (m, 3H), 7.43–7.31 (m, 2H), 7.09 (dd, *J* = 8.3, 5.8 Hz, 1H), 6.35 (dd, *J* = 9.8, 2.9 Hz, 1H), 5.85–5.68 (m, 2H), 5.23–5.10 (m, 1H), 4.02 (dq, *J* = 9.1, 7.0 Hz, 2H), 1.33 (t, *J* = 7.1 Hz, 3H); ^13^C­{^1^H} NMR (101 MHz, CDCl_3_) δ 160.0 (d, *J* = 266.0 Hz), 150.8, 149.6 (dd, *J* = 11.5, 4.3 Hz), 136.6 (d, *J* = 12.1 Hz), 134.9 (d, *J* = 10.8 Hz), 130.1, 129.6 (2C), 128.6 (2C), 107.0 (dd, *J* = 21.7, 16.9 Hz), 106.7 (d, *J* = 124.1 Hz), 101.5 (d, *J* = 30.5 Hz), 86.9 (dd, *J* = 147.5, 6.7 Hz), 61.9 (d, *J* = 6.0 Hz), 17.0 (d, *J* = 6.2 Hz); ^31^P­{^1^H} NMR (162 MHz, CDCl_3_) δ 16.01; ^19^F NMR (376 MHz, CDCl_3_) δ −110.91; *R_f_* = 0.63 (92:8 CHCl_3_/MeOH); EI MS 303 (<3%, M^+^), 211 (100%); HRMS (ESI/QTOF) *m*/*z* [M + H]^+^ calcd for [C_16_H_16_FNO_2_P]^+^ 304.0897, found 304.0894 (100%).

#### 2-Ethoxy-8-methyl-4-phenylpyrido­[1,2-*a*]­[1,4]­azaphosphinine 2-Oxide (**4e** *_para_*)

GP3_A_ was followed with **3e**_*para*_ (100 mg, 0.334 mmol), CF_3_COOAg (3.7 mg, 3.2 μmol), and 2.2 mL of DCM. The resulting product was purified by column chromatography (CHCl_3_/MeOH, gradient from 100:0 to 96:4), yielding 95.0 mg of 1,4-azaphosphinine **4e**_*para*_ (0.317 mmol, 95%) as a yellow amorphous solid: ^1^H NMR (400 MHz, CDCl_3_) δ 7.52–7.43 (m, 3H), 7.34 (d, *J* = 5.7 Hz, 2H), 6.95 (d, *J* = 7.7 Hz, 1H), 6.55–6.47 (m, 1H), 5.71 (dd, *J* = 4.2, 1.6 Hz, 1H), 5.67 (dd, *J* = 7.7, 2.1 Hz, 1H), 5.10 (dd, *J* = 4.5, 1.9 Hz, 1H), 4.05–3.90 (m, 2H), 2.05 (s, 3H), 1.32 (t, *J* = 7.1 Hz, 3H); ^13^C­{^1^H} NMR (101 MHz, CDCl_3_) δ 151.1 (d, *J* = 2.0 Hz), 149.2 (d, *J* = 4.3 Hz), 137.5 (d, *J* = 3.2 Hz), 136.7 (d, *J* = 12.2 Hz), 130.5, 129.7, 129.3 (2C), 128.6 (2C), 123.8 (d, *J* = 16.2 Hz), 110.5, 105.3 (d, *J* = 123.9 Hz), 84.4 (d, *J* = 147.1 Hz), 61.6 (d, *J* = 6.1 Hz), 20.5, 16.9 (d, *J* = 6.5 Hz); ^31^P­{^1^H} NMR (162 MHz, CDCl_3_) δ 18.27; *R_f_* = 0.52 (92:8 CHCl_3_/MeOH); EI MS 299 (3%, M^+^), 207 (100%); HRMS (ESI/QTOF) *m*/*z* [M + H]^+^ calcd for [C_17_H_19_NO_2_P]^+^ 300.1148, found 300.1148 (100%).

#### 2-Ethoxy-8-methoxy-4-phenylpyrido­[1,2-*a*]­[1,4]­azaphosphinine 2-Oxide (**4f** *_para_*)

GP3_A_ was followed with **3f**_*para*_ (70 mg, 0.22 mmol), CF_3_COOAg (2.5 mg, 11 μmol), and 1.5 mL of DCM. The resulting product was purified by column chromatography (CHCl_3_/MeOH, gradient from 100:0 to 96:4), yielding 63 mg of 1,4-azaphosphinine **4f**_*para*_ (0.20 mmol, 90%) as a yellow oil: ^1^H NMR (400 MHz, CDCl_3_) δ 7.51–7.40 (m, 3H), 7.39–7.29 (m, 2H), 6.97 (d, *J* = 8.2 Hz, 1H), 5.93 (dd, *J* = 2.9, 1.3 Hz, 1H), 5.67 (dd, *J* = 4.1, 1.6 Hz, 1H), 5.63 (dd, *J* = 8.1, 2.8 Hz, 1H), 5.03 (dd, *J* = 4.2, 2.4 Hz, 1H), 3.96 (dqd, *J* = 9.2, 7.0, 0.9 Hz, 2H), 3.75 (s, 3H), 1.31 (t, *J* = 7.0 Hz, 3H); ^13^C­{^1^H} NMR (101 MHz, CDCl_3_) δ 157.4 (d, *J* = 4.7 Hz), 150.8 (d, *J* = 4.8 Hz), 136.9 (d, *J* = 12.2 Hz), 132.8, 129.7, 129.4 (2C), 128.7 (2C), 105.6 (d, *J* = 123.5 Hz), 104.3, 99.8 (d, *J* = 17.2 Hz), 83.2 (d, *J* = 149.2 Hz), 61.6 (d, *J* = 6.1 Hz), 55.5, 17.0 (d, *J* = 6.4 Hz); ^31^P­{^1^H} NMR (162 MHz, CDCl_3_) δ 18.00; *R_f_* = 0.50 (92:8 CHCl_3_/MeOH); EI MS 315 (2%, M^+^), 223 (100%), 208 (33%), 180 (20%); HRMS (ESI/QTOF) *m*/*z* [M + H]^+^ calcd for [C_17_H_19_NO_3_P]^+^ 316.1097, found 316.1102 (100%).

#### Methyl 2-Ethoxy-4-phenylpyrido­[1,2-*a*]­[1,4]­azaphosphinine-8-carboxylate 2-Oxide (**4g** *_para_*)

GP3_A_ was followed with **3g**_*para*_ (190 mg, 0.553 mmol), CF_3_COOAg (6.1 mg, 28 μmol), and 3.7 mL of DCM. The resulting product was purified by column chromatography (CHCl_3_/MeOH, gradient from 100:0 to 96:4), yielding 172 mg of 1,4-azaphosphinine **4g**_*para*_ (0.501 mmol, 91%) as a yellow amorphous solid: ^1^H NMR (400 MHz, CDCl_3_) δ 7.54–7.44 (m, 4H), 7.40–7.30 (m, 2H), 7.06 (d, *J* = 7.8 Hz, 1H), 6.26 (dd, *J* = 7.9, 2.0 Hz, 1H), 5.79 (dd, *J* = 4.2, 1.5 Hz, 1H), 5.50 (dd, *J* = 4.3, 1.9 Hz, 1H), 4.04 (dq, *J* = 9.2, 7.0 Hz, 2H), 3.88 (s, 3H), 1.34 (t, *J* = 7.0 Hz, 3H); ^13^C­{^1^H} NMR (101 MHz, CDCl_3_) δ 164.3 (d, *J* = 1.7 Hz), 151.2 (d, *J* = 2.1 Hz), 147.1 (d, *J* = 4.0 Hz), 136.2 (d, *J* = 12.2 Hz), 131.5, 130.8 (d, *J* = 16.6 Hz), 129.9, 129.4 (2C), 128.48, 128.46 (2C), 105.9 (d, *J* = 125.5 Hz), 105.2, 91.9 (d, *J* = 142.7 Hz), 61.8 (d, *J* = 6.1 Hz), 52.7, 16.8 (d, *J* = 6.3 Hz); ^31^P­{^1^H} NMR (162 MHz, CDCl_3_) δ 16.13; *R_f_* = 0.65 (92:8 CHCl_3_/MeOH); EI MS 343 (<2%, M^+^), 251 (100%), 192 (18%); HRMS (ESI/QTOF) *m*/*z* [M + H]^+^ calcd for [C_18_H_19_NO_4_P]^+^ 344.1046, found 344.1048 (100%).

#### 3-Ethoxy-1-phenyl-[1,4]­azaphosphinino­[1,2-*a*]­quinoline 3-Oxide (**4h**)

GP3_A_ was followed with **3h** (50.0 mg, 0.149 mmol), CF_3_COOAg (1.65 mg, 7.46 μmol), and 2.9 mL of DCM. The reaction mixture was stirred at room temperature for 13 days to achieve full conversion of the starting material, as monitored by NMR. The resulting product was purified by column chromatography (CHCl_3_/MeOH, gradient from 100:0 to 96:4), yielding 29 mg of 1,4-azaphosphinine **4h** (0.086 mmol, 58%) as a yellow amorphous solid: ^1^H NMR (400 MHz, CDCl_3_) δ 7.29 (dd, *J* = 7.7, 1.6 Hz, 1H), 7.26–7.15 (m, 5H), 7.05–6.96 (m, 2H), 6.86 (ddd, *J* = 8.8, 7.3, 1.6 Hz, 1H), 6.77 (d, *J* = 9.4 Hz, 1H), 6.63 (d, *J* = 8.4 Hz, 1H), 5.86 (dd, *J* = 3.1, 2.2 Hz, 1H), 5.40 (t, *J* = 2.9 Hz, 1H), 4.04 (dqd, *J* = 8.4, 7.0, 1.3 Hz, 2H), 1.32 (t, *J* = 7.0 Hz, 3H); ^13^C­{^1^H} NMR (101 MHz, CDCl_3_) δ 153.3, 149.9 (d, *J* = 2.6 Hz), 138.5 (d, *J* = 12.3 Hz), 137.5, 129.2, 128.9, 128.8 (d, *J* = 2.6 Hz, 2C), 127.7 (2C), 127.6, 127.3, 126.4 (d, *J* = 15.4 Hz), 124.7, 123.8, 123.7, 108.2 (d, *J* = 127.7 Hz), 94.0 (d, *J* = 140.7 Hz), 61.1 (d, *J* = 6.1 Hz), 16.9 (d, *J* = 6.5 Hz); ^31^P­{^1^H} NMR (162 MHz, CDCl_3_) δ 18.54; *R_f_* = 0.62 (92:8 CHCl_3_/MeOH); EI MS 335 (<1%, M^+^), 243 (100%); HRMS (ESI/QTOF) *m*/*z* [M + H]^+^ calcd for [C_20_H_19_NO_2_P]^+^ 336.1148, found 336.1147 (100%).

#### 3-Ethoxy-1-phenyl-[1,4]­azaphosphinino­[1,2-*a*]­quinoxaline 3-Oxide (**4i**)

GP3_A_ was followed with **3i** (100 mg, 0.149 mmol), CF_3_COOAg (3.3 mg, 15 μmol), and 6 mL of DCM. The reaction mixture was stirred at room temperature for 18 days to achieve approximately 50% conversion of the starting material, as monitored by NMR. The resulting product was purified by column chromatography (EtOAc/MeOH, gradient from 100:0 to 94:6), yielding 30 mg of 1,4-azaphosphinine **4i** (0.089 mmol, 30%) as a yellow amorphous solid: ^1^H NMR (400 MHz, CDCl_3_) δ 8.31 (s, 1H), 7.62 (dd, *J* = 7.9, 1.6 Hz, 1H), 7.37–7.27 (m, 3H), 7.25–7.20 (m, 2H), 7.10 (td, *J* = 7.6, 1.2 Hz, 1H), 6.89 (ddd, *J* = 8.8, 7.3, 1.6 Hz, 1H), 6.54 (dd, *J* = 8.3, 1.2 Hz, 1H), 5.89 (dd, *J* = 3.3, 1.8 Hz, 1H), 5.81–5.73 (m, 1H), 4.18–4.05 (m, 2H), 1.35 (t, *J* = 7.1 Hz, 3H); ^13^C­{^1^H} NMR (101 MHz, CDCl_3_) δ 154.2 (d, *J* = 16.0 Hz), 151.7, 142.5 (d, *J* = 2.3 Hz), 137.1 (d, *J* = 12.0 Hz), 136.2, 130.1, 129.9, 129.2 (2C), 129.1, 127.9, 127.8 (2C), 124.8, 122.4, 108.7 (d, *J* = 130.0 Hz), 98.9 (d, *J* = 136.2 Hz), 61.7 (d, *J* = 6.0 Hz), 16.9 (d, *J* = 6.4 Hz); ^31^P­{^1^H} NMR (162 MHz, CDCl_3_) δ 16.65; *R_f_* = 0.54 (93:7 CHCl_3_/MeOH); EI MS 336 (<1%, M^+^), 244 (100%); HRMS (ESI/QTOF) *m*/*z* [M + H]^+^ calcd for [C_19_H_18_N_2_O_2_P]^+^ 337.1100, found 337.1106 (100%).

#### 2-Ethoxy-4-phenyl-[1,4]­azaphosphinino­[2,1-*a*]­isoquinoline 2-Oxide (**4j**)

GP3_A_ was followed with **3j** (150 mg, 0.447 mmol), CF_3_COOAg (2.0 mg, 8.9 μmol), and 3 mL of DCM. The resulting product was purified by column chromatography (CHCl_3_/MeOH, gradient from 100:0 to 96:4), yielding 126 mg of 1,4-azaphosphinine **4j** (0.376 mmol, 84%) as a white solid. Single crystals suitable for X-ray analysis were obtained by slowly cooling its EtOAc solution: ^1^H NMR (400 MHz, CDCl_3_) δ 8.01 (d, *J* = 7.9 Hz, 1H), 7.51–7.42 (m, 5H), 7.41–7.36 (m, 2H), 7.30 (dd, *J* = 7.4, 1.6 Hz, 1H), 6.85 (d, *J* = 7.8 Hz, 1H), 6.18 (t, *J* = 4.1 Hz, 1H), 6.12 (d, *J* = 7.8 Hz, 1H), 5.73 (d, *J* = 3.9 Hz, 1H), 4.04 (dq, *J* = 9.0, 7.0 Hz, 2H), 1.35 (t, *J* = 7.1 Hz, 3H); ^13^C­{^1^H} NMR (101 MHz, CDCl_3_) δ 153.0 (d, *J* = 2.5 Hz), 148.0 (d, *J* = 3.7 Hz), 137.4 (d, *J* = 12.7 Hz), 130.7, 130.1 (d, *J* = 2.2 Hz), 129.8, 129.3 (2C), 128.8 (2C), 128.4, 128.3, 127.3 (d, *J* = 13.2 Hz), 126.4, 124.8, 107.6, 103.7 (d, *J* = 126.9 Hz), 88.4 (d, *J* = 141.9 Hz), 61.7 (d, *J* = 6.0 Hz), 17.0 (d, *J* = 6.5 Hz); ^31^P­{^1^H} NMR (162 MHz, CDCl_3_) δ 17.84; *R_f_* = 0.33 (95:5 CHCl_3_/MeOH); EI MS 335 (<1%, M^+^), 243 (100%); HRMS (ESI) *m*/*z* [M + H]^+^ calcd for [C_20_H_19_NO_2_P]^+^ 336.1148, found 336.1149 (100%).

#### 2,11-Diethoxy-4,9-diphenylpyrazino­[1,2-*a*:4,3-*a*′]­bis­([1,4]­azaphosphinine) 2,11-Dioxide (**4l**)

GP3_A_ was followed with **3l** (30 mg, 0.061 mmol), CF_3_COOAg (1.3 mg, 6.1 μmol), and 1.2 mL of DCM. The resulting product was purified by column chromatography (CHCl_3_/MeOH, gradient from 100:0 to 96:4), yielding 28 mg of 1,4-azaphosphinine **4l** (0.057 mmol, 93%) as a yellow solid: ^1^H NMR (400 MHz, CDCl_3_) δ 7.49–7.39 (m, 6H), 7.38–7.30 (m, 4H), 6.29 (t, *J* = 3.6 Hz, 2H), 5.74 (s, 2H), 5.65 (d, *J* = 3.7 Hz, 2H), 4.10 (dq, *J* = 9.2, 7.0 Hz, 4H), 1.38 (t, *J* = 7.1 Hz, 6H); ^13^C­{^1^H} NMR (101 MHz, CDCl_3_) δ 152.2 (d, *J* = 2.6 Hz, 2C), 142.3 (dd, *J* = 16.2, 4.7 Hz, 2C), 135.4 (d, *J* = 12.8 Hz, 2C), 130.3 (2C), 129.2 (4C), 128.8 (4C), 110.1 (2C), 102.0 (d, *J* = 131.0 Hz, 2C), 97.2 (d, *J* = 133.5 Hz, 2C), 62.2 (d, *J* = 6.1 Hz, 2C), 17.0 (d, *J* = 6.3 Hz, 2C); ^31^P­{^1^H} NMR (162 MHz, CDCl_3_) δ 17.03; *R_f_* = 0.55 (93:7 CHCl_3_/MeOH); HRMS (ESI) *m*/*z* [M + H]^+^ calcd for [C_26_H_27_N_2_O_4_P_2_]^+^ 493.1441, found 493.1439 (100%); mp 111.0–113.2 °C (EtOAc).

## Supplementary Material





## Data Availability

The data underlying this study are available in the published article and its . Additional primary research data have been uploaded to a compliant third-party repository and are publicly available at 10.5281/zenodo.17302225.

## Notes

^1^H, ^13^C, ^31^P, and ^19^F NMR spectra, computational data, and UV–vis data (PDF)
FAIR data, including the primary NMR FID files, for compounds **1a**–**u**, **2a**–**u**, **3a**–**m**, **4a**–**l**, **7r**–**u**, and **8p**–**u** (ZIP)
