# Supplementary material for: Synthesis of Quinolizidine-Based 1,4-Azaphosphinines via Cyclization of Heteroarylmethyl(alkynyl)phosphinates
Source: J Org Chem. 2025 Dec 22;91(1):698–719. doi: 10.1021/acs.joc.5c02870 (PMC12797286; doi:10.1021/acs.joc.5c02870)
Supplement: Supplementary file 1 [file jo5c02870_si_001.pdf]

Supporting Information

Synthesis of Quinolizidine-based 1,4-Azaphosphinines via Cyclization of Heteroarylmethyl(alkynyl)phosphinates

Martin Kos[1,*], Tomáš Beránek[1], Jaroslav Žádný[1], Natálie Kochová[1], Illia Panov[1], Karolína Václavíková[1], Jan Storch[1], Ivana Císařová[2], Jan Sýkora[3], and Vladimír Církva[1]

[1]Department of Advanced Materials and Organic Synthesis, Institute of Chemical Process Fundamentals of the Czech Academy of Sciences, v. v. i., Rozvojová 135, 165 00 Prague 6, Czech Republic

[2]Department of Inorganic Chemistry, Faculty of Science, Charles University in Prague, Hlavova 2030, 128 40 Prague 2, Czech Republic

[3]Department of Analytical Chemistry, Faculty of Chemical Engineering, University of Chemistry and Technology, Prague, Technická 5, 166 28 Prague 6, Czech Republic

Table of Contents

1. Synthetic Schemes and Procedures S3

2. ^{1}H, ^{13}C, ^{19}F, and ^{31}P NMR Spectra of Prepared Compounds S5

3. DFT Calculations S142

4. UV-Vis spectra of Selected Compound and Excitation/Emission spectra of **4l** S186

6. X-ray analysis S190

7. References S193

1. Synthetic Schemes and Procedures

Cyclization of *Ethyl (phenyl(pyridin-2-yl)methyl)(phenylethynyl)phosphinate (**1p**) in TFA*

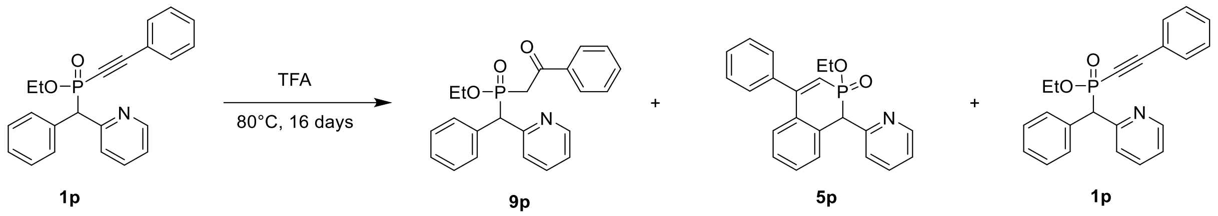

Molar ratio: **10** : **1.7** : **1**

Scheme S1 Reaction of **1p** in TFA.

A Schlenk flask was charged with 140 mg of phosphinate **1p** (0.387 mmol), and 3.8 mL of TFA under an inert atmosphere. The resulting mixture was stirred at 80°C for 16 days. The reaction mixture was neutralized by saturated aqueous solution of sodium bicarbonate. The resulting mixture was extracted with dichloromethane (3 x 5 mL). The organic phase was washed with brine, and dried over magnesium sulfate The ^{31}P NMR and GC-MS analysis (see Fig. S274) revealed following composition of the reaction mixture: ketone **9p** (78 %), cyclic phosphinate **5p** (13 %), and unreacted starting phosphinate **1p** (8 %). As a result, this approach to **5p** was found to be unsuitable and was not investigated further.

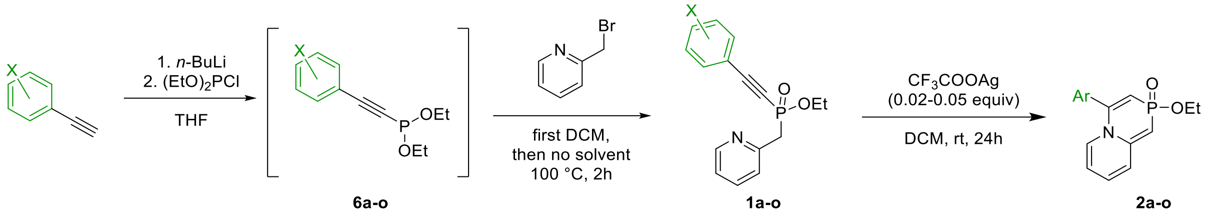

Scheme S2 Preparation of 1,4-azaphosphinines **2a-o**.

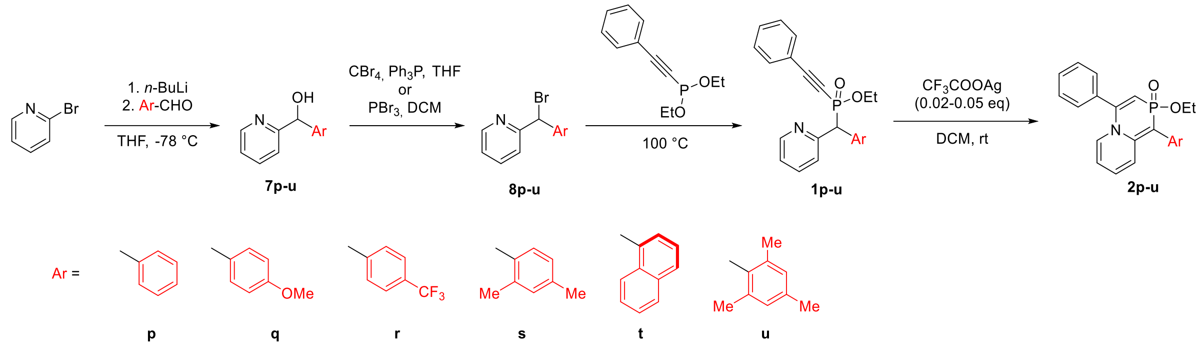

Scheme S3 Preparation of 1,4-azaphosphinines **2p-u**.

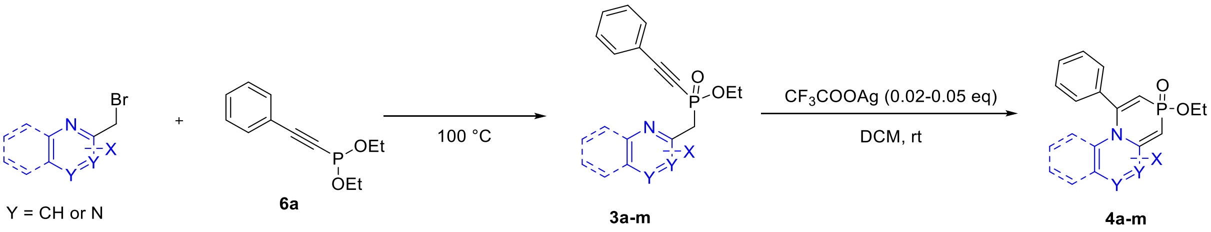

Scheme S4 Preparation of 1,4-azaphosphinines **4a-m**.

2. ^{1}H, ^{13}C, ^{19}F, and ^{31}P NMR Spectra of Prepared Compounds

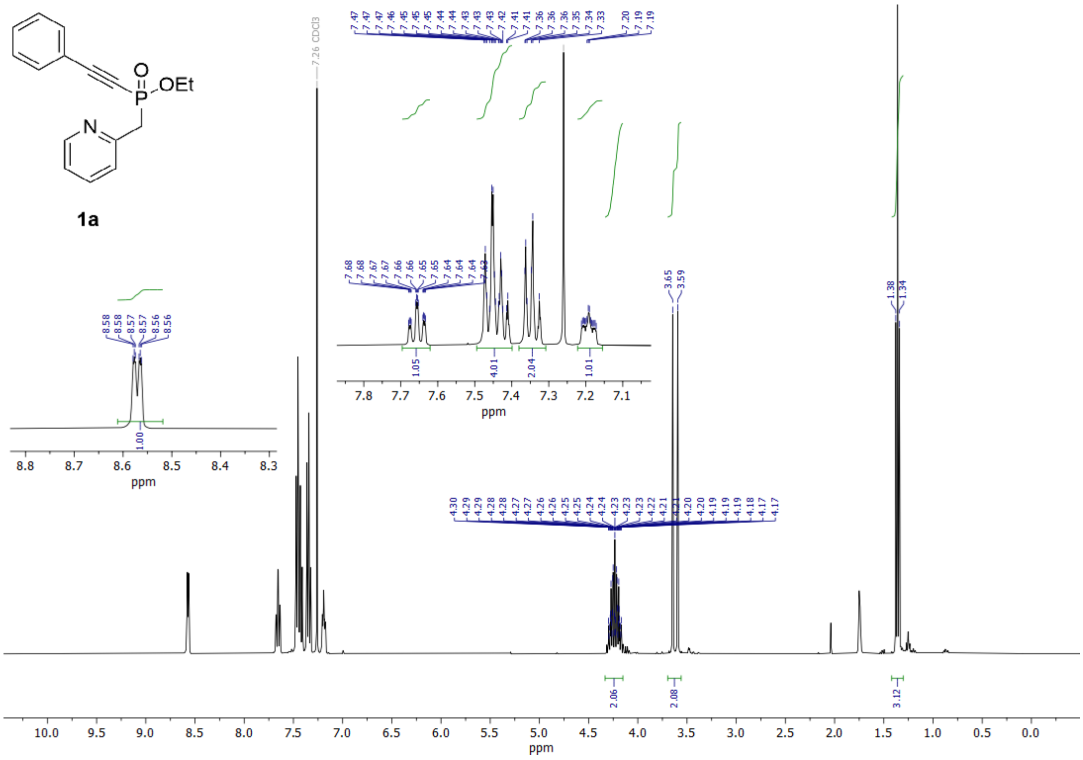

Figure S1 *^{1}H NMR spectrum of **1a** (400 MHz, $CDCl_3$).*

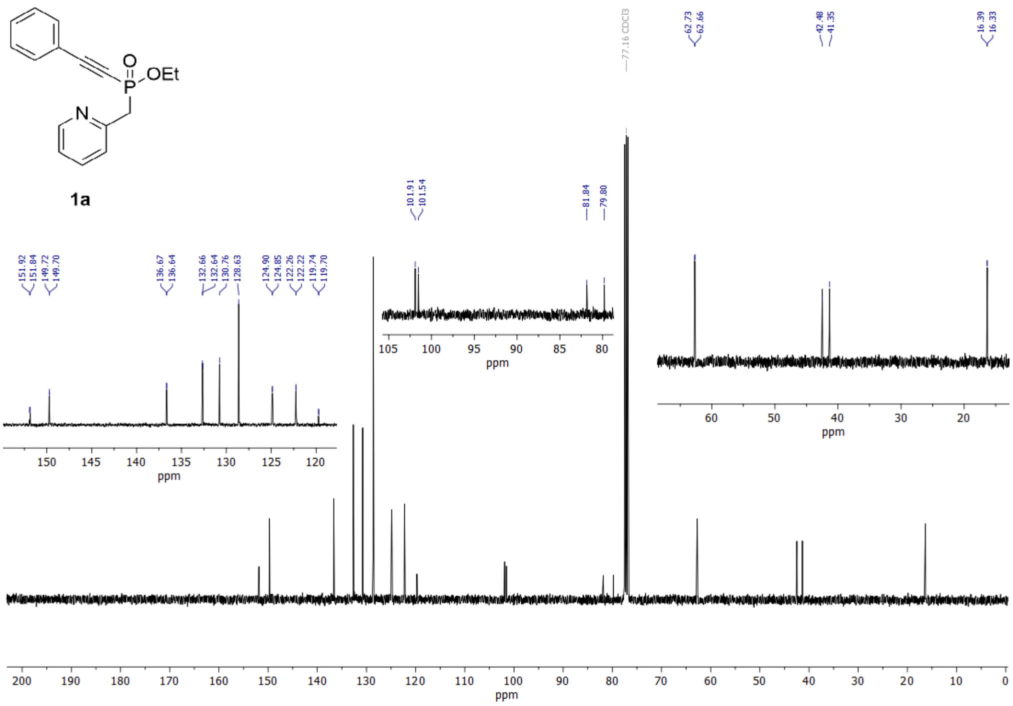

Figure S2 *^{13}C {^{1}H} NMR spectrum of **1a** (101 MHz, $CDCl_3$).*

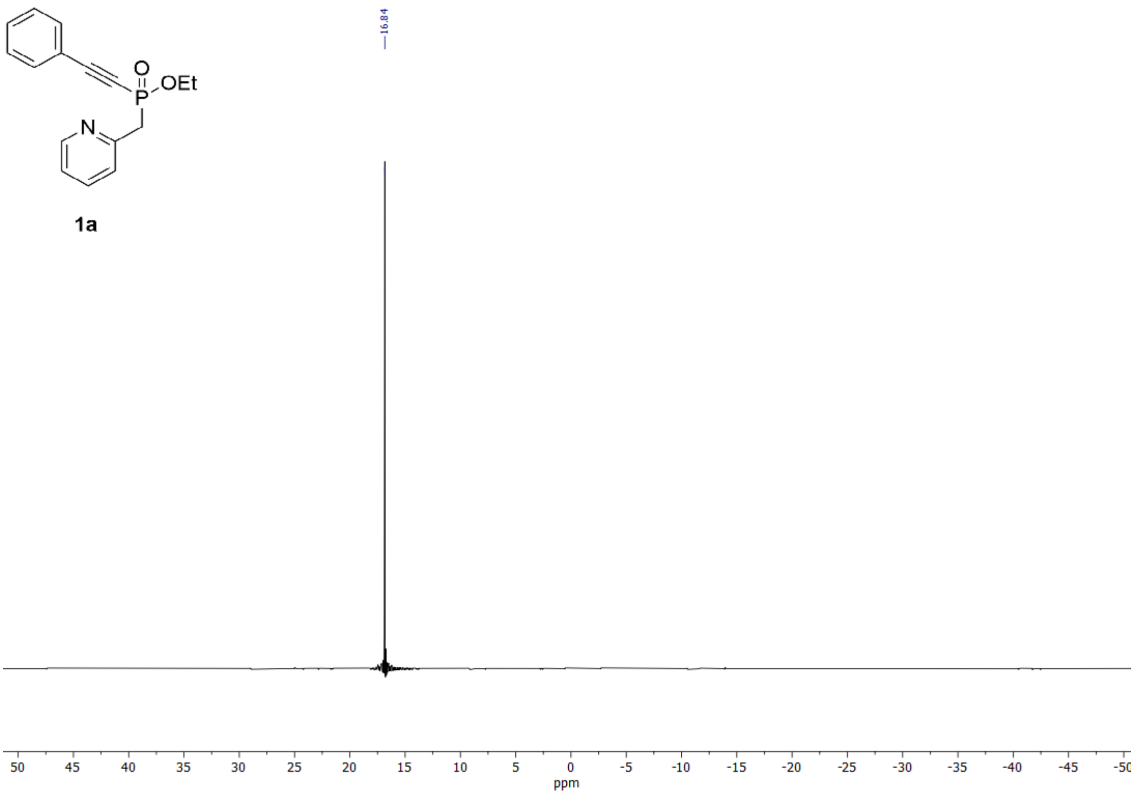

Figure S3 *^{31}P {^{1}H} NMR spectrum of **1a** (162 MHz, $CDCl_3$).*

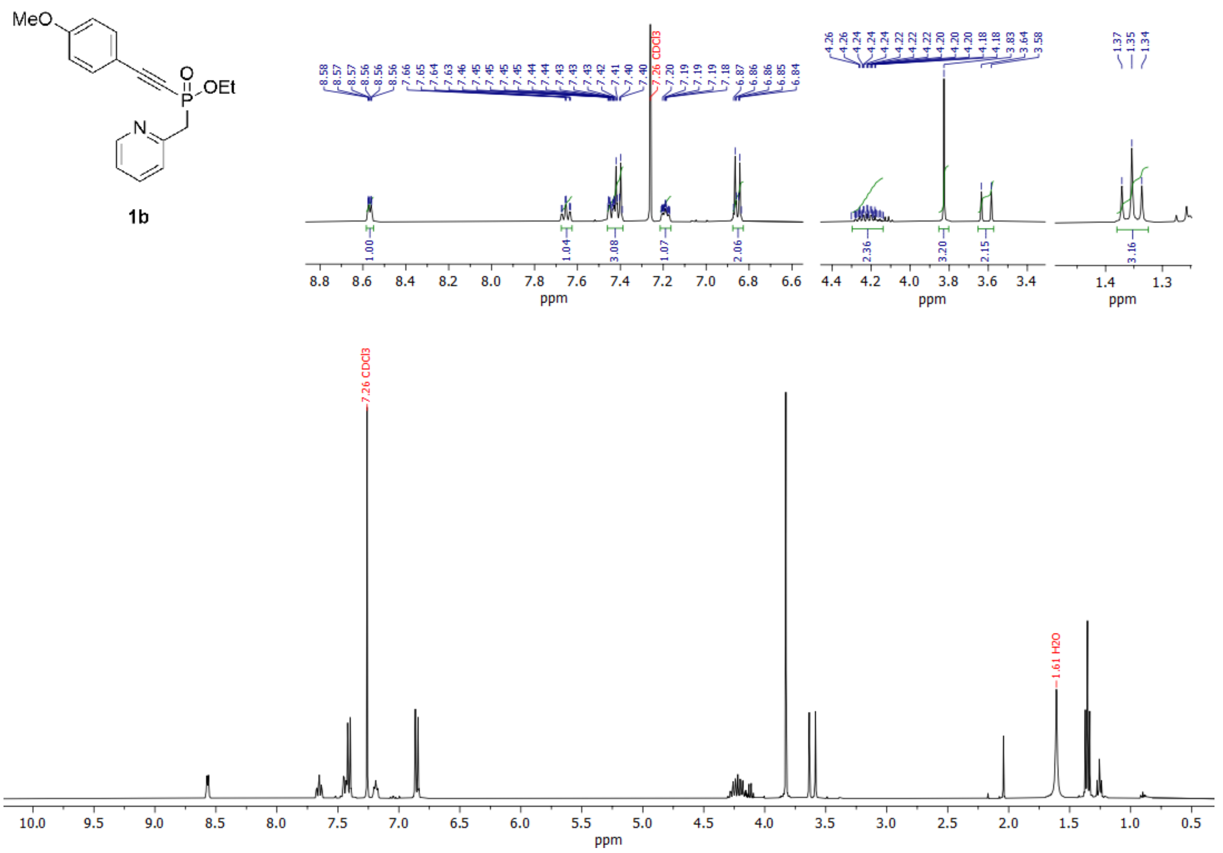

Figure S4 *^{1}H NMR spectrum of **1b** (400 MHz, CDCl$_3$).*

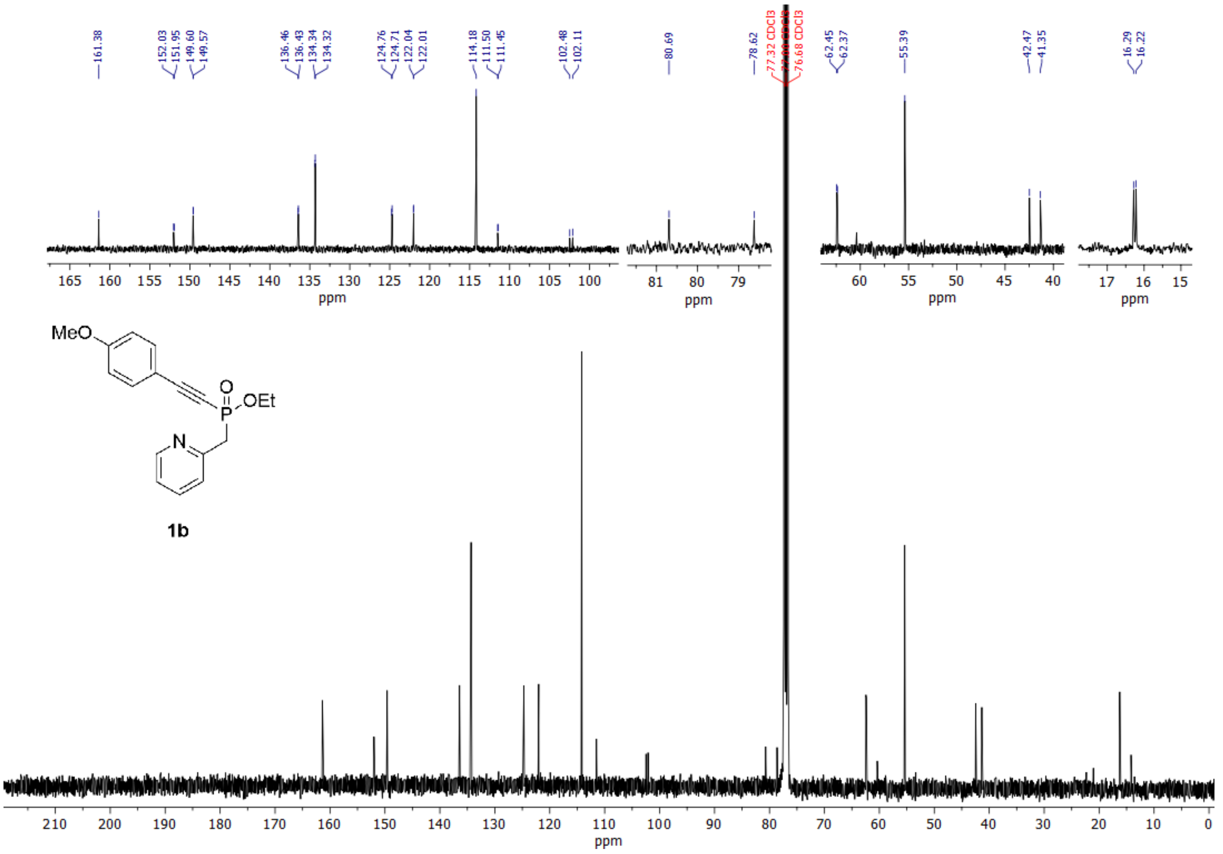

Figure S5 *^{13}C {^{1}H} NMR spectrum of **1b** (101 MHz, CDCl$_3$).*

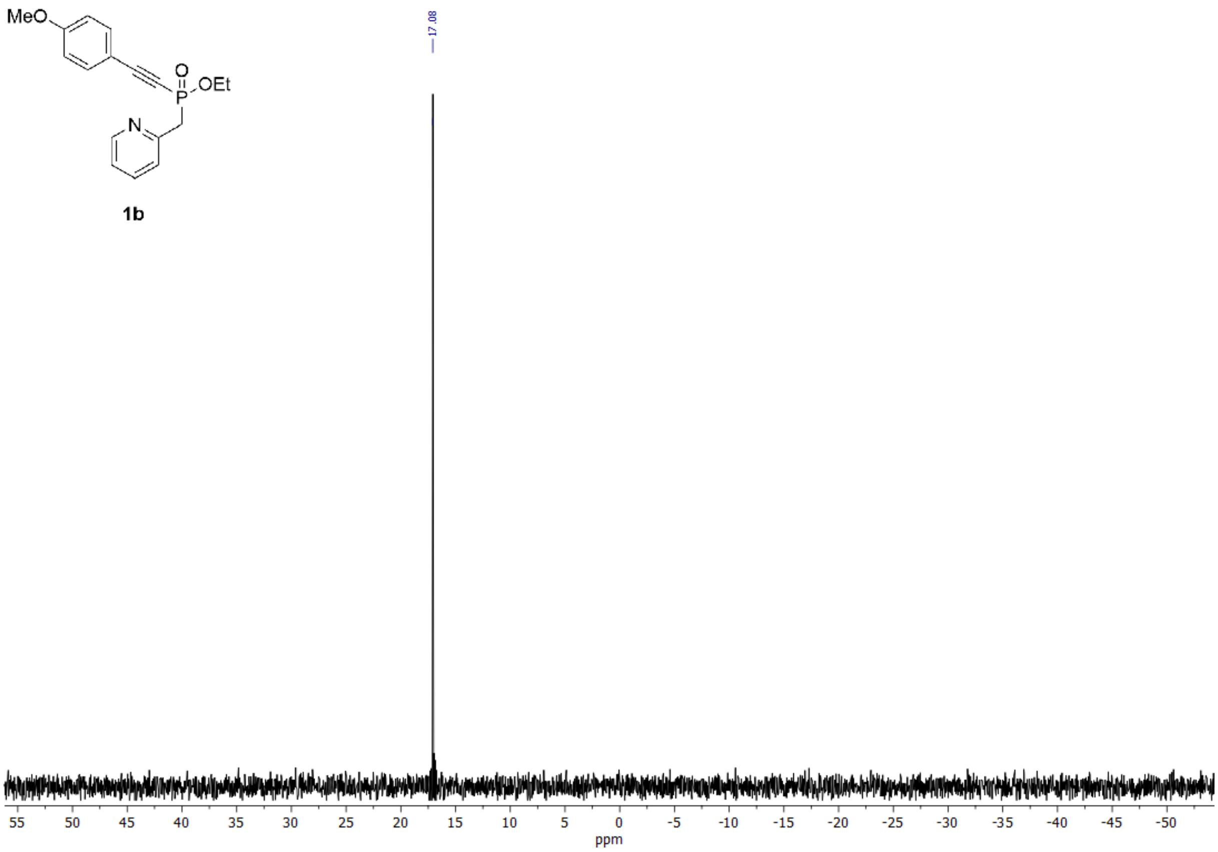

Figure S6 ^{31}P {^{1}H} *NMR spectrum of **1b** (162 MHz, CDCl$_3$).*

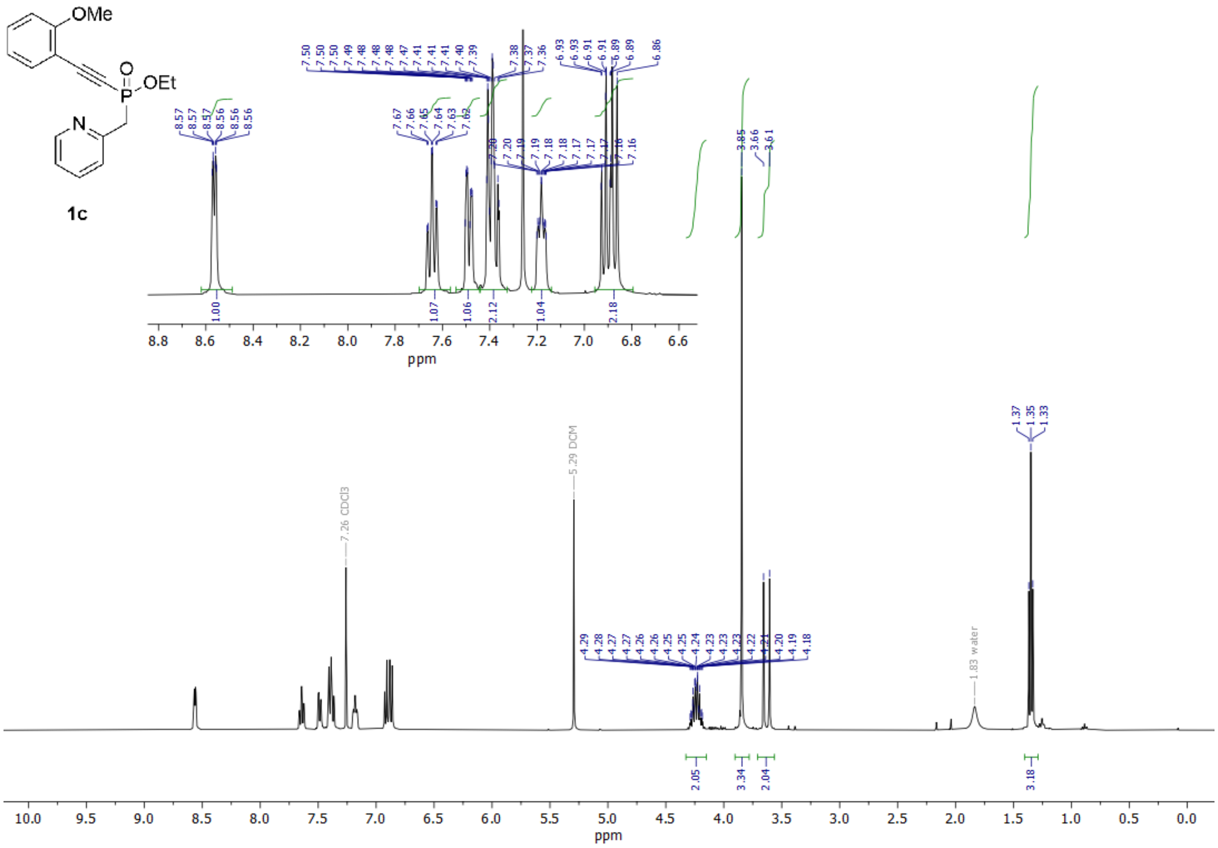

Figure S7 ^{1}H *NMR spectrum of **1c** (400 MHz, CDCl$_3$).*

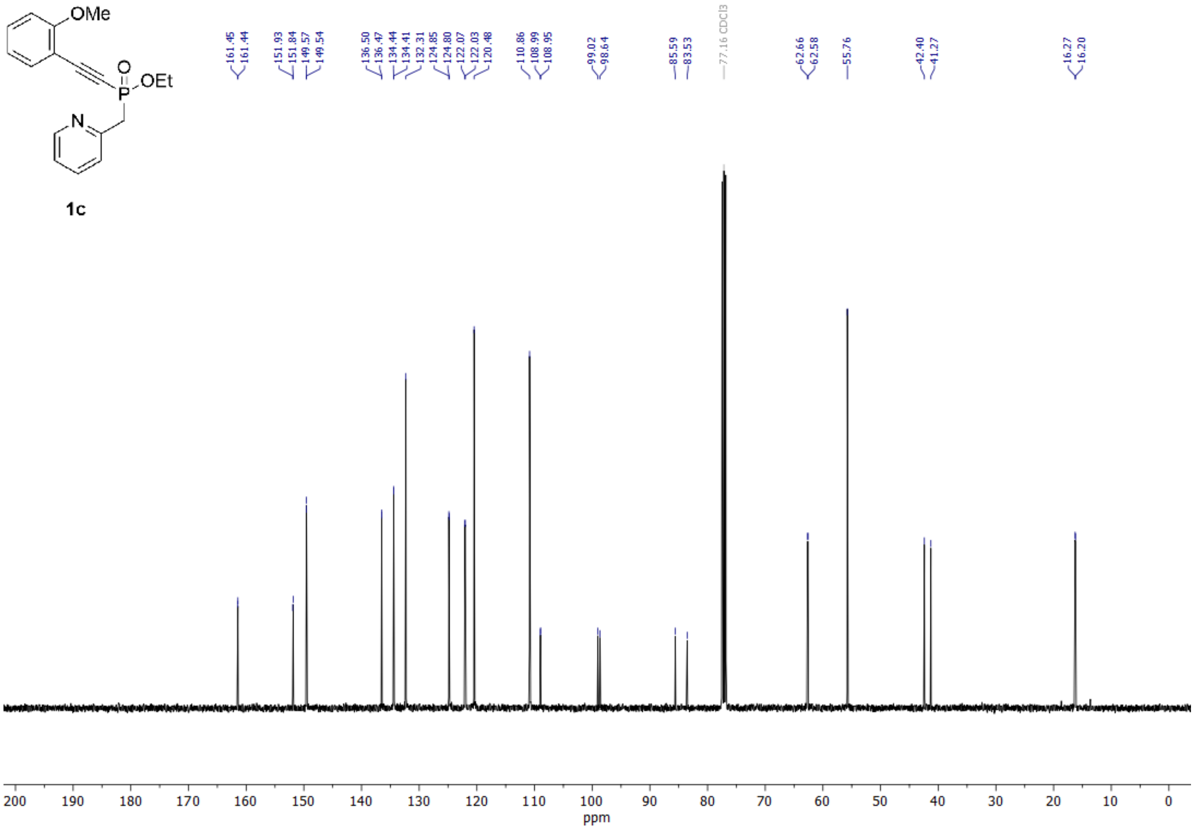

Figure S8 ^{13}C {^{1}H} NMR spectrum of **1c** (101 MHz, $CDCl_3$).

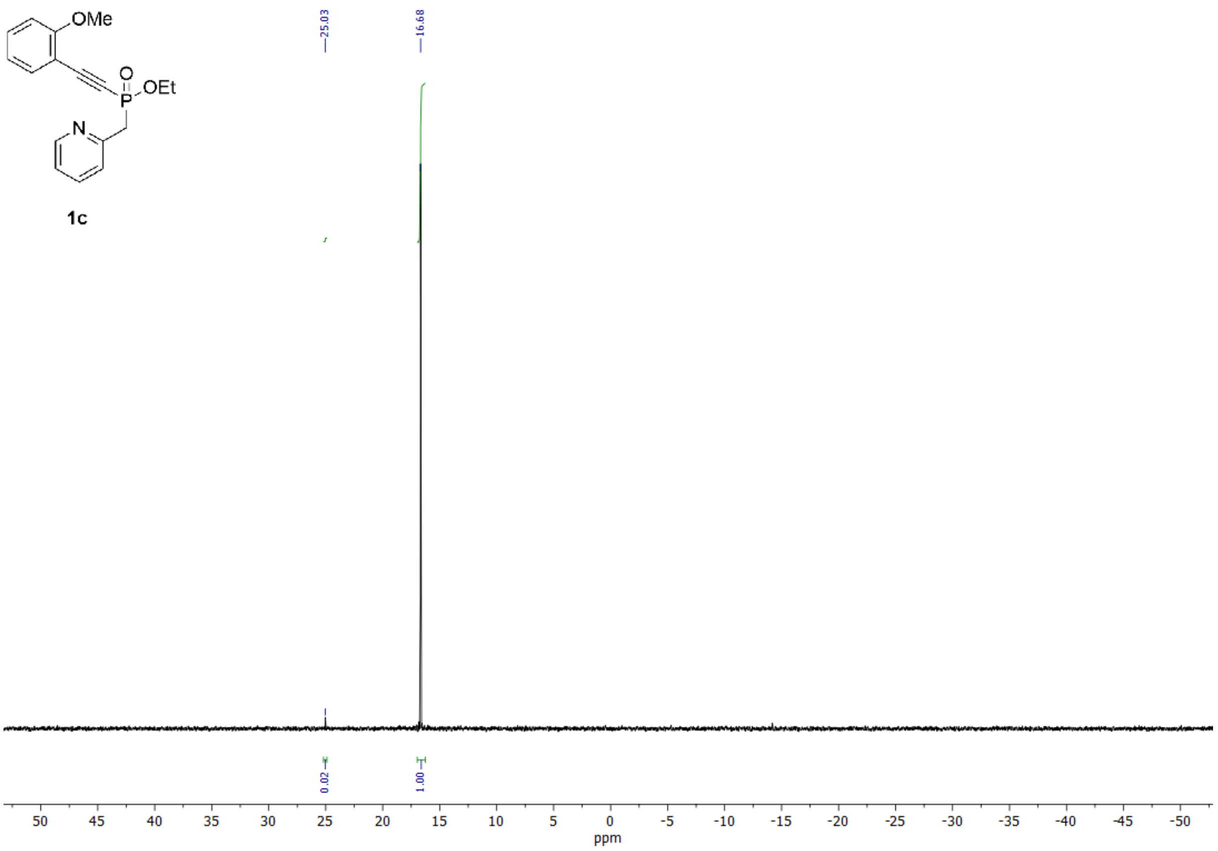

Figure S9 *^{31}P {^{1}H} NMR spectrum of **1c** (162 MHz, $CDCl_3$).*

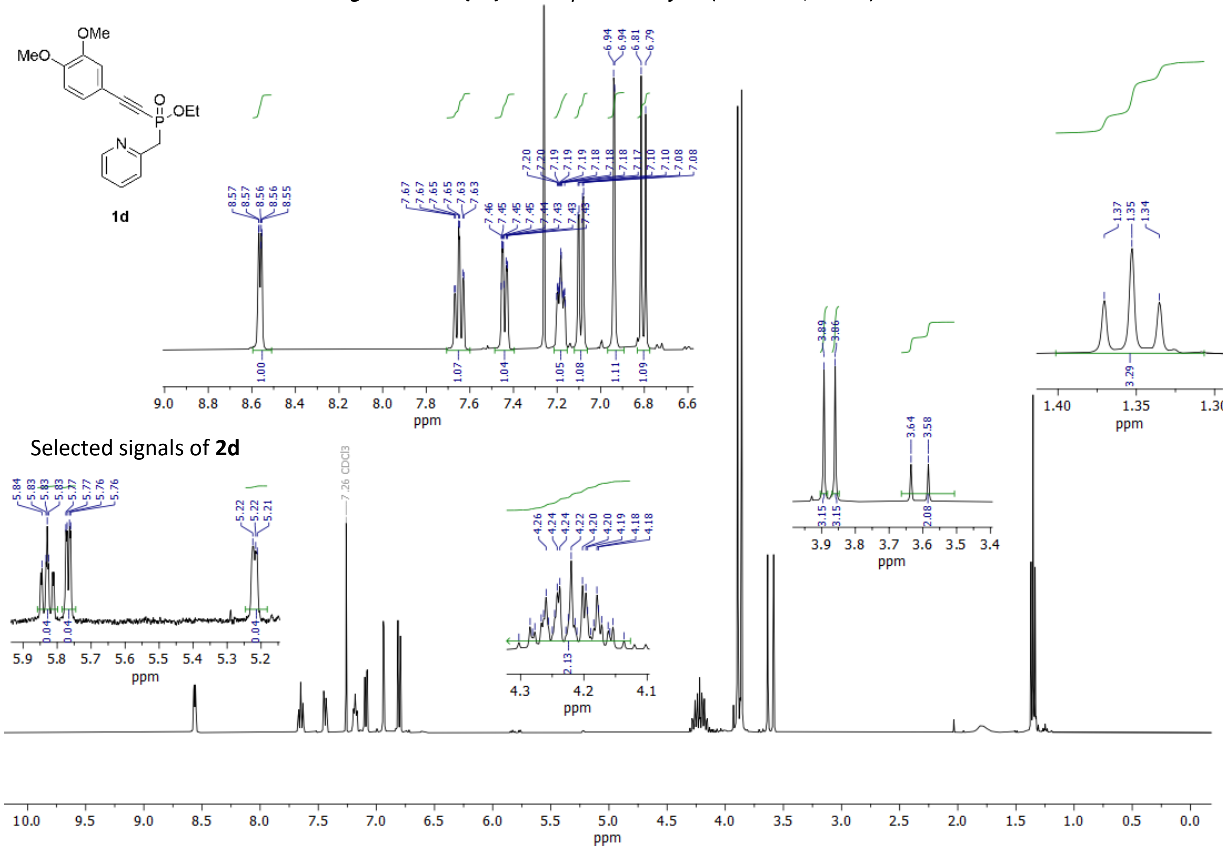

Figure S10 *^{1}H NMR spectrum of **1d** (400 MHz, $CDCl_3$). The signals at 5-6 ppm are attributed to product **2d**, formed via spontaneous cyclization.*

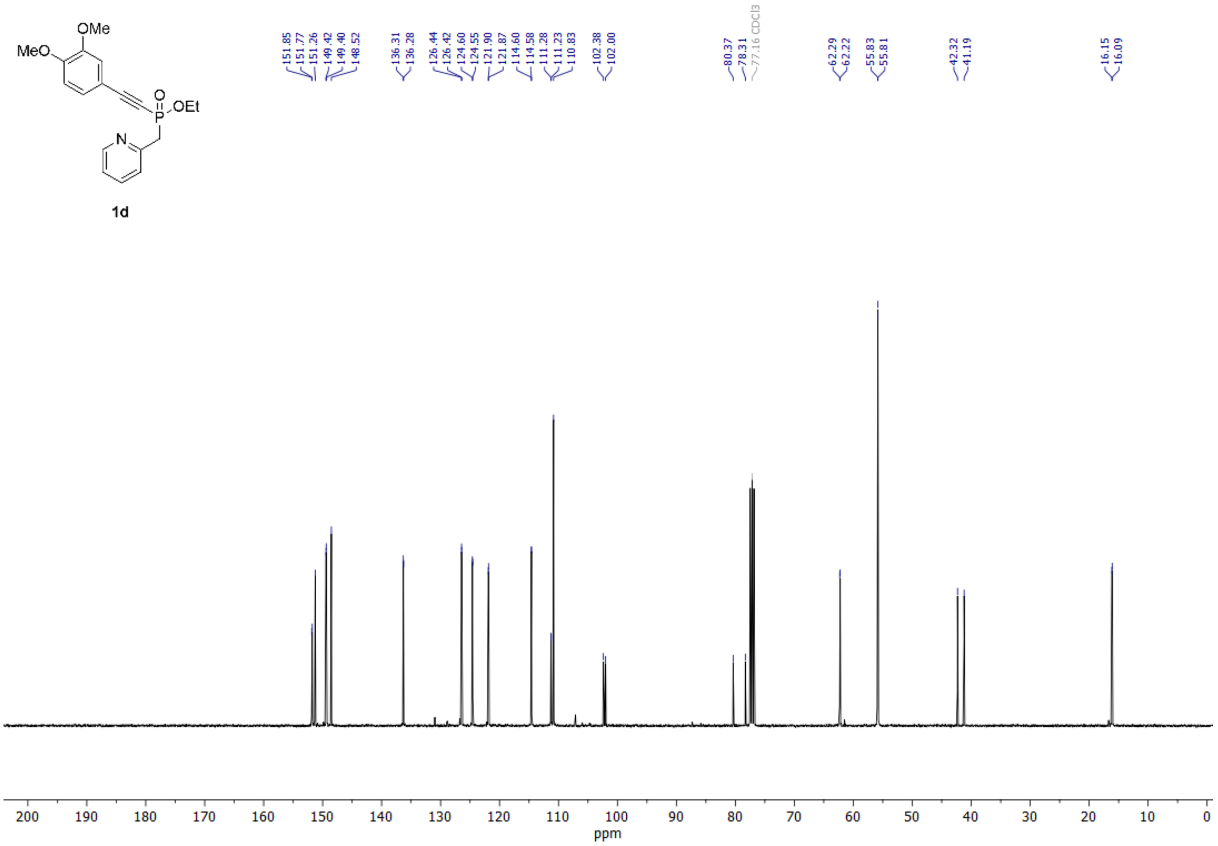

Figure S11 *^{13}C {^{1}H} NMR spectrum of* ***1d*** *(101 MHz, $CDCl_3$).*

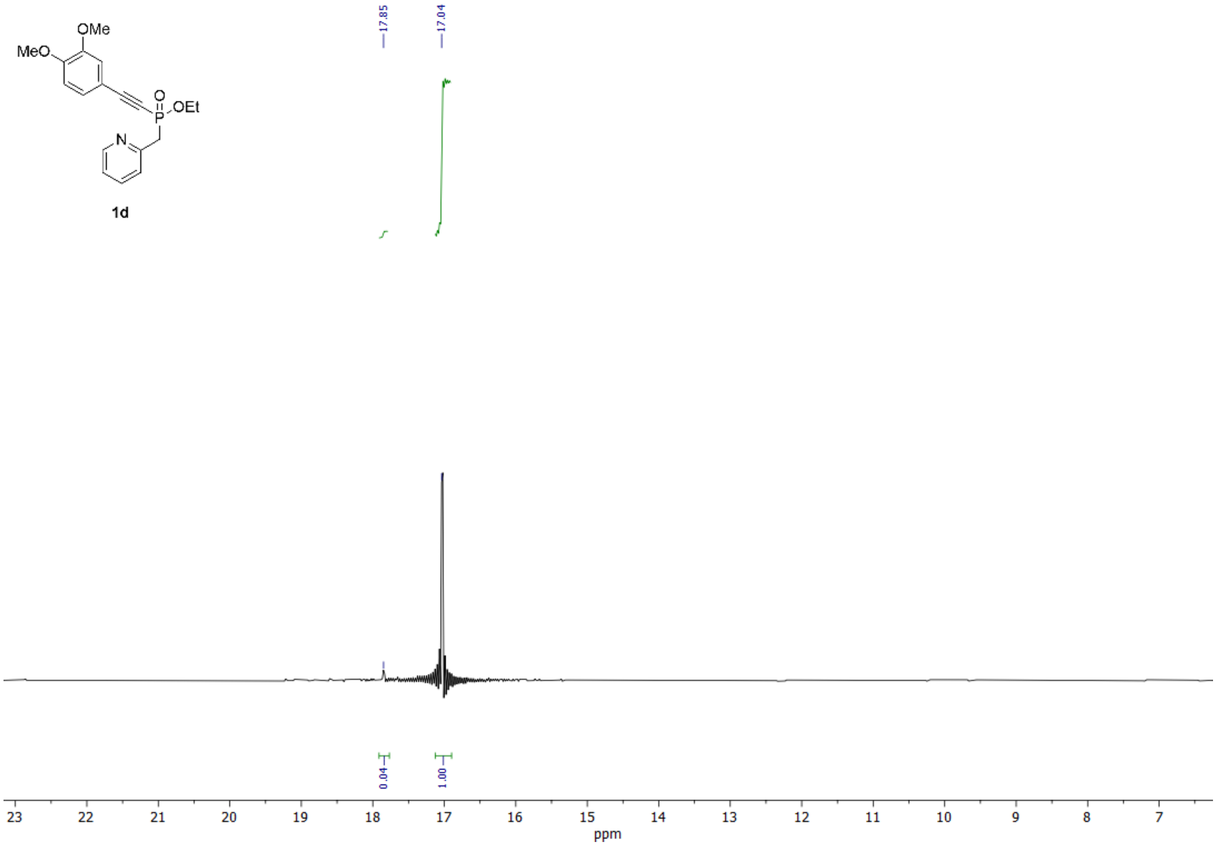

Figure S12 *^{31}P {^{1}H} NMR spectrum of* ***1d*** *(162 MHz, $CDCl_3$). The signal at 17.85 ppm is attributed to product* ***2d****, formed via spontaneous cyclization.*

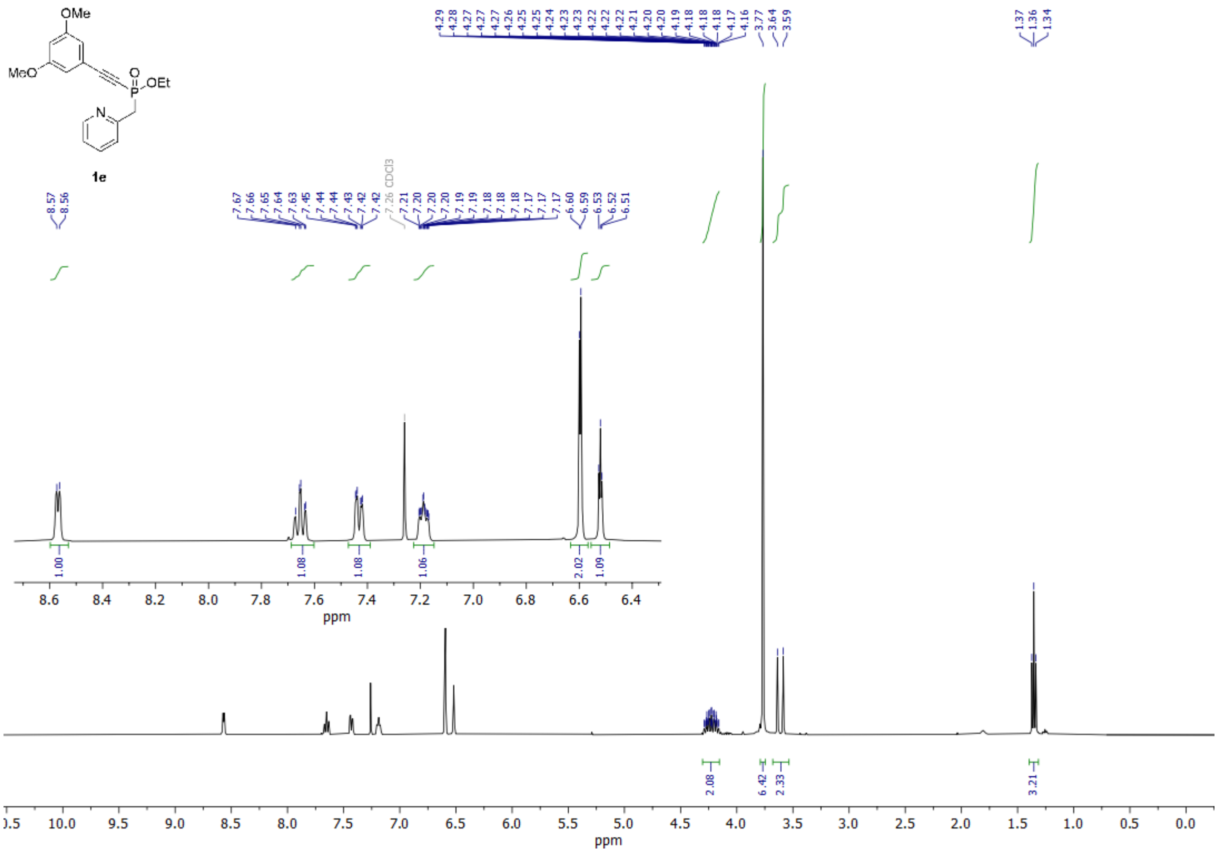

Figure S13 *^{1}H NMR spectrum of **1e** (400 MHz, $CDCl_3$).*

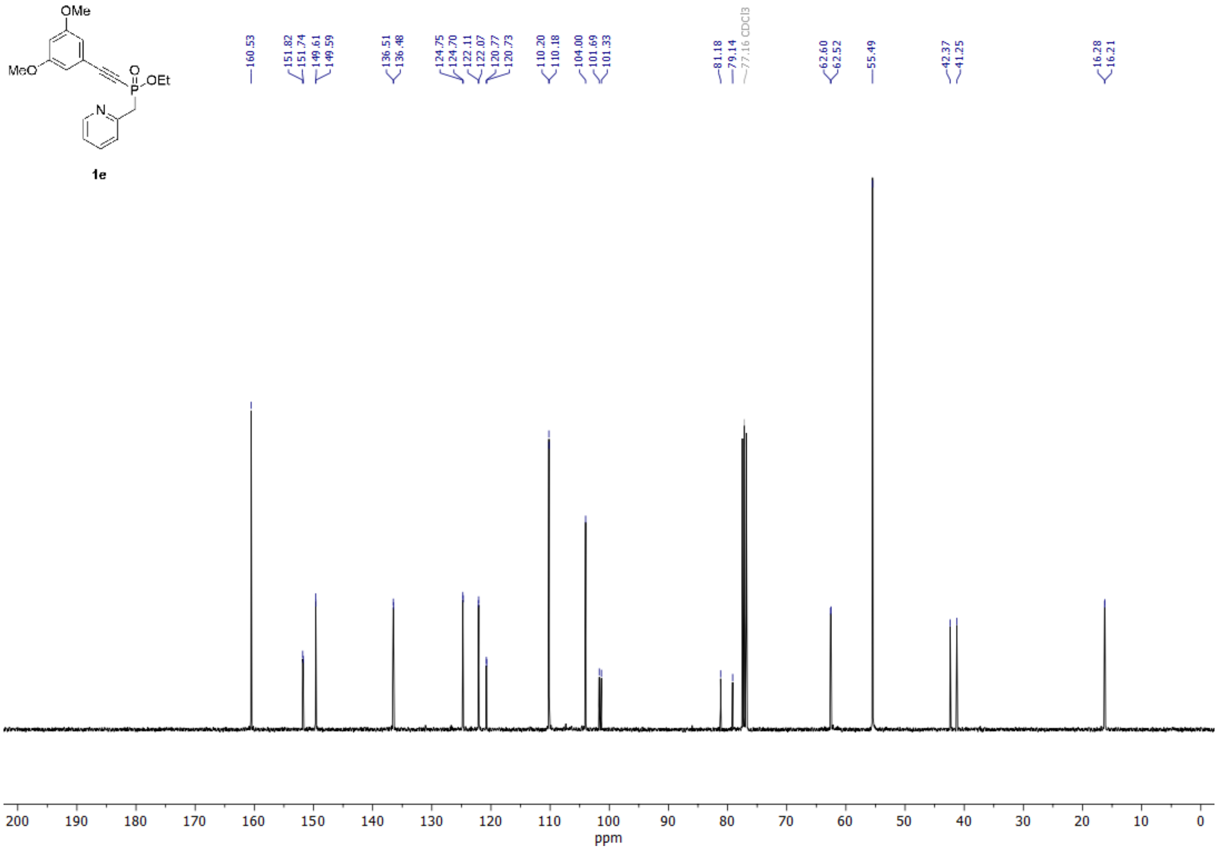

Figure S14 *^{13}C {^{1}H} NMR spectrum of **1e** (101 MHz, $CDCl_3$).*

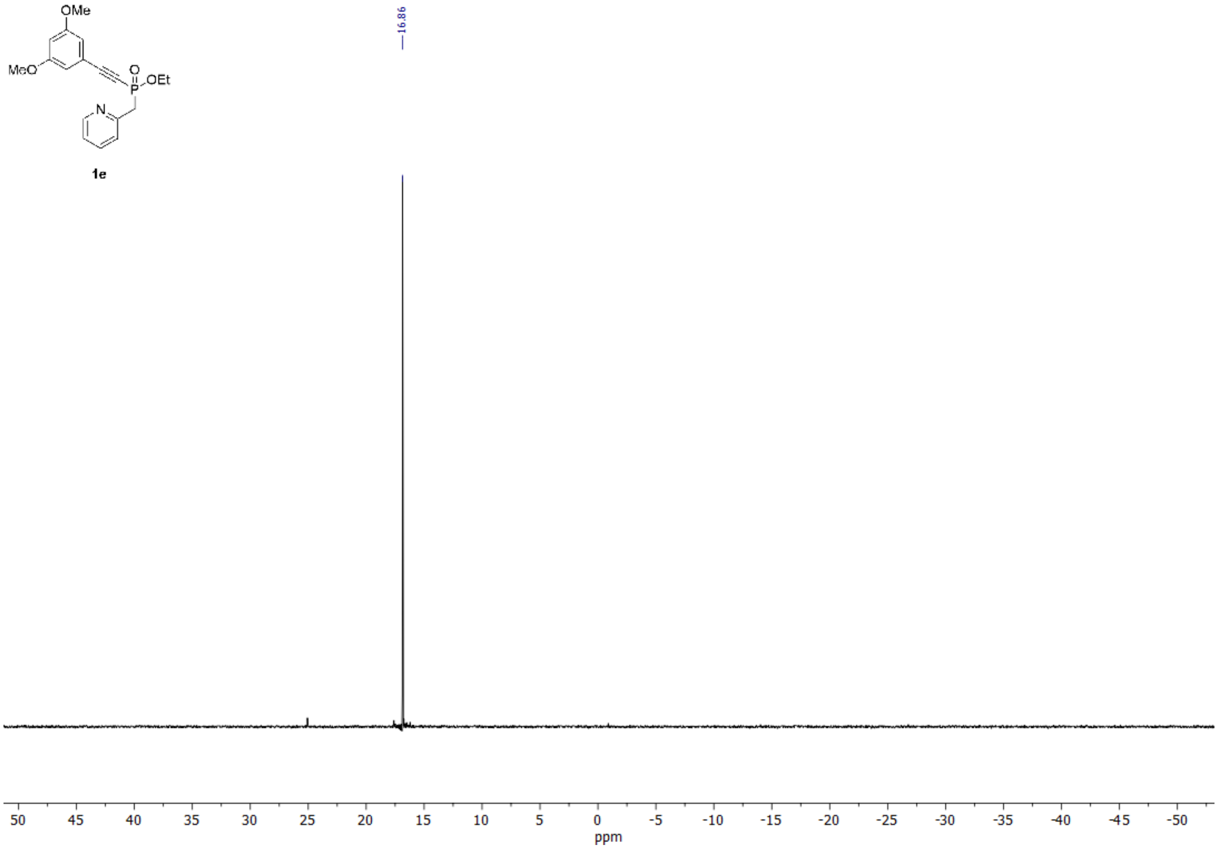

Figure S15 *^{31}P {^{1}H} NMR spectrum of* ***1e*** *(162 MHz, $CDCl_3$).*

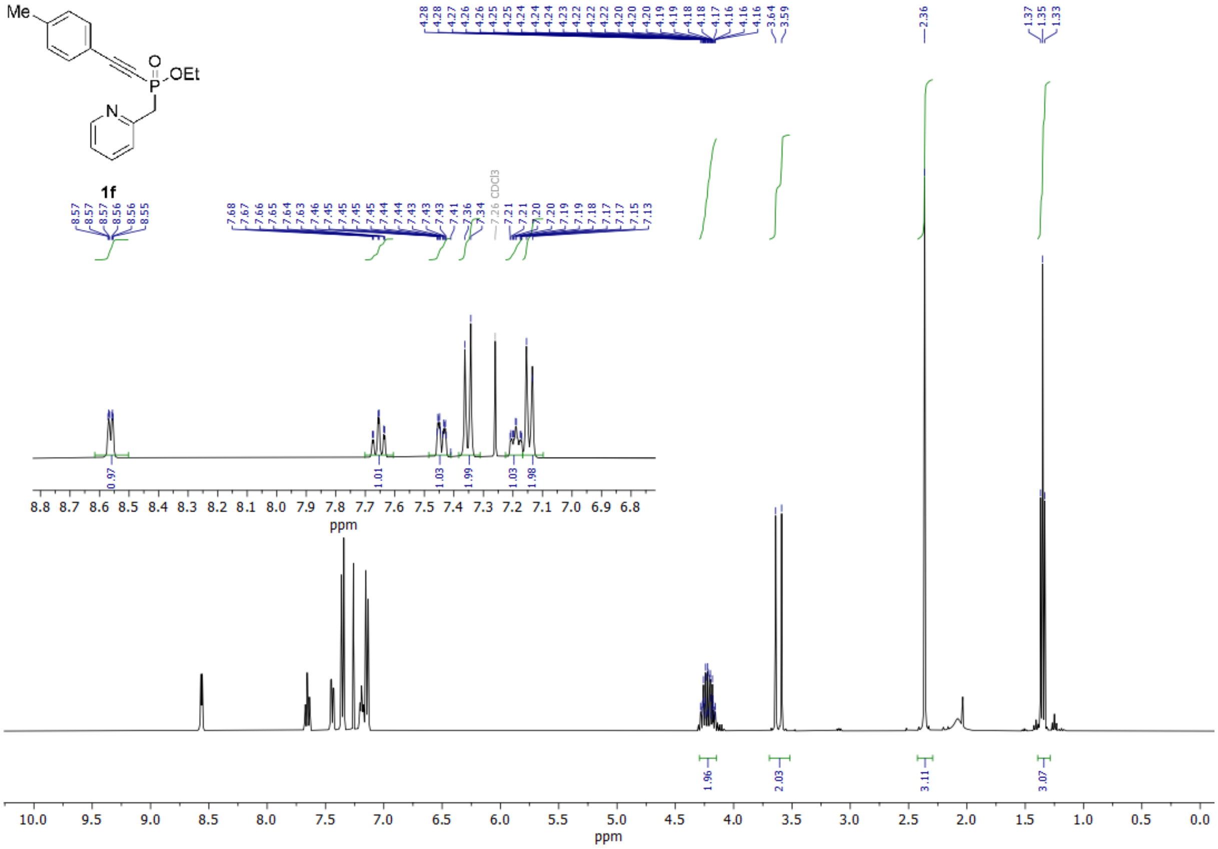

Figure S16 *^{1}H NMR spectrum of* ***1f*** *(400 MHz, $CDCl_3$).*

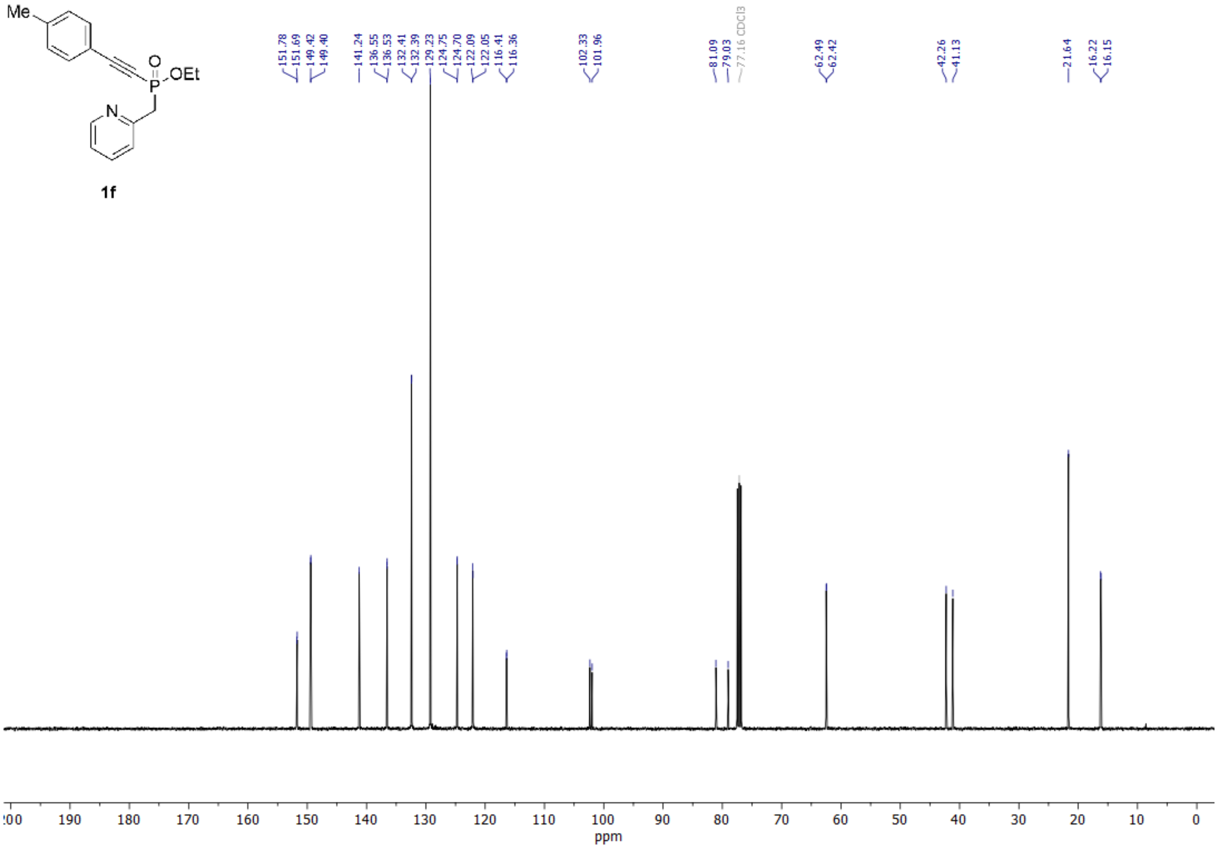

Figure S17 *^{13}C {^{1}H} NMR spectrum of* ***1f*** *(101 MHz, $CDCl_3$).*

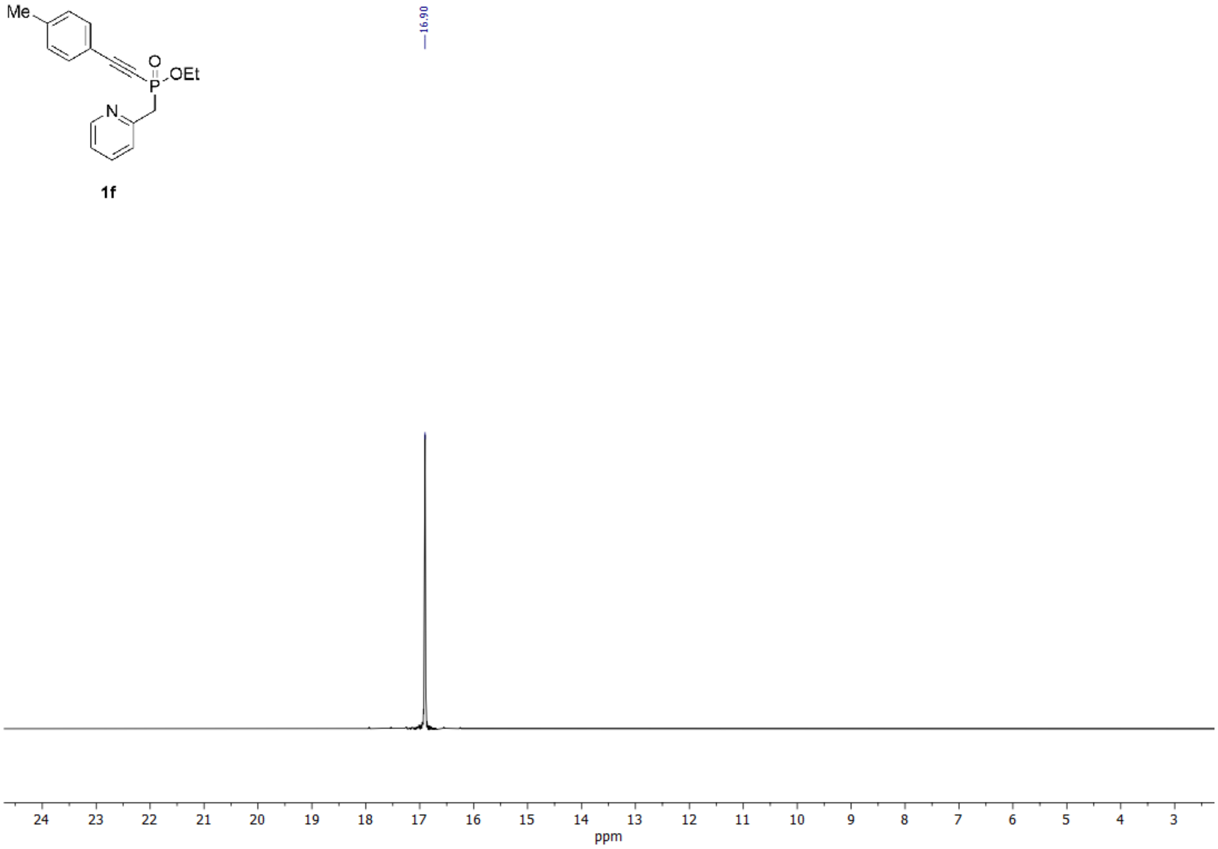

Figure 18 ^{31}P {^{1}H} NMR spectrum of ***1f*** *(162 MHz, $CDCl_3$).*

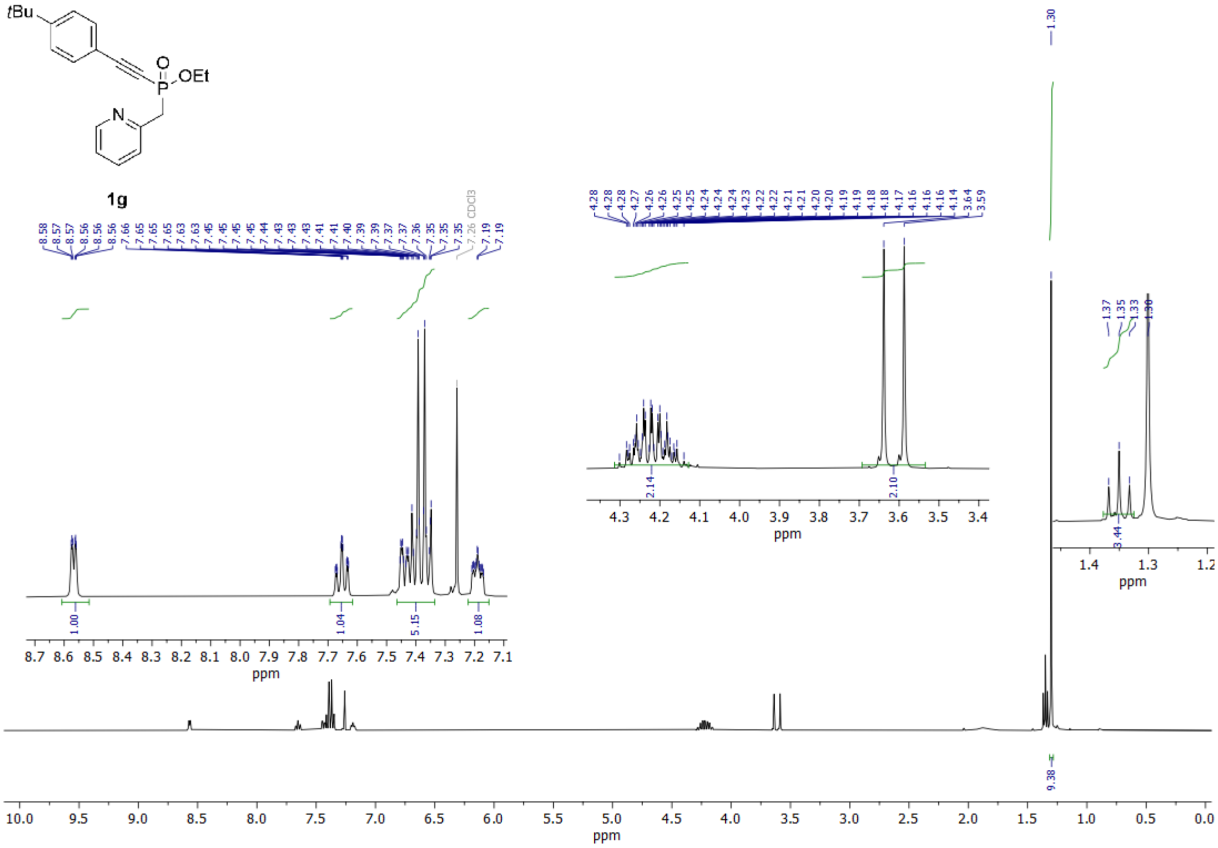

Figure S19 ^{1}H *NMR spectrum of* ***1g*** *(400 MHz, $CDCl_3$).*

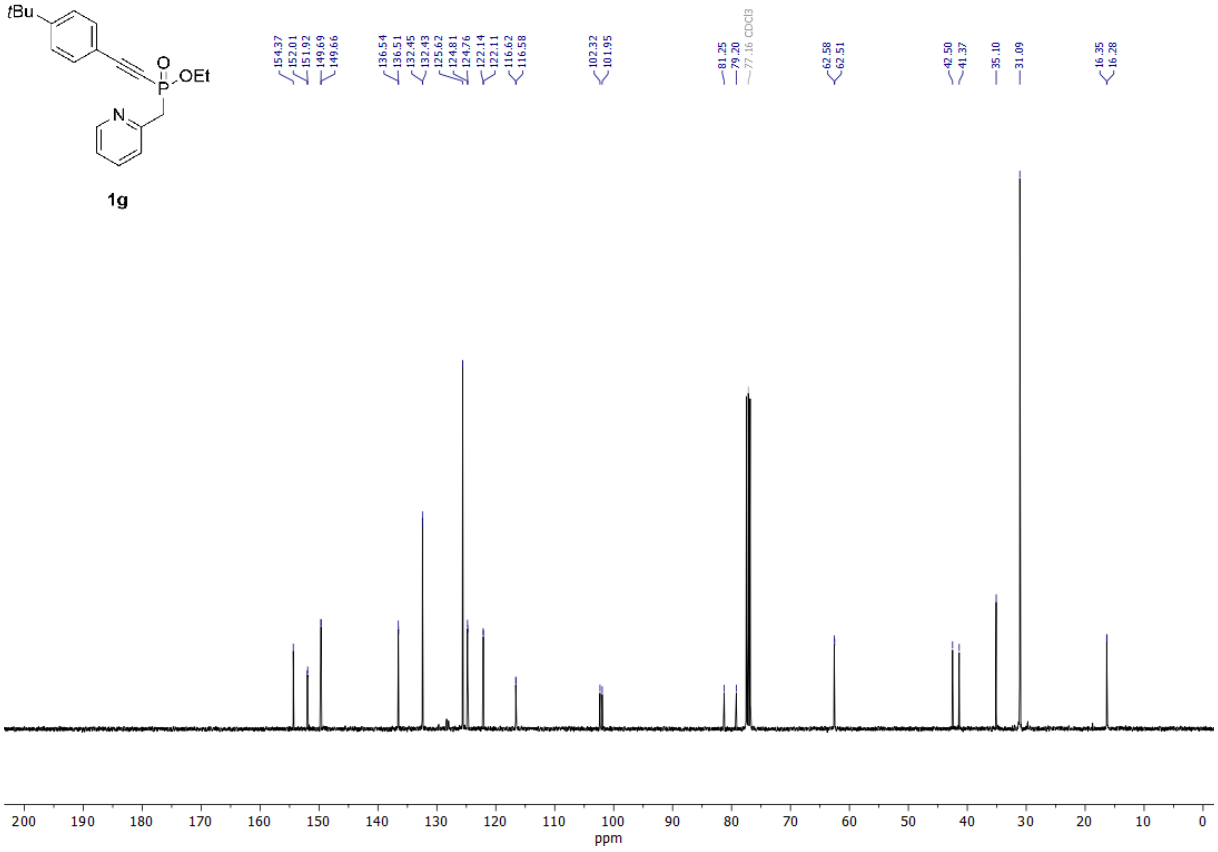

Figure S20 ^{13}C *{^{1}H} NMR spectrum of* ***1g*** *(101 MHz, $CDCl_3$).*

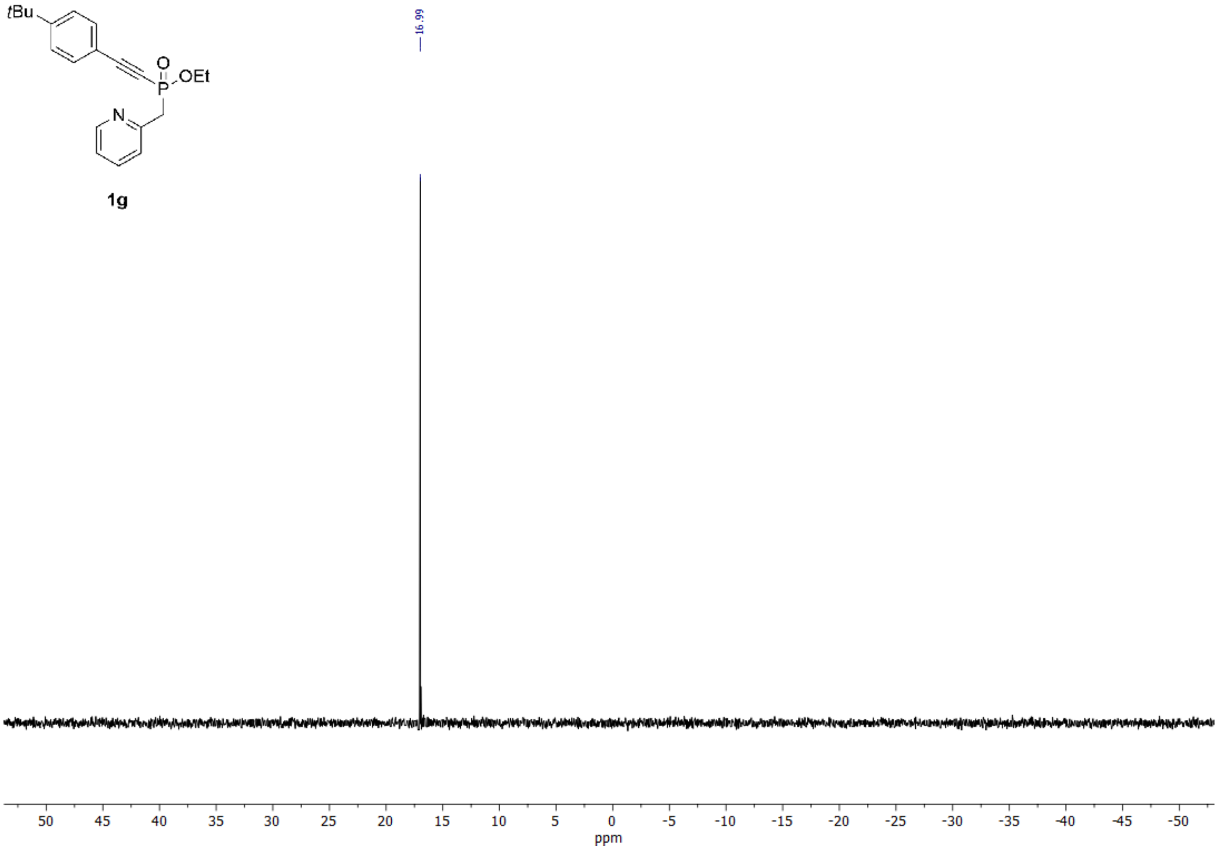

Figure S21 *^{31}P {^{1}H} NMR spectrum of **1g** (162 MHz, CDCl$_3$).*

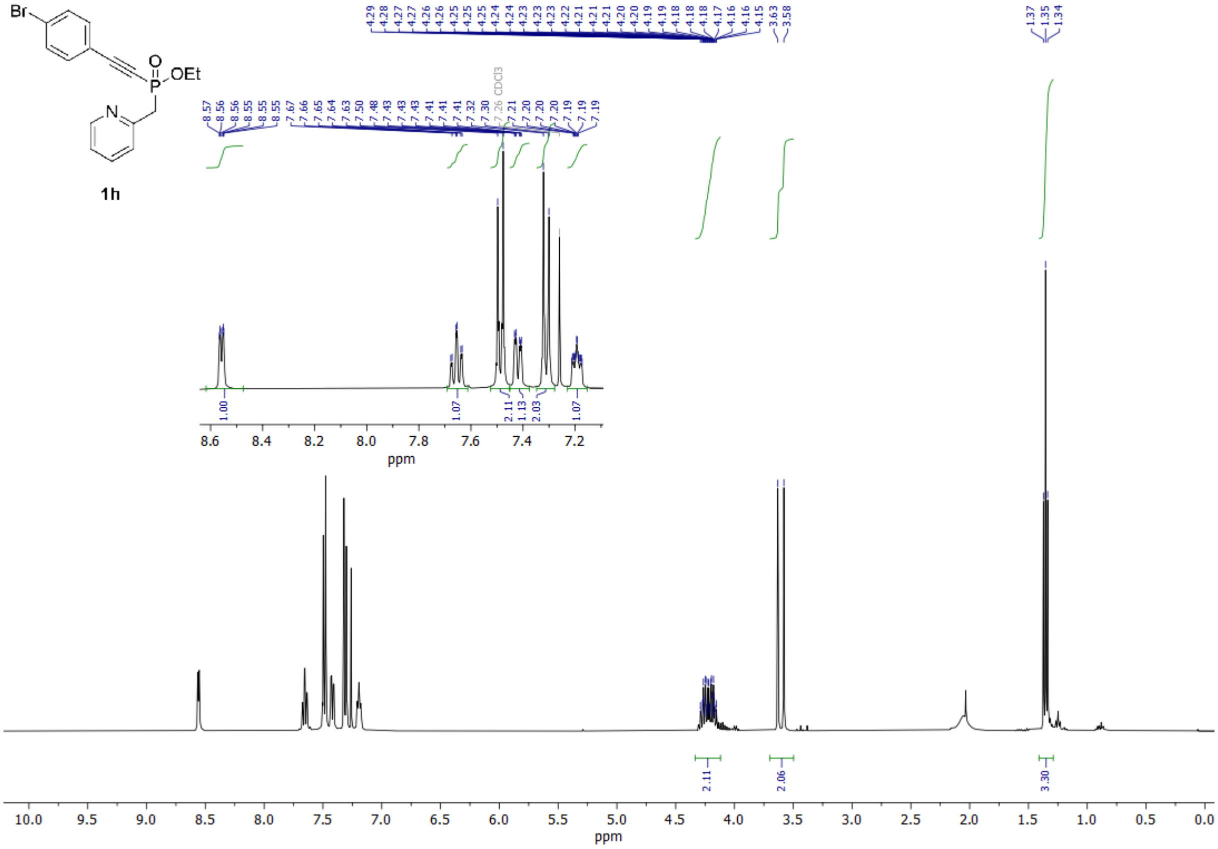

Figure S22 *^{1}H NMR spectrum of **1h** (400 MHz, CDCl$_3$).*

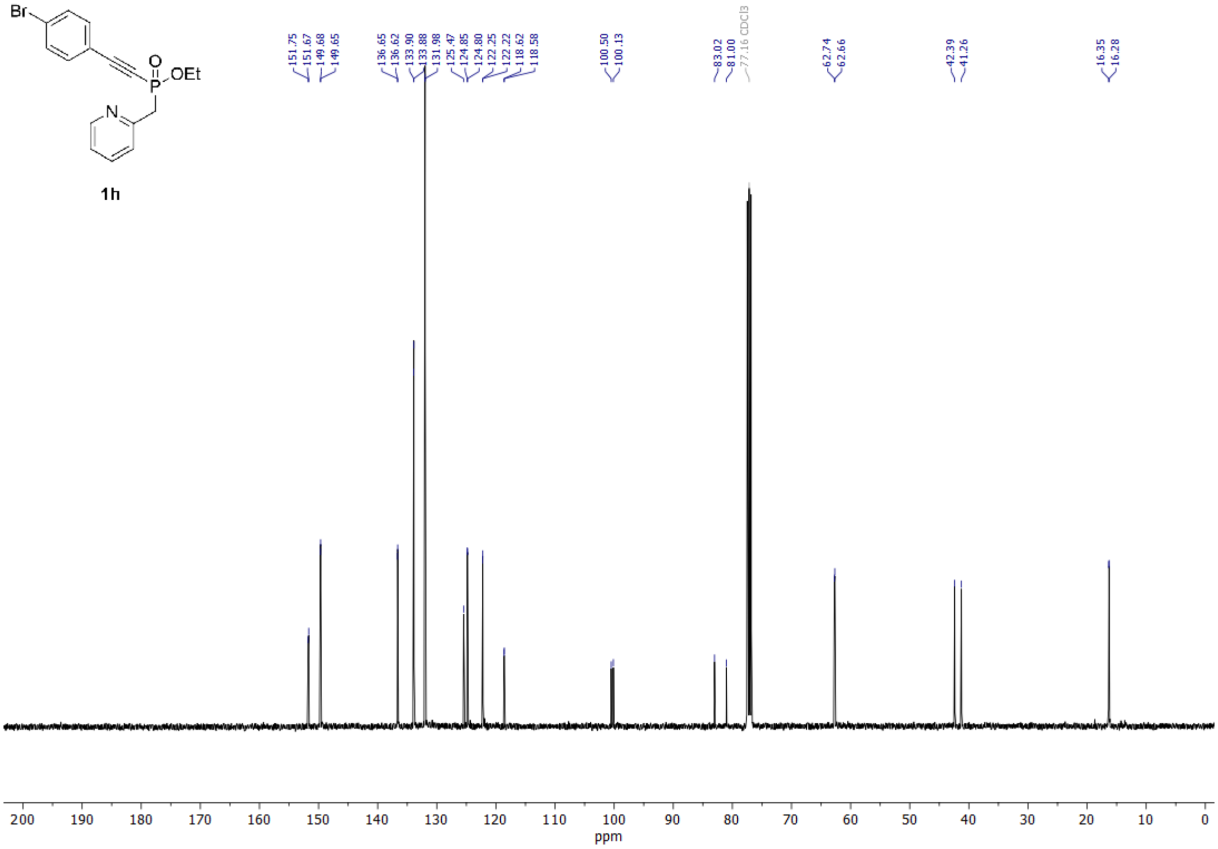

Figure S23 $^{13}C\ \{^1H\}$ *NMR spectrum of* ***1h*** *(101 MHz, $CDCl_3$).*

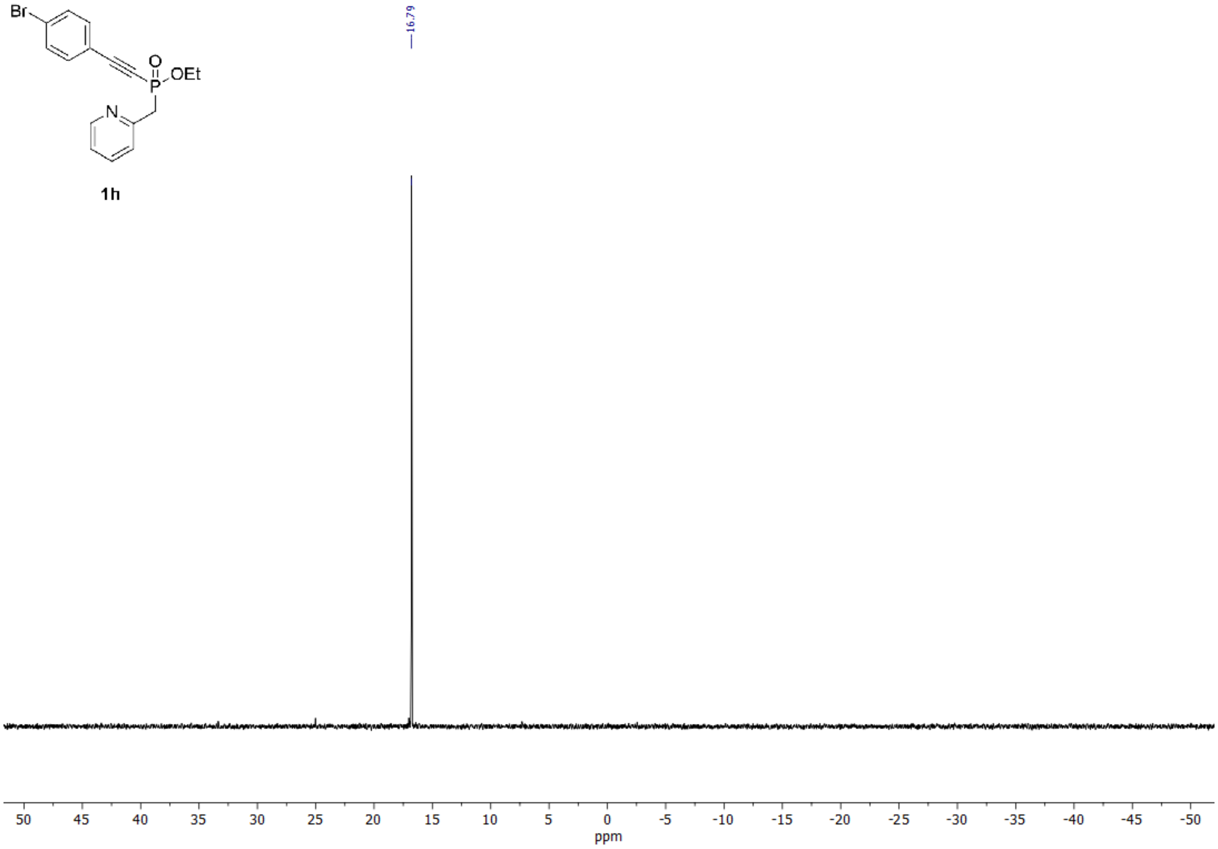

Figure S24 $^{31}P\ \{^1H\}$ *NMR spectrum of* ***1h*** *(162 MHz, $CDCl_3$).*

1h

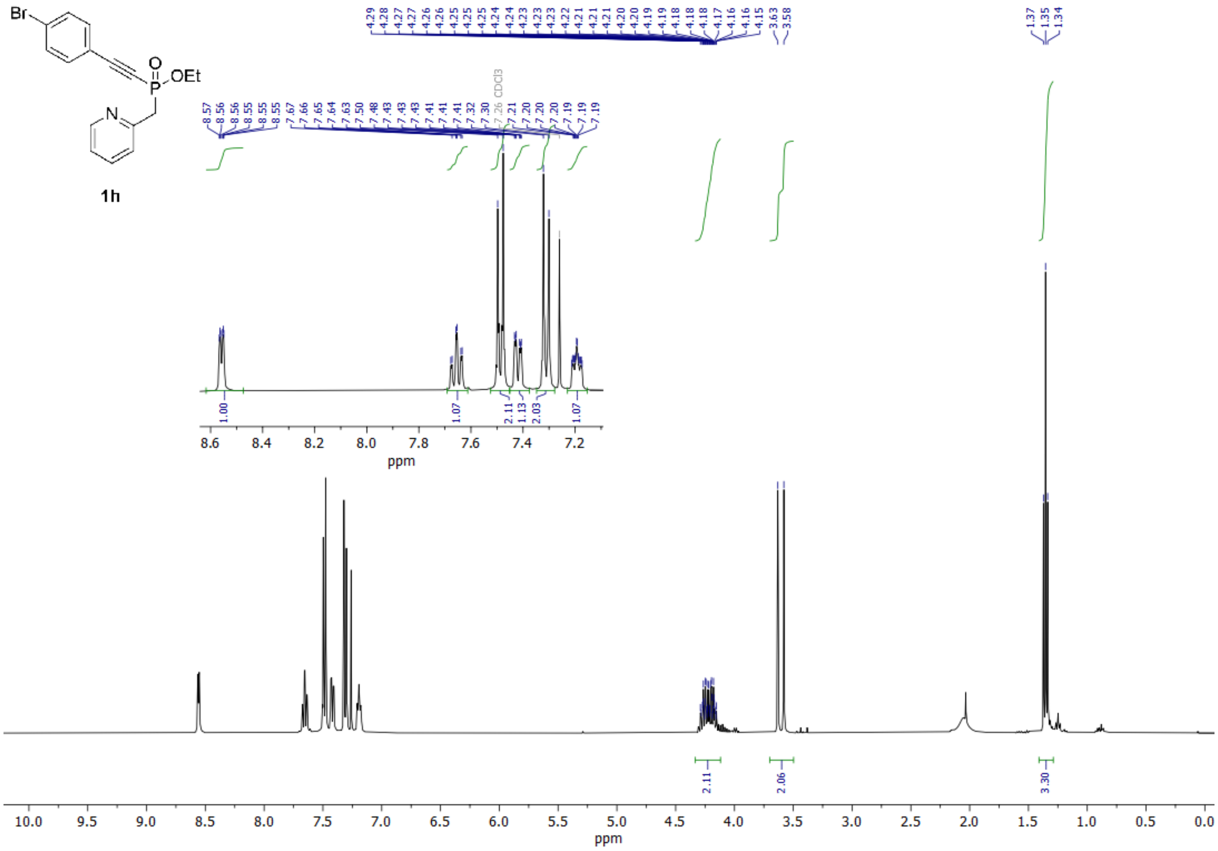

Figure S25 *^{1}H NMR spectrum of **1h** (400 MHz, CDCl$_3$).*

1i

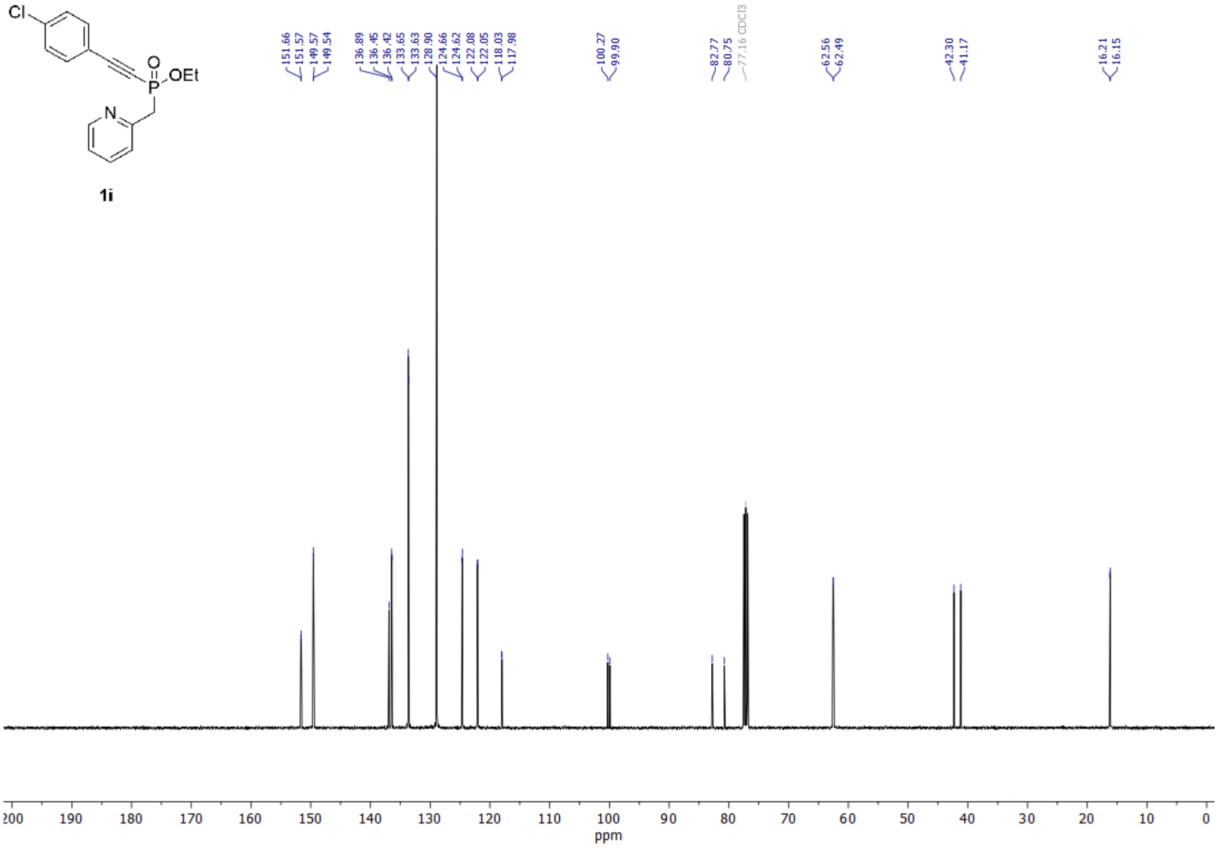

Figure S26 *^{13}C {^{1}H} NMR spectrum of **1i** (101 MHz, CDCl$_3$).*

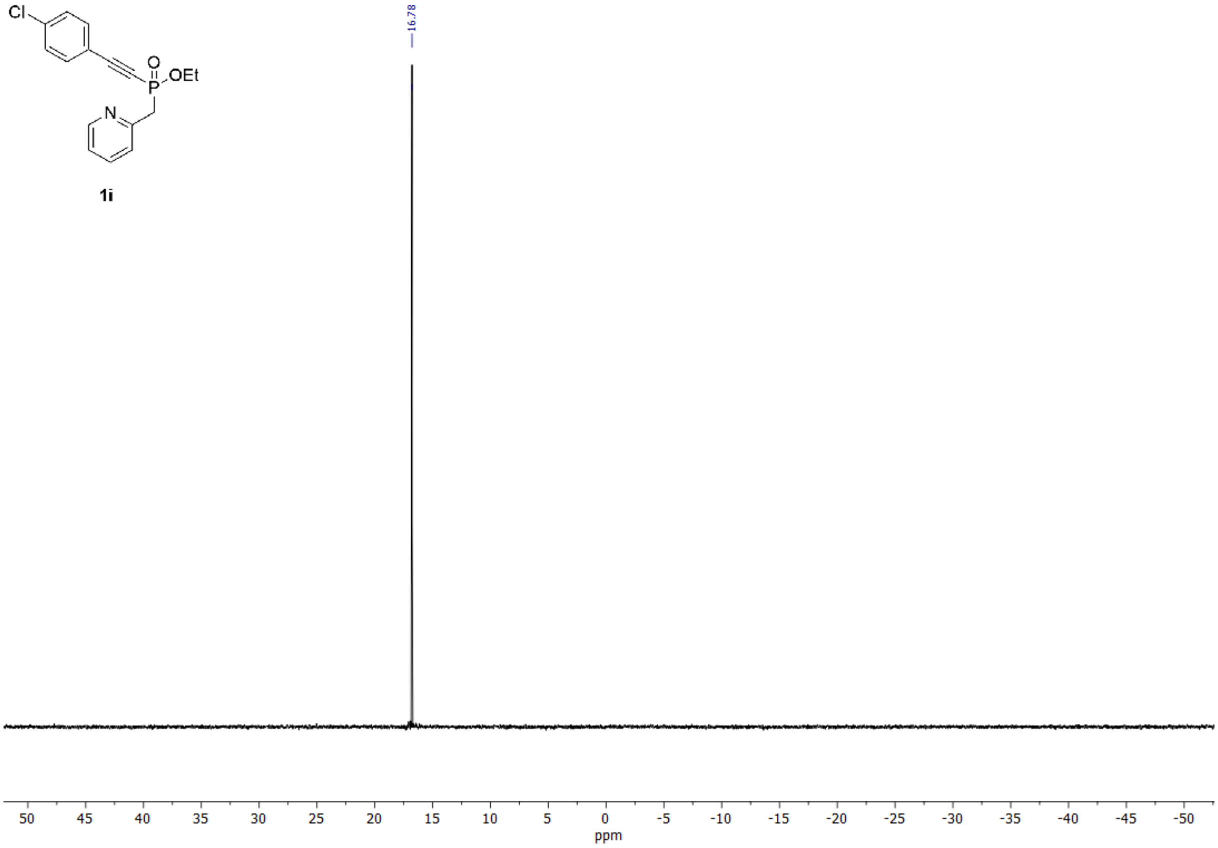

Figure S27 $^{31}P\ \{^{1}H\}$ *NMR spectrum of* ***1i*** *(162 MHz, $CDCl_3$).*

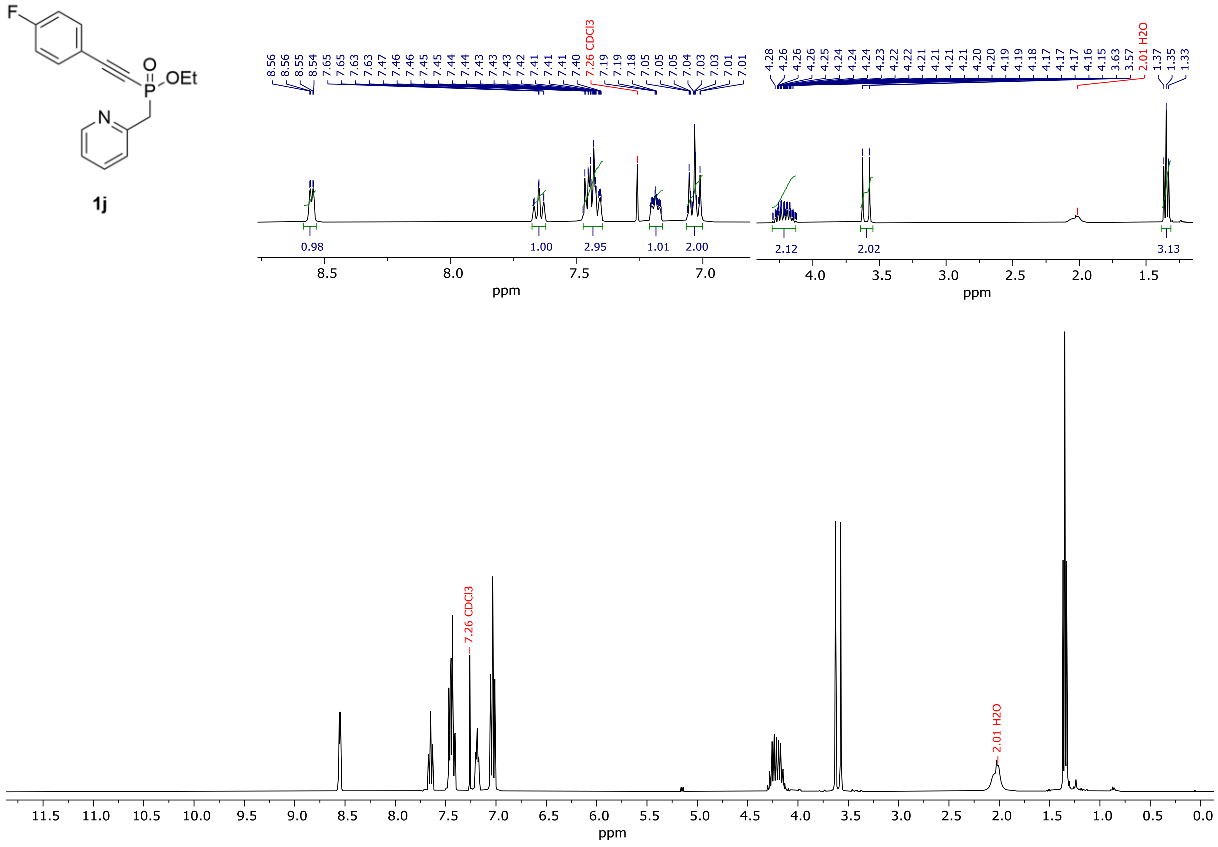

Figure S28 ^{1}H *NMR spectrum of* ***1j*** *(400 MHz, $CDCl_3$).*

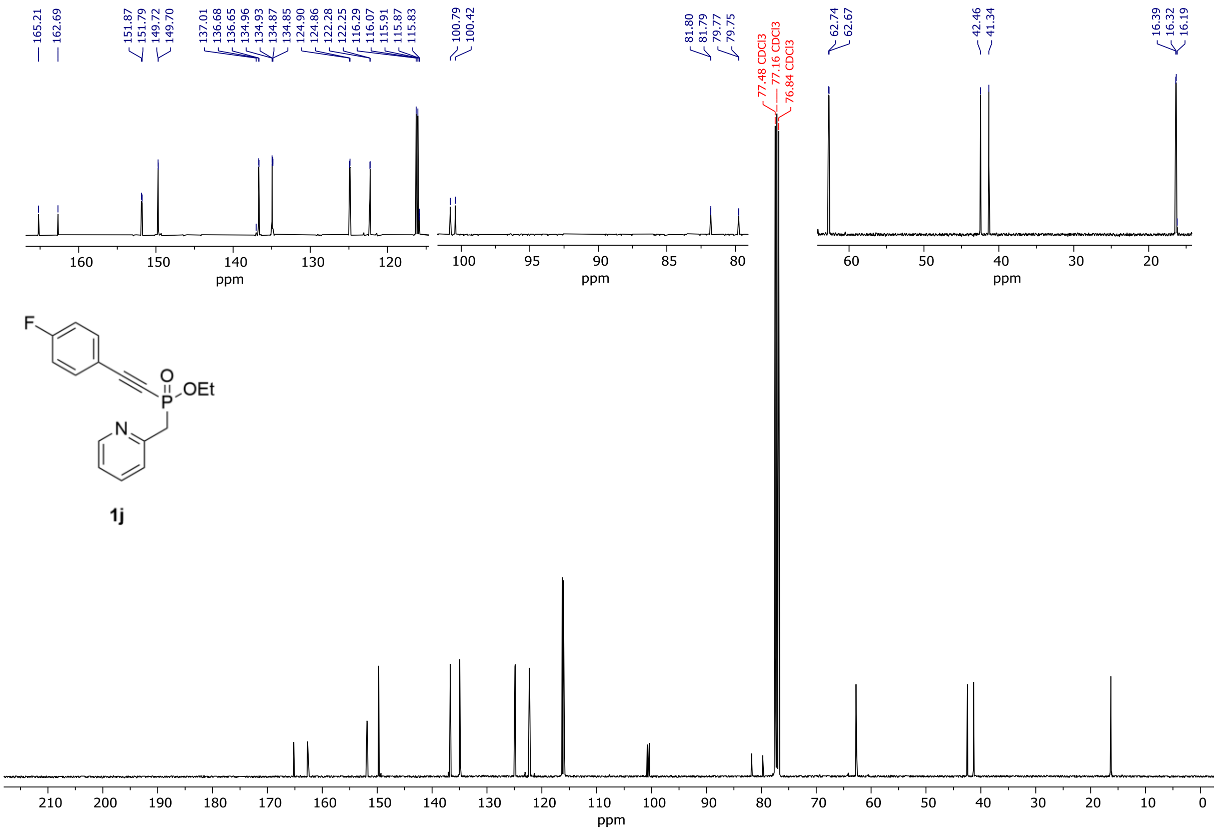

1j

Figure S29 *^{13}C {^1H} NMR spectrum of **1j** (101 MHz, $CDCl_3$).*

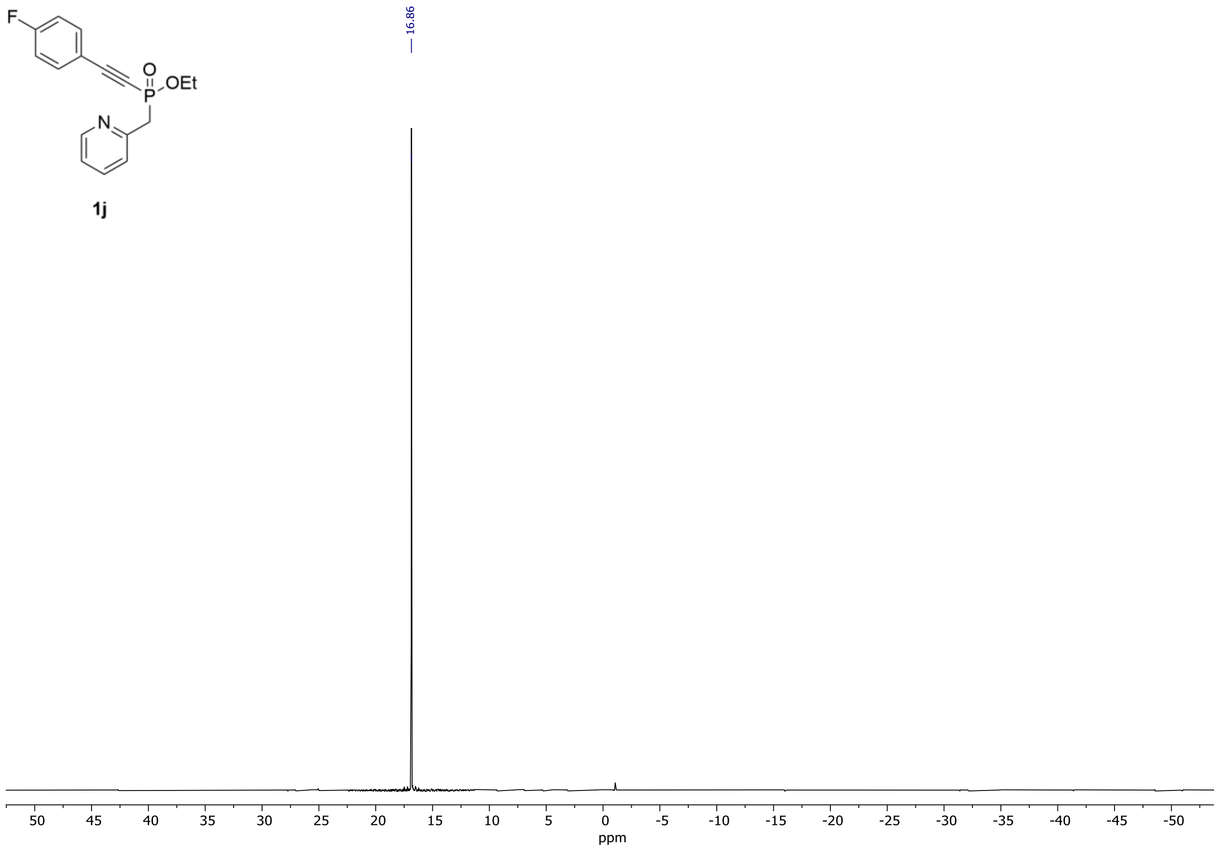

1j

Figure S30 *^{31}P {^1H} NMR spectrum of **1j** (162 MHz, $CDCl_3$).*

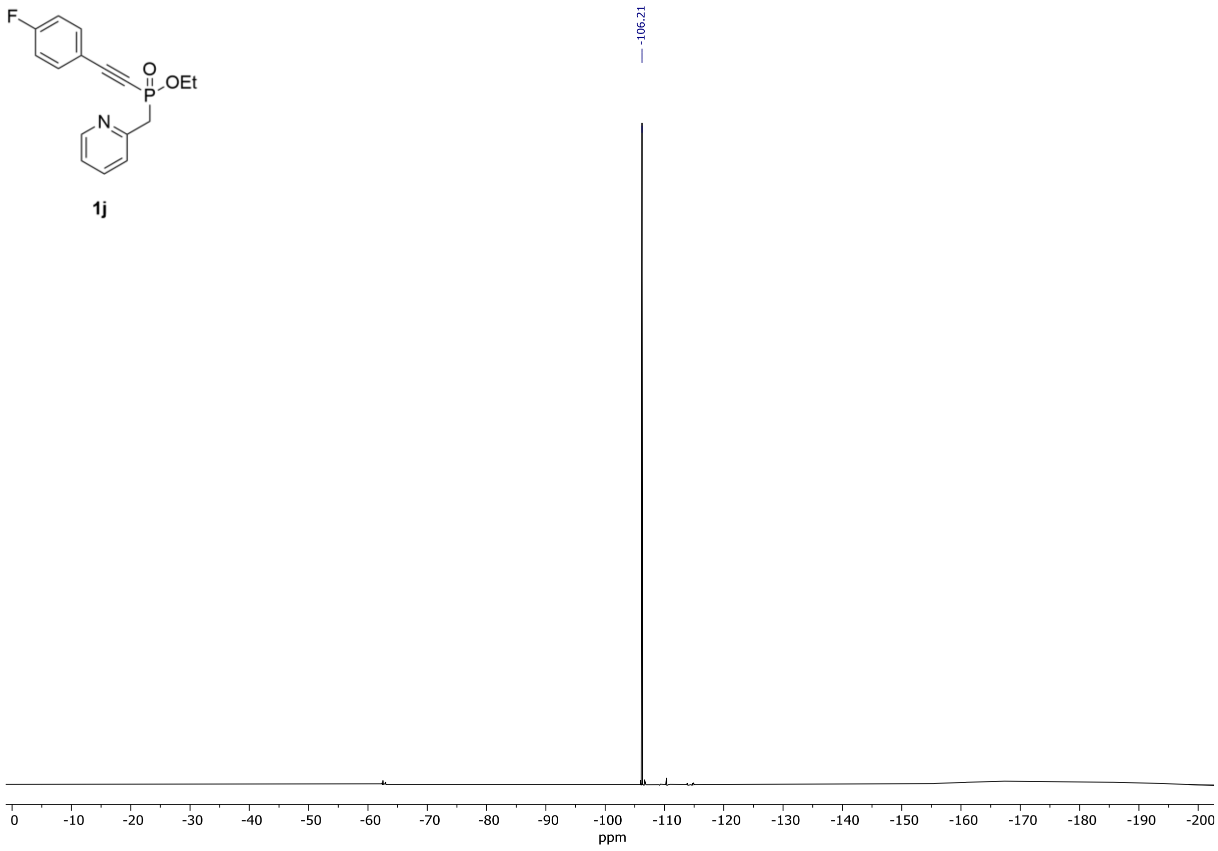

Figure S31 ^{19}F {^{1}H} NMR spectrum of **1j** (376 MHz, $CDCl_3$).

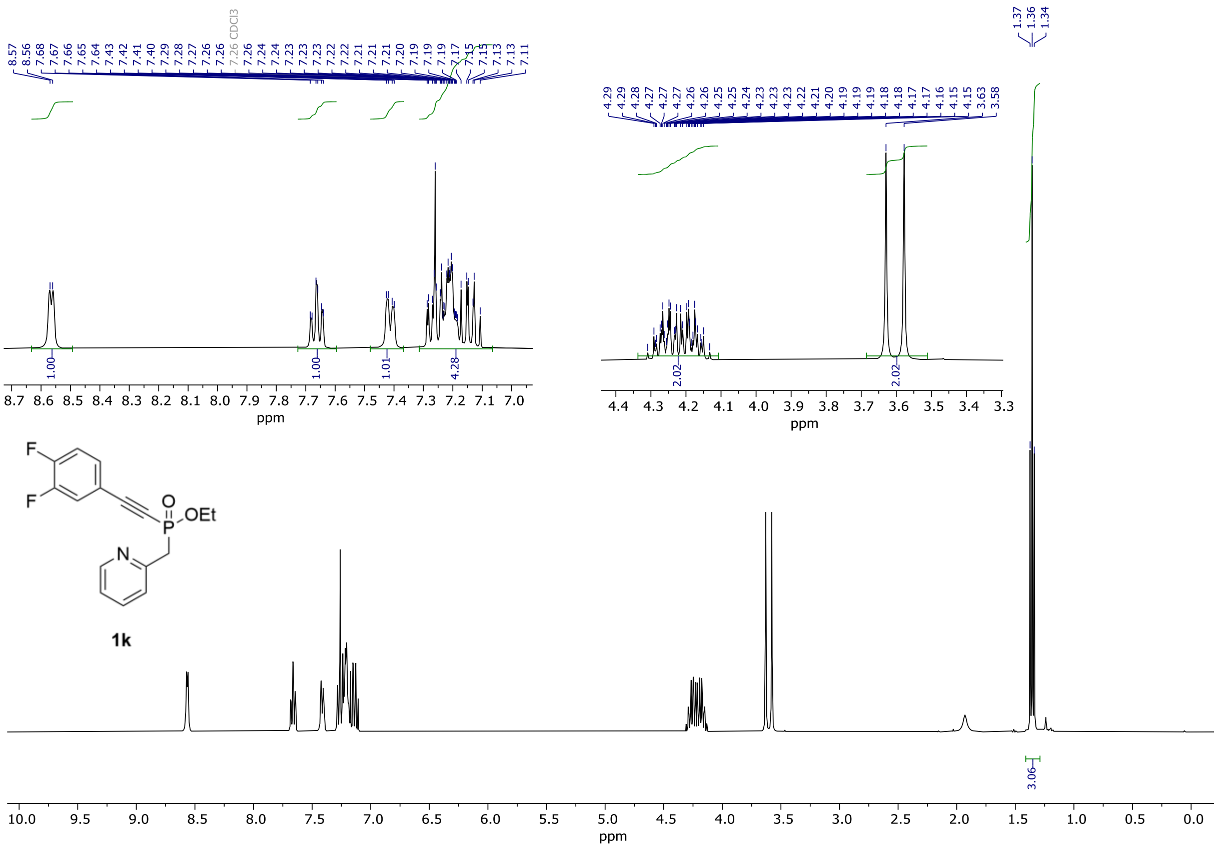

Figure S32 ^{1}H NMR spectrum of **1k** (400 MHz, $CDCl_3$).

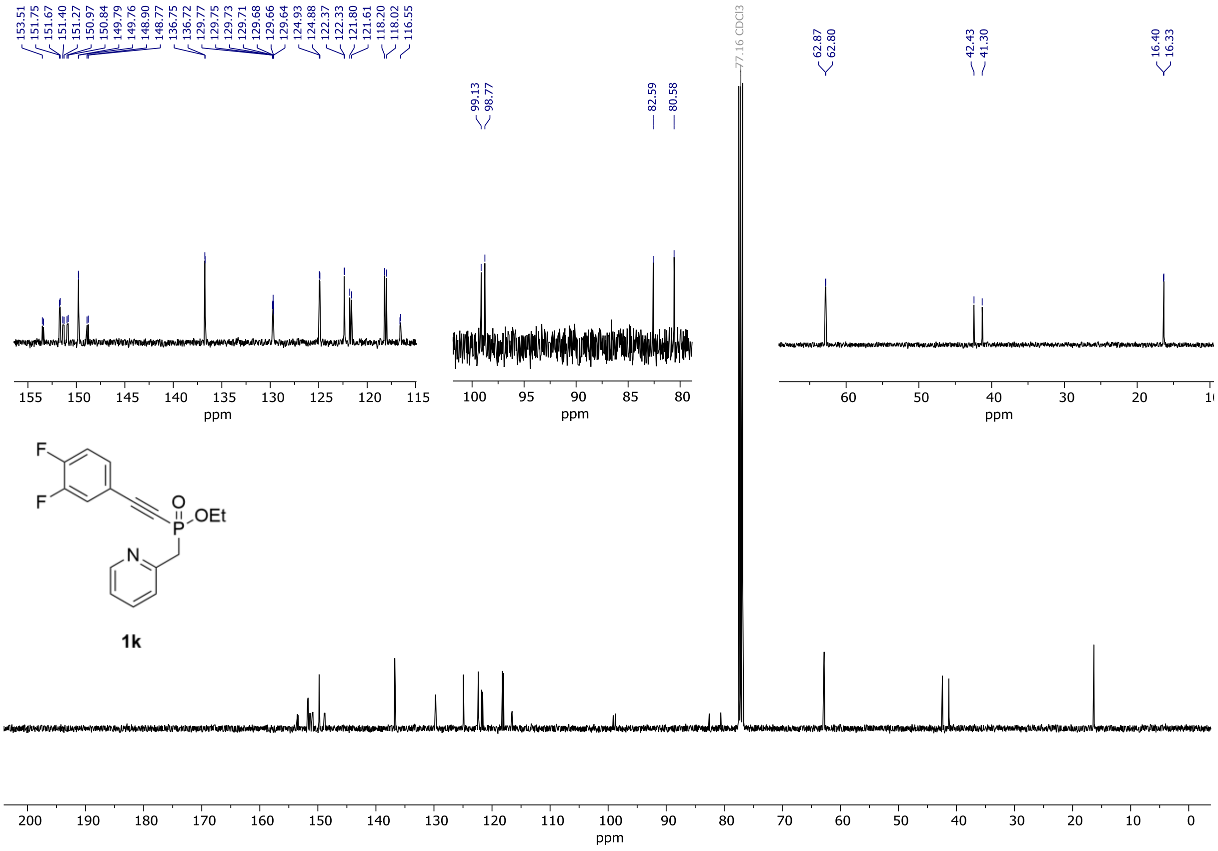

Figure S33 ^{13}C {^{1}H} NMR spectrum of **1k** (101 MHz, $CDCl_3$).

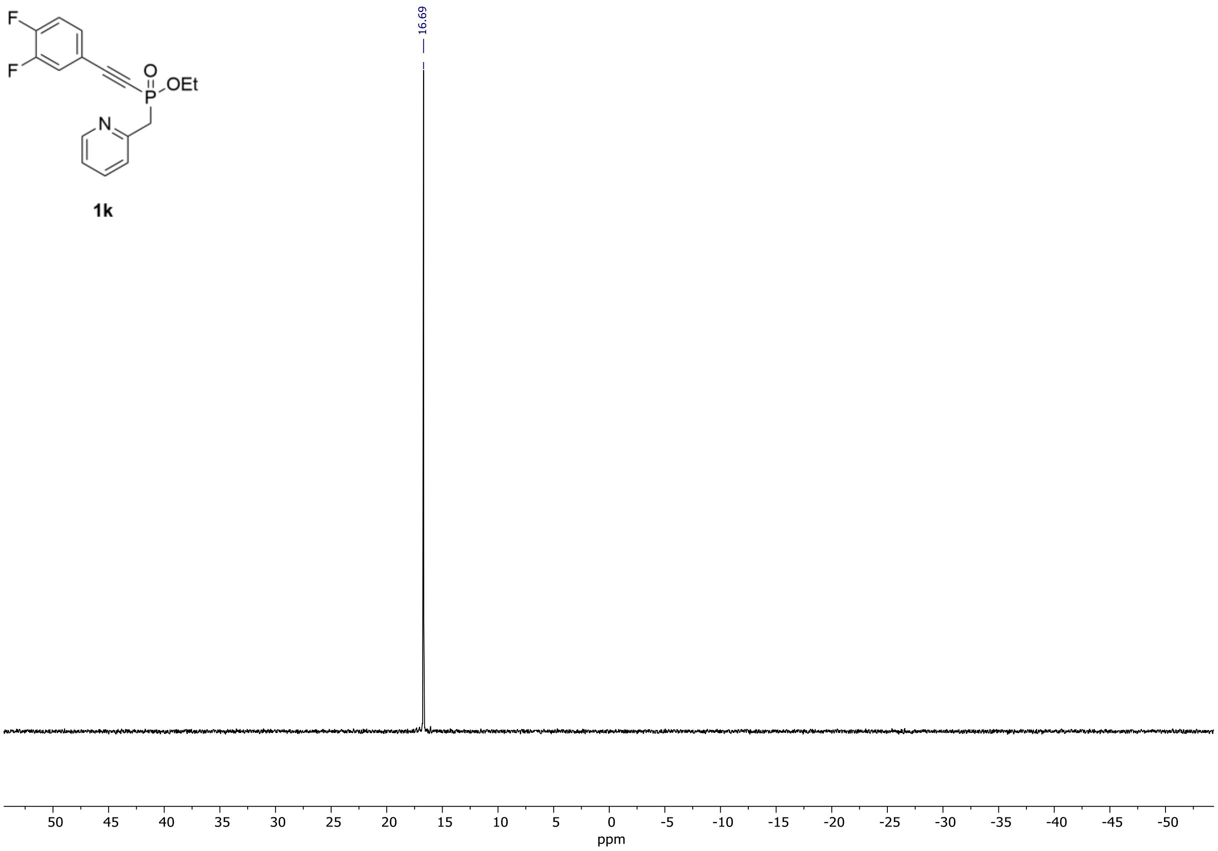

Figure S34 ^{31}P {^{1}H} NMR spectrum of **1k** (162 MHz, $CDCl_3$).

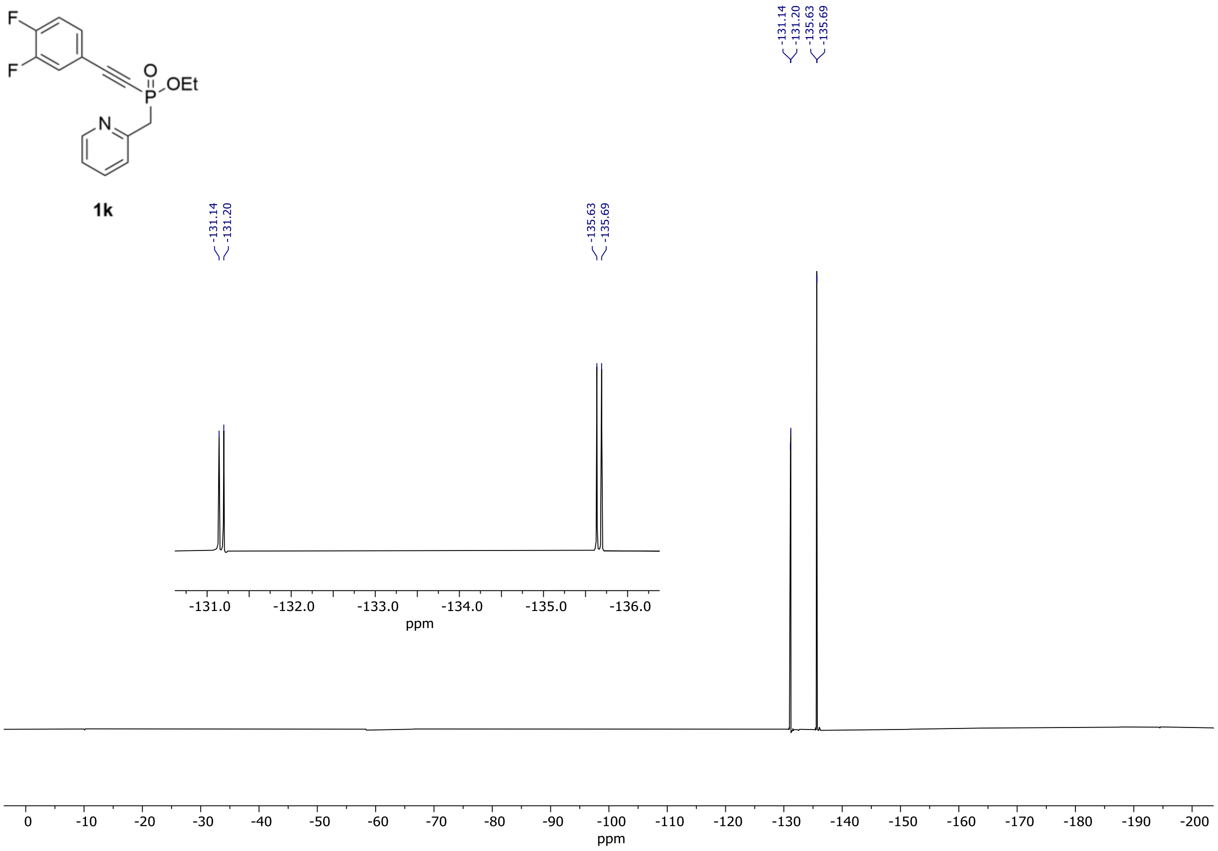

Figure S35 *^{19}F {^{1}H} NMR spectrum of* ***1k*** *(376 MHz, $CDCl_3$).*

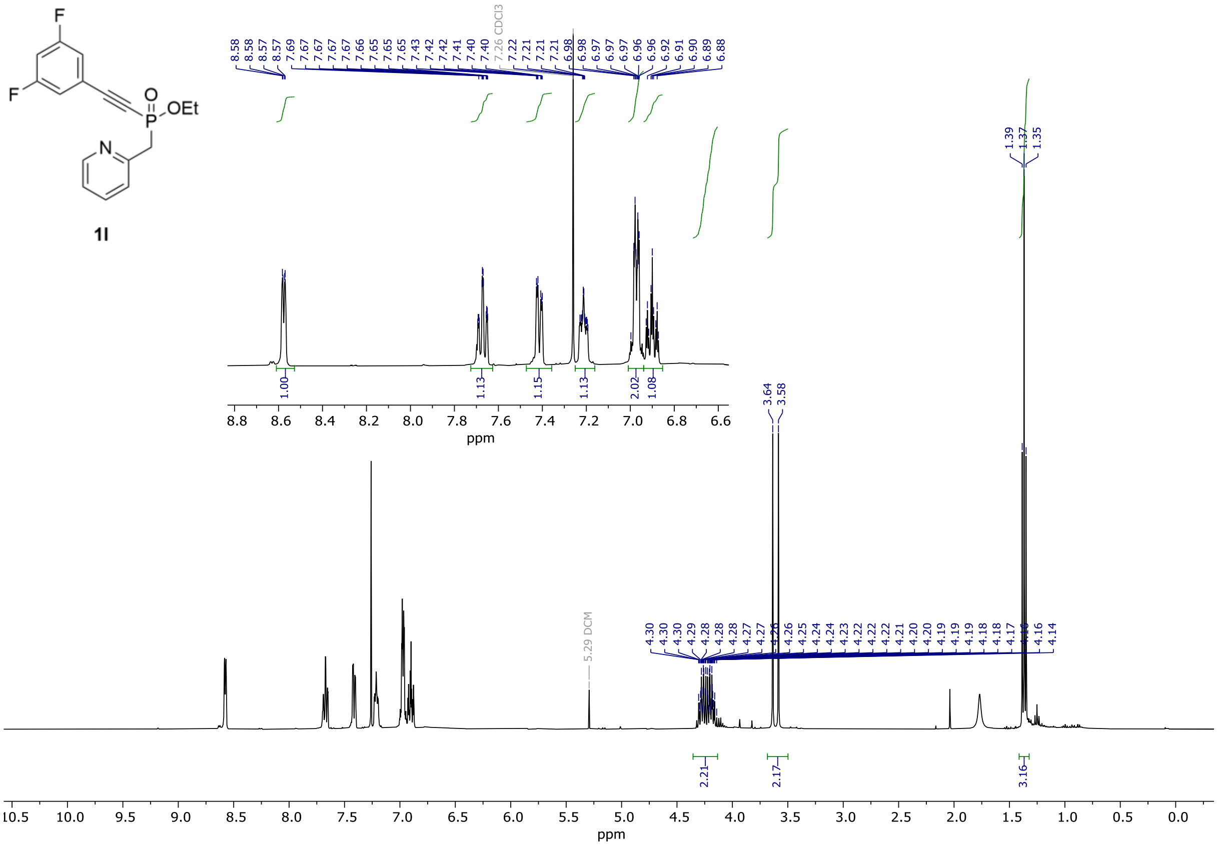

Figure S36 *^{1}H NMR spectrum of* ***1l*** *(400 MHz, $CDCl_3$).*

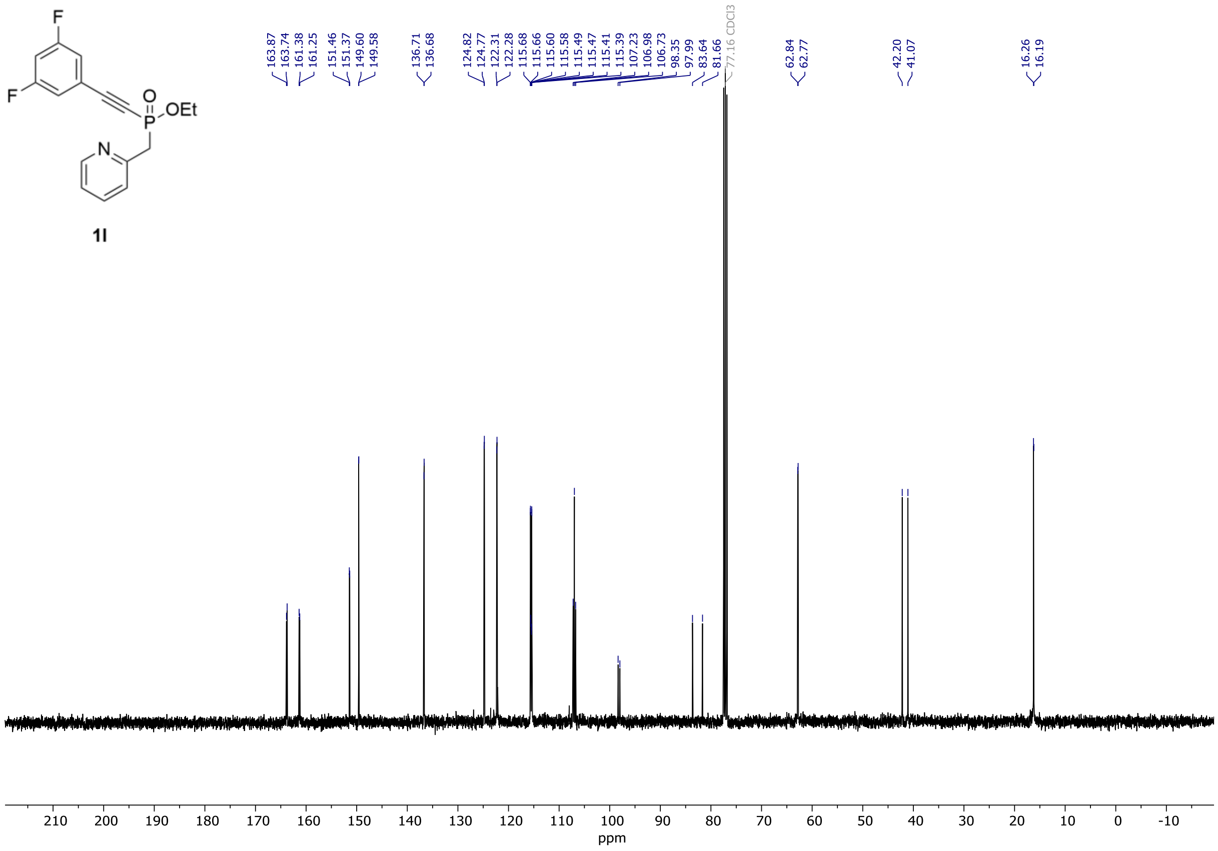

1l

Figure S37 *^{13}C {^{1}H} NMR spectrum of **1l** (101 MHz, $CDCl_3$).*

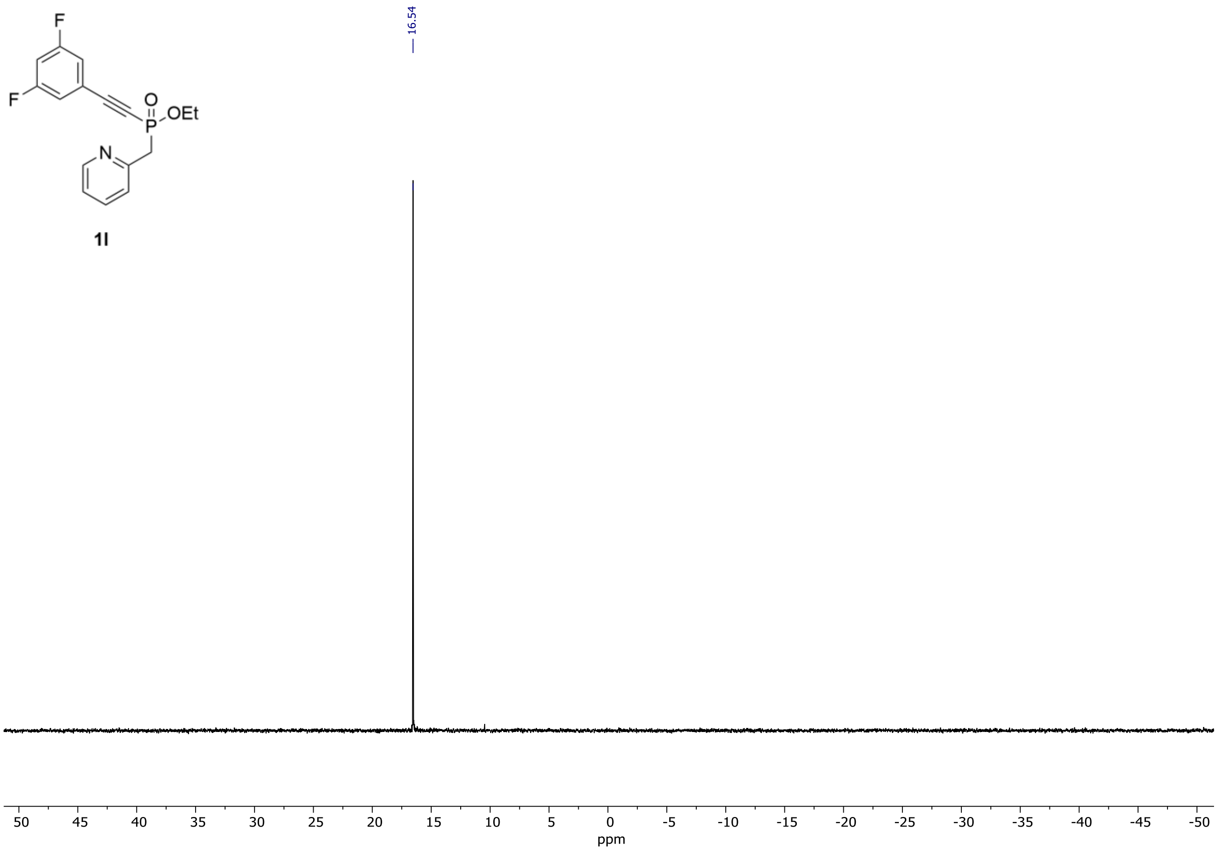

1l

Figure S38 *^{31}P {^{1}H} NMR spectrum of **1l** (162 MHz, $CDCl_3$).*

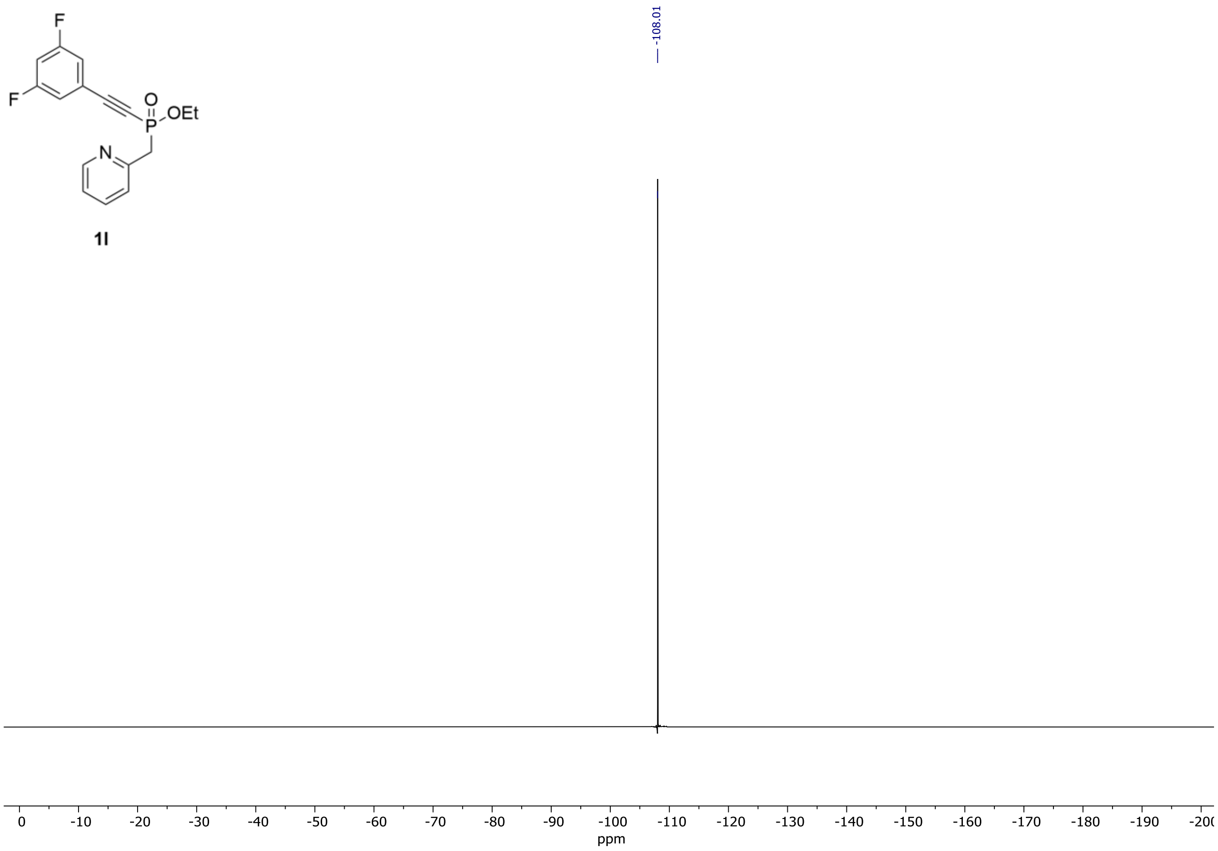

Figure S39 *^{19}F {^{1}H} NMR spectrum of **1l** (376 MHz, $CDCl_3$).*

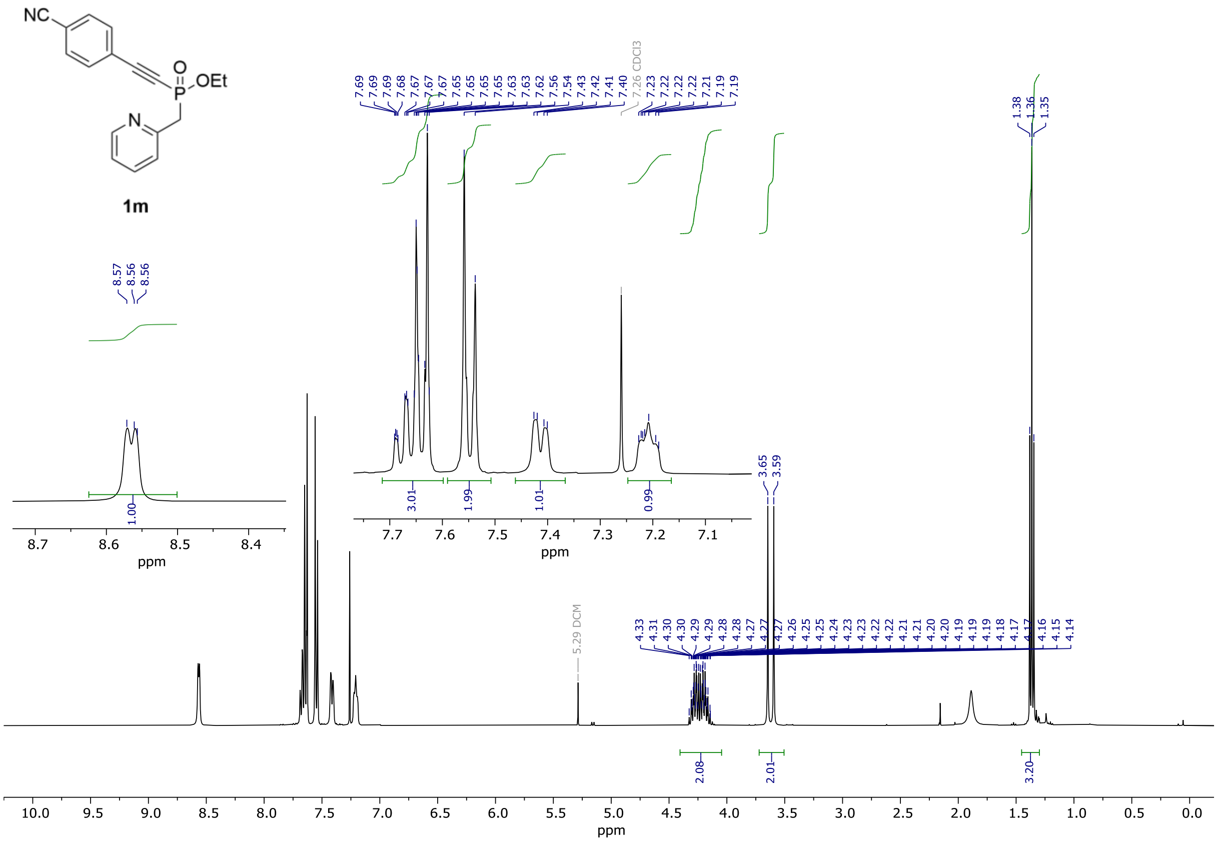

Figure S40 *^{1}H NMR spectrum of **1m** (400 MHz, $CDCl_3$).*

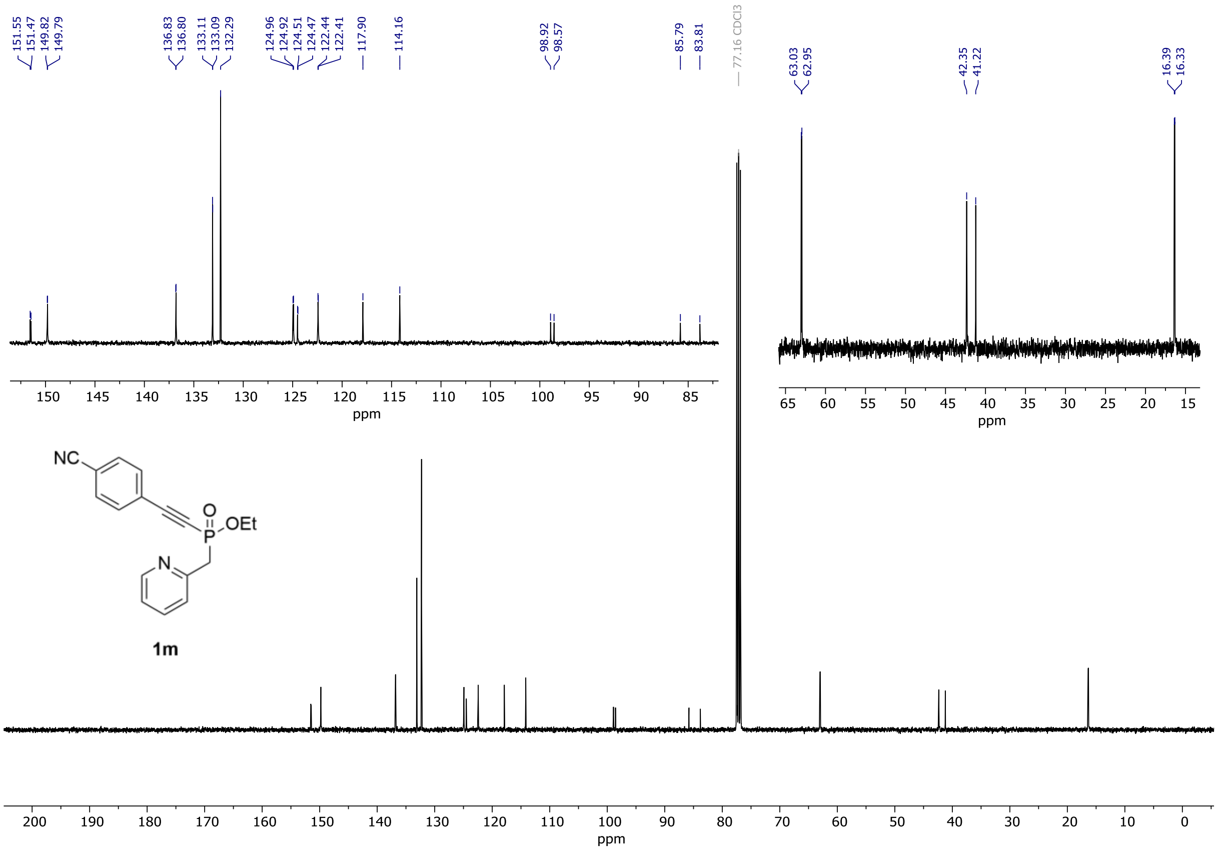

Figure S41 *^{13}C {^{1}H} NMR spectrum of* ***1m*** *(101 MHz, $CDCl_3$).*

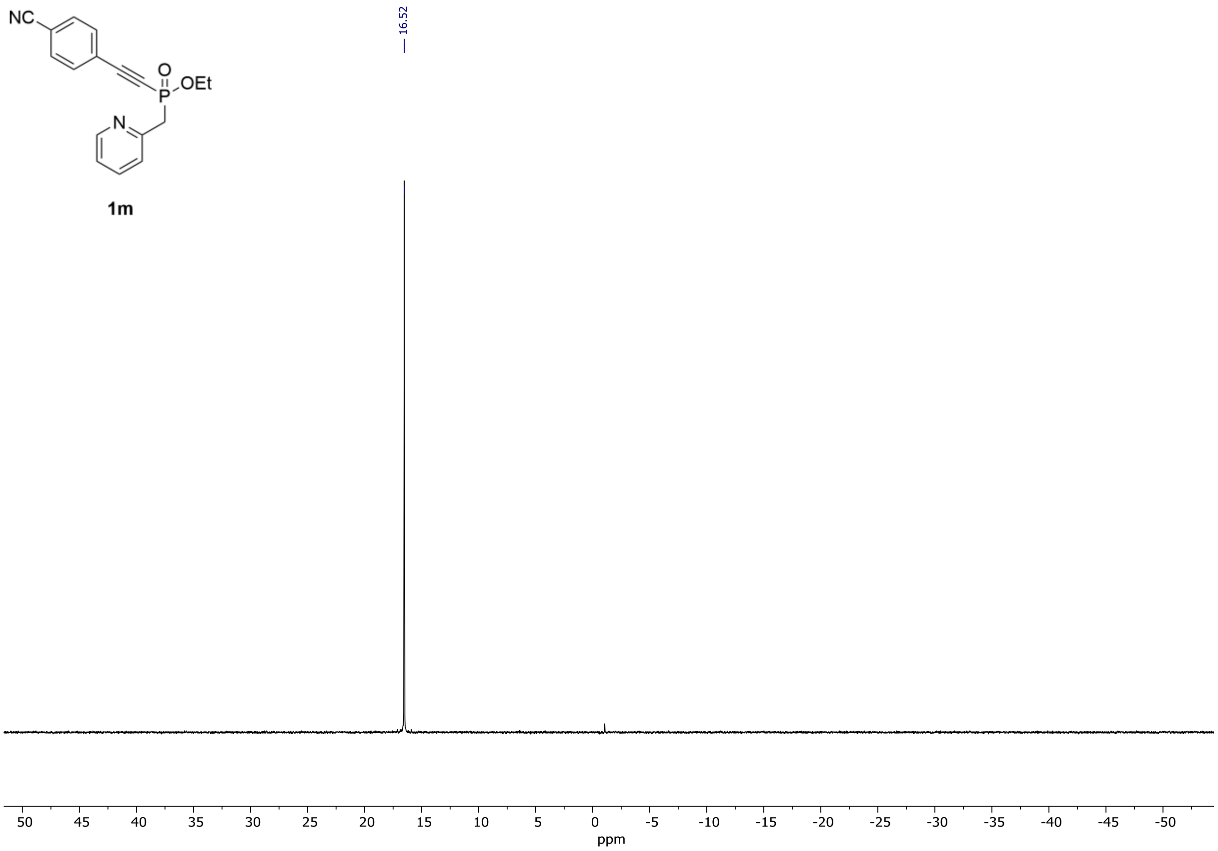

Figure S42 *^{31}P {^{1}H} NMR spectrum of* ***1m*** *(162 MHz, $CDCl_3$).*

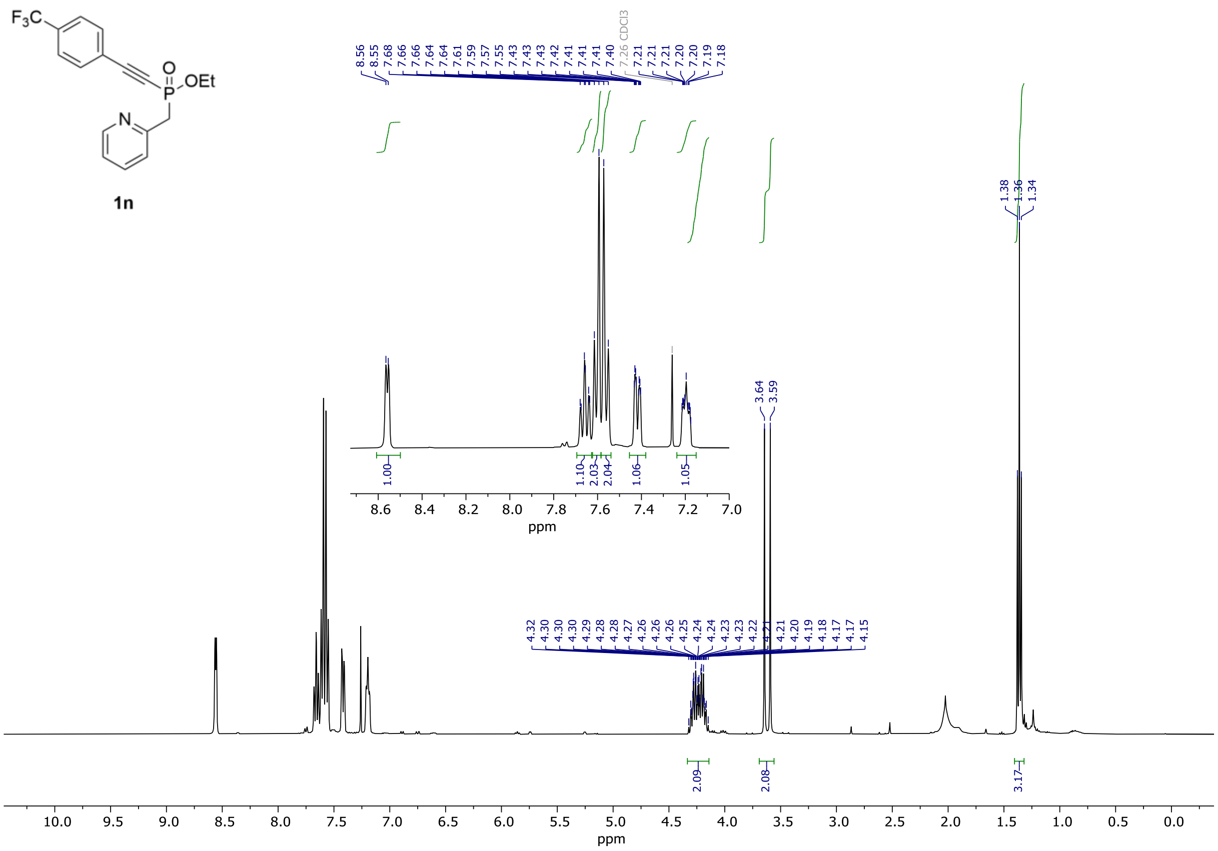

Figure S43 ^{1}*H NMR spectrum of* ***1n*** *(400 MHz, CDCl$_3$).*

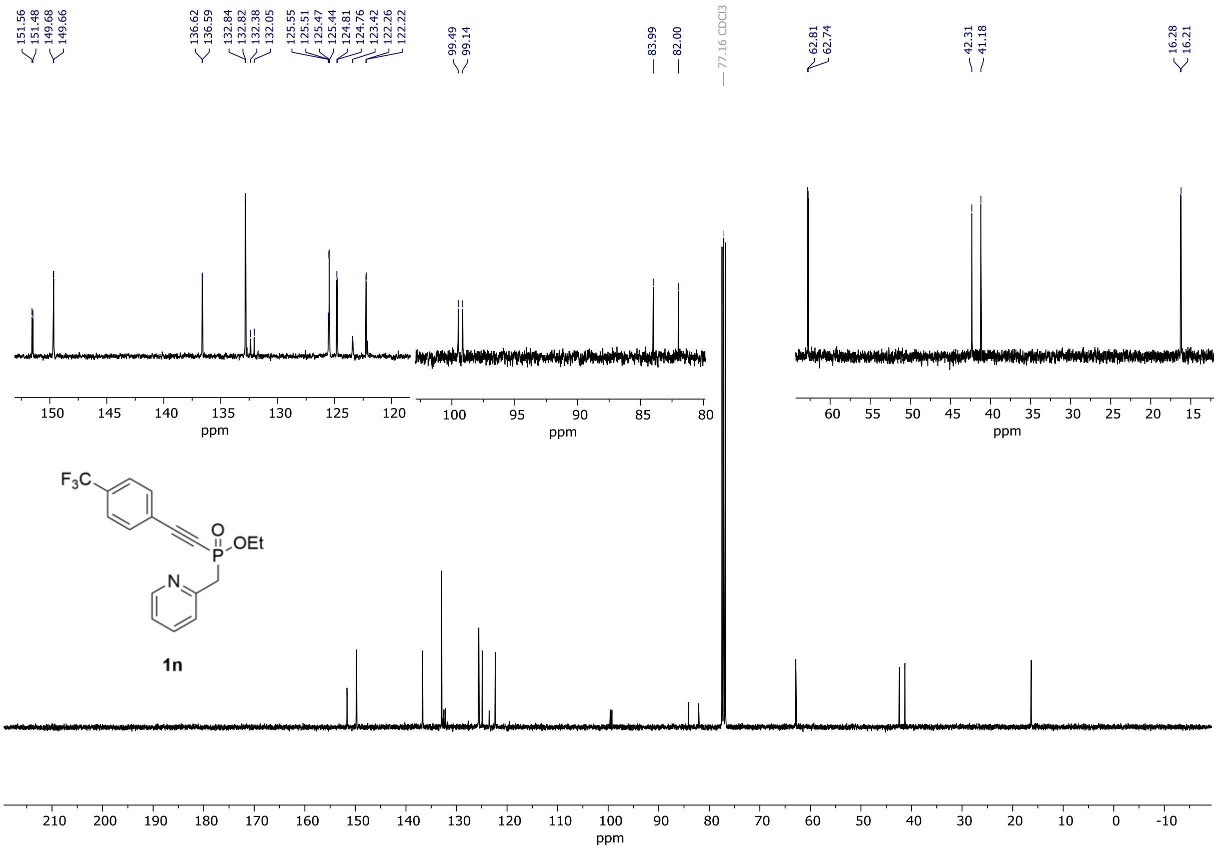

Figure S44 ^{13}*C {^{1}H} NMR spectrum of* ***1n*** *(101 MHz, CDCl$_3$).*

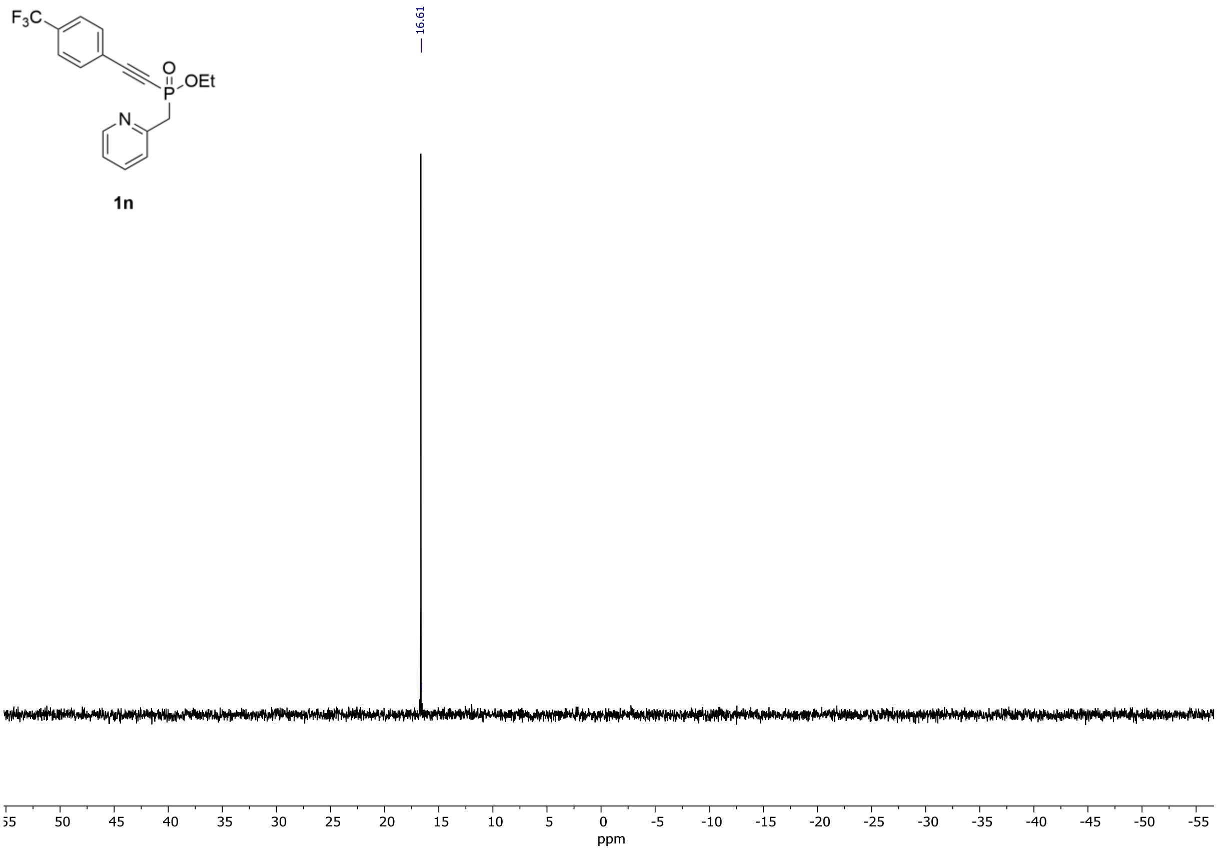

Figure S45 *^{31}P {^{1}H} NMR spectrum of **1n** (162 MHz, $CDCl_3$).*

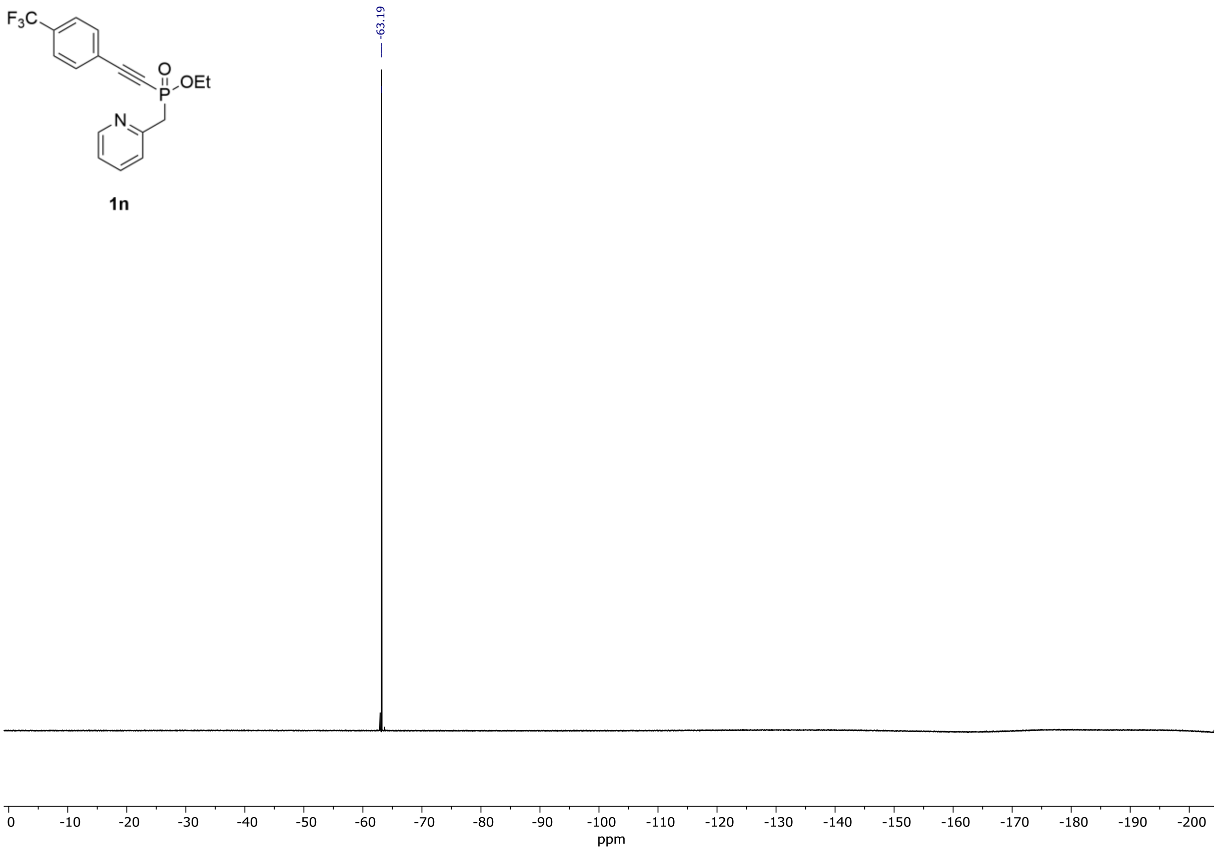

Figure S46 *^{19}F {^{1}H} NMR spectrum of **1n** (376 MHz, $CDCl_3$).*

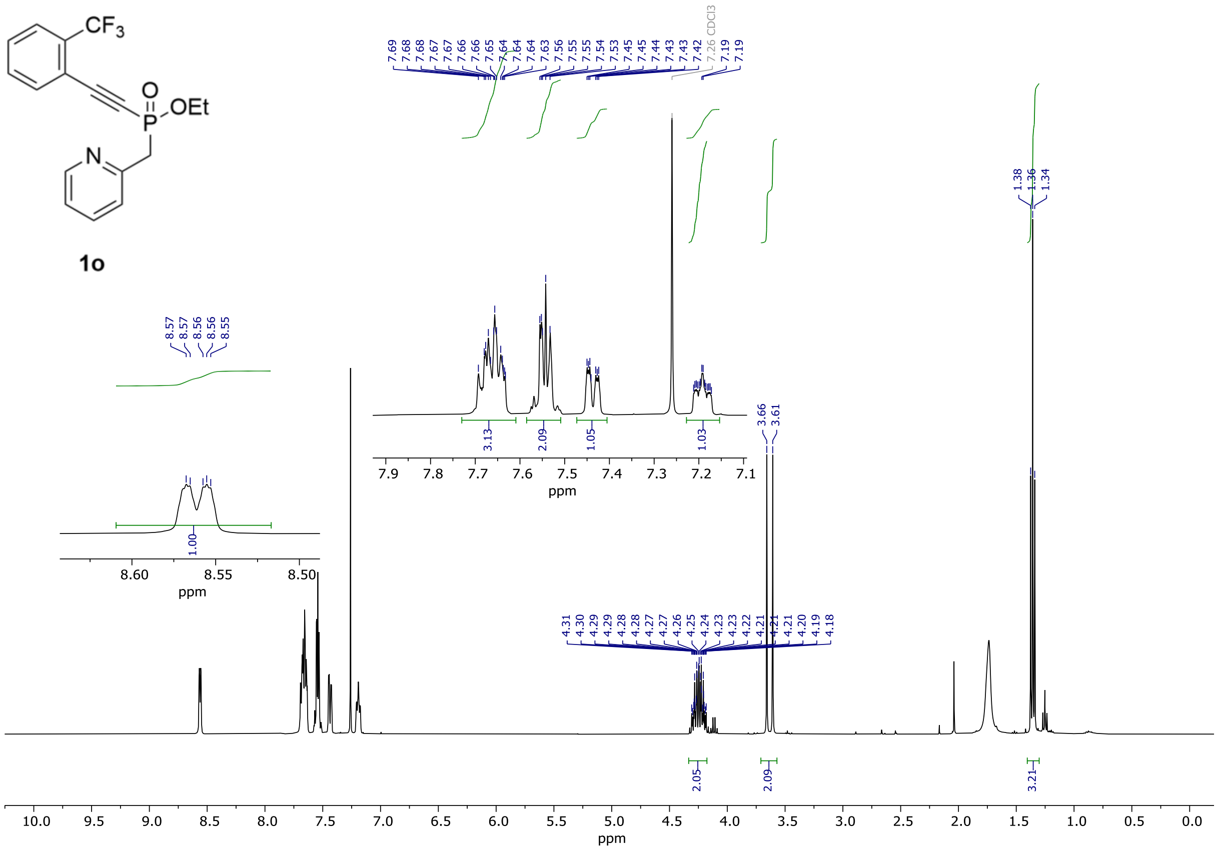

1o

Figure S47 *^{1}H NMR spectrum of **1o** (400 MHz, CDCl$_3$).*

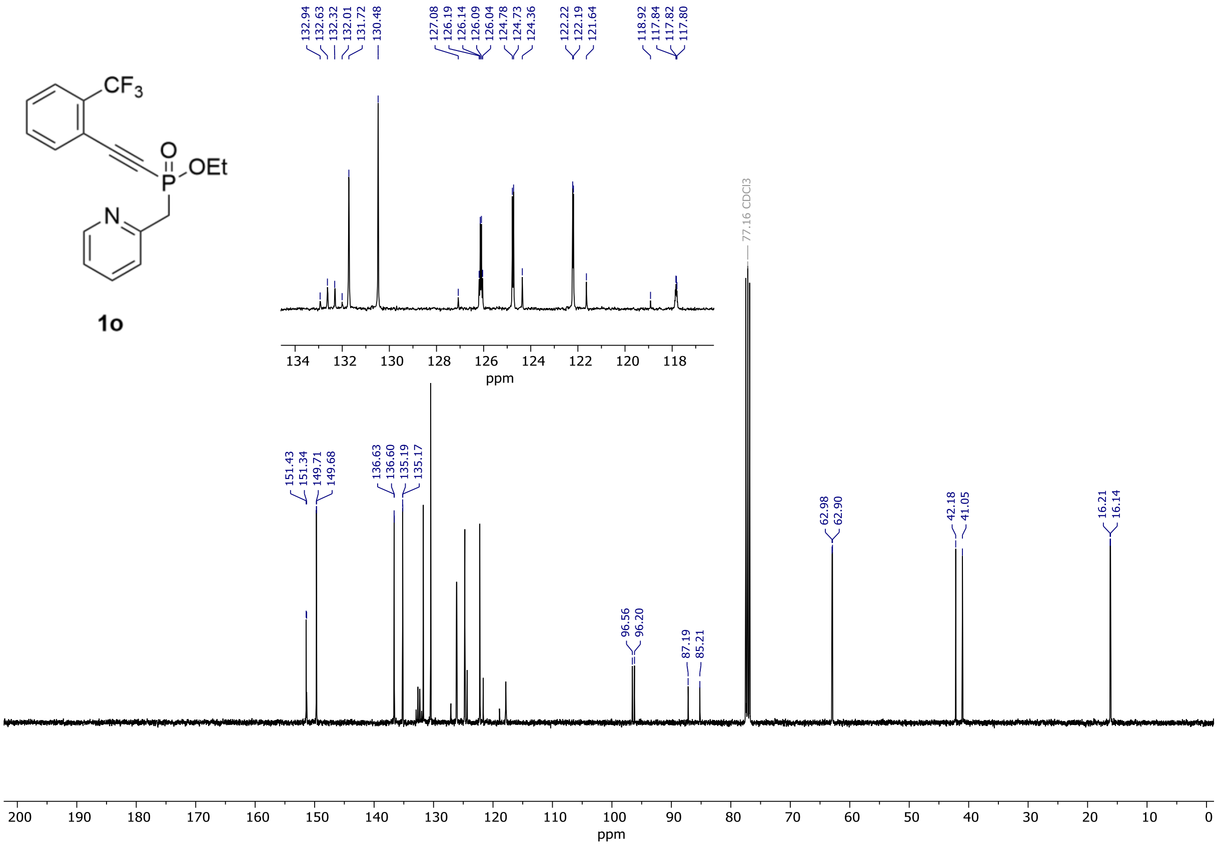

1o

Figure S48 *^{13}C {^{1}H} NMR spectrum of **1o** (101 MHz, CDCl$_3$).*

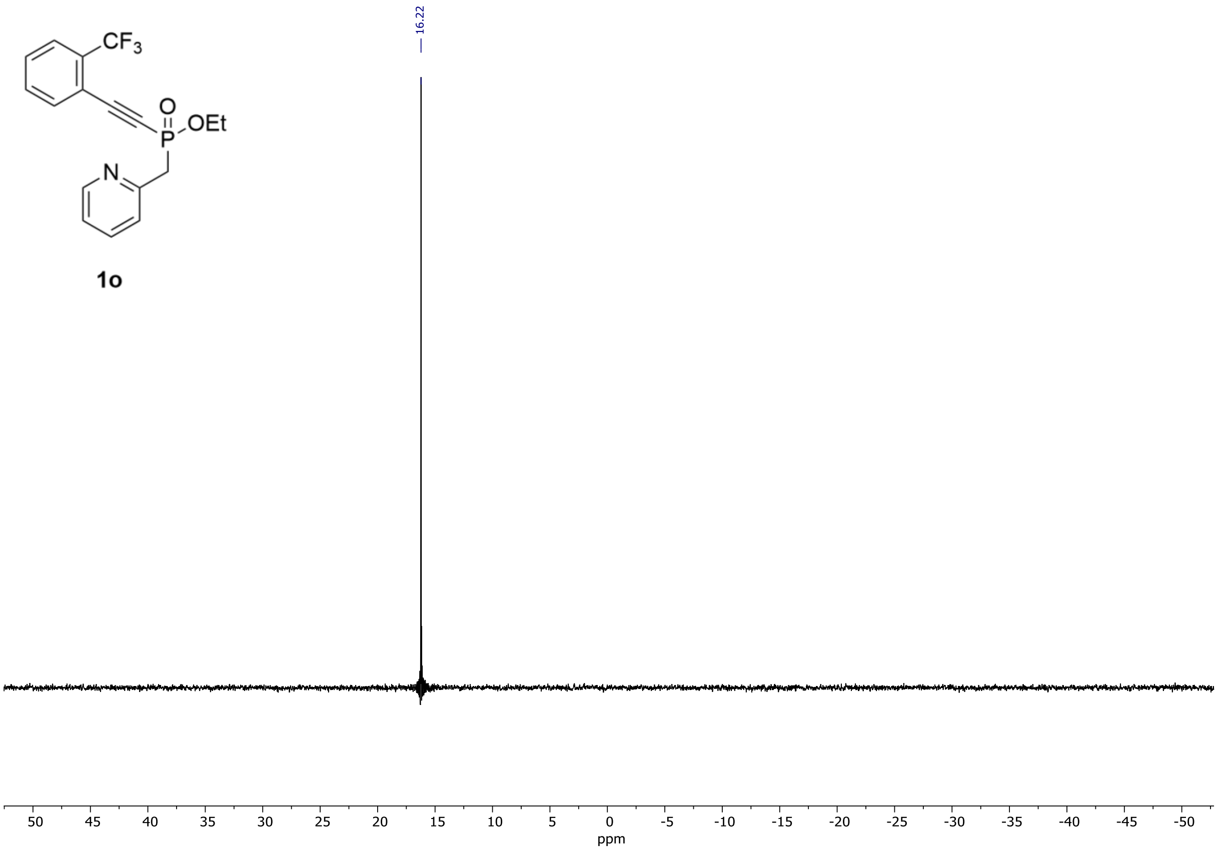

Figure S49 *^{31}P {^1H} NMR spectrum of **1o** (162 MHz, $CDCl_3$).*

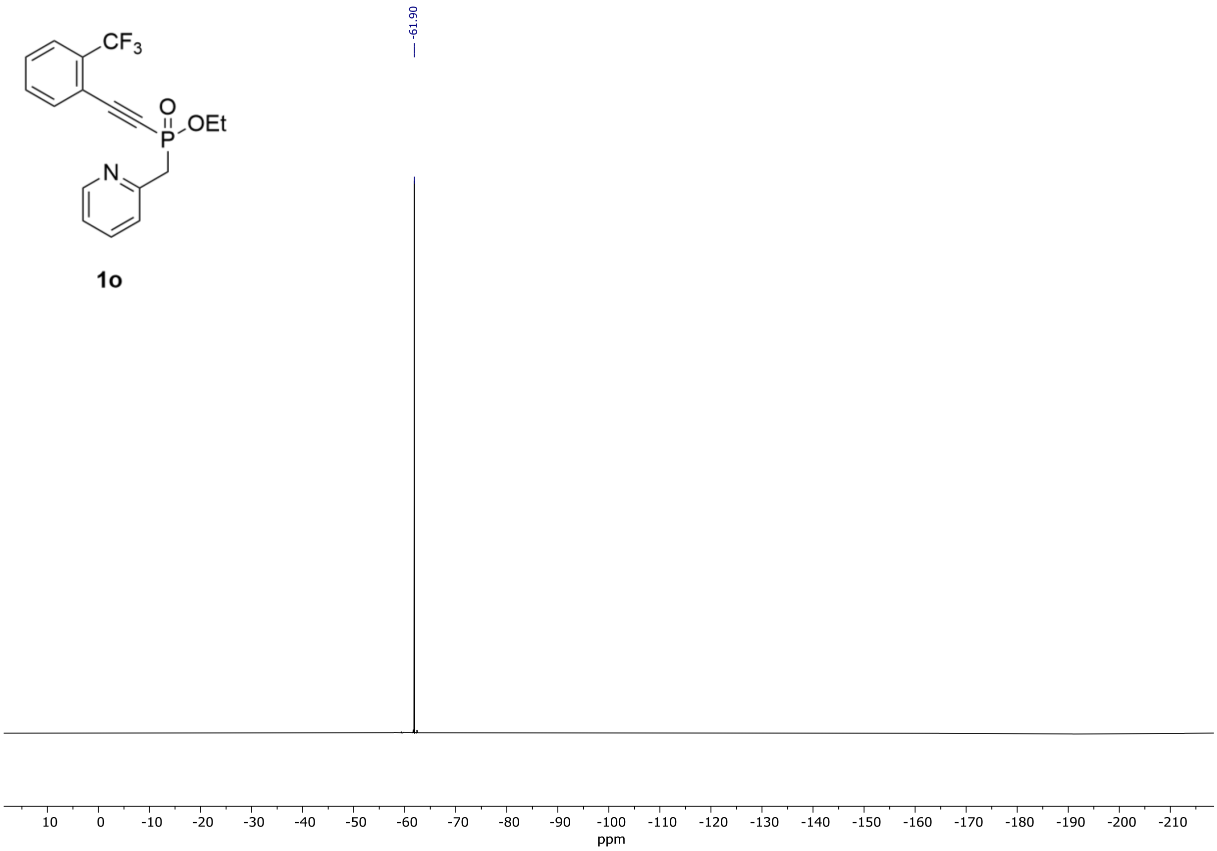

Figure S50 *^{19}F {^1H} NMR spectrum of **1o** (376 MHz, $CDCl_3$).*

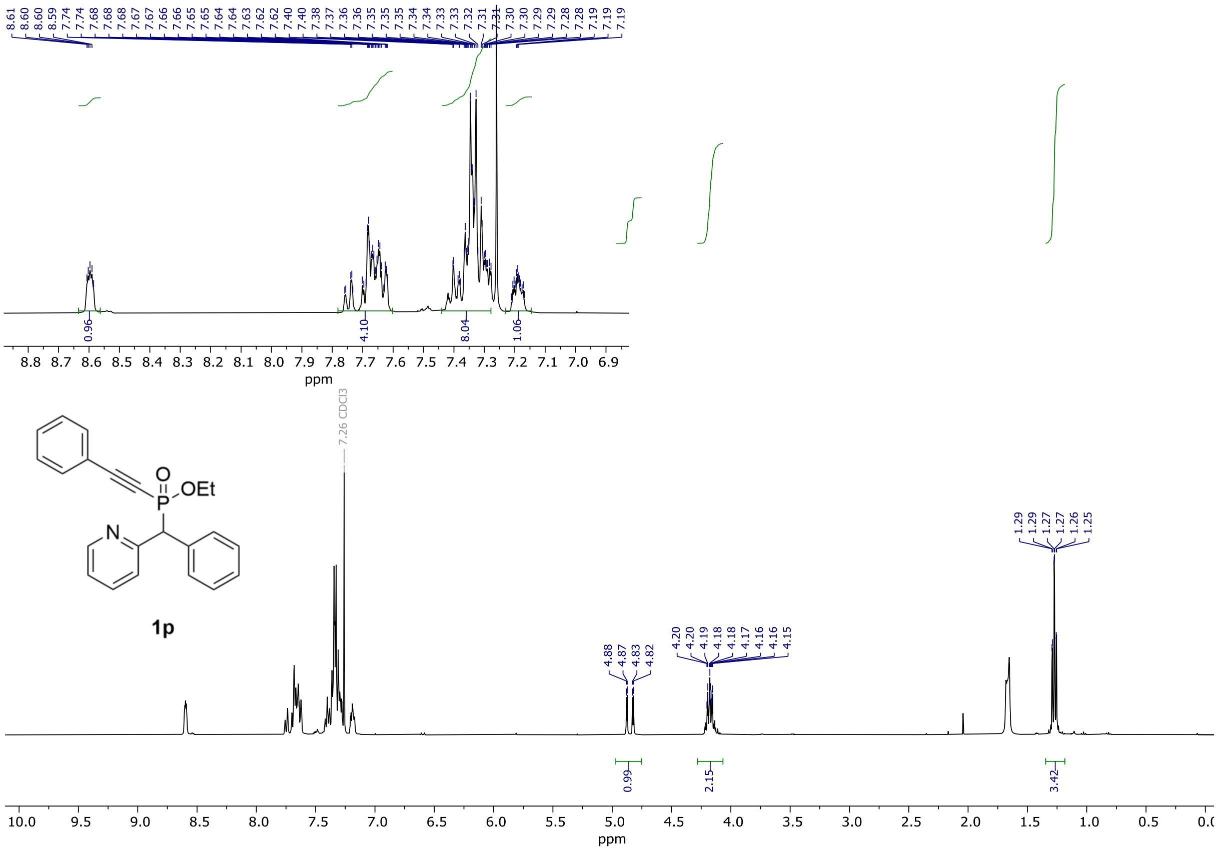

Figure S51 ^{1}H NMR spectrum of **1p** (400 MHz, $CDCl_3$).

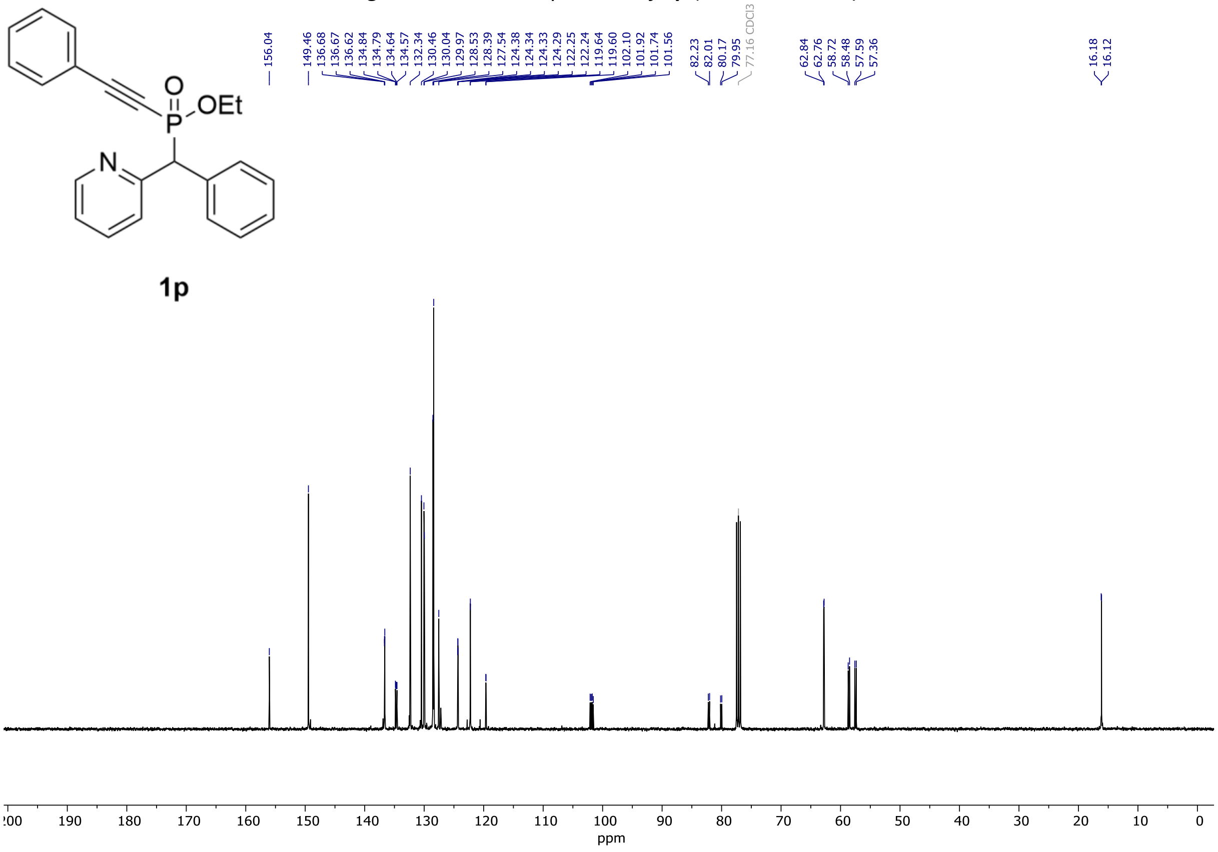

Figure S52 ^{13}C {^{1}H} NMR spectrum of **1p** (101 MHz, $CDCl_3$).

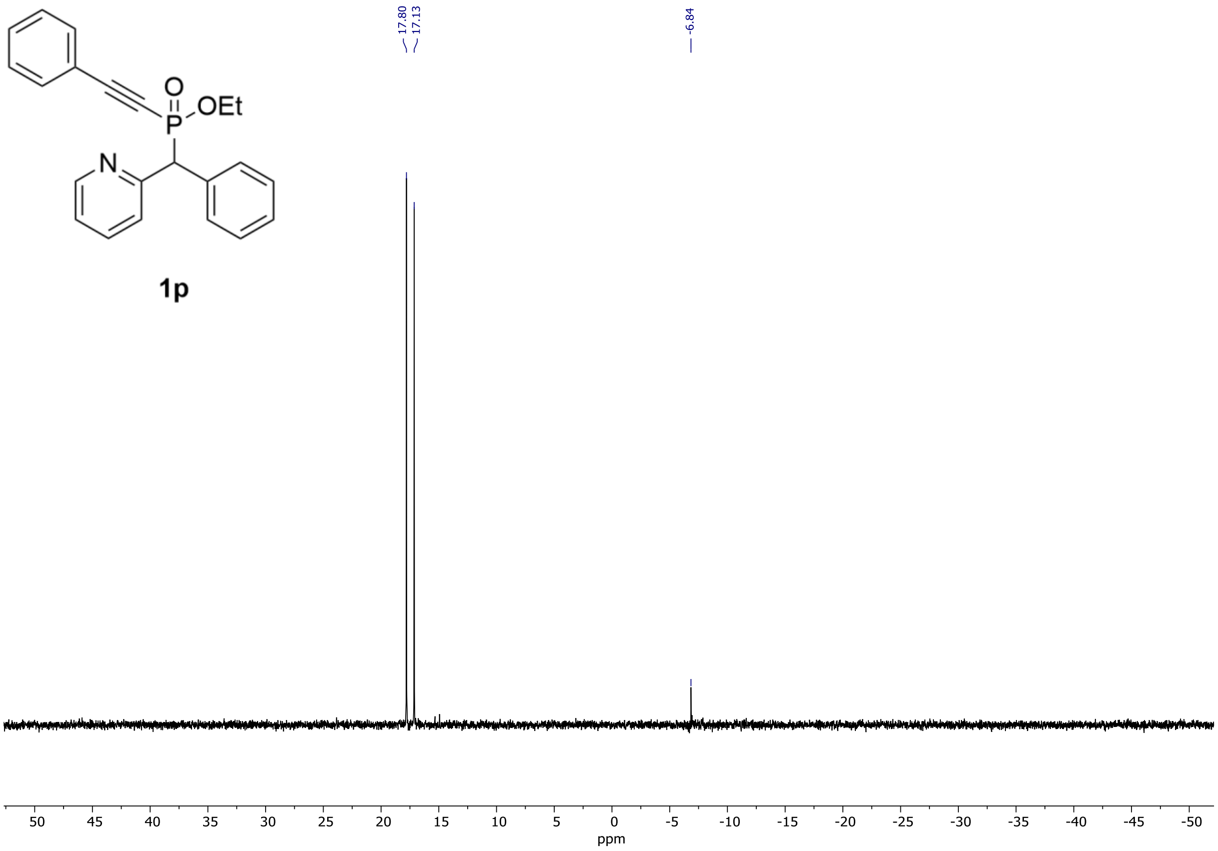

Figure S53 *^{31}P {^{1}H} NMR spectrum of **1p** (162 MHz, $CDCl_3$).*

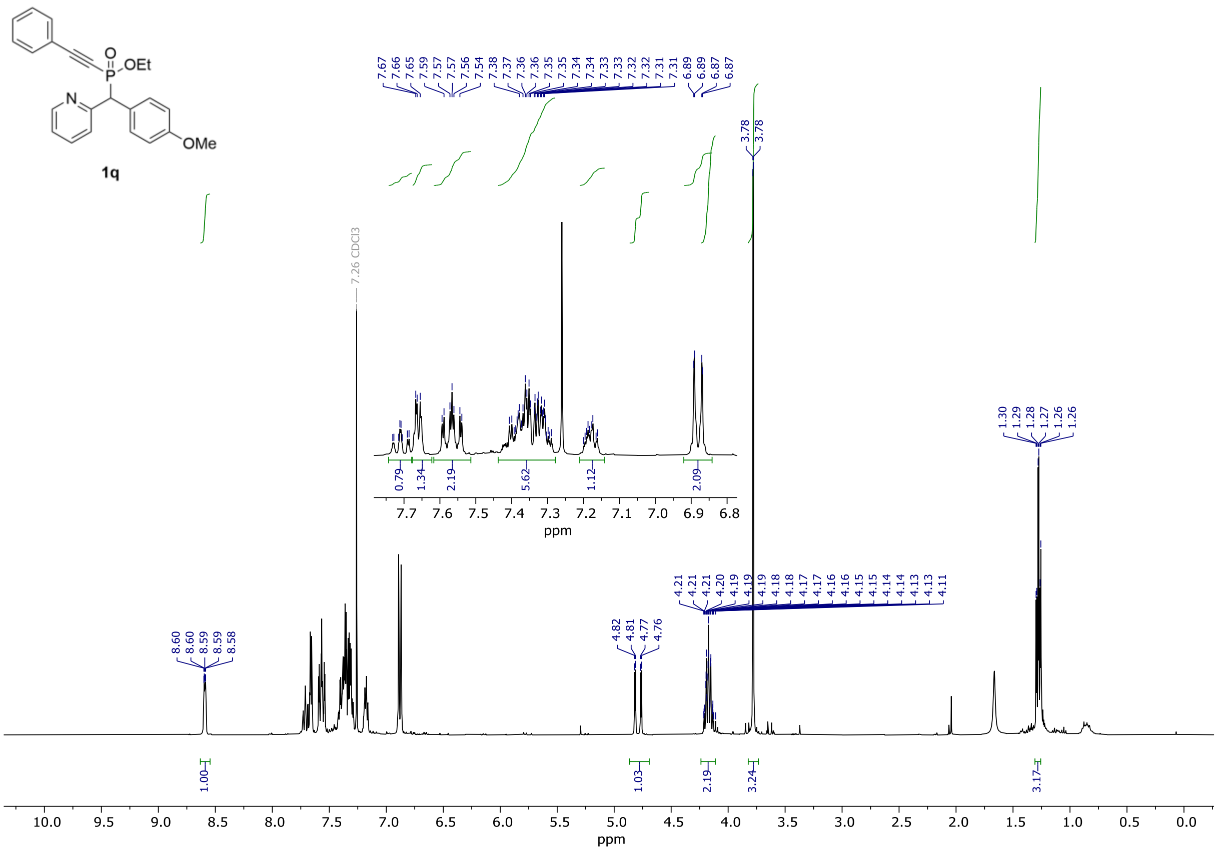

Figure S54 *^{1}H NMR spectrum of **1q** (400 MHz, $CDCl_3$).*

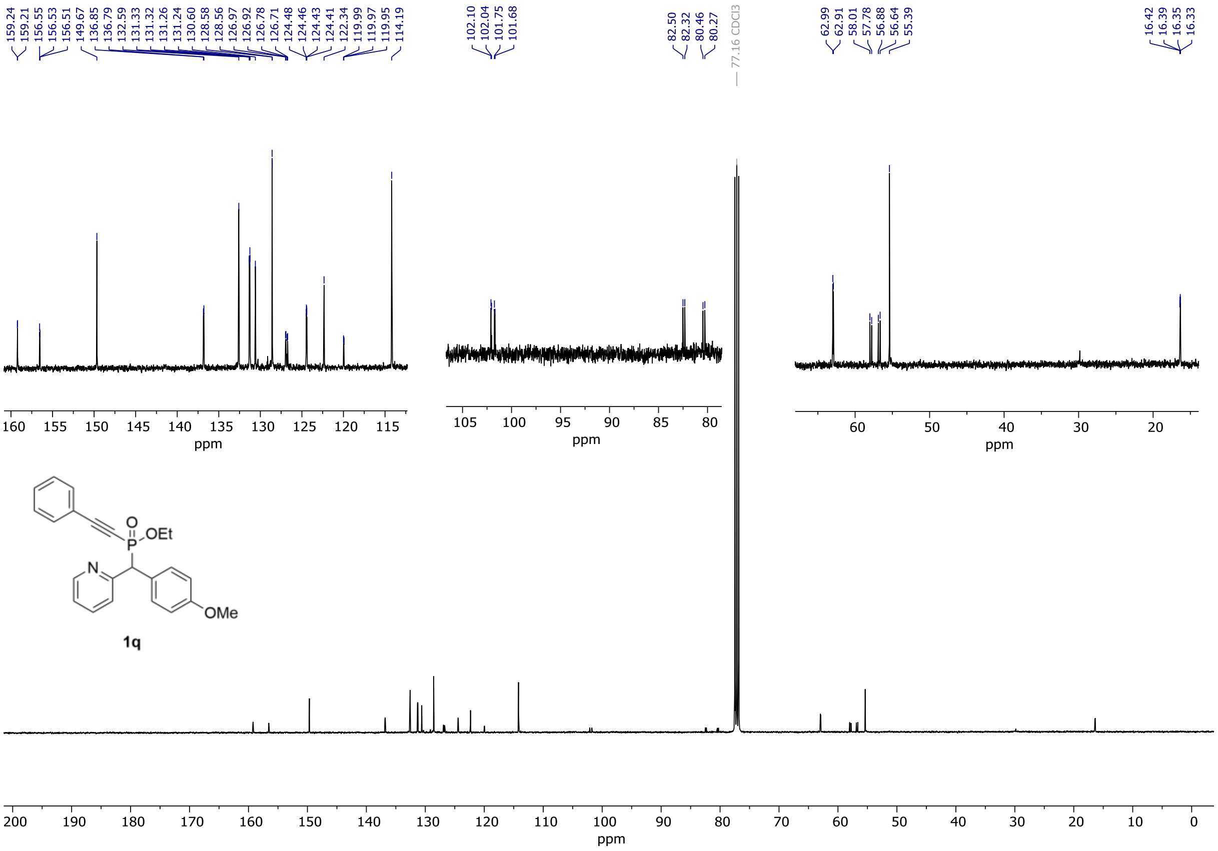

Figure S55 *^{13}C {^{1}H} NMR spectrum of* ***1q*** *(101 MHz, $CDCl_3$).*

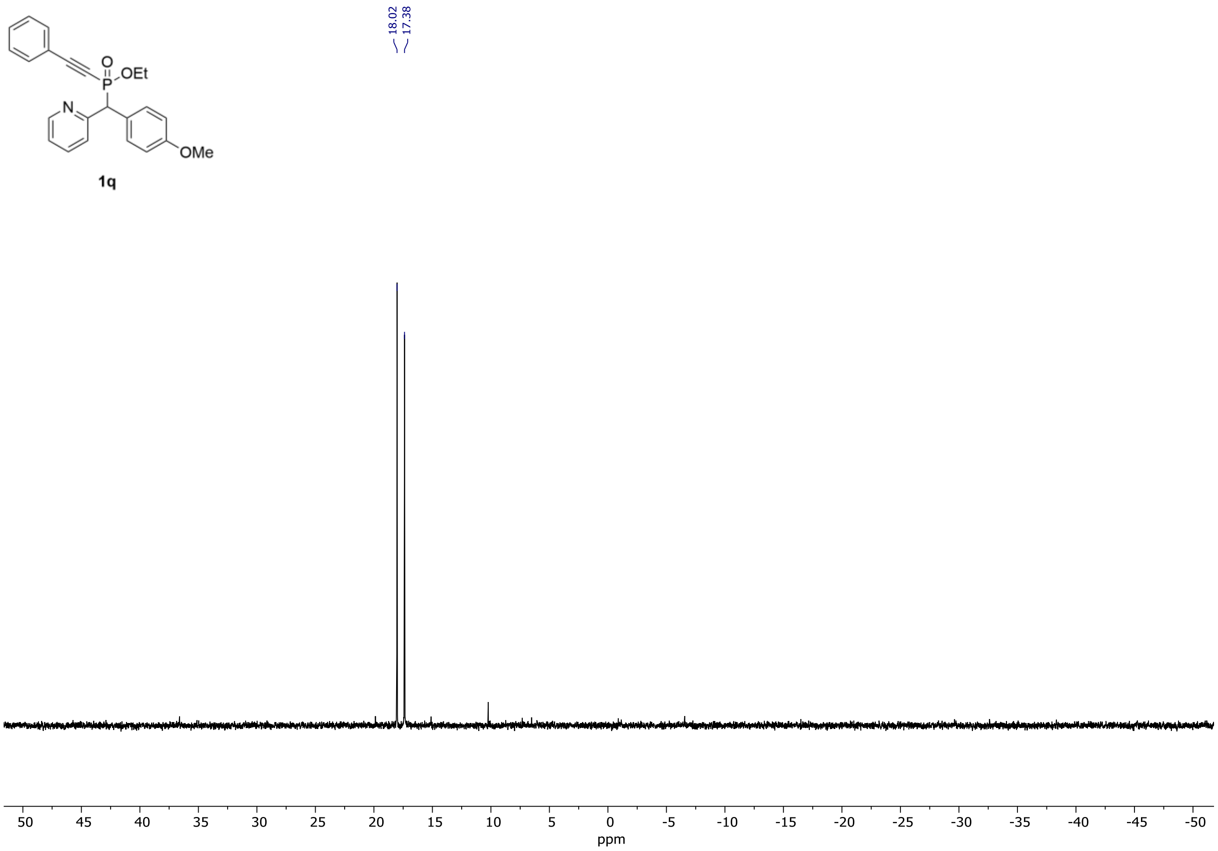

Figure S56 *^{31}P {^{1}H} NMR spectrum of* ***1q*** *(162 MHz, $CDCl_3$).*

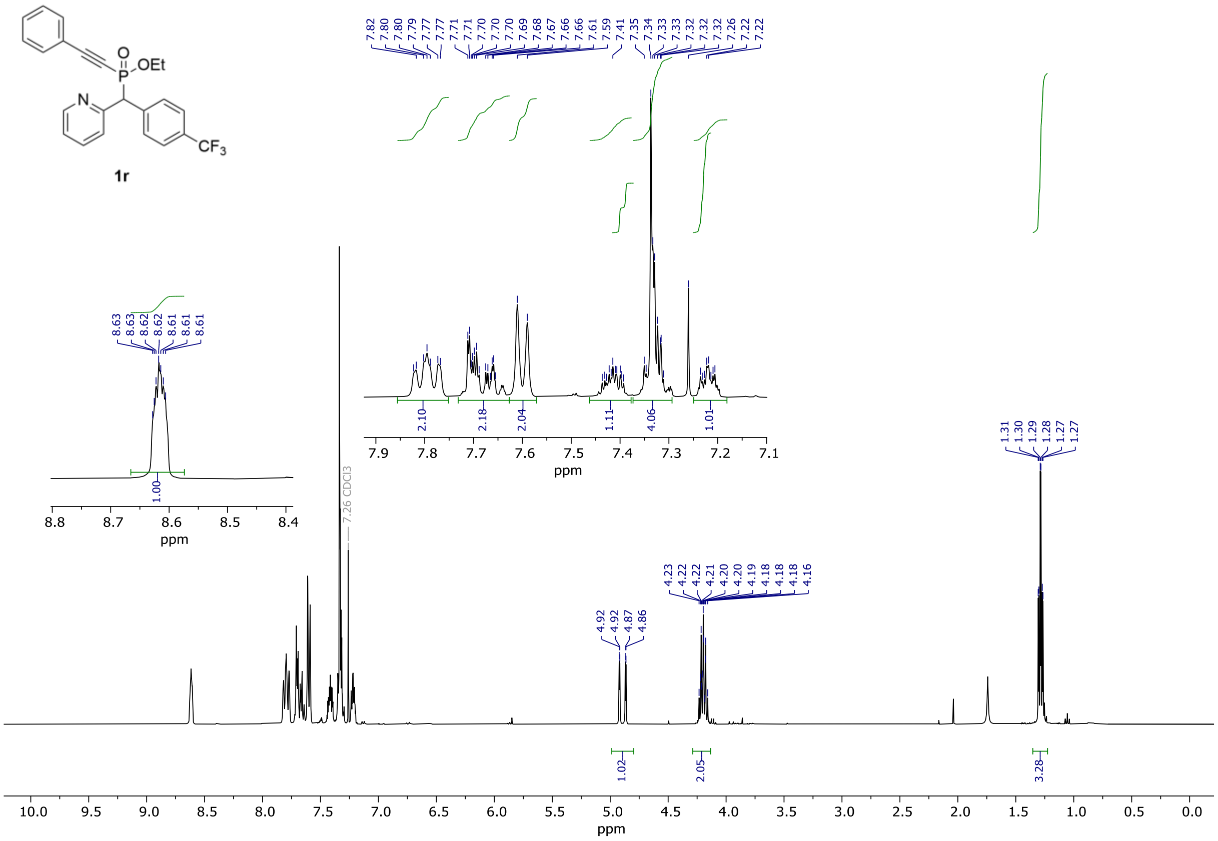

Figure S57 *^{1}H NMR spectrum of **1r** (400 MHz, $CDCl_3$).*

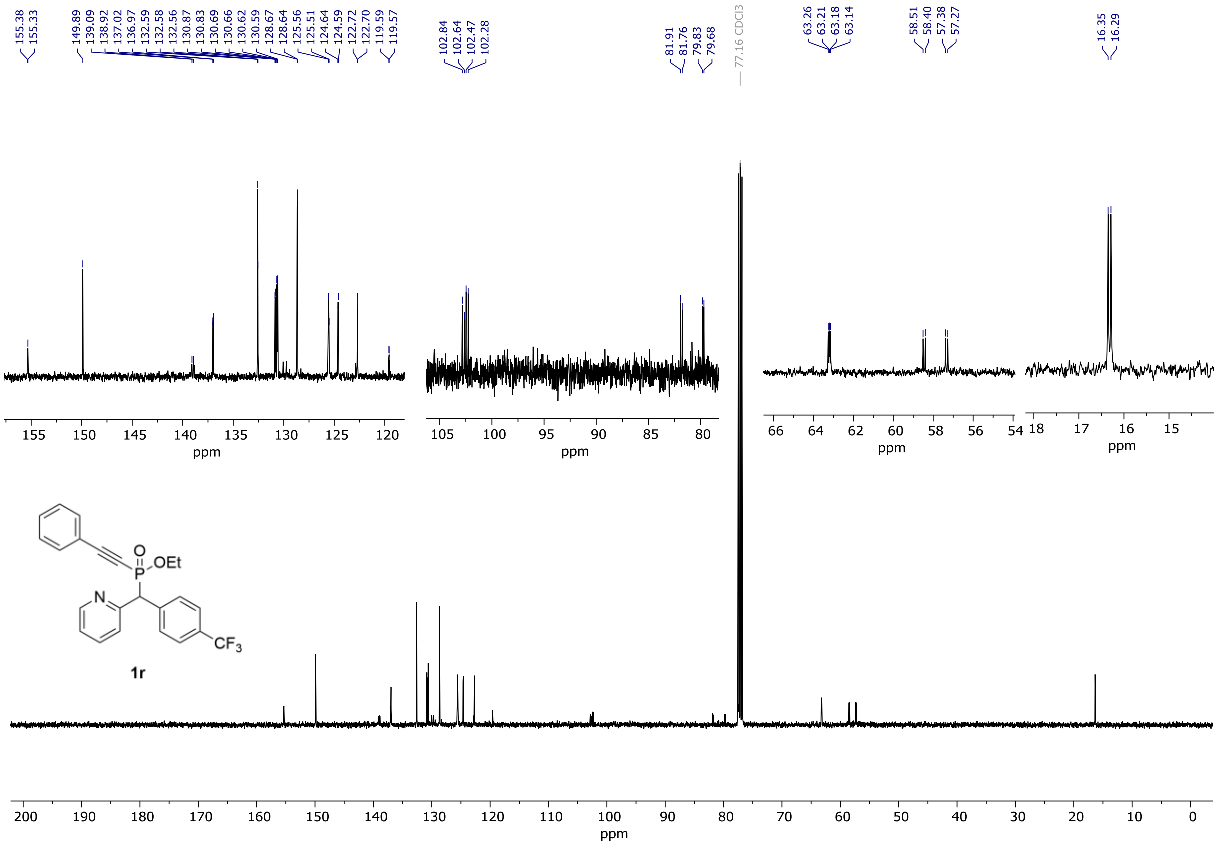

Figure S58 *^{13}C {^{1}H} NMR spectrum of **1r** (101 MHz, $CDCl_3$).*

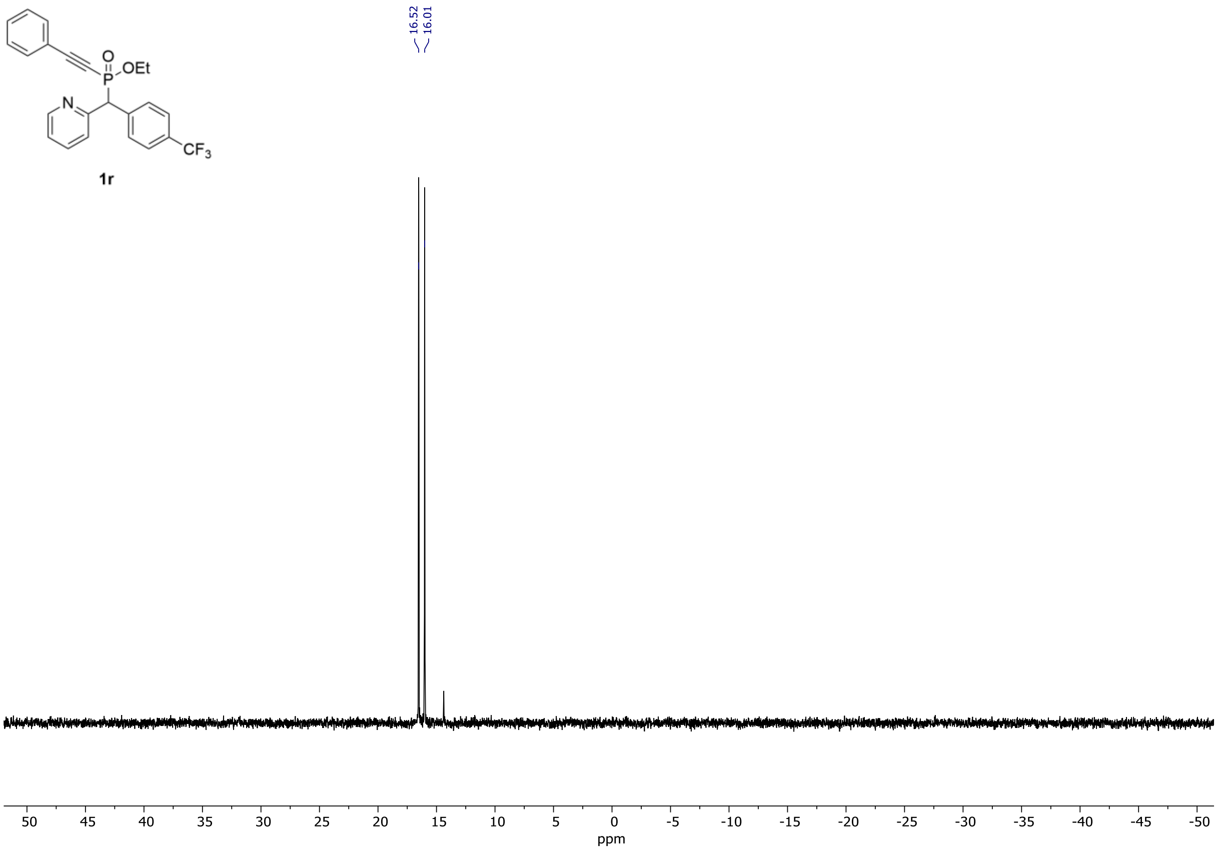

Figure S59 *^{31}P {^{1}H} NMR spectrum of **1r** (162 MHz, $CDCl_3$).*

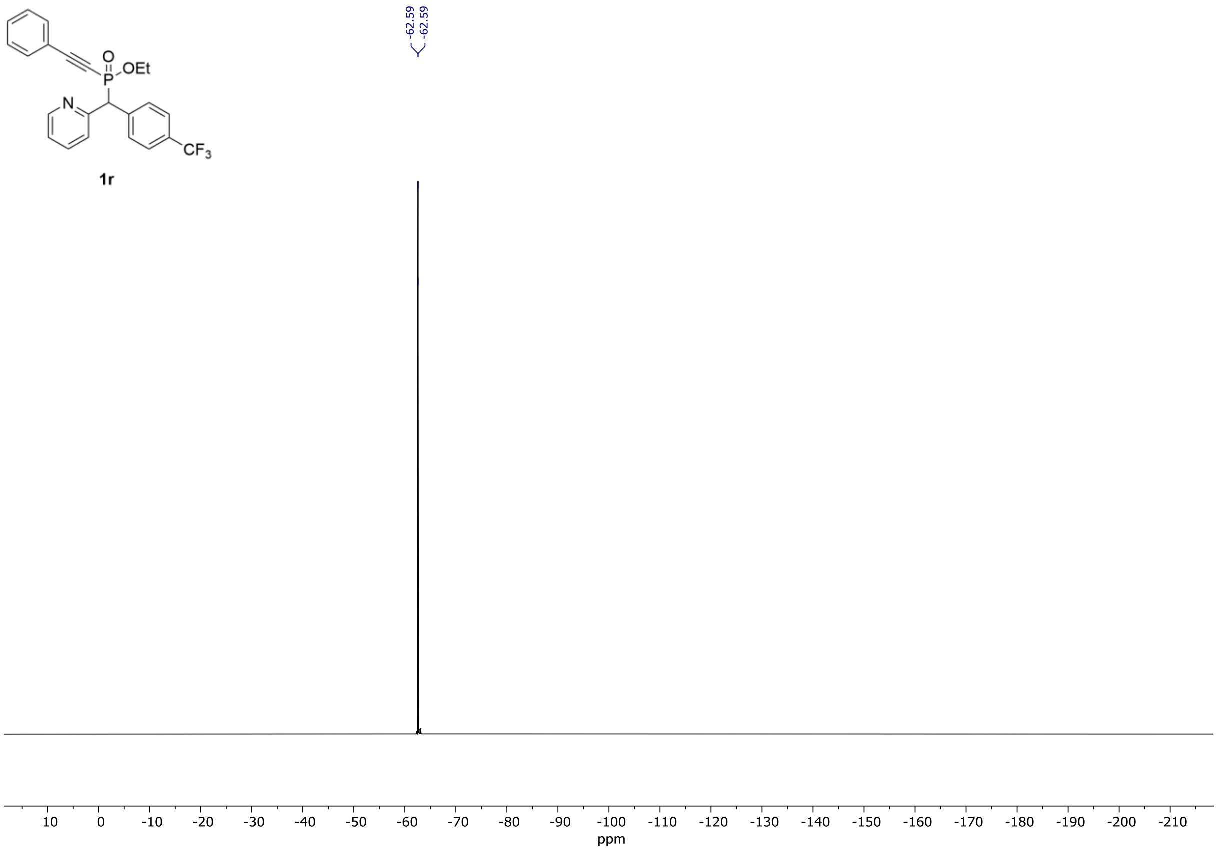

Figure S60 *^{19}F {^{1}H} NMR spectrum of **1r** (376 MHz, $CDCl_3$).*

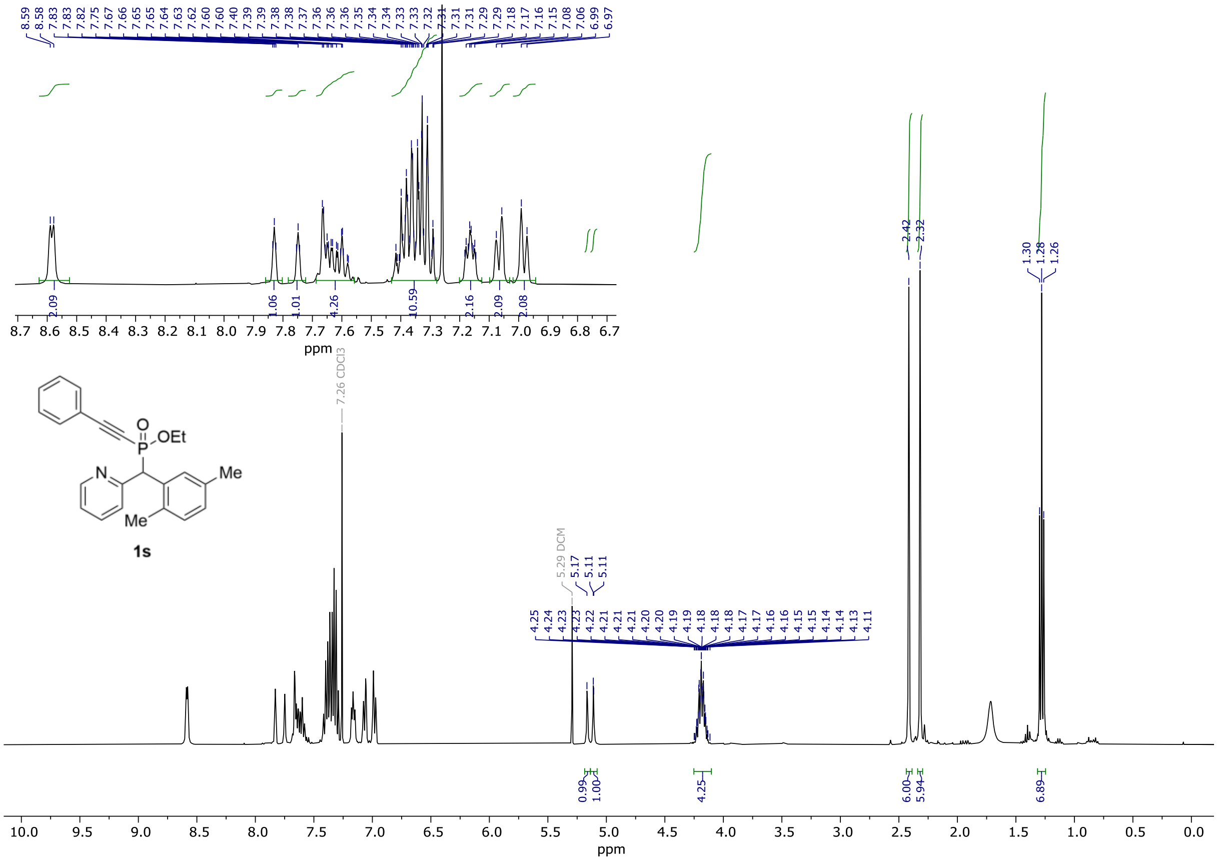

Figure S61 ^{1}H *NMR spectrum of* ***1s*** *(400 MHz, CDCl$_3$).*

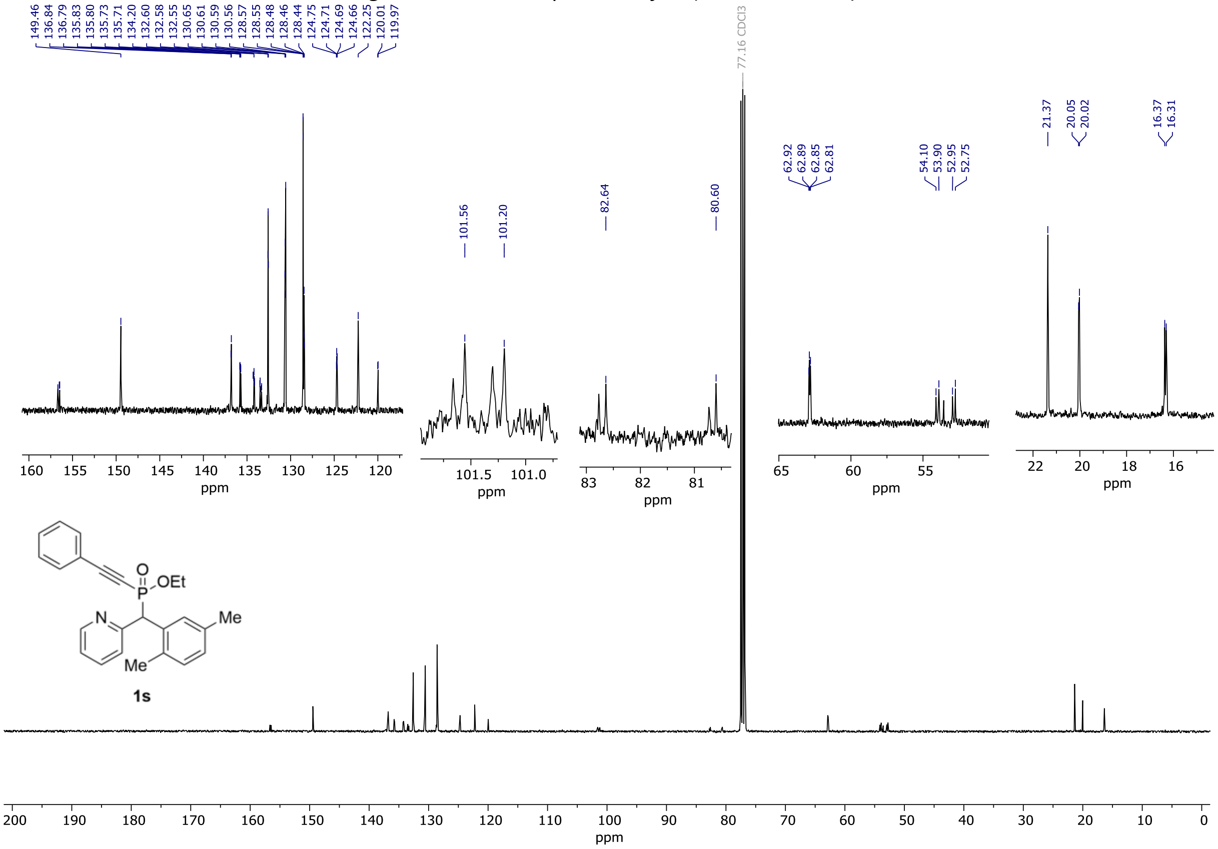

Figure S62 ^{13}C *{^{1}H} NMR spectrum of* ***1s*** *(101 MHz, CDCl$_3$).*

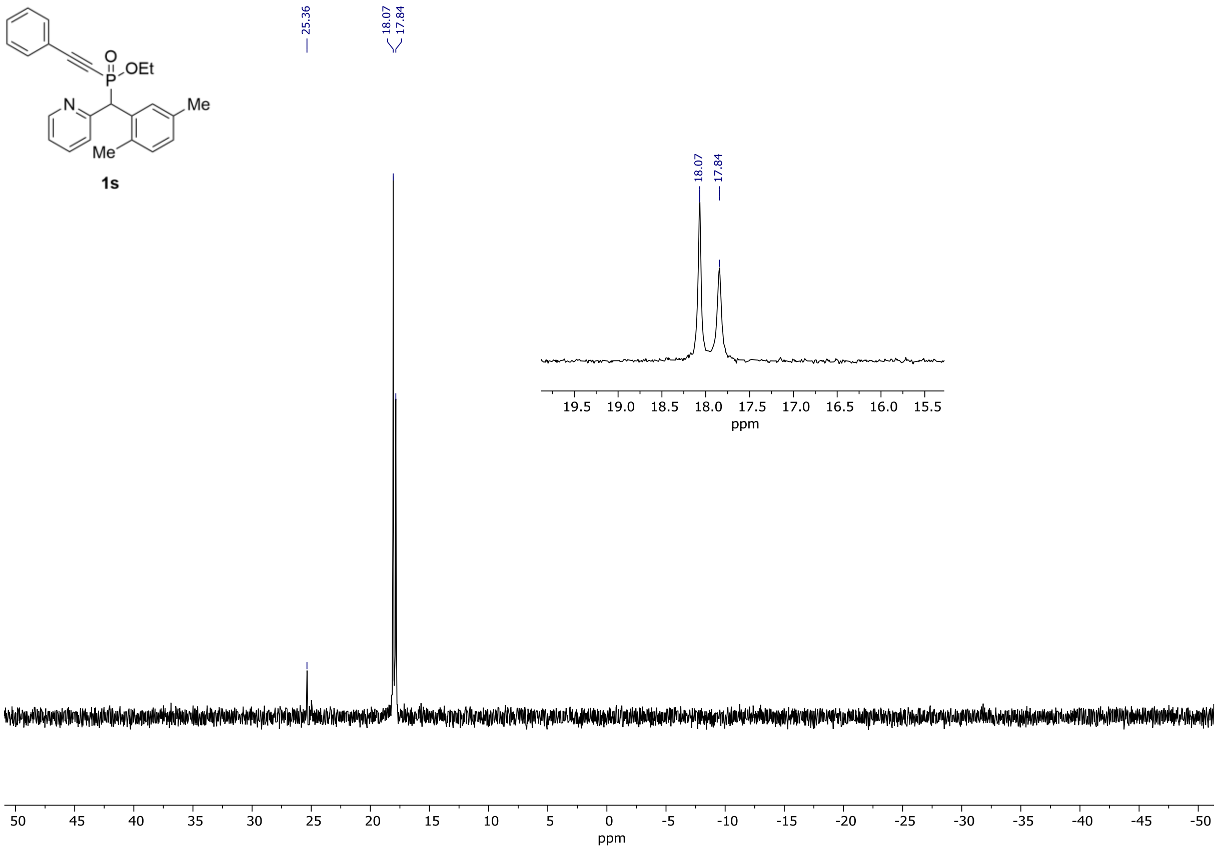

Figure S63 *^{31}P {^{1}H} NMR spectrum of* ***1s*** *(162 MHz, $CDCl_3$).*

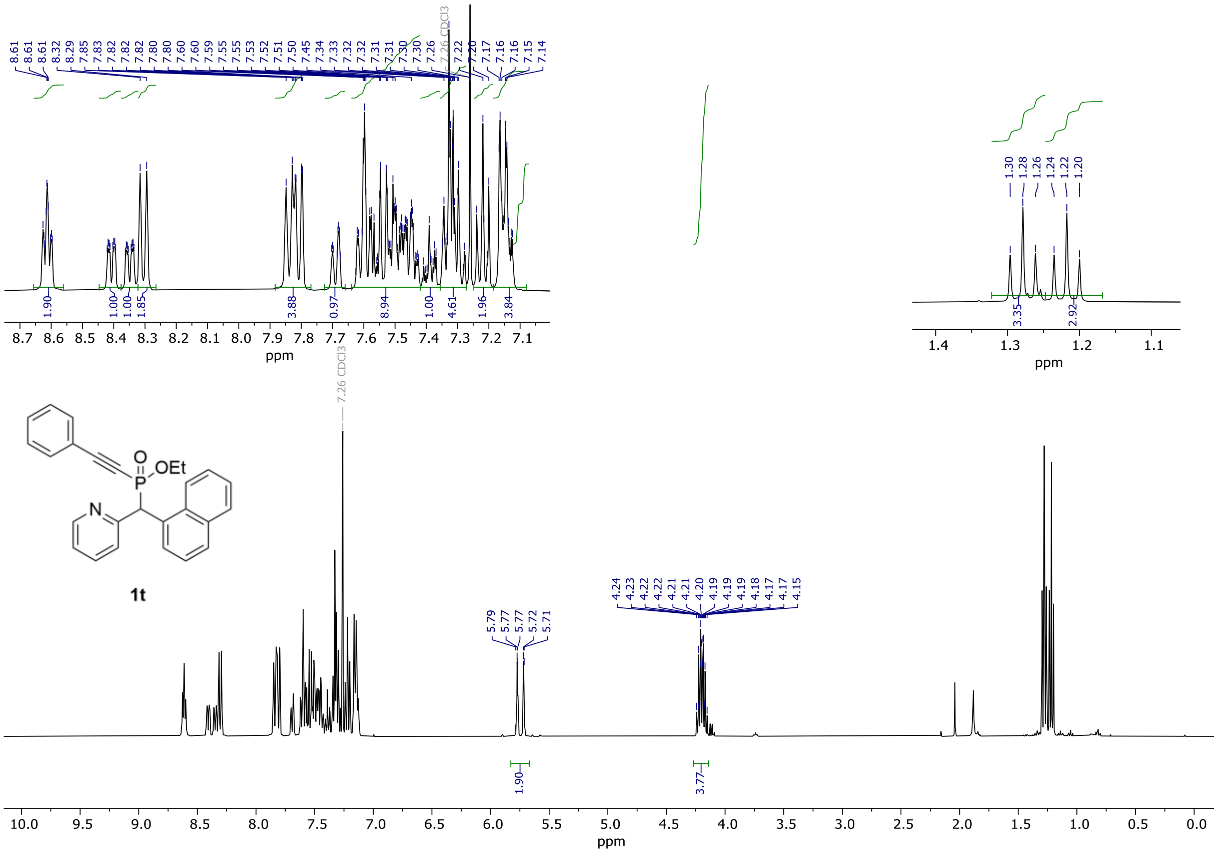

Figure S64 *^{1}H NMR spectrum of* ***1t*** *(400 MHz, $CDCl_3$).*

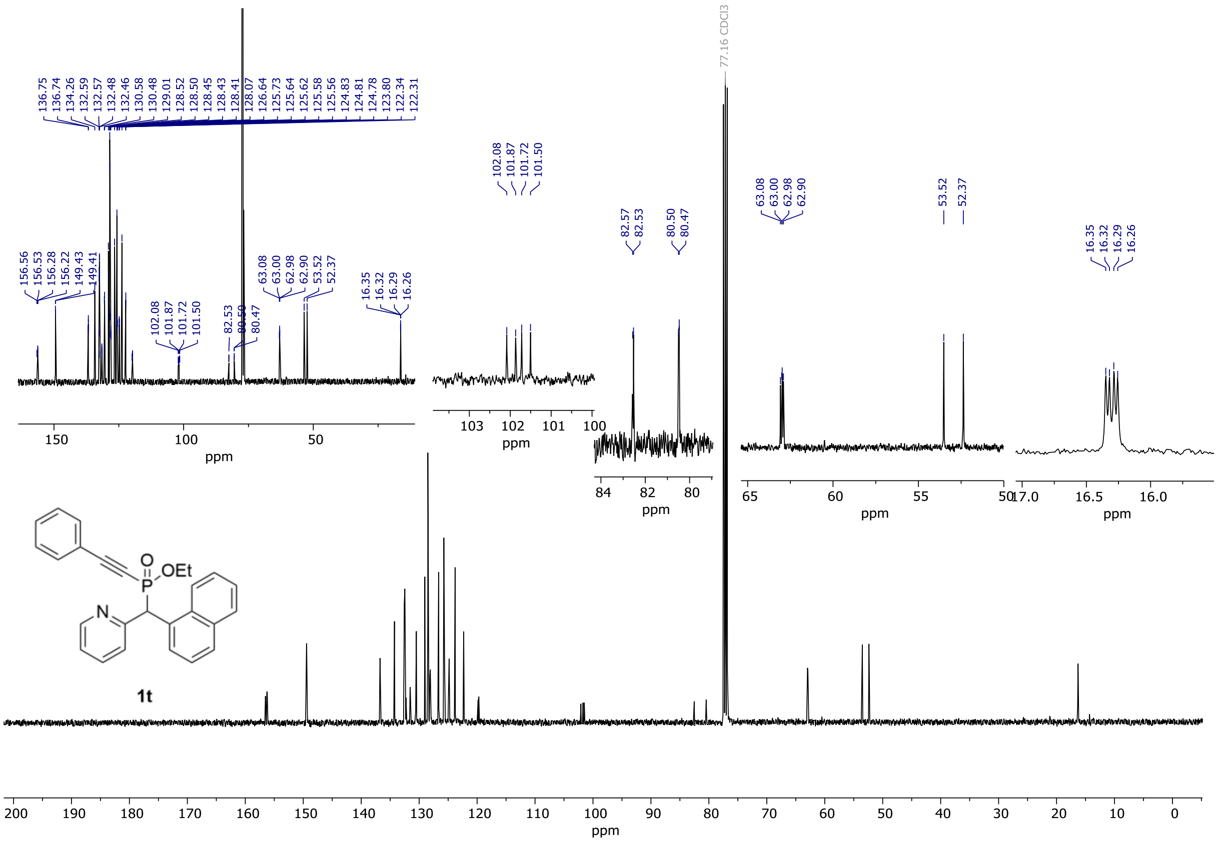

Figure S65 *^{13}C {^{1}H} NMR spectrum of **1t** (101 MHz, CDCl$_3$).*

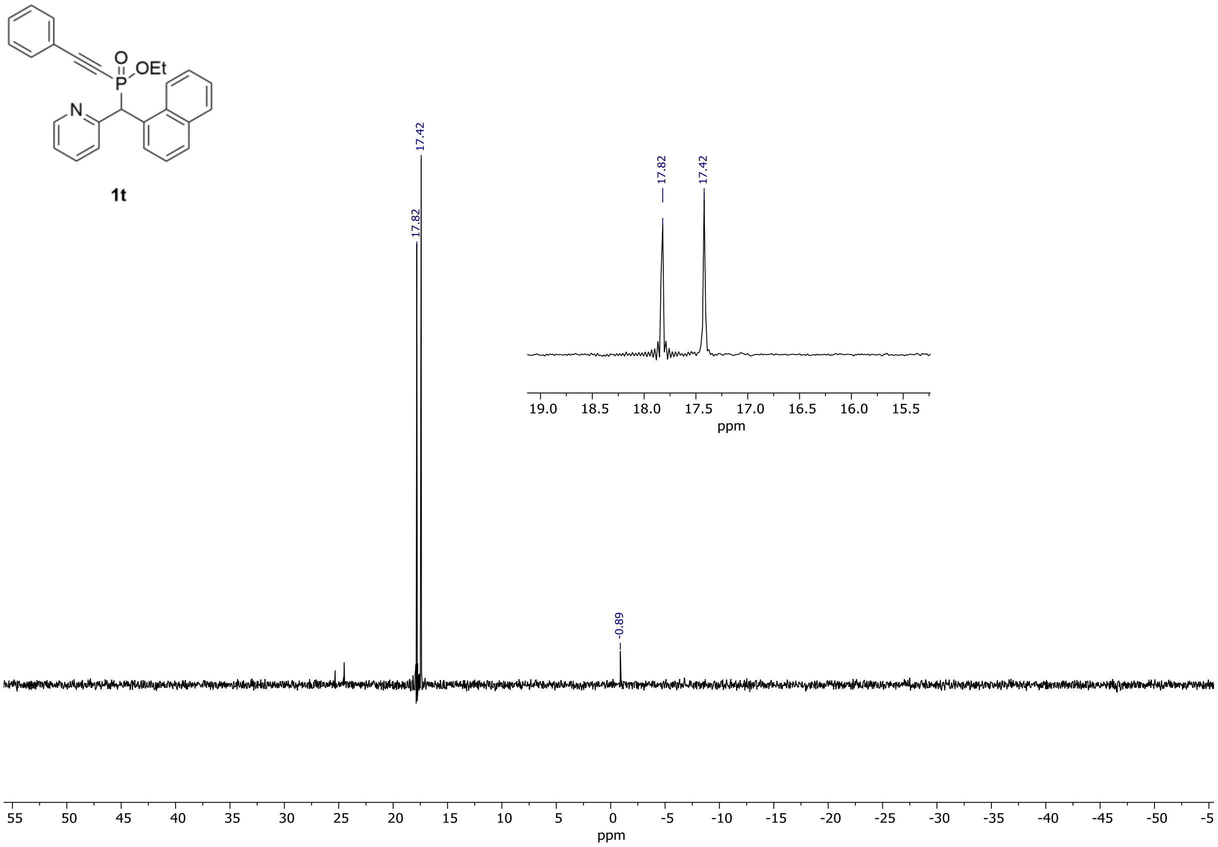

Figure S66 *^{31}P {^{1}H} NMR spectrum of **1t** (162 MHz, CDCl$_3$).*

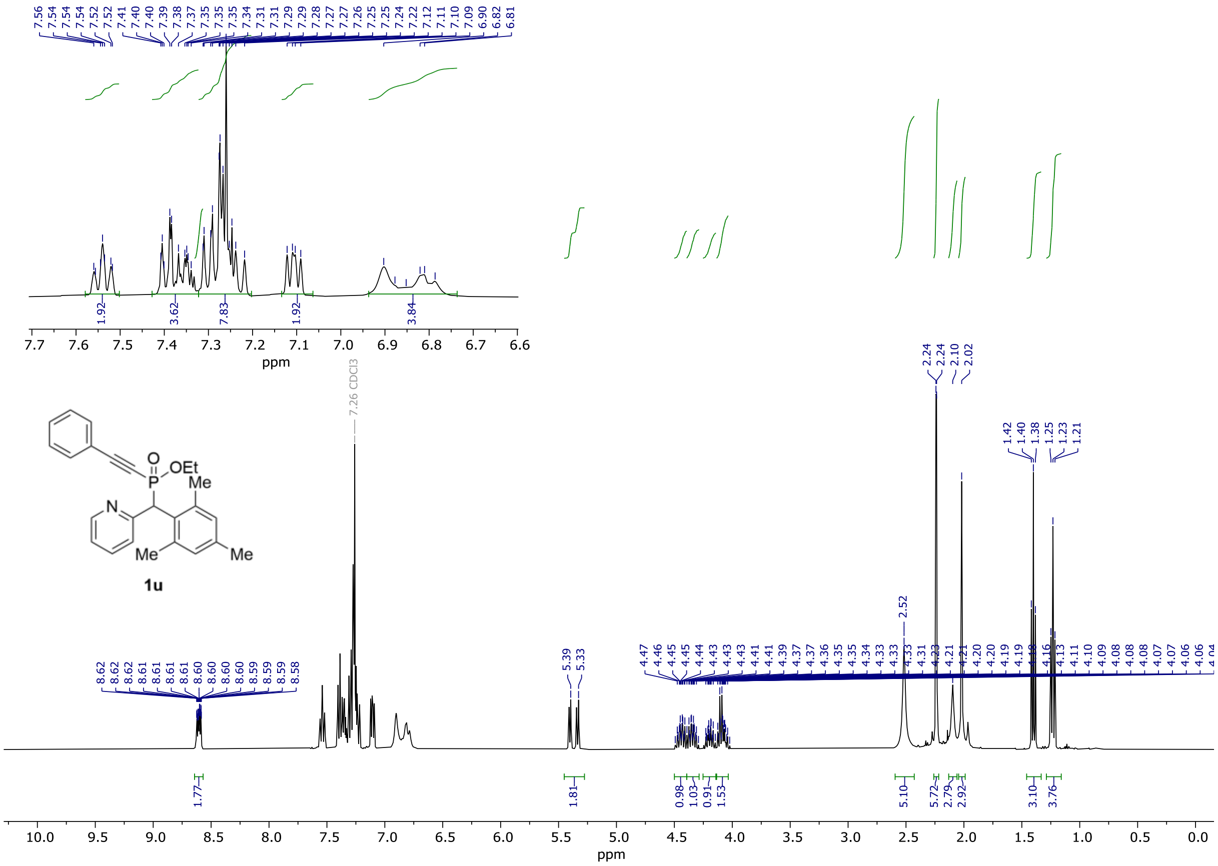

Figure S67 ^{1}H NMR spectrum of **1u** (400 MHz, $CDCl_3$).

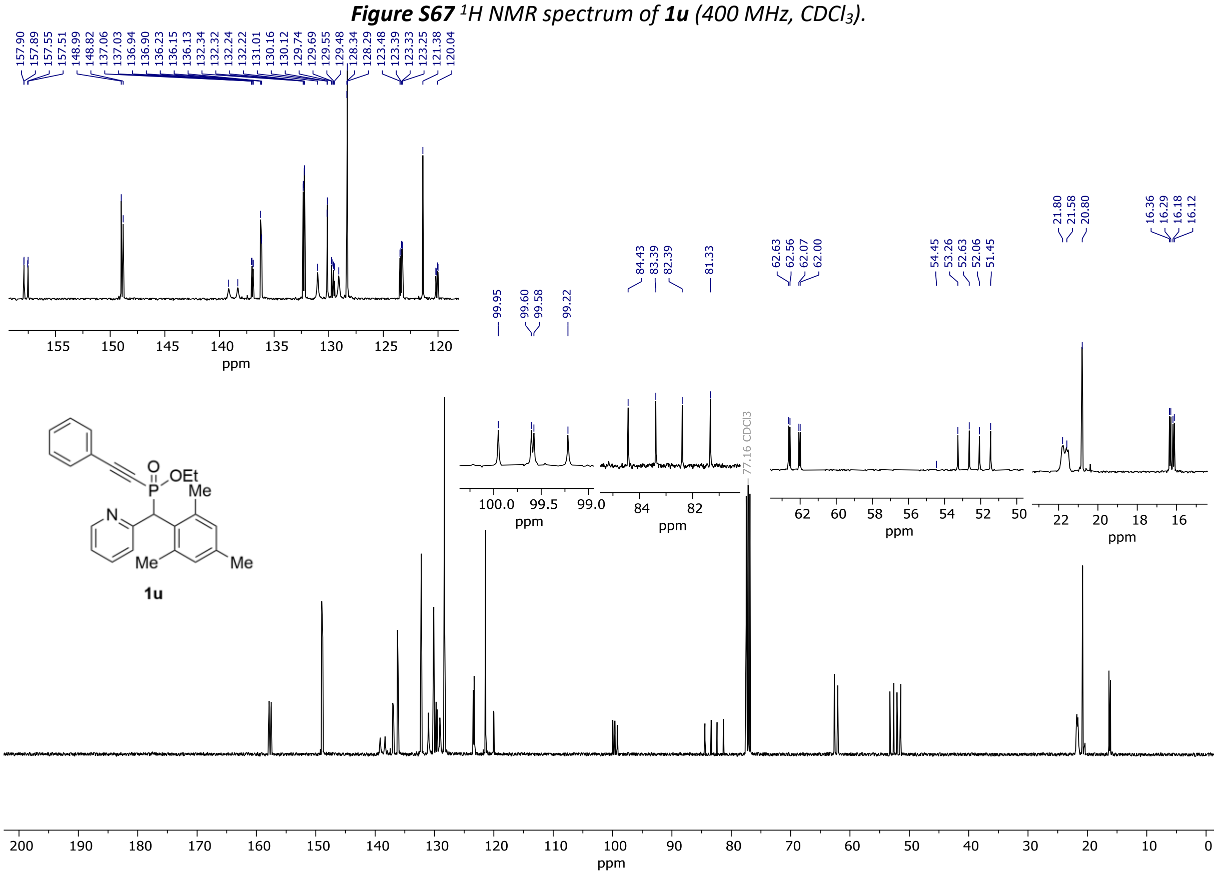

Figure S68 ^{13}C {^{1}H} NMR spectrum of **1u** (101 MHz, $CDCl_3$).

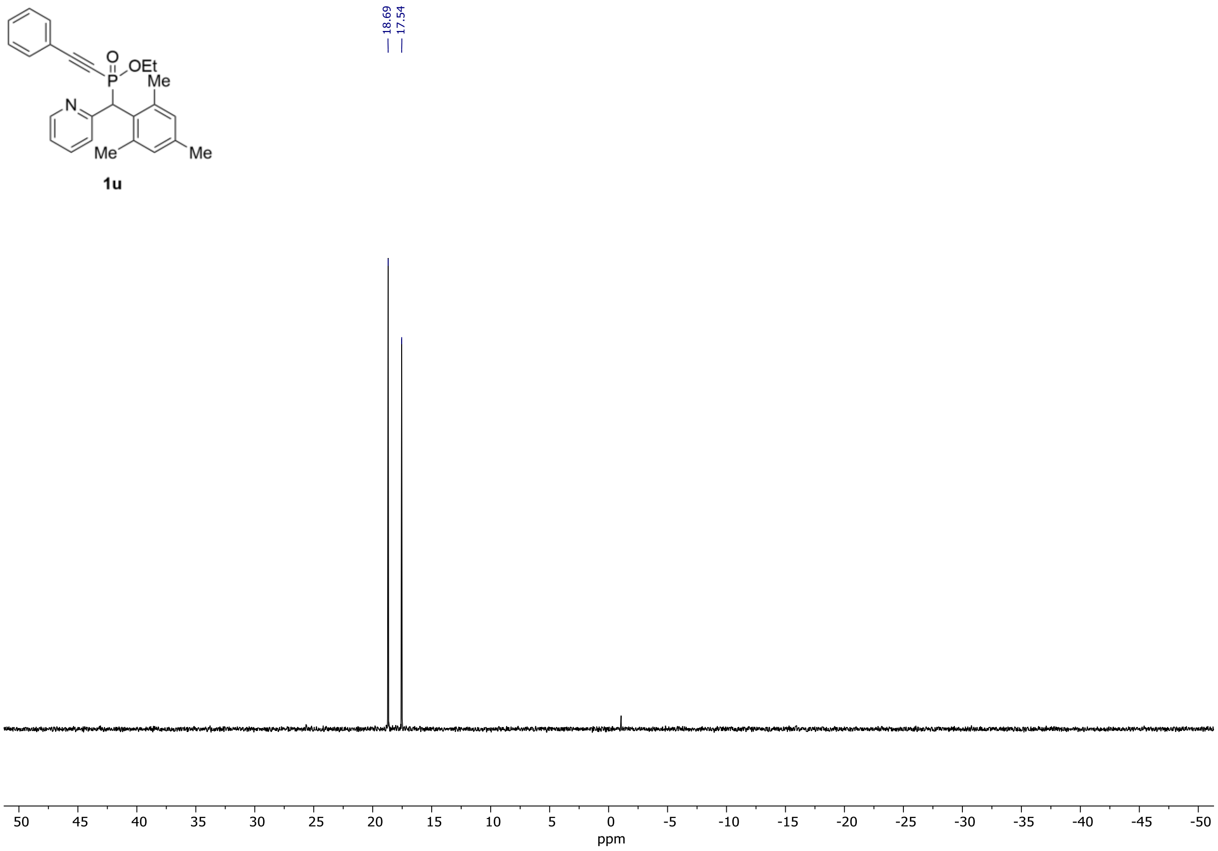

Figure S69 *^{31}P {^{1}H} NMR spectrum of* ***1u*** *(162 MHz, $CDCl_3$).*

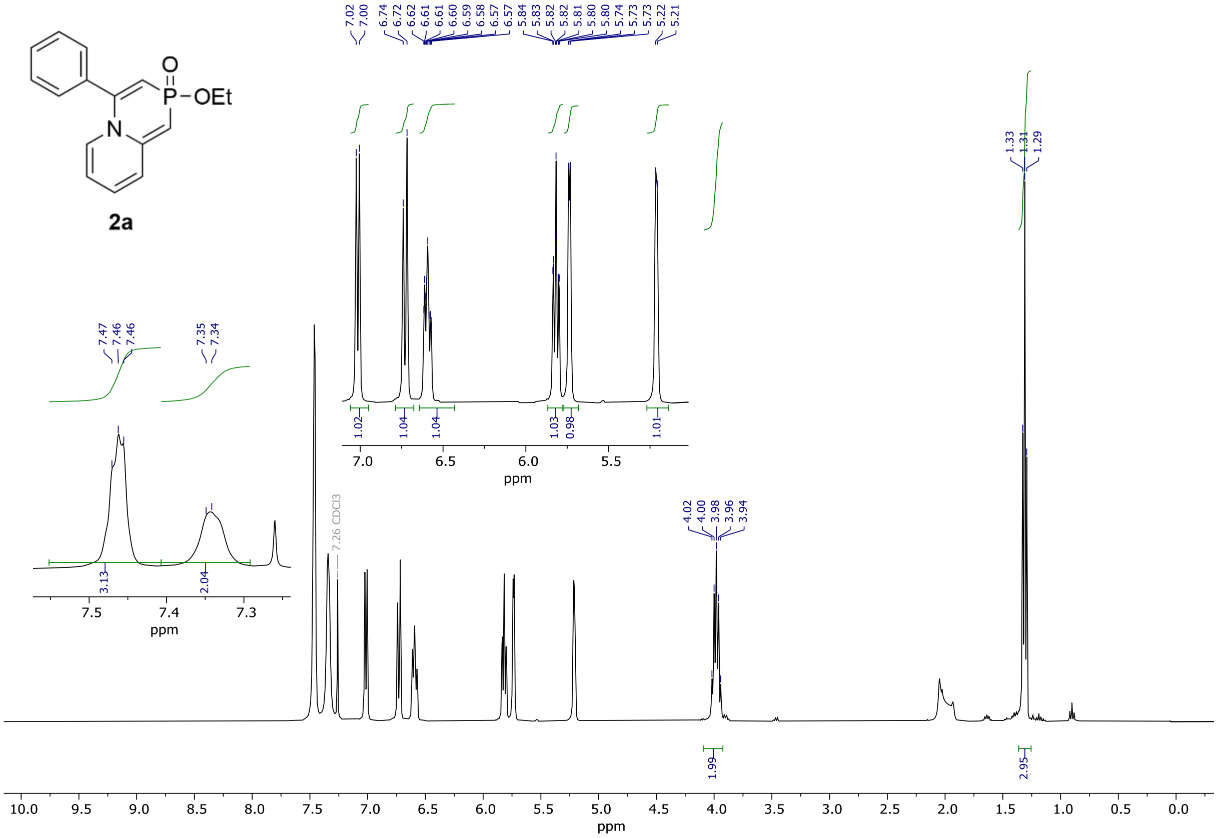

Figure S70 *^{1}H NMR spectrum of* ***2a*** *(400 MHz, $CDCl_3$).*

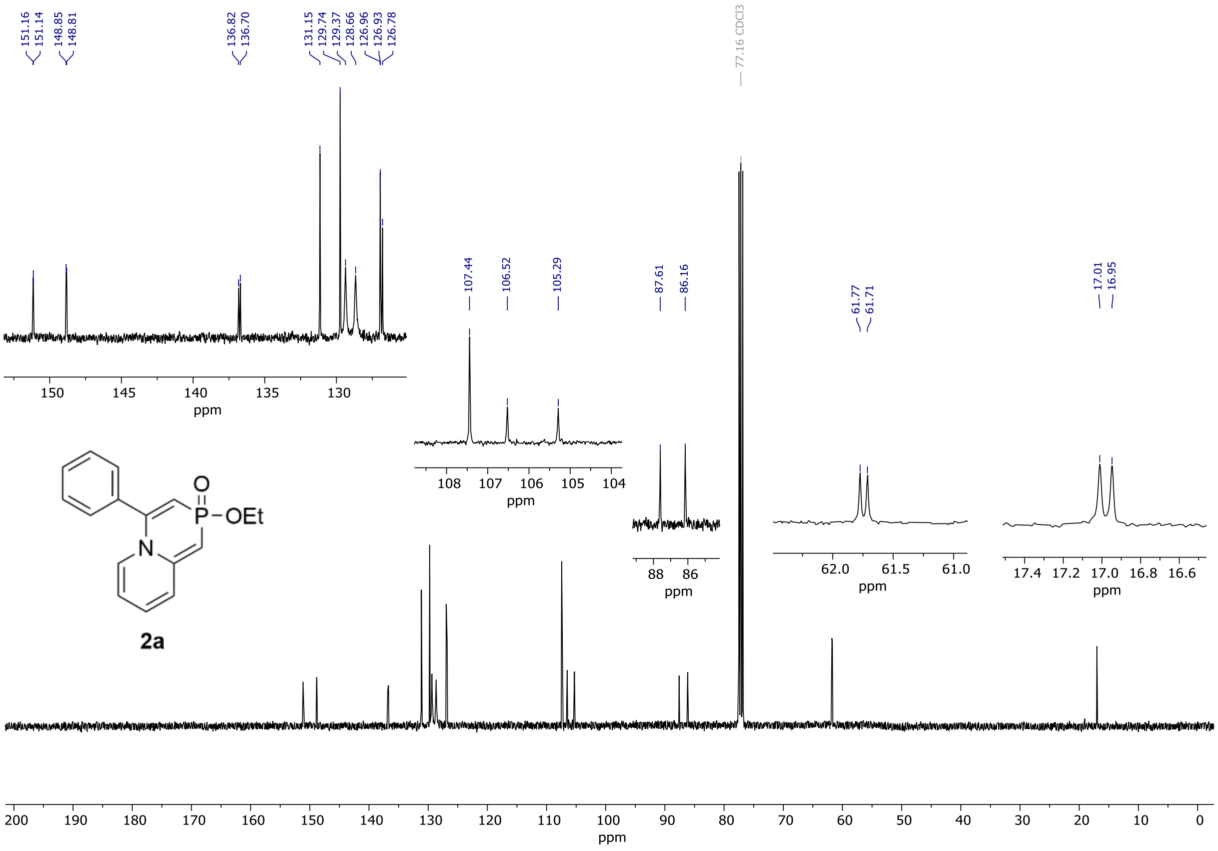

Figure S71 ^{13}C {^{1}H} *NMR spectrum of **2a** (101 MHz, $CDCl_3$).*

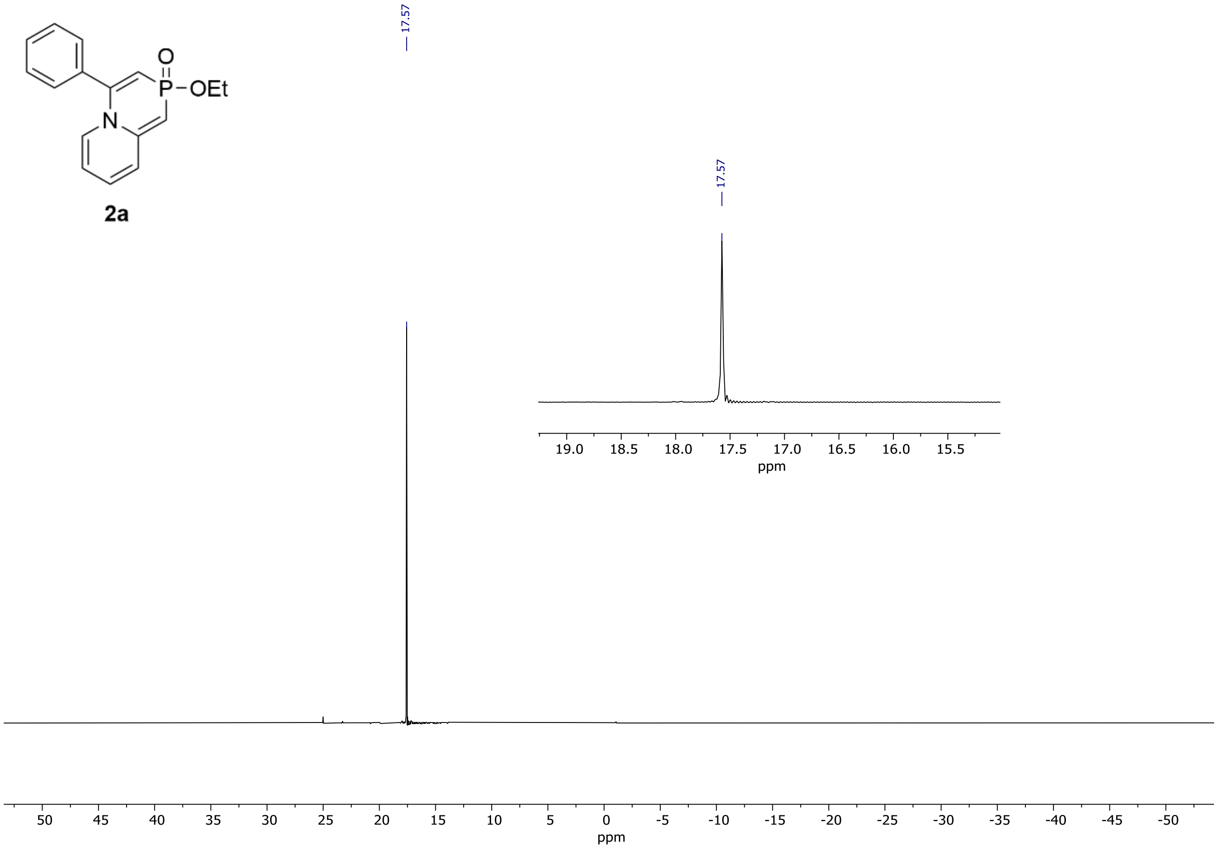

Figure S72 ^{31}P {^{1}H} *NMR spectrum of **2a** (162 MHz, $CDCl_3$).*

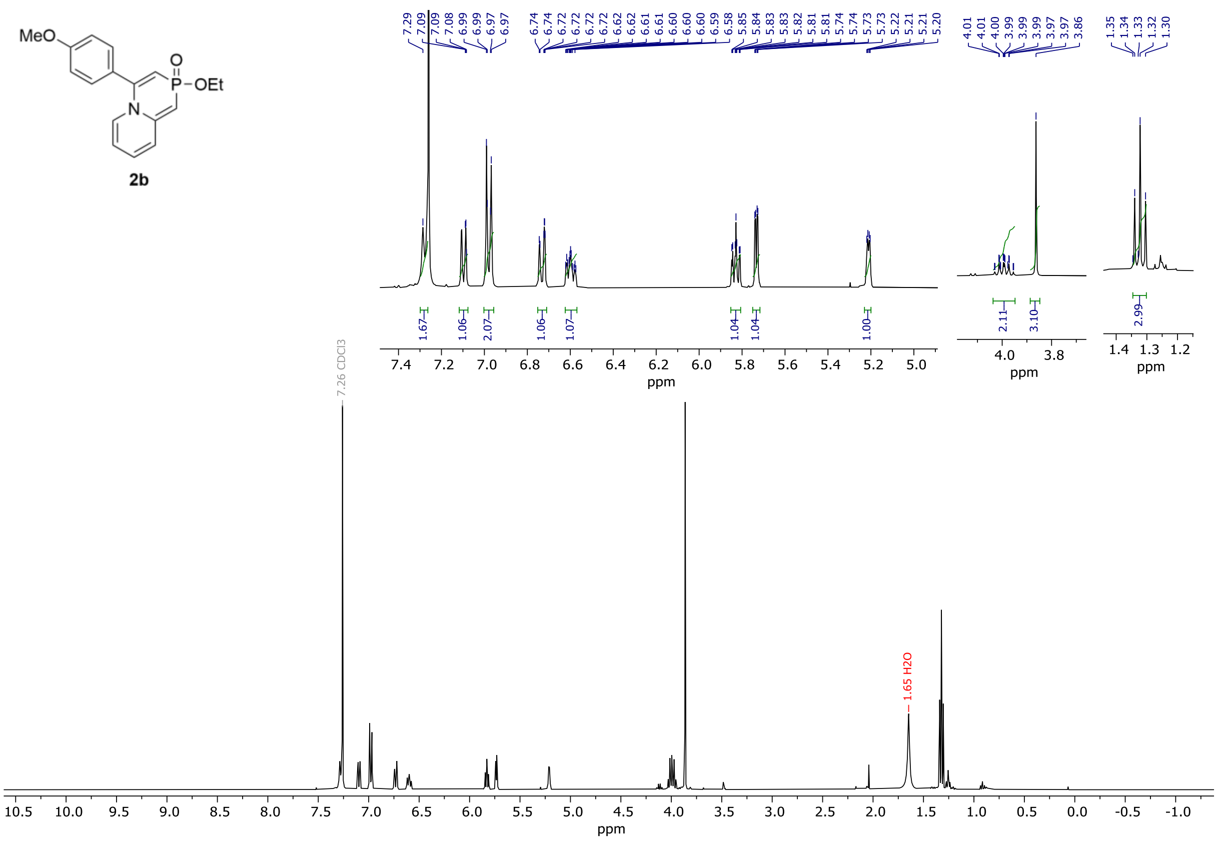

Figure S73 ^{1}H *NMR spectrum of* ***2b*** *(400 MHz, $CDCl_3$).*

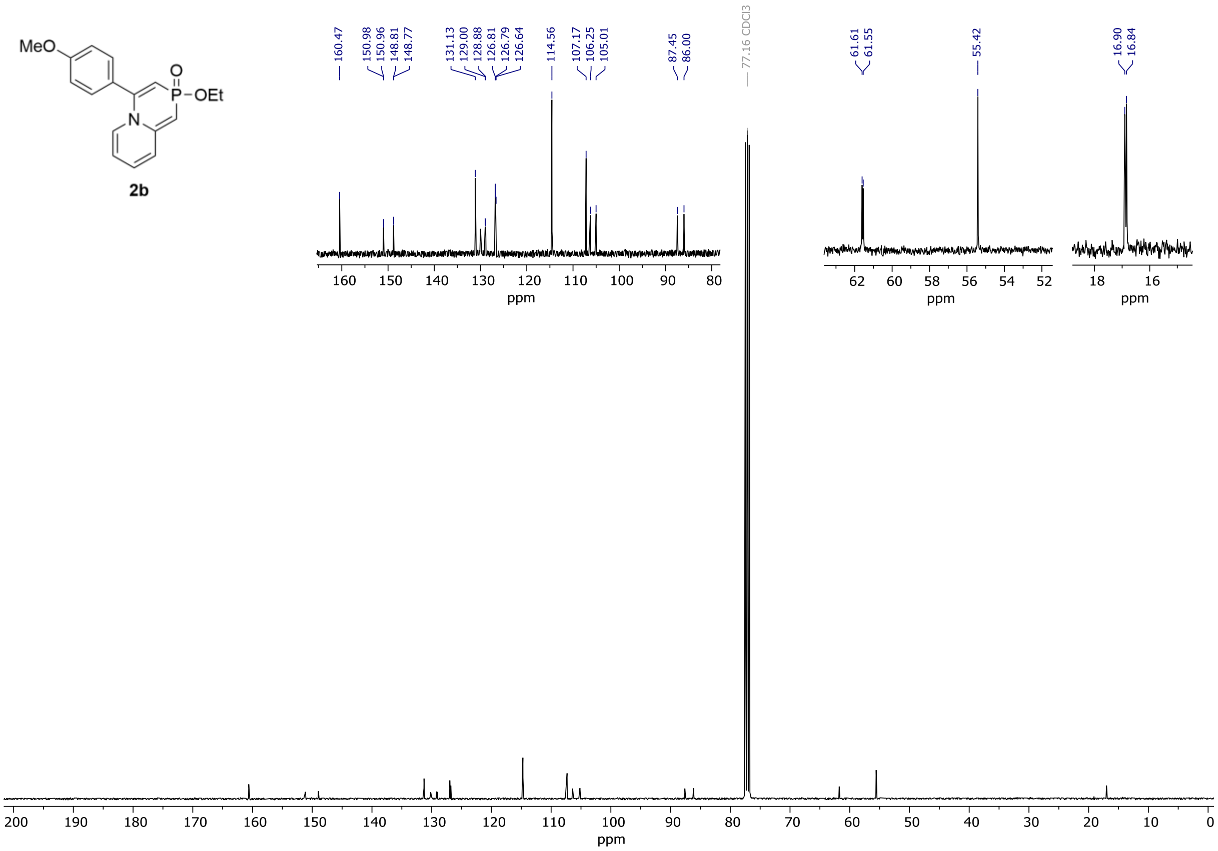

Figure S74 ^{13}C *{^{1}H} NMR spectrum of* ***2b*** *(101 MHz, $CDCl_3$).*

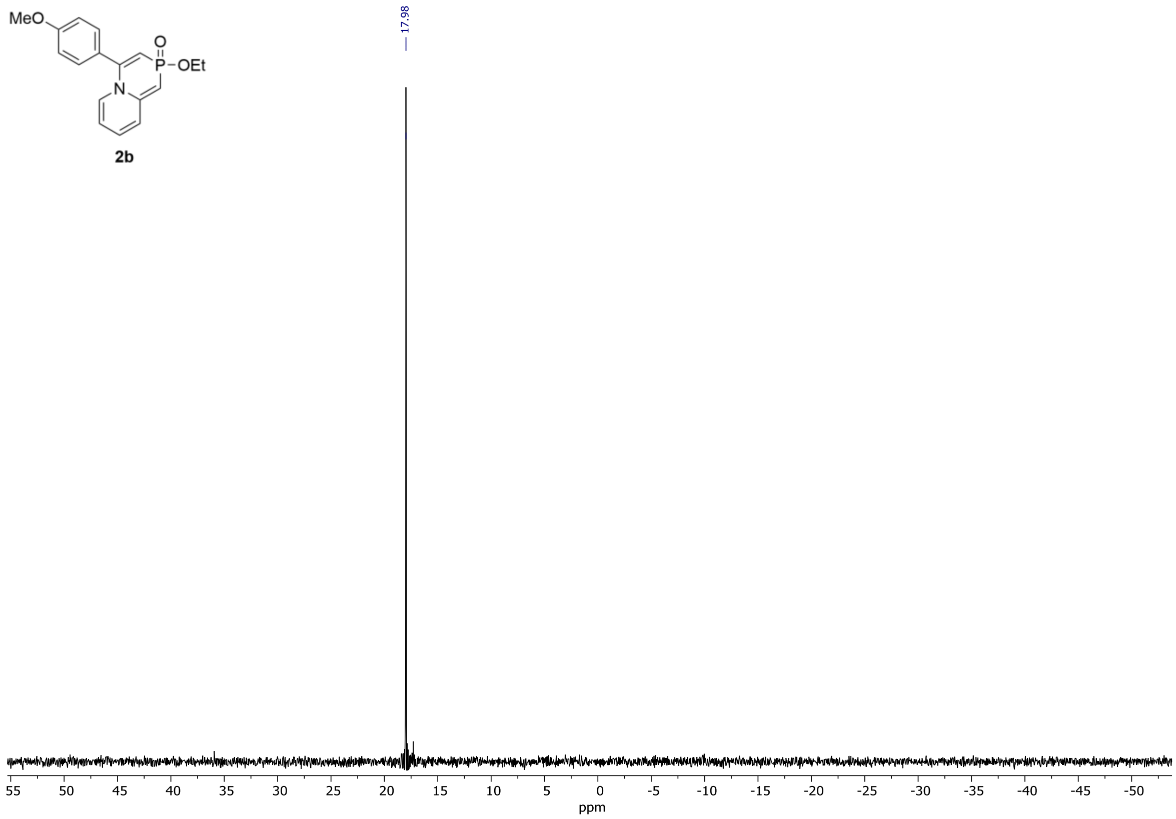

Figure S75 ^{31}P {^{1}H} NMR spectrum of **2b** (162 MHz, CDCl$_3$).

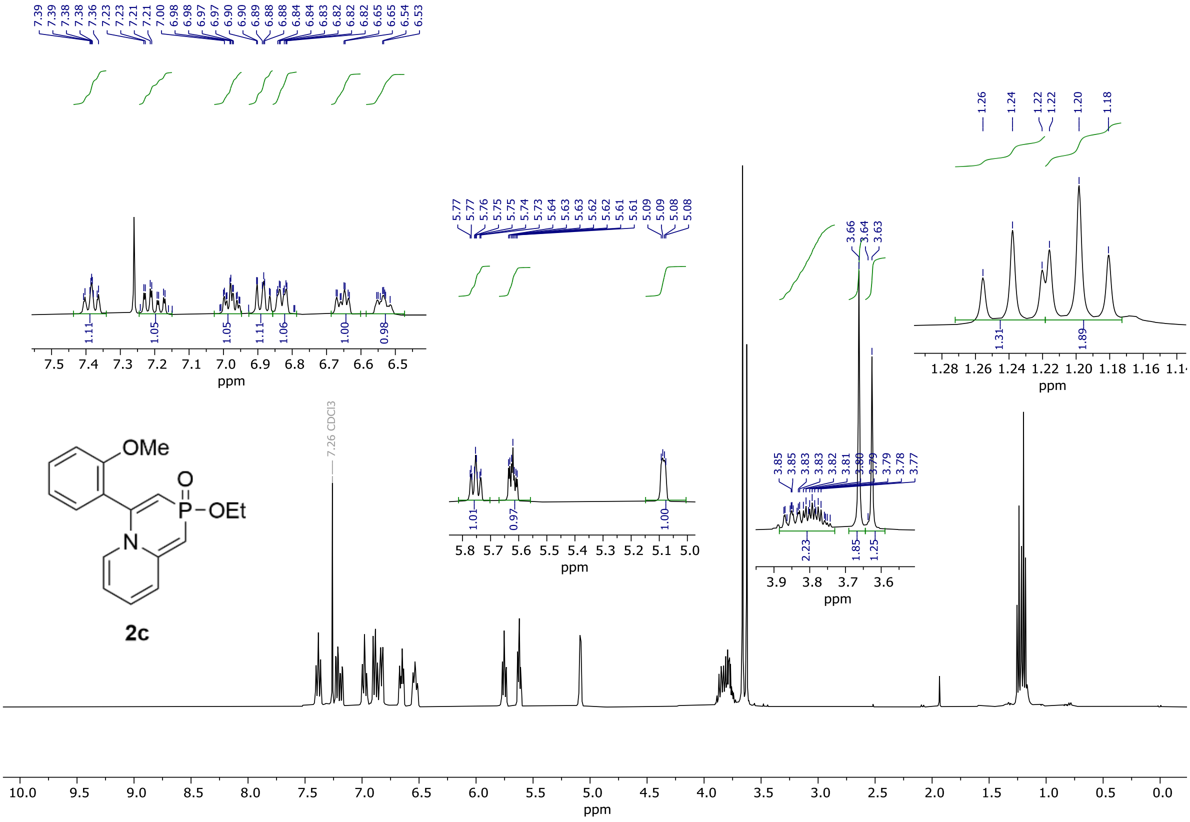

Figure S76 ^{1}H NMR spectrum of **2c** (400 MHz, CDCl$_3$).

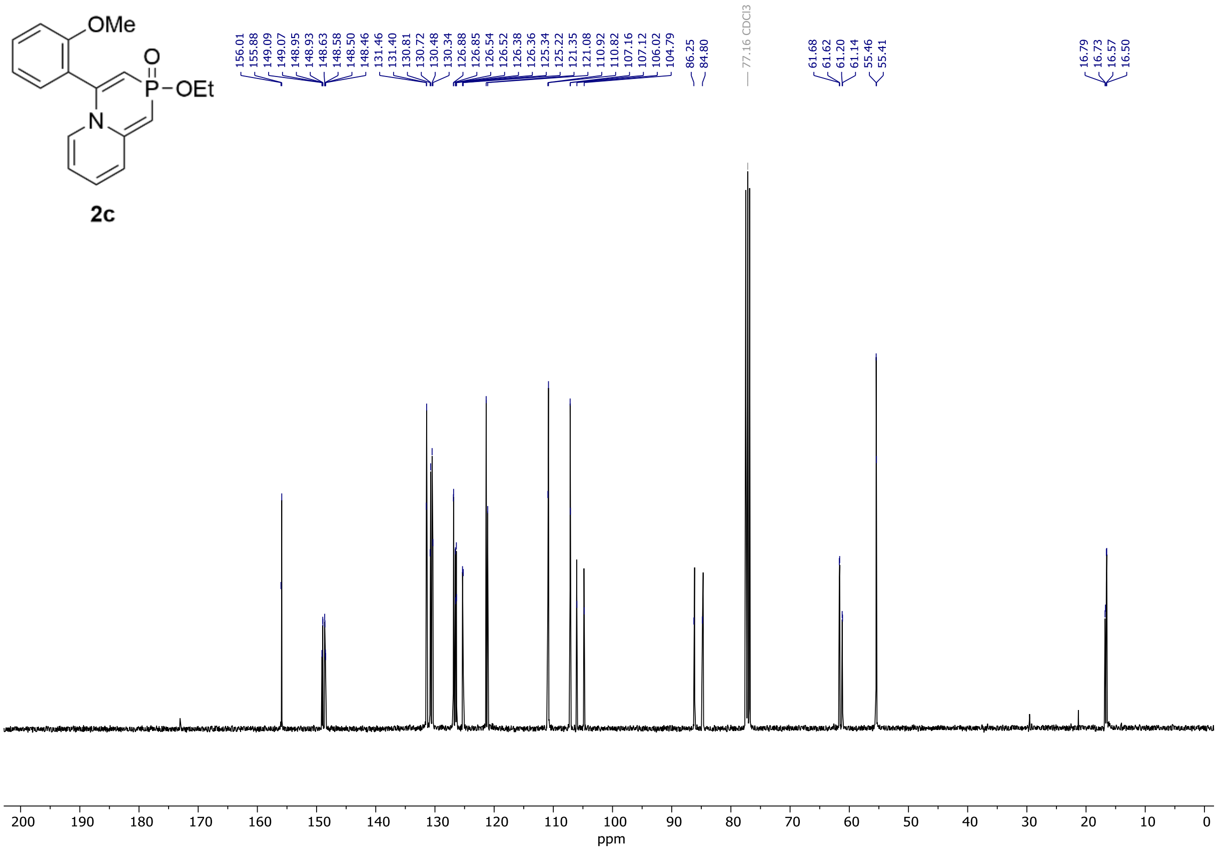

Figure S77 ^{13}C {^{1}H} *NMR spectrum of* ***2c*** *(101 MHz, $CDCl_3$).*

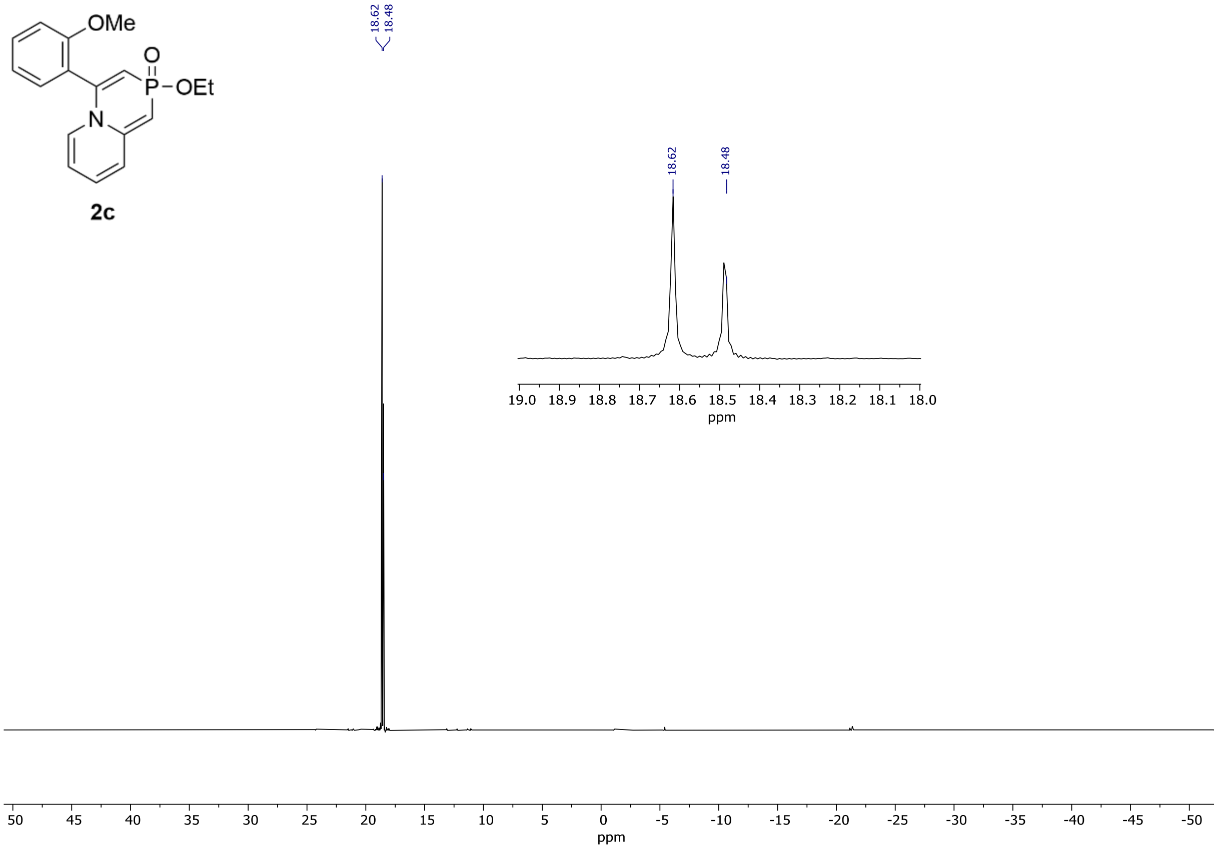

Figure S78 ^{31}P {^{1}H} *NMR spectrum of* ***2c*** *(162 MHz, $CDCl_3$).*

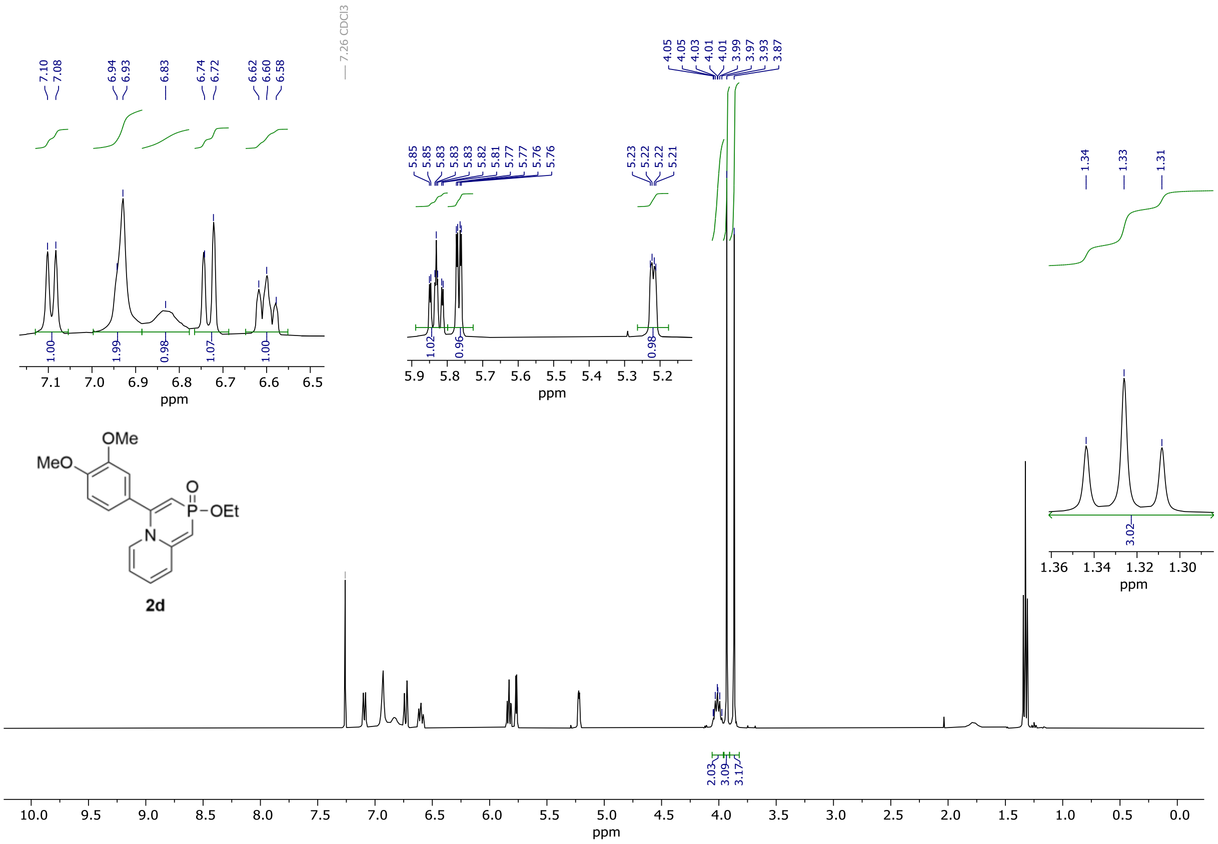

Figure S79 ^{1}H *NMR spectrum of* ***2d*** *(400 MHz, $CDCl_3$).*

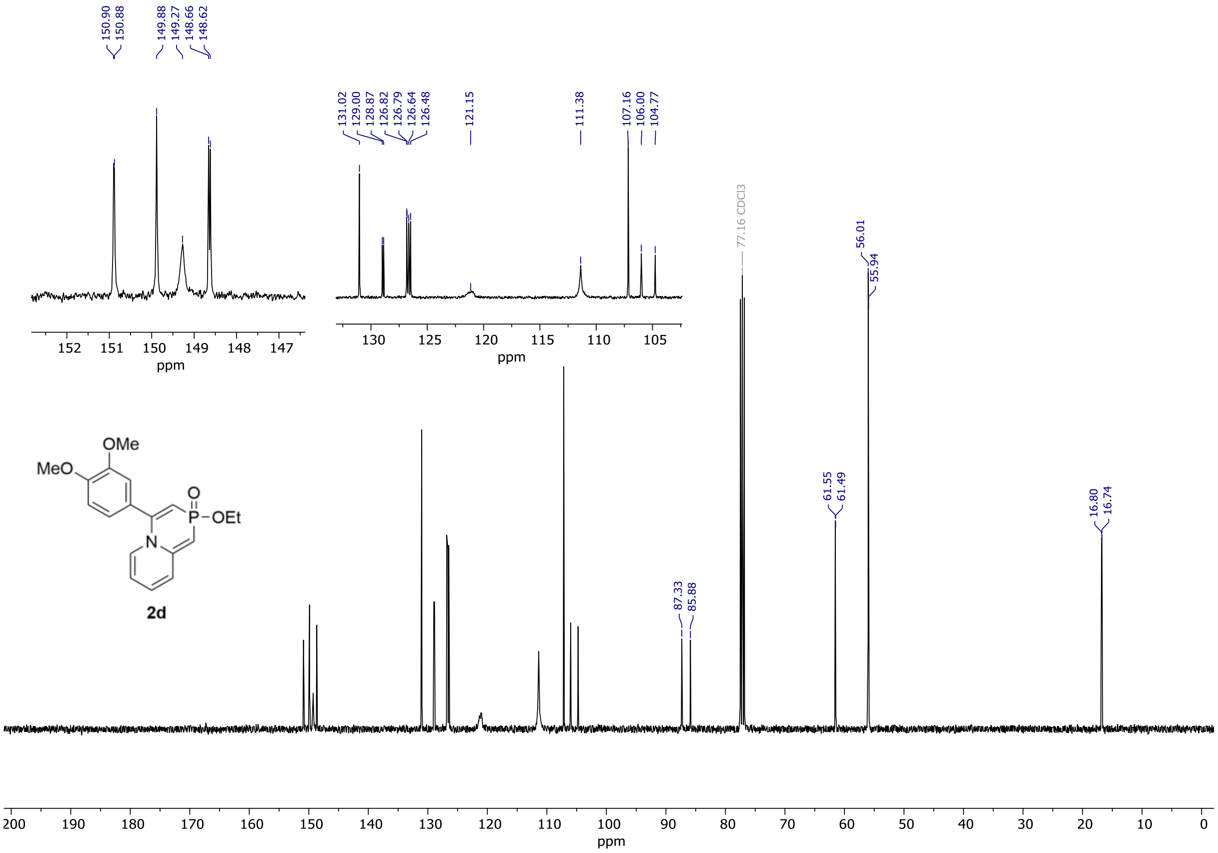

Figure S80 $^{13}C\ \{^{1}H\}$ *NMR spectrum of* ***2d*** *(101 MHz, $CDCl_3$).*

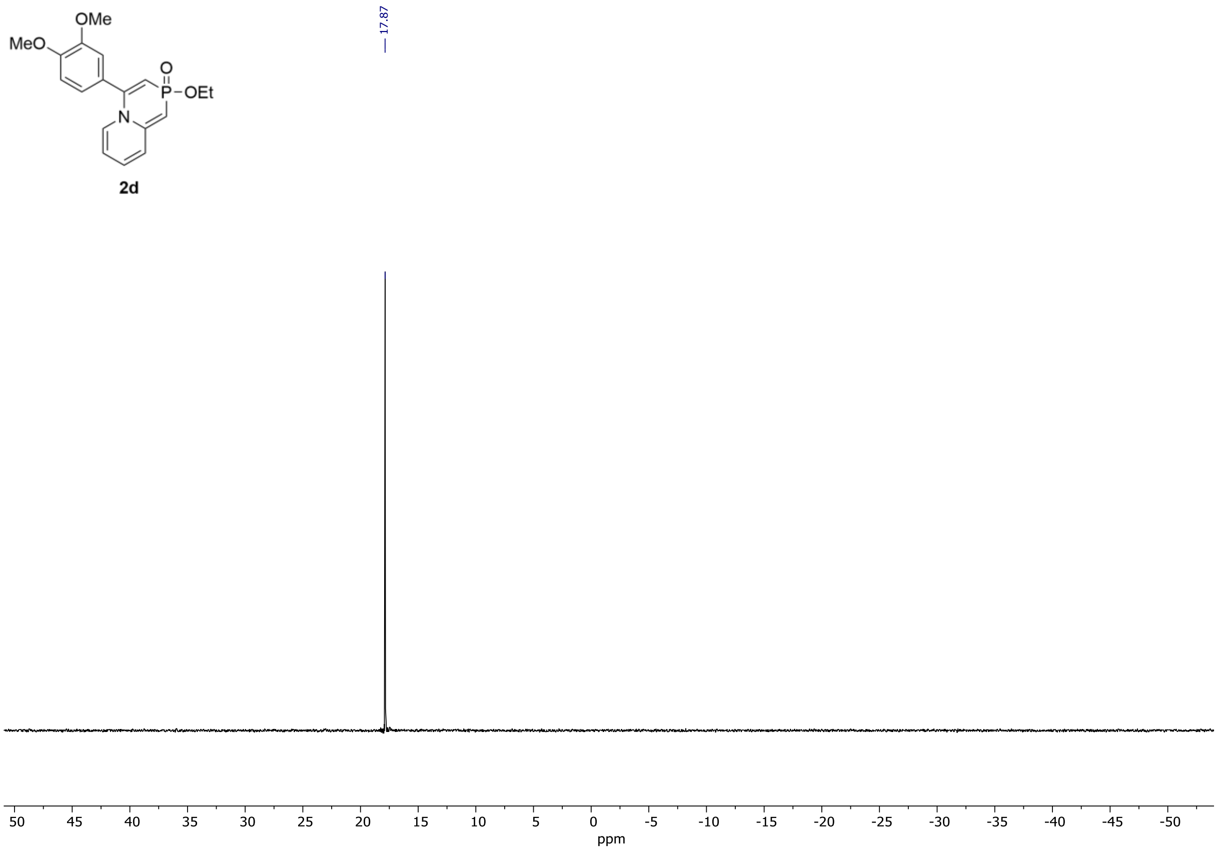

Figure S81 *^{31}P {^{1}H} NMR spectrum of* ***2d*** *(162 MHz, CDCl$_3$).*

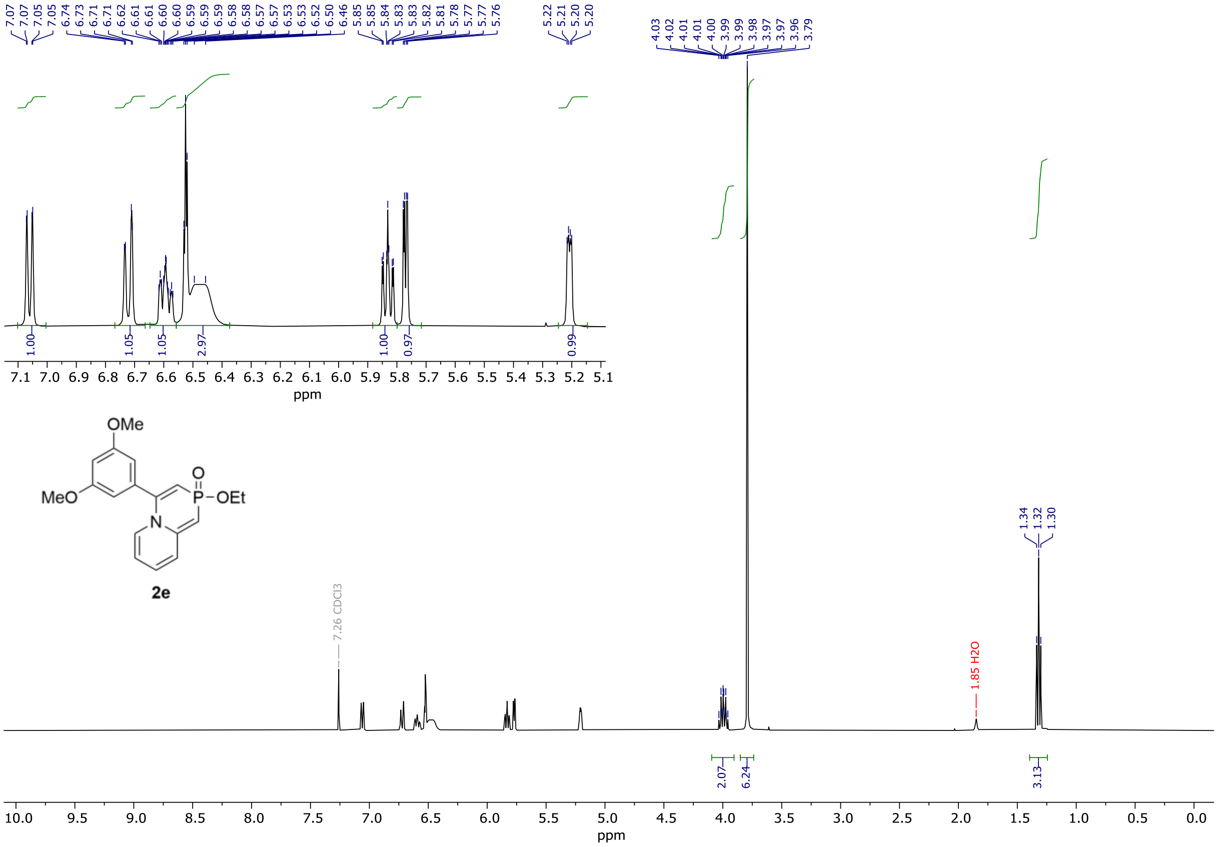

Figure S82 *^{31}P {^{1}H} NMR spectrum of* ***2e*** *(162 MHz, CDCl$_3$).*

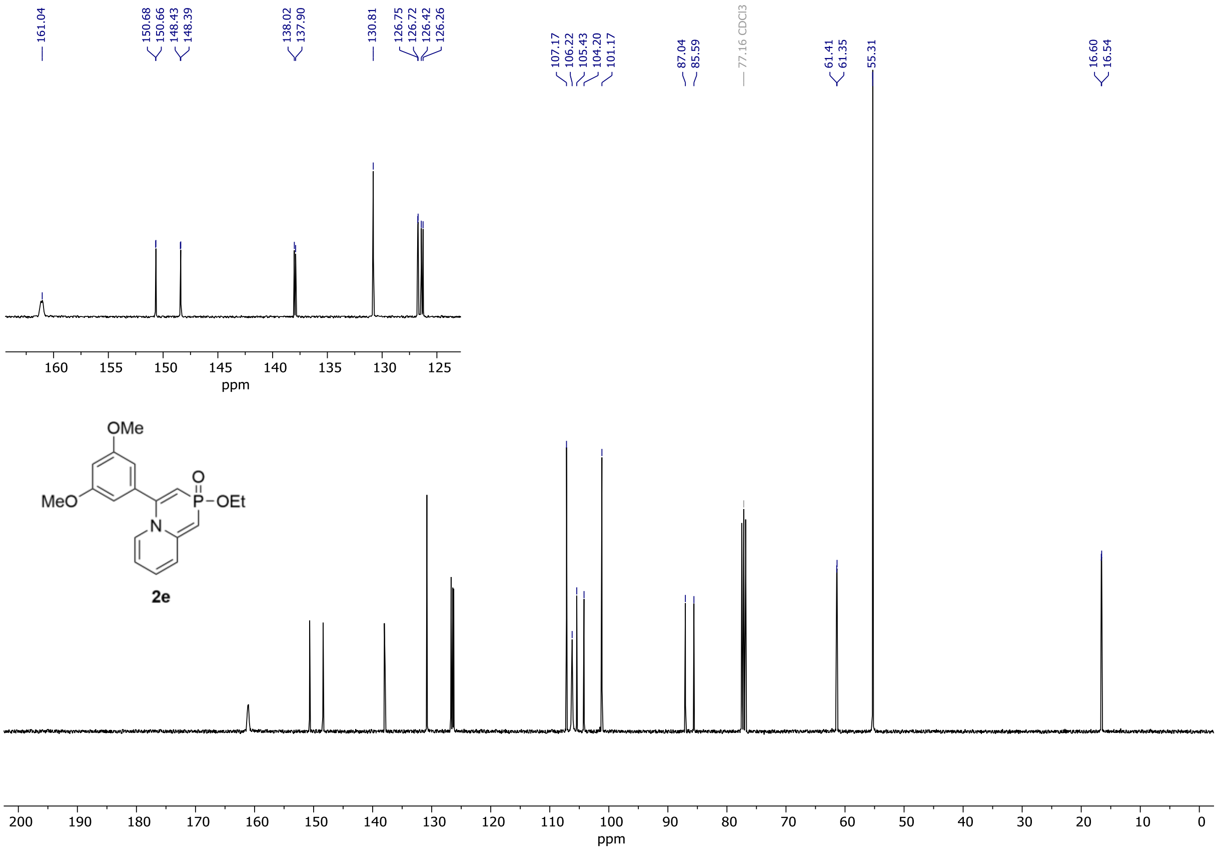

Figure S83 *^{13}C {^{1}H} NMR spectrum of **2e** (101 MHz, $CDCl_3$).*

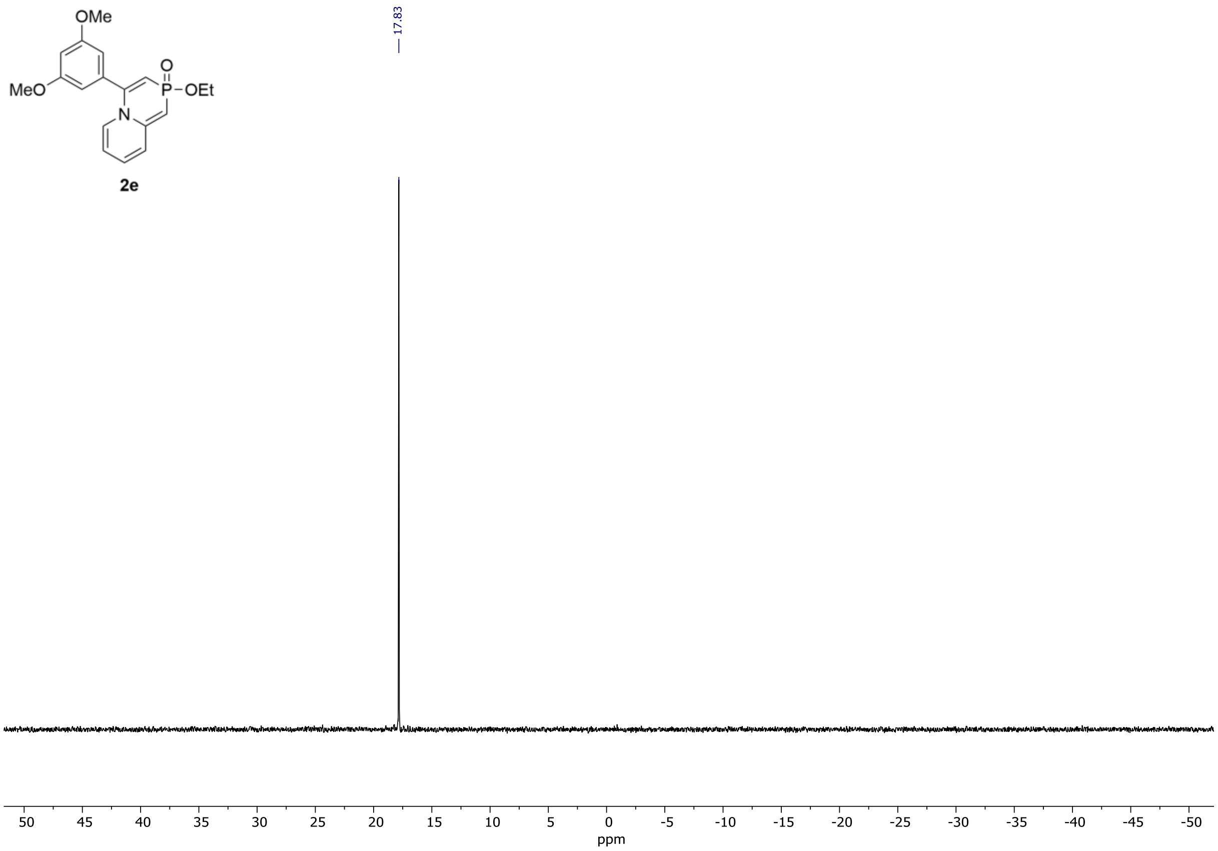

Figure S84 *^{31}P {^{1}H} NMR spectrum of **2e** (162 MHz, $CDCl_3$).*

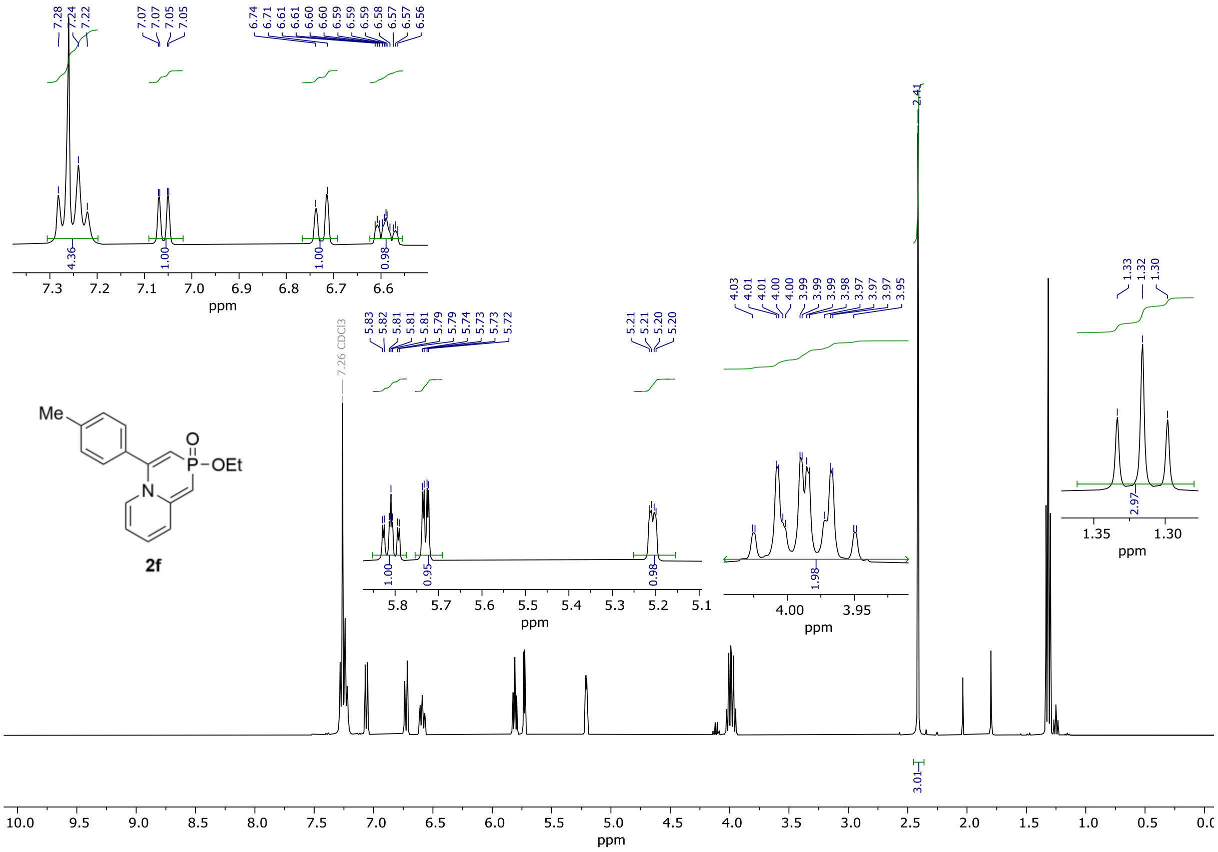

Figure S85 *^{1}H NMR spectrum of **2f** (400 MHz, $CDCl_3$).*

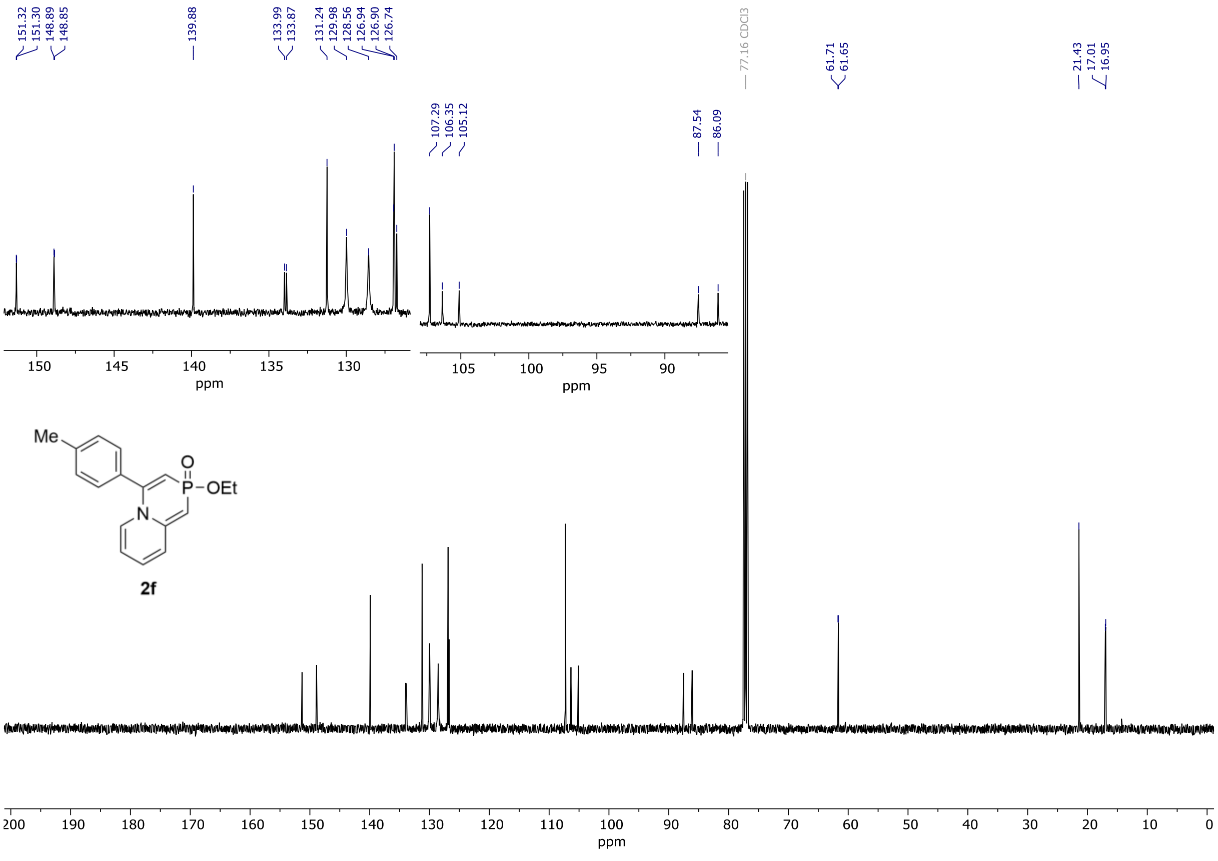

Figure S86 *^{13}C {^{1}H} NMR spectrum of **2f** (101 MHz, $CDCl_3$).*

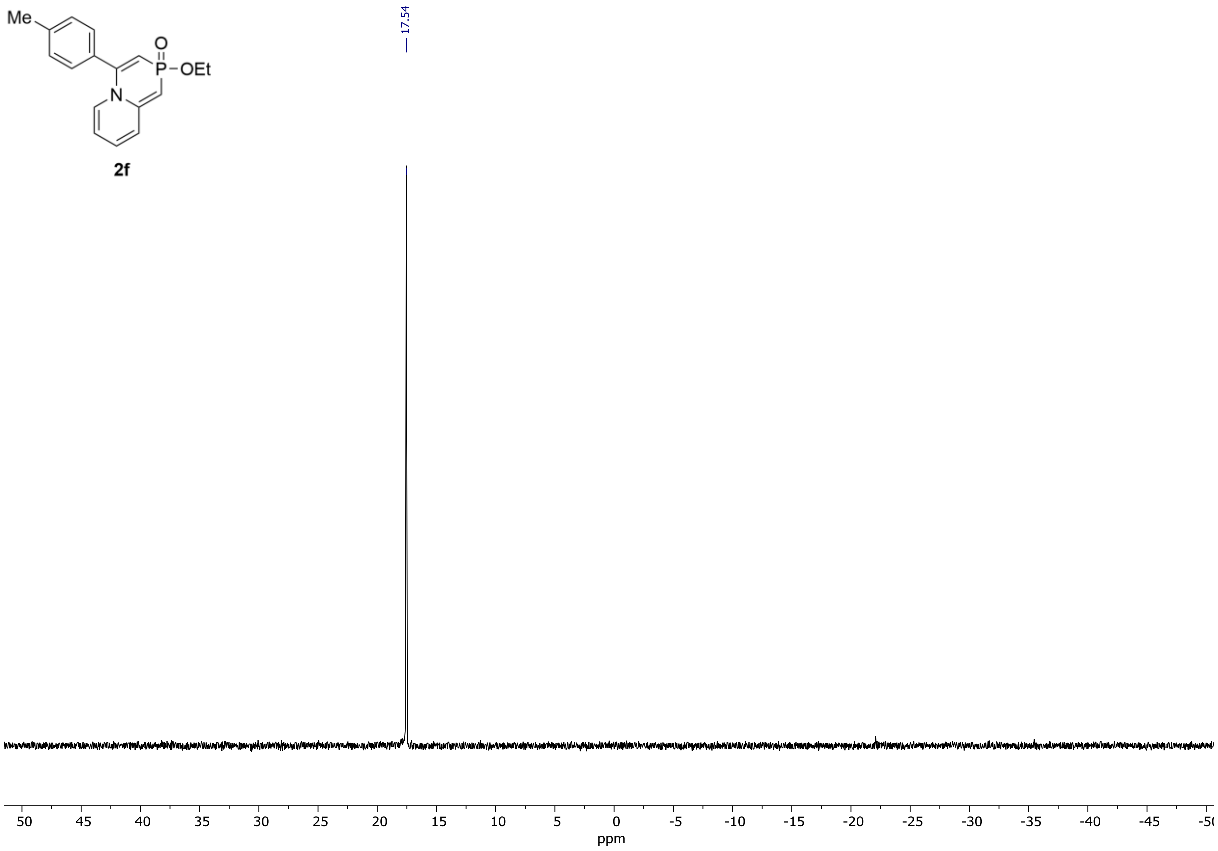

Figure S87 ^{31}P {^{1}H} NMR spectrum of ***2f*** (376 MHz, CDCl$_3$).

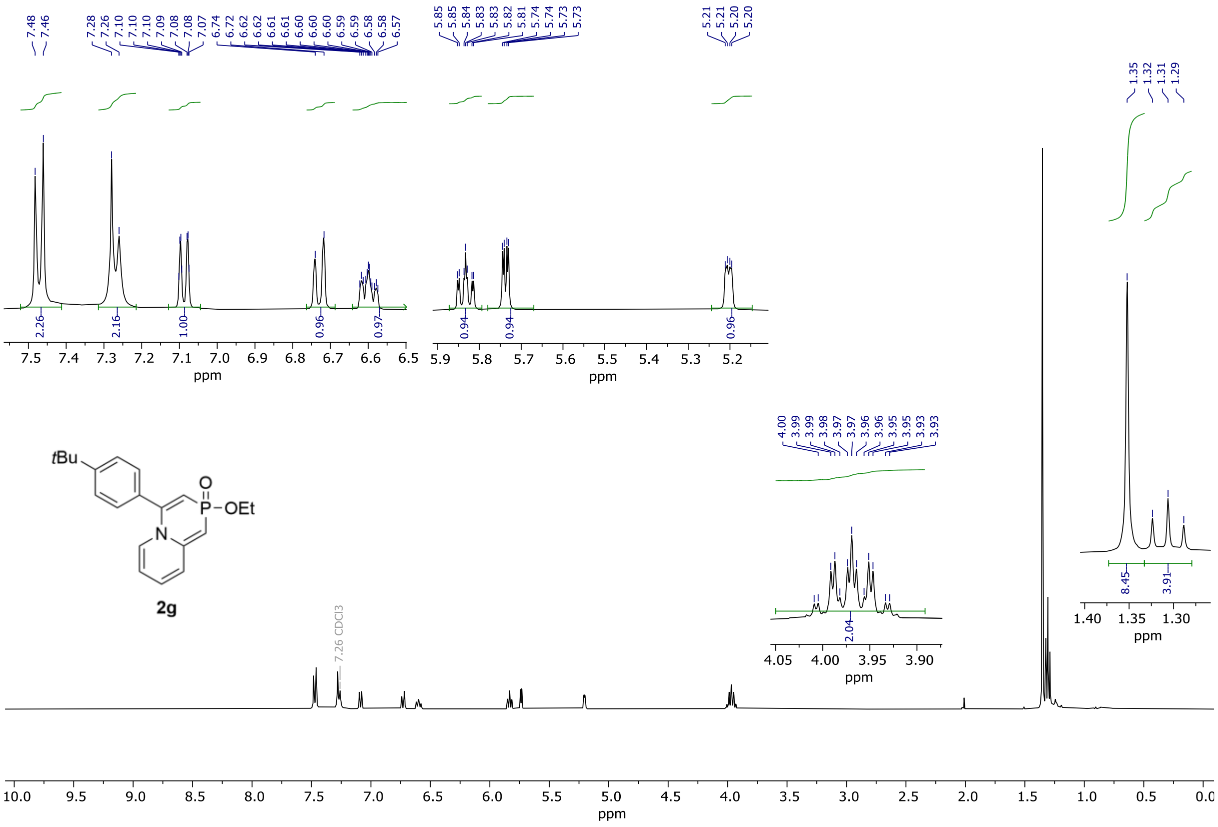

Figure S88 ^{1}H NMR spectrum of ***2g*** (400 MHz, CDCl$_3$).

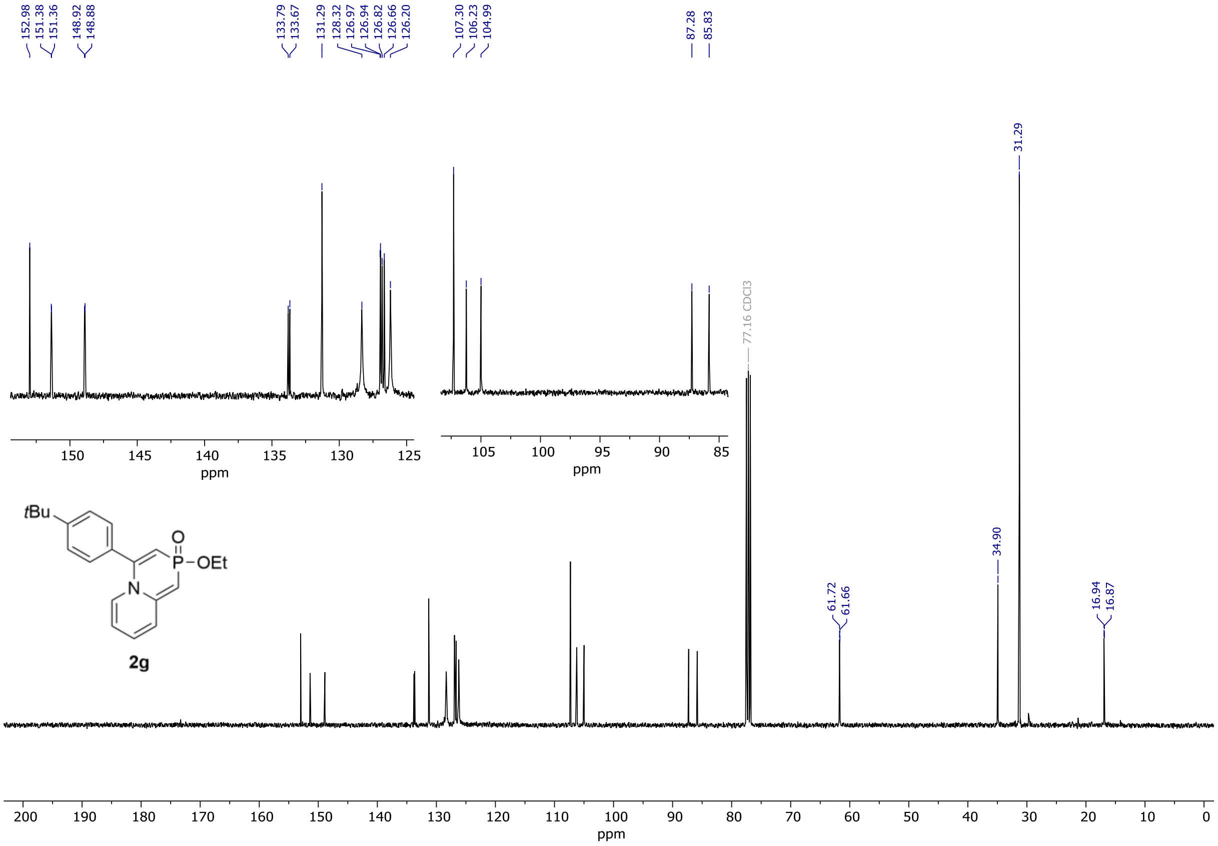

Figure S89 *^{13}C{^{1}H} NMR of **2g** (101 MHz, $CDCl_3$).*

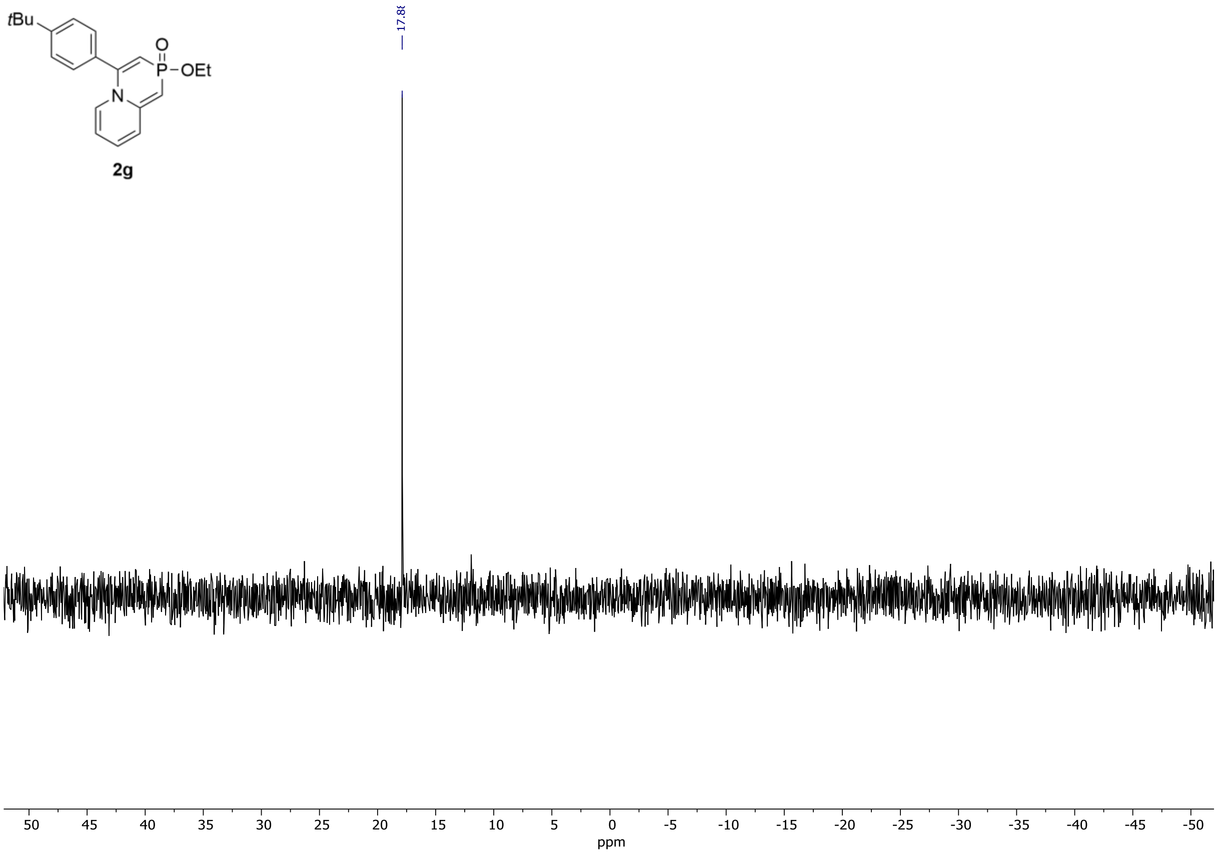

Figure S90 *^{31}P {^{1}H} NMR spectrum of **2g** (376 MHz, $CDCl_3$).*

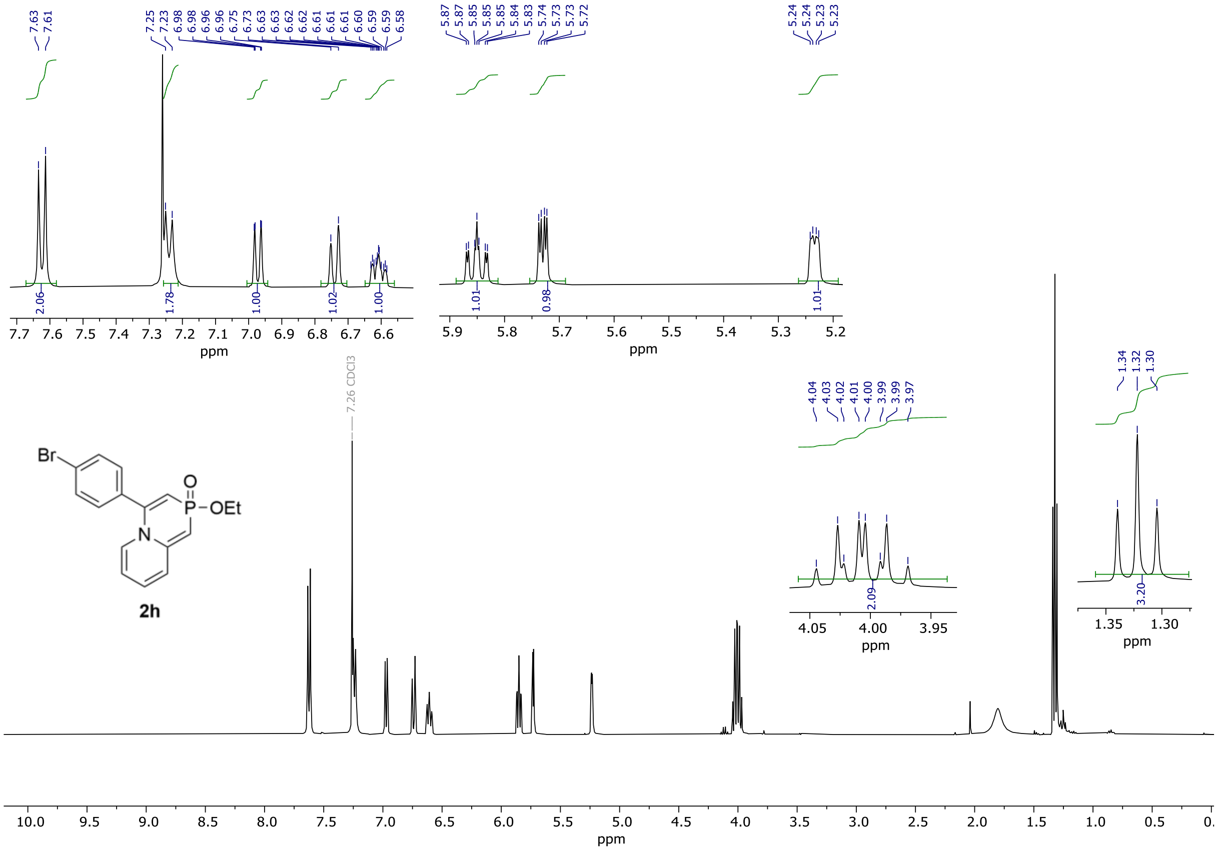

Figure S91 [1]*H NMR spectrum of* ***2h*** *(400 MHz, CDCl*$_3$*).*

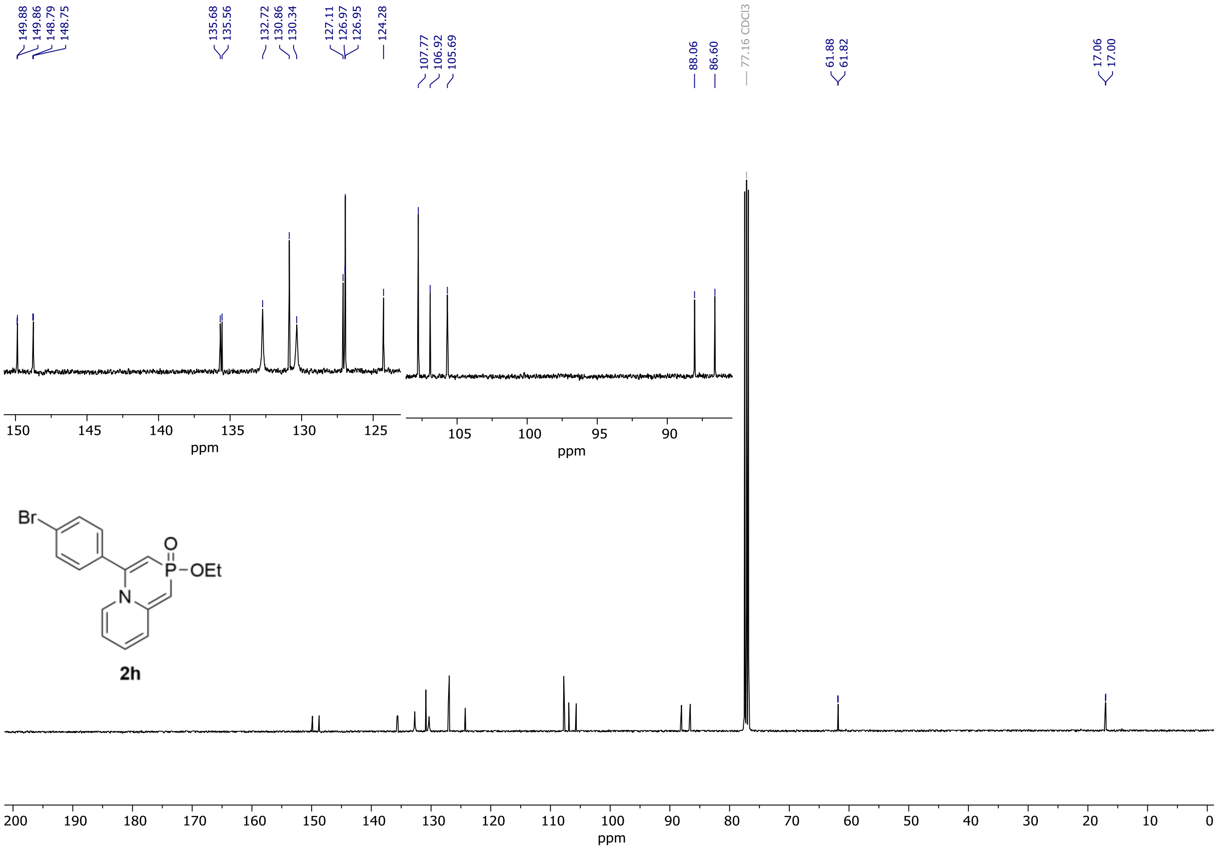

Figure S92 [13]*C {*[1]*H} NMR spectrum of* ***2h*** *(101 MHz, CDCl*$_3$*).*

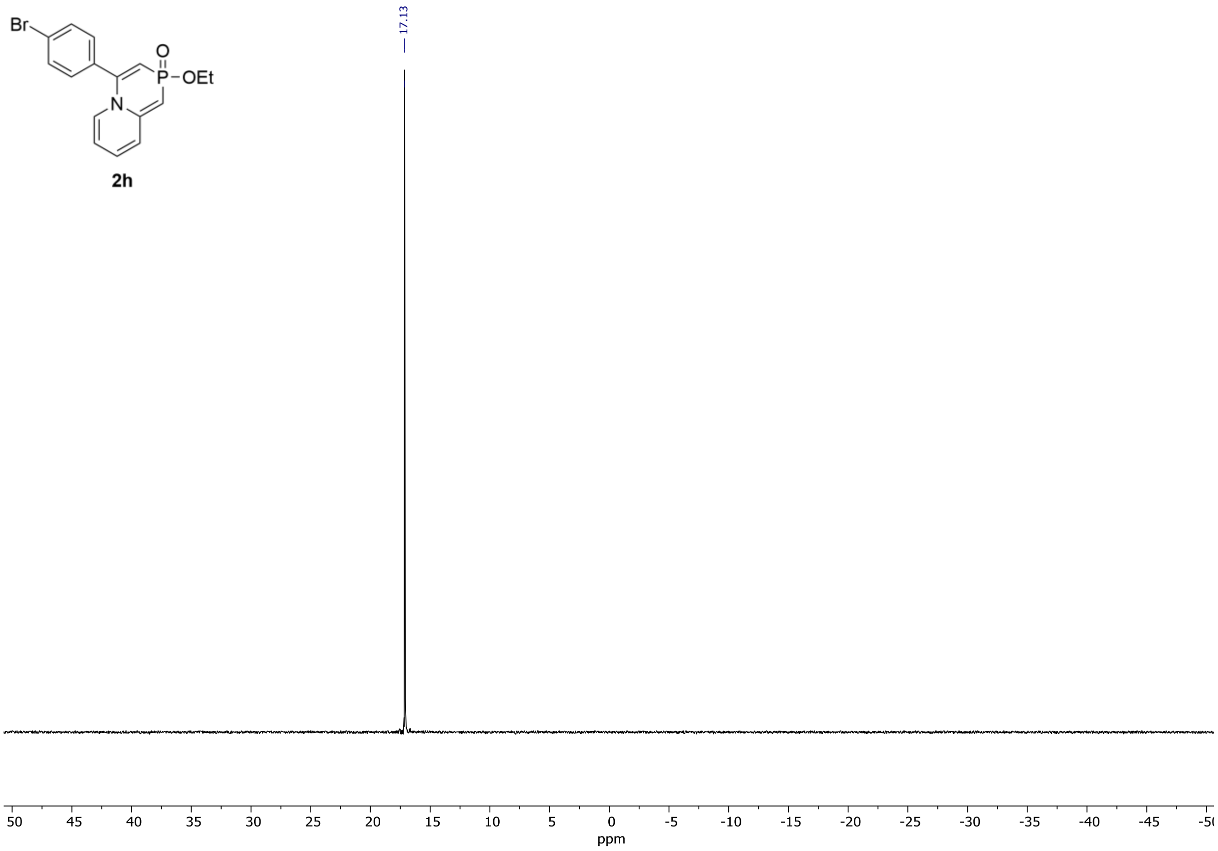

Figure S93 $^{31}P\ \{^{1}H\}$ *NMR spectrum of* ***2h*** *(376 MHz, $CDCl_3$).*

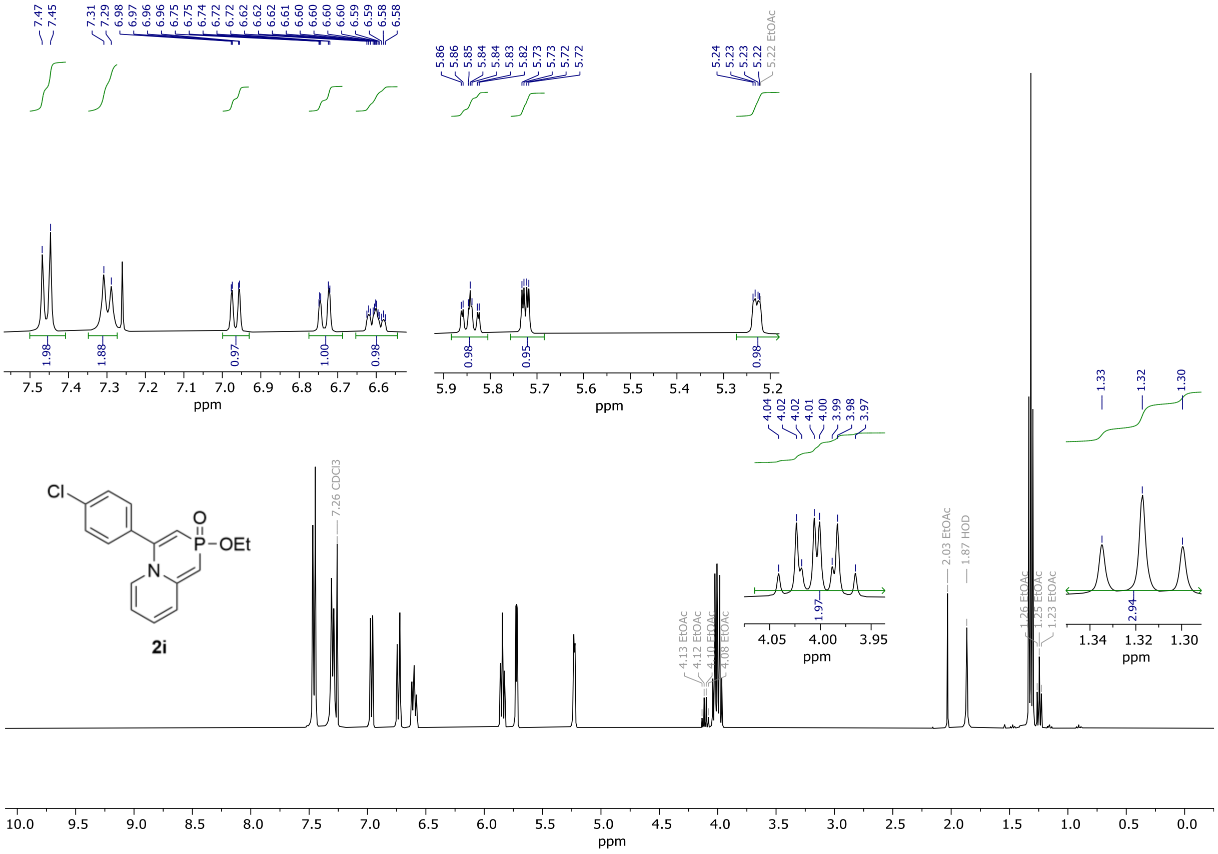

Figure S94 ^{1}H *NMR spectrum of* ***2i*** *(400 MHz, $CDCl_3$).*

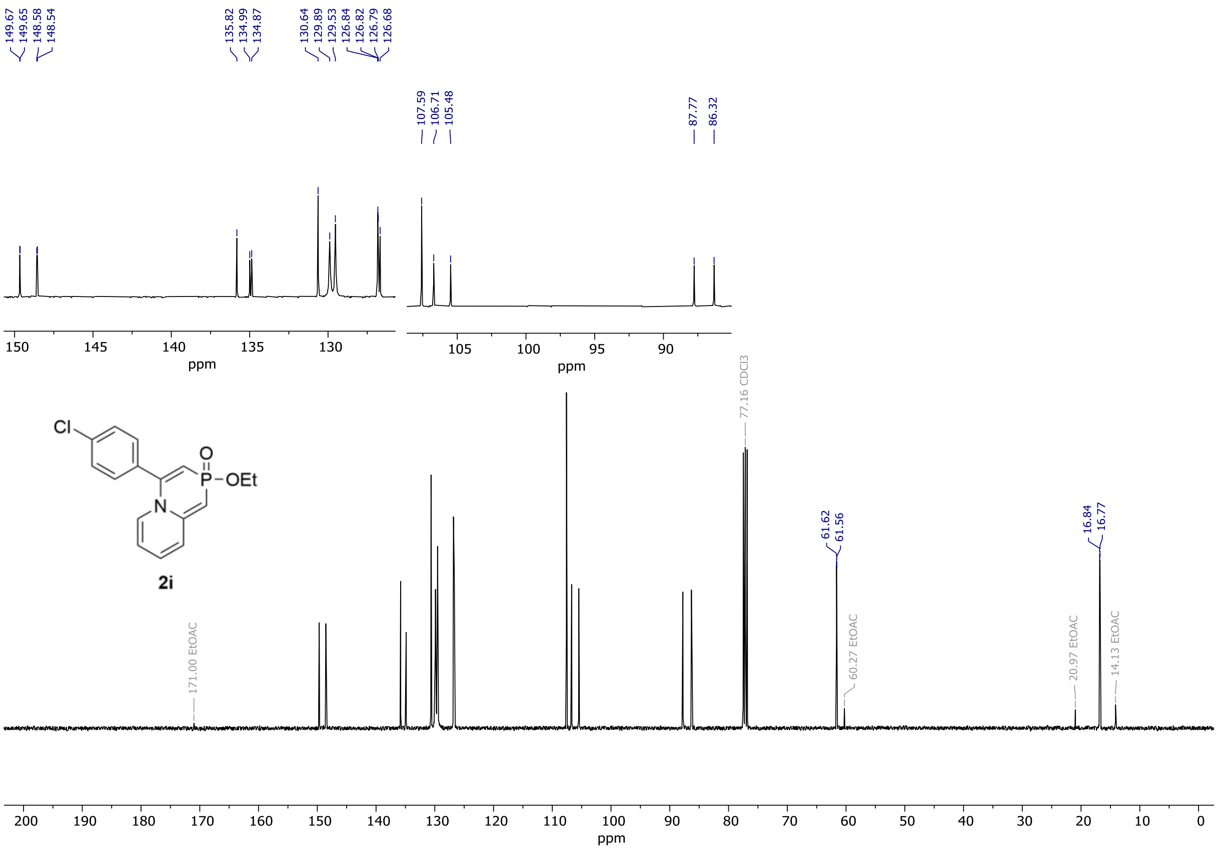

Figure S95 *^{13}C {^{1}H} NMR spectrum of **2i** (101 MHz, $CDCl_3$).*

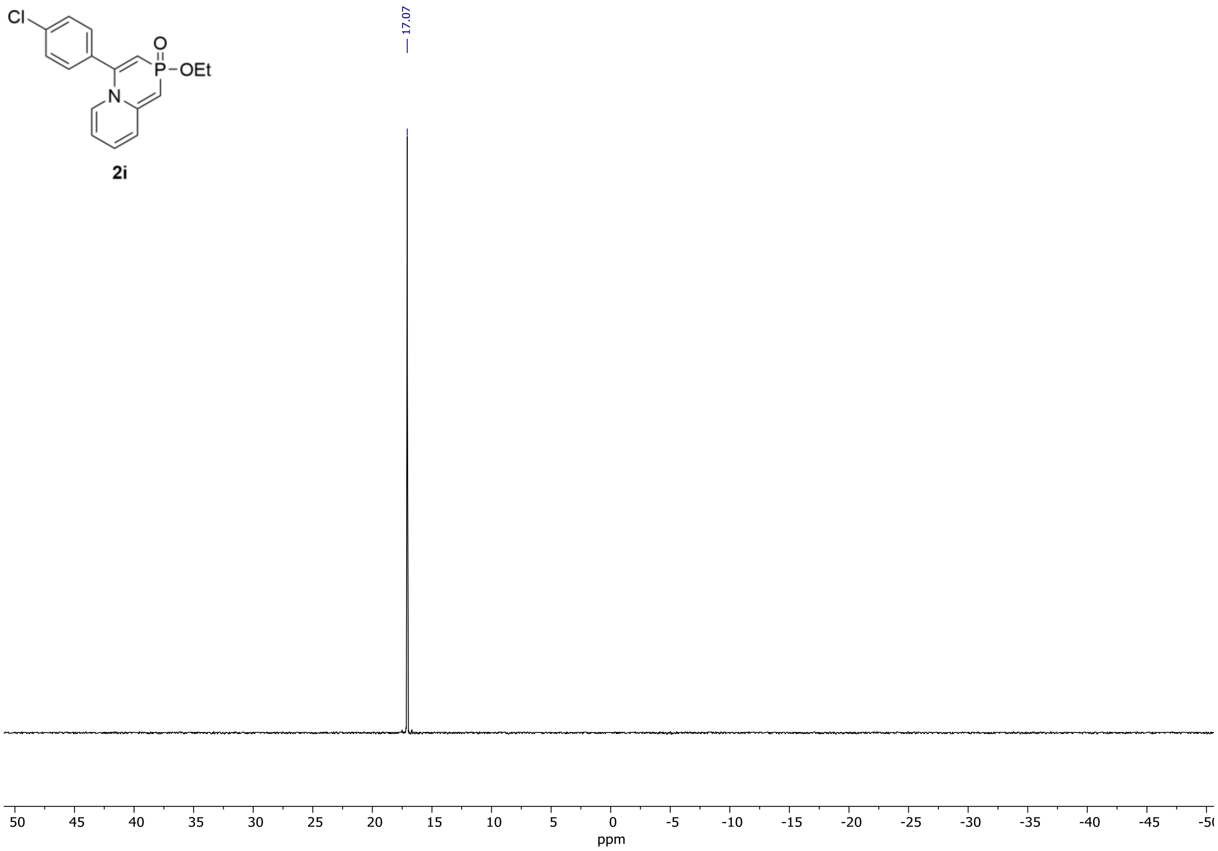

Figure S96 *^{31}P {^{1}H} NMR spectrum of **2i** (376 MHz, $CDCl_3$).*

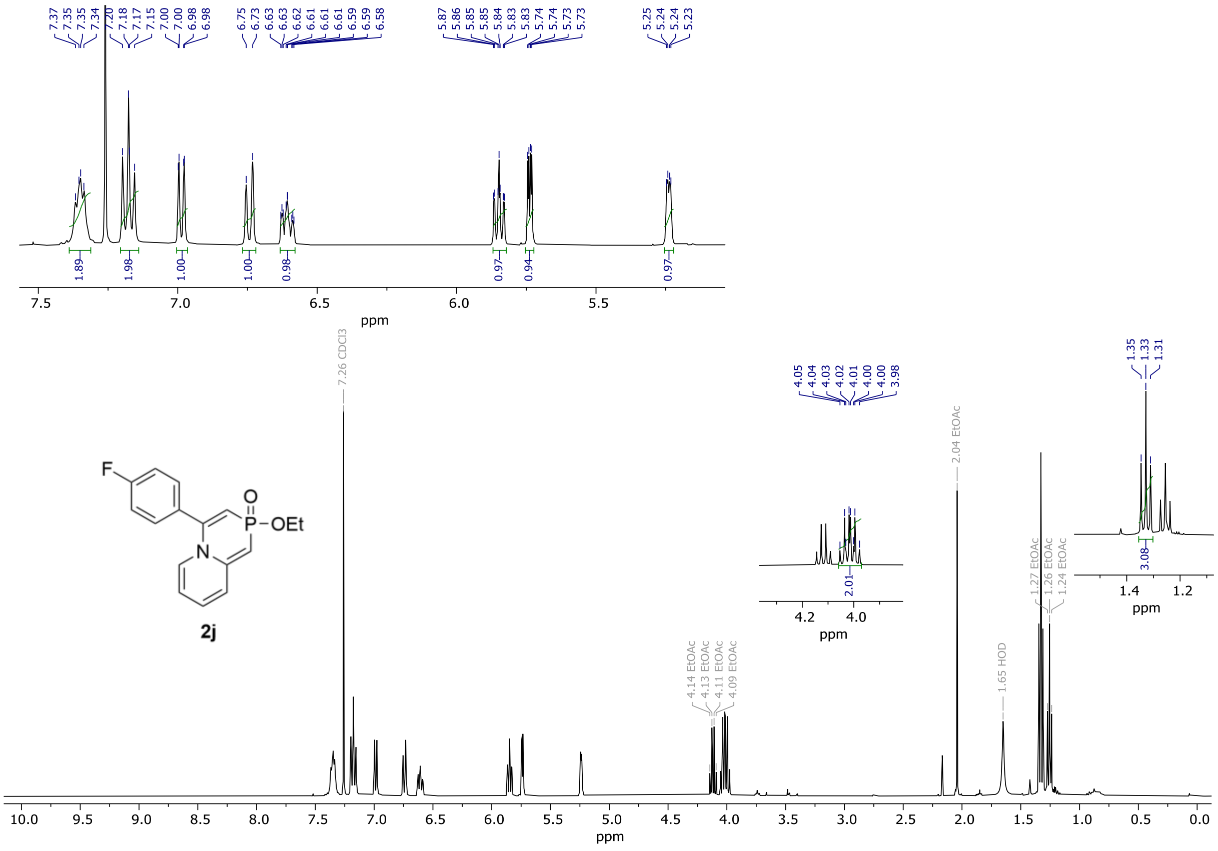

Figure S97 ¹H NMR spectrum of **2j** (400 MHz, $CDCl_3$).

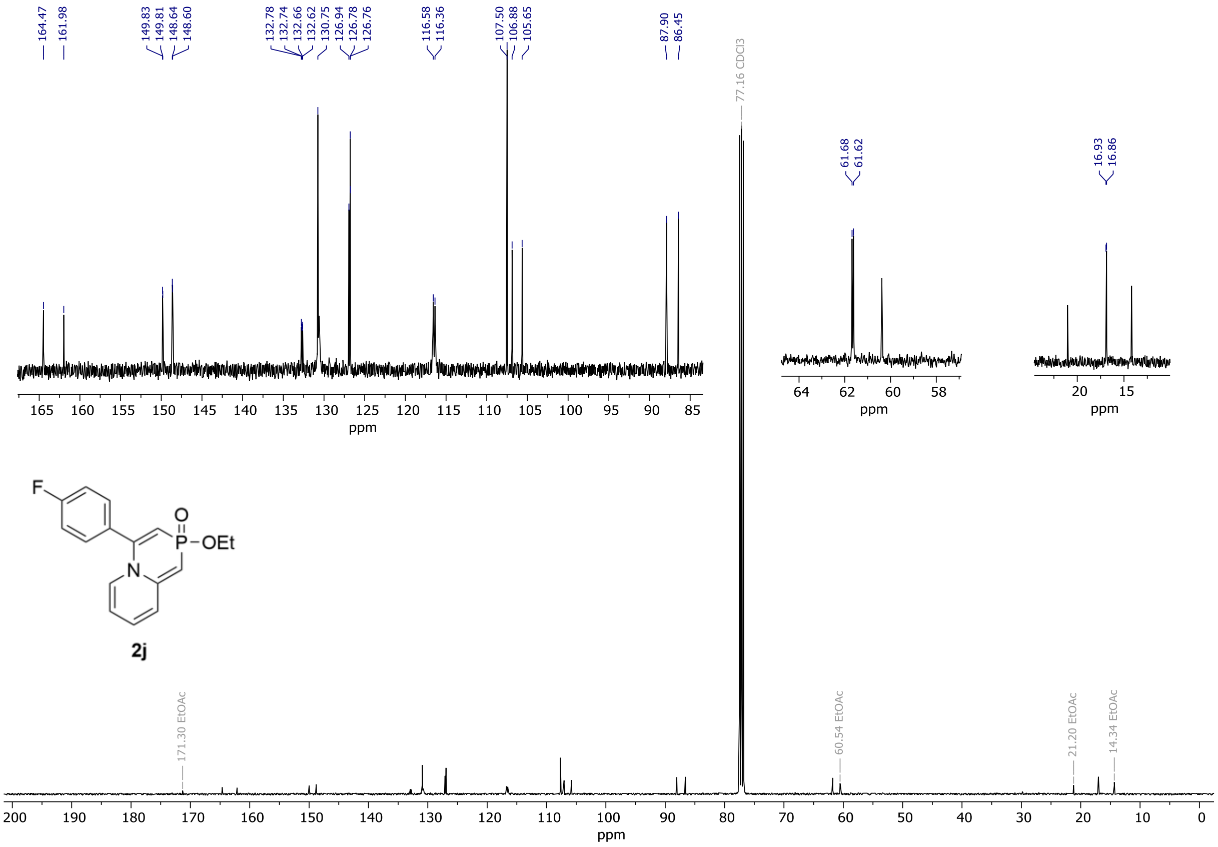

Figure S98 ^{13}C {¹H} NMR spectrum of **2j** (101 MHz, $CDCl_3$).

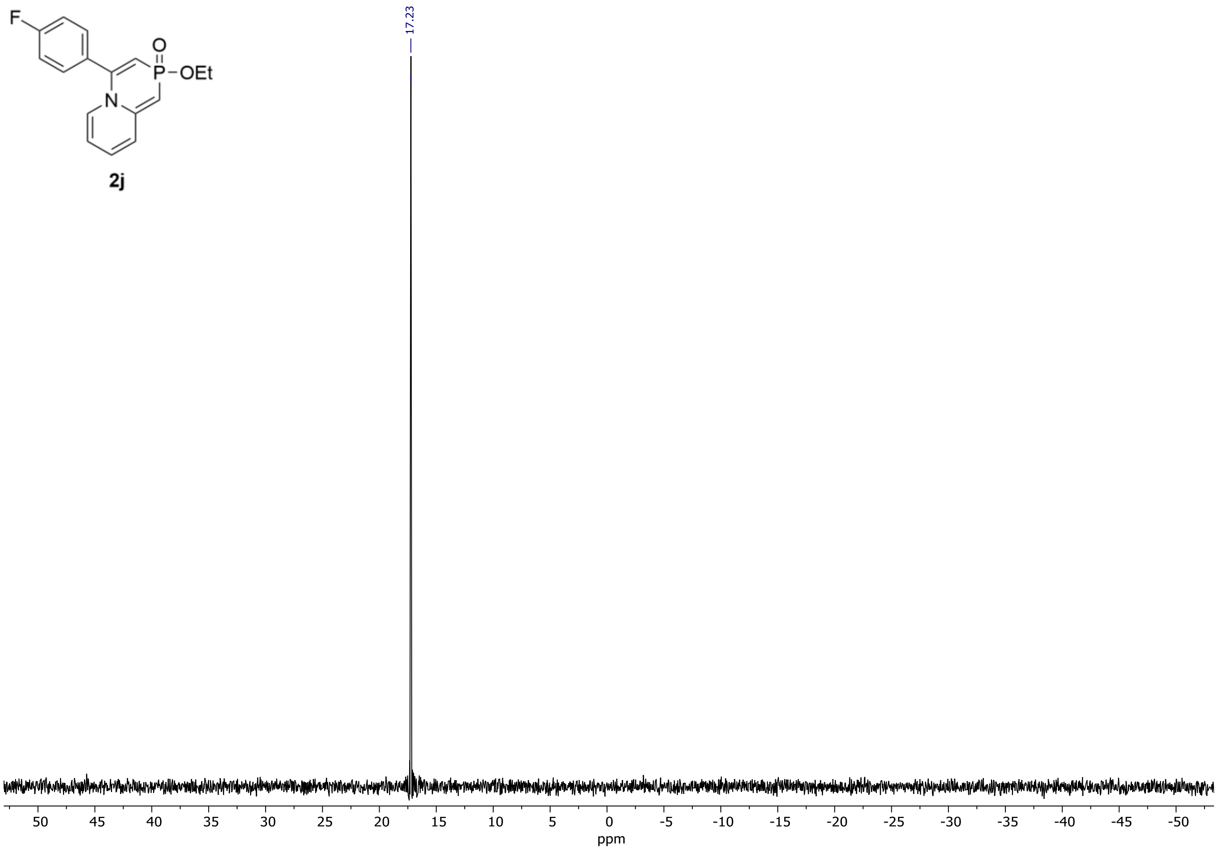

Figure S99 *^{31}P {^{1}H} NMR spectrum of **2j** (162 MHz, $CDCl_3$).*

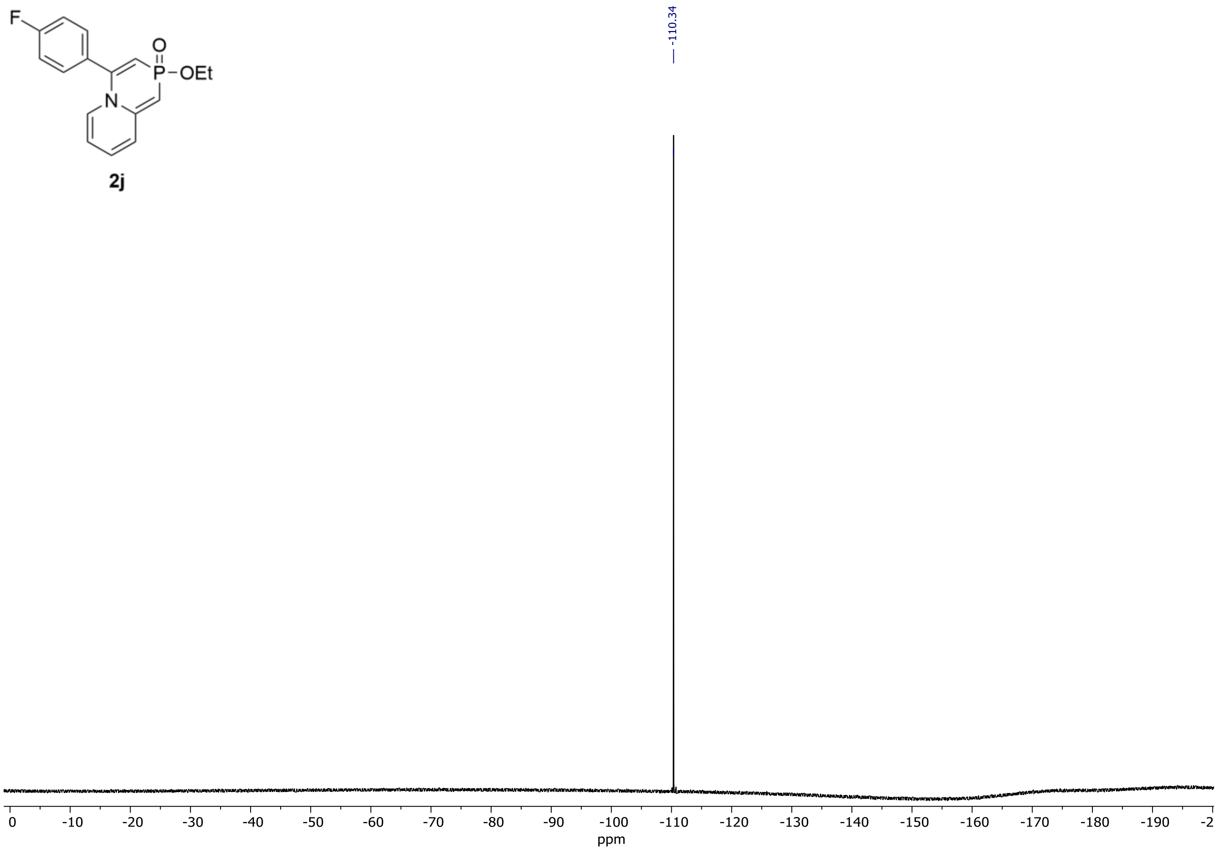

Figure S100 *^{19}F {^{1}H} NMR spectrum of **2j** (376 MHz, $CDCl_3$).*

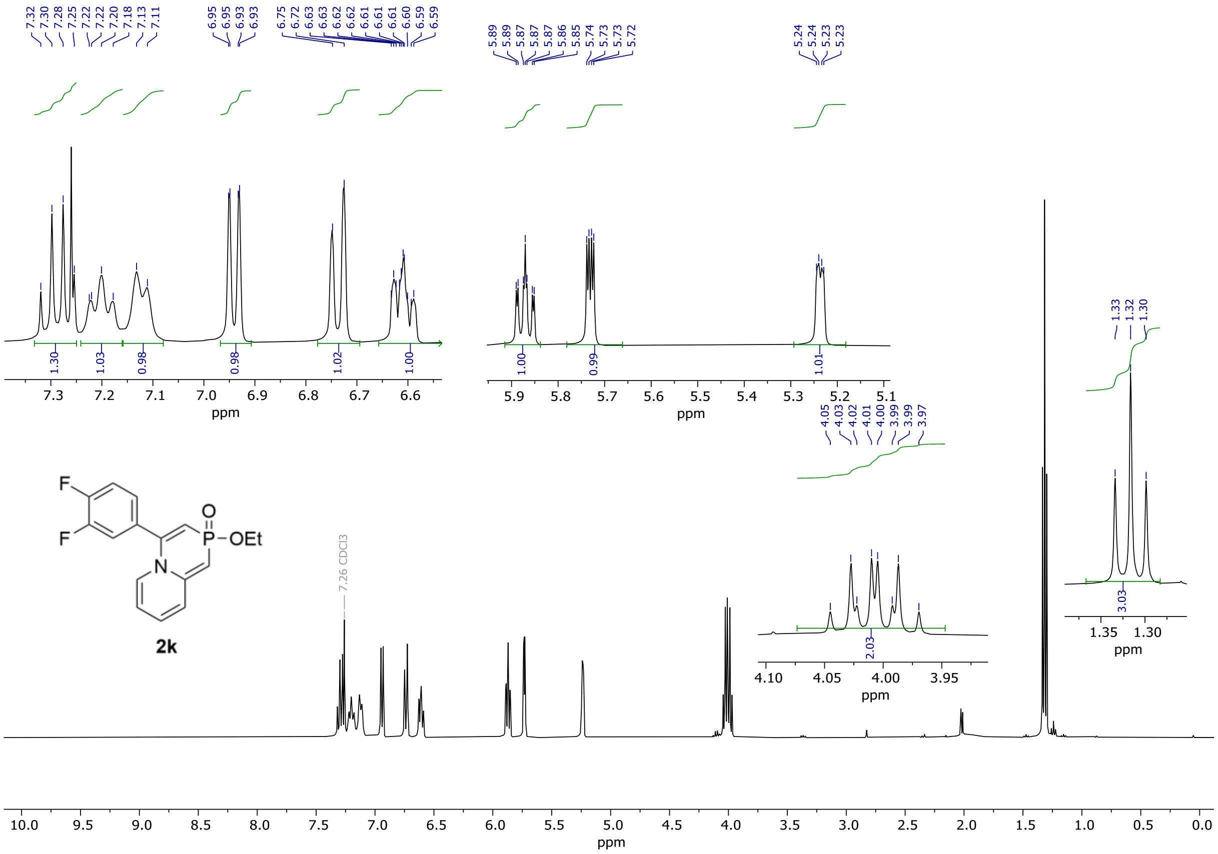

Figure S101 *^{1}H NMR spectrum of **2k** (400 MHz, $CDCl_3$).*

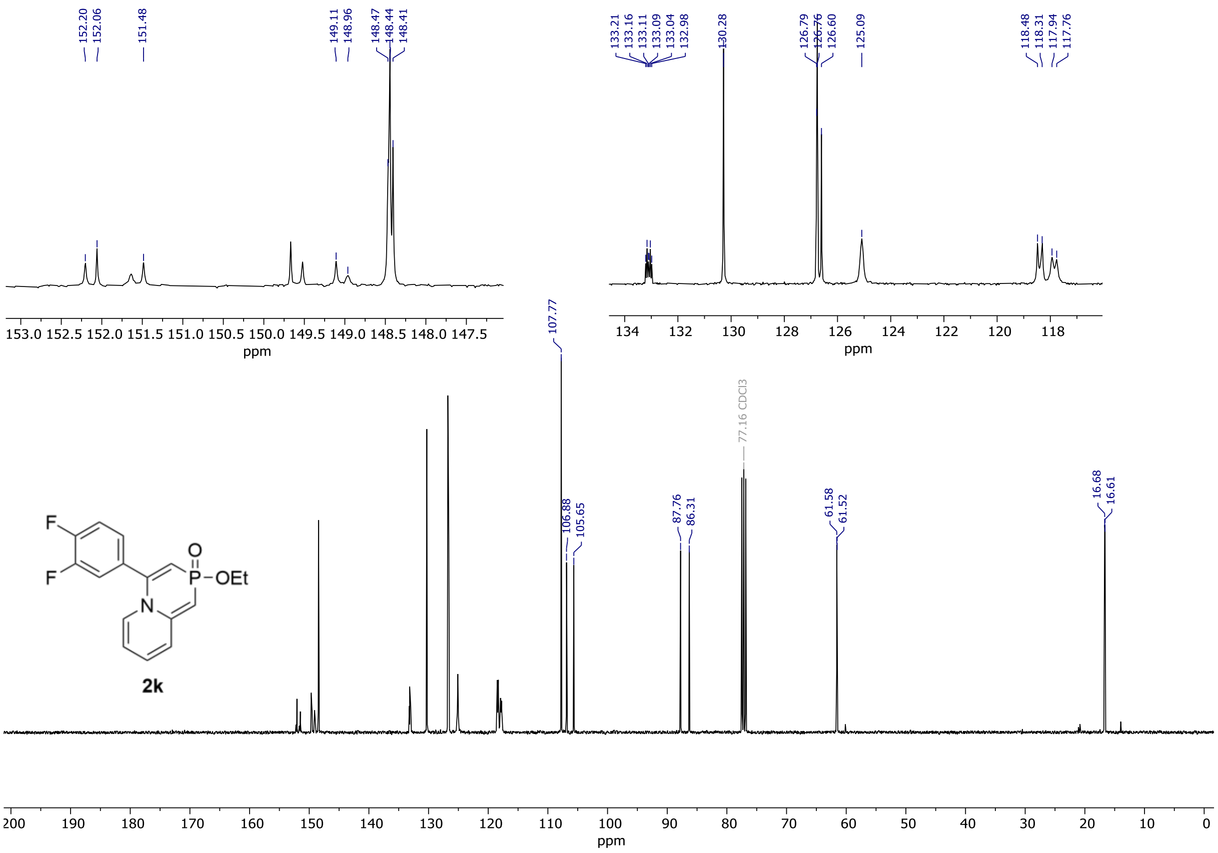

Figure S102 *^{13}C {^{1}H} NMR spectrum of **2k** (101 MHz, $CDCl_3$).*

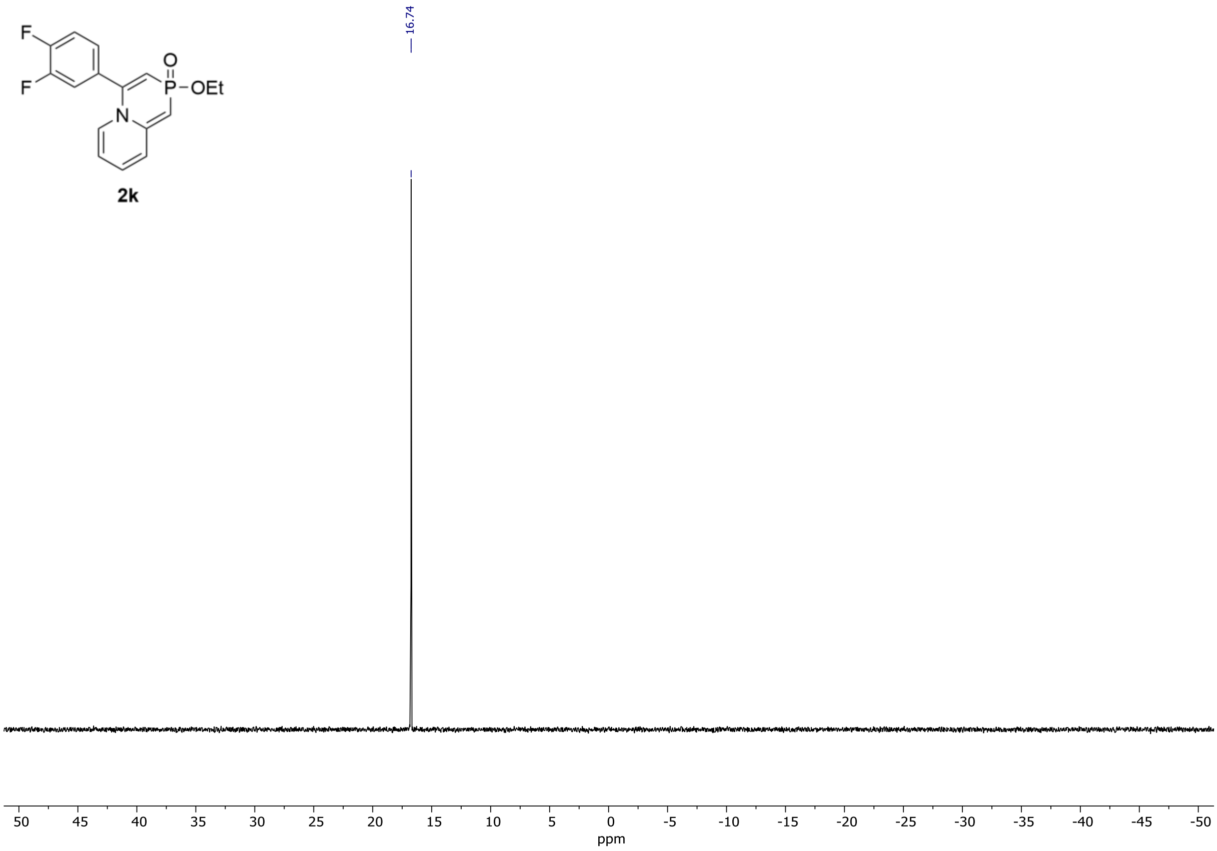

Figure S103 *^{31}P {^{1}H} NMR spectrum of **2k** (162 MHz, $CDCl_3$).*

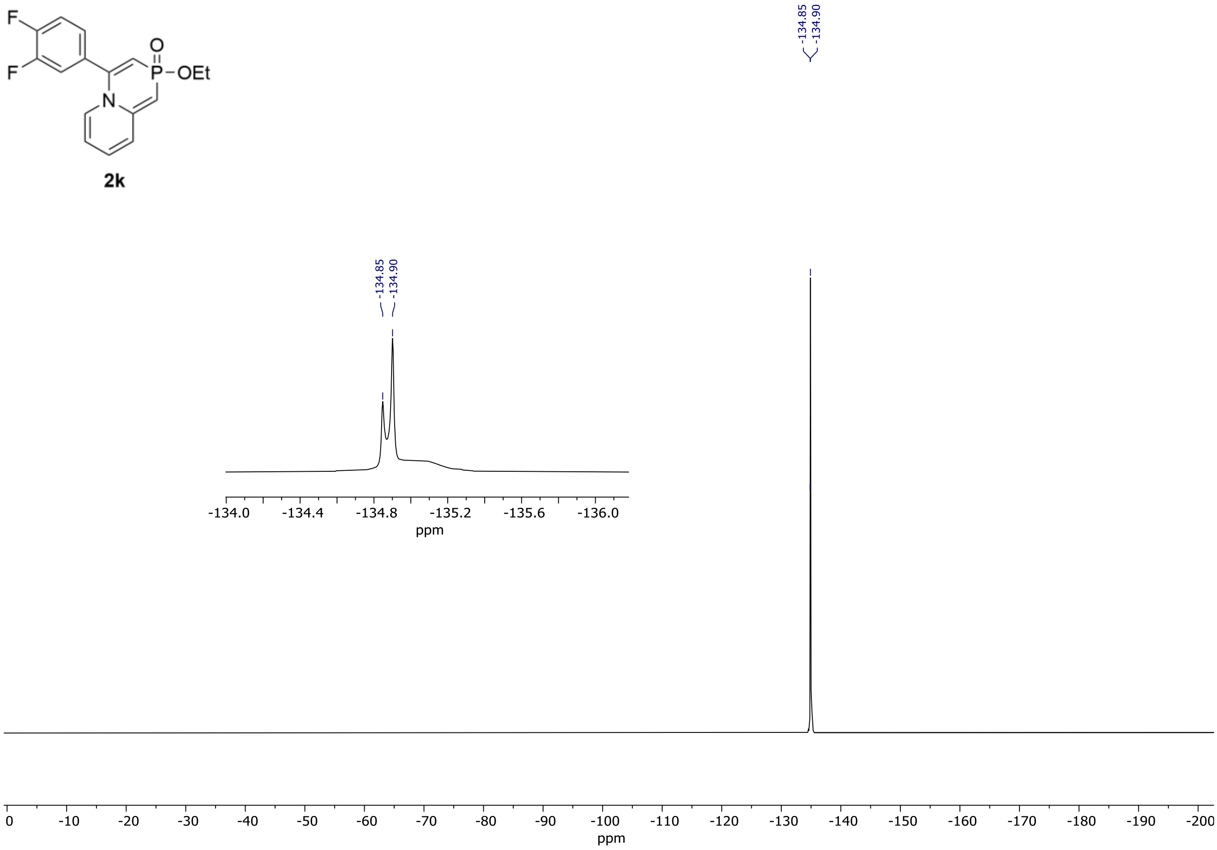

Figure S104 *^{19}F {^{1}H} NMR spectrum of **2k** (376 MHz, $CDCl_3$).*

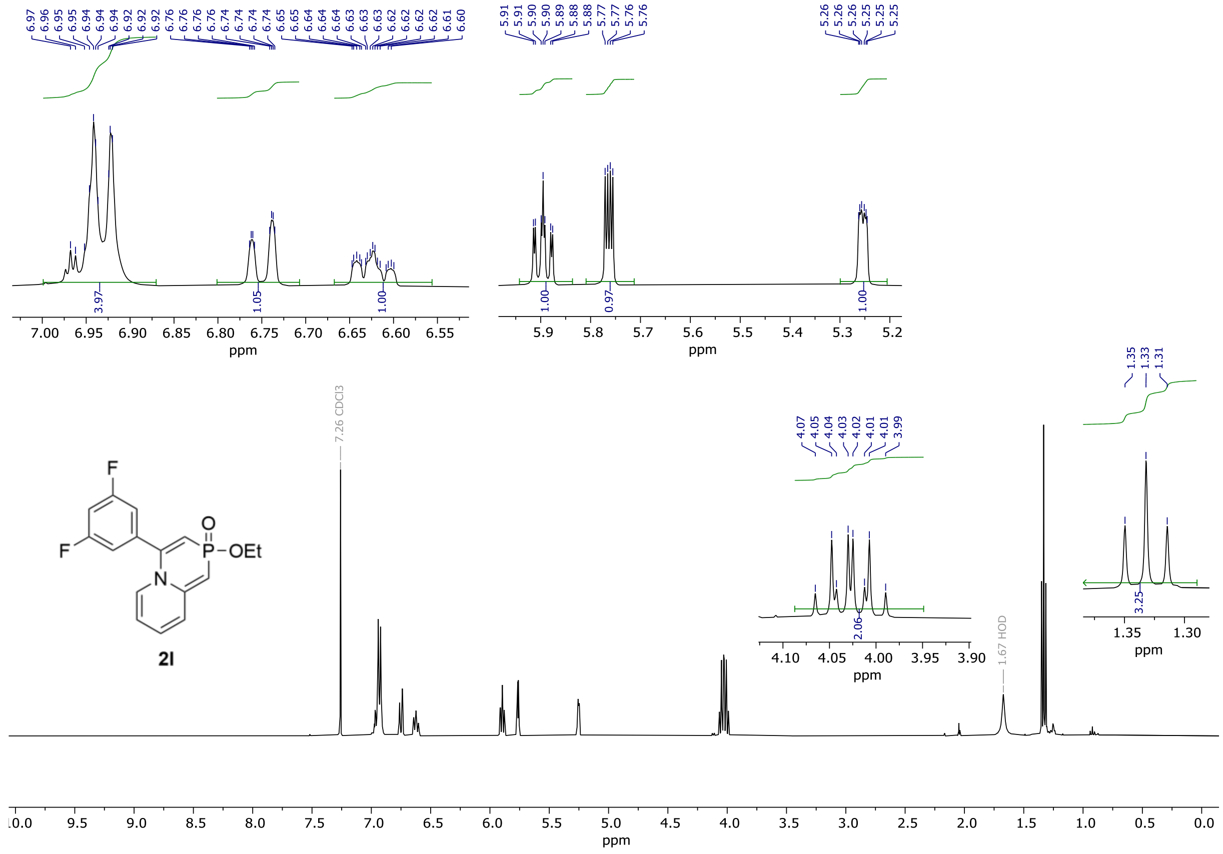

Figure S105 ^{1}H NMR spectrum of **2l** (400 MHz, $CDCl_3$).

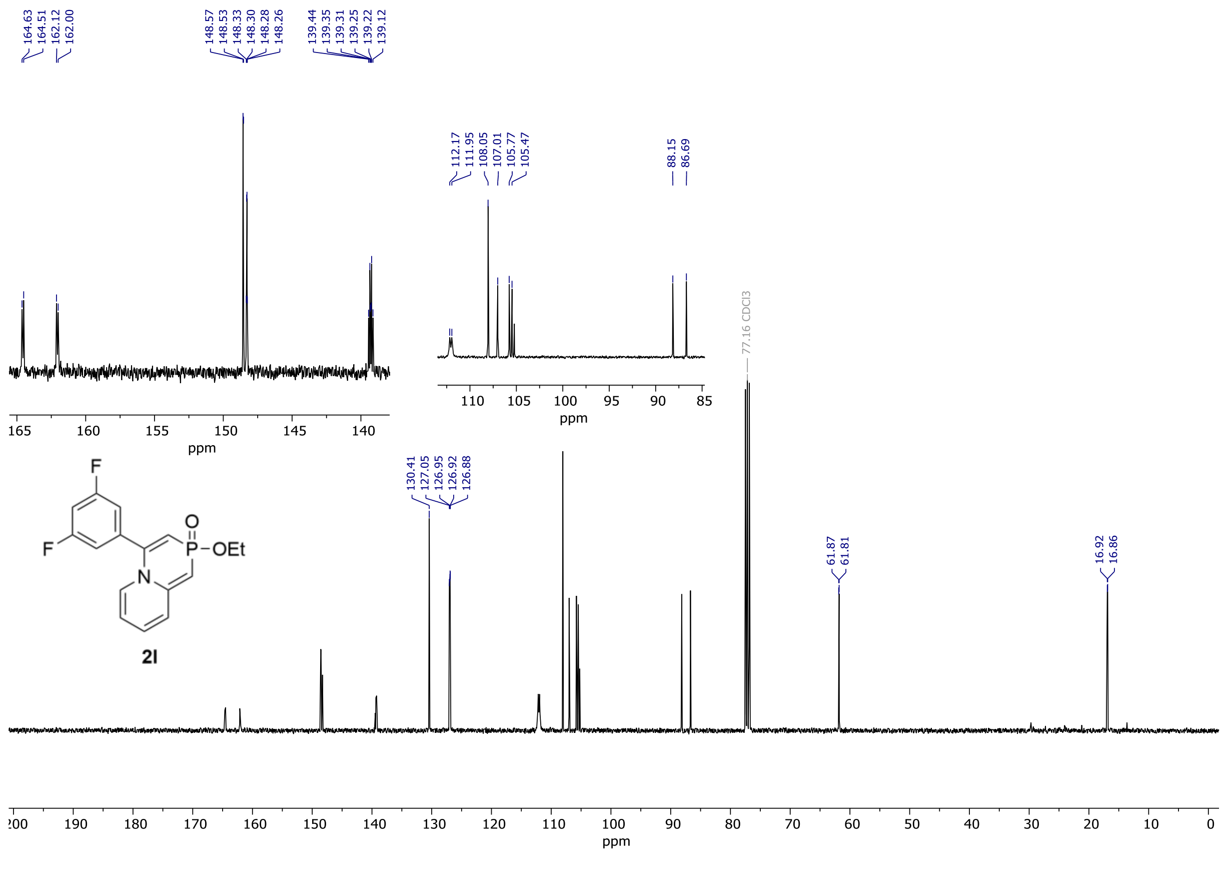

Figure S106 $^{13}C\{^{1}H\}$ NMR spectrum of **2l** (101 MHz, $CDCl_3$).

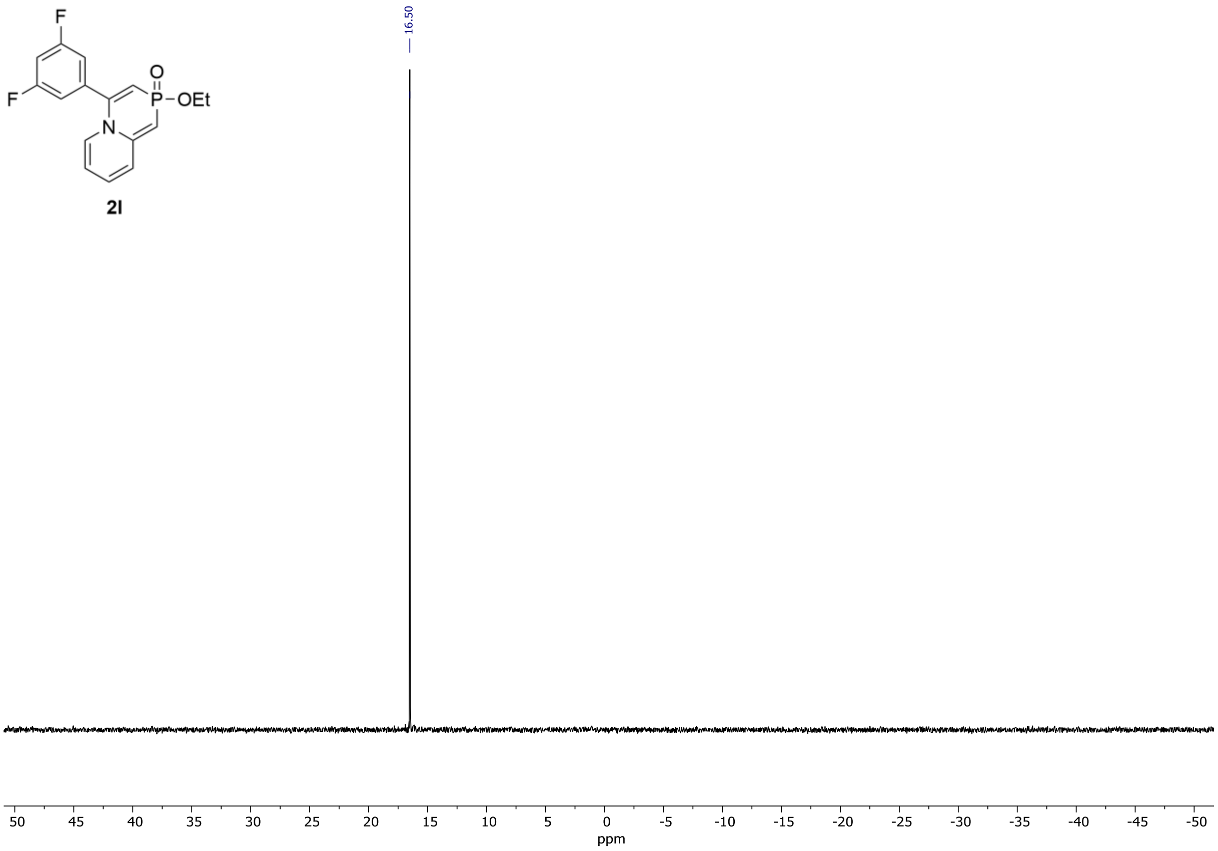

Figure S107 *^{31}P {^{1}H} NMR spectrum of* ***2l*** *(162 MHz, $CDCl_3$).*

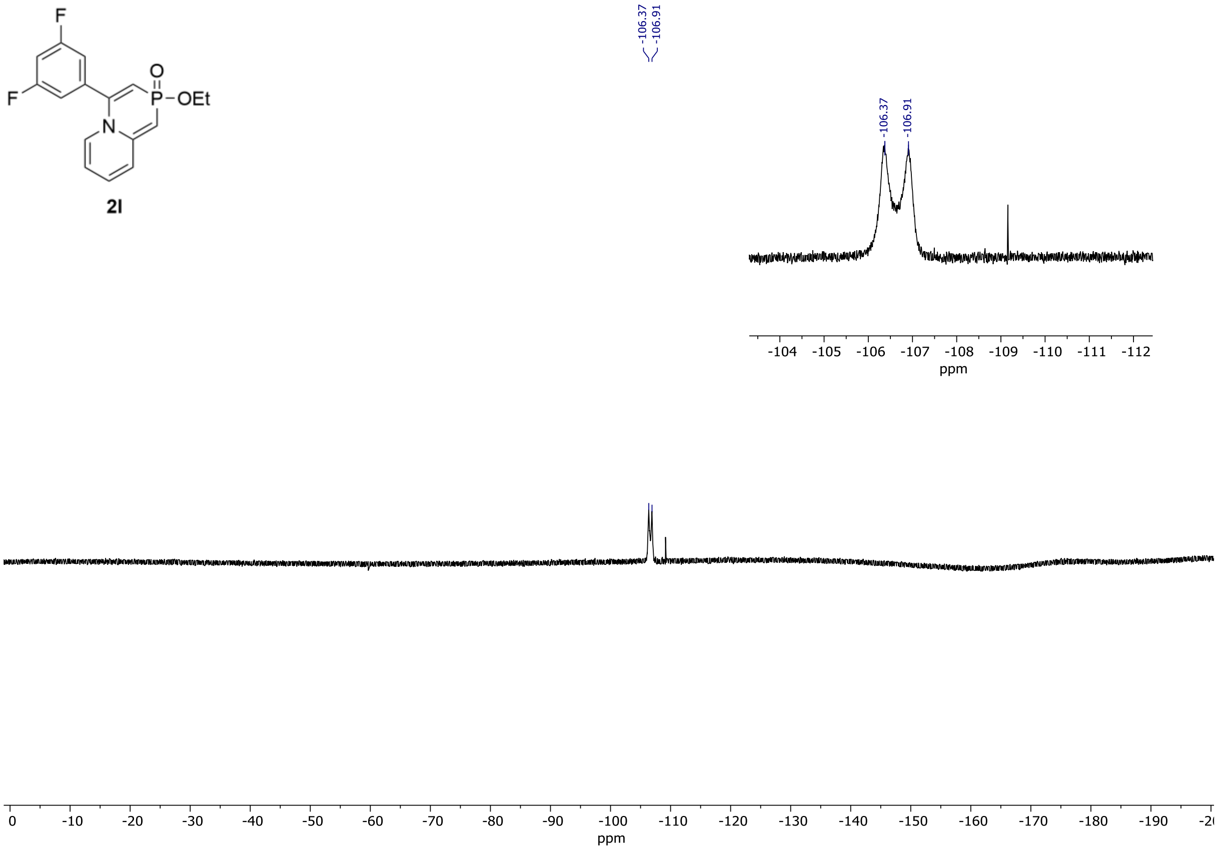

Figure S108 *^{19}F {^{1}H} NMR spectrum of* ***2l*** *(376 MHz, $CDCl_3$).*

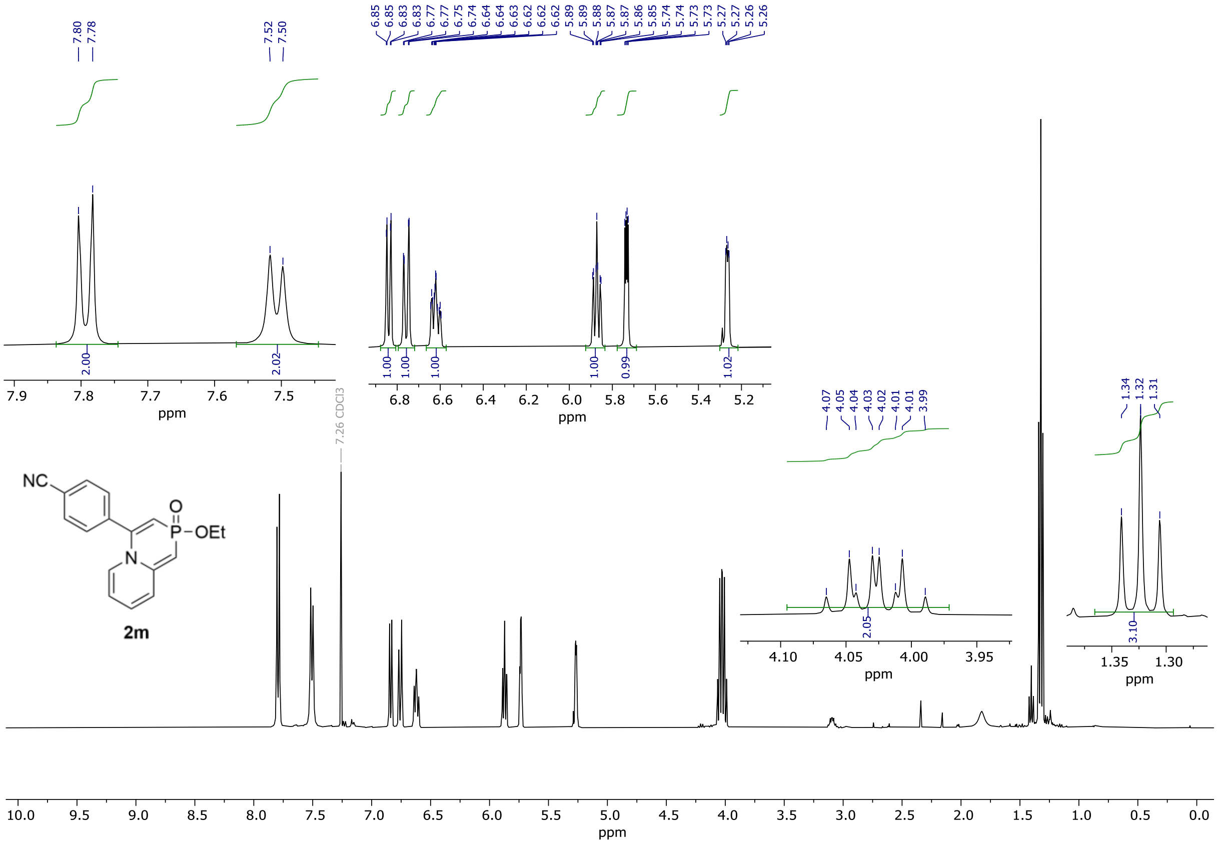

Figure S109 *^{1}H NMR spectrum of **2m** (400 MHz, CDCl$_3$).*

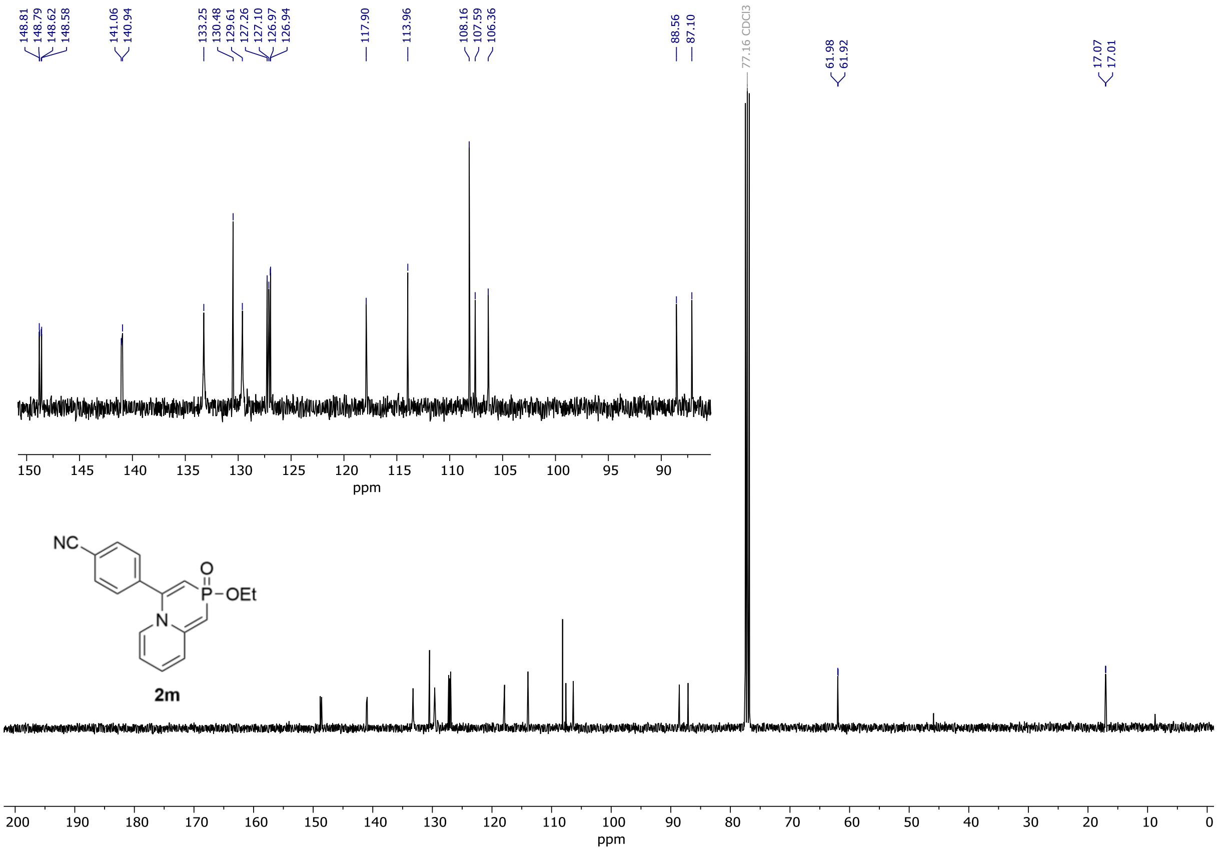

Figure S110 *^{13}C {^{1}H} NMR spectrum of **2m** (101 MHz, CDCl$_3$).*

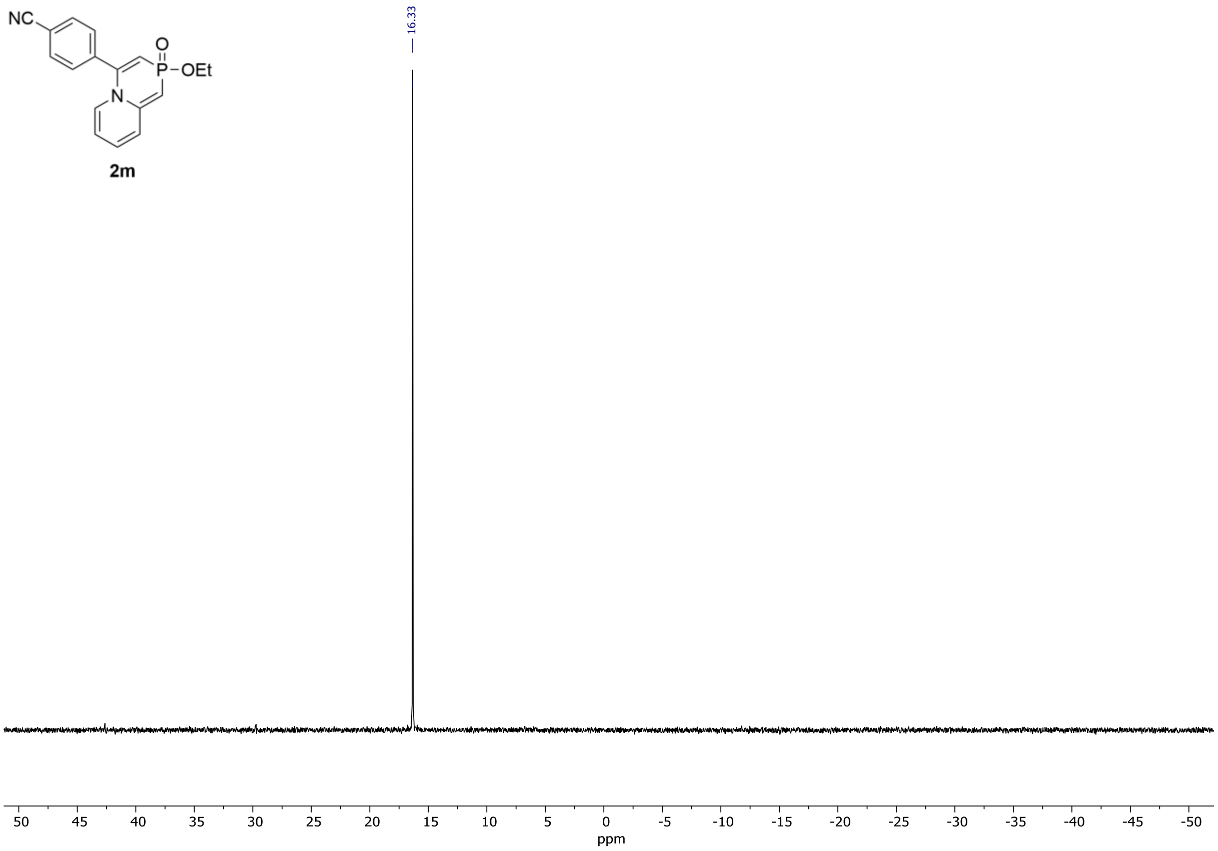

Figure S111 *^{31}P {^{1}H} NMR spectrum of **2m** (162 MHz, CDCl$_3$).*

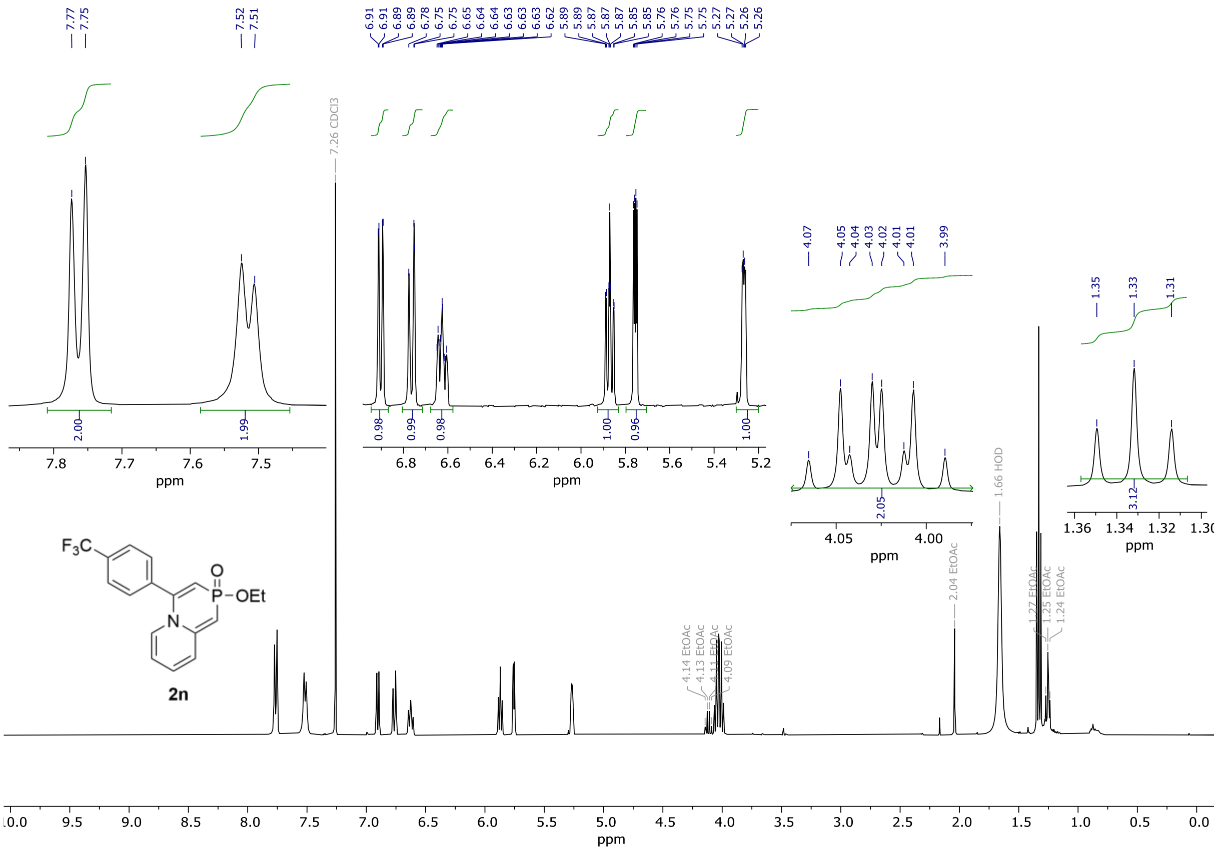

Figure S112 *^{1}H NMR spectrum of **2n** (400 MHz, CDCl$_3$).*

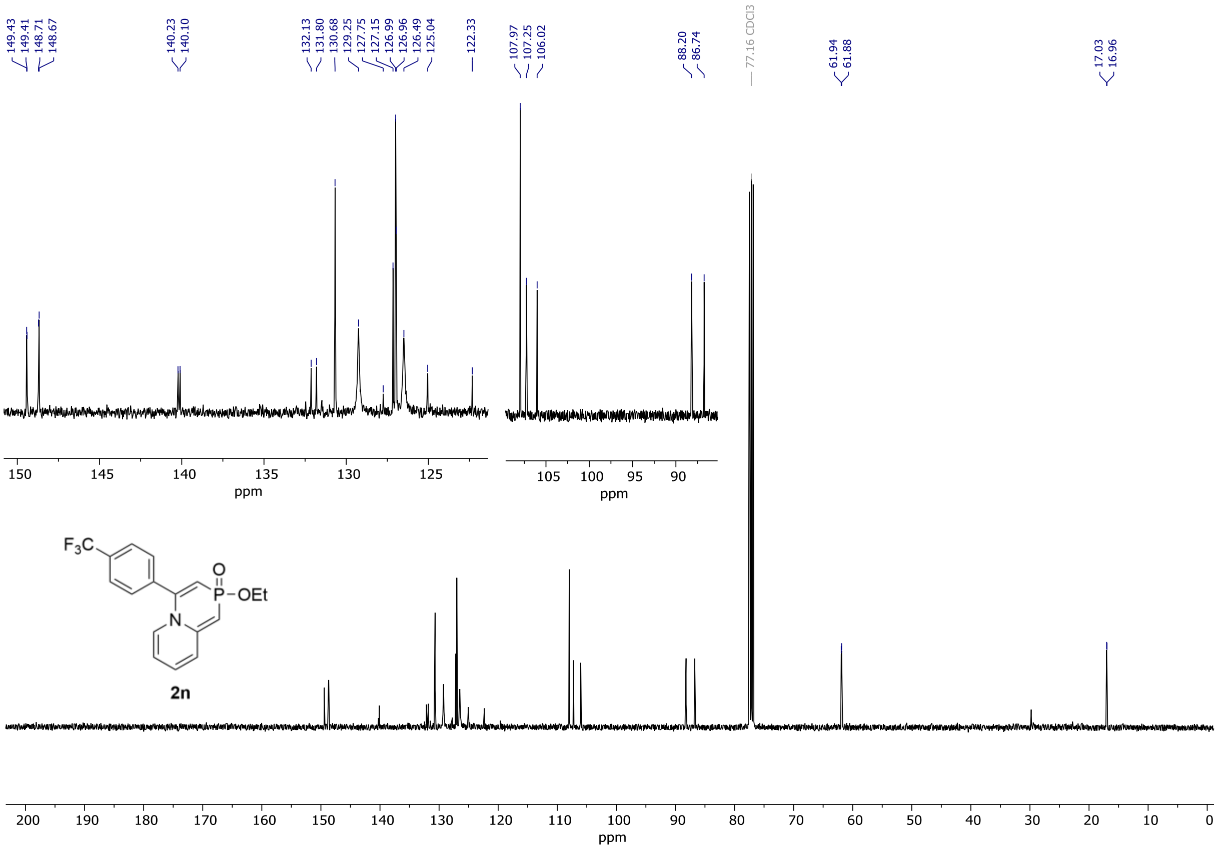

Figure S113 *$^{13}C\ \{^{1}H\}$ NMR spectrum of* ***2n*** *(101 MHz, $CDCl_3$).*

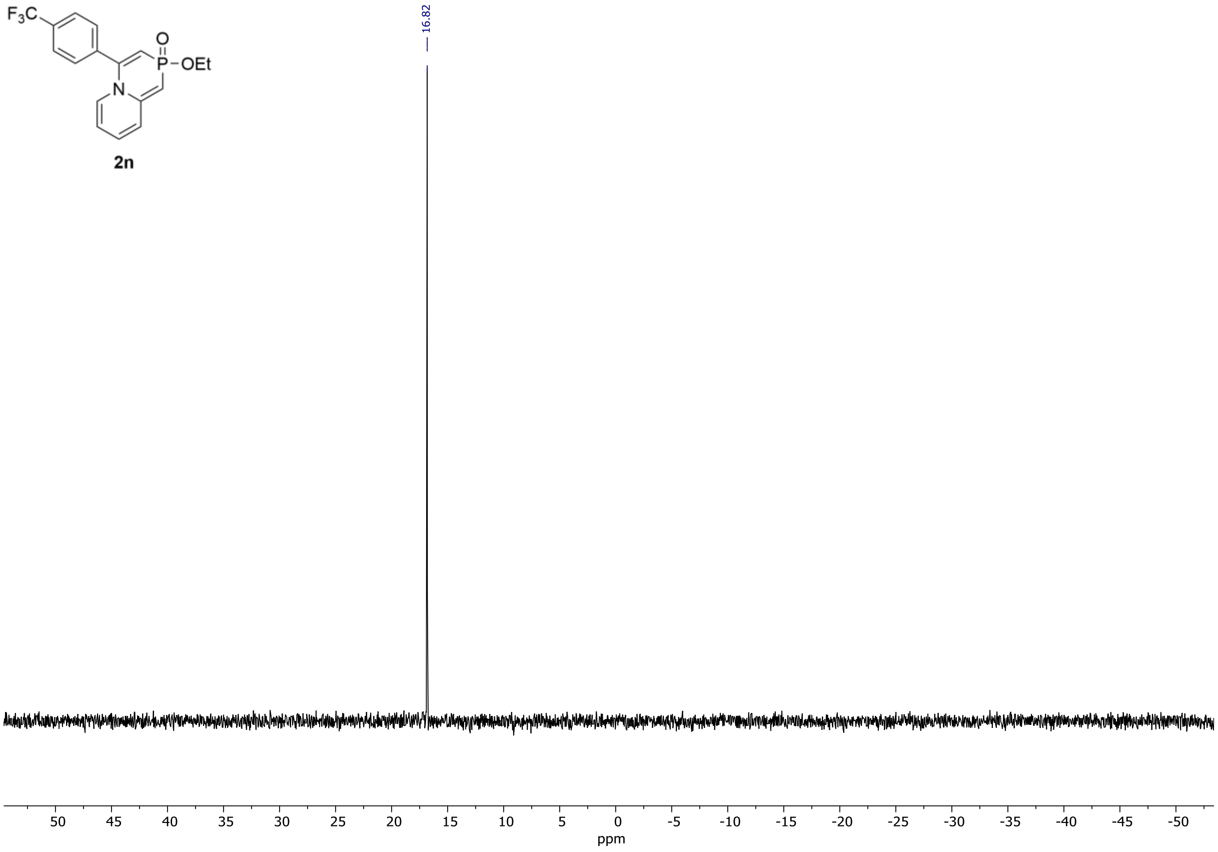

Figure S114 *$^{31}P\ \{^{1}H\}$ NMR spectrum of* ***2n*** *(162 MHz, $CDCl_3$).*

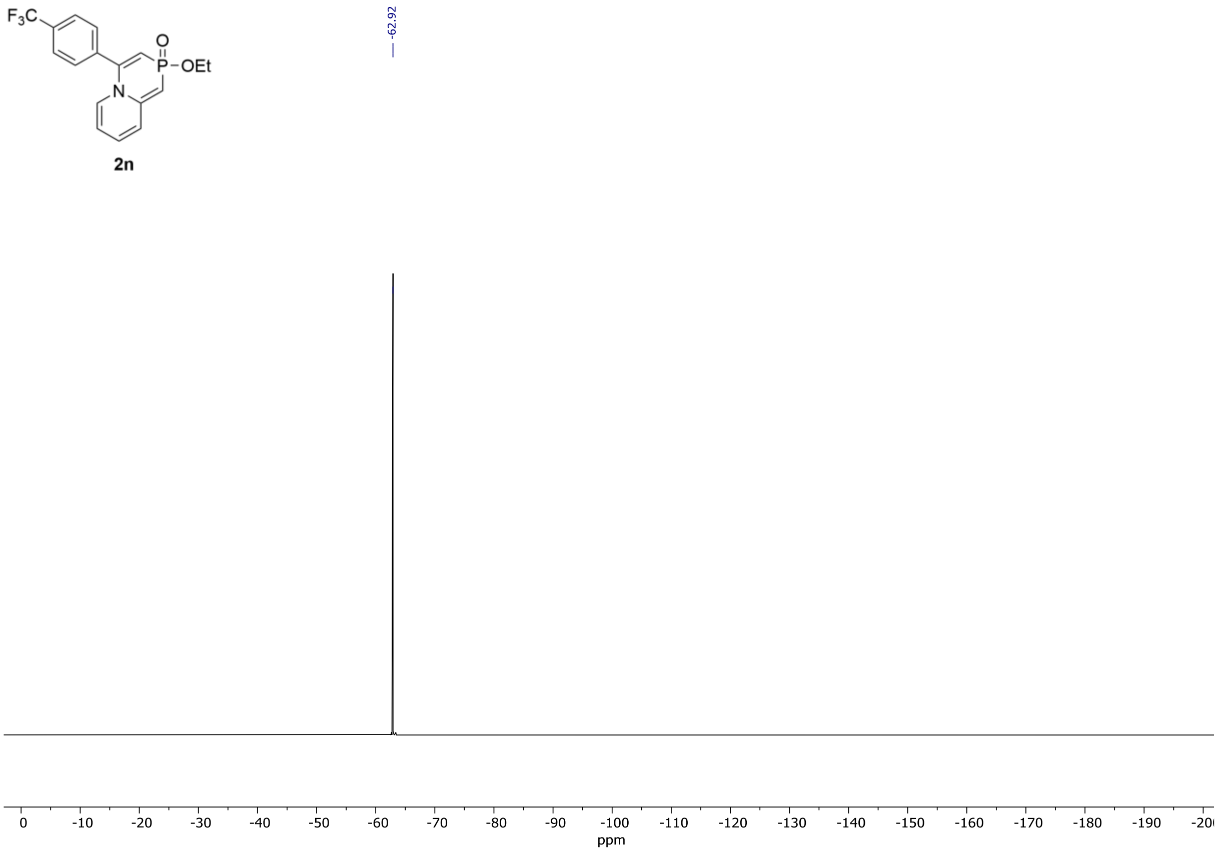

Figure S115 ^{19}F {^{1}H} *NMR spectrum of **2n** (376 MHz, $CDCl_3$).*

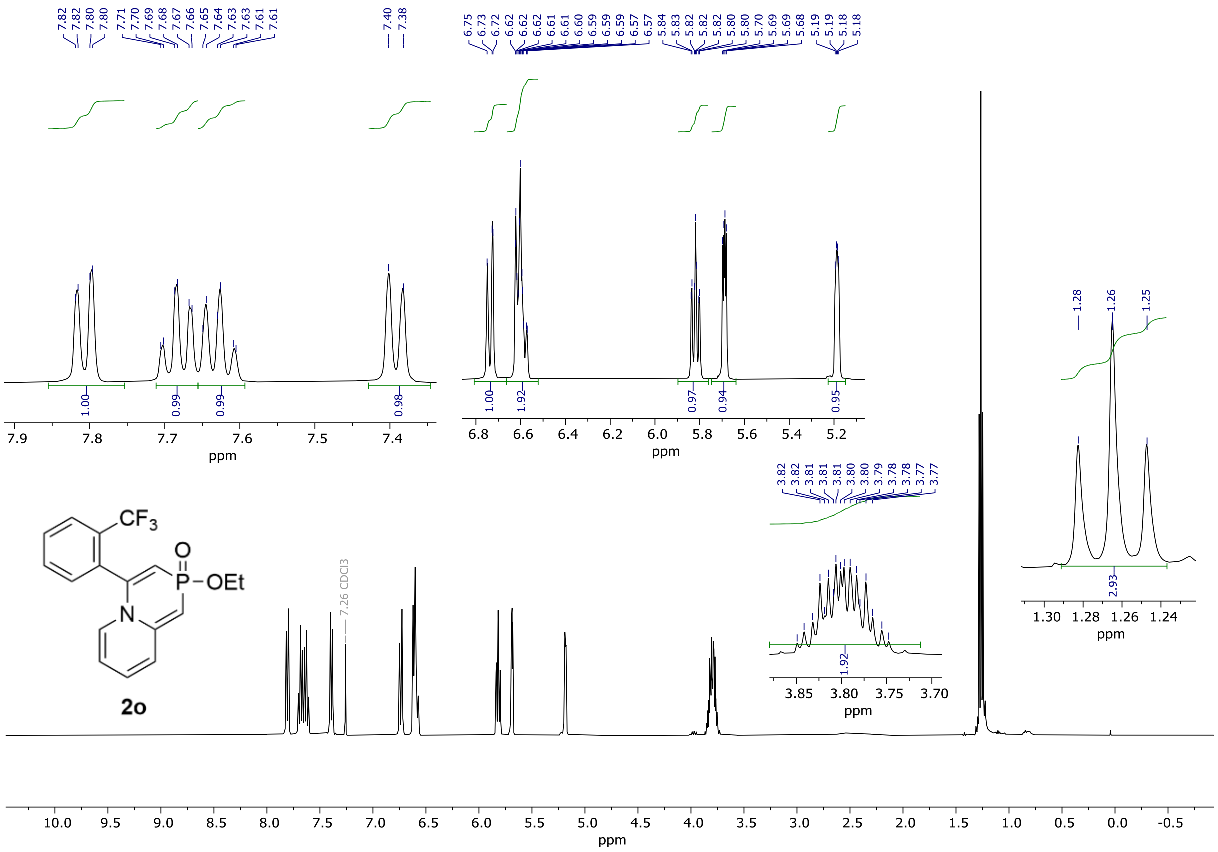

Figure S116 ^{1}H *NMR spectrum of **2o** (400 MHz, $CDCl_3$).*

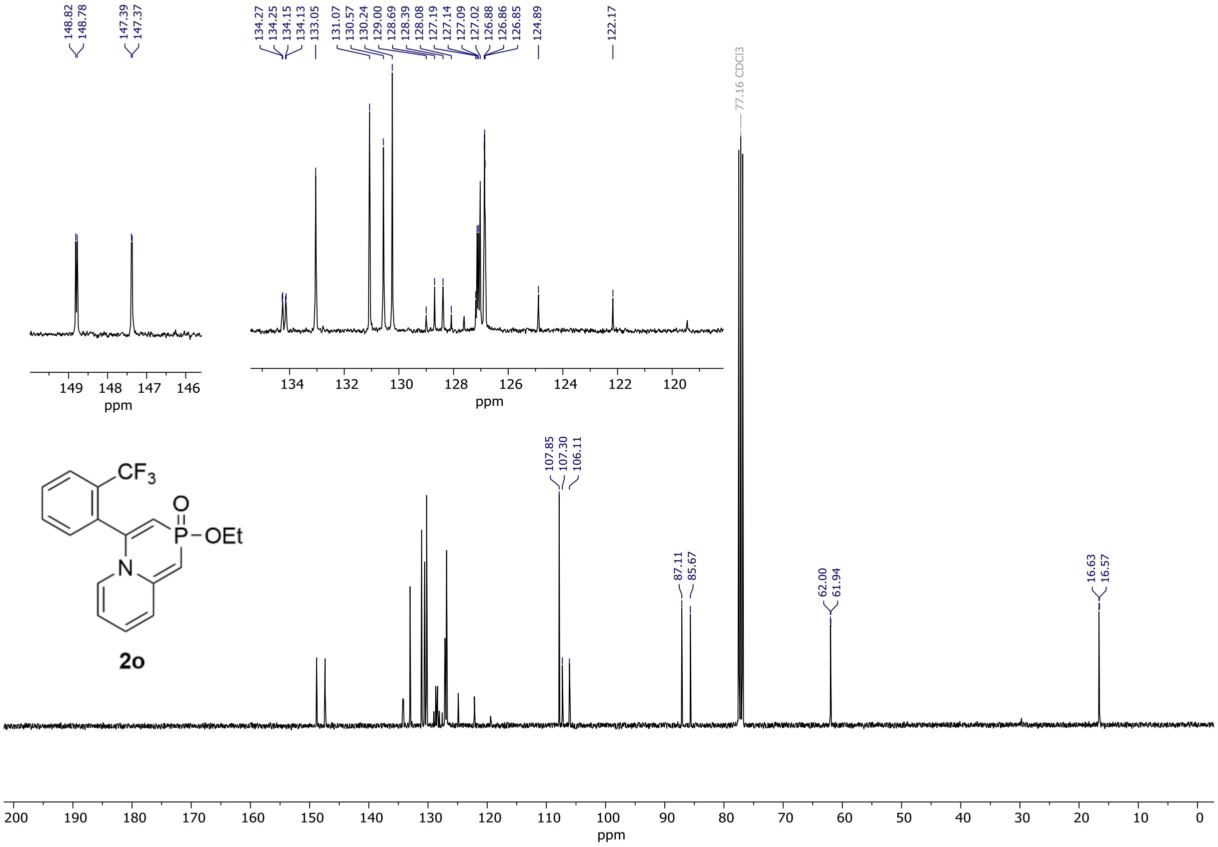

Figure S117 $^{13}C\ \{^{1}H\}$ *NMR spectrum of* ***2o*** *(101 MHz, $CDCl_3$).*

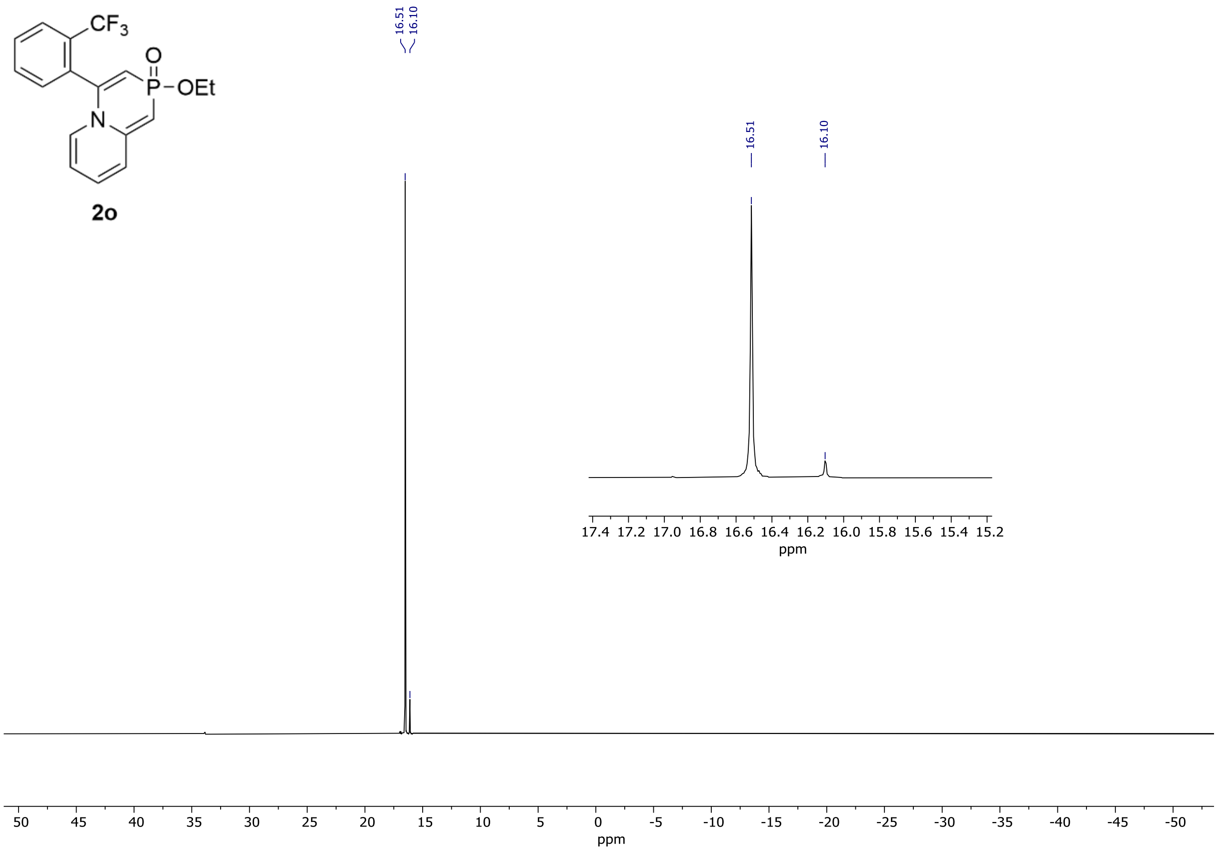

Figure S118 $^{31}P\ \{^{1}H\}$ *NMR spectrum of* ***2o*** *(162 MHz, $CDCl_3$).*

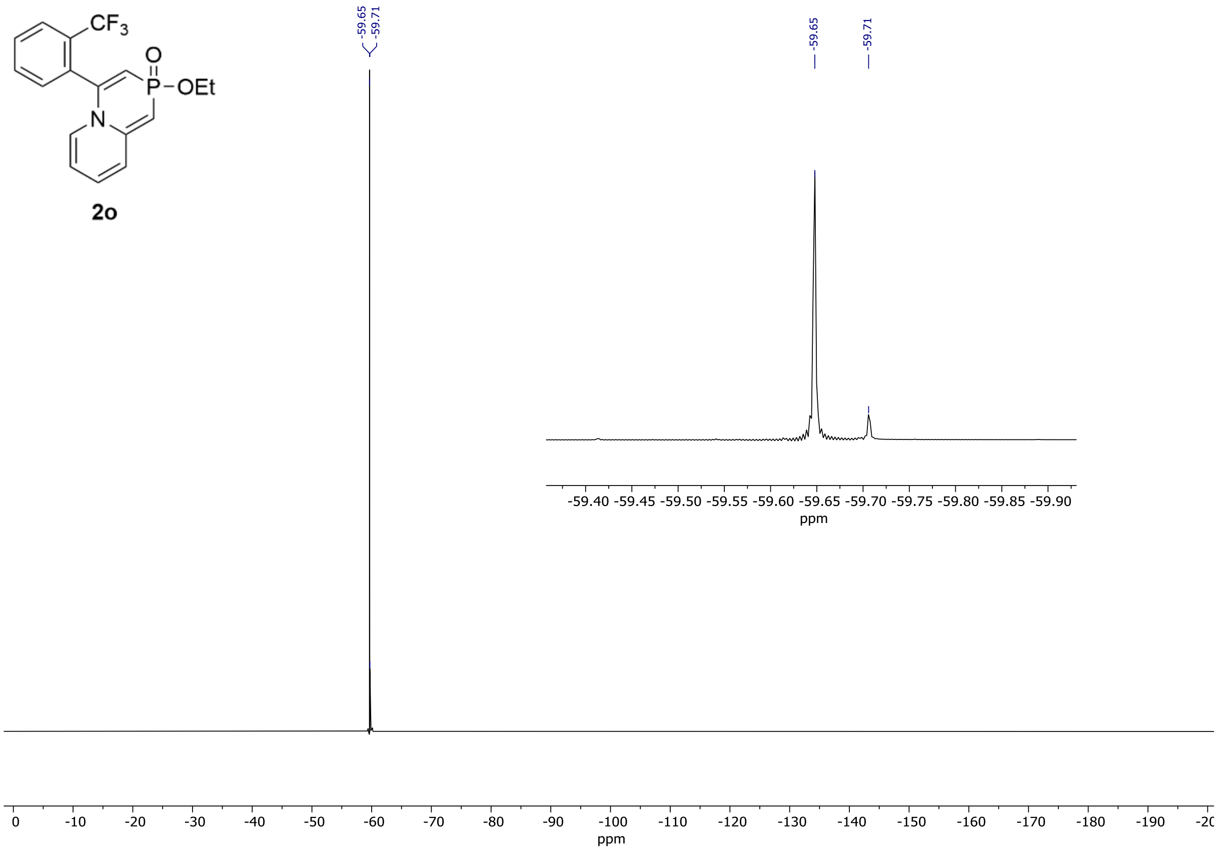

Figure S119 ^{19}F {^{1}H} *NMR spectrum of **2o** (376 MHz, CDCl$_3$).*

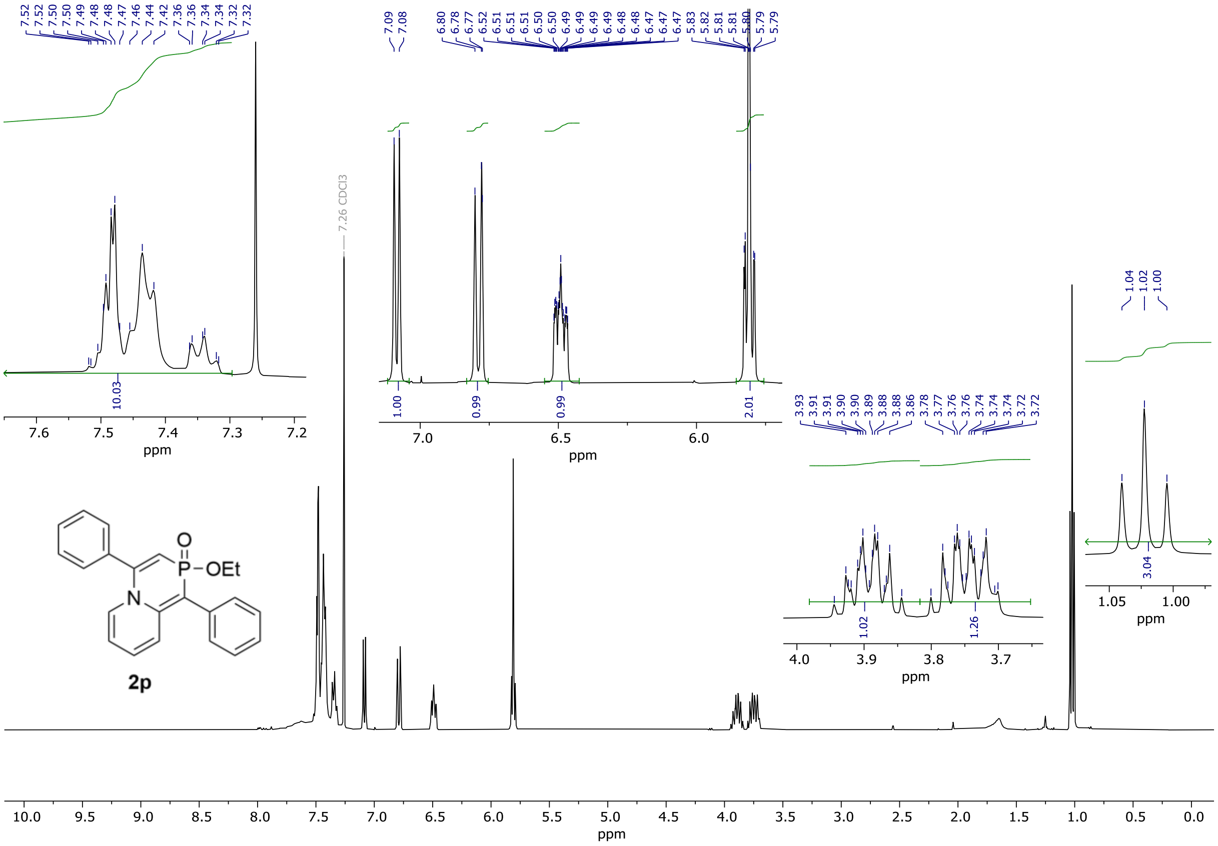

Figure S120 ^{1}H *NMR spectrum of **2p** (400 MHz, CDCl$_3$).*

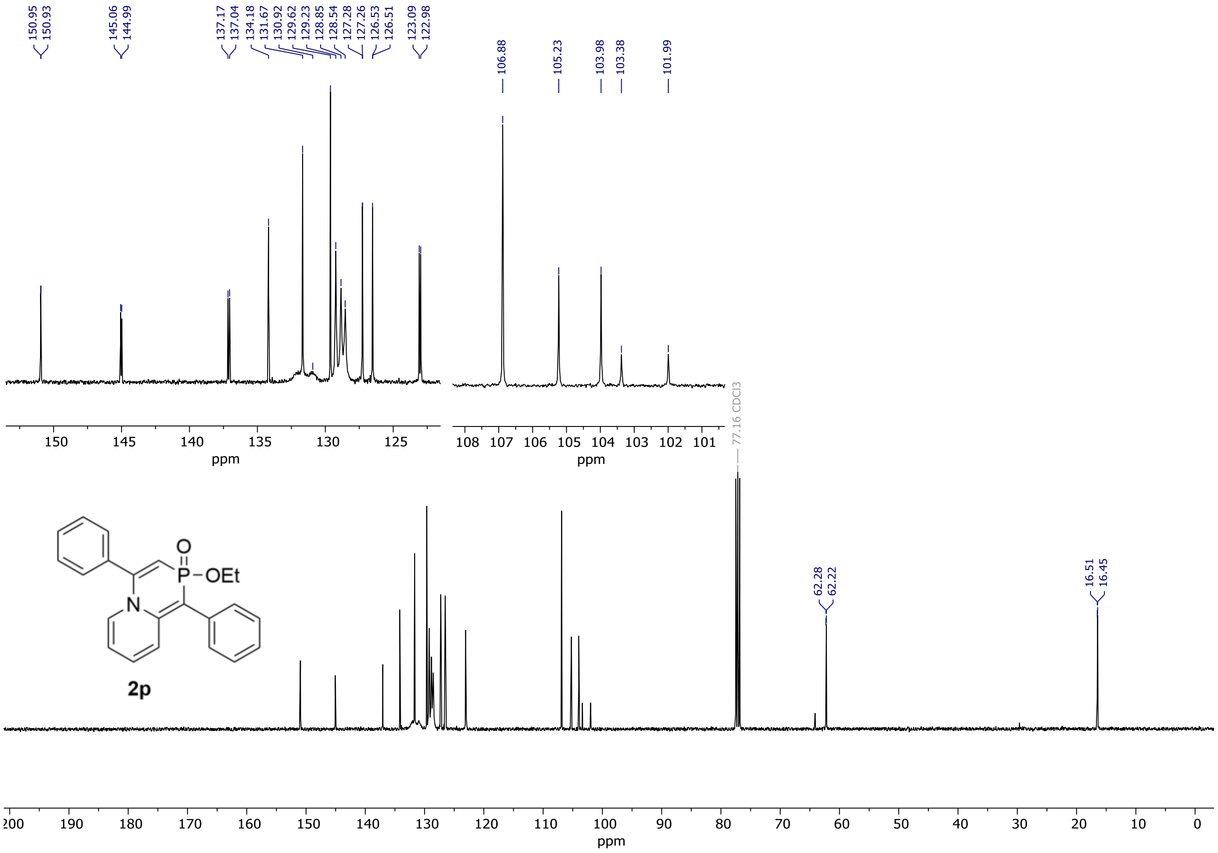

Figure S121 $^{13}C\ \{^1H\}$ *NMR spectrum of* ***2p*** *(101 MHz, $CDCl_3$).*

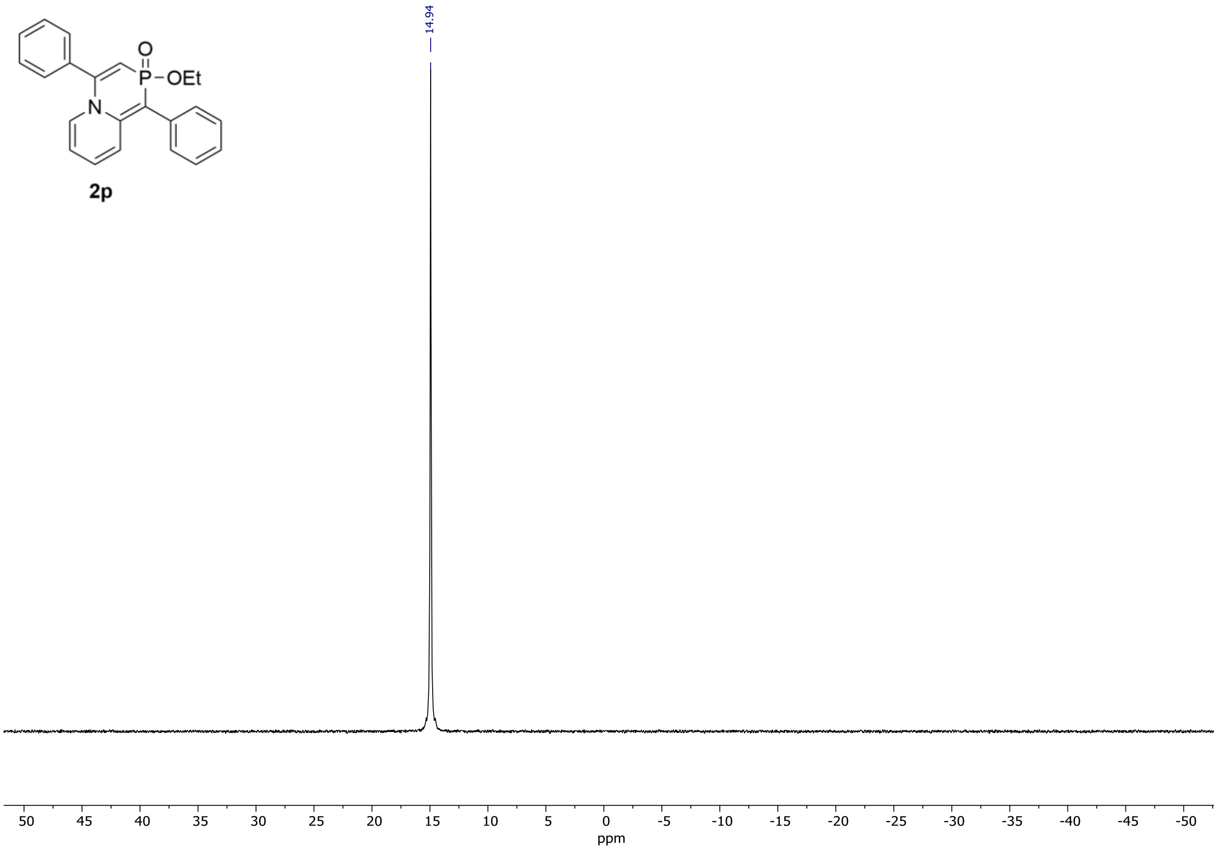

Figure S122 $^{31}P\ \{^1H\}$ *NMR spectrum of* ***2p*** *(162 MHz, $CDCl_3$).*

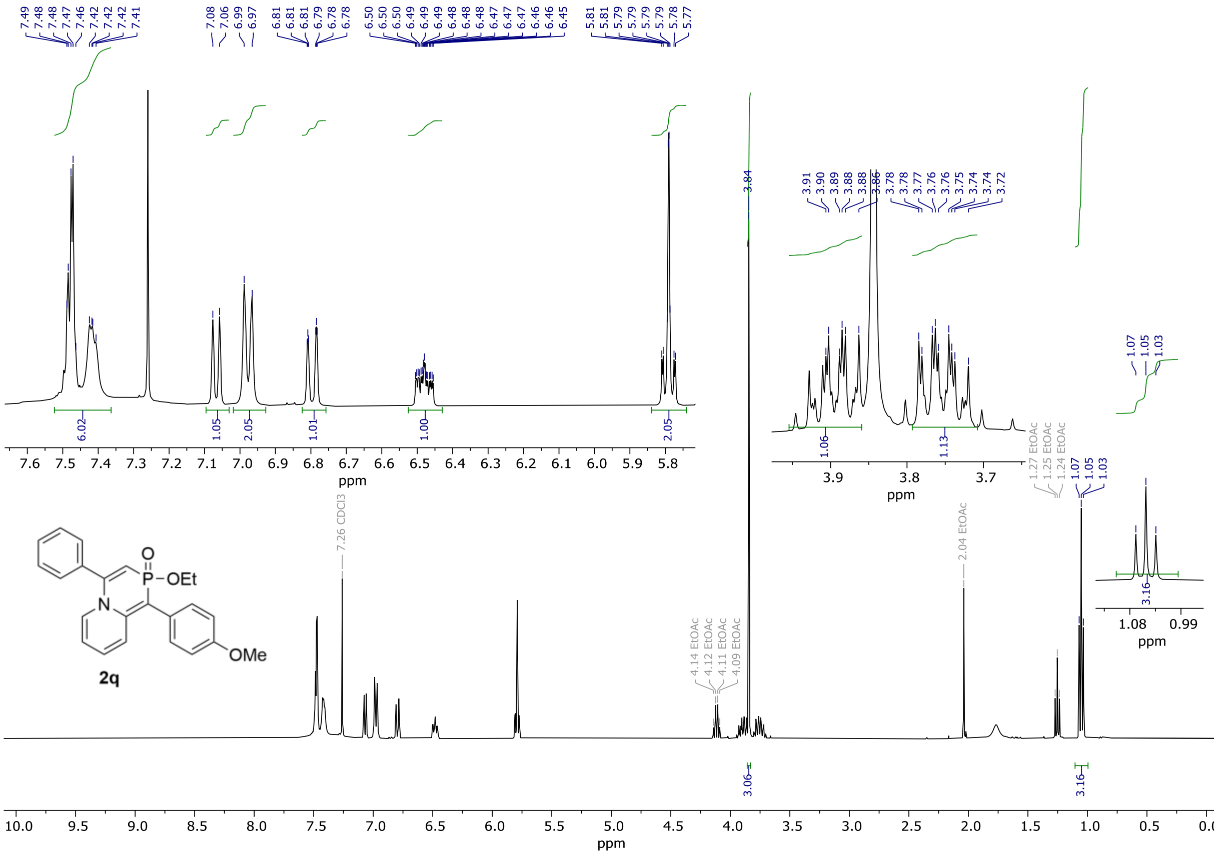

Figure S123 *^{1}H NMR spectrum of **2q** (400 MHz, $CDCl_3$).*

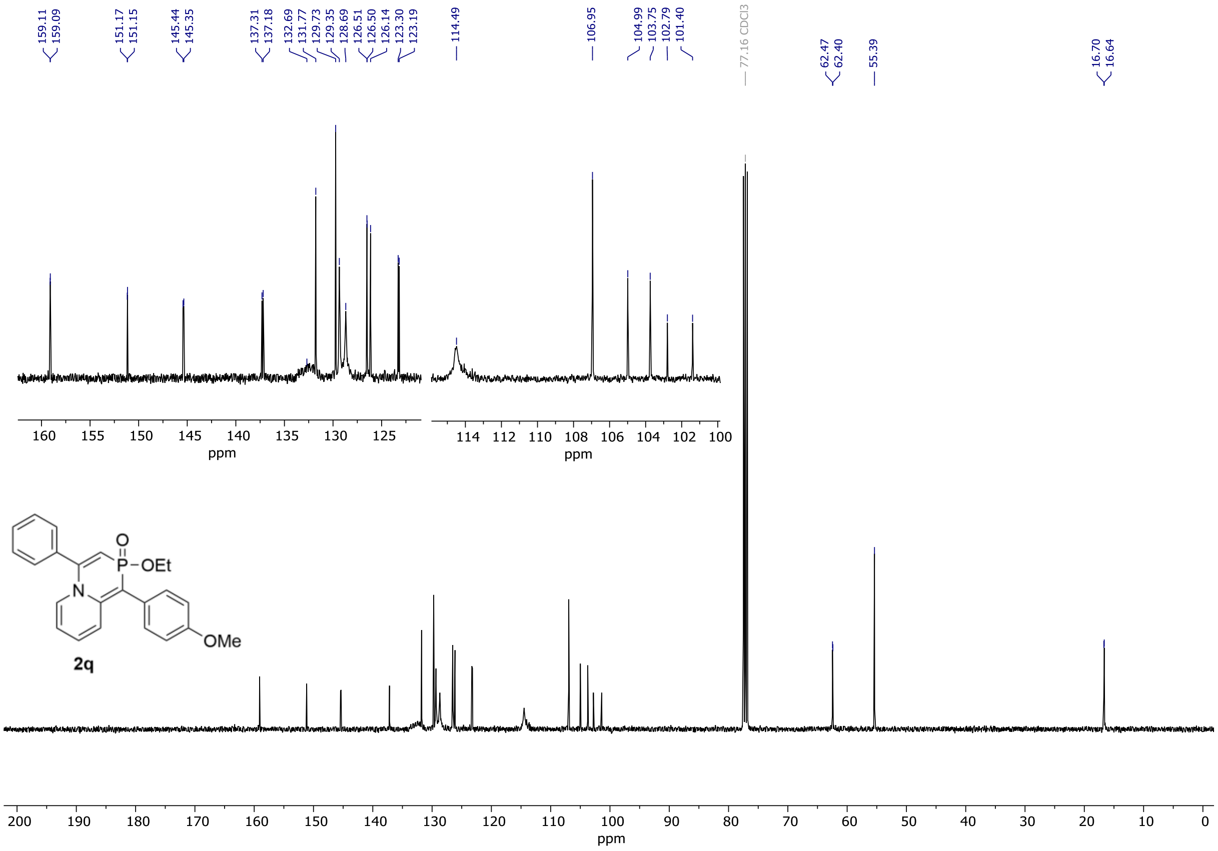

Figure S124 *^{13}C {^{1}H} NMR spectrum of **2q** (101 MHz, $CDCl_3$).*

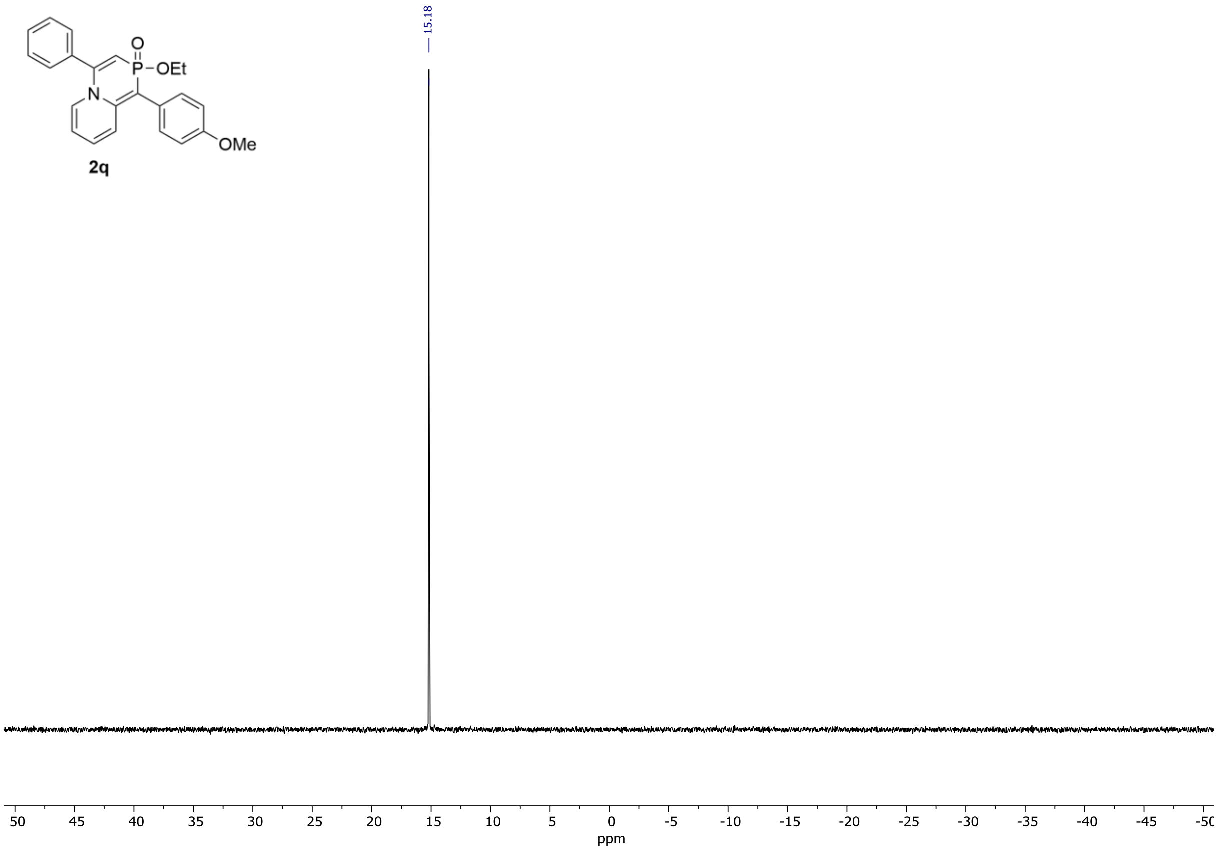

Figure S125 ^{31}P {^1H} *NMR spectrum of **2q** (162 MHz, $CDCl_3$).*

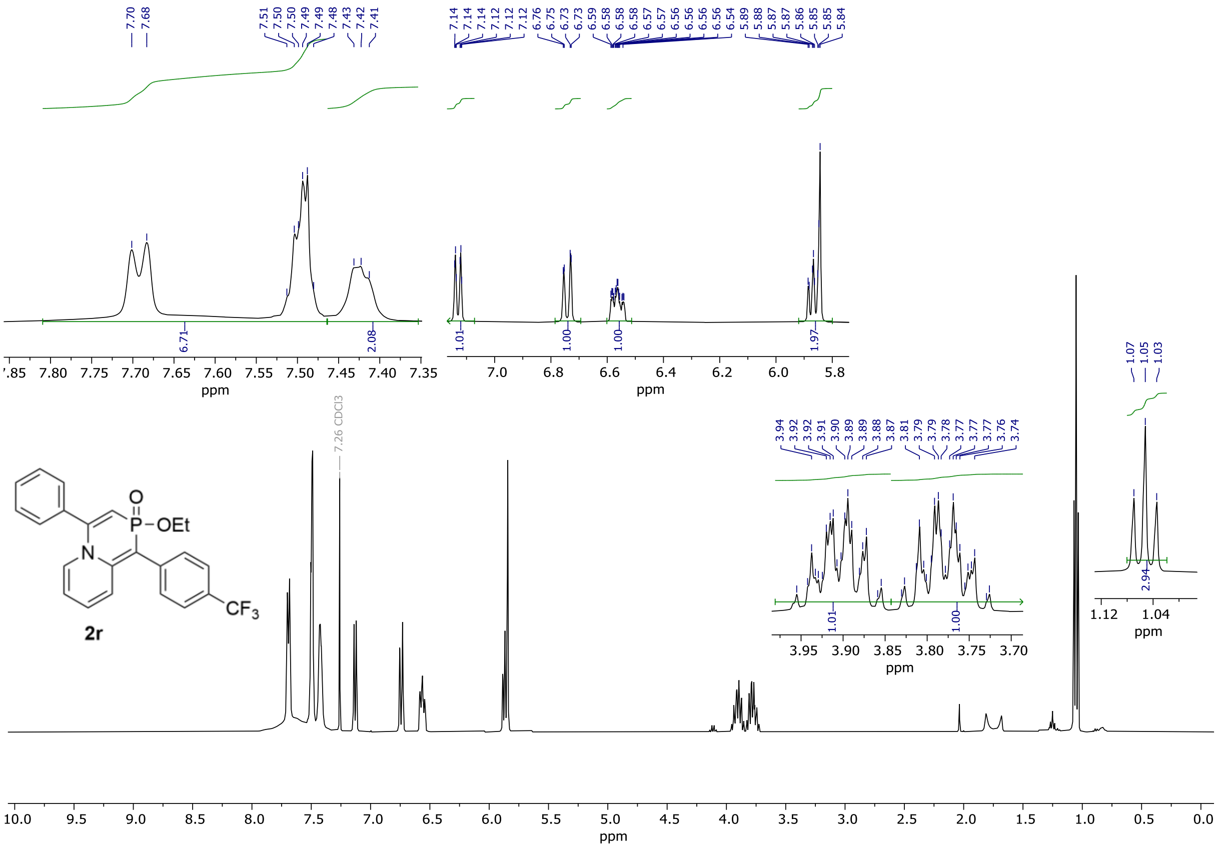

Figure S126 ^1H *NMR spectrum of **2r** (400 MHz, $CDCl_3$).*

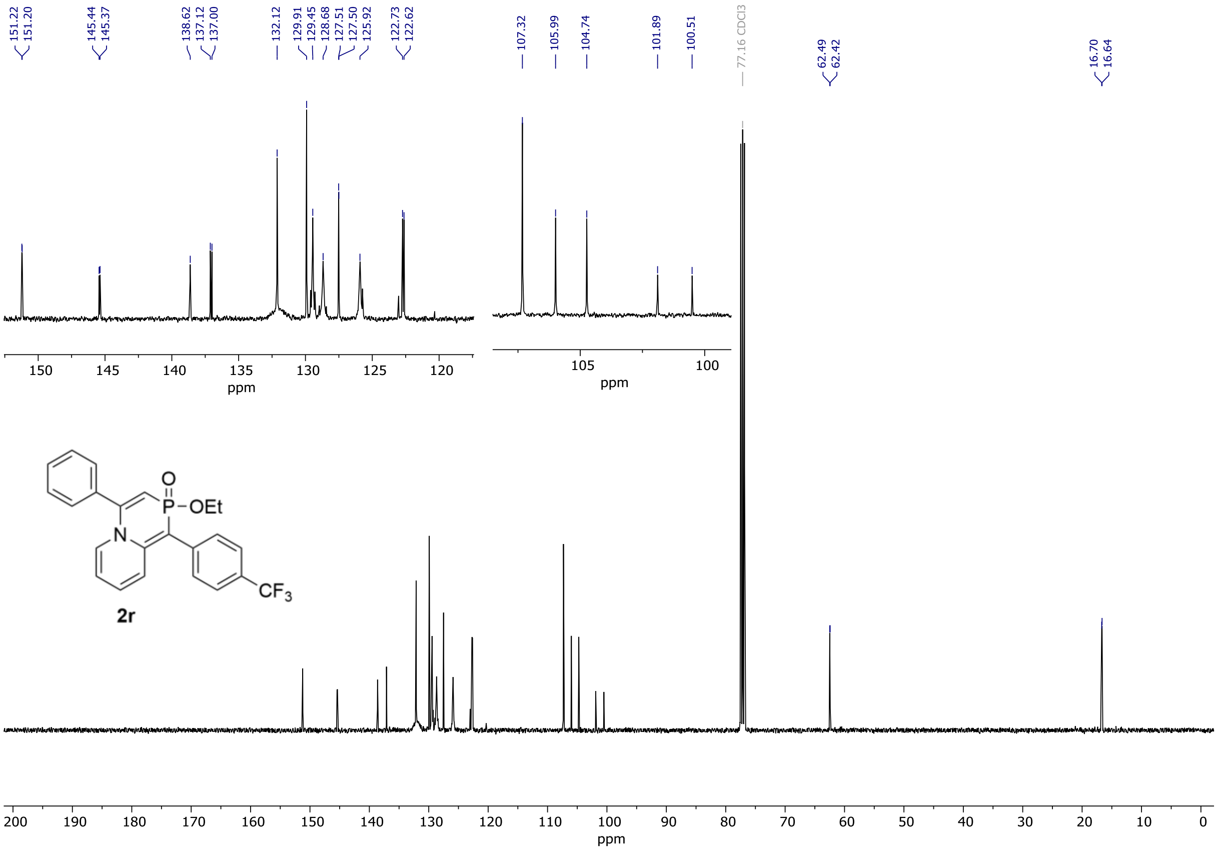

Figure S127 *^{13}C {^{1}H} NMR spectrum of* ***2r*** *(101 MHz, $CDCl_3$).*

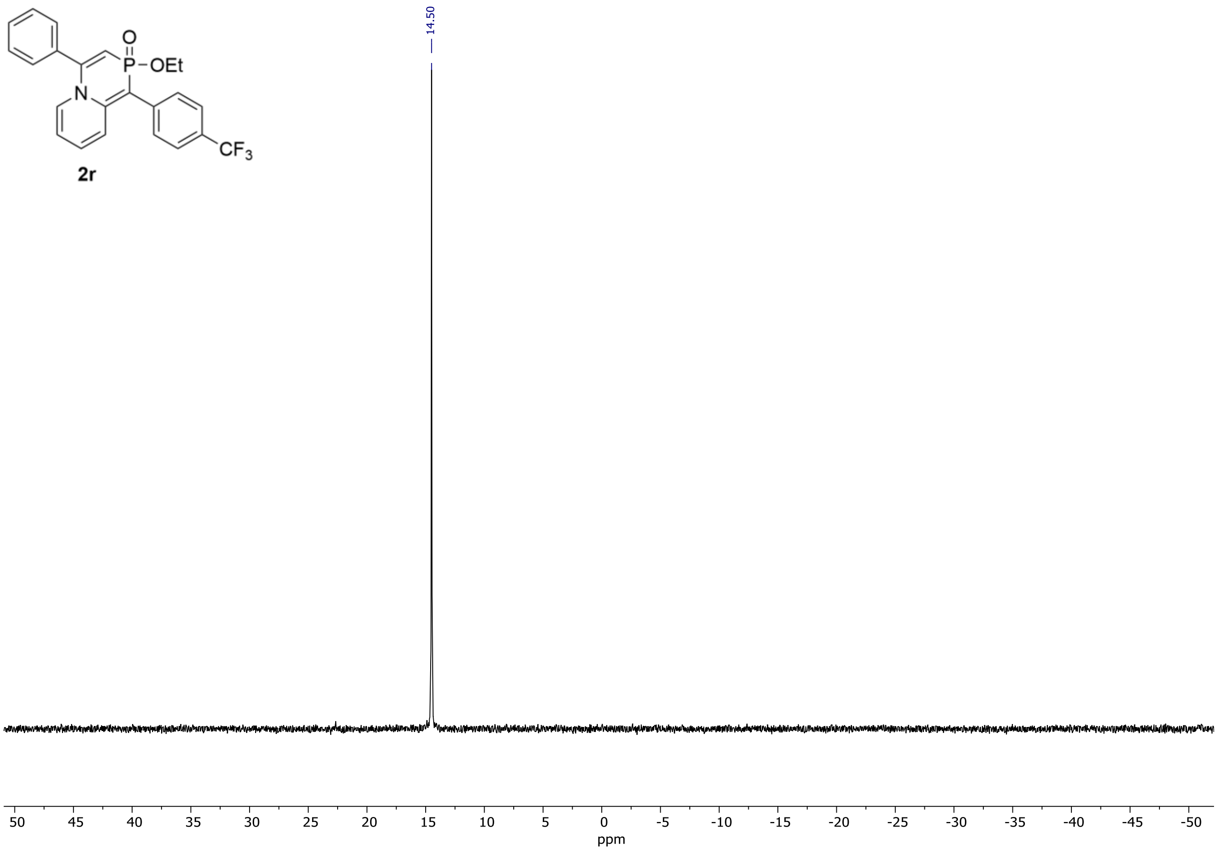

Figure S128 *^{31}P {^{1}H} NMR spectrum of* ***2r*** *(376 MHz, $CDCl_3$).*

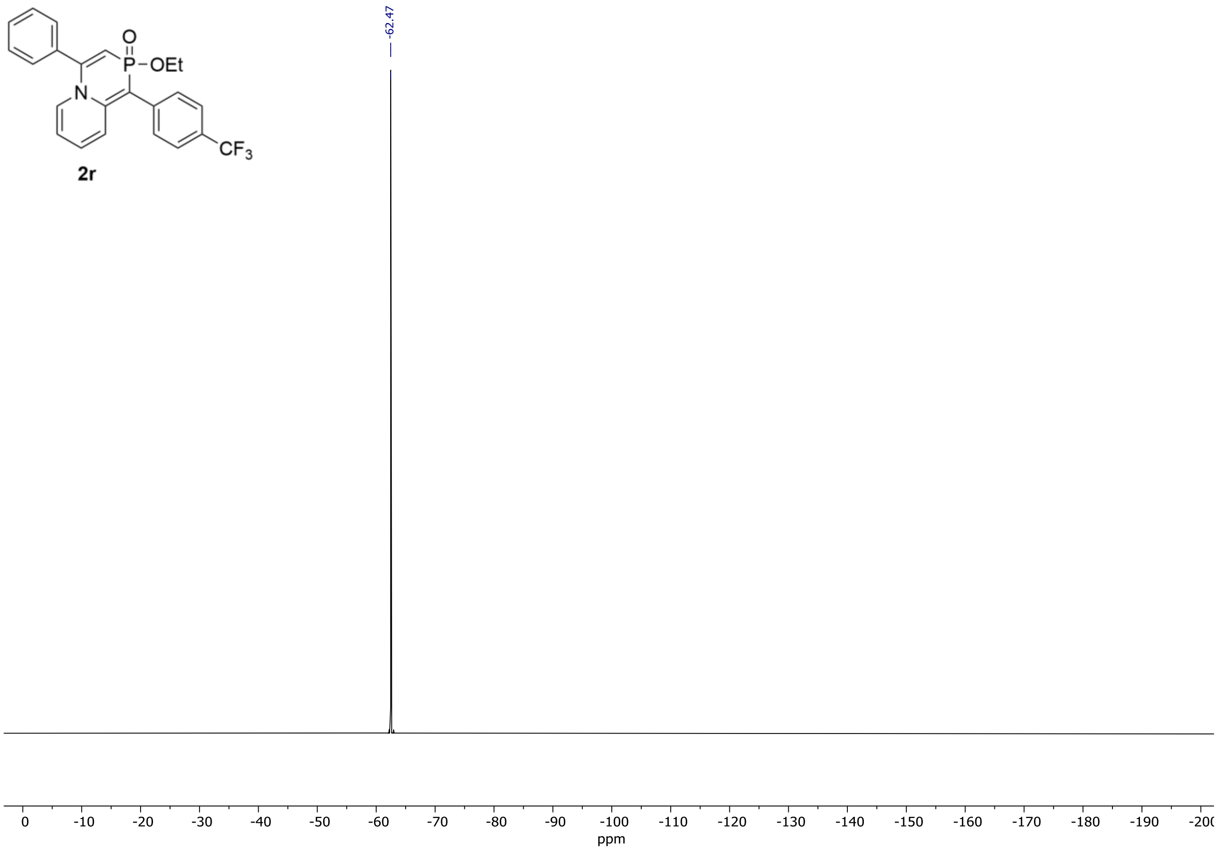

Figure 129 *^{19}F {^{1}H} NMR spectrum of **2r** (376 MHz, CDCl$_3$).*

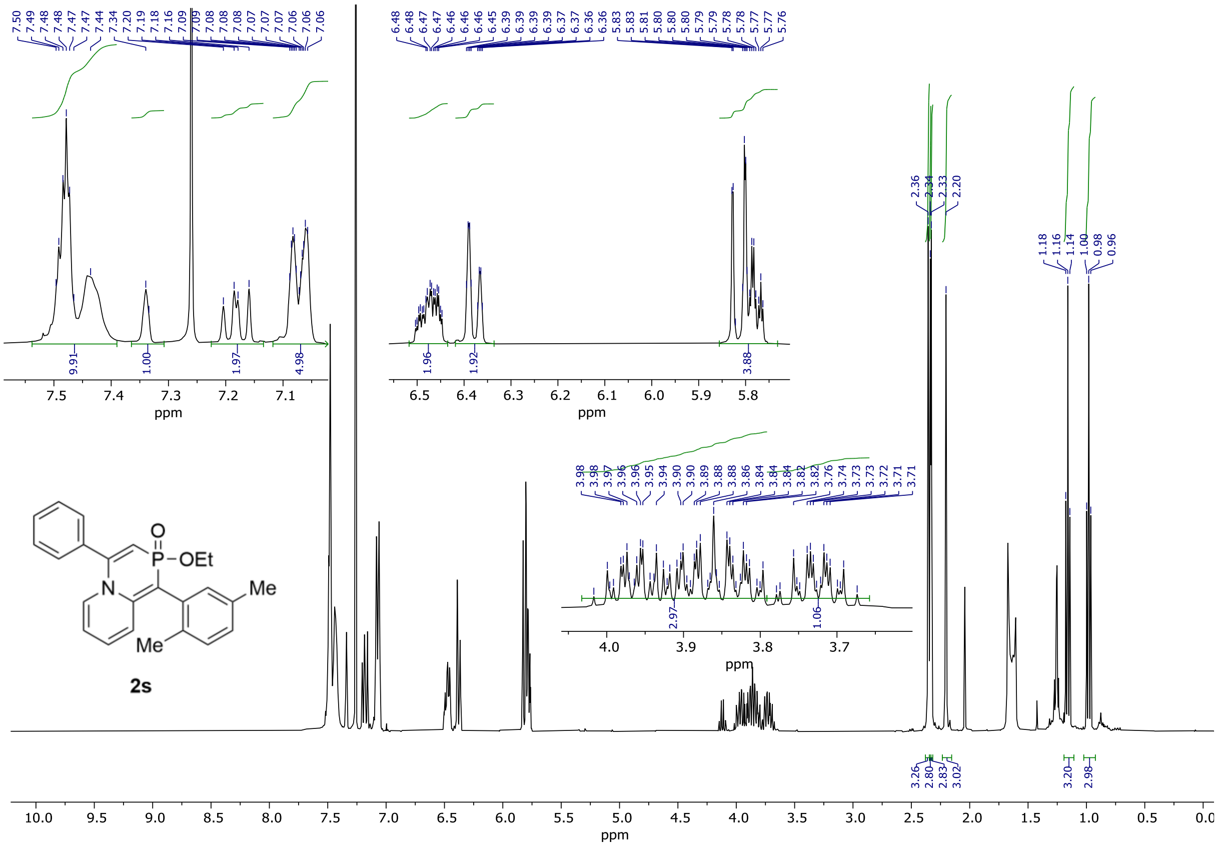

Figure S130 *^{13}C {^{1}H} NMR spectrum of **2s** (101 MHz, CDCl$_3$).*

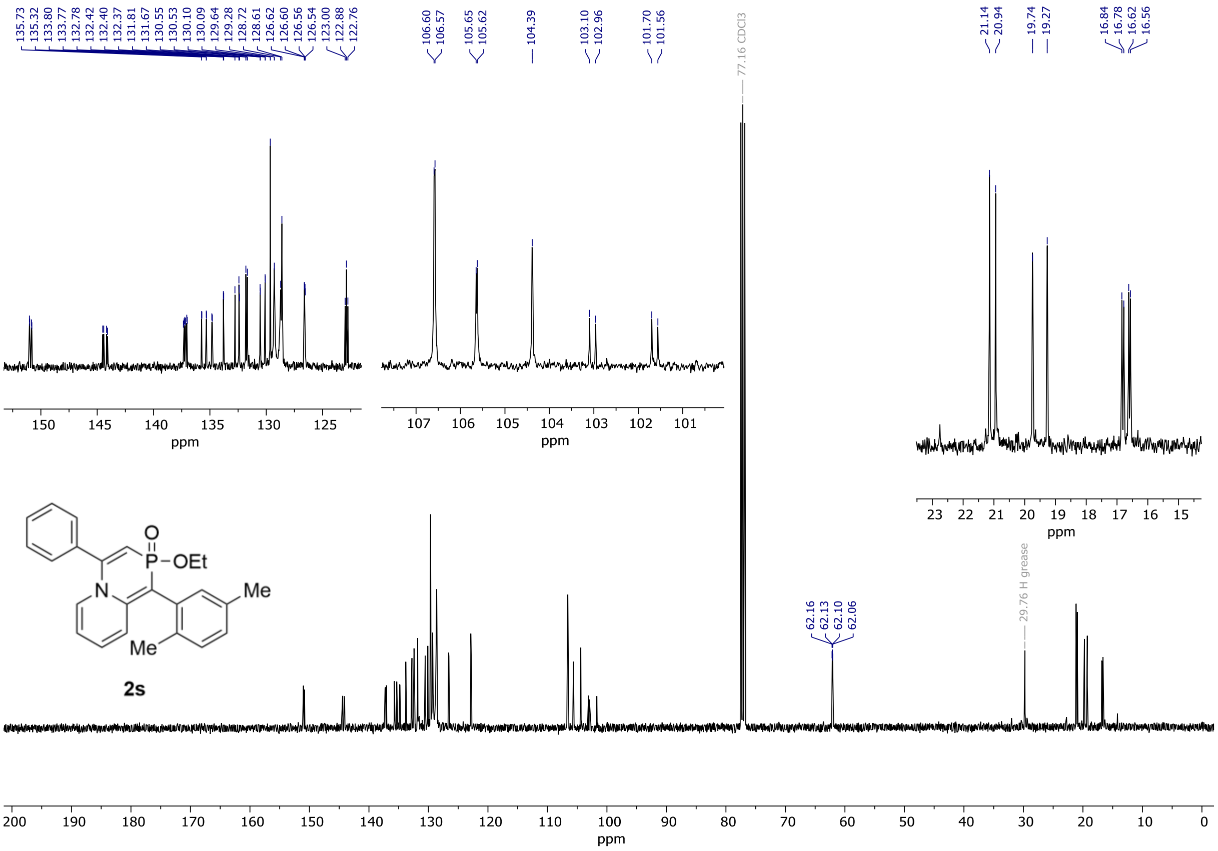

Figure S131 *^{13}C {^{1}H} NMR spectrum of* ***2s*** *(101 MHz, $CDCl_3$).*

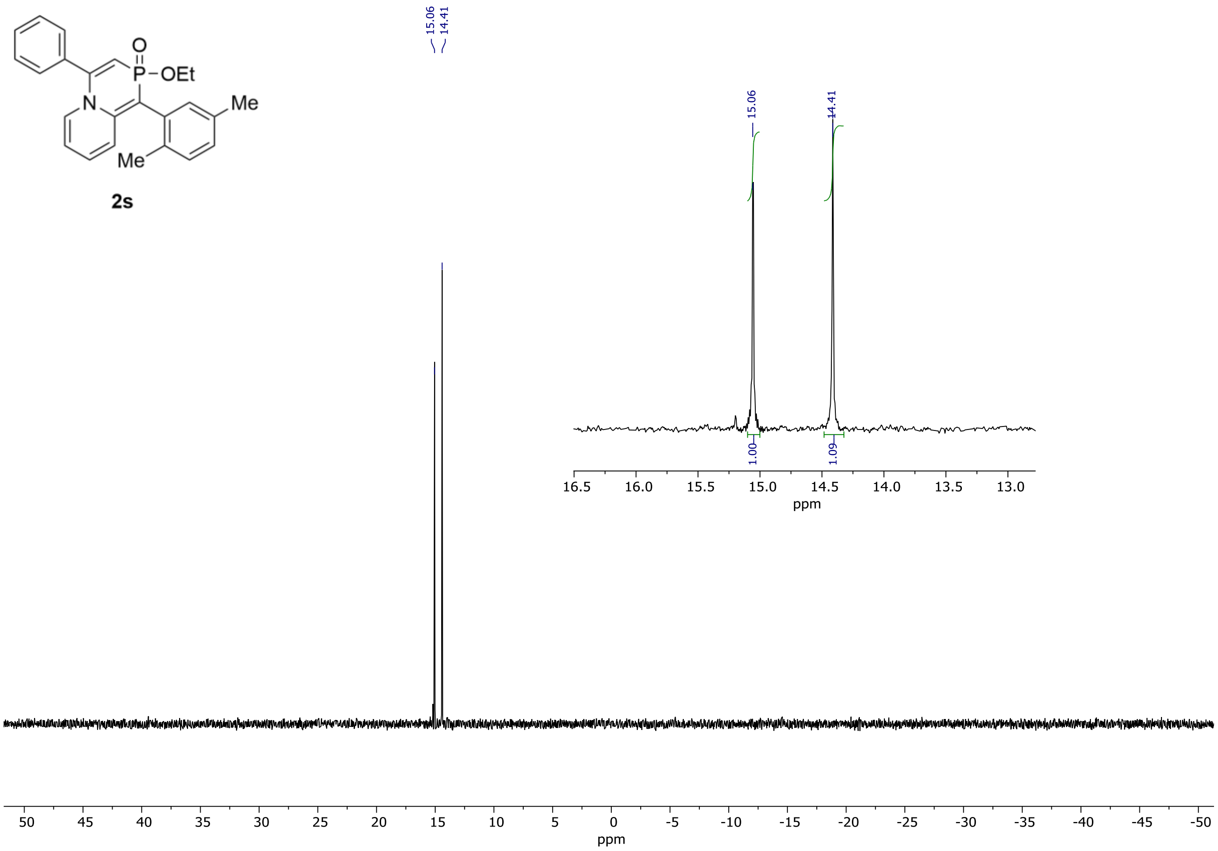

Figure S132 *^{31}P {^{1}H} NMR spectrum of* ***2s*** *(162 MHz, $CDCl_3$).*

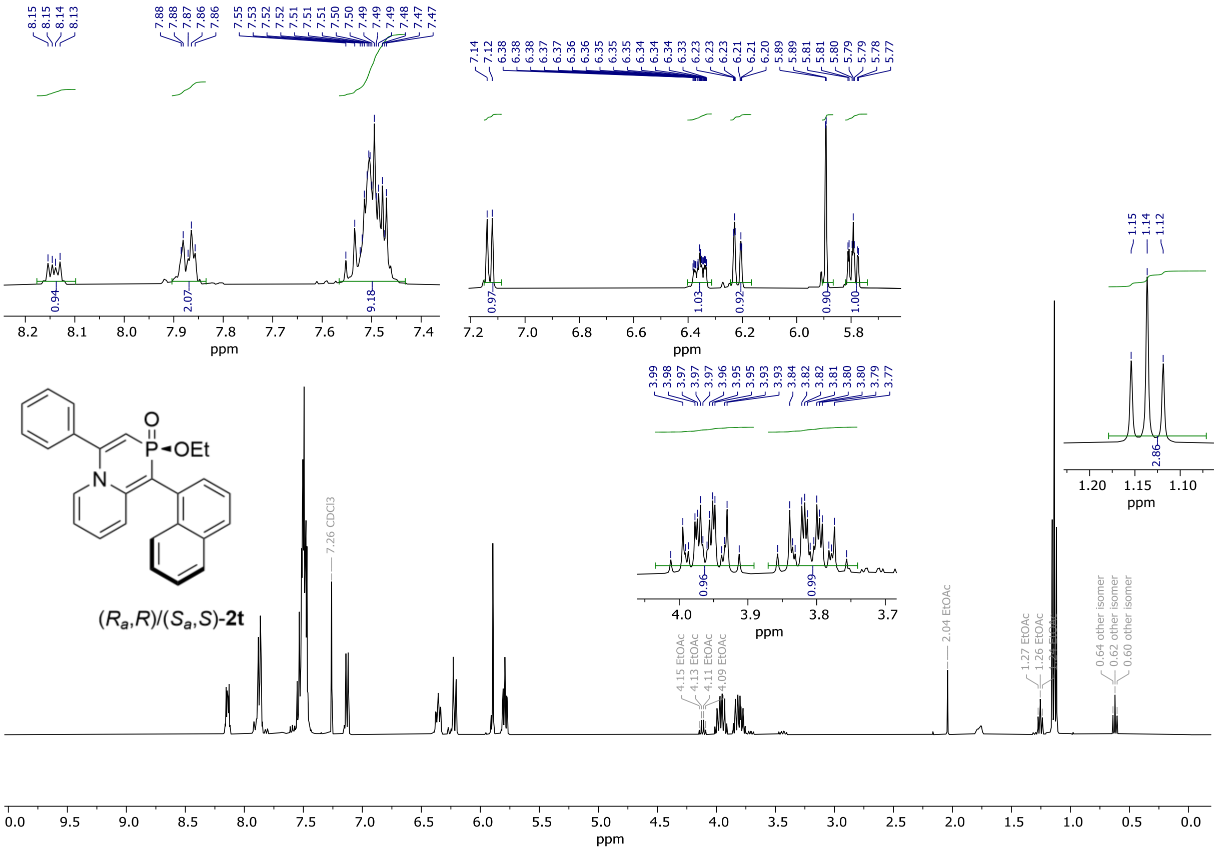

Figure S133 *^{1}H NMR spectrum of (Rₐ,R)/(Sₐ,S)-**2t** (400 MHz, CDCl$_3$).*

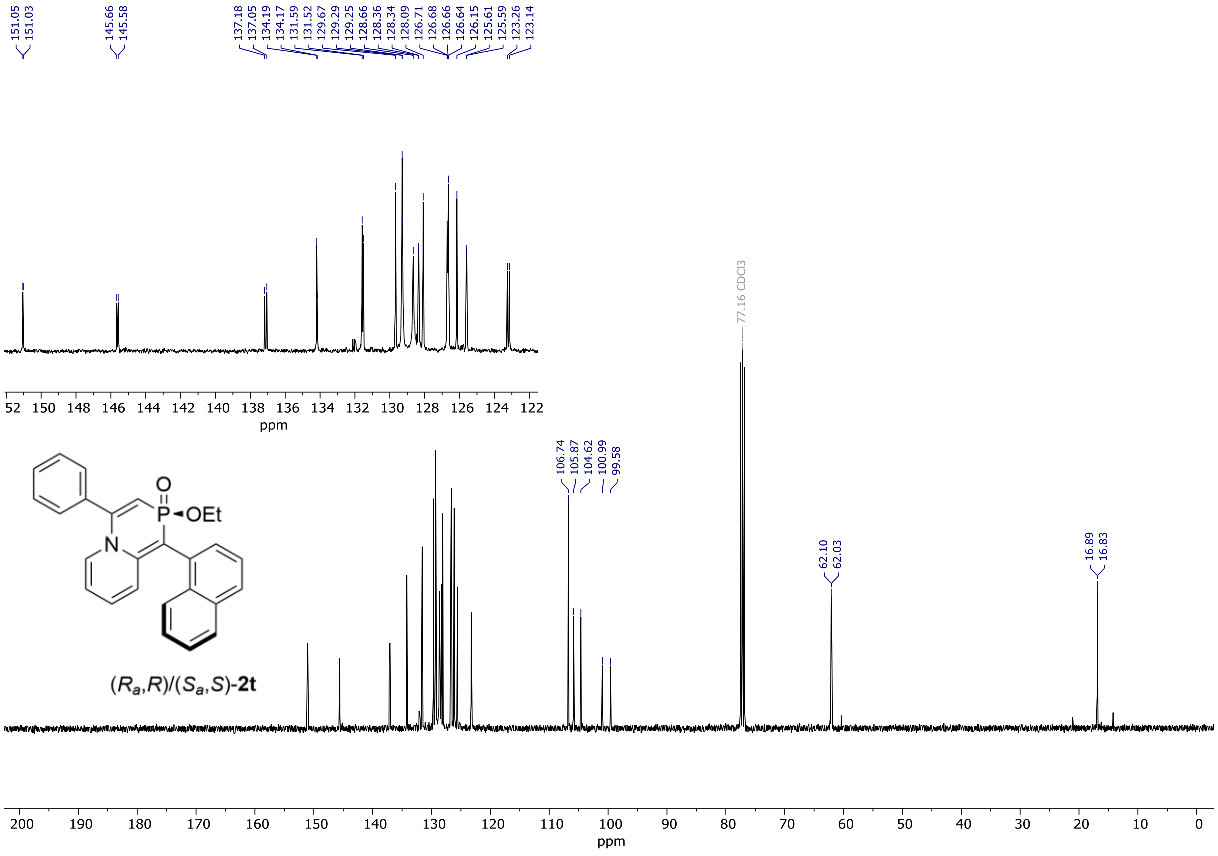

Figure S134 *^{31}P {^{1}H} NMR spectrum of (Rₐ,R)/(Sₐ,S)-**2t** (162 MHz, CDCl$_3$).*

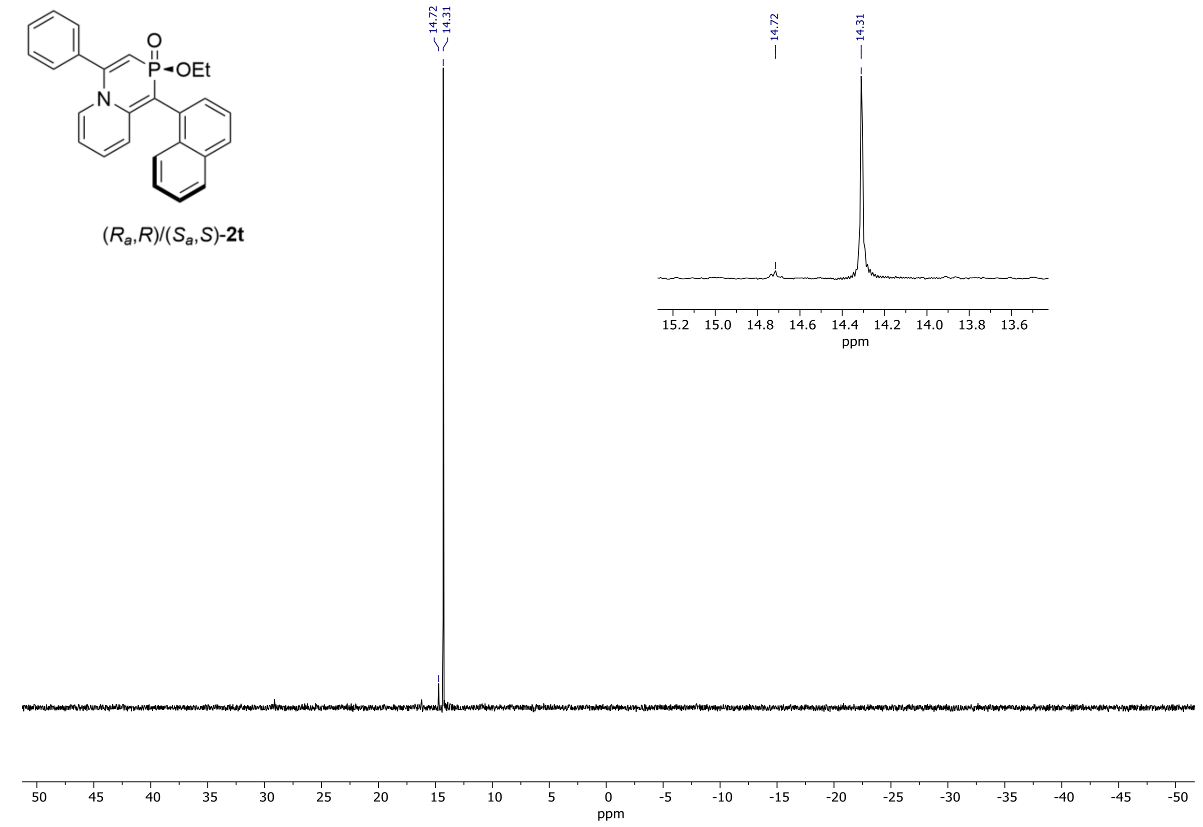

Figure S135 *^{31}P {^{1}H} NMR spectrum of (Rα,R)/(Sα,S)-**2t** (162 MHz, $CDCl_3$).*

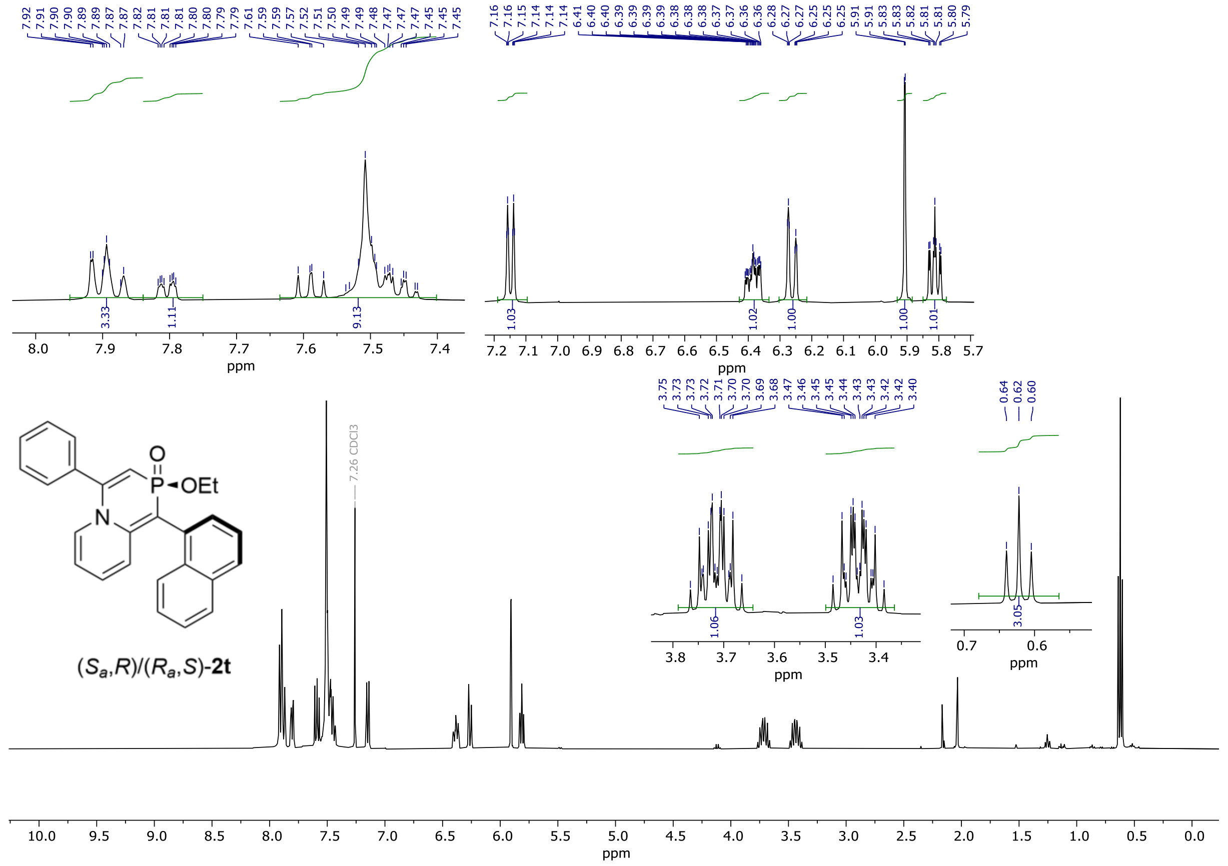

Figure S136 *^{1}H NMR spectrum of (Sα,R)/(Rα,S)-**2t** (400 MHz, $CDCl_3$).*

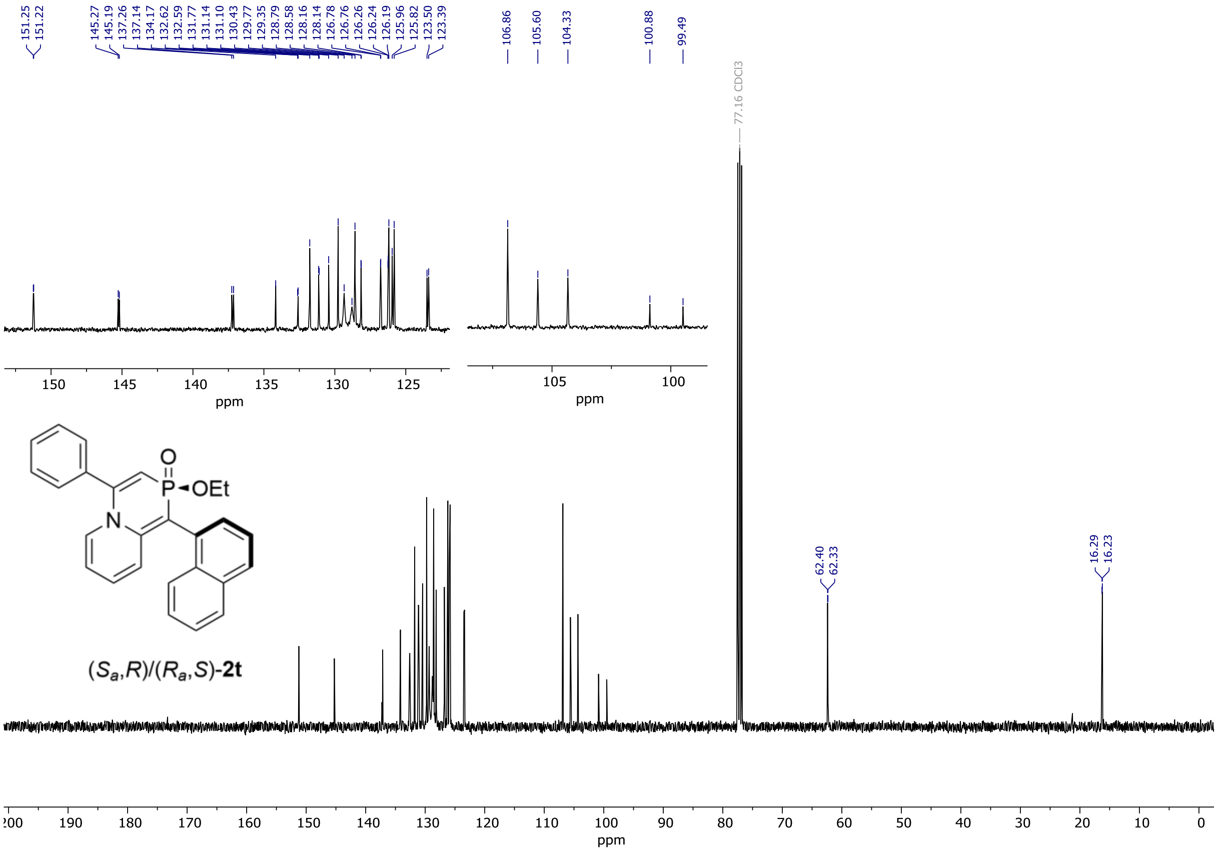

Figure S137 $^{13}C\{^1H\}$ NMR spectrum of (S_a,R)/(R_a,S)-**2t** (101 MHz, $CDCl_3$).

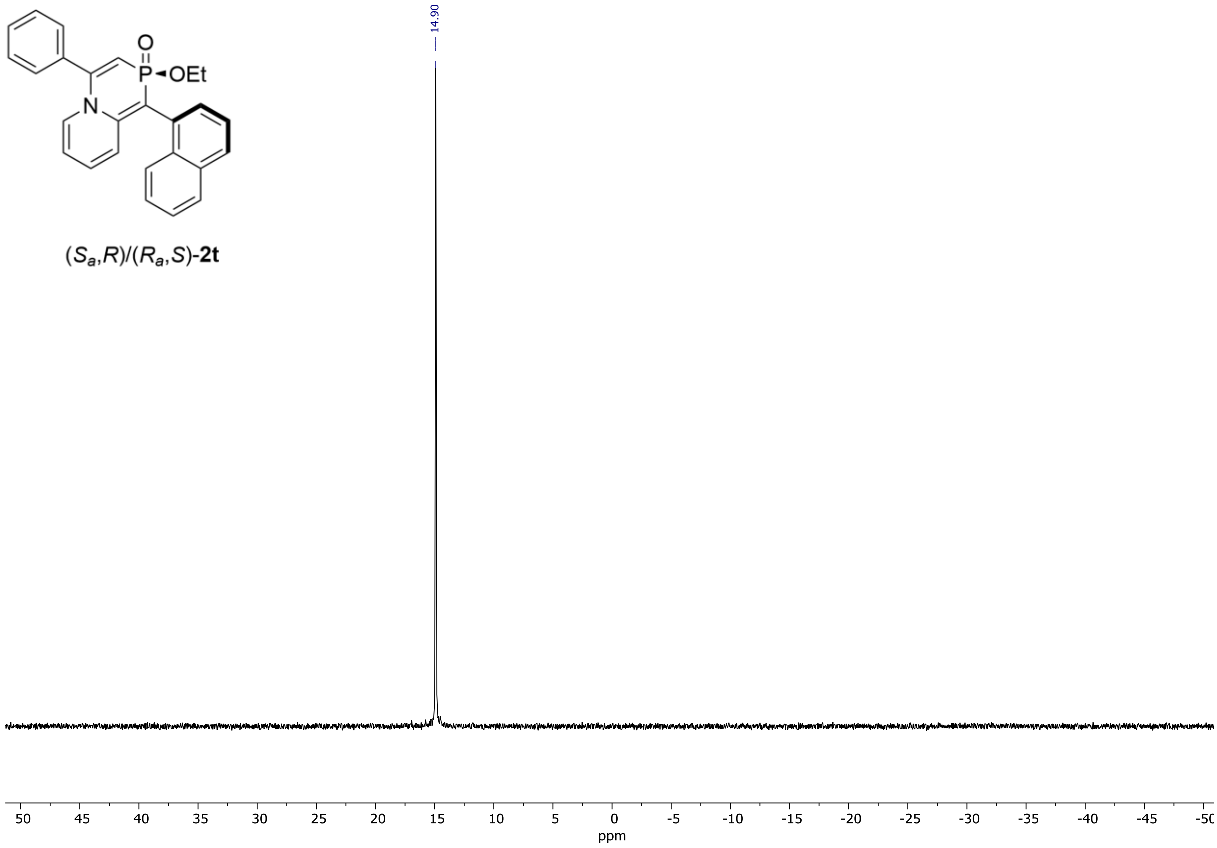

Figure S138 $^{31}P\{^1H\}$ NMR spectrum of (S_a,R)/(R_a,S)-**2t** (162 MHz, $CDCl_3$).

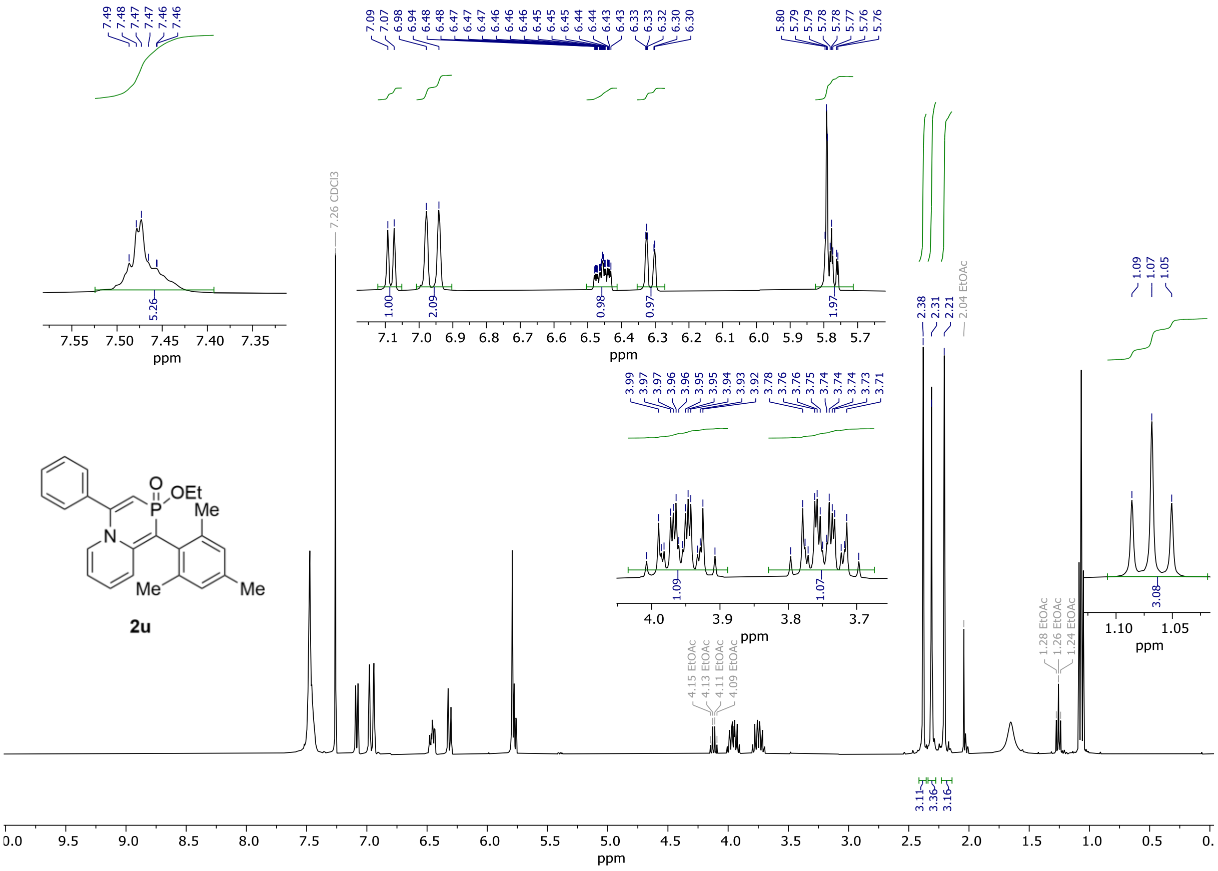

Figure S139 *^{1}H NMR spectrum of **2u** (400 MHz, $CDCl_3$).*

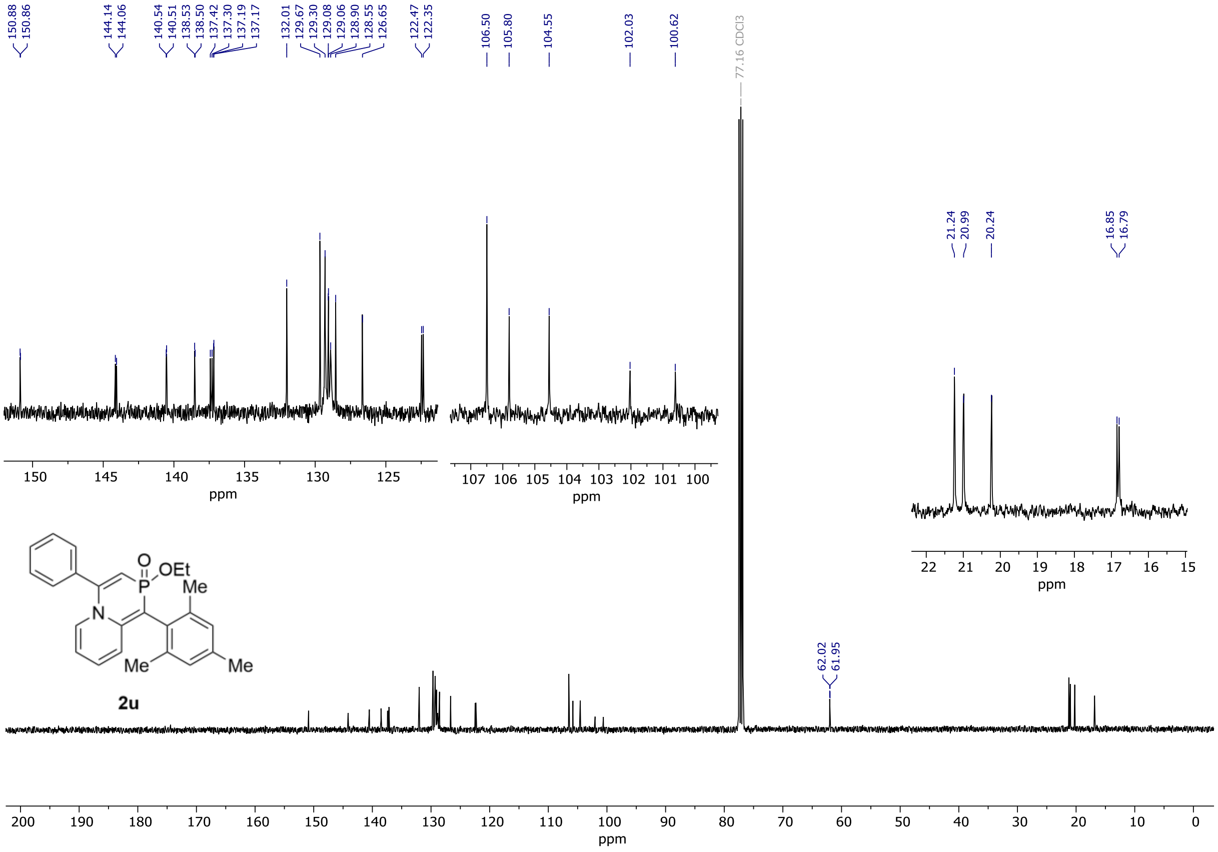

Figure S140 *^{13}C {^{1}H} NMR spectrum of **2u** (101 MHz, $CDCl_3$).*

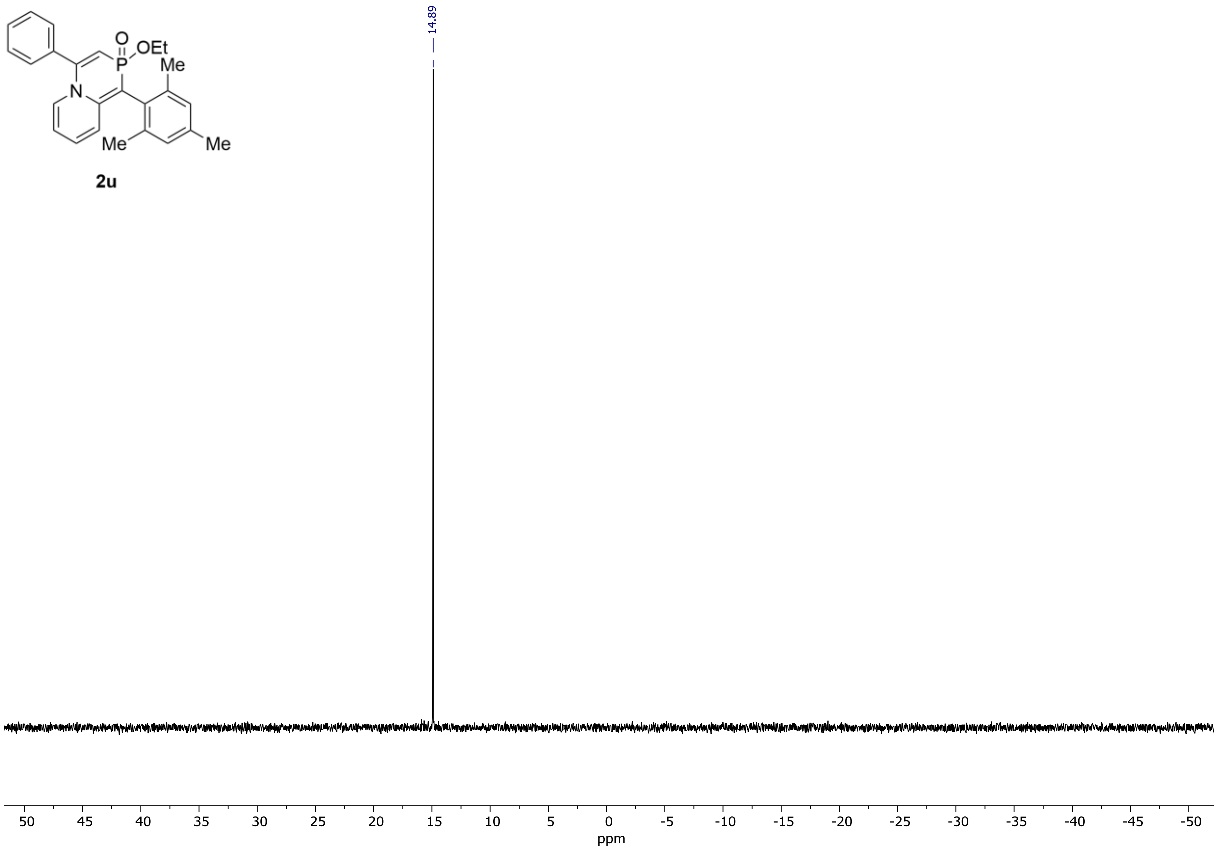

Figure S141 $^{31}P\ \{^{1}H\}$ *NMR spectrum of* ***2u*** *(162 MHz, $CDCl_3$).*

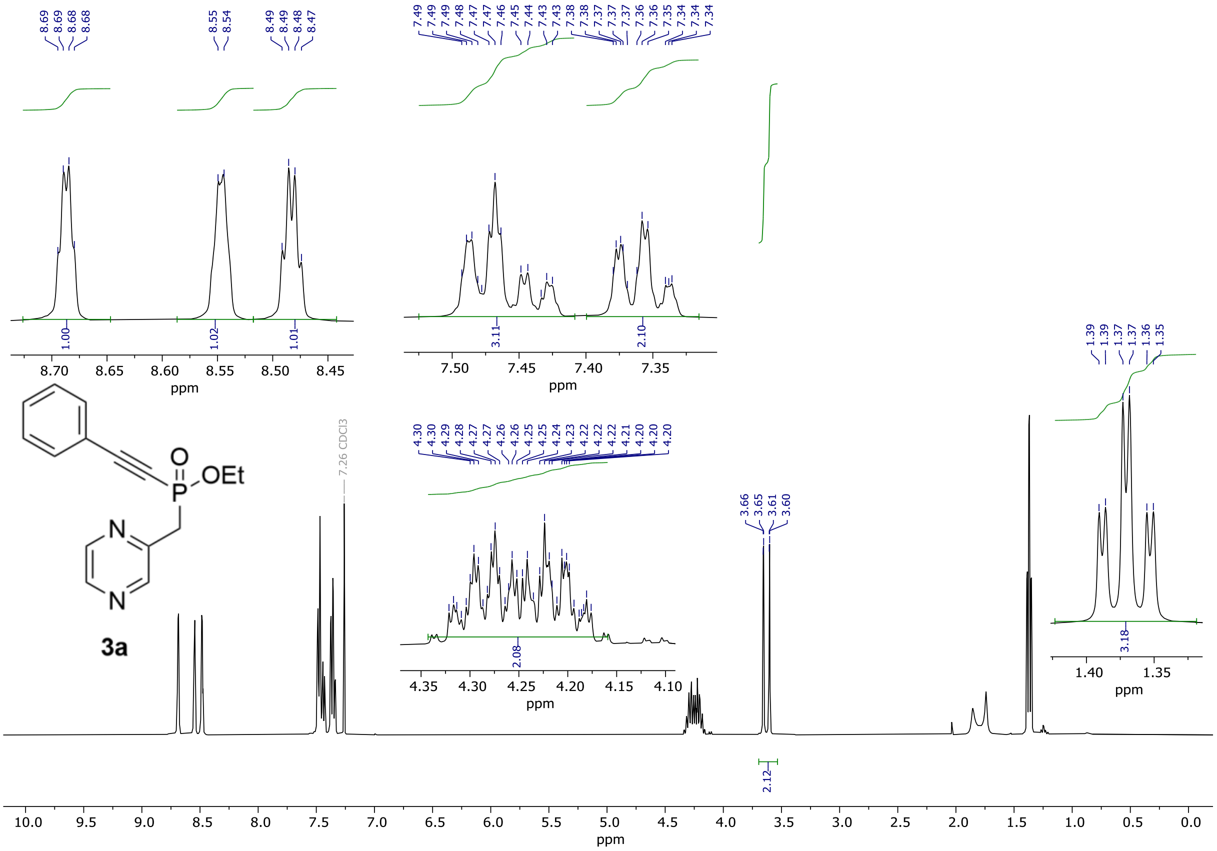

Figure S142 ^{1}H *NMR spectrum of* ***3a*** *(400 MHz, $CDCl_3$).*

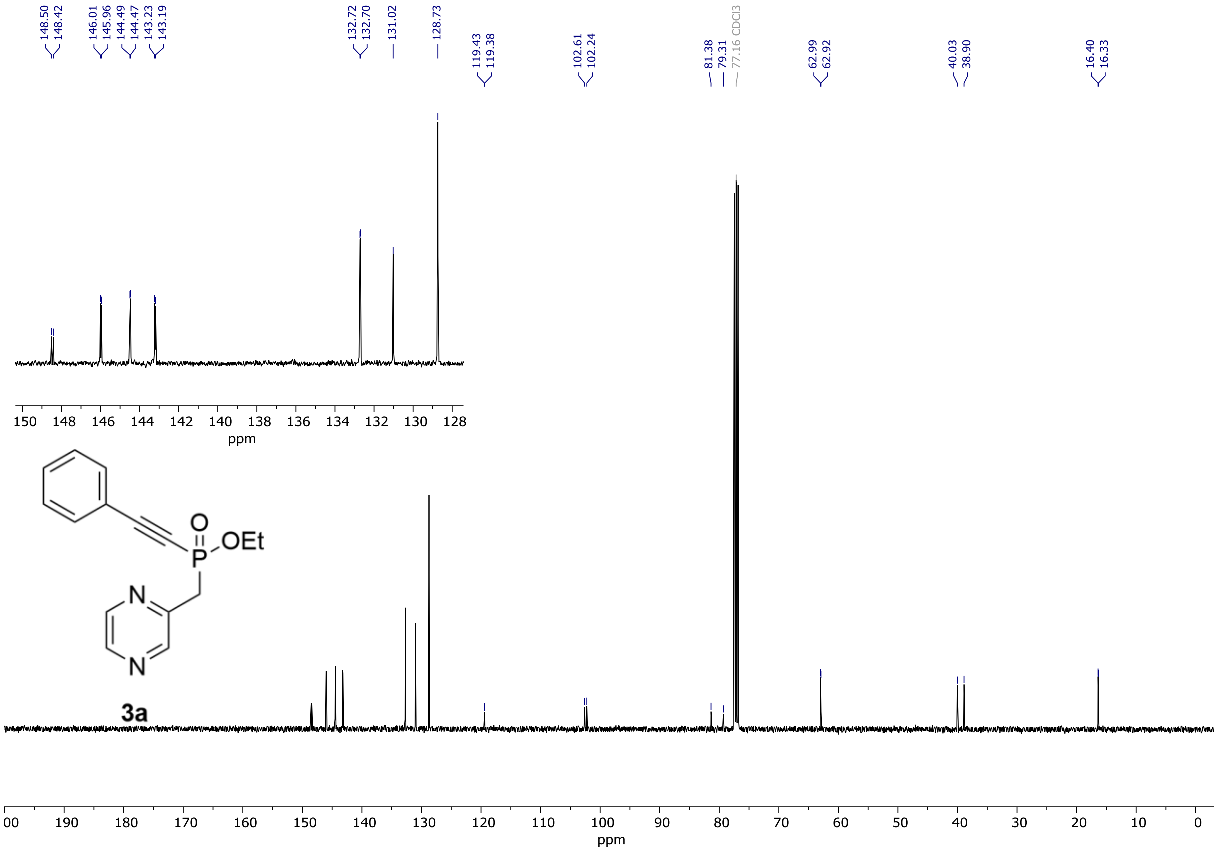

Figure S143 ^{13}C {^1H} *NMR spectrum of **3a** (101 MHz, $CDCl_3$).*

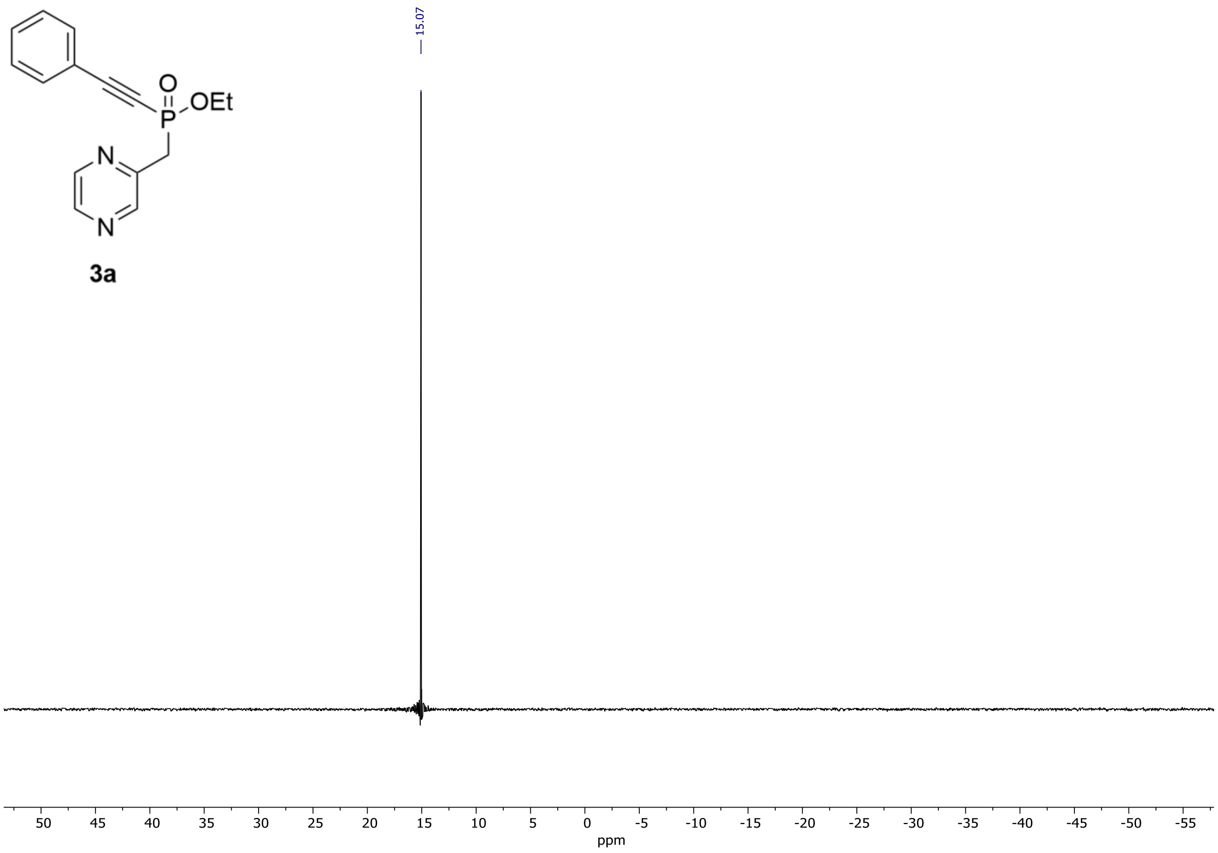

Figure S144 ^{31}P {^1H} *NMR spectrum of **3a** (162 MHz, $CDCl_3$).*

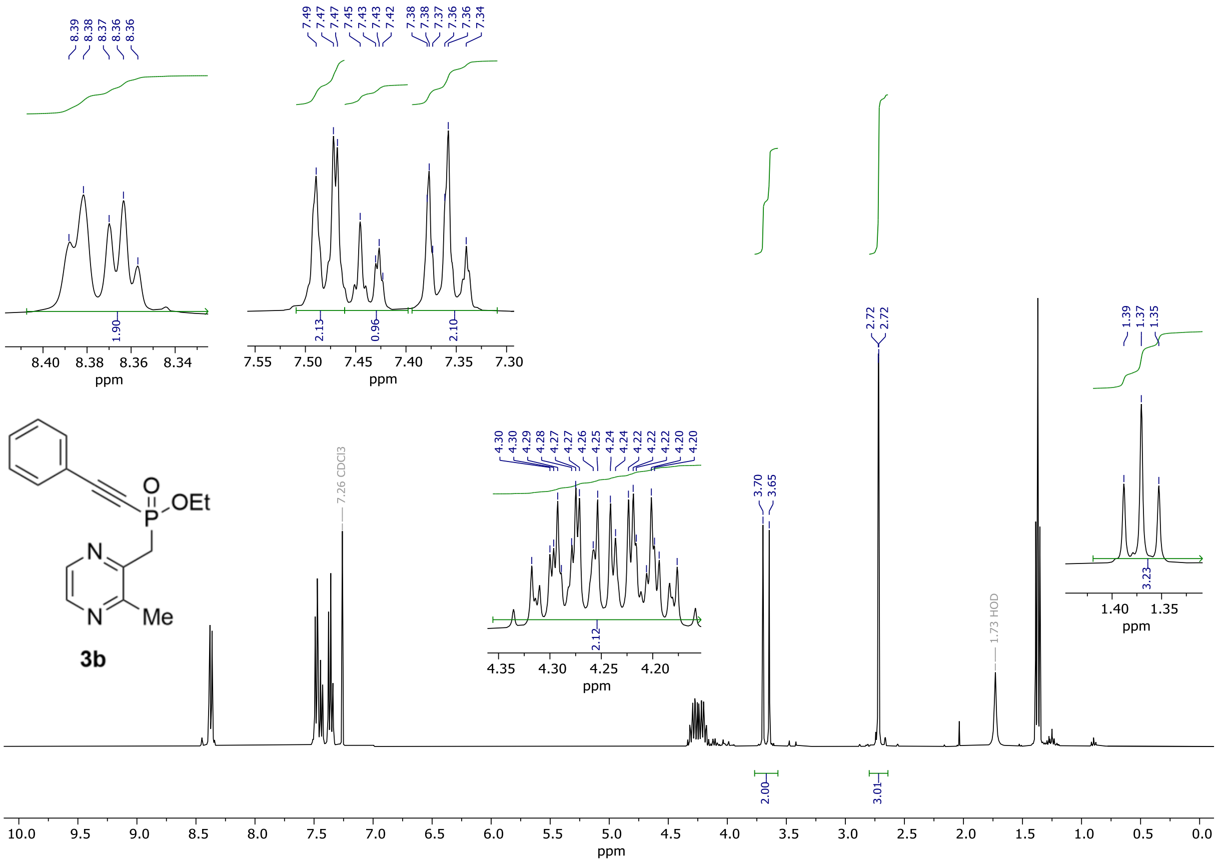

Figure S145 ^{1}H *NMR spectrum of **3b** (400 MHz, $CDCl_3$).*

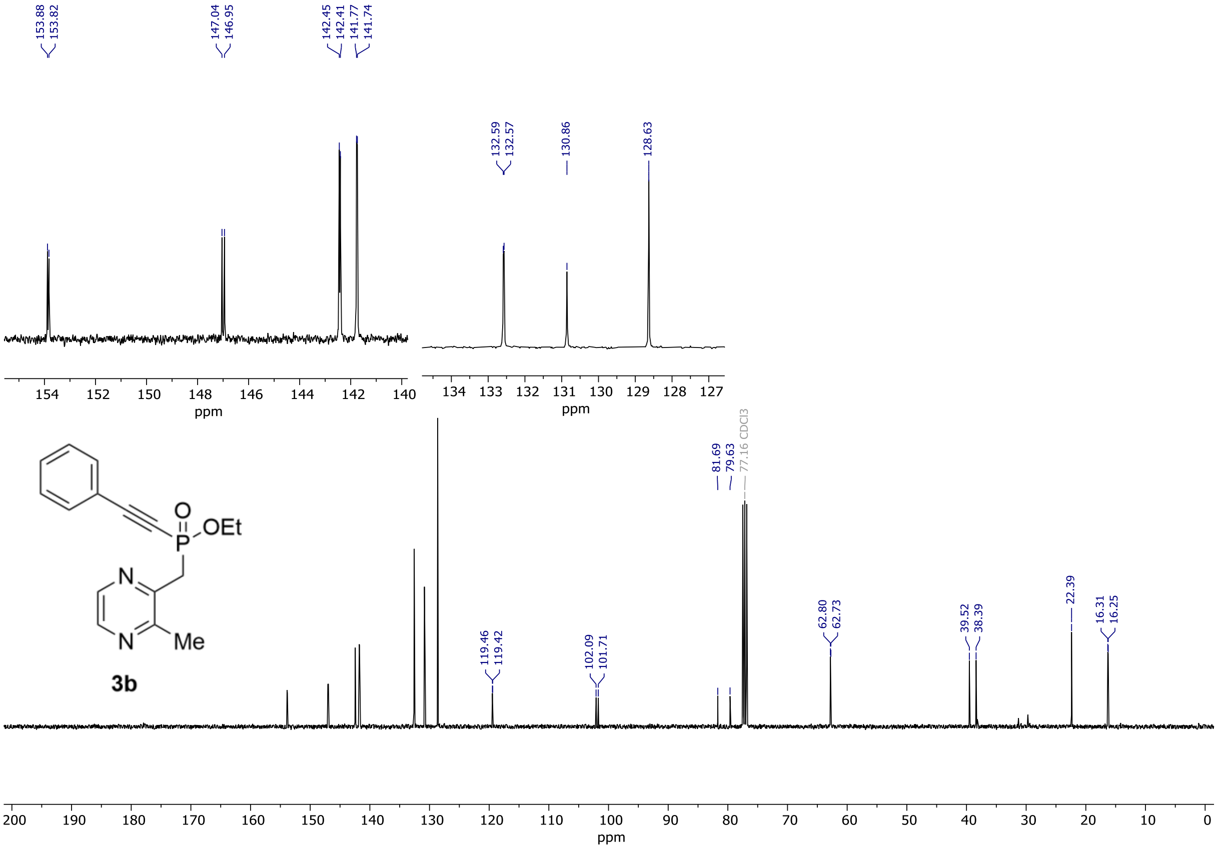

Figure S146 $^{13}C\ \{^{1}H\}$ *NMR spectrum of **3b** (101 MHz, $CDCl_3$).*

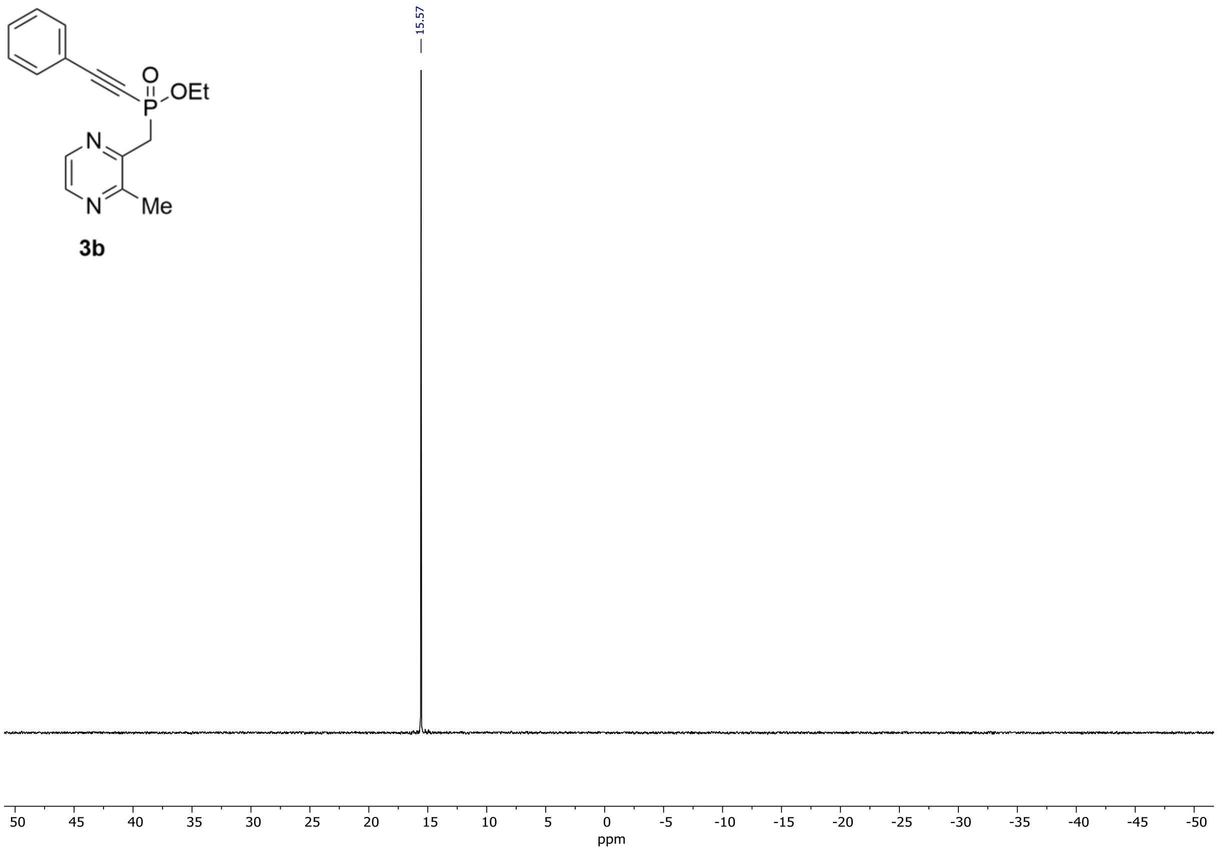

Figure S147 ^{31}P {^{1}H} *NMR spectrum of* ***3b*** *(162 MHz, $CDCl_3$).*

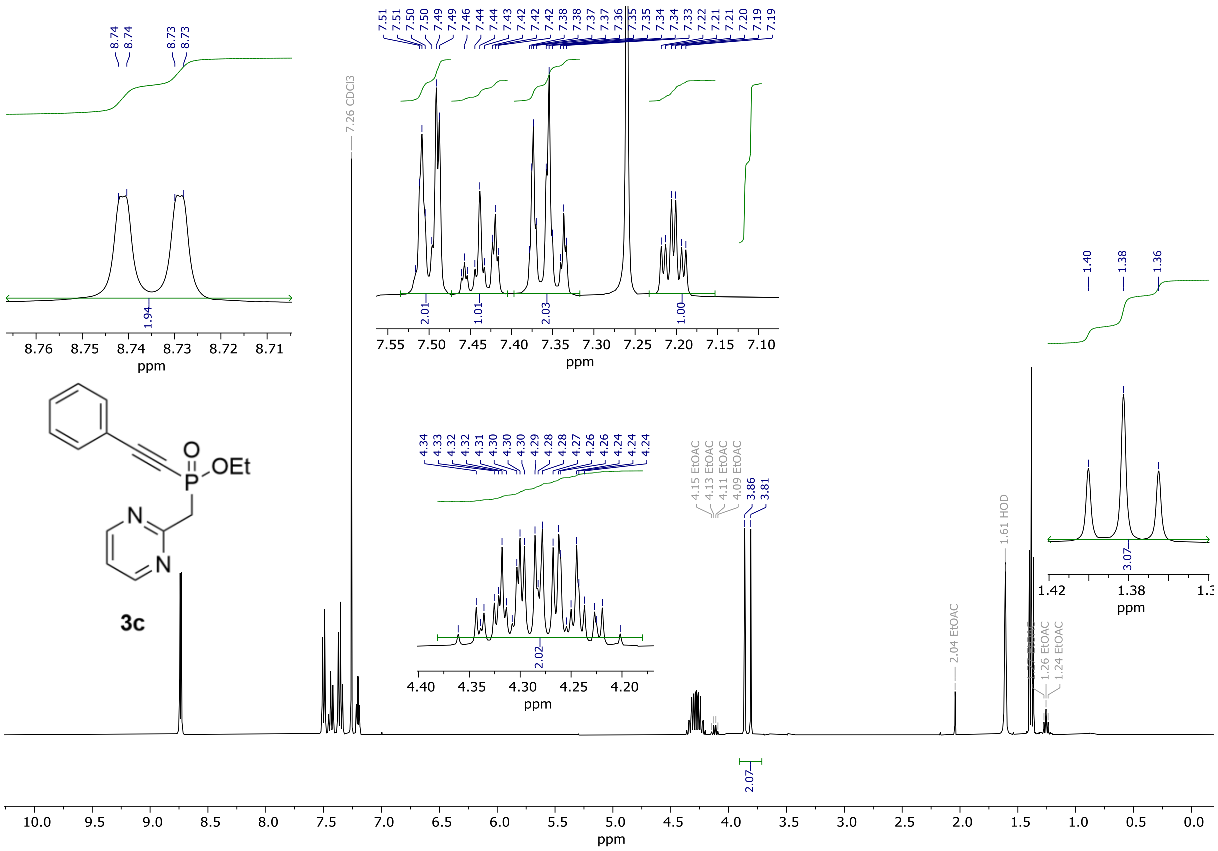

Figure S148 ^{1}H *NMR spectrum of* ***3c*** *(400 MHz, $CDCl_3$).*

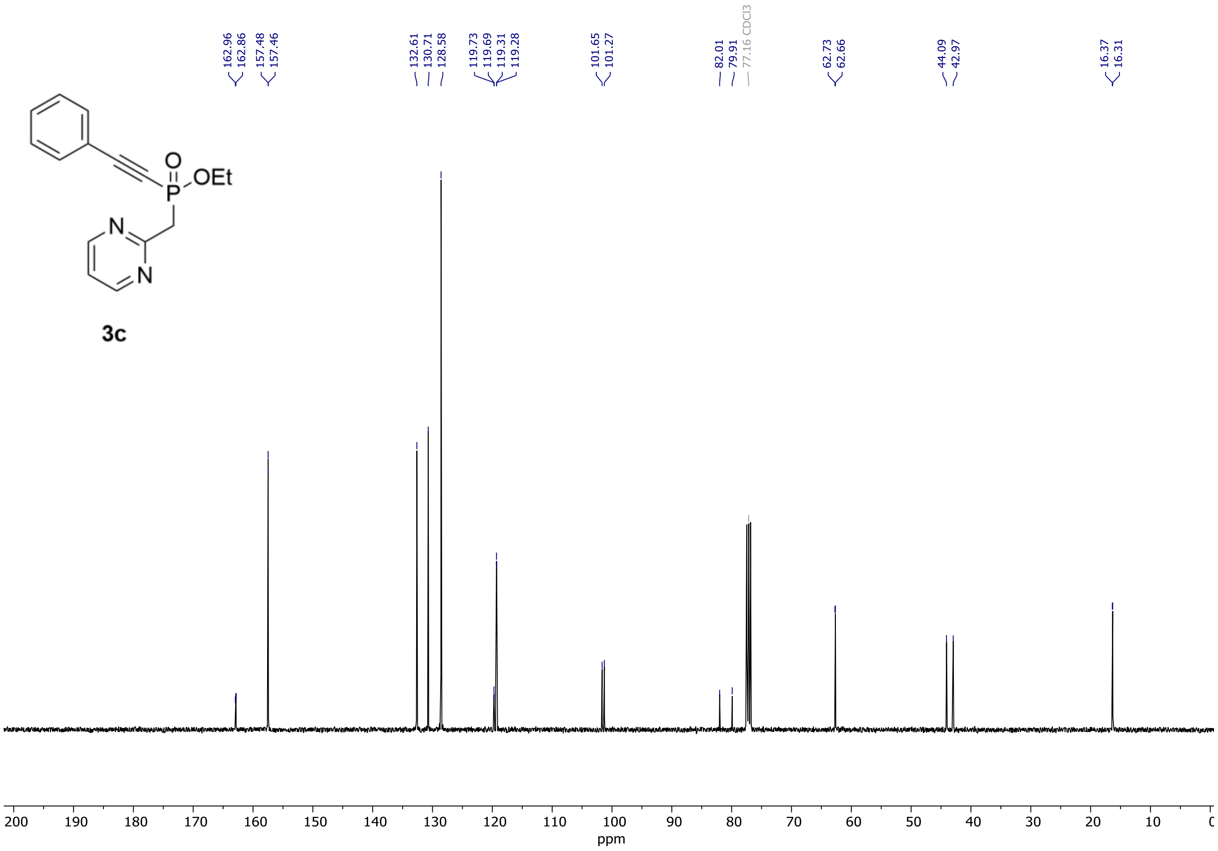

Figure S149 ^{13}*C {*^{1}*H} NMR spectrum of* ***3c*** *(101 MHz,* $CDCl_3$*).*

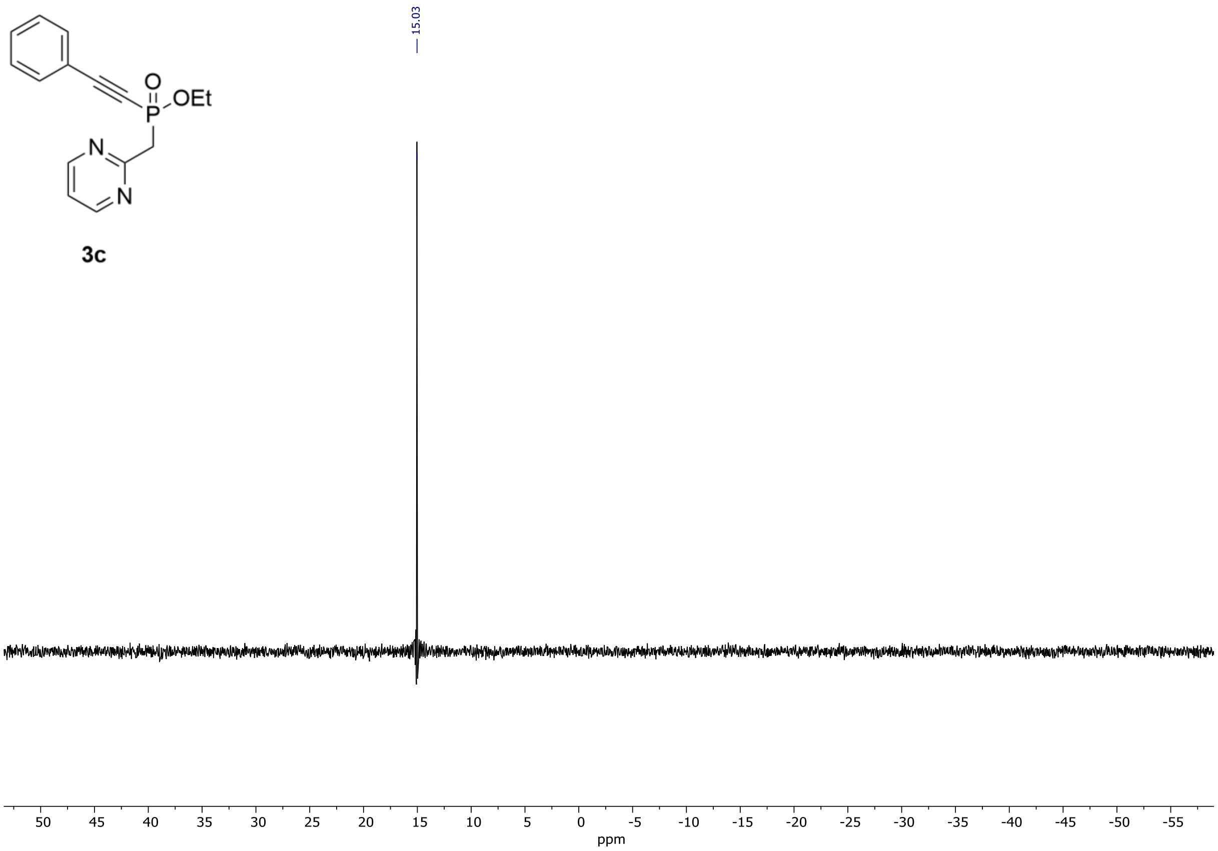

Figure S150 ^{31}*P {*^{1}*H} NMR spectrum of* ***3c*** *(162 MHz,* $CDCl_3$*).*

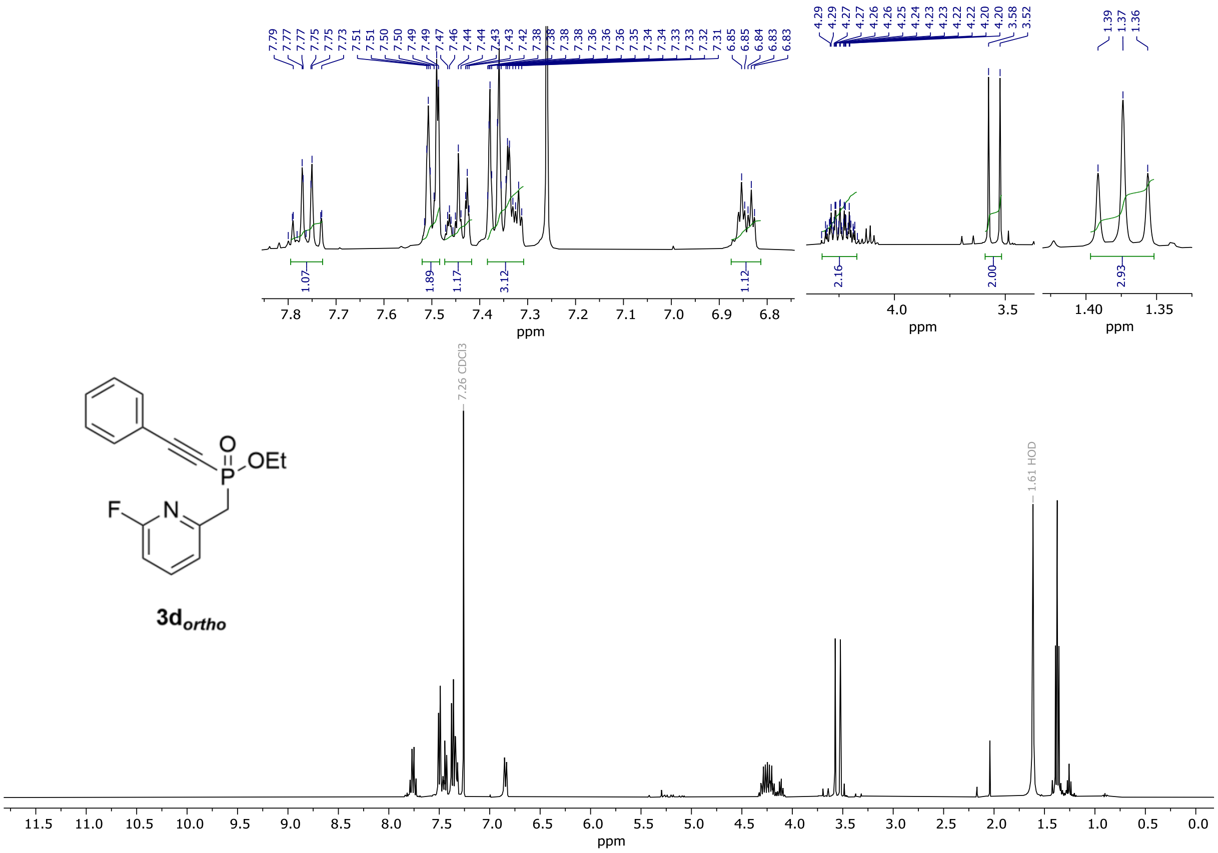

Figure S151 *^{1}H NMR spectrum of **3d**$_{\textbf{ortho}}$ (400 MHz, CDCl$_3$).*

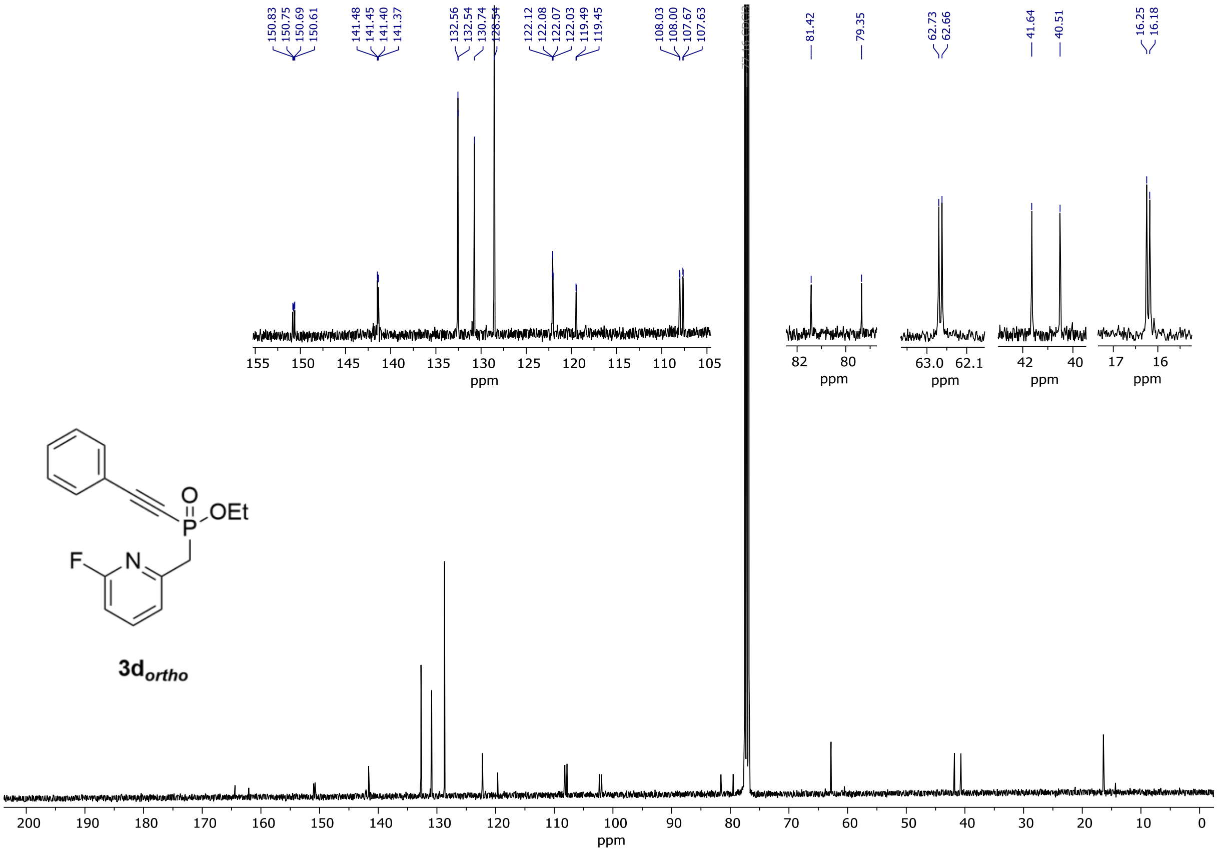

Figure S152 *^{13}C {^{1}H} NMR spectrum of **3d**$_{\textbf{ortho}}$ (101 MHz, CDCl$_3$).*

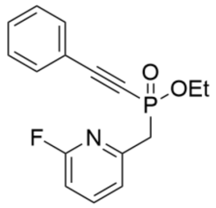

3d*ortho*

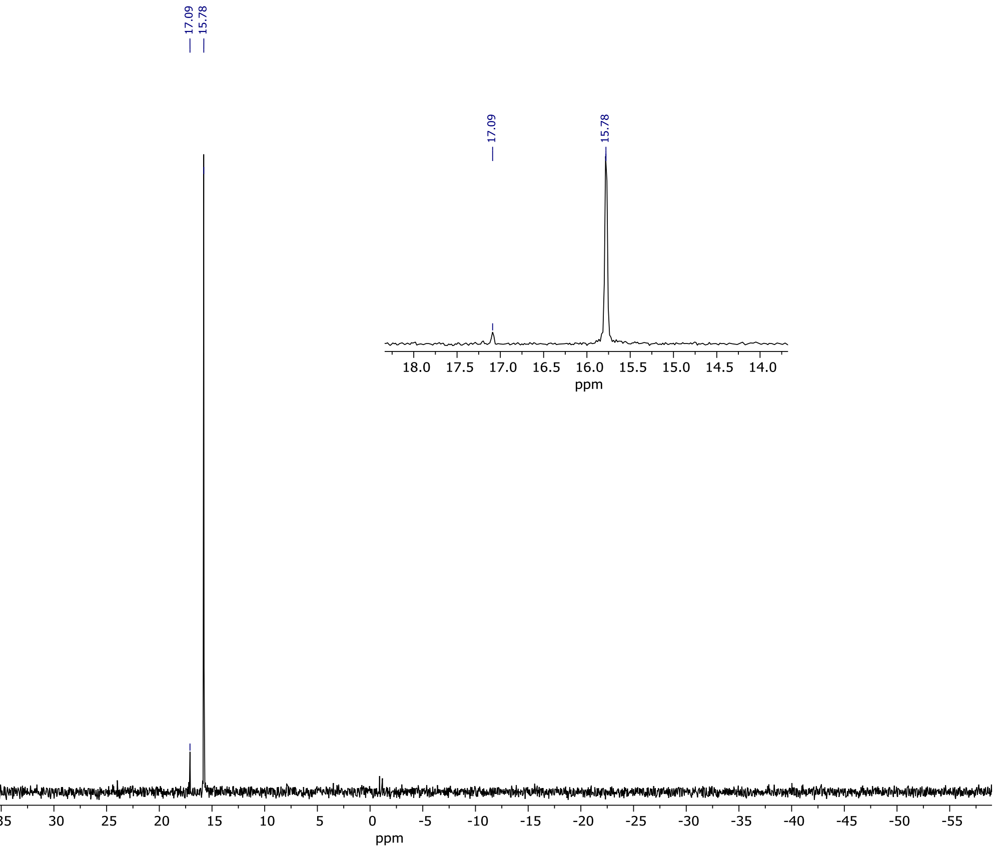

Figure S153 *^{31}P {^{1}H} NMR spectrum of* ***3d*** ***ortho*** *(162 MHz, $CDCl_3$).*

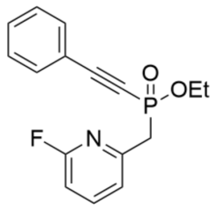

3d*ortho*

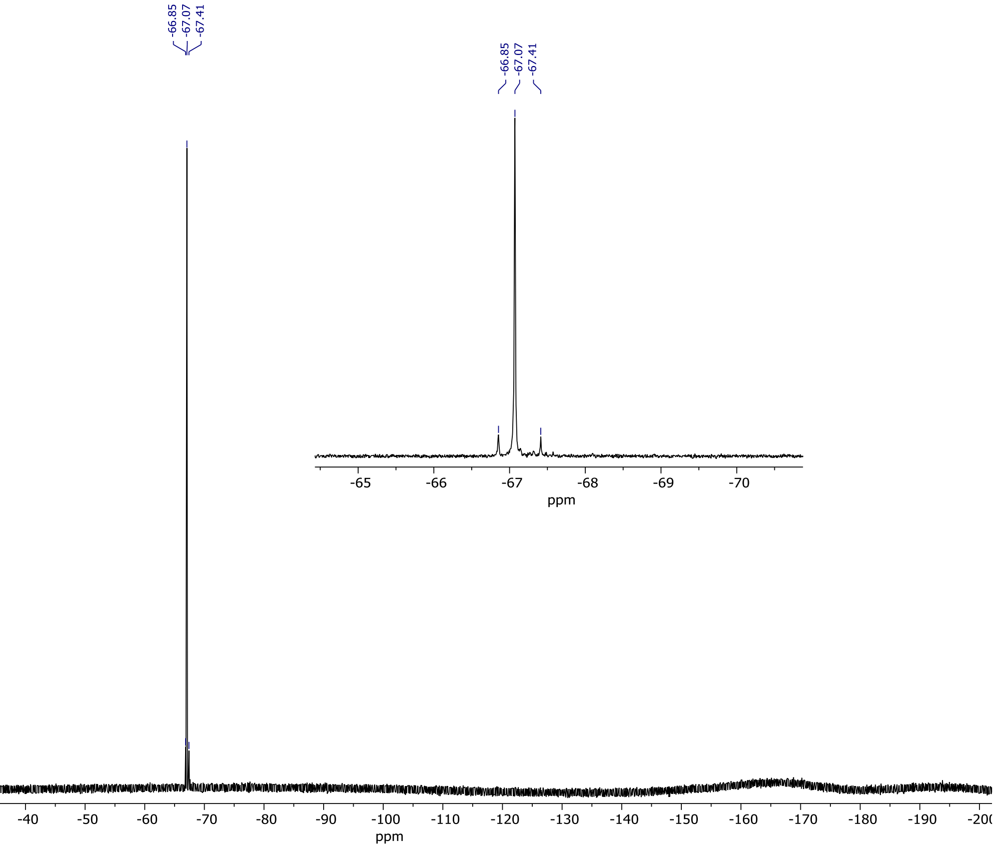

Figure S154 *^{19}F {^{1}H} NMR spectrum of* ***3d*** ***ortho*** *(376 MHz, $CDCl_3$).*

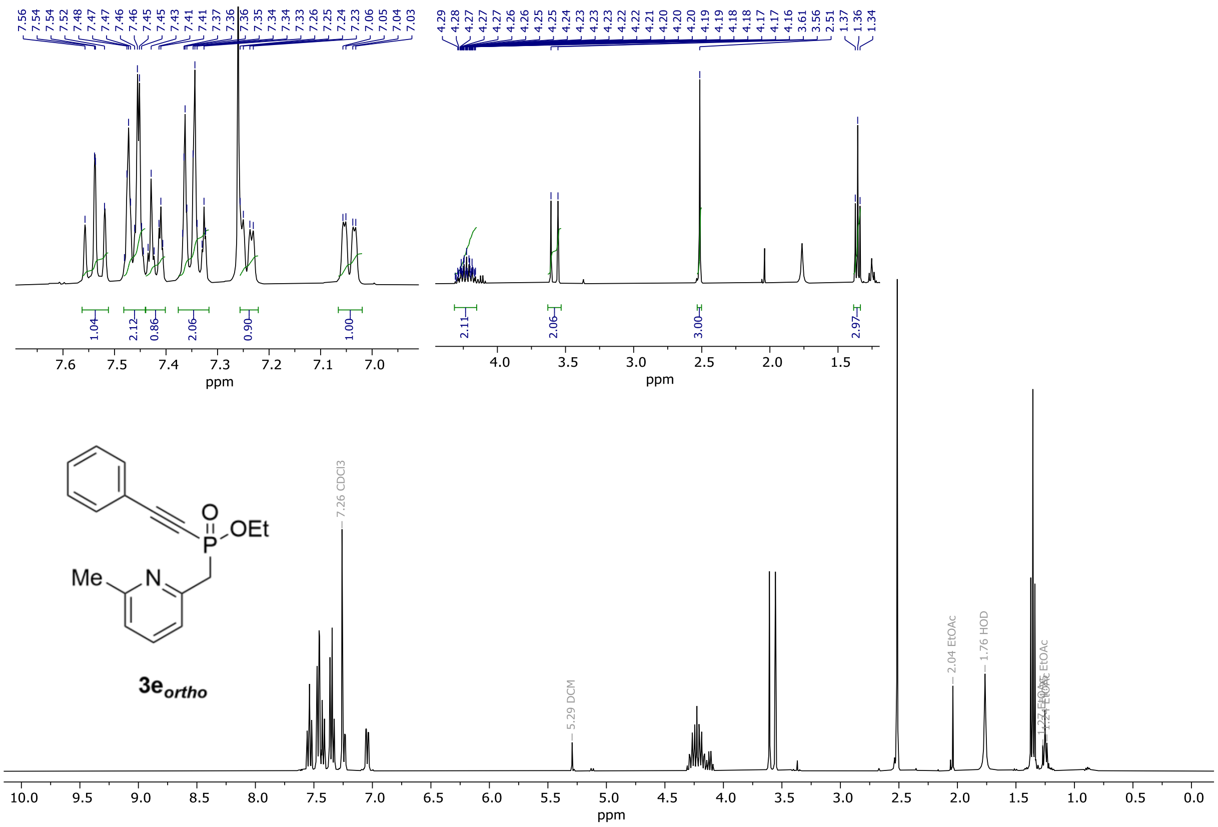

Figure S155 *^{1}H NMR spectrum of* ***3e****$_{ortho}$* *(400 MHz, CDCl$_3$).*

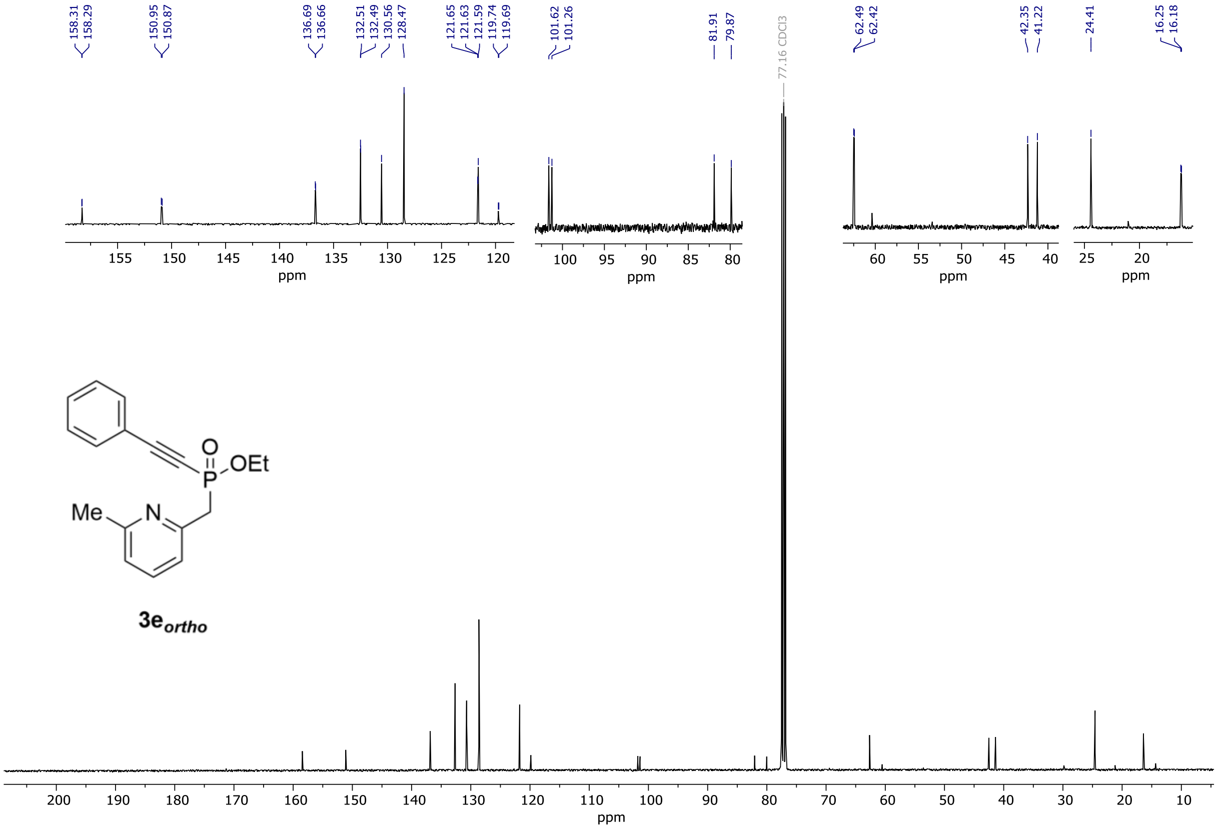

Figure S156 *^{13}C {^{1}H} NMR spectrum of* ***3e****$_{ortho}$* *(101 MHz, CDCl$_3$).*

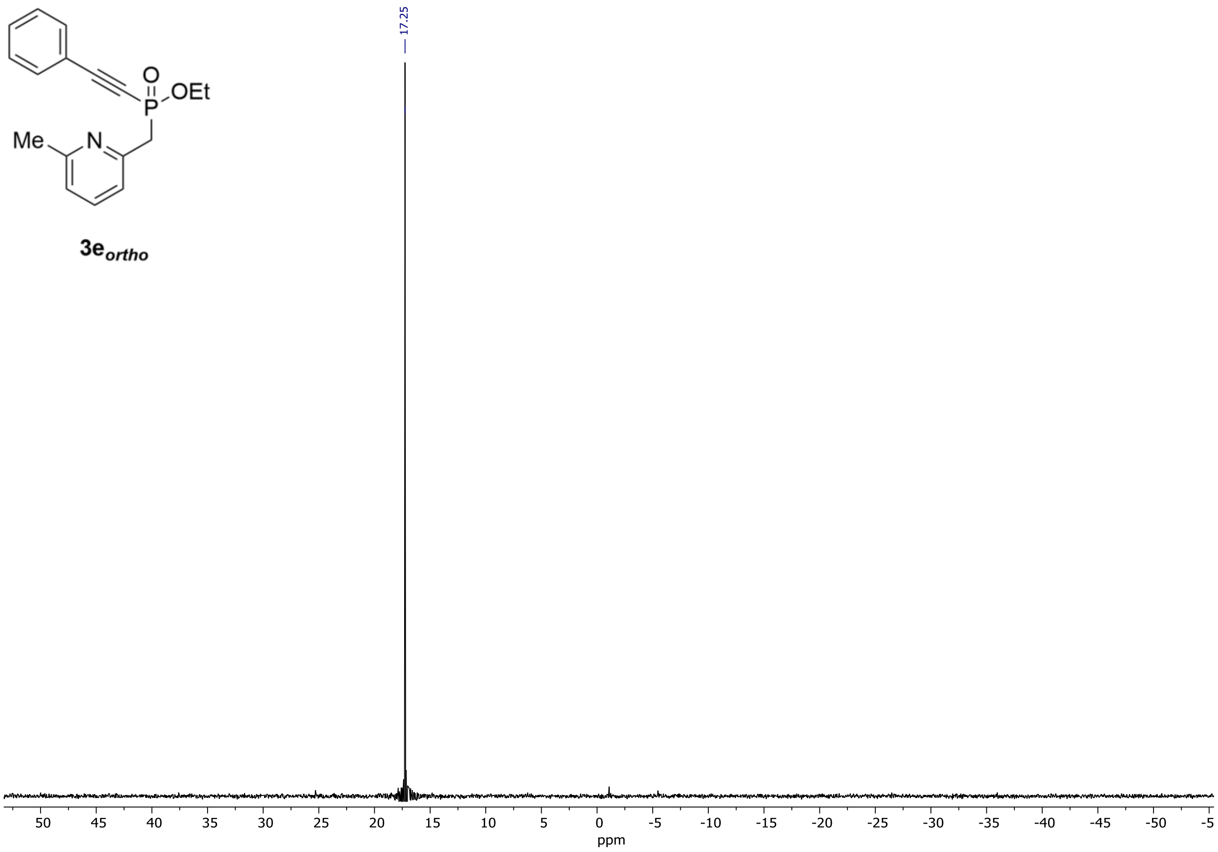

Figure S157 ^{31}P {1H} NMR spectrum of **3e$_{ortho}$** (162 MHz, $CDCl_3$).

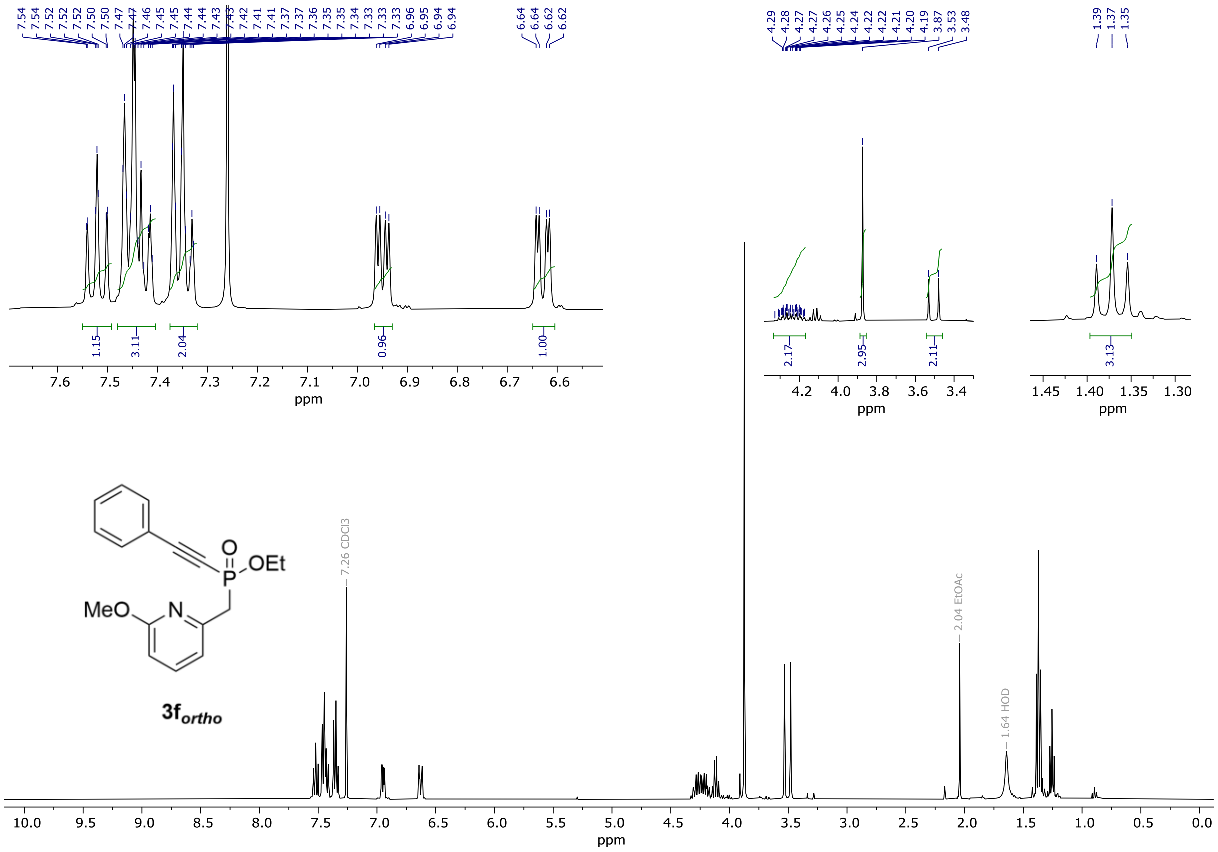

Figure S158 ^{1}H NMR spectrum of **3f$_{ortho}$** (400 MHz, $CDCl_3$).

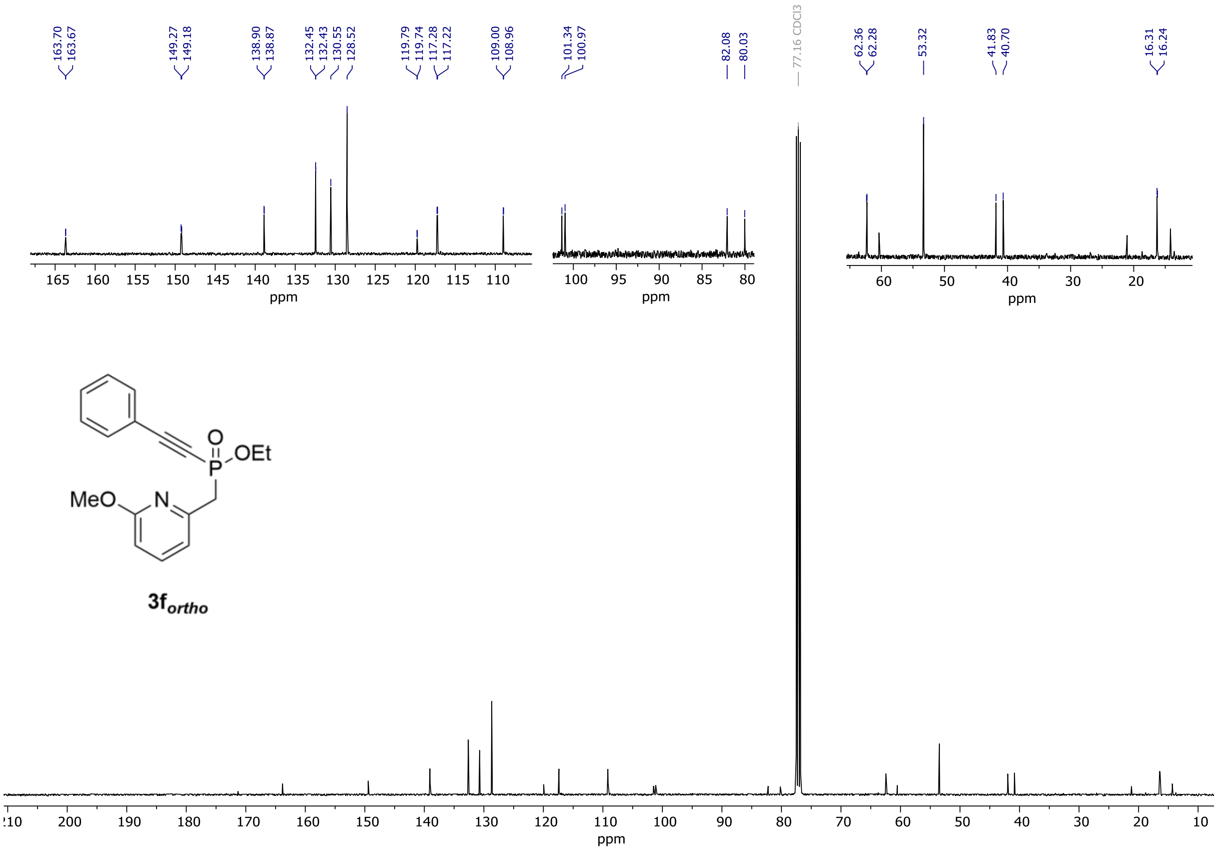

Figure S159 *^{13}C {^{1}H} NMR spectrum of **$3f_{ortho}$** (101 MHz, $CDCl_3$).*

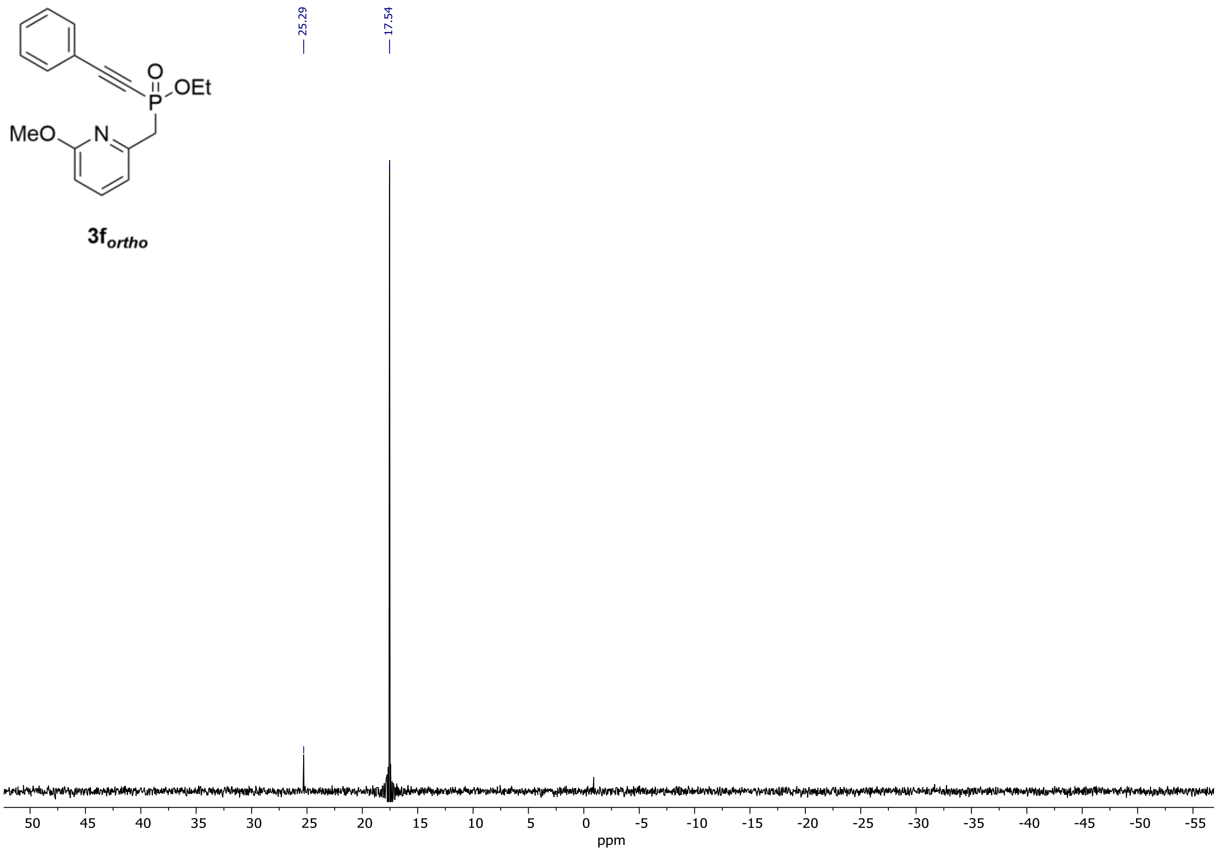

Figure S160 *^{31}P {^{1}H} NMR spectrum of **$3f_{ortho}$** (162 MHz, $CDCl_3$).*

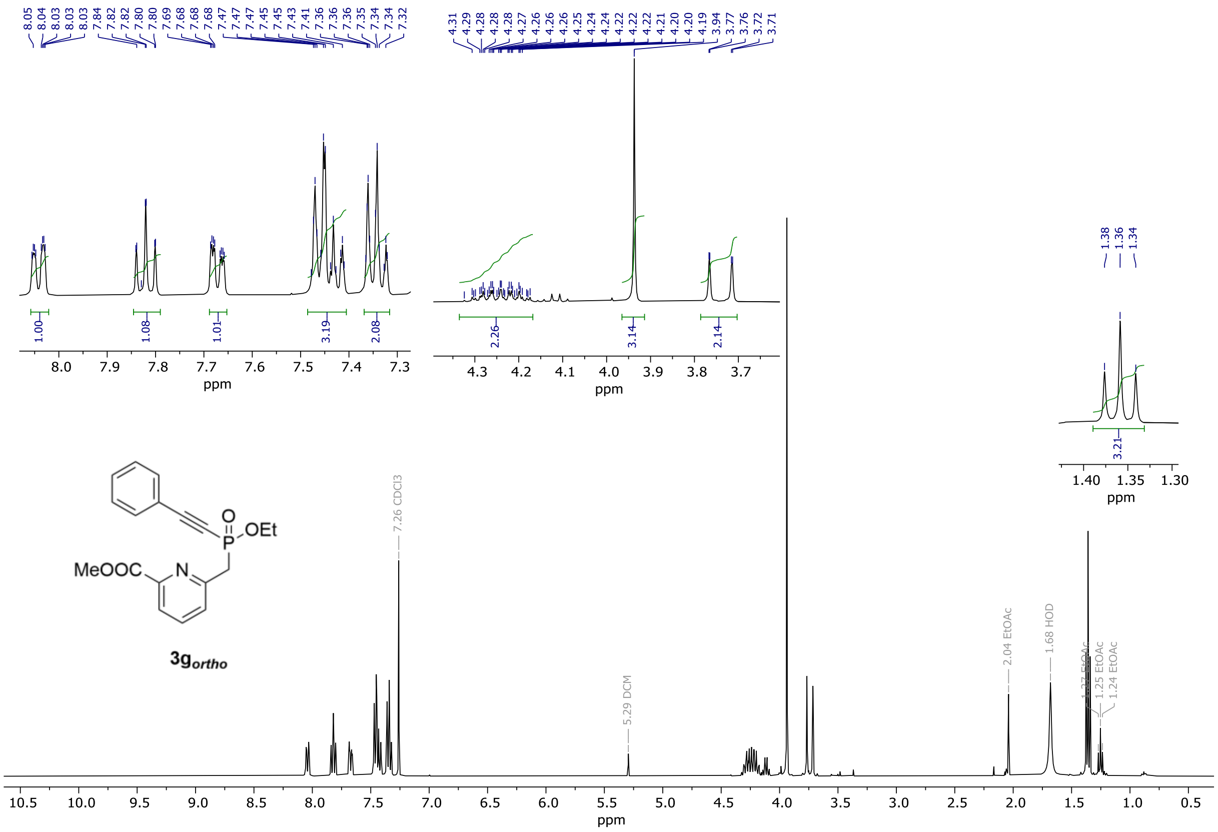

Figure S161 ^{1}H NMR spectrum of **3g**$_{ortho}$ (400 MHz, $CDCl_3$).

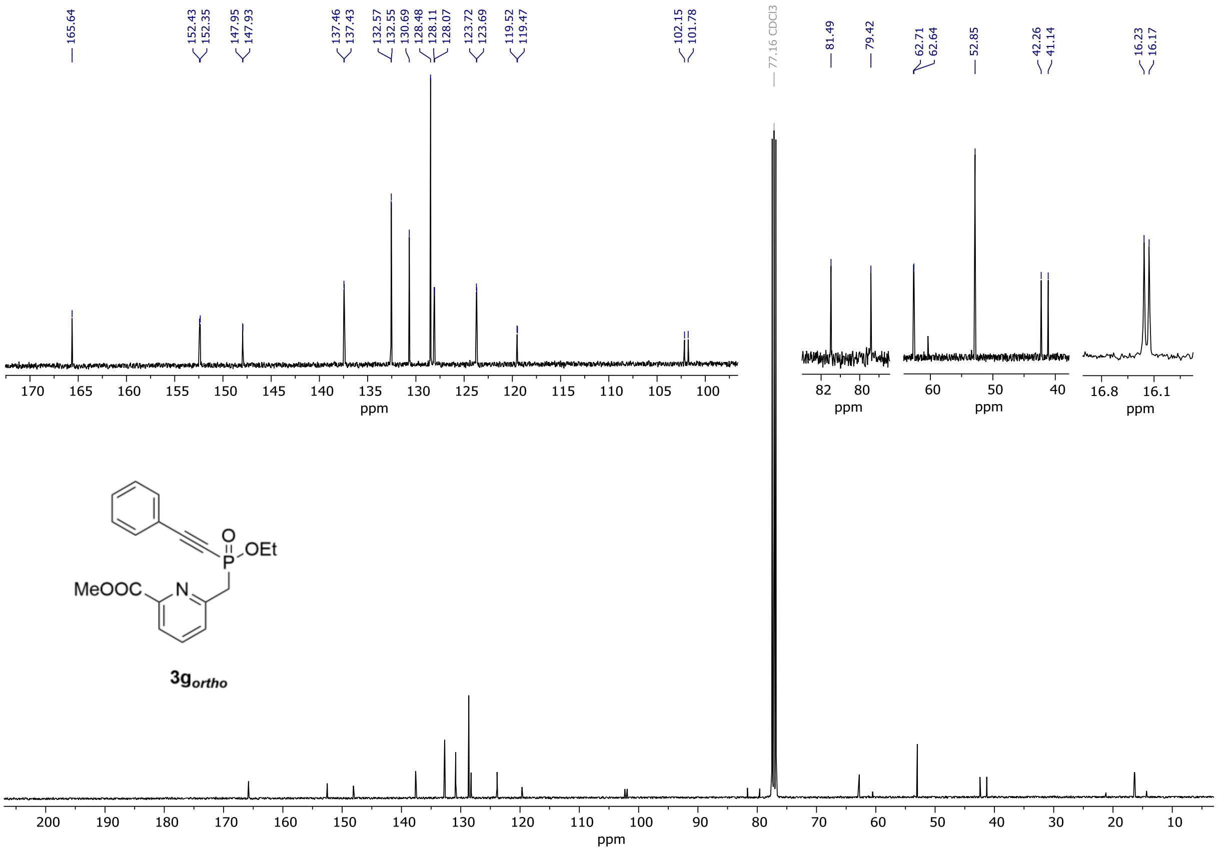

Figure S162 ^{13}C {^{1}H} NMR spectrum of **3g**$_{ortho}$ (101 MHz, $CDCl_3$).

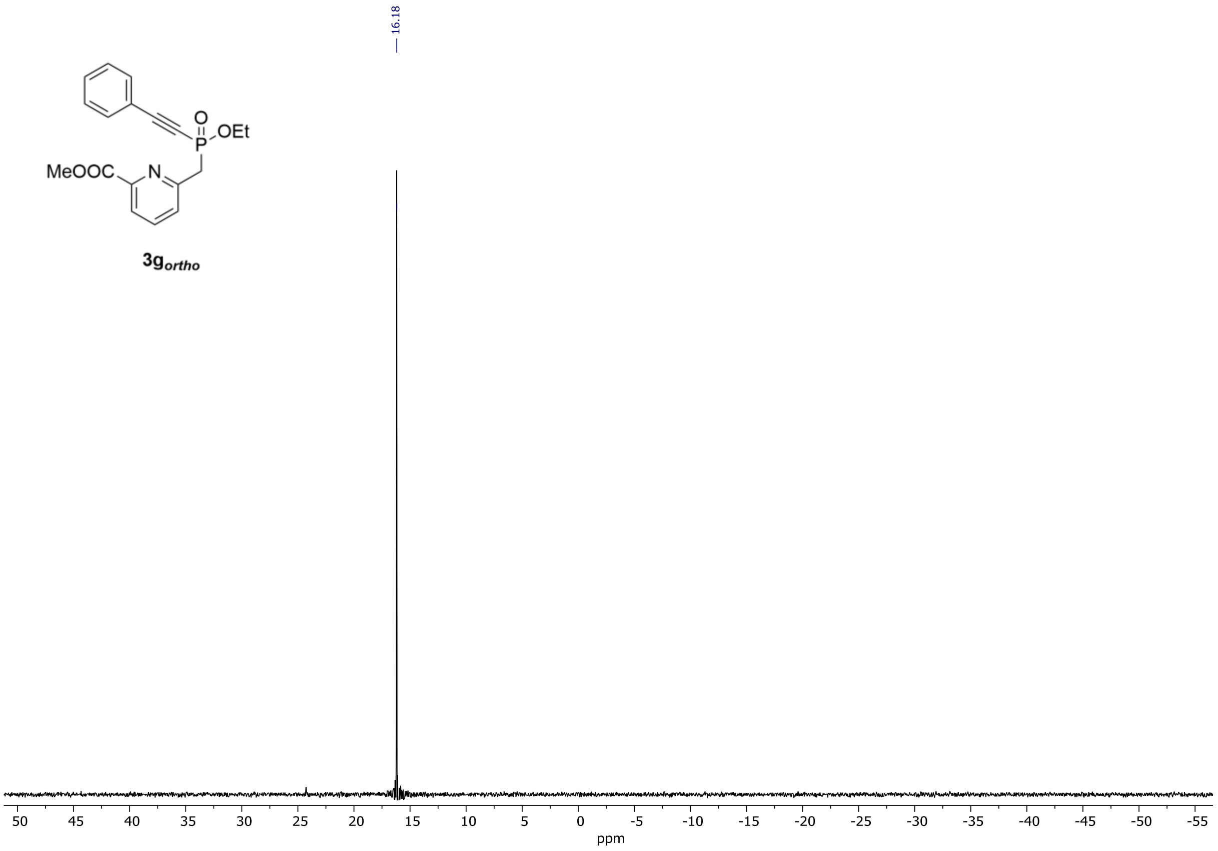

Figure S163 ^{31}P $\{^1H\}$ *NMR spectrum of* ***3g*** $_{\textbf{\textit{ortho}}}$ *(162 MHz,* $CDCl_3$*).*

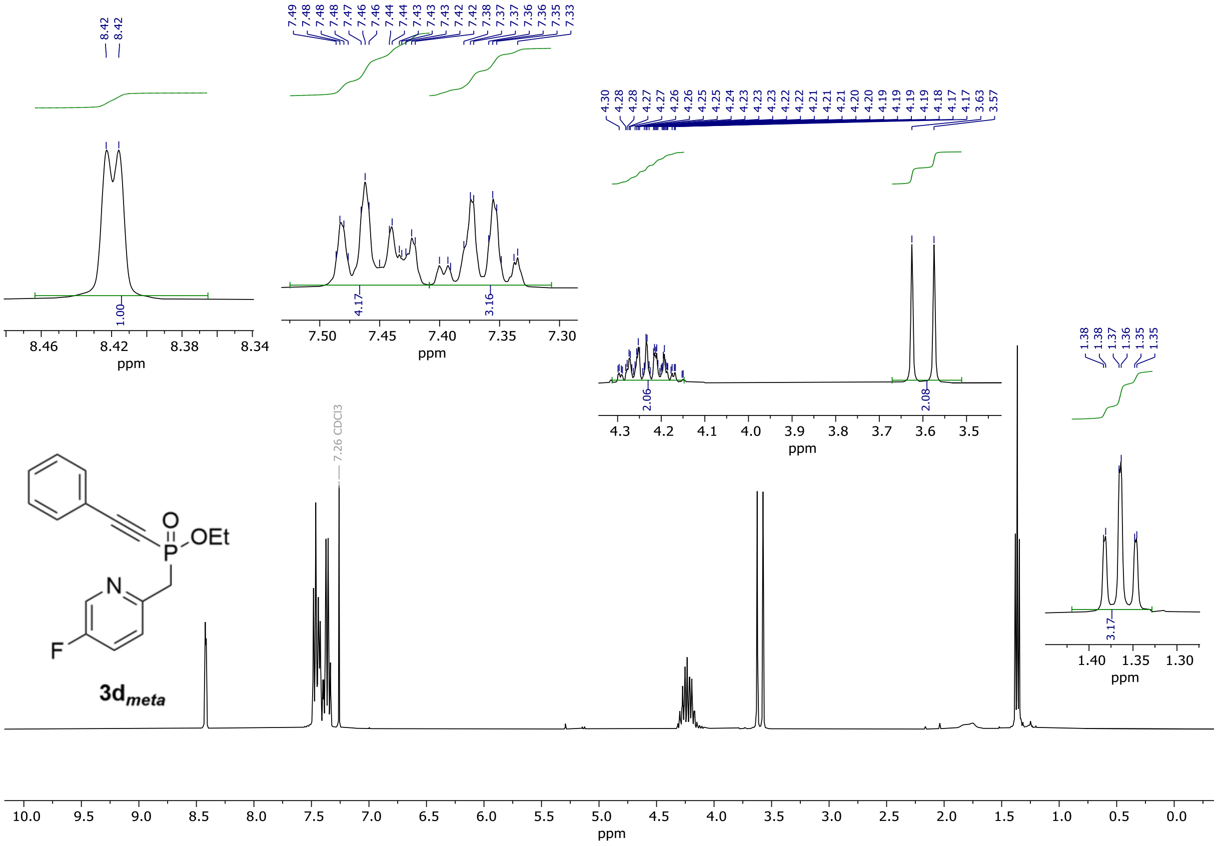

Figure S164 ^1H *NMR spectrum of* ***3d*** $_{\textbf{\textit{meta}}}$ *(400 MHz,* $CDCl_3$*).*

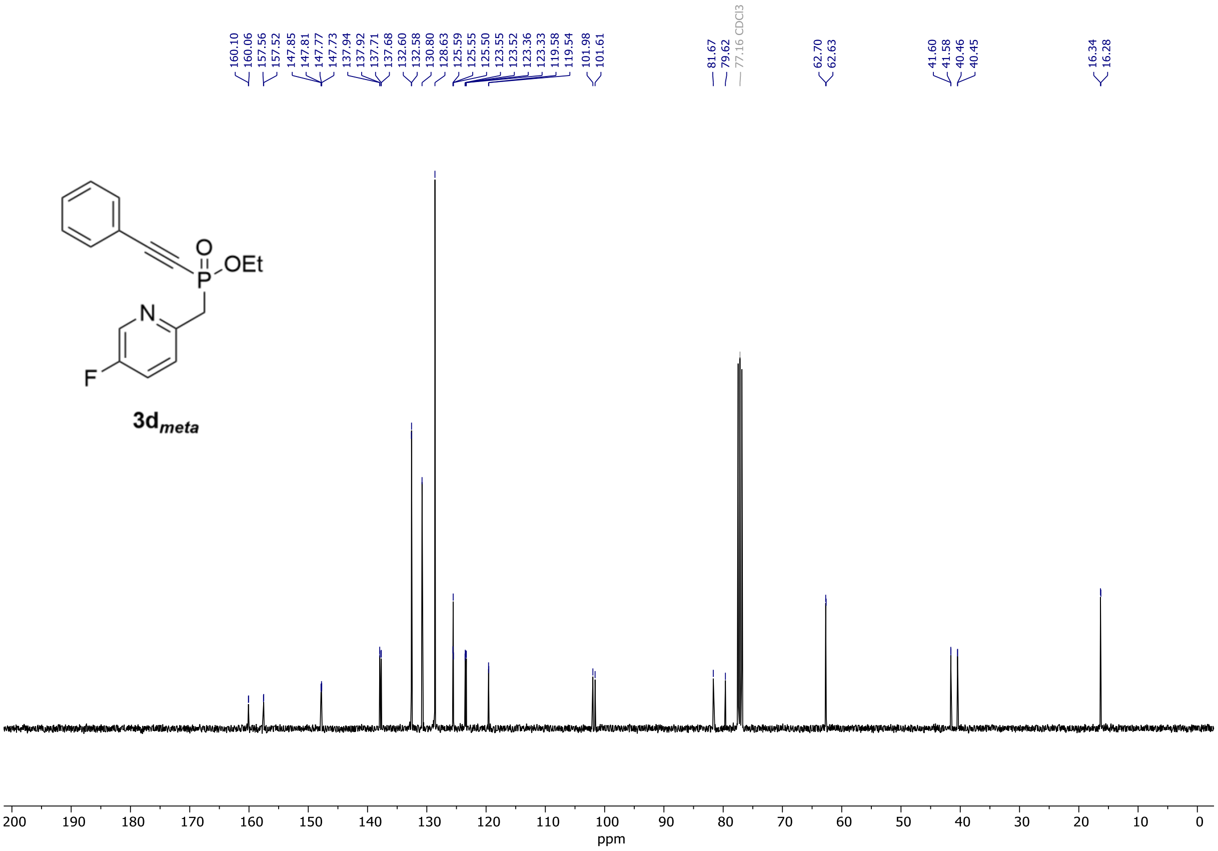

Figure S165 *^{31}P {^{1}H} NMR spectrum of **3d$_{meta}$** (162 MHz, CDCl$_3$).*

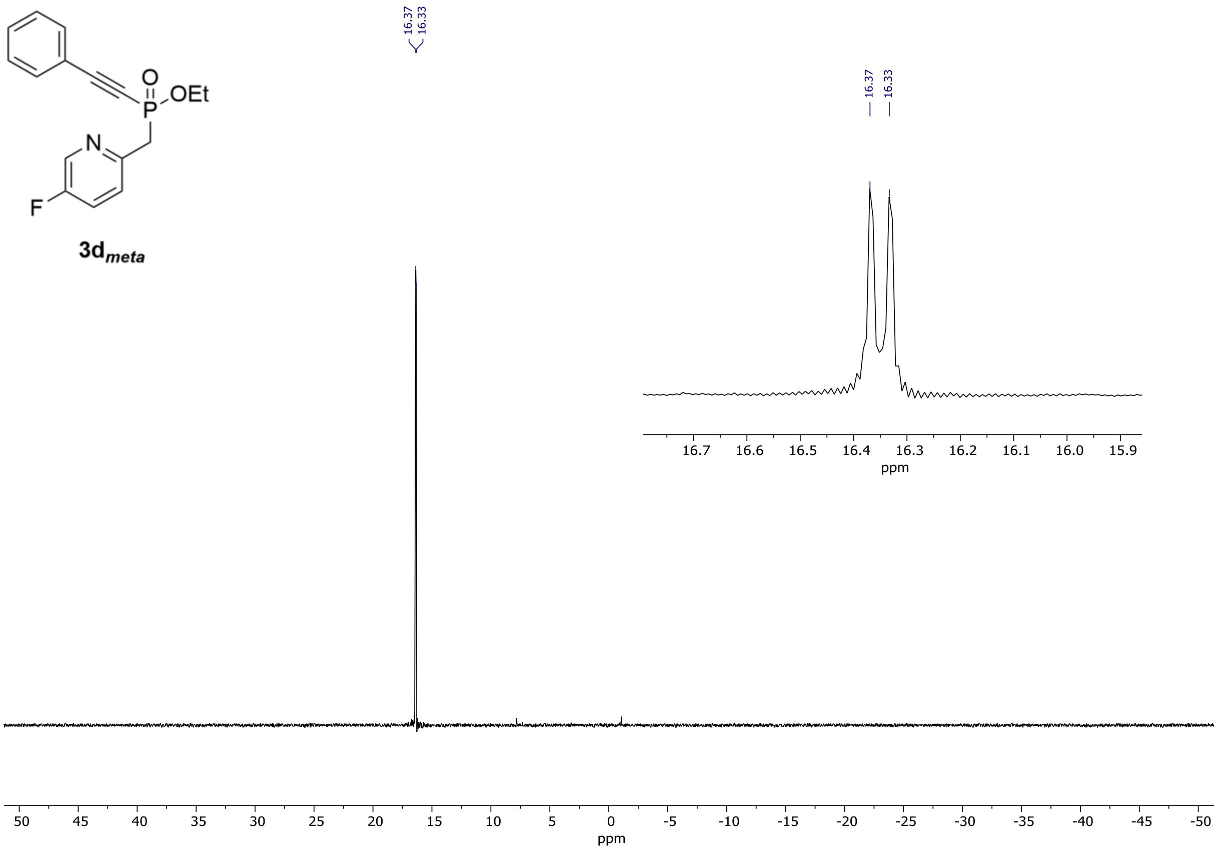

Figure S166 *^{31}P {^{1}H} NMR spectrum of **3d$_{meta}$** (162 MHz, CDCl$_3$).*

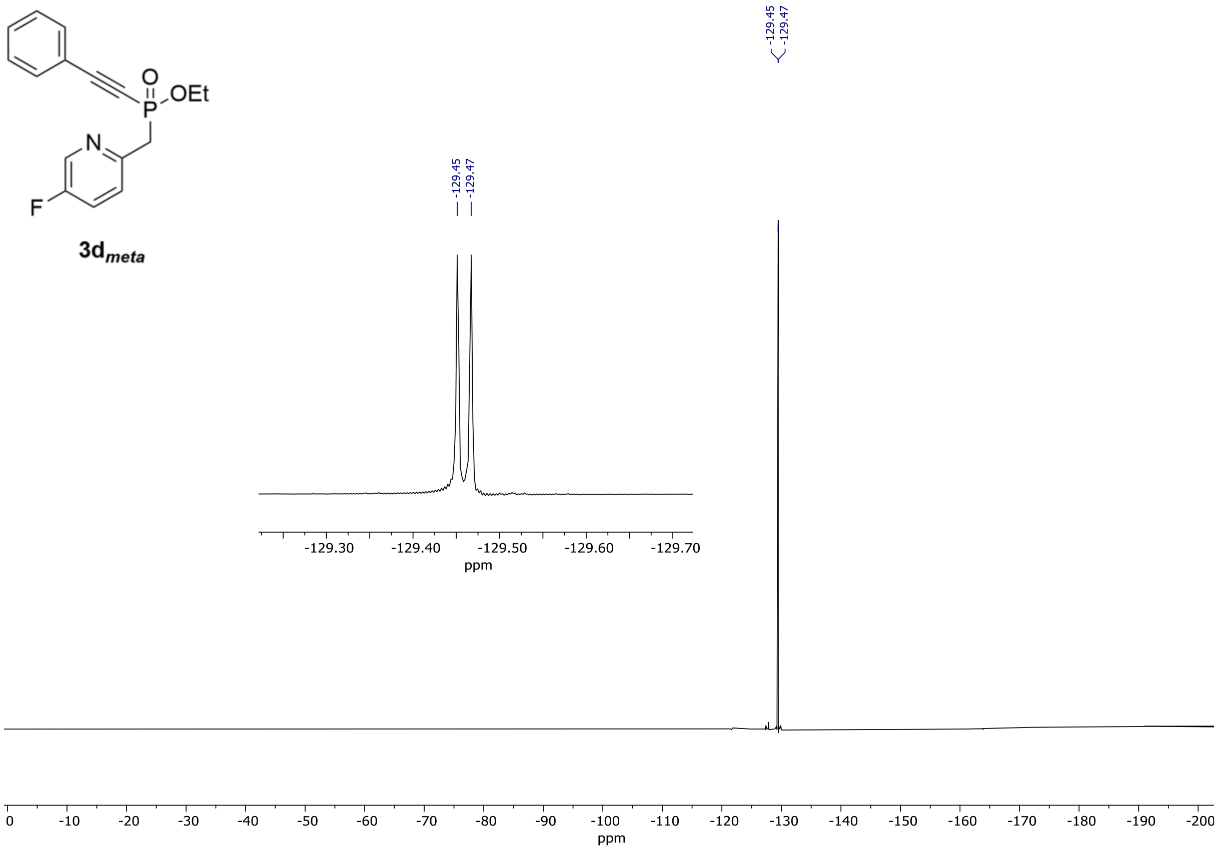

3d$_{meta}$

Figure S167 *^{19}F {^{1}H} NMR spectrum of* **3d$_{meta}$** *(376 MHz, CDCl$_3$).*

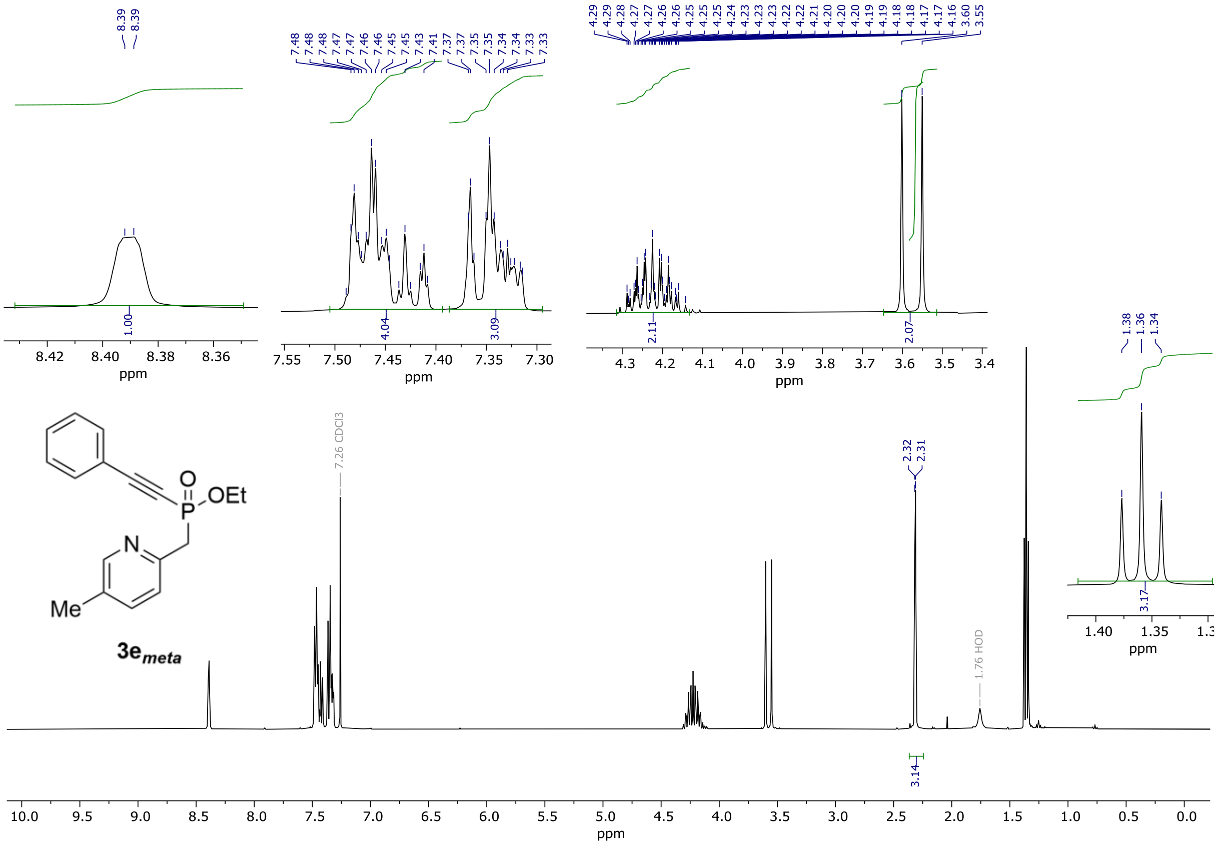

3e$_{meta}$

Figure S168 *^{1}H NMR spectrum of* ***3e$_{meta}$*** *(400 MHz, CDCl$_3$).*

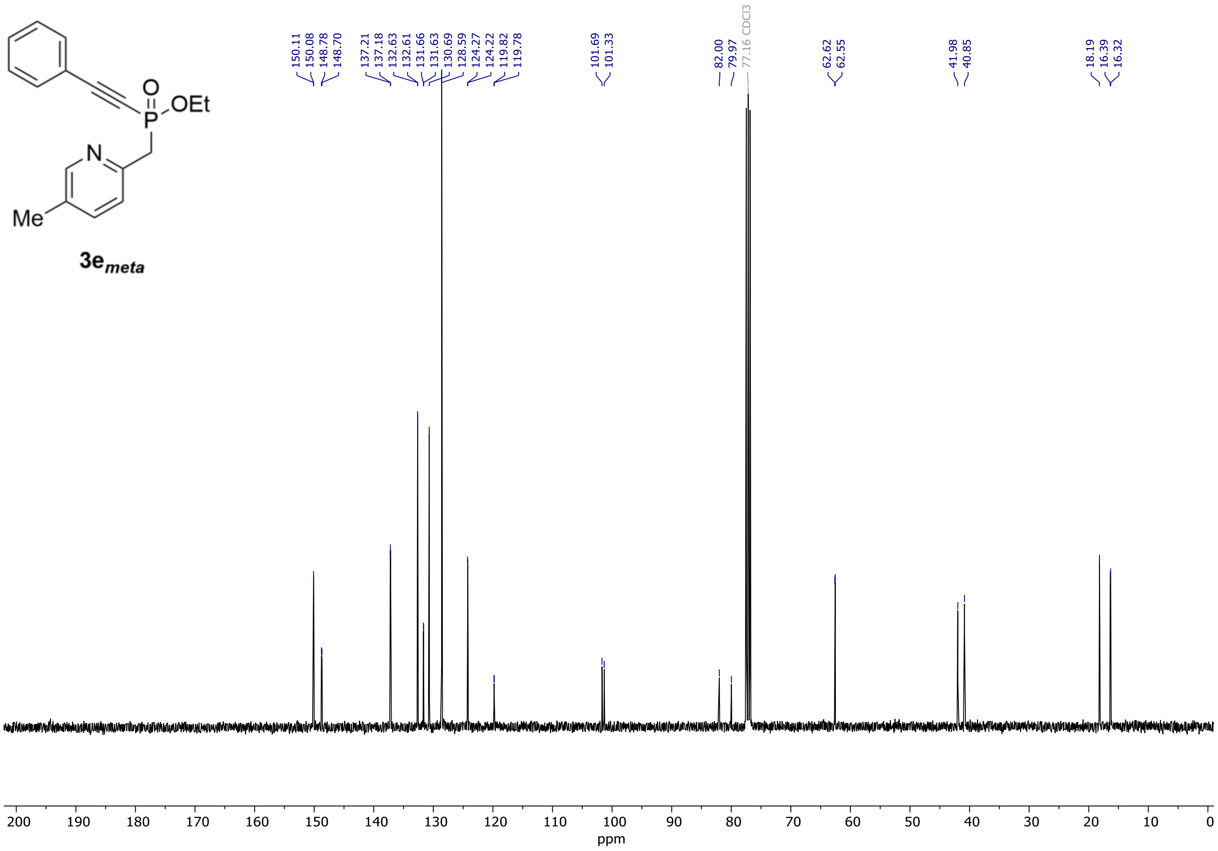

Figure S169 ^{13}C {^{1}H} *NMR spectrum of* ***3e**$_{meta}$* *(101 MHz, CDCl$_3$).*

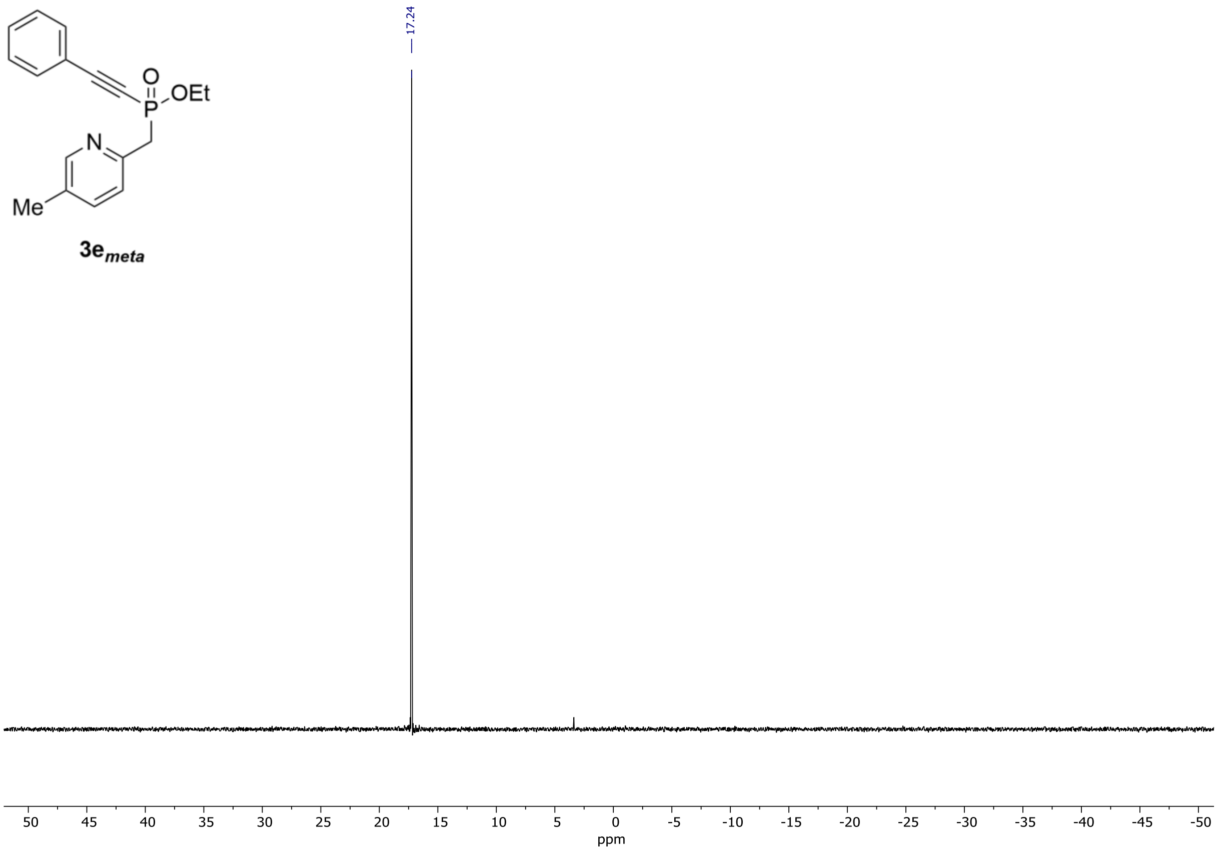

Figure S170 ^{31}P {^{1}H} *NMR spectrum of* ***3e**$_{meta}$* *(162 MHz, CDCl$_3$).*

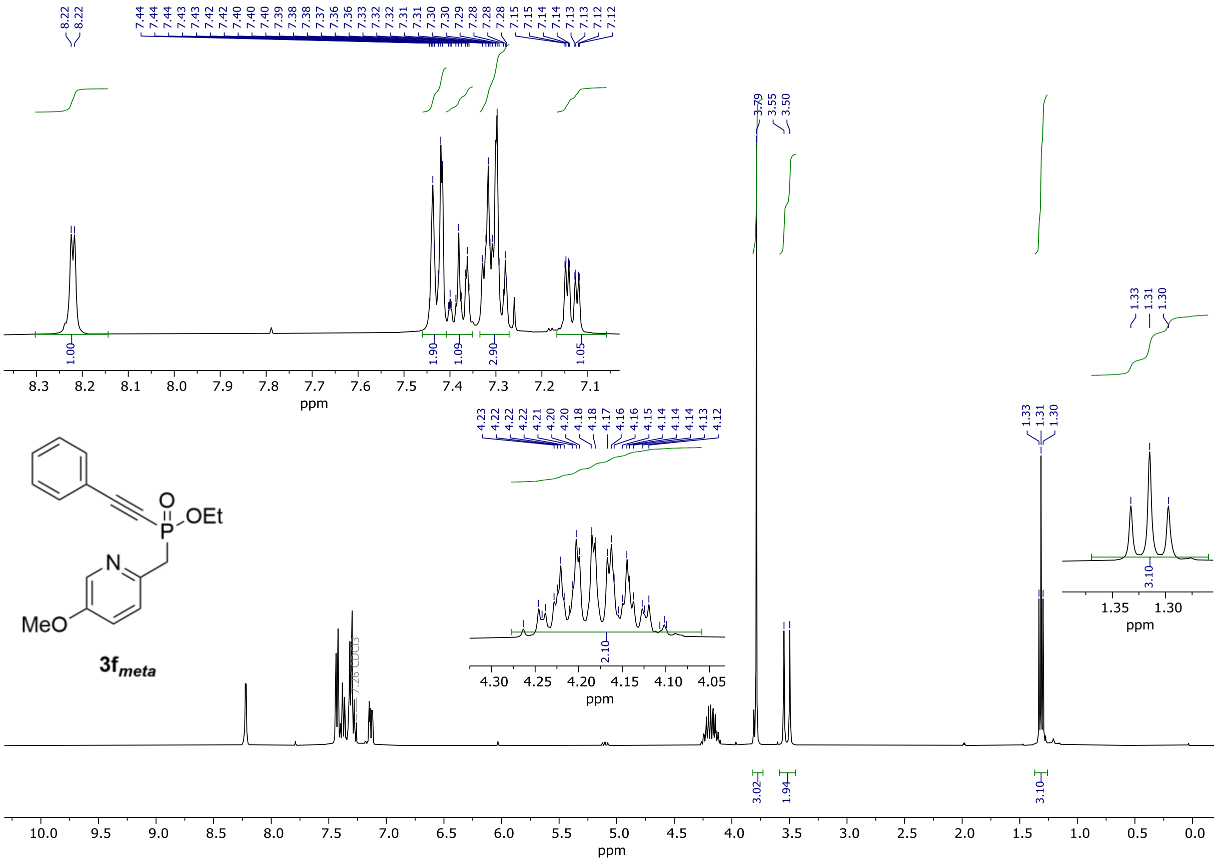

Figure S171 ¹H NMR spectrum of **3f**$_{meta}$ (400 MHz, $CDCl_3$).

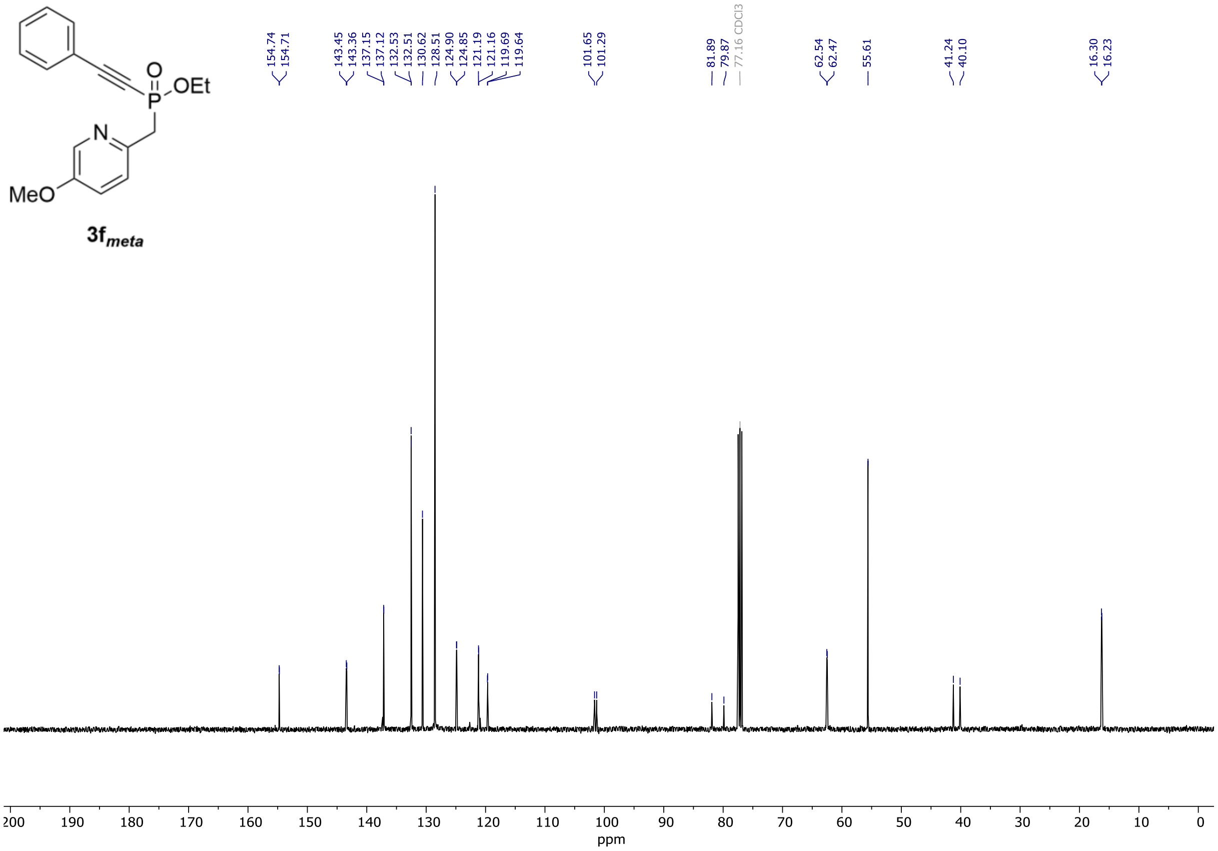

Figure S172 ¹³C {¹H} NMR spectrum of **3f**$_{meta}$ (101 MHz, $CDCl_3$).

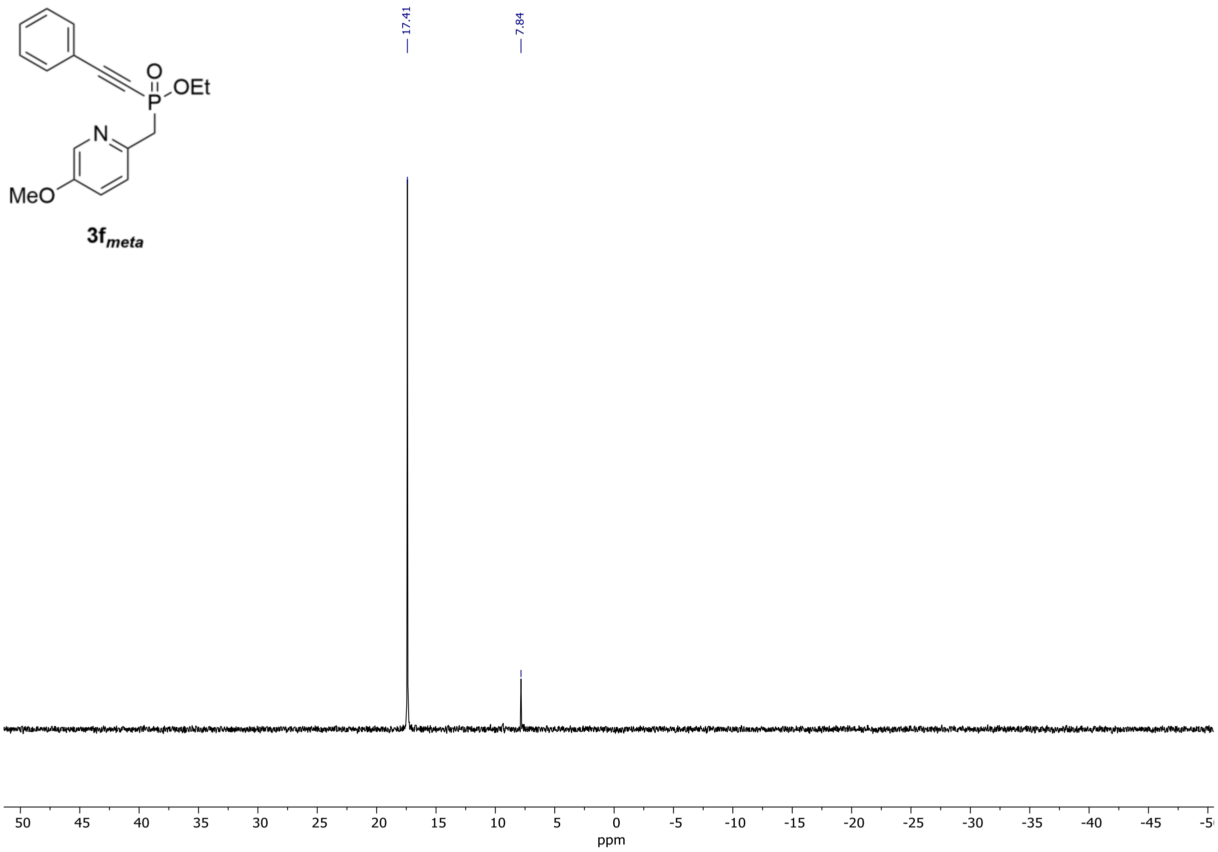

Figure S173 $^{31}P\ \{^1H\}$ *NMR spectrum of **3f**$_{meta}$ (162 MHz, $CDCl_3$).*

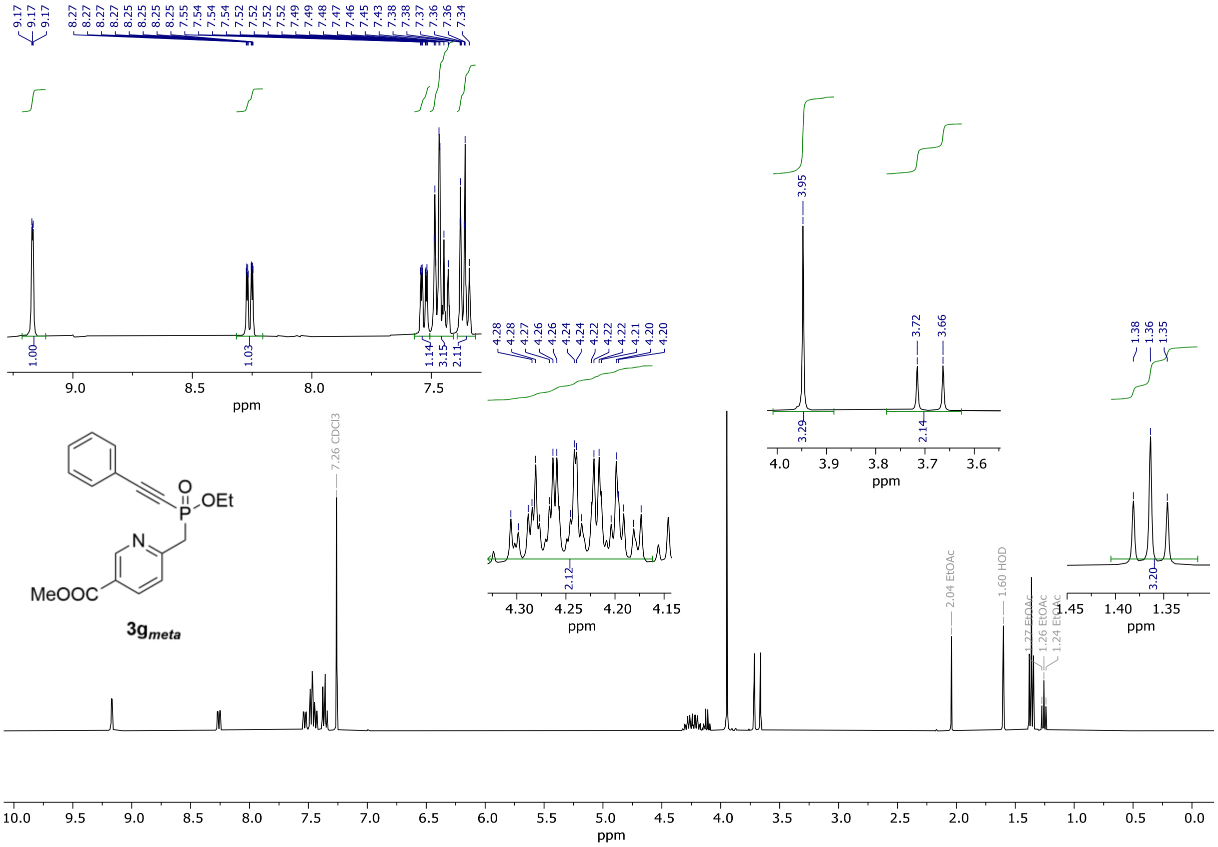

Figure S174 ^1H *NMR spectrum of **3g**$_{meta}$ (400 MHz, $CDCl_3$).*

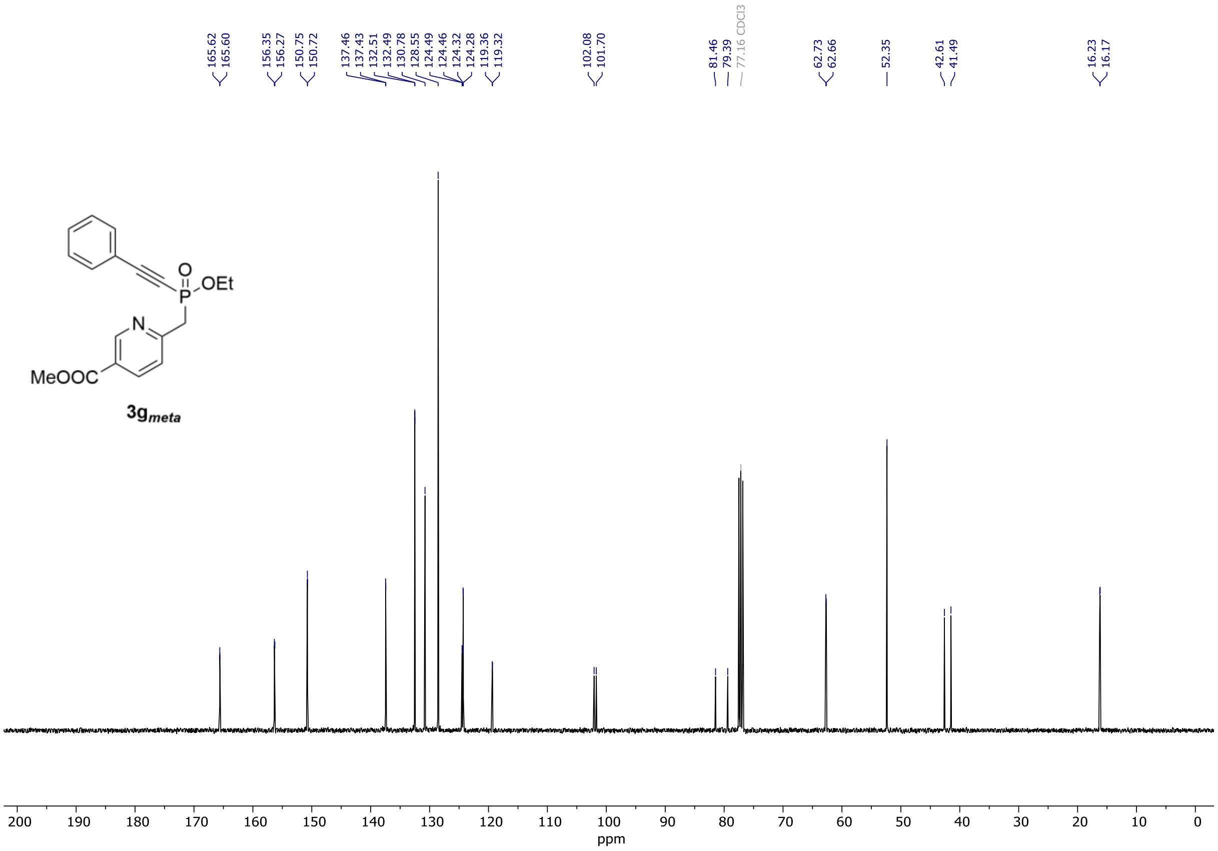

Figure S175 *^{13}C {^{1}H} NMR spectrum of **3g$_{meta}$** (101 MHz, $CDCl_3$).*

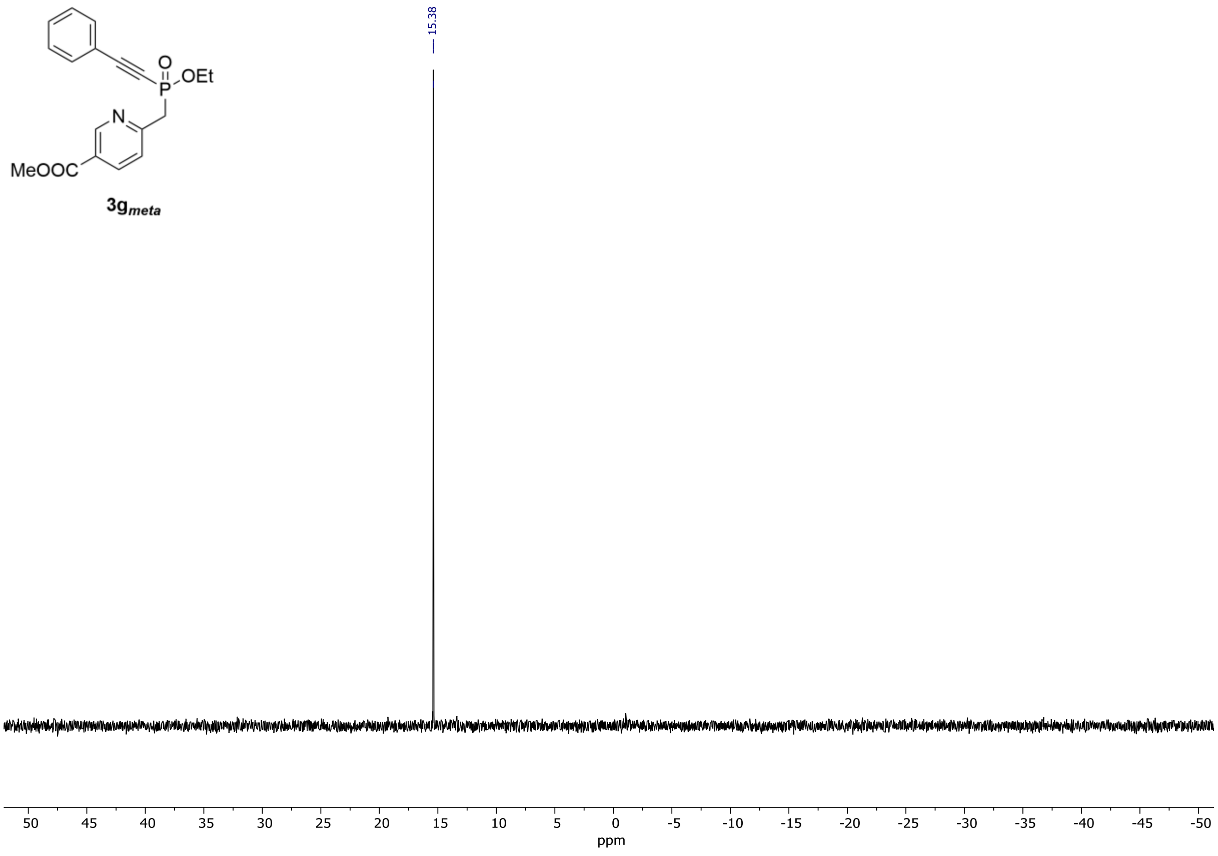

Figure S176 *^{31}P {^{1}H} NMR spectrum of **3g$_{meta}$** (162 MHz, $CDCl_3$).*

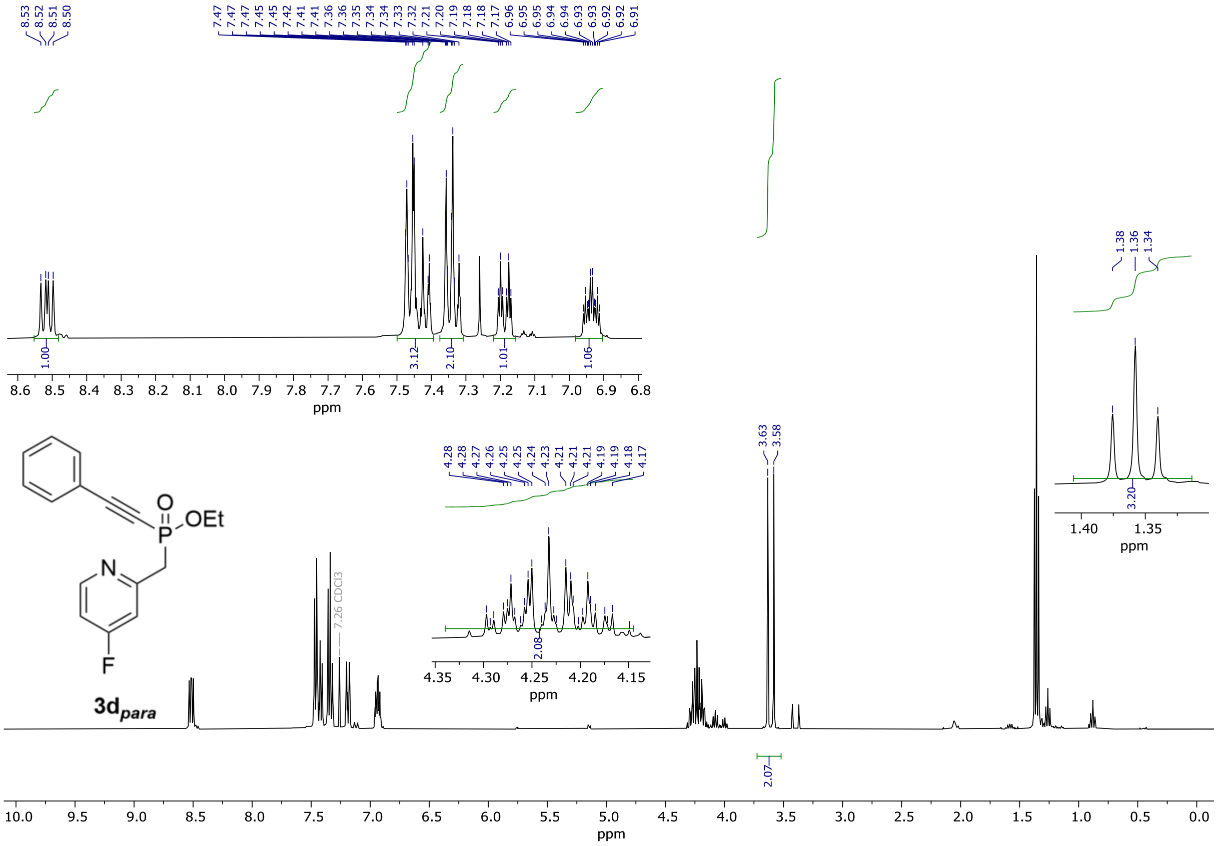

Figure S177 ^{1}H *NMR spectrum of* ***3d$_{para}$*** *(400 MHz, $CDCl_3$).*

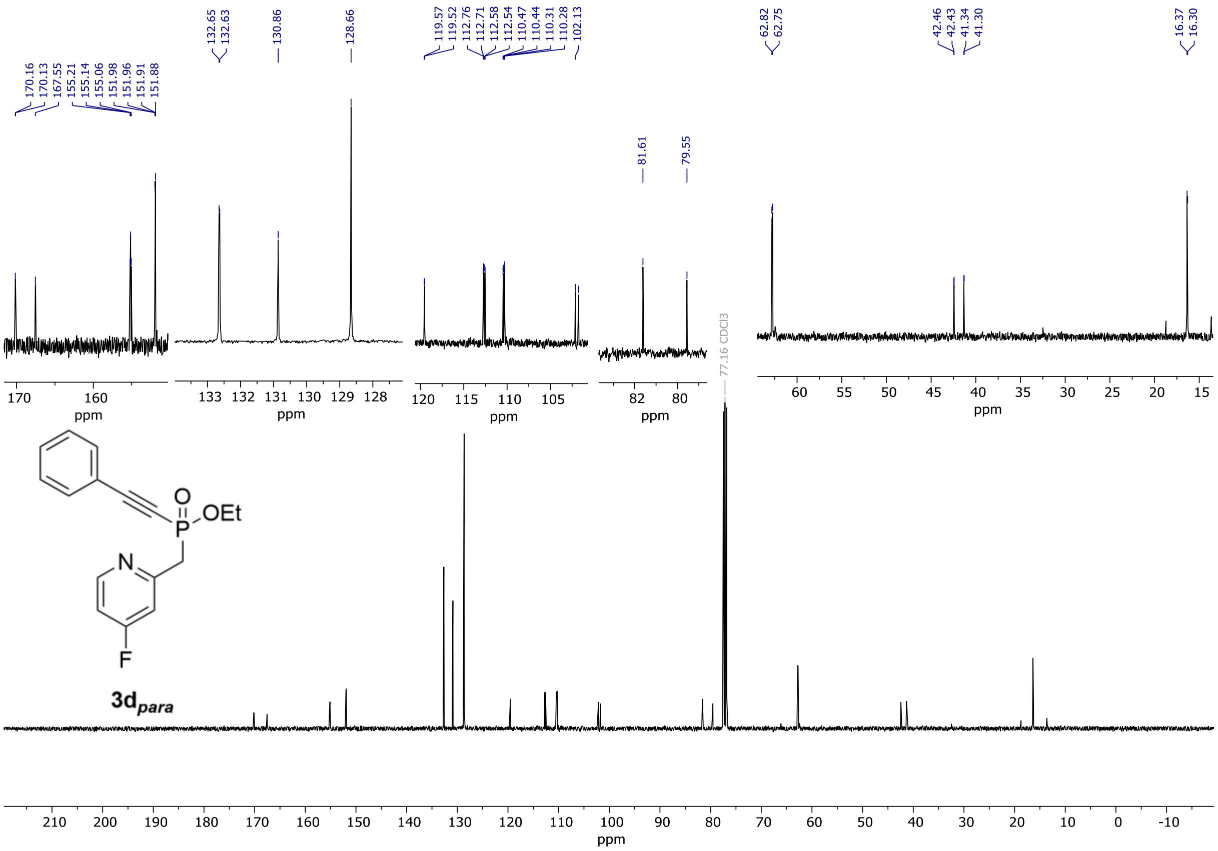

Figure S178 ^{13}C *{^{1}H} NMR spectrum of* ***3d$_{para}$*** *(101 MHz, $CDCl_3$).*

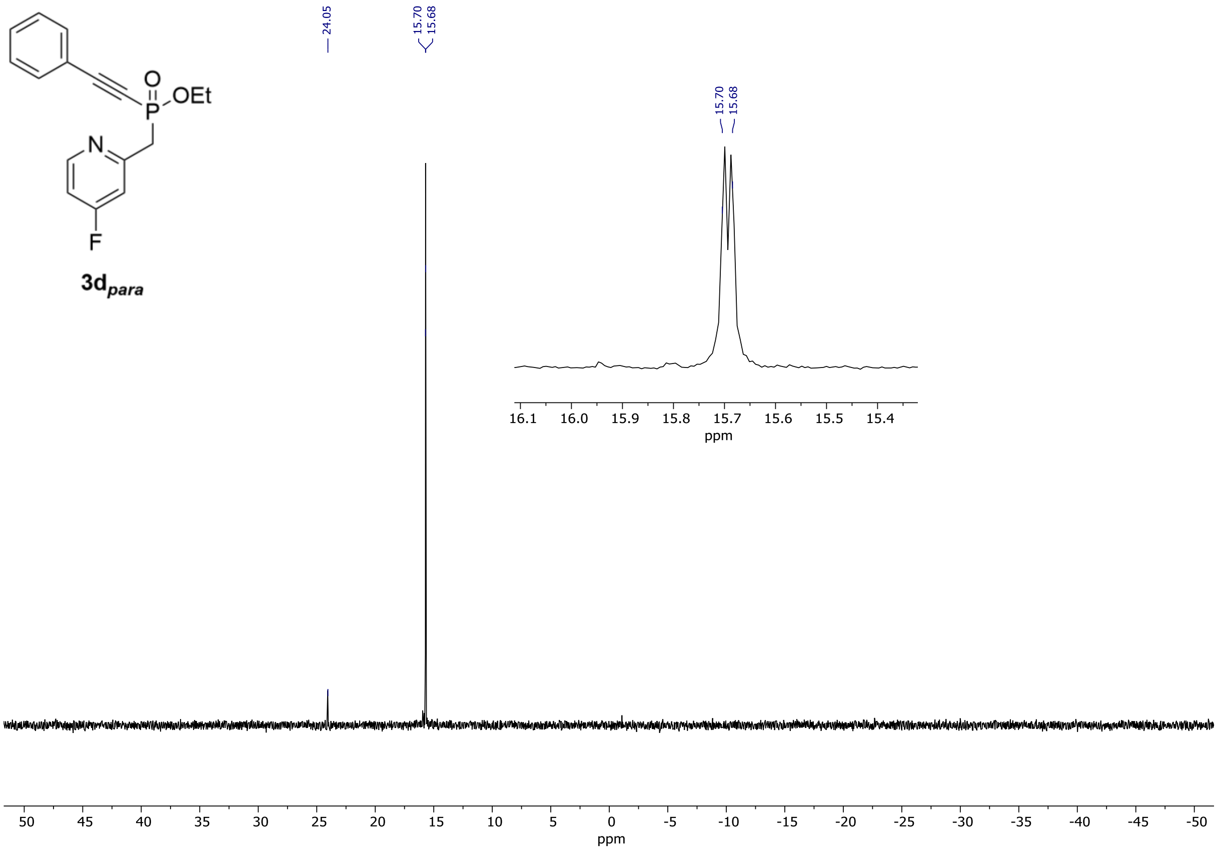

Figure S179 $^{31}P\ \{^{1}H\}$ *NMR spectrum of* ***3d****$_{para}$ (162 MHz, $CDCl_3$).*

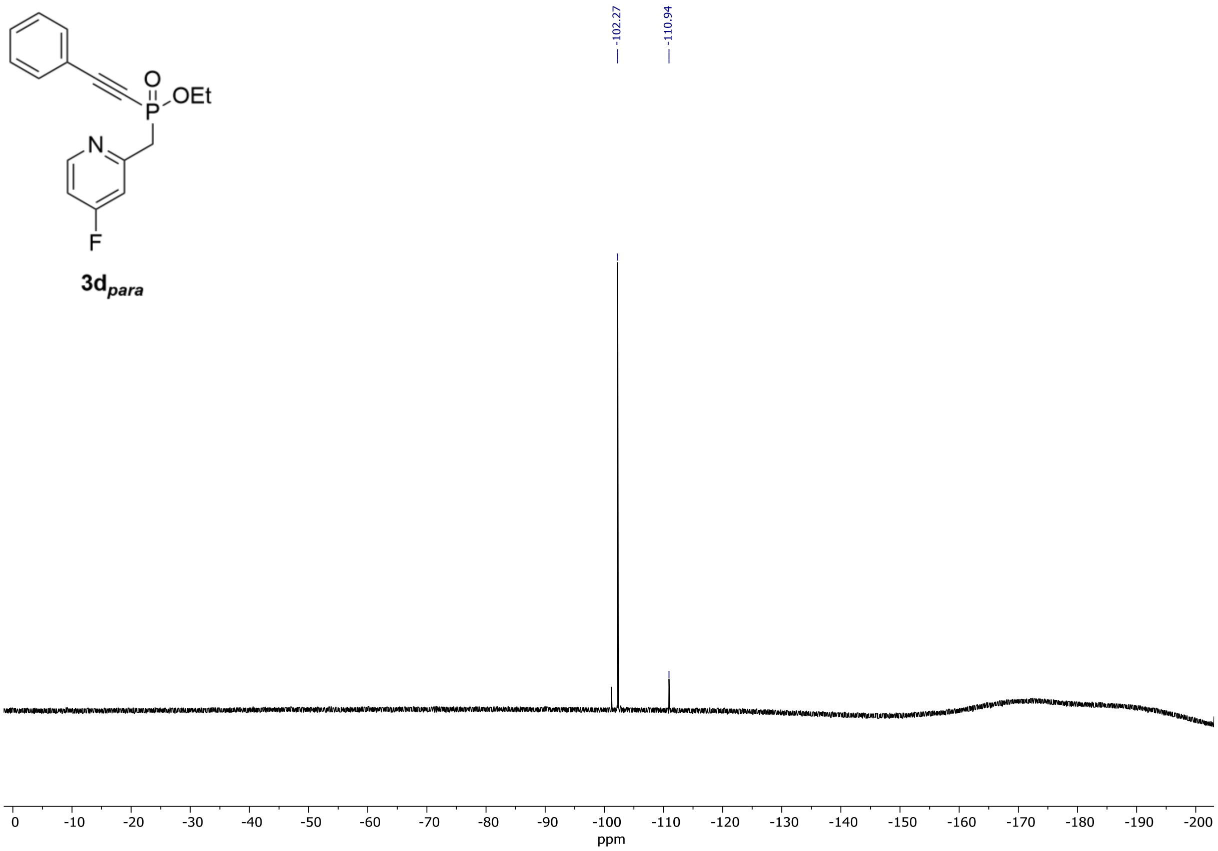

Figure S180 $^{19}F\ \{^{1}H\}$ *NMR spectrum of* ***3d****$_{para}$ (376 MHz, $CDCl_3$).*

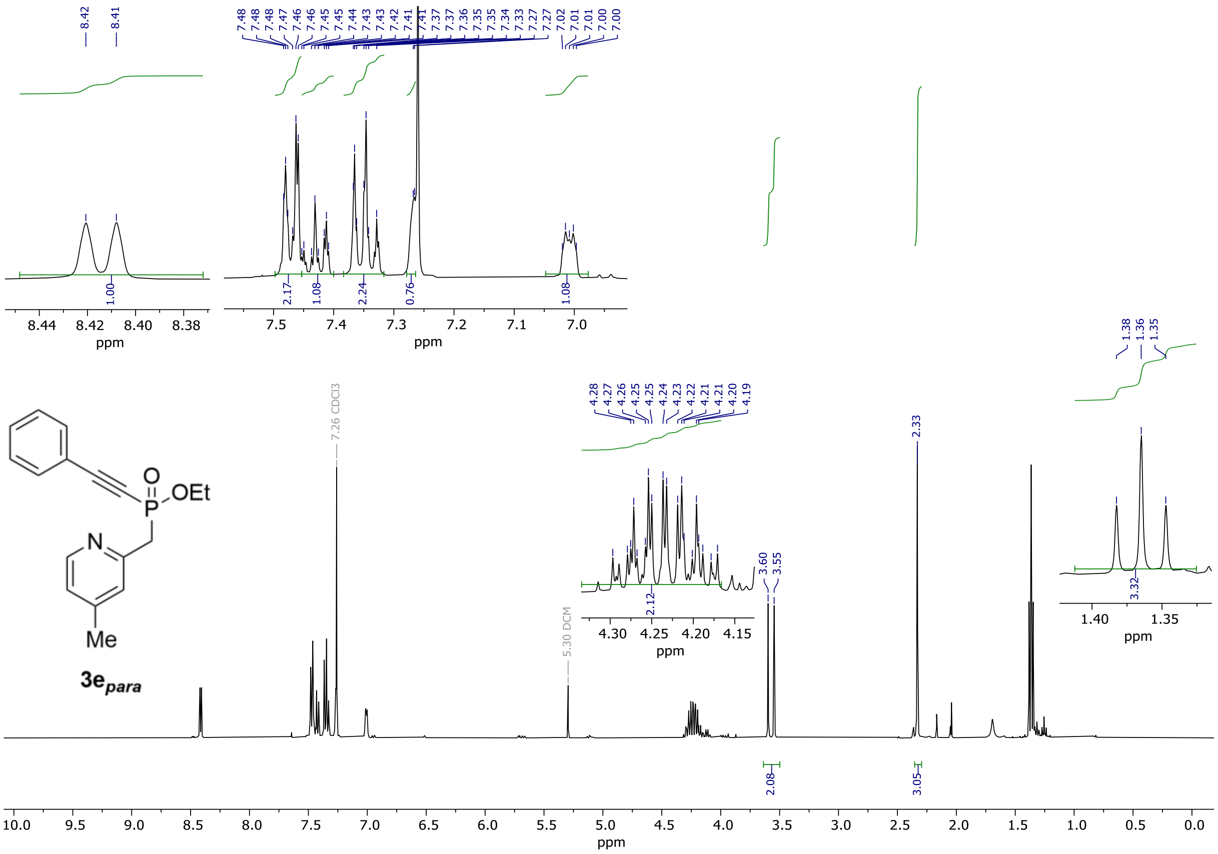

Figure S181 ^{1}H *NMR spectrum of* ***3e$_{para}$*** *(400 MHz, $CDCl_3$).*

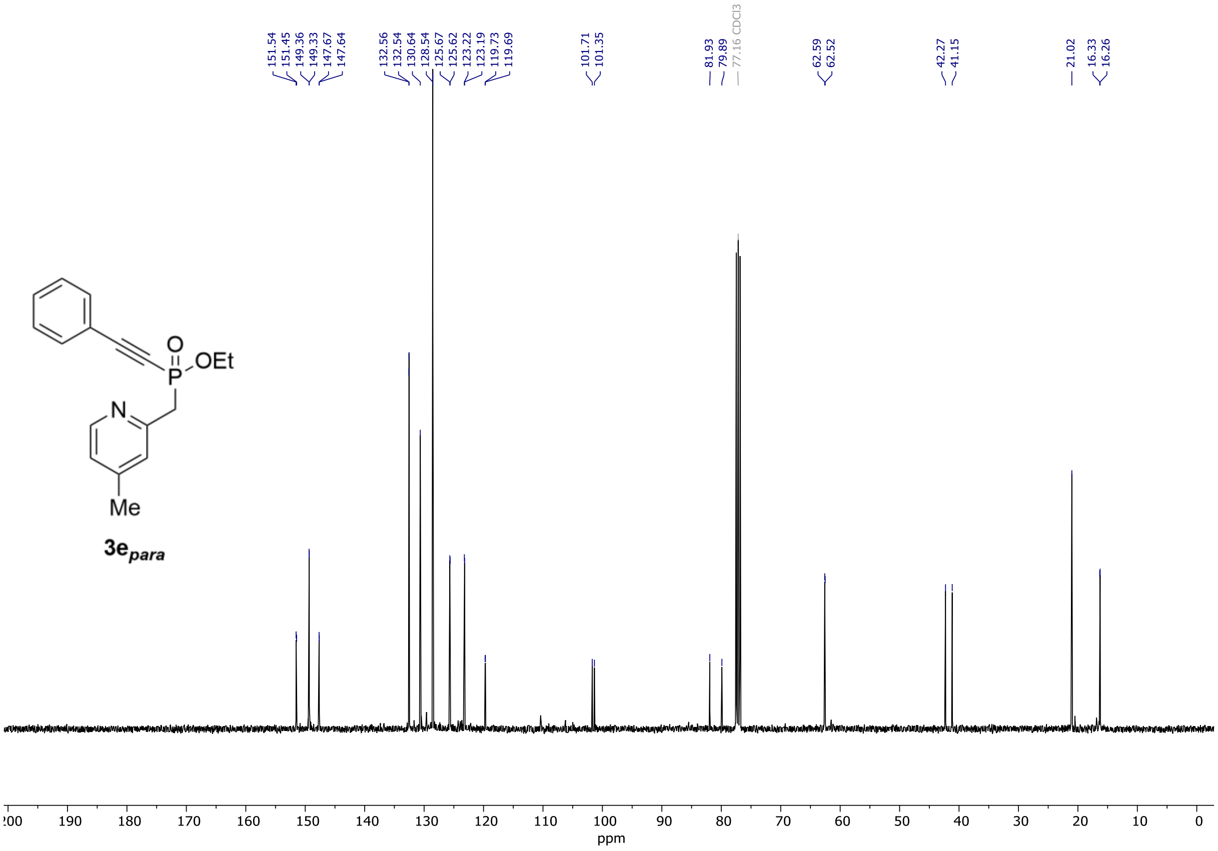

Figure S182 ^{13}C *{^{1}H} NMR spectrum of* ***3e$_{para}$*** *(101 MHz, $CDCl_3$).*

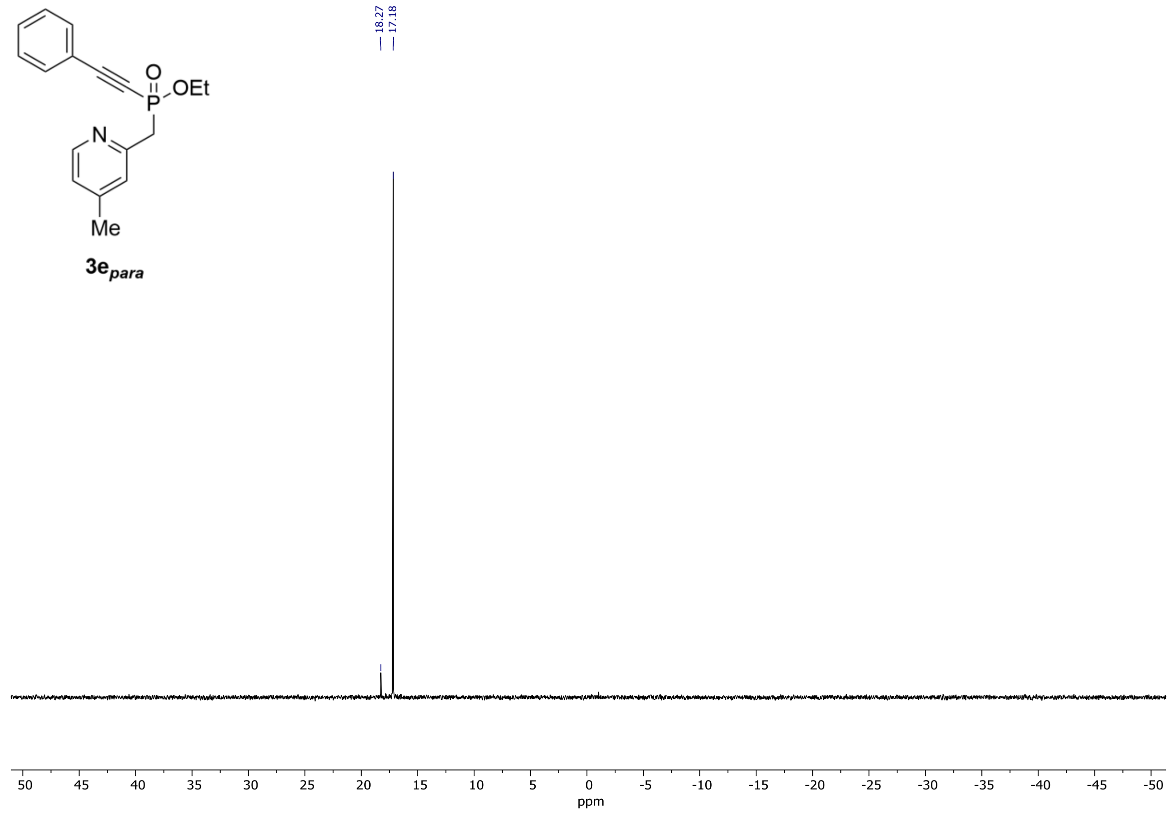

Figure S183 ^{31}P {^{1}H} NMR spectrum of ***3e$_{para}$*** (162 MHz, $CDCl_3$).

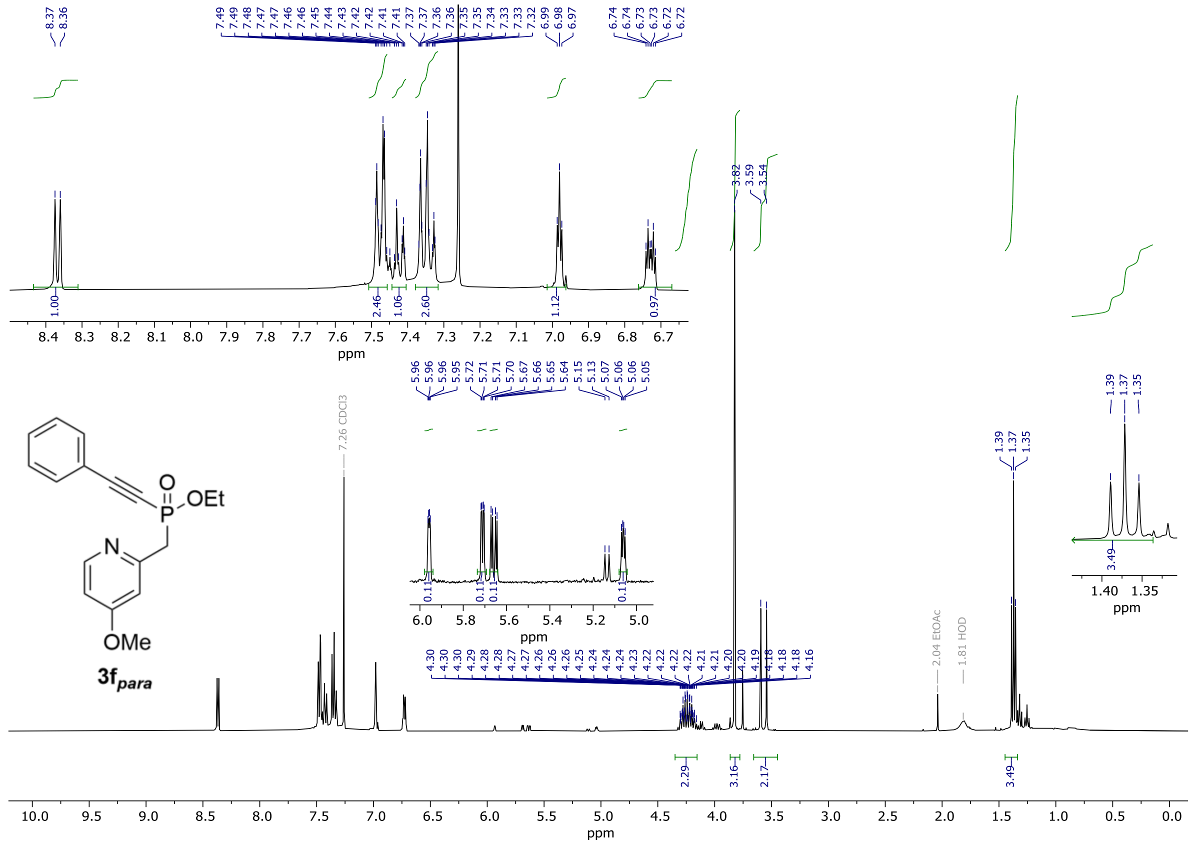

Figure S184 ^{1}H NMR spectrum of ***3f$_{para}$*** (400 MHz, $CDCl_3$). The signals at 5-6 ppm are attributed to product ***3f$_{para}$***, formed via spontaneous cyclization.

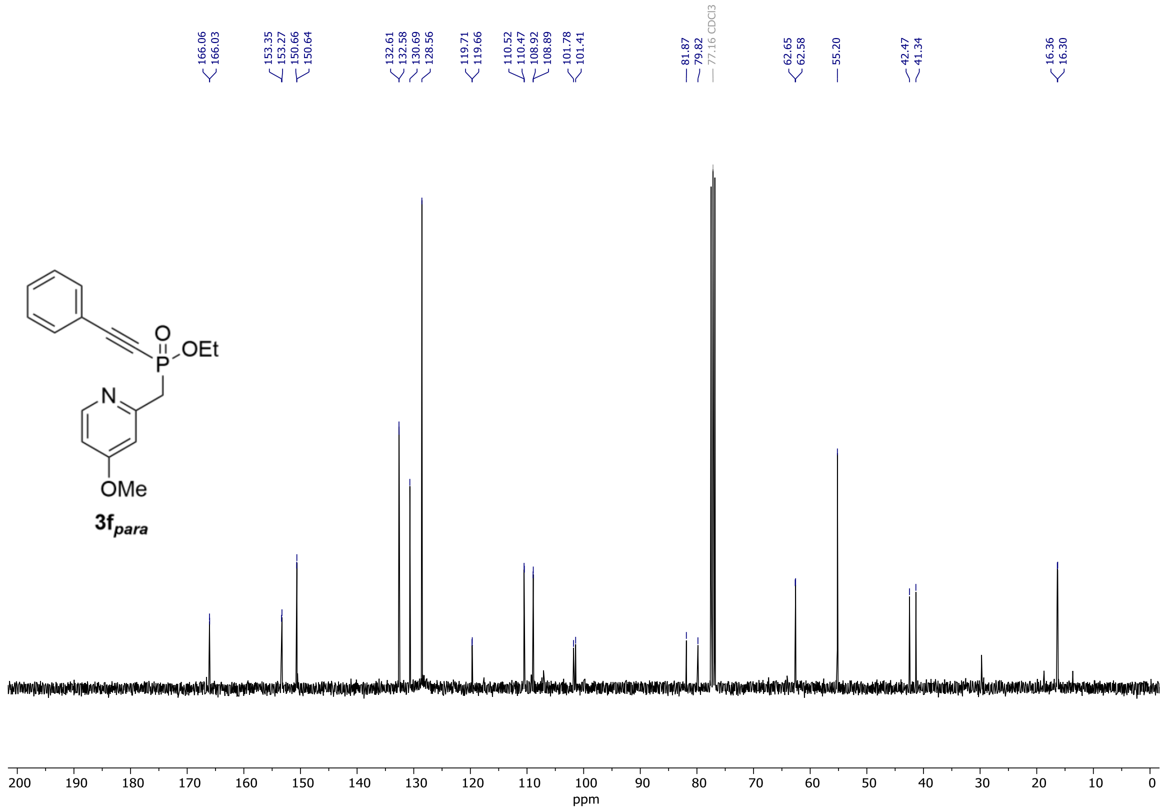

Figure S185 ^{13}C {^{1}H} *NMR spectrum of* ***$3f_{para}$*** *(101 MHz, $CDCl_3$).*

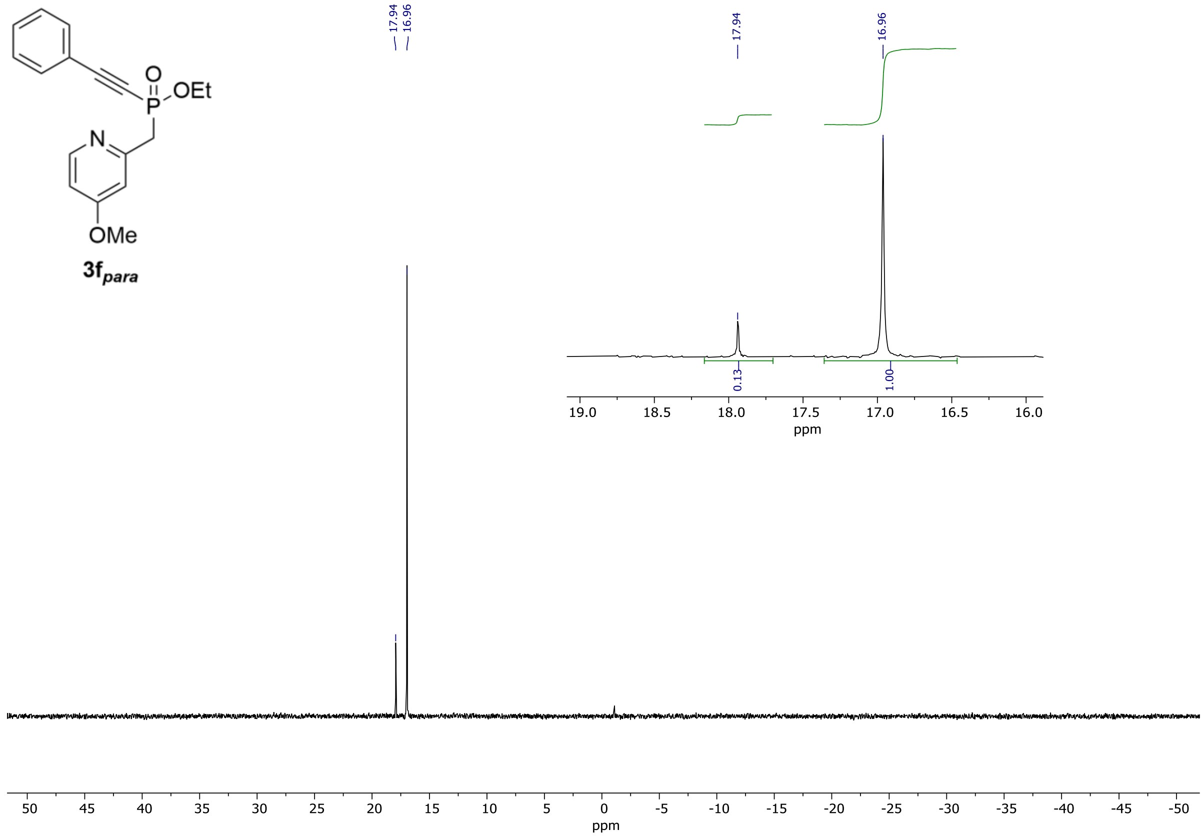

Figure S186 ^{31}P {^{1}H} *NMR spectrum of* ***$3f_{para}$*** *(162 MHz, $CDCl_3$). The signal at 17.94 ppm is attributed to product* ***$4f_{para}$****, formed via spontaneous cyclization.*

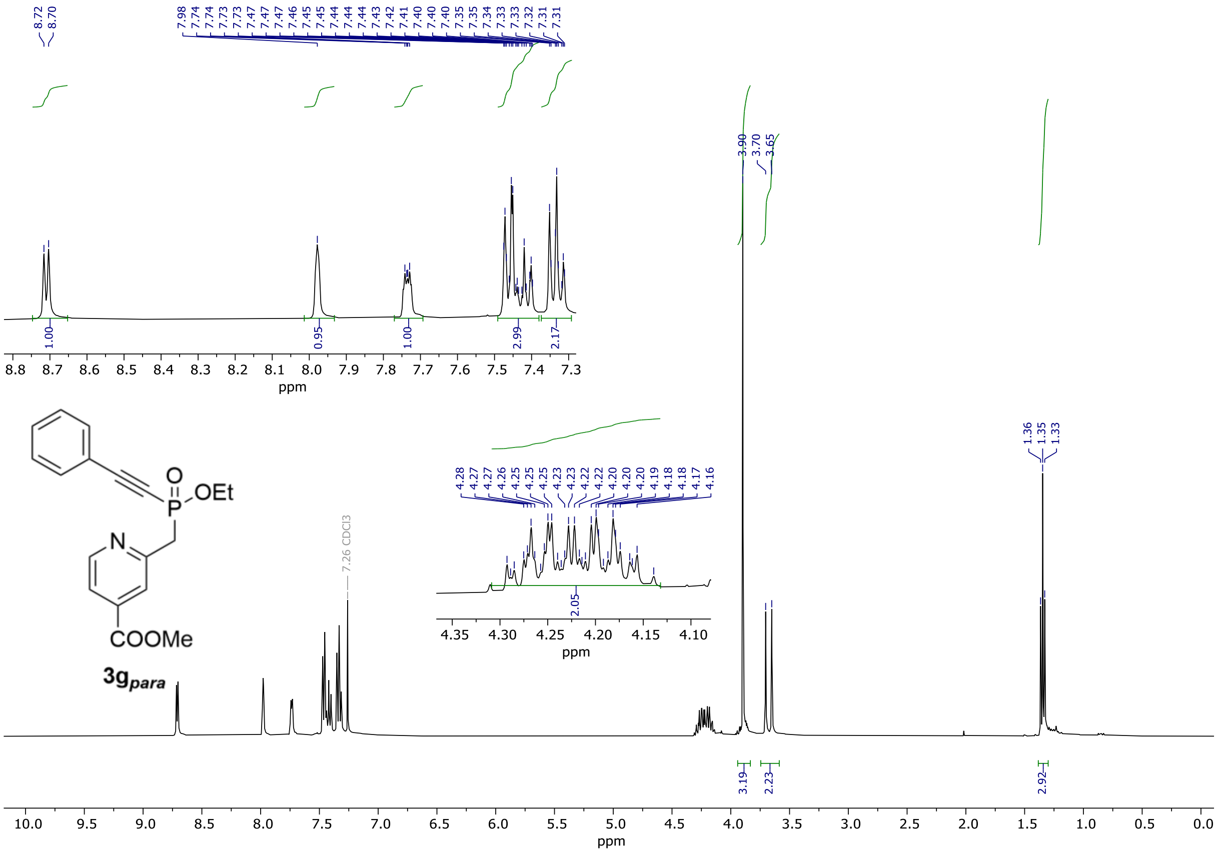

Figure S187 *^{1}H NMR spectrum of **3g**$_{para}$ (400 MHz, $CDCl_3$).*

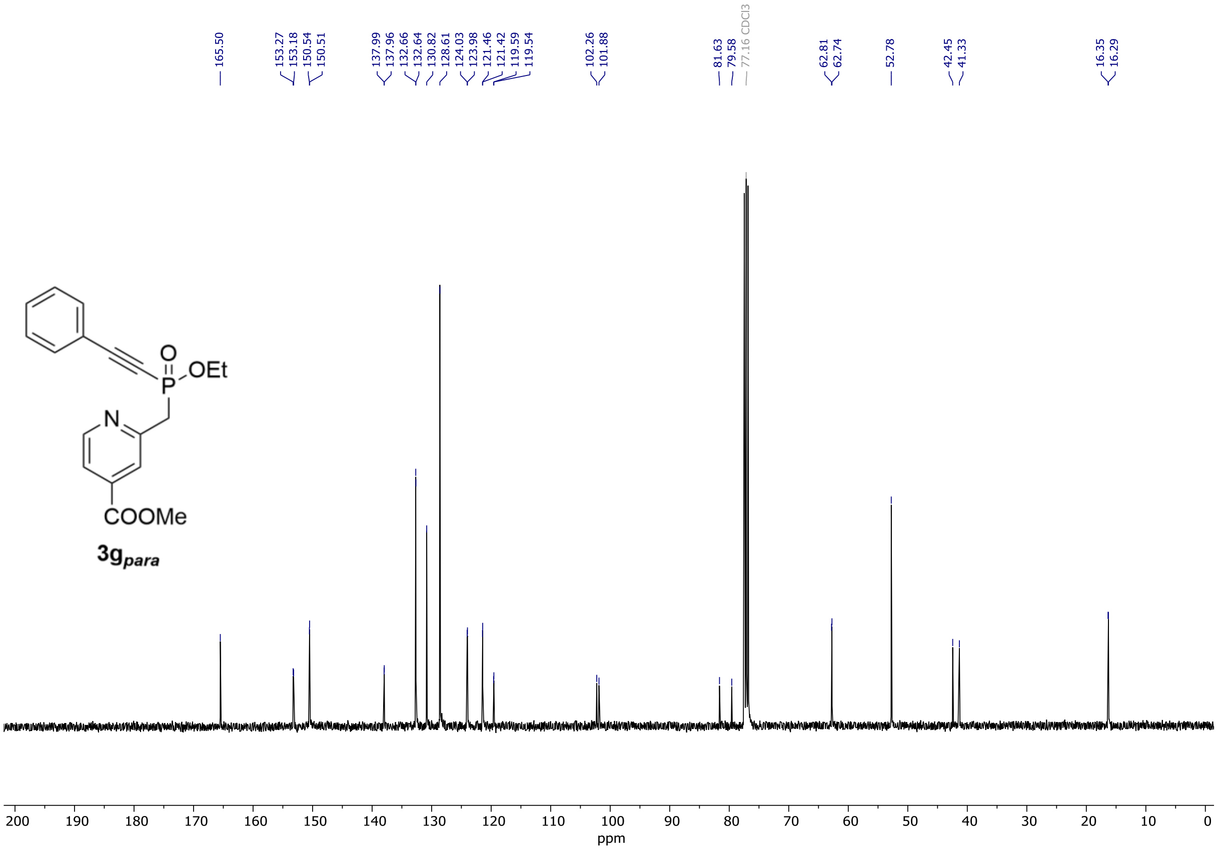

Figure S188 *^{13}C {^{1}H} NMR spectrum of **3g**$_{para}$ (101 MHz, $CDCl_3$).*

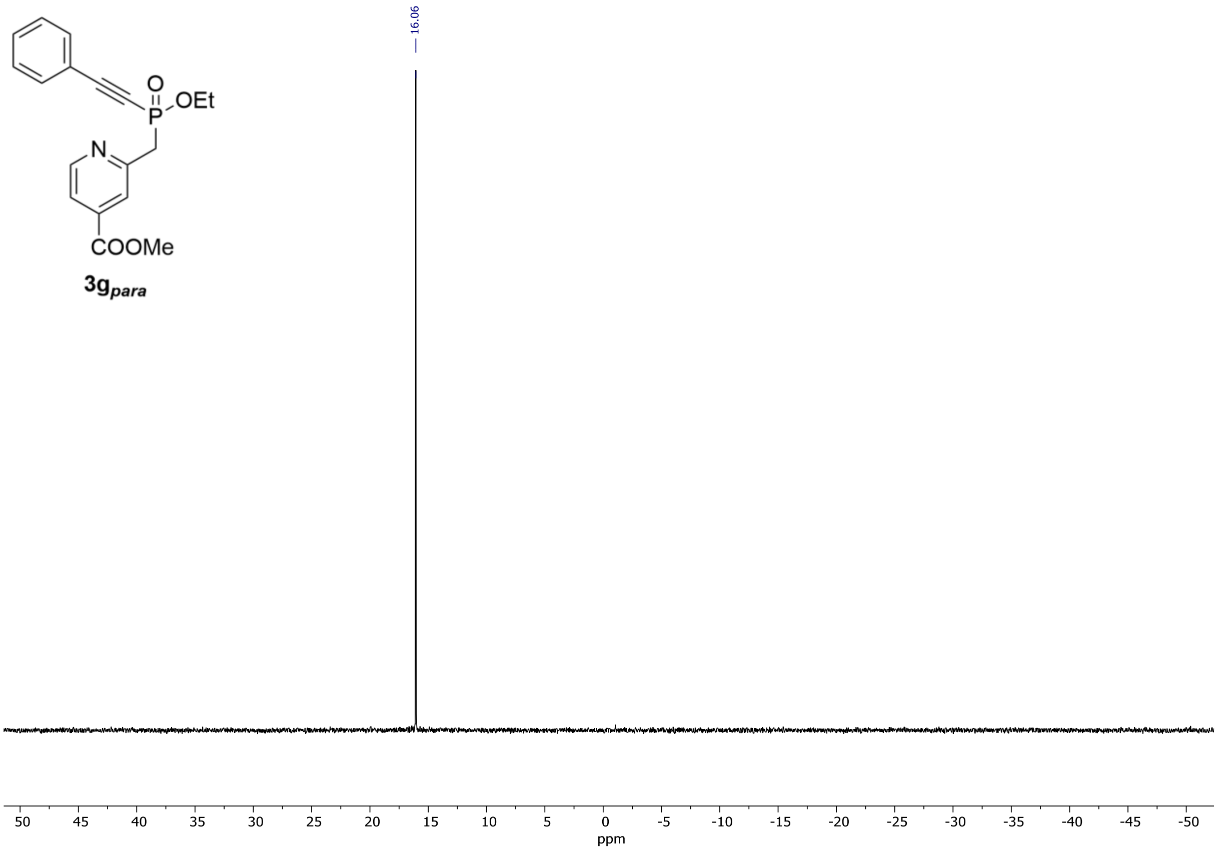

Figure S189 *^{31}P {^{1}H} NMR spectrum of **3g$_{para}$** (162 MHz, CDCl$_3$).*

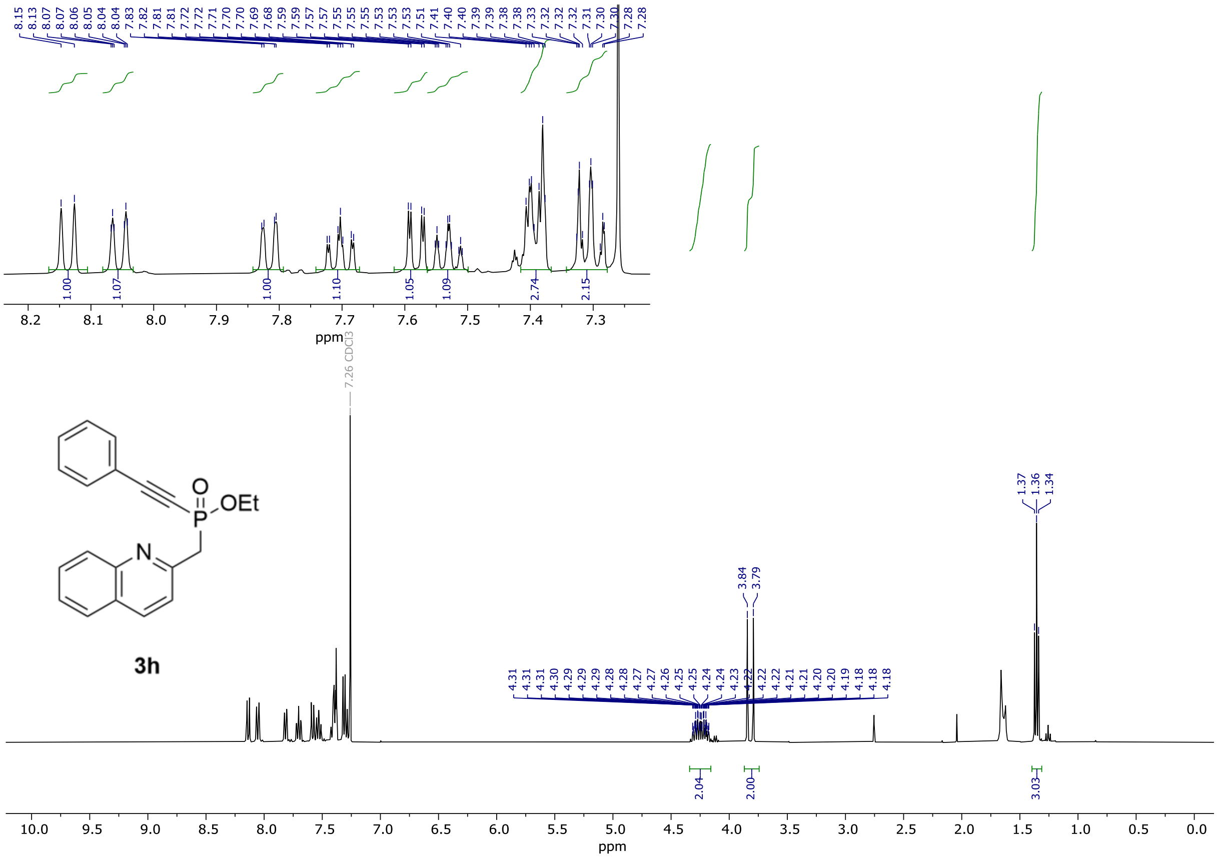

Figure S190 *^{1}H NMR spectrum of **3h** (400 MHz, CDCl$_3$).*

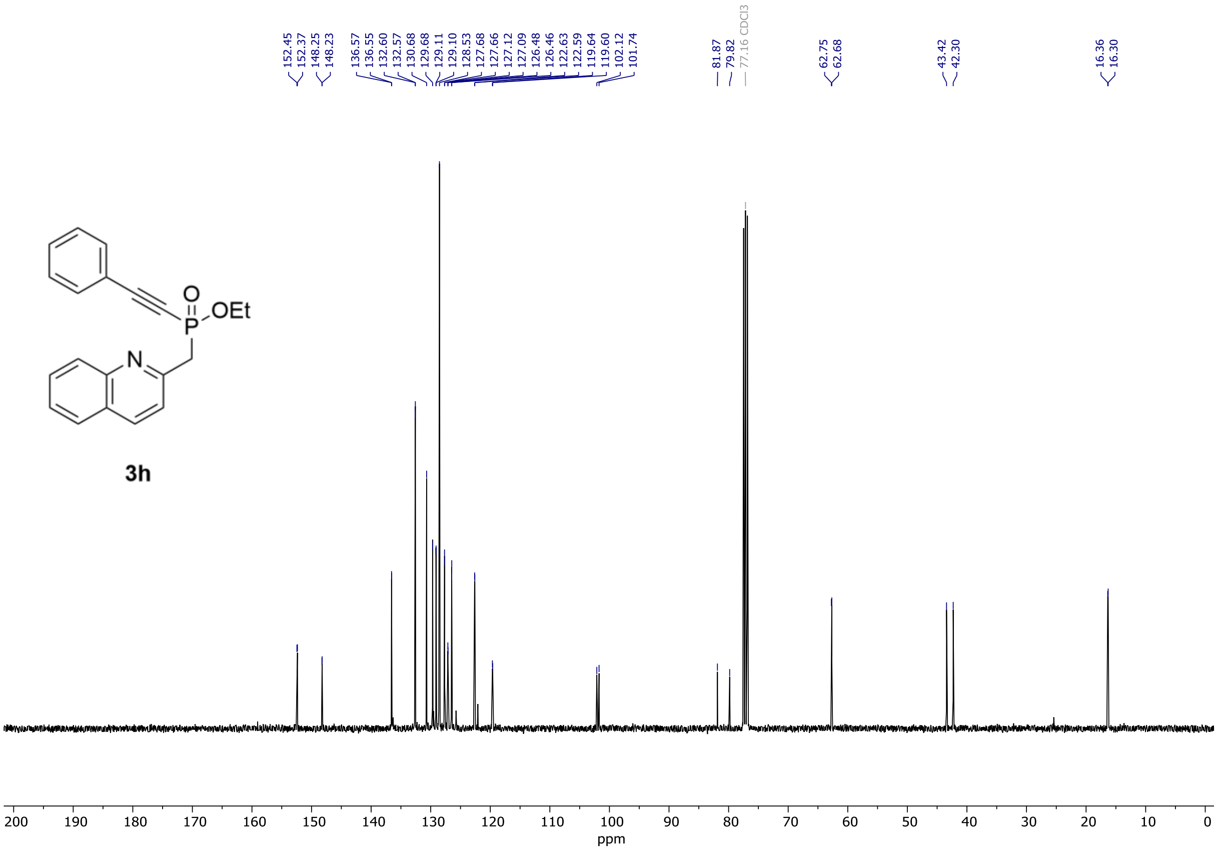

3h

Figure S191 ^{13}C {^{1}H} NMR spectrum of **3h** (101 MHz, $CDCl_3$).

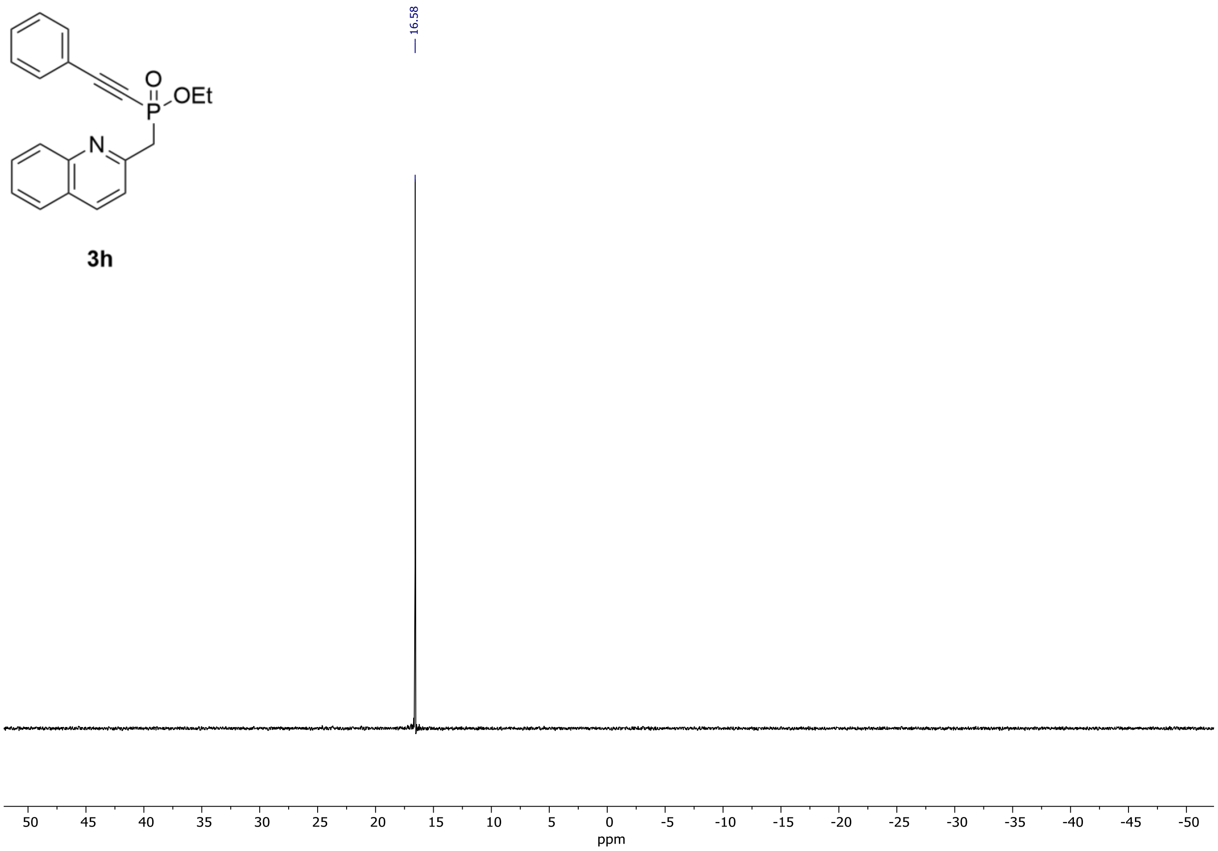

3h

Figure S192 ^{31}P {^{1}H} NMR spectrum of **3h** (162 MHz, $CDCl_3$).

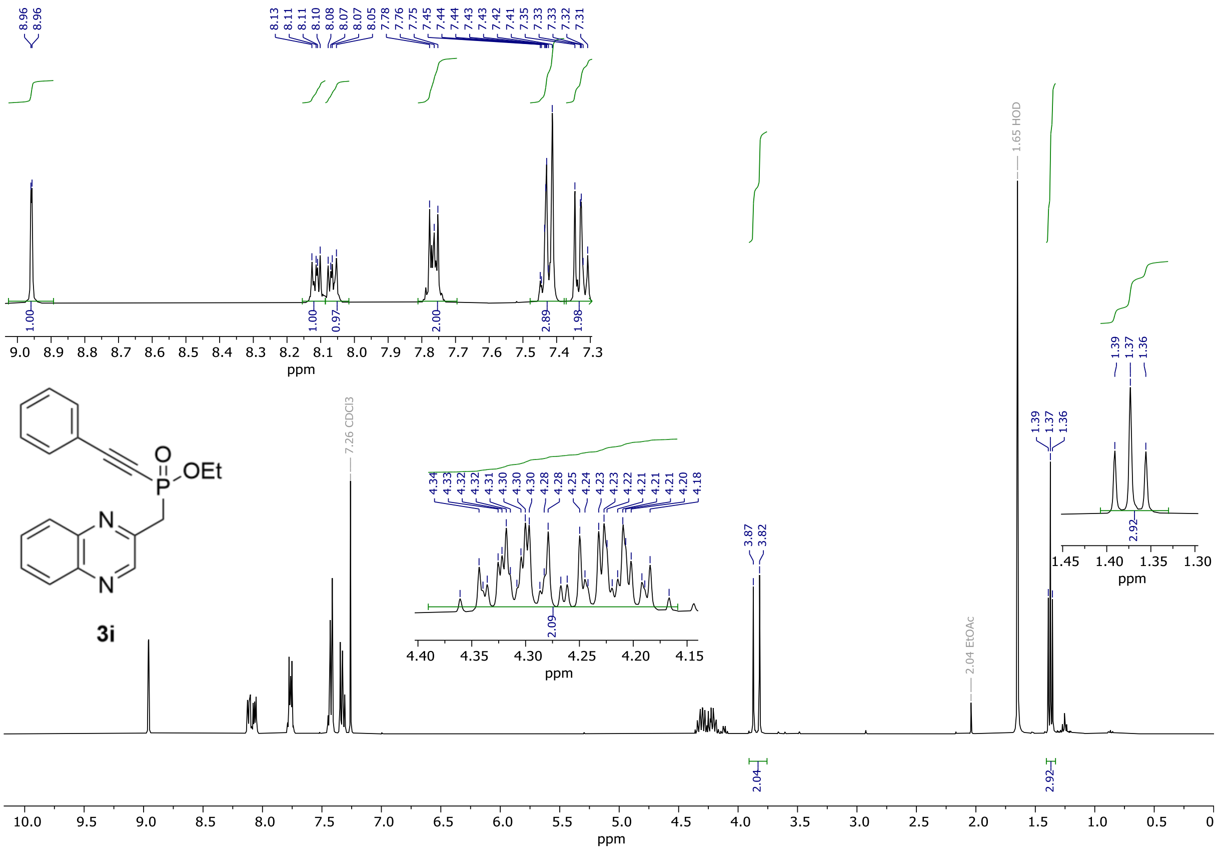

Figure S193 ^{1}H *NMR spectrum of* ***3i*** *(400 MHz, $CDCl_3$).*

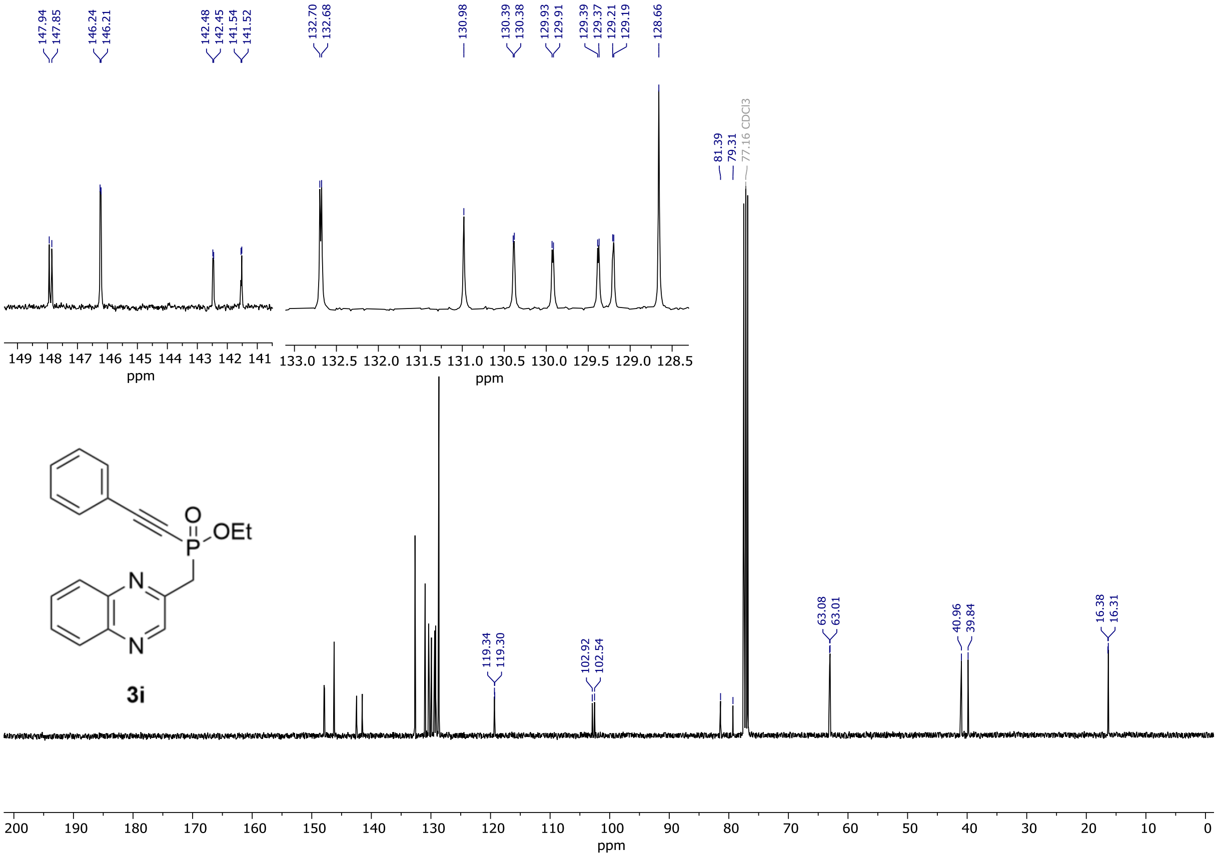

Figure S194 *^{13}C {^{1}H} NMR spectrum of* ***3i*** *(101 MHz, $CDCl_3$).*

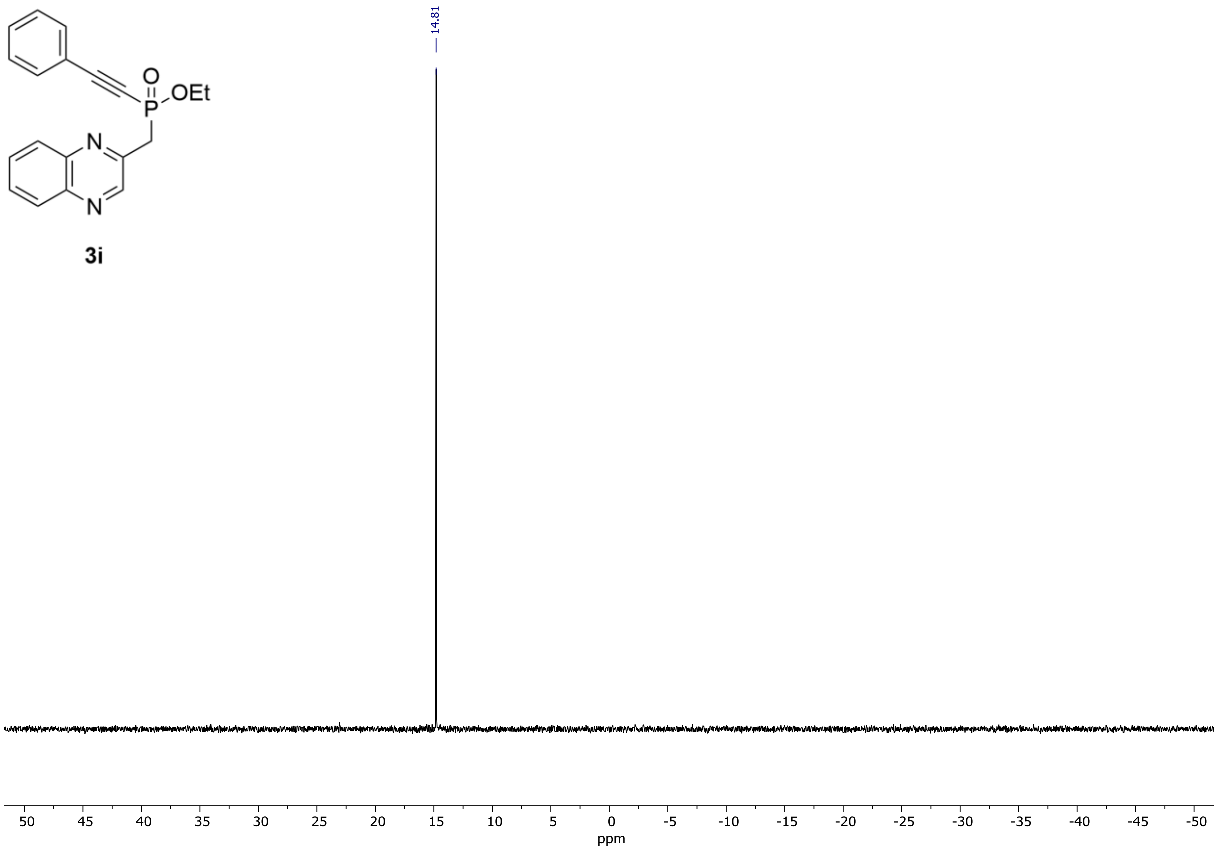

3i

Figure S195 $^{31}P\ \{^{1}H\}$ *NMR spectrum of* ***3i*** *(162 MHz, $CDCl_3$).*

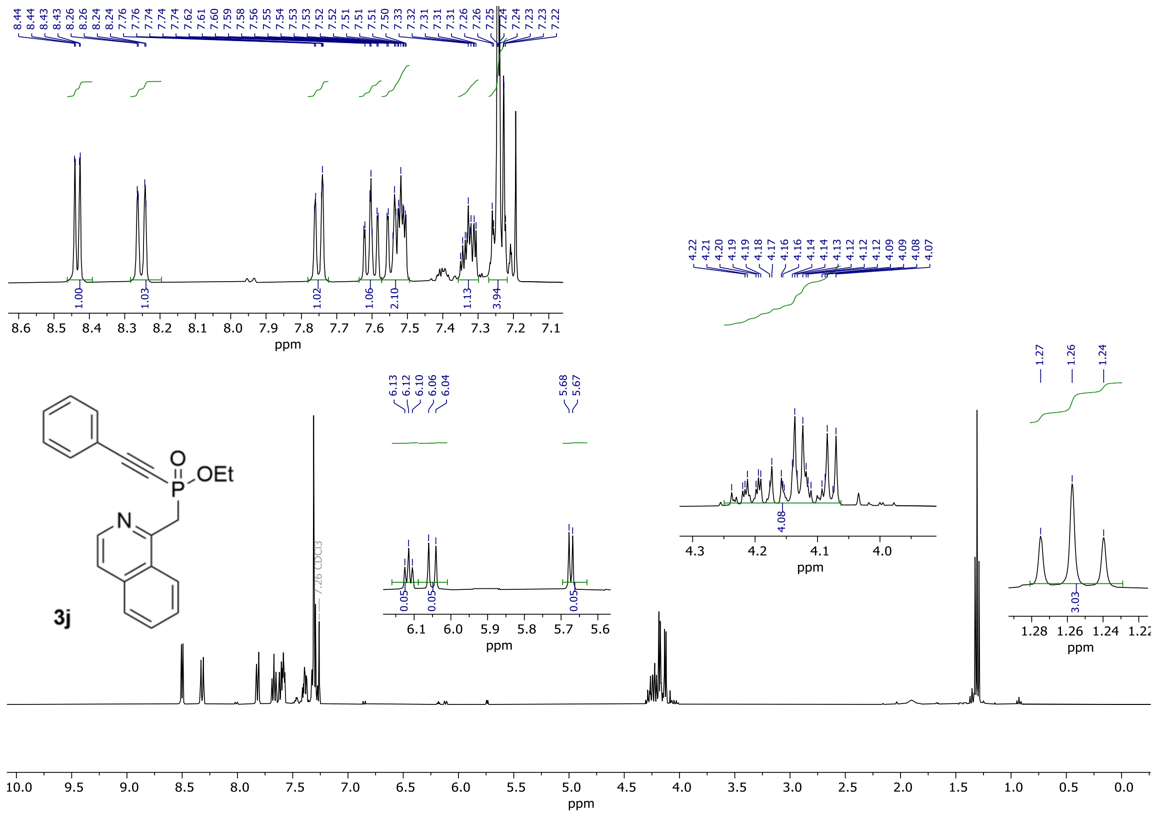

3j

Figure S196 ^{1}H *NMR spectrum of* ***3j*** *(400 MHz, $CDCl_3$). The signals at 5.6-6.2 ppm are attributed to product* ***4j****, formed via spontaneous cyclization.*

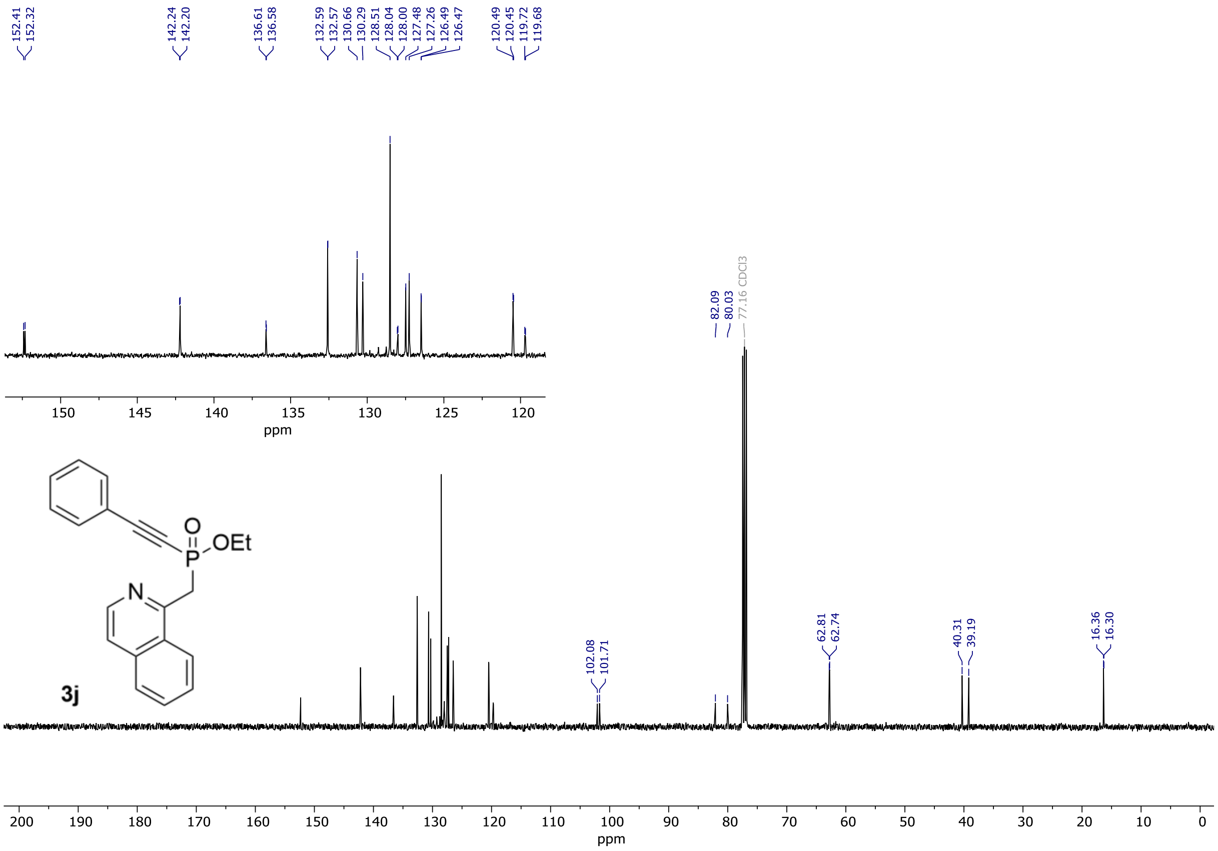

Figure S197 ^{13}C {^{1}H} *NMR spectrum of* ***3j*** *(101 MHz, $CDCl_3$).*

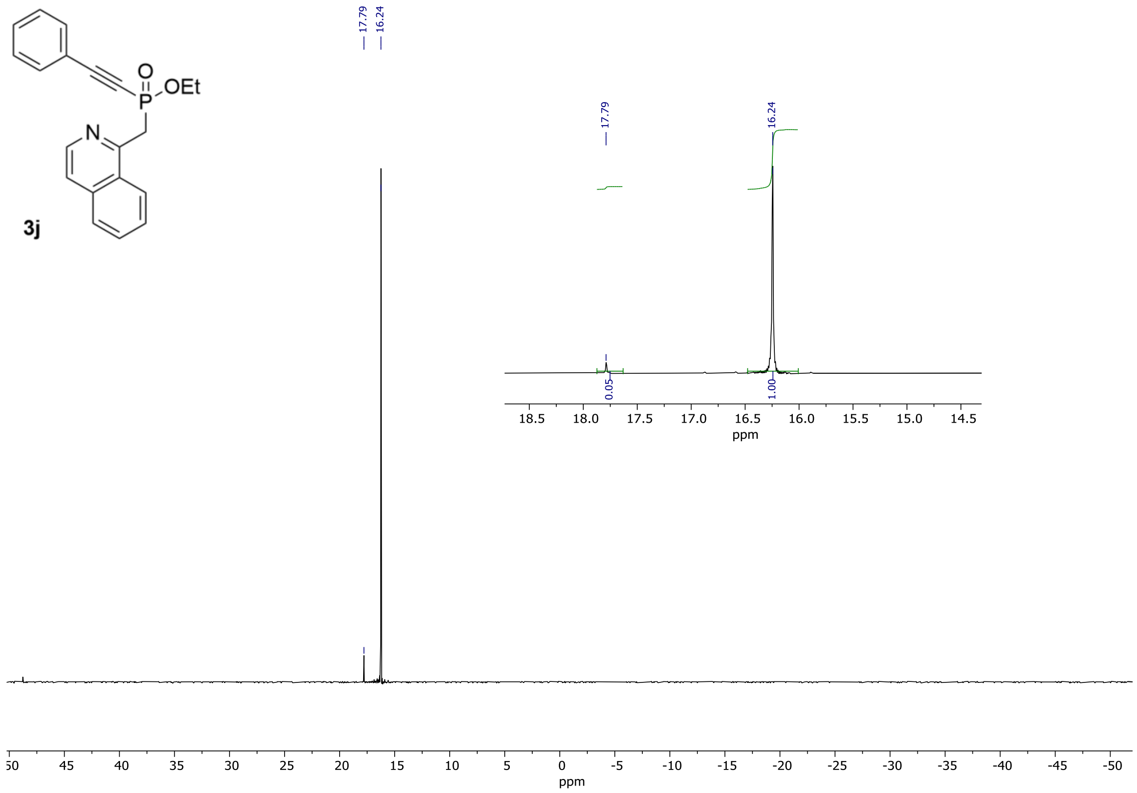

Figure S198 ^{31}P {^{1}H} *NMR spectrum of* ***3j*** *(162 MHz, $CDCl_3$). The signal at 17.79 ppm is attributed to product* ***3j****, formed via spontaneous cyclization.*

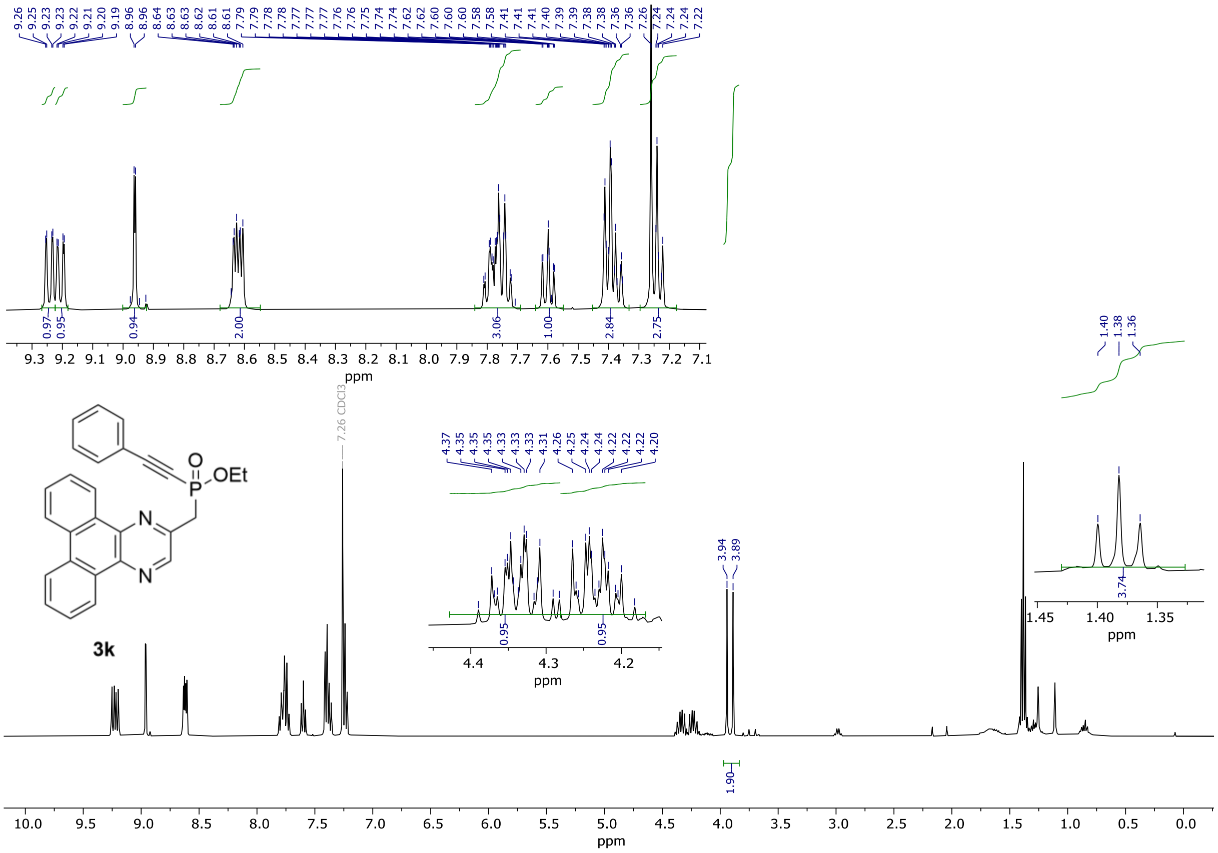

Figure S199 *^{1}H NMR spectrum of **3k** (400 MHz, $CDCl_3$).*

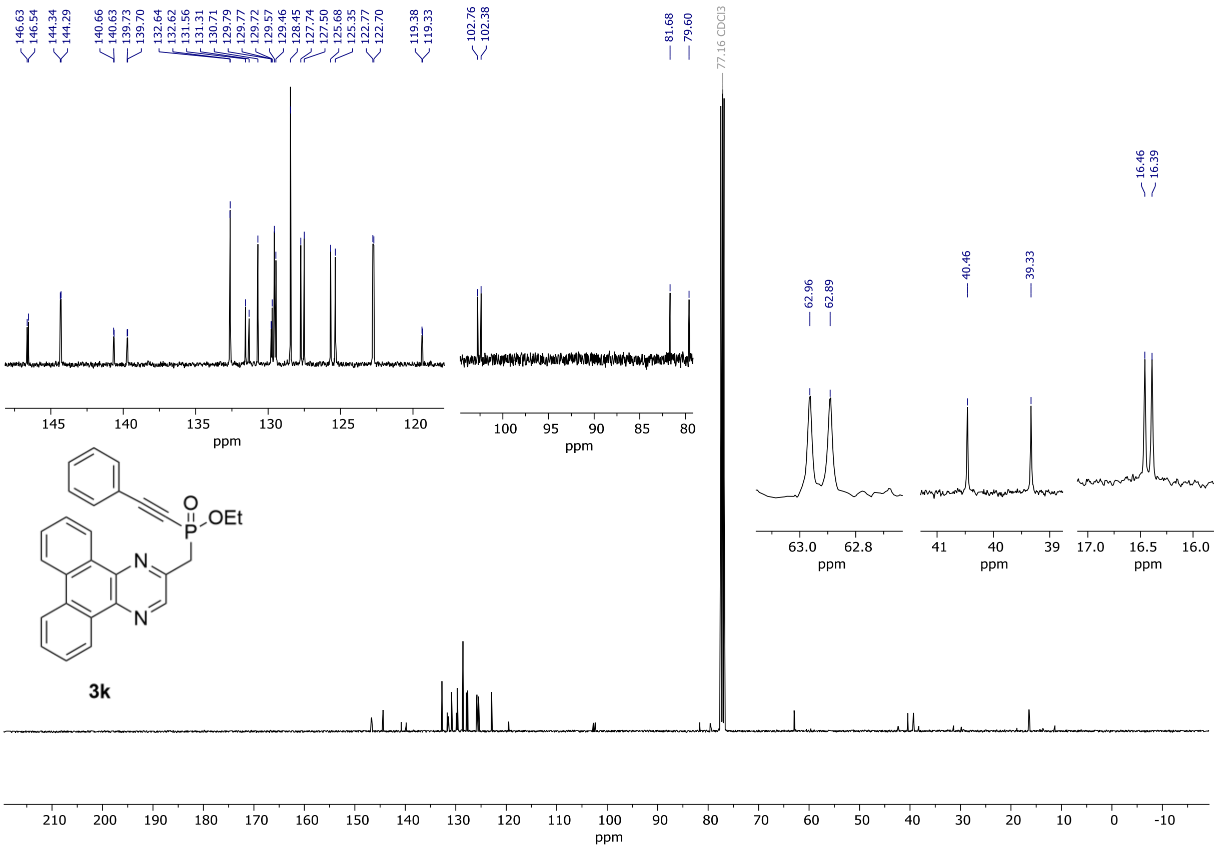

Figure S200 *^{13}C {^{1}H} NMR spectrum of **3k** (101 MHz, $CDCl_3$).*

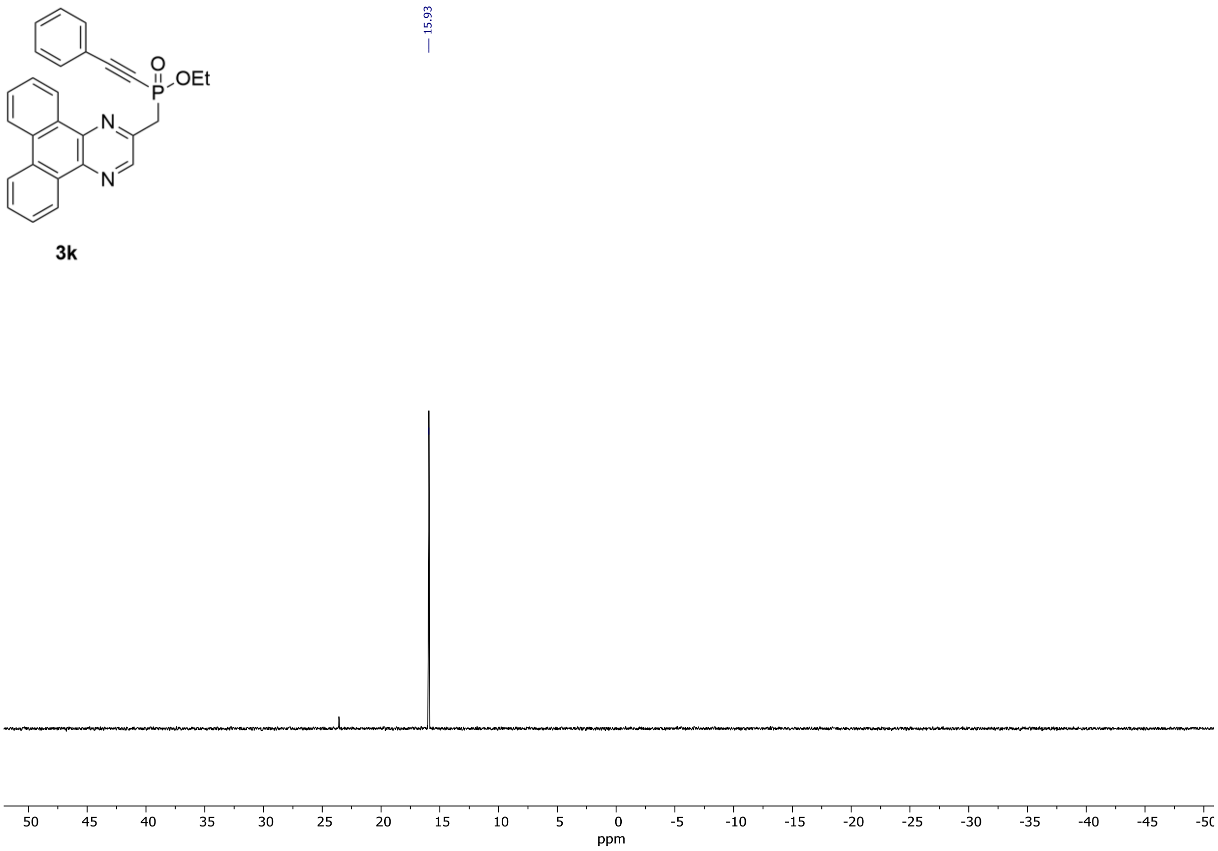

Figure S201 ^{31}P {^{1}H} *NMR spectrum of* ***3k*** *(162 MHz,* $CDCl_3$*).*

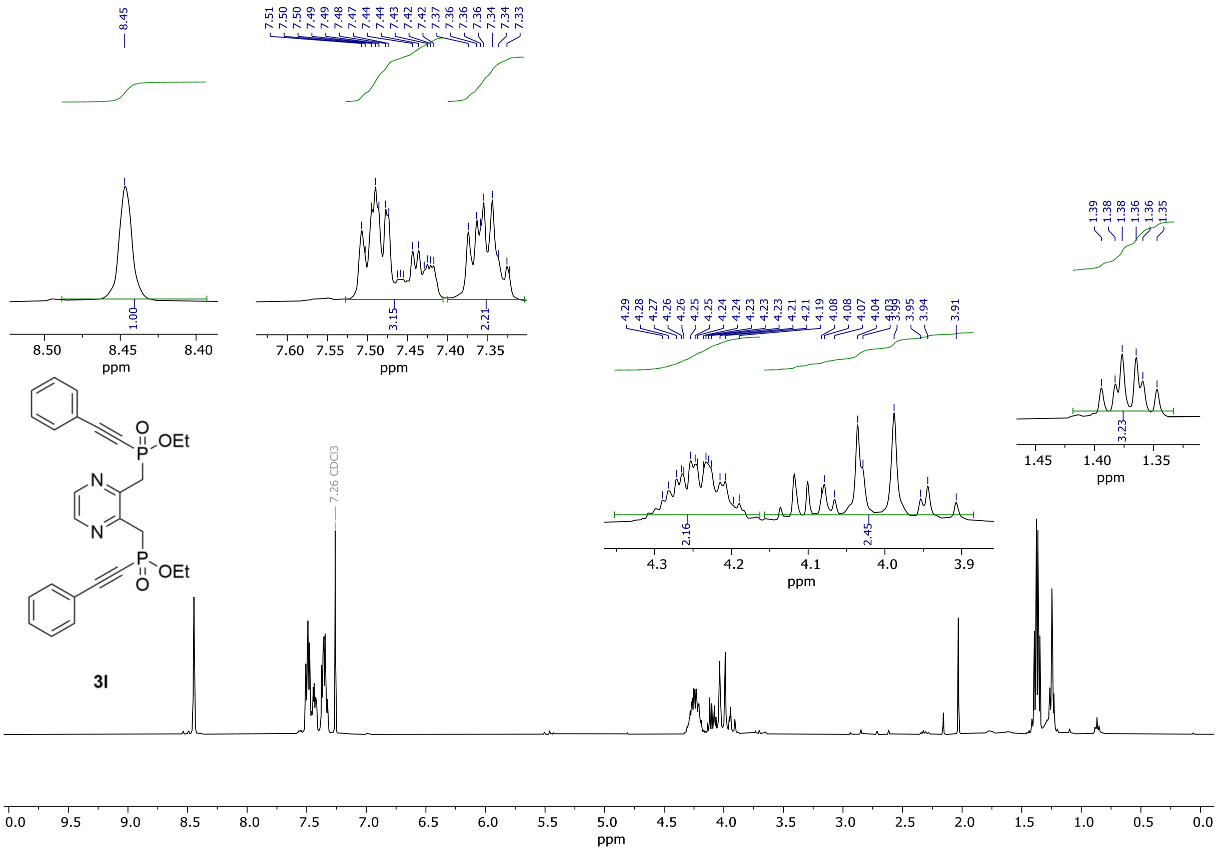

Figure S202 ^{1}H *NMR spectrum of* ***3l*** *(400 MHz,* $CDCl_3$*).*

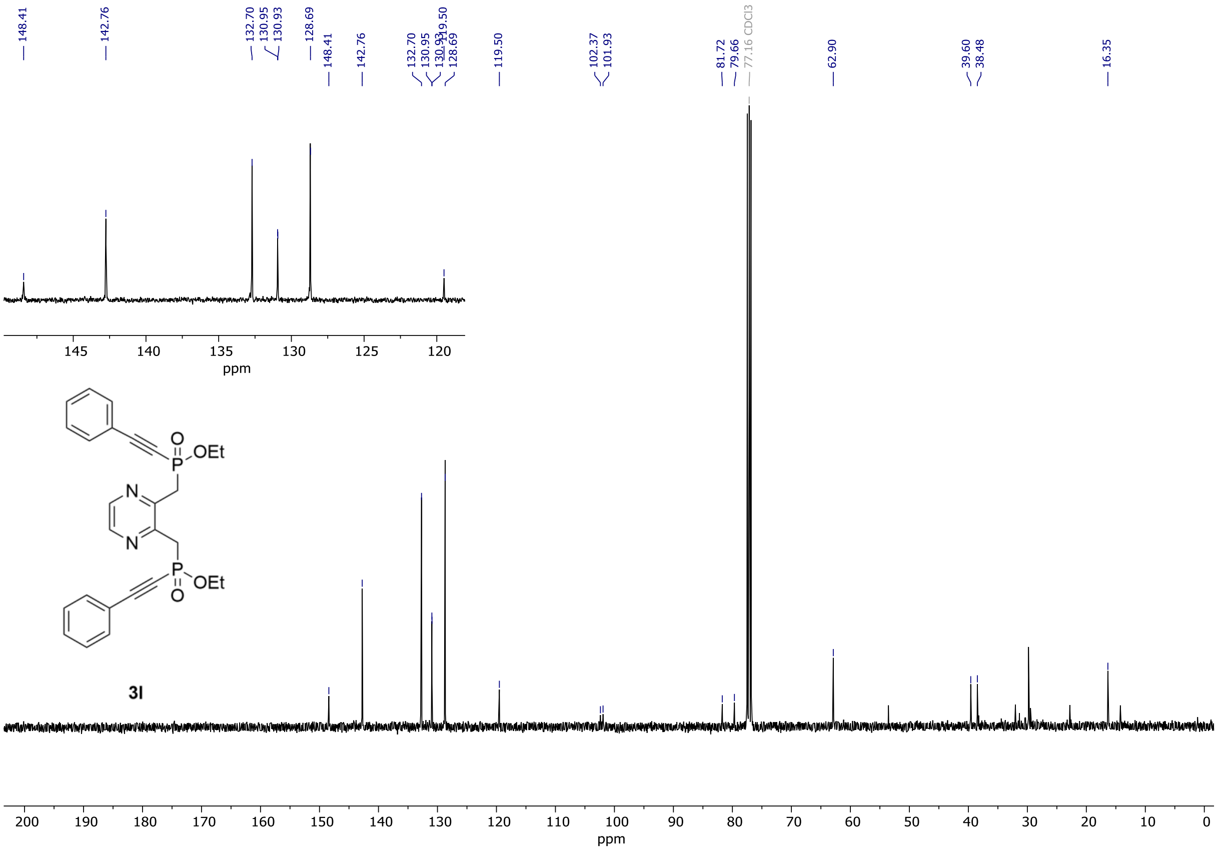

Figure S203 *^{13}C {^{1}H} NMR spectrum of **3l** (101 MHz, $CDCl_3$).*

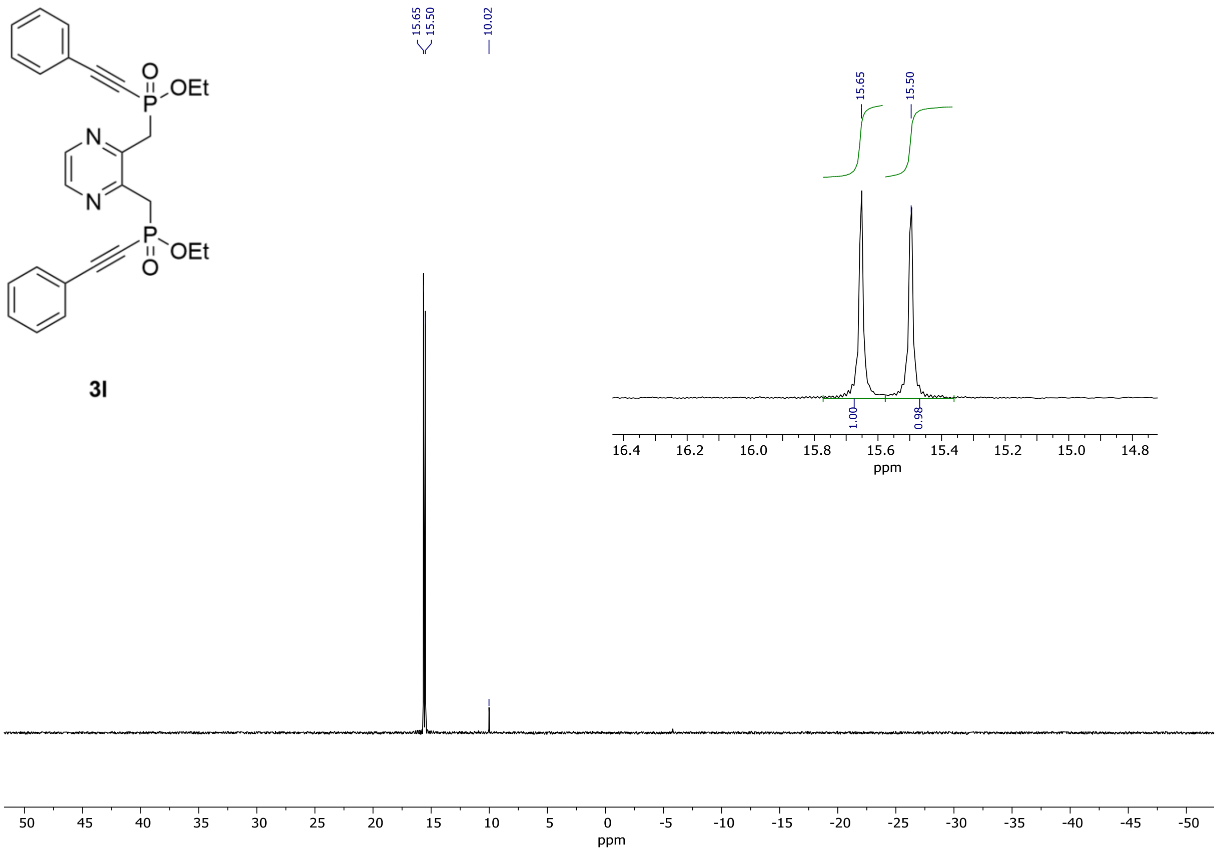

Figure S204 *^{31}P {^{1}H} NMR spectrum of **3l** (162 MHz, $CDCl_3$).*

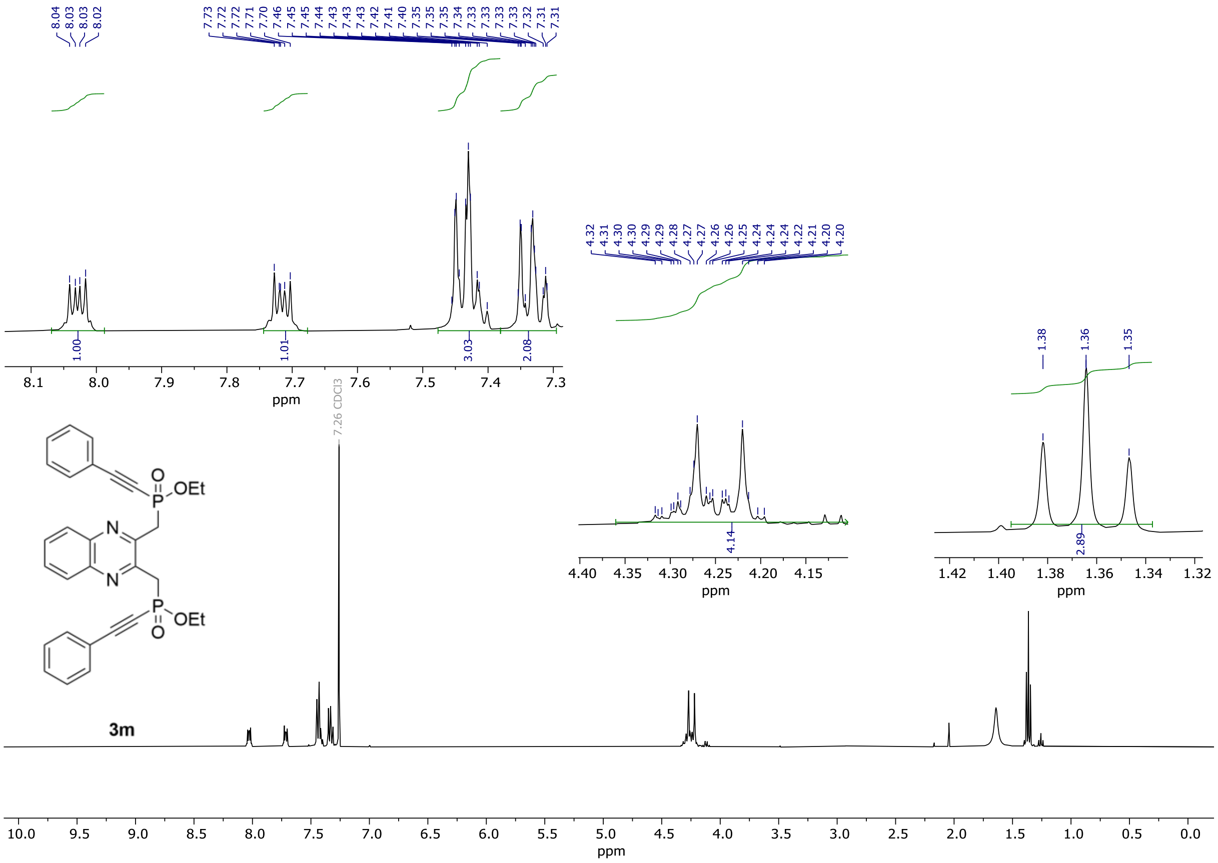

Figure S205 ^{1}H NMR spectrum of **3m** (400 MHz, $CDCl_3$).

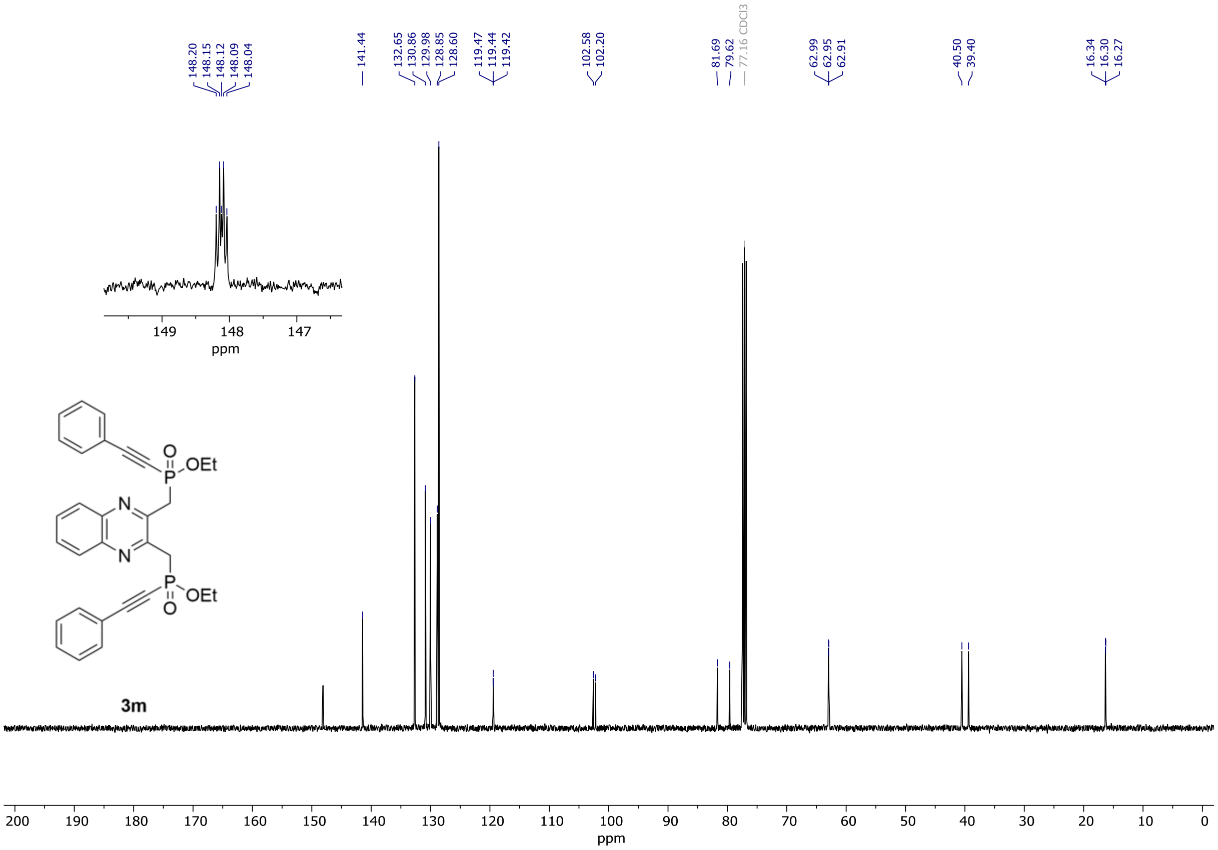

Figure S206 ^{13}C {^{1}H} NMR spectrum of **3m** (101 MHz, $CDCl_3$).

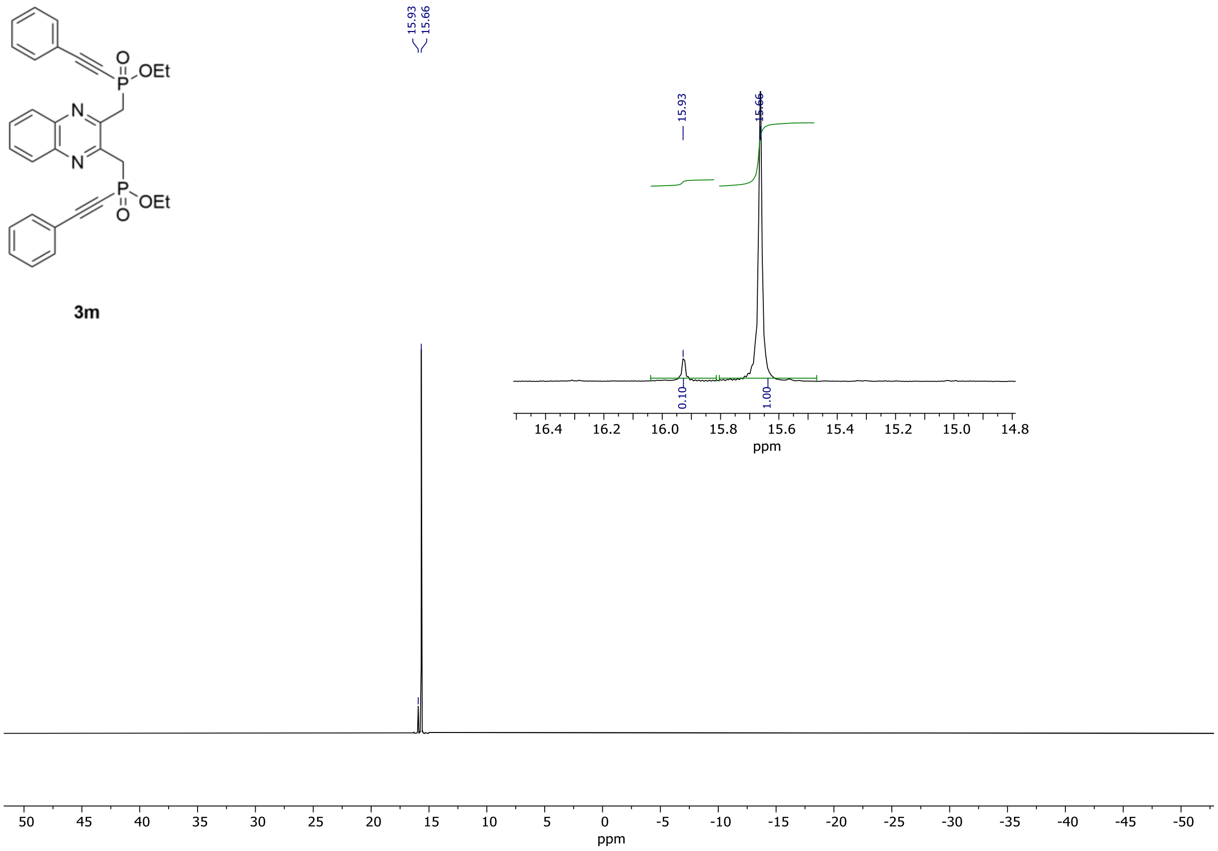

Figure S207 $^{31}P\{^1H\}$ *NMR spectrum of* ***3m*** *(162 MHz, $CDCl_3$).*

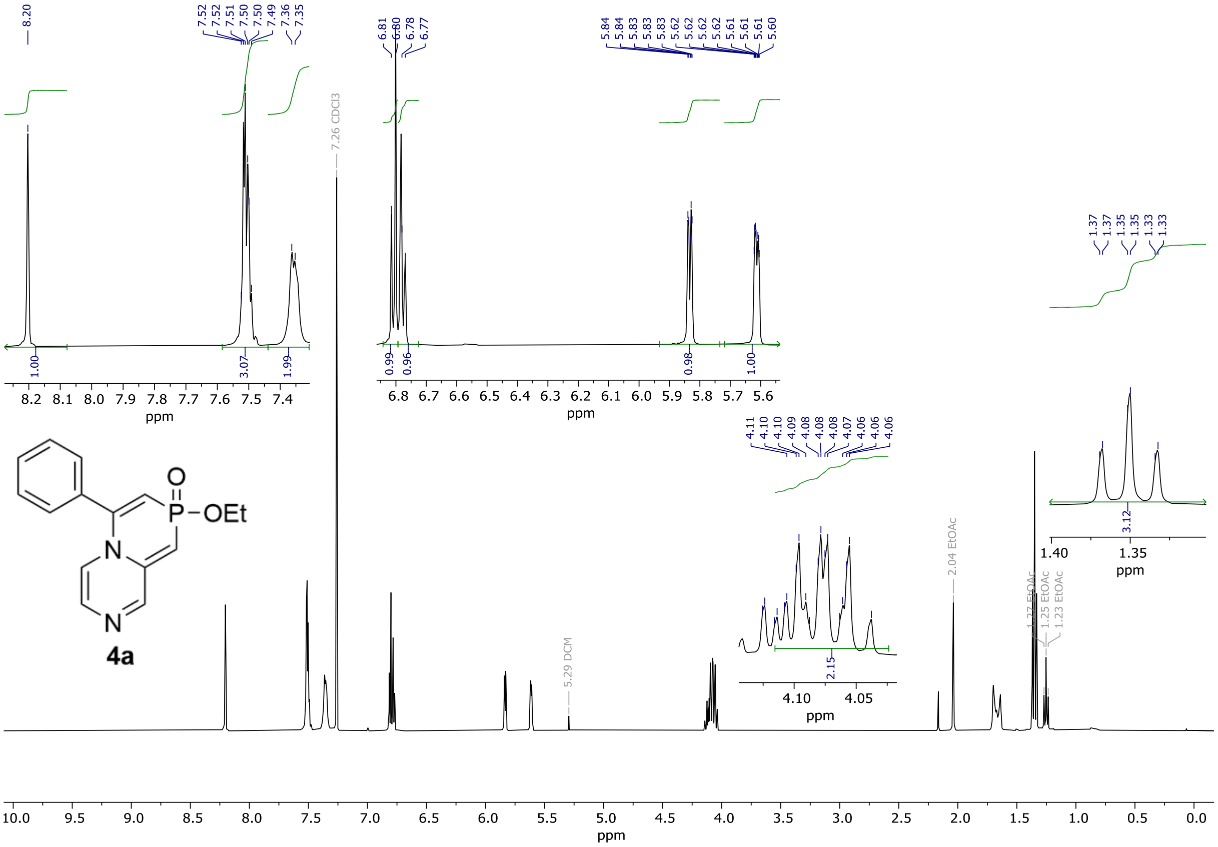

Figure S208 ^1H *NMR spectrum of* ***4a*** *(400 MHz, $CDCl_3$).*

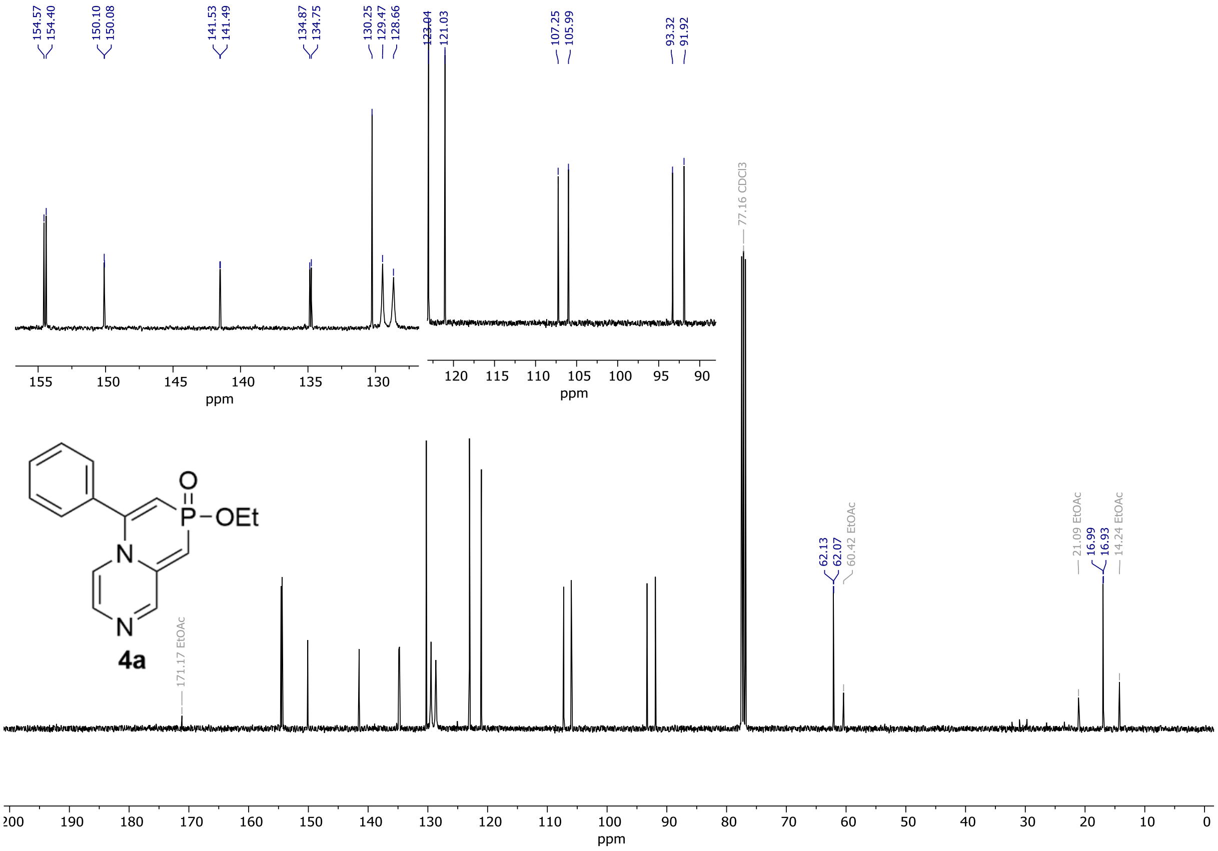

Figure S209 $^{13}C\{^{1}H\}$ *NMR spectrum of* ***4a*** *(101 MHz, $CDCl_3$).*

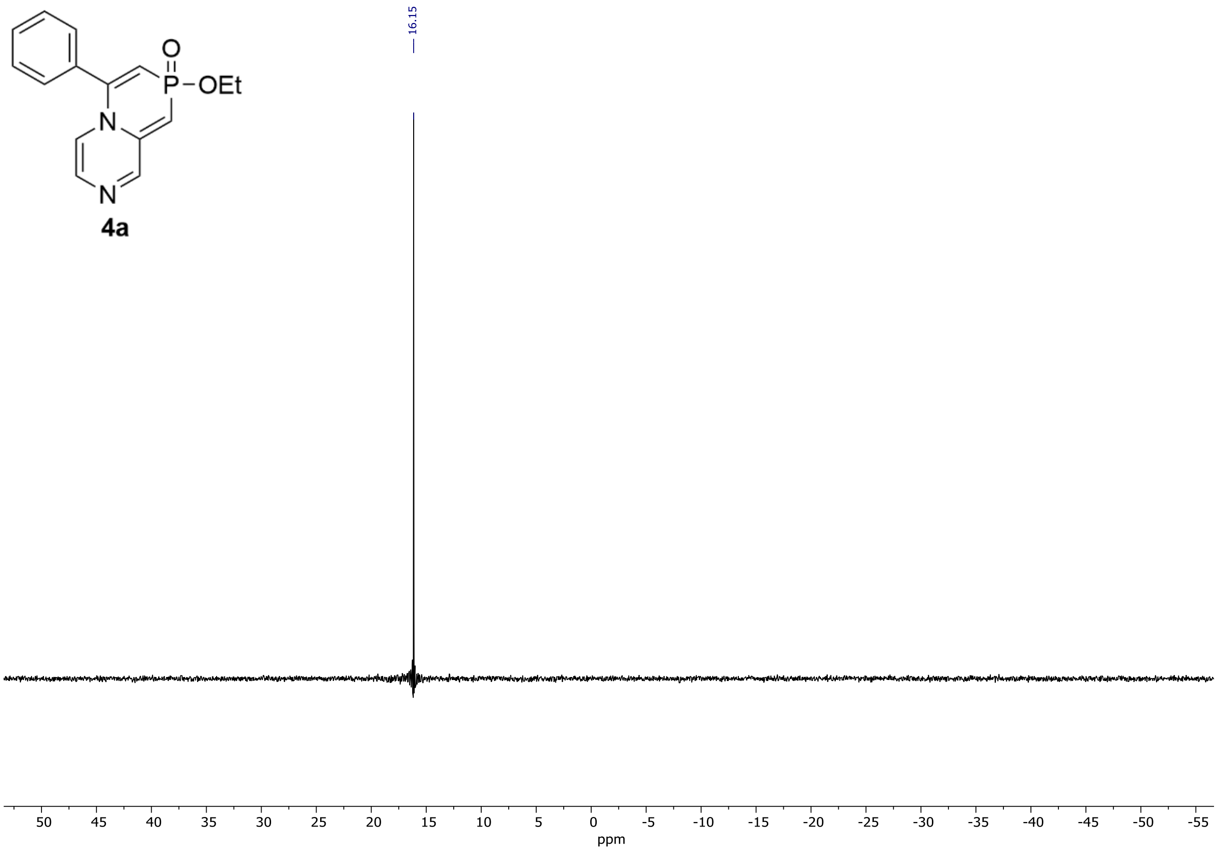

Figure S210 $^{31}P\{^{1}H\}$ *NMR spectrum of* ***4a*** *(162 MHz, $CDCl_3$).*

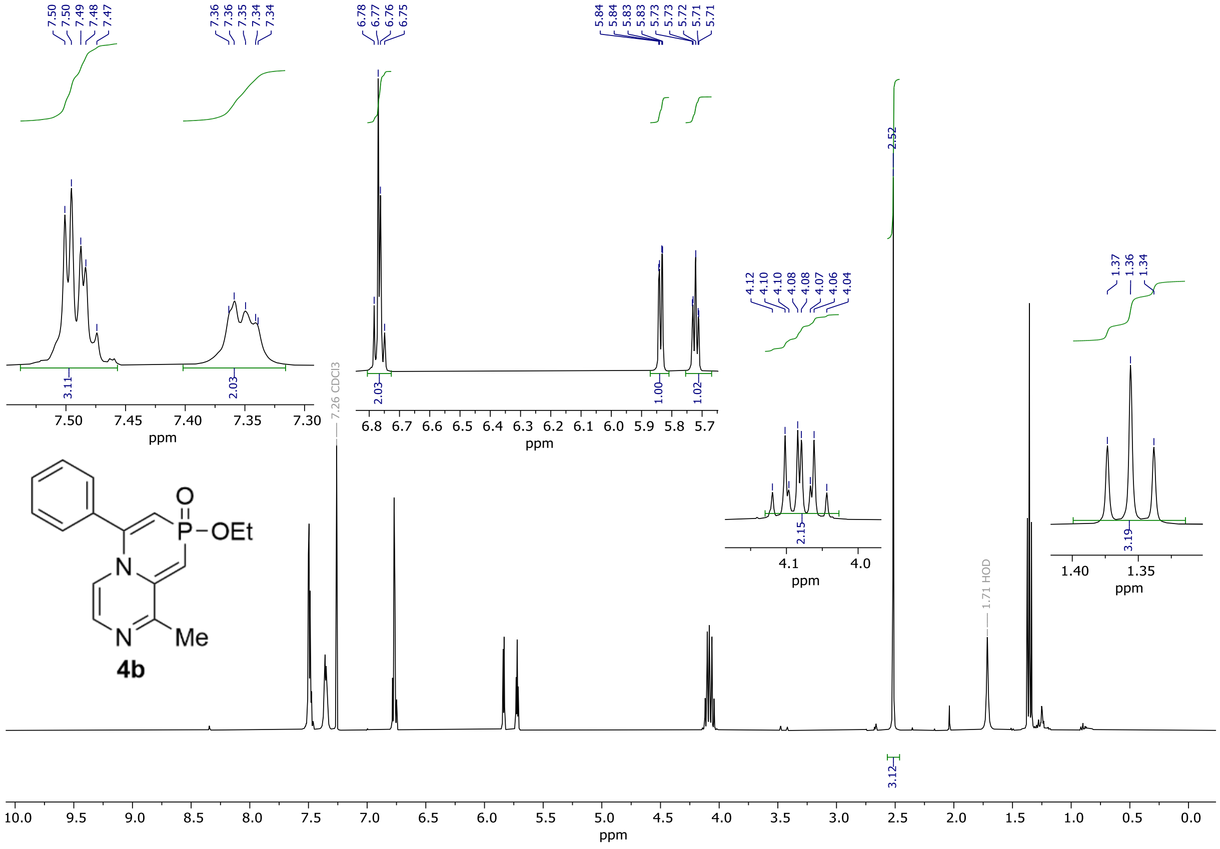

Figure S211 *¹H NMR spectrum of* ***4b*** *(400 MHz, $CDCl_3$).*

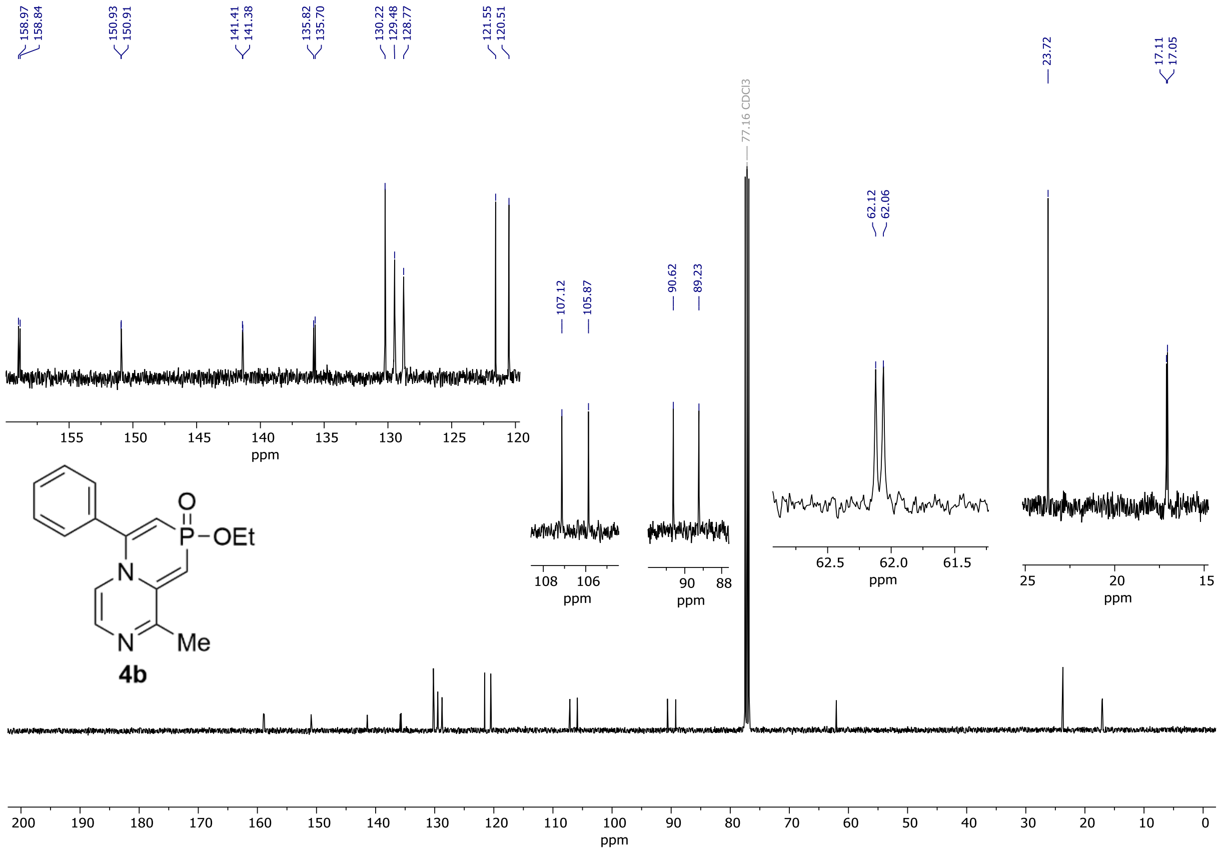

Figure S212 *$^{13}C\{^1H\}$ NMR spectrum of* ***4b*** *(101 MHz, $CDCl_3$).*

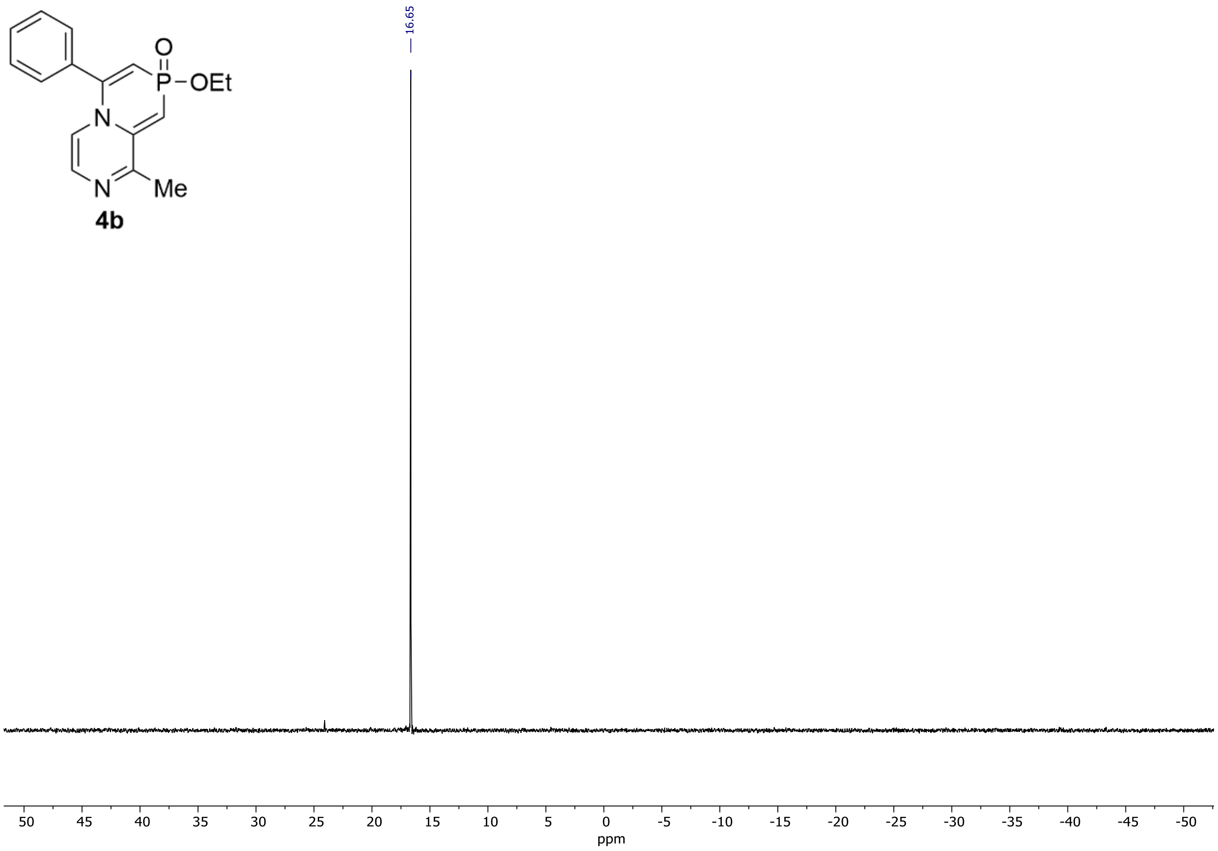

Figure S213 $^{31}P\ \{^1H\}$ *NMR spectrum of* ***4b*** *(162 MHz,* $CDCl_3$*).*

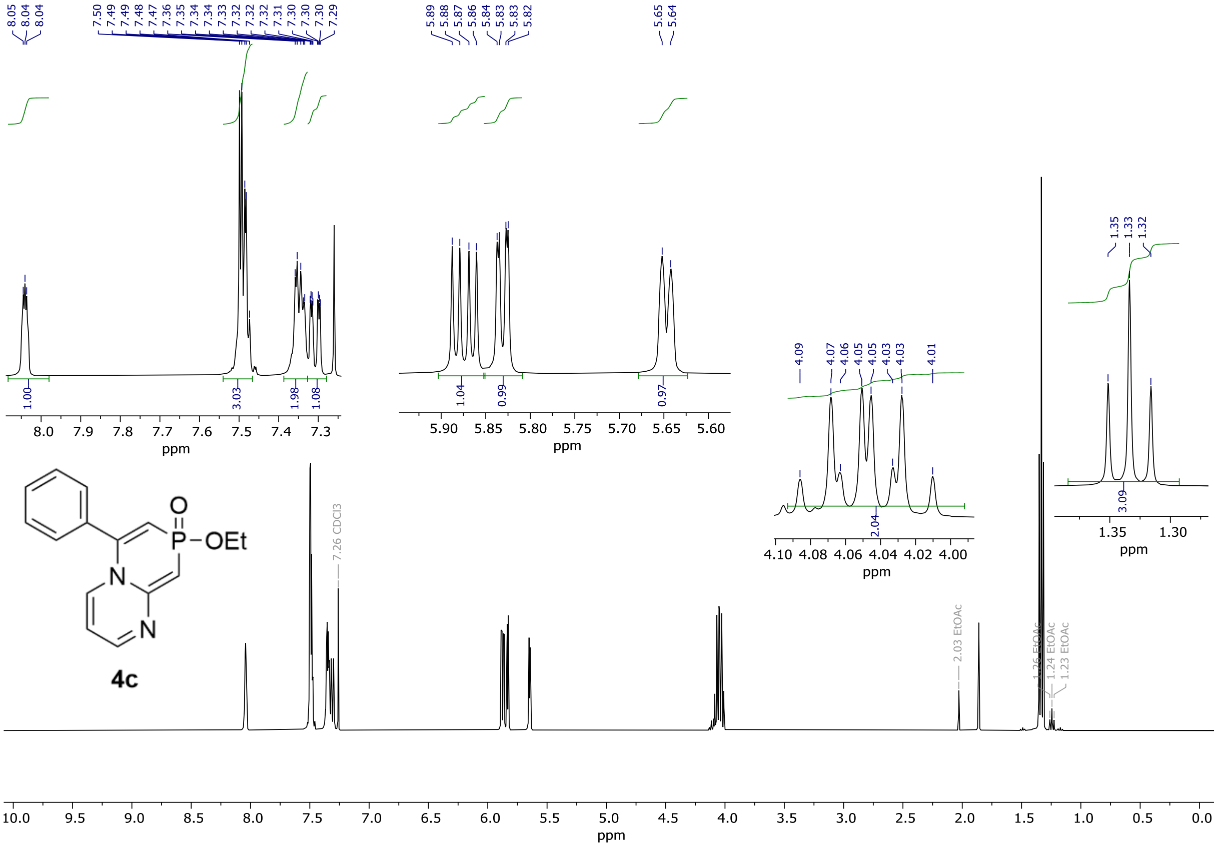

Figure S214 ^1H *NMR spectrum of* ***4c*** *(400 MHz,* $CDCl_3$*).*

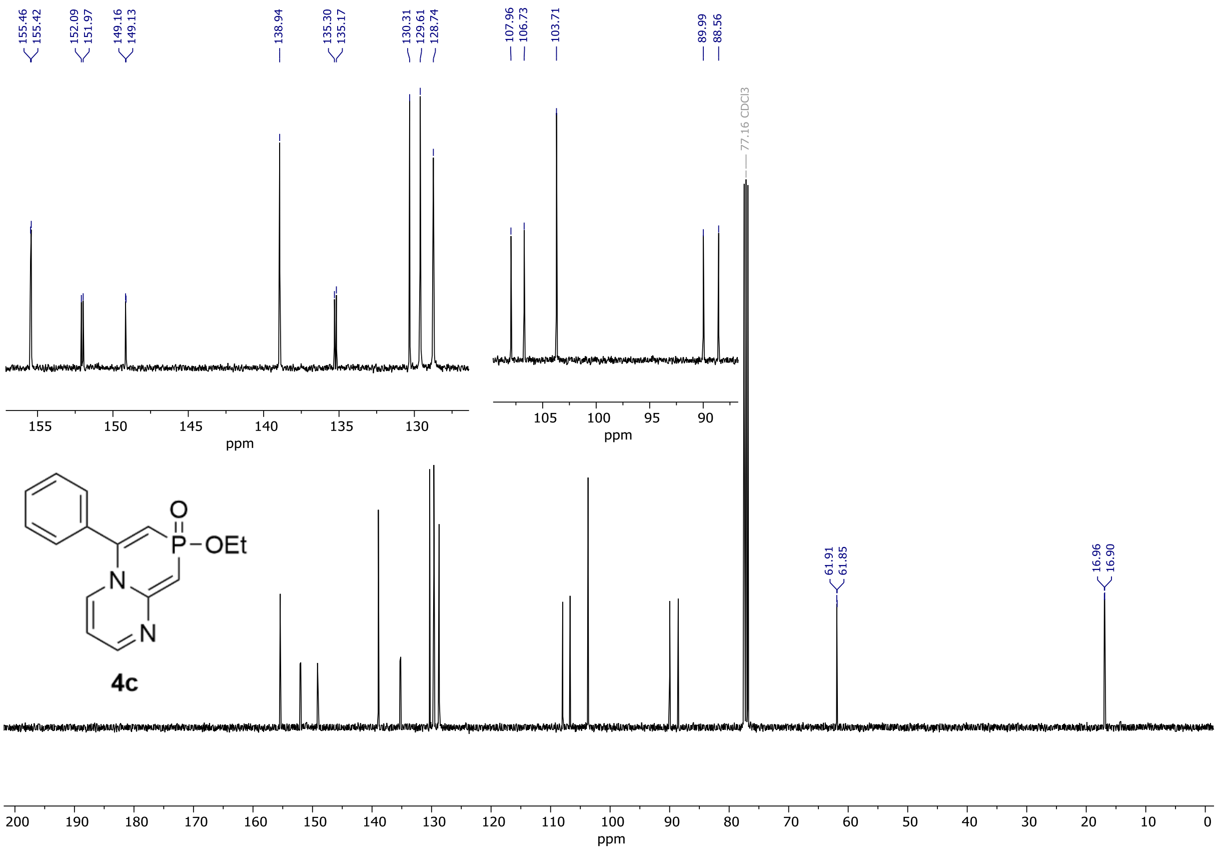

Figure S215 *^{13}C {^{1}H} NMR spectrum of* ***4c*** *(101 MHz, $CDCl_3$).*

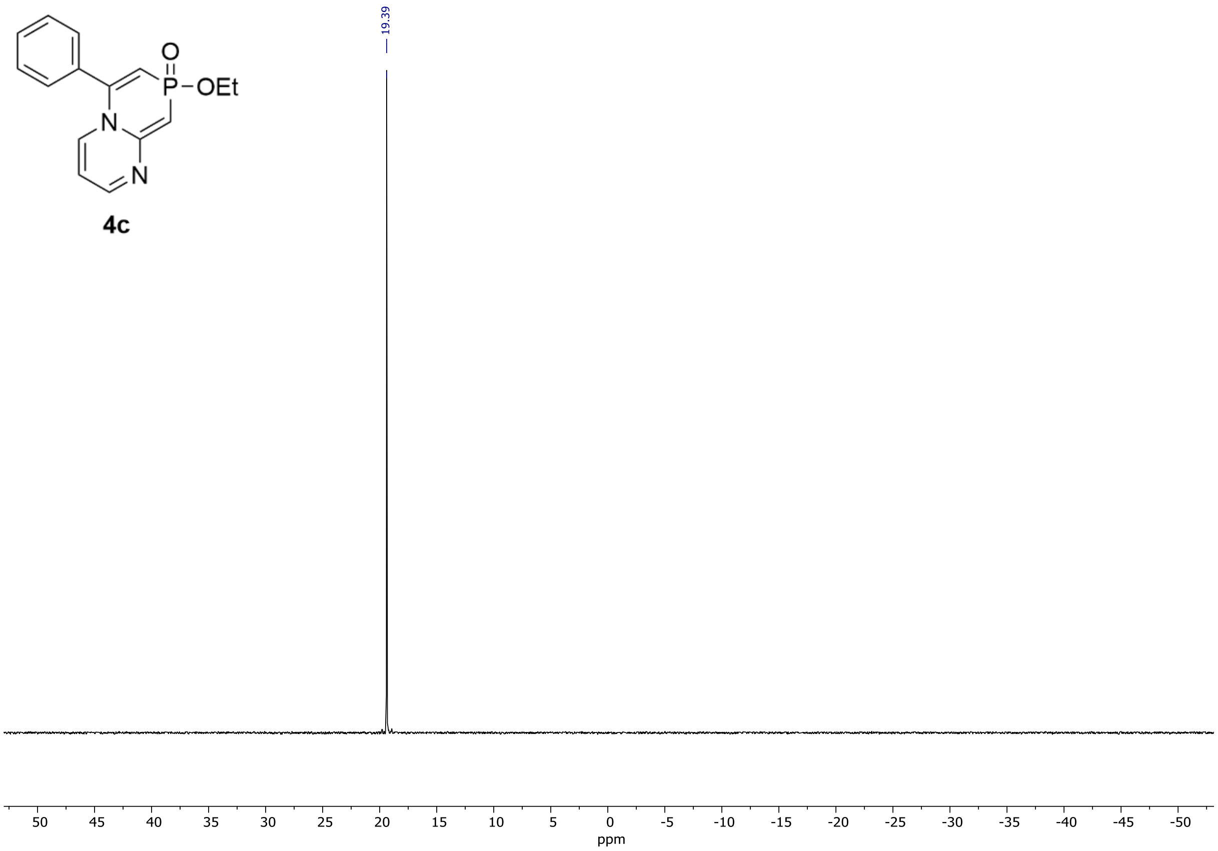

Figure S216 *^{31}P {^{1}H} NMR spectrum of* ***4c*** *(162 MHz, $CDCl_3$).*

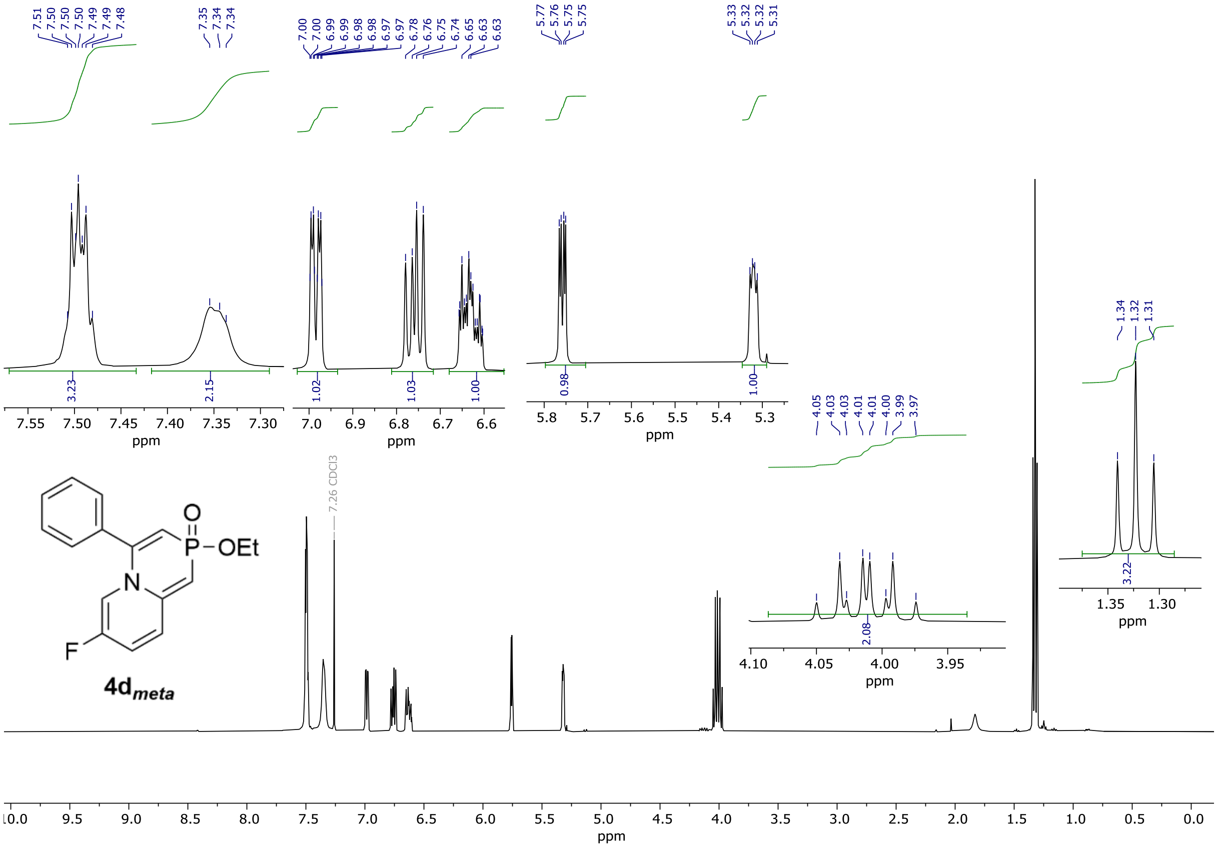

Figure S217 *^{1}H NMR spectrum of* ***$4d_{meta}$*** *(400 MHz, $CDCl_3$).*

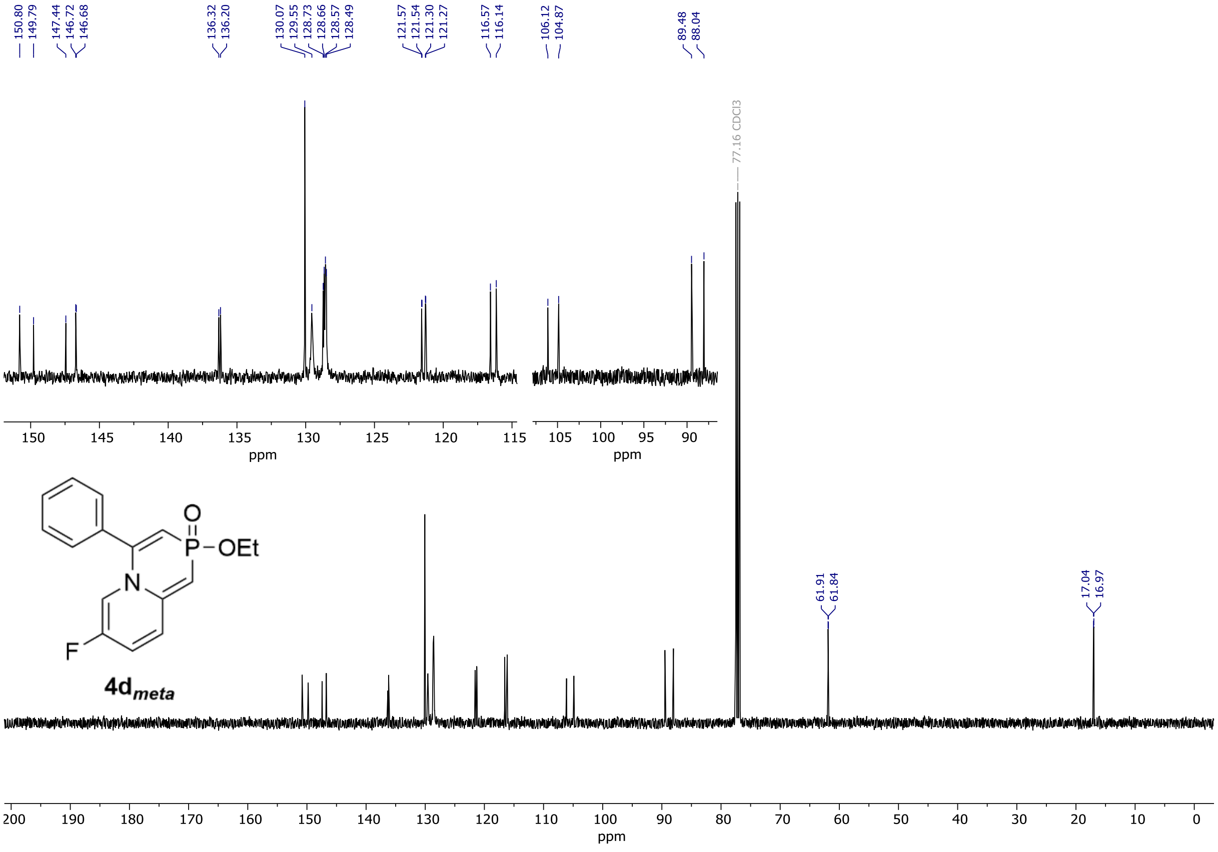

Figure S218 *^{13}C {^1H} NMR spectrum of* ***$4d_{meta}$*** *(101 MHz, $CDCl_3$).*

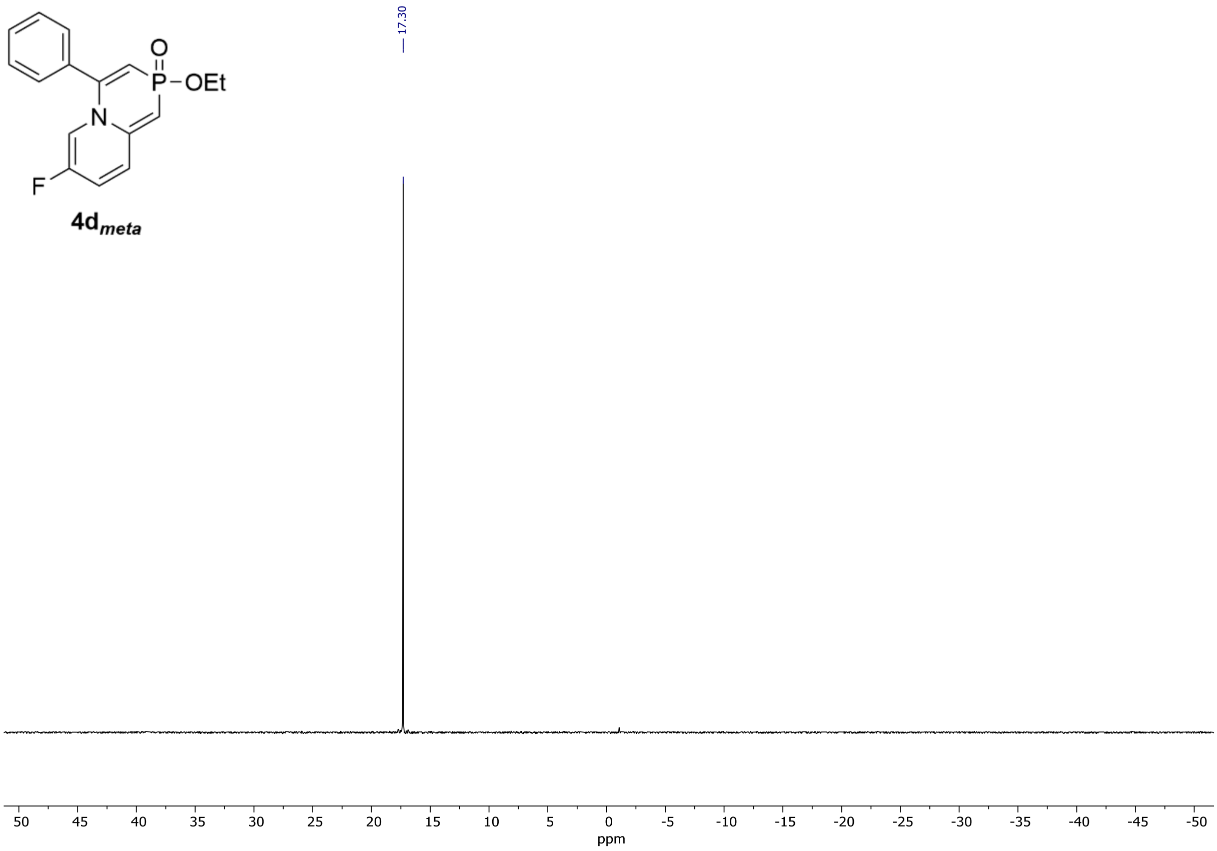

Figure S219 ^{31}P {^{1}H} *NMR spectrum of* ***4d$_{meta}$*** *(162 MHz, CDCl$_3$).*

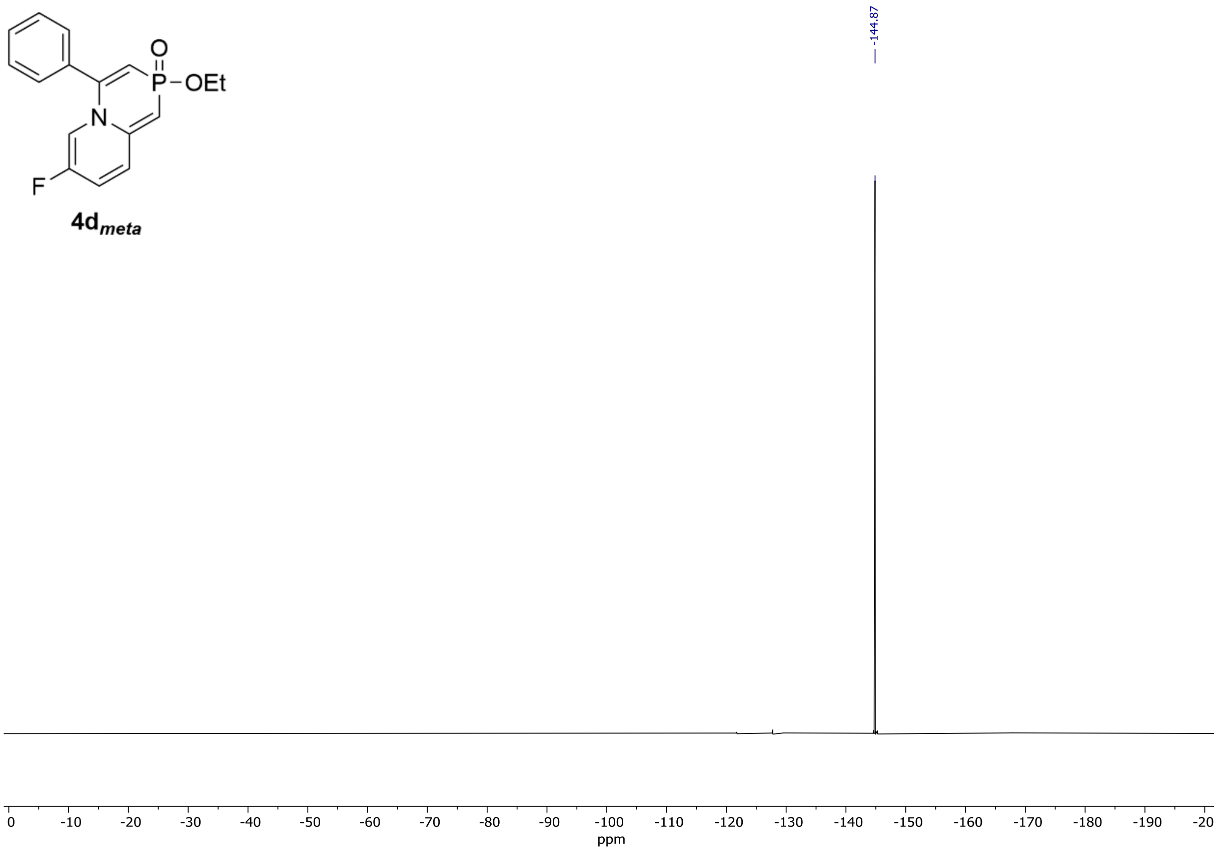

Figure S220 ^{19}F {^{1}H} *NMR spectrum of* ***4d$_{meta}$*** *(376 MHz, CDCl$_3$).*

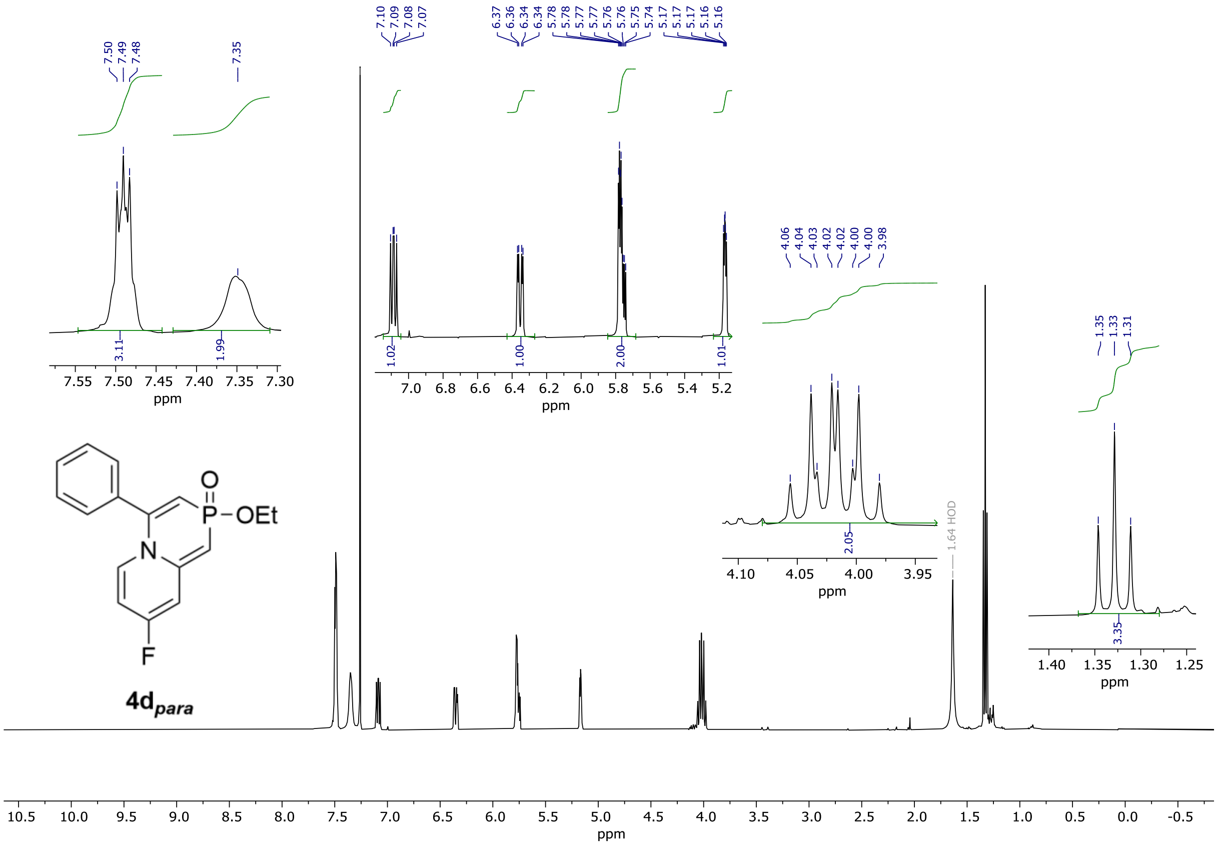

Figure S221 *¹H NMR spectrum of* ***4d$_{para}$*** *(400 MHz, $CDCl_3$).*

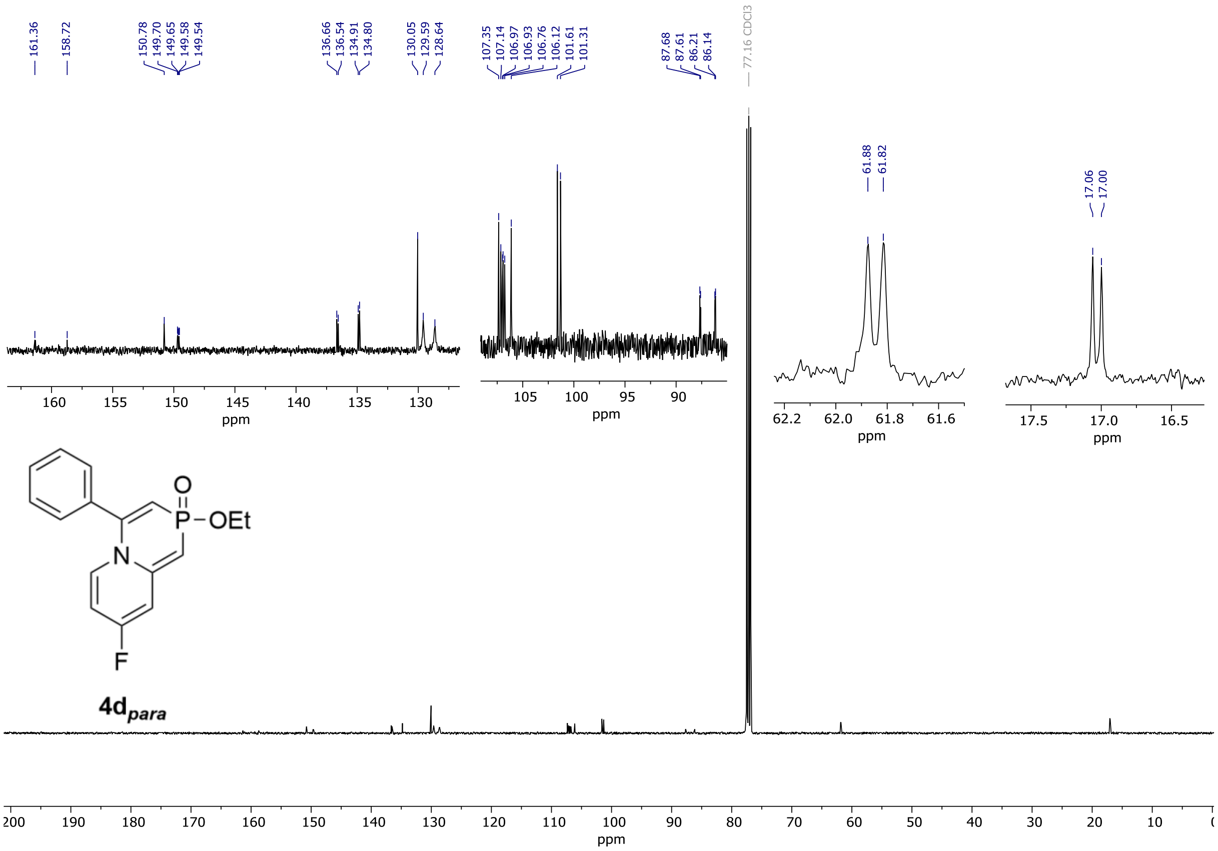

Figure S222 *¹³C {¹H} NMR spectrum of* ***4d$_{para}$*** *(101 MHz, $CDCl_3$).*

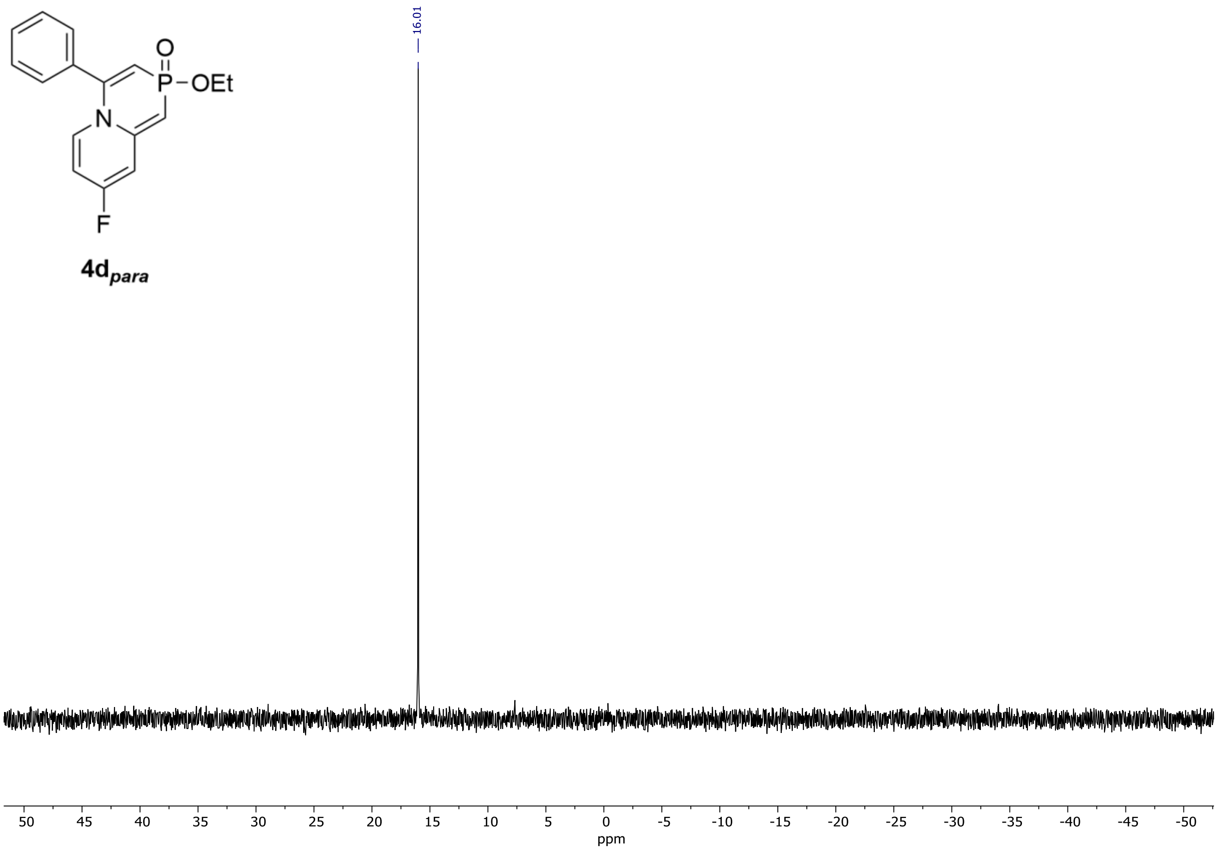

Figure S223 *^{31}P {^{1}H} NMR spectrum of **4d$_{para}$** (162 MHz, CDCl$_3$).*

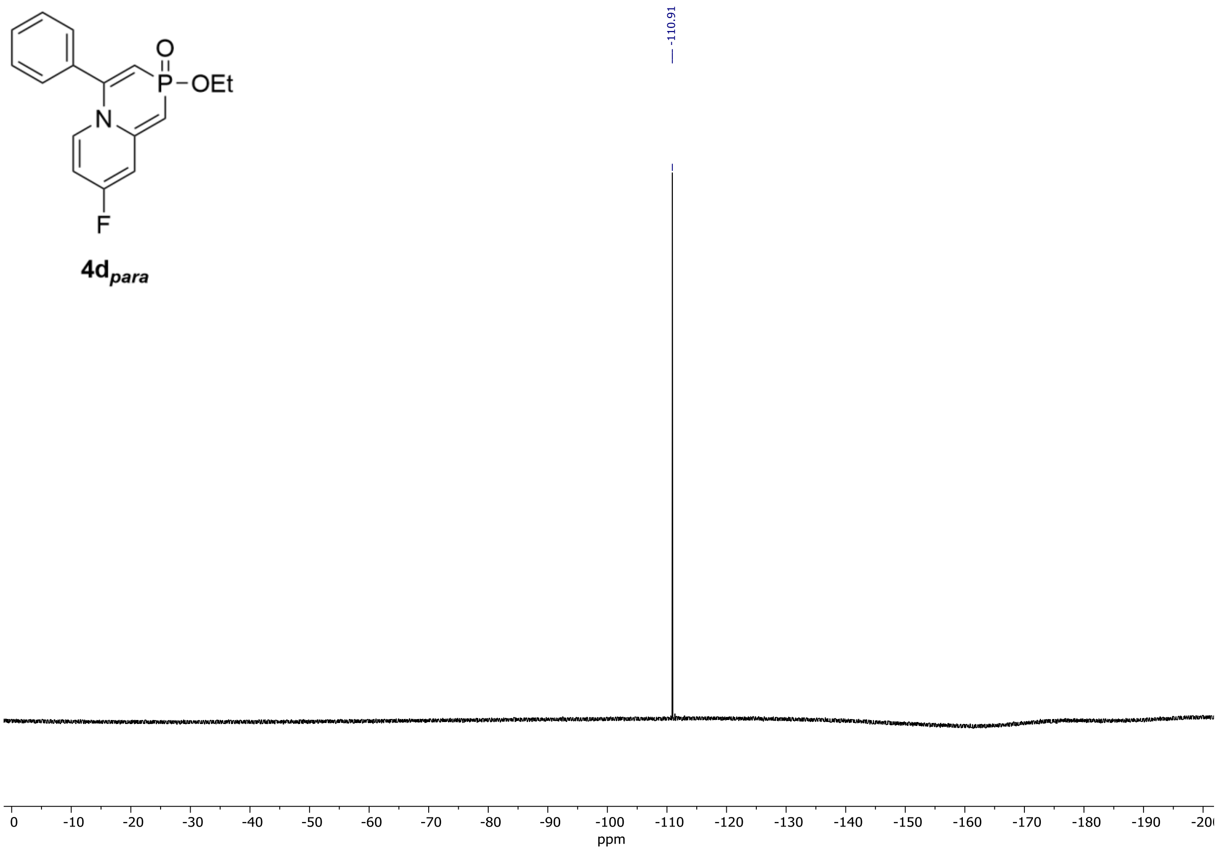

Figure S224 *^{19}F {^{1}H} NMR spectrum of **4d$_{para}$** (376 MHz, CDCl$_3$).*

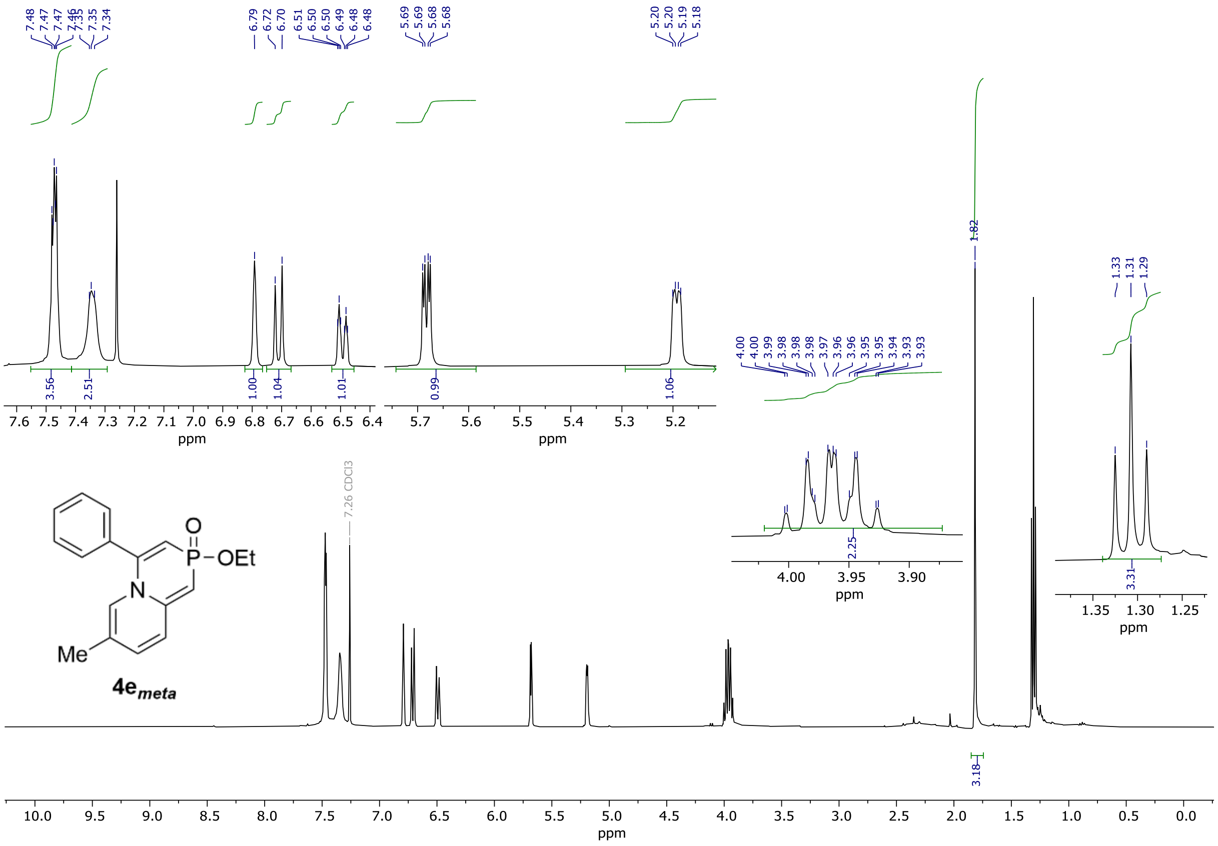

Figure S225 ^{1}*H NMR spectrum of* ***4e$_{meta}$*** *(400 MHz, $CDCl_3$).*

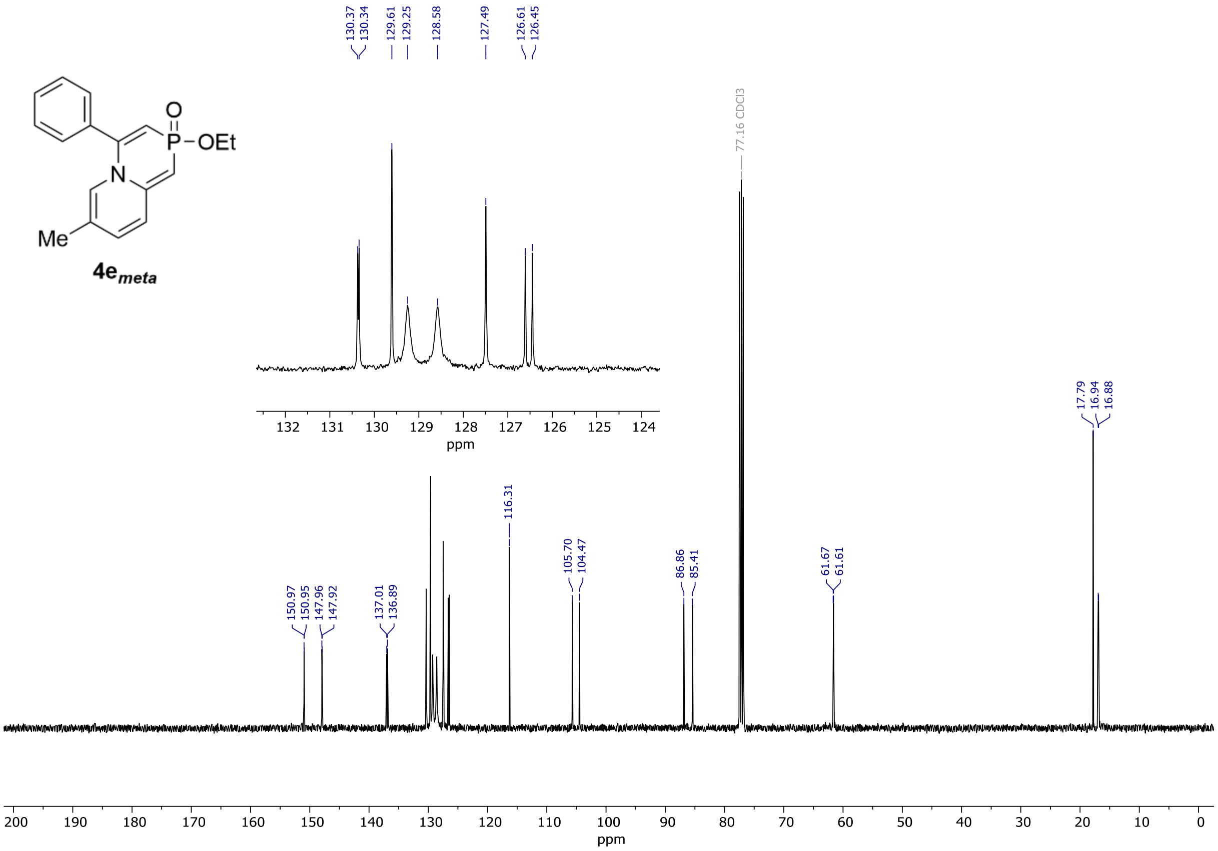

Figure S226 ^{13}*C {^{1}H} NMR spectrum of* ***4e$_{meta}$*** *(101 MHz, $CDCl_3$).*

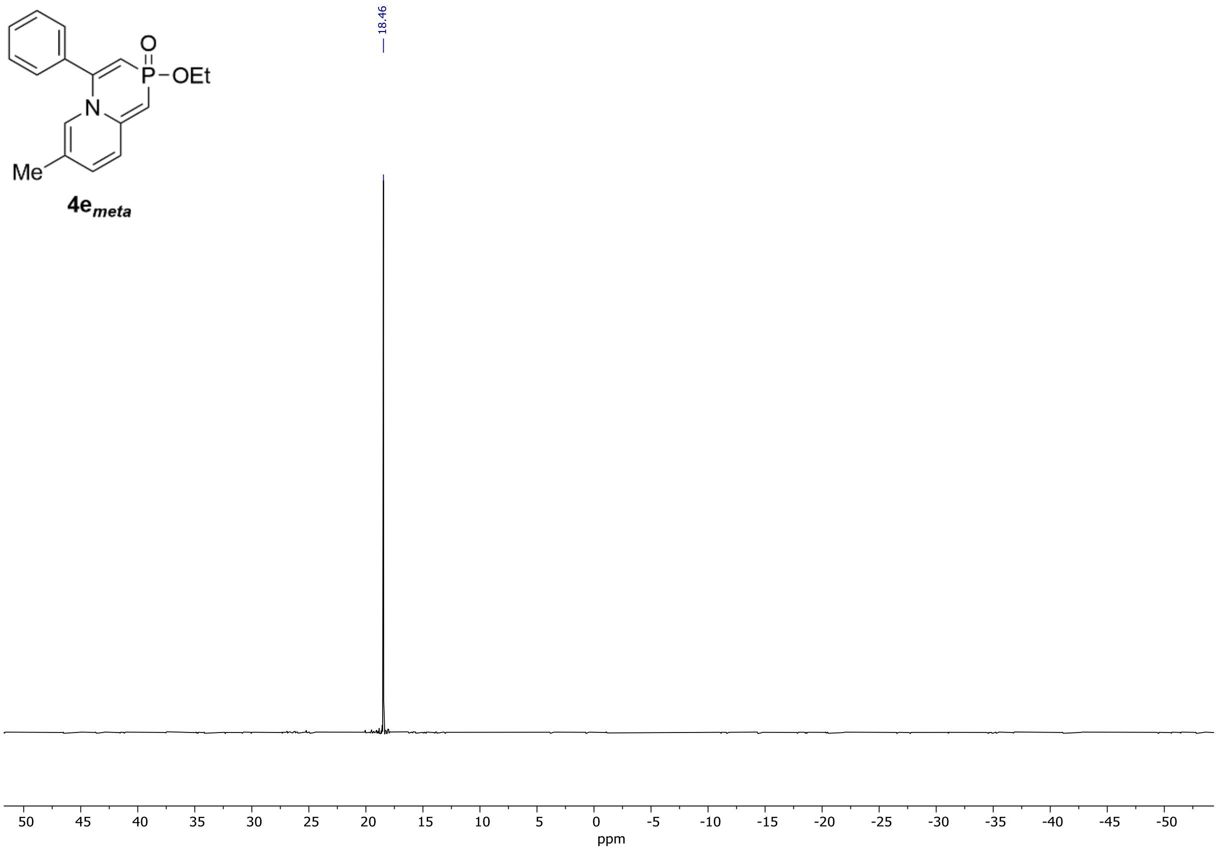

Figure S227 $^{31}P\ \{^1H\}$ *NMR spectrum of* ***$4e_{meta}$*** *(162 MHz, $CDCl_3$).*

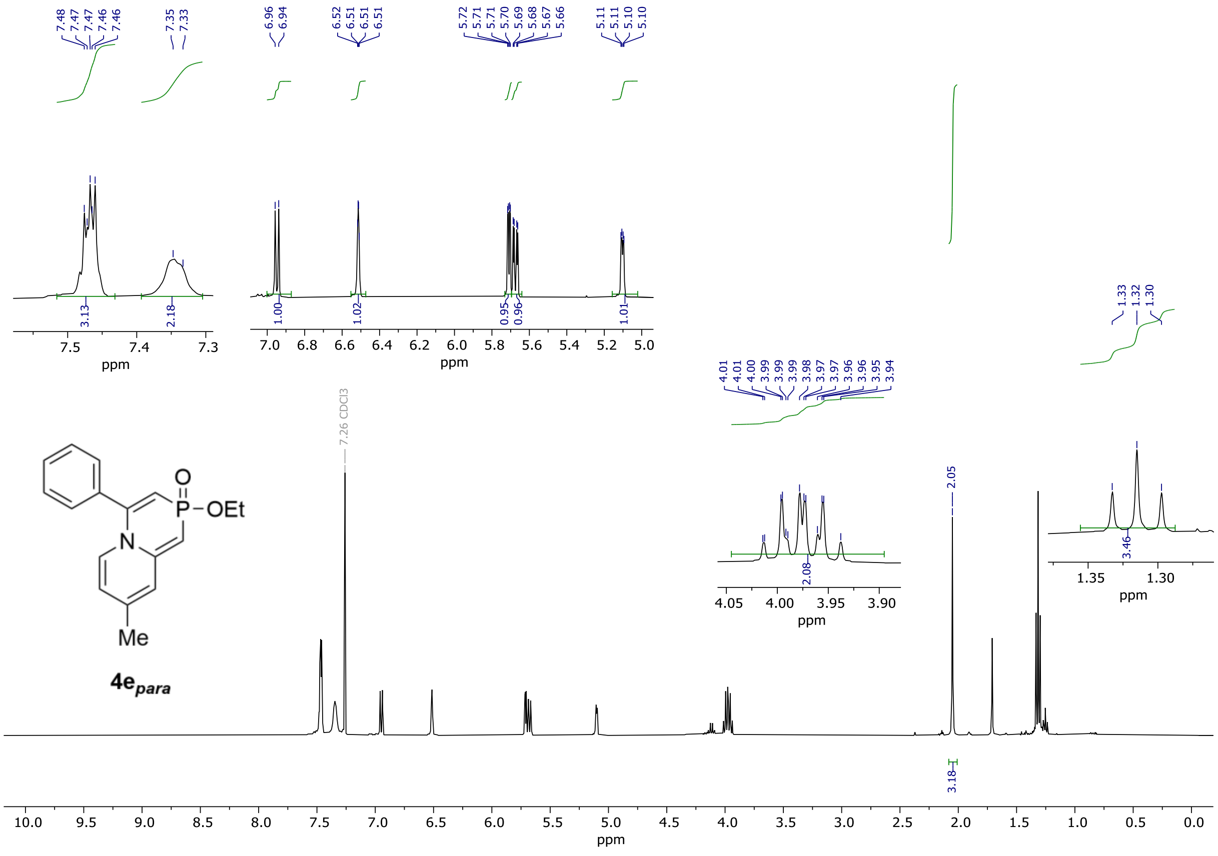

Figure S228 ^{1}H *NMR spectrum of* ***$4e_{para}$*** *(400 MHz, $CDCl_3$).*

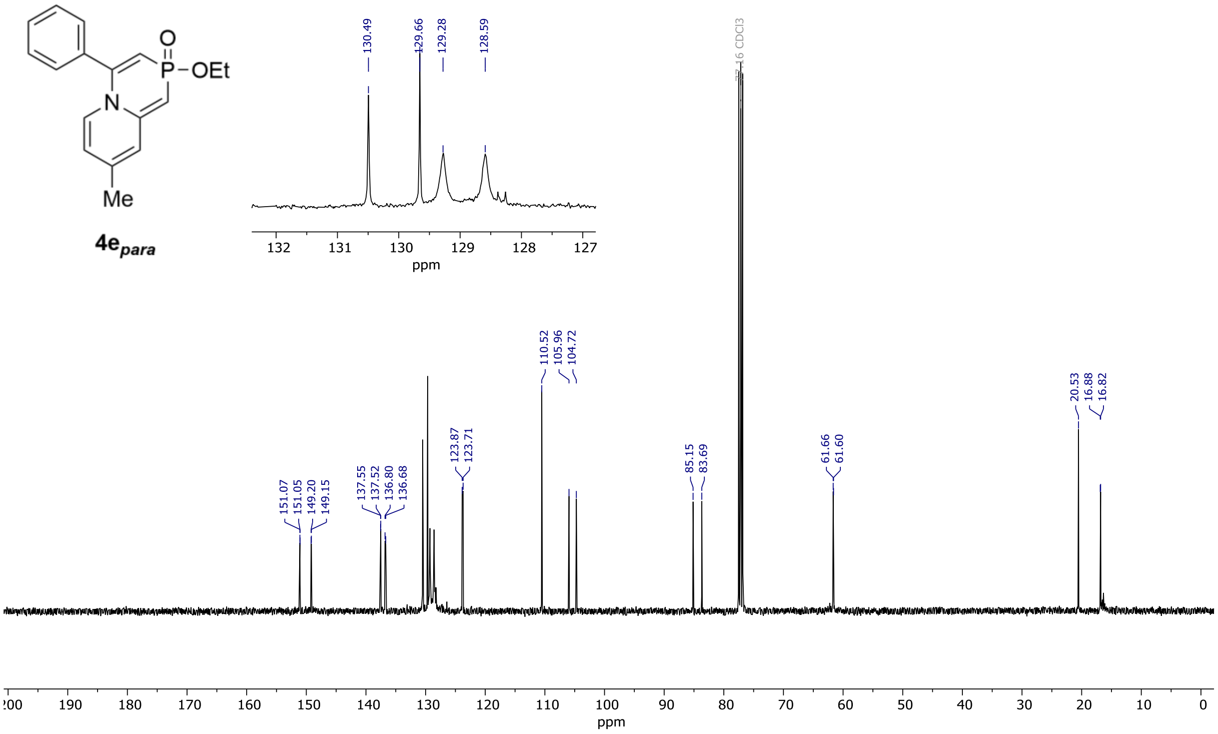

Figure S229 ^{13}C $\{^1H\}$ *NMR spectrum of* $\textbf{4e}_{\textbf{para}}$ *(101 MHz, $CDCl_3$).*

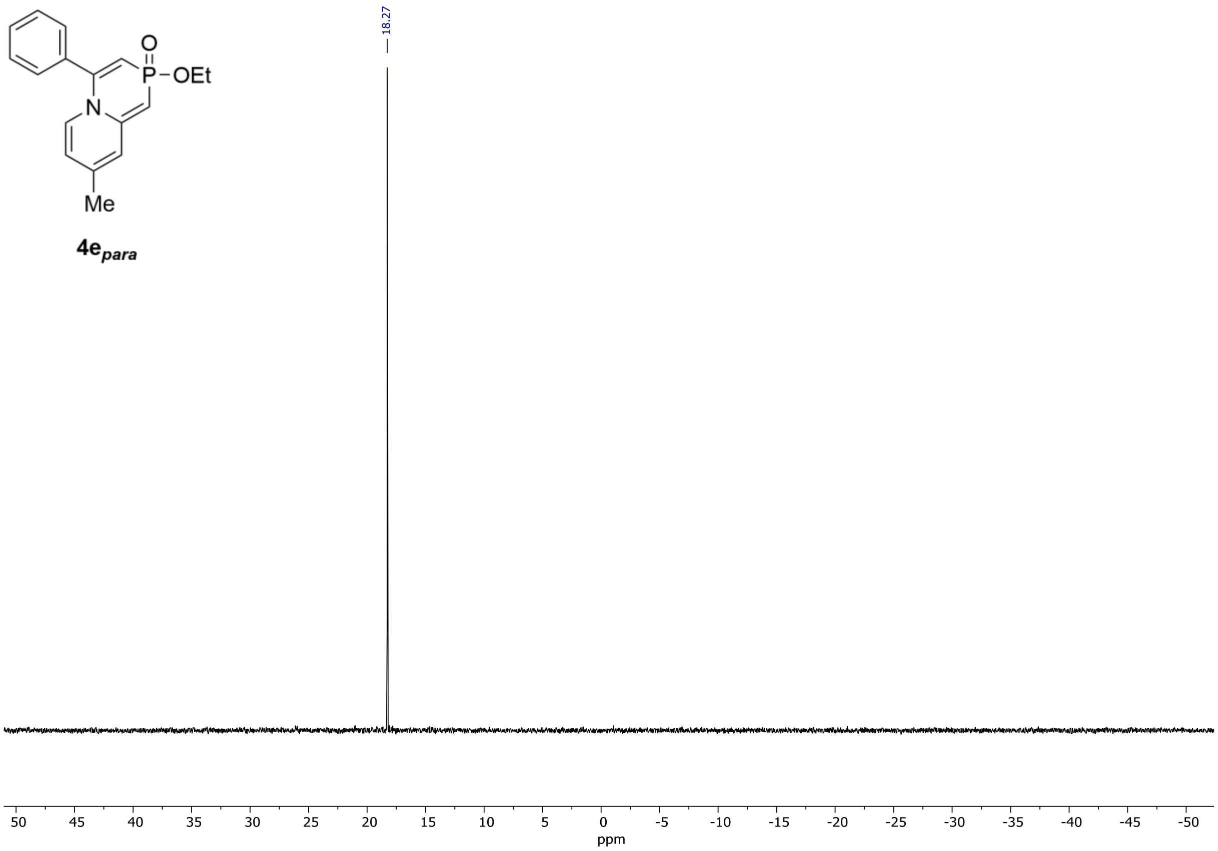

Figure S230 ^{31}P $\{^1H\}$ *NMR spectrum of* $\textbf{4e}_{\textbf{para}}$ *(162 MHz, $CDCl_3$).*

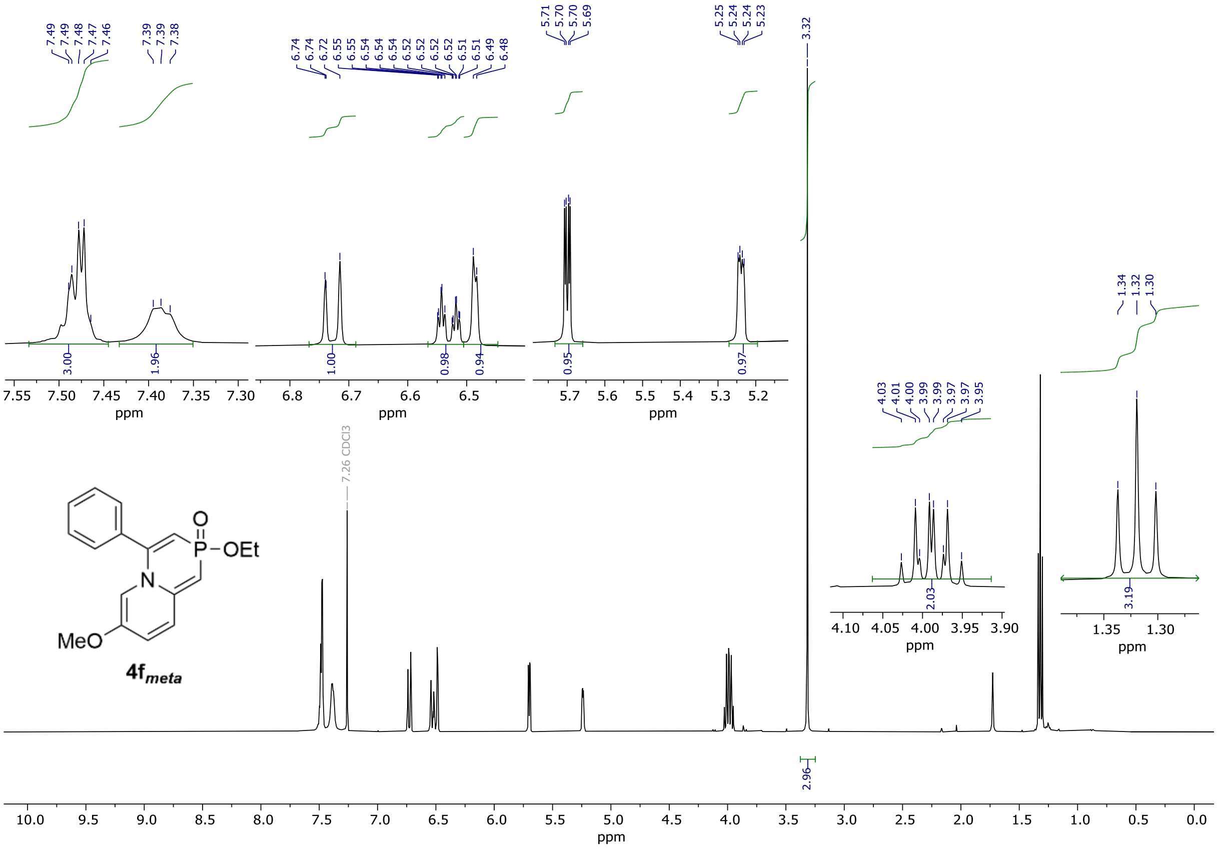

Figure S231 *^{1}H NMR spectrum of **4f$_{meta}$** (400 MHz, CDCl$_3$).*

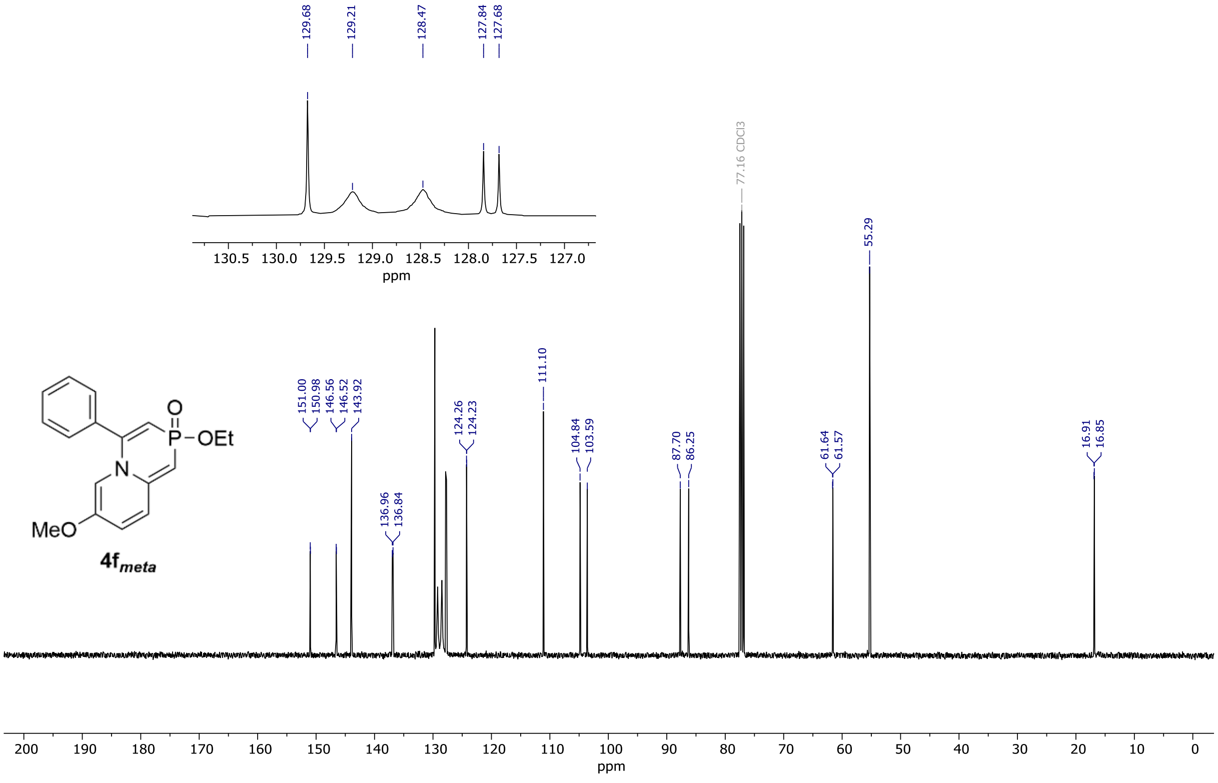

Figure S232 *^{13}C {^{1}H} NMR spectrum of **4f$_{meta}$** (101 MHz, CDCl$_3$).*

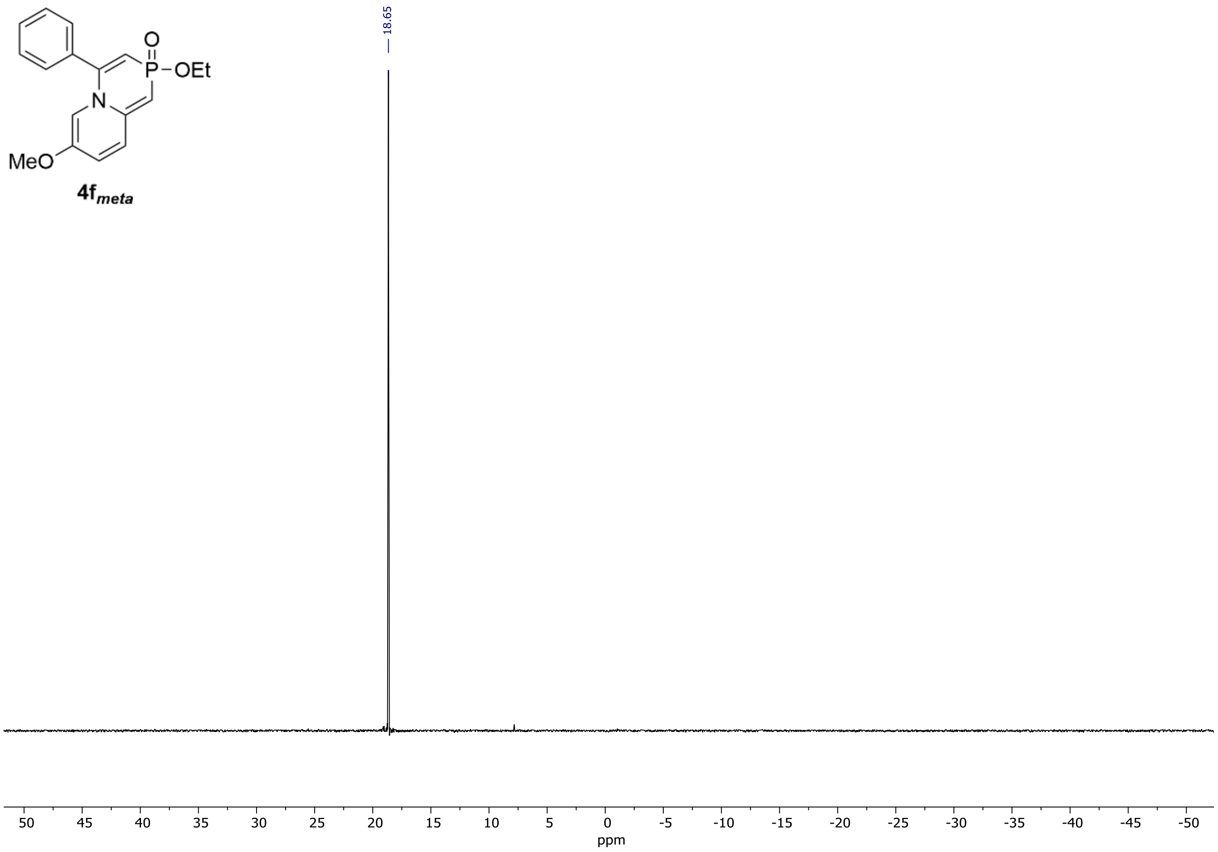

Figure S233 ^{31}P {^{1}H} NMR spectrum of **4f$_{meta}$** (162 MHz, CDCl$_3$).

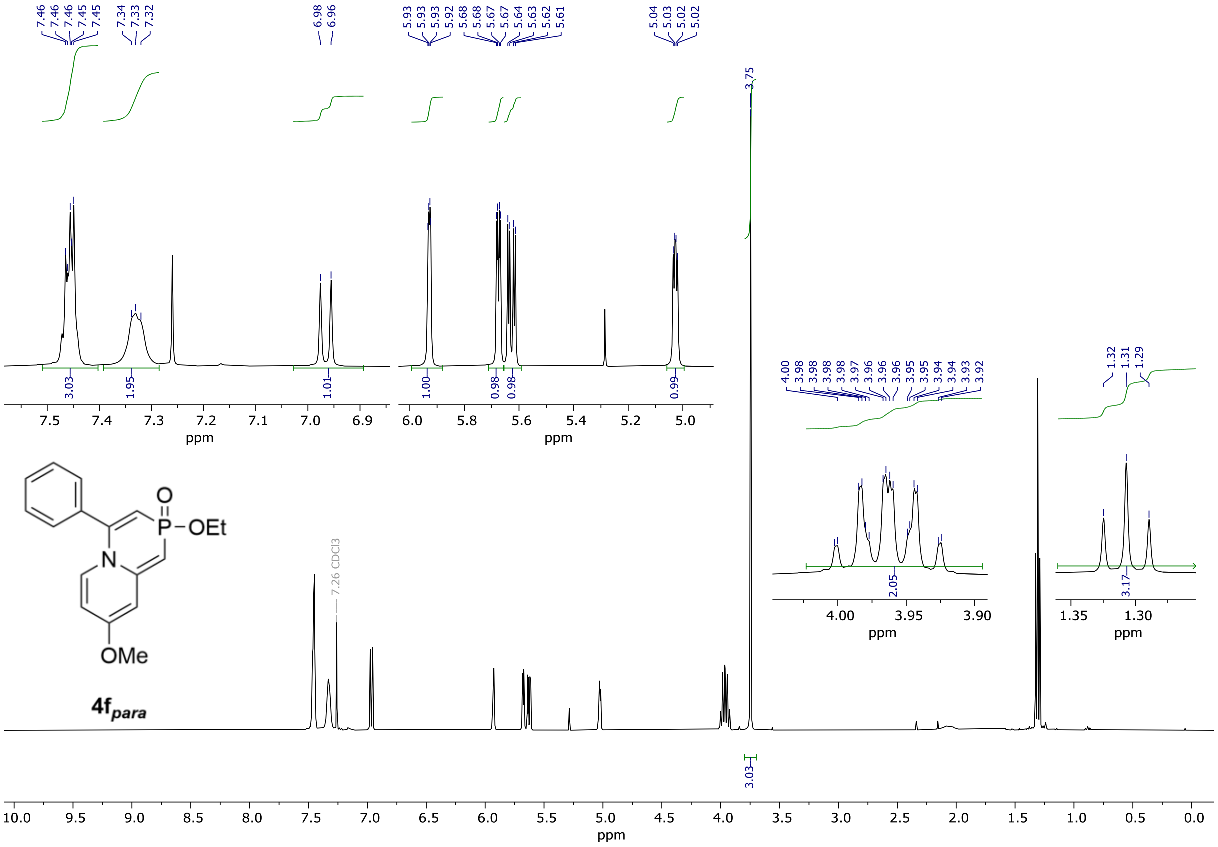

Figure S234 ^{1}H NMR spectrum of **4f$_{para}$** (400 MHz, CDCl$_3$).

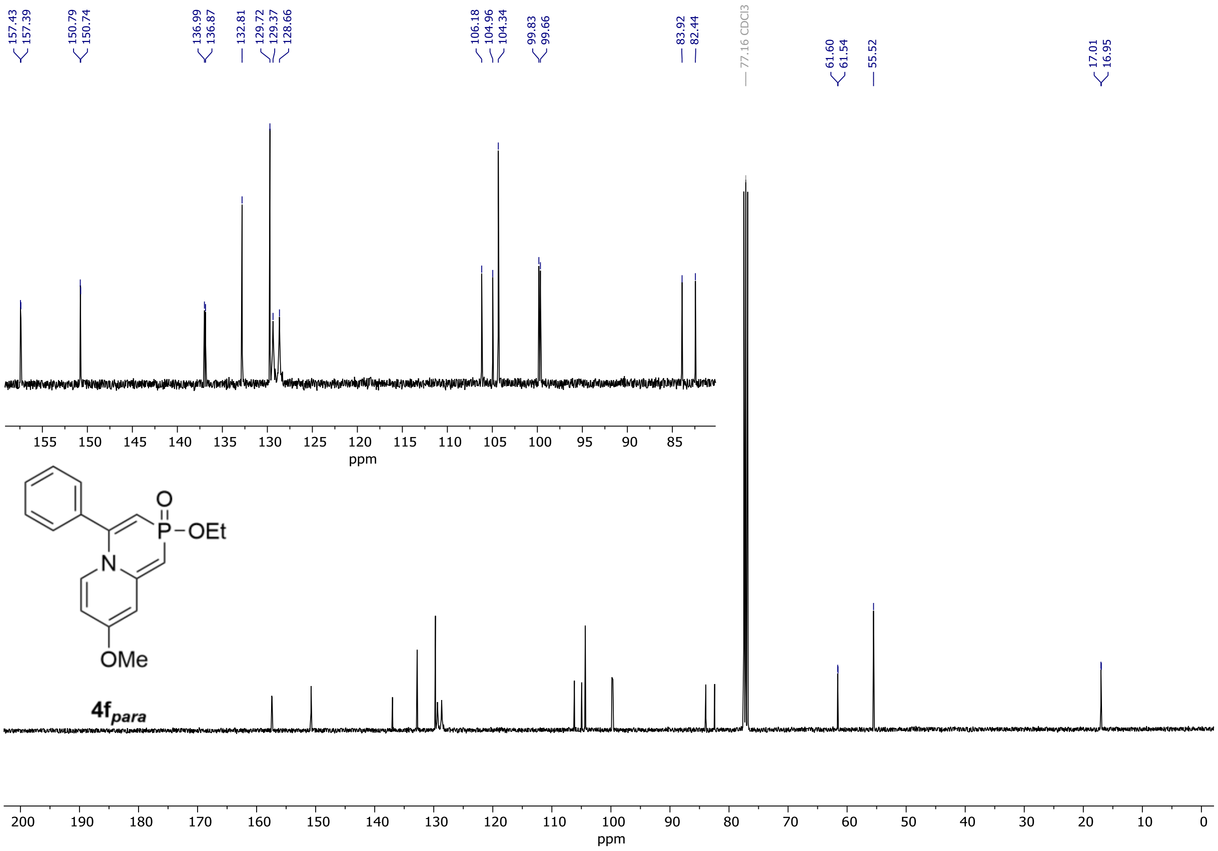

Figure S235 *^{13}C {^{1}H} NMR spectrum of **4f$_{para}$** (101 MHz, $CDCl_3$).*

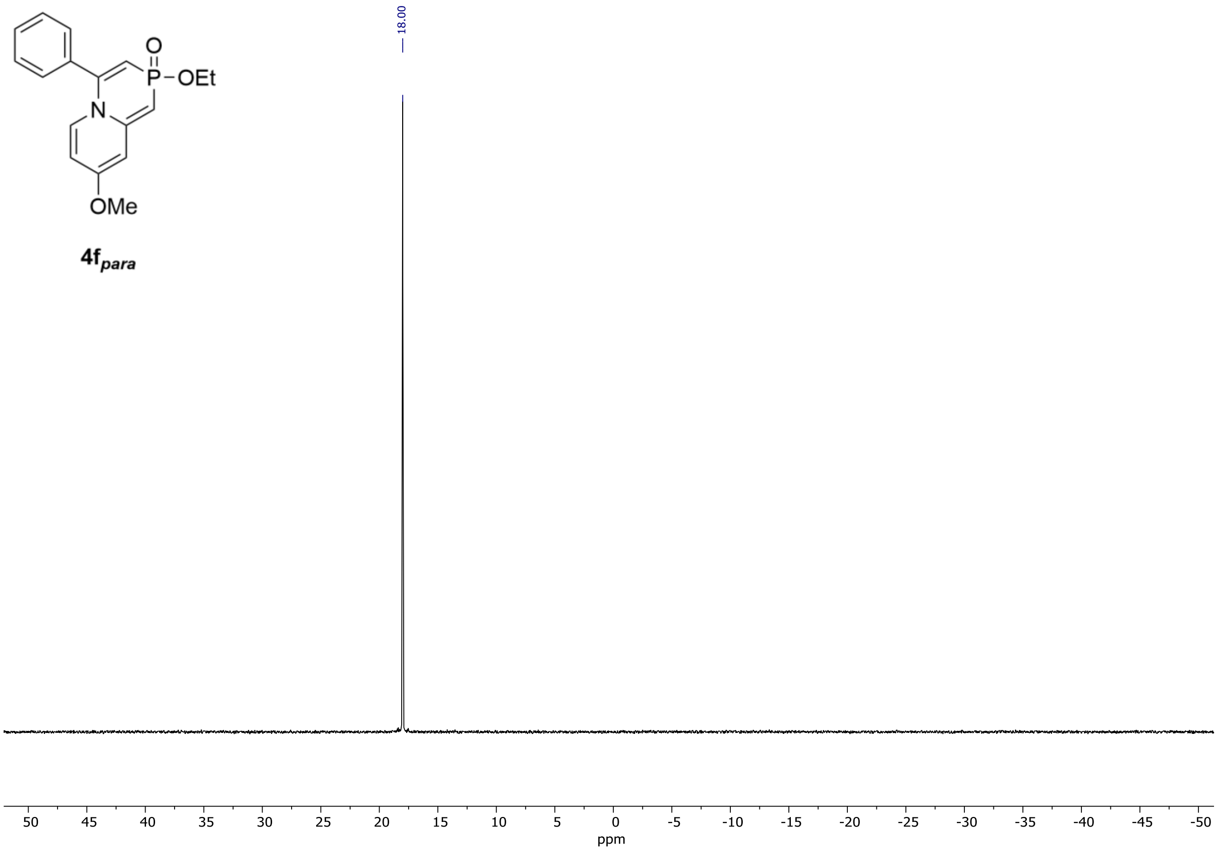

Figure S236 *^{31}P {^{1}H} NMR spectrum of **4f$_{para}$** (162 MHz, $CDCl_3$).*

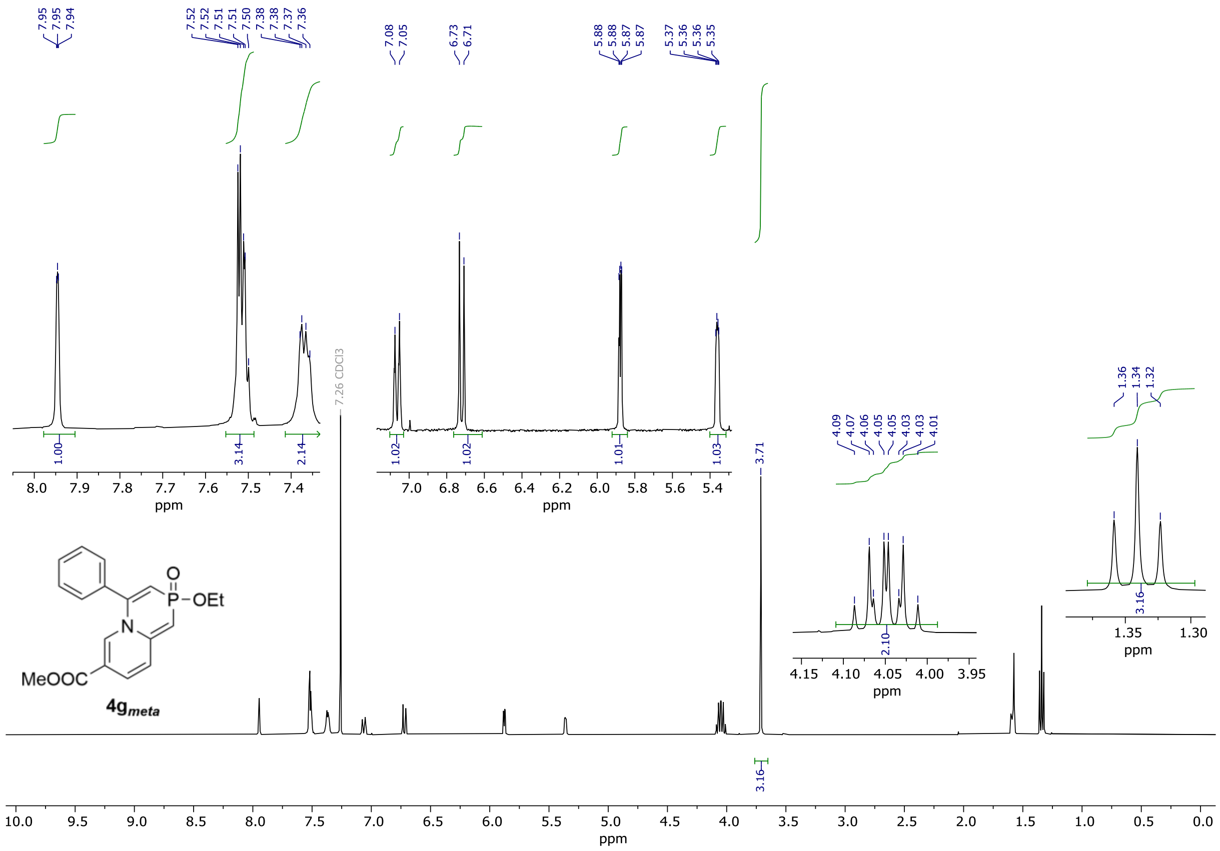

Figure S237 ^{1}H NMR spectrum of $\mathbf{4g_{meta}}$ (400 MHz, $CDCl_3$).

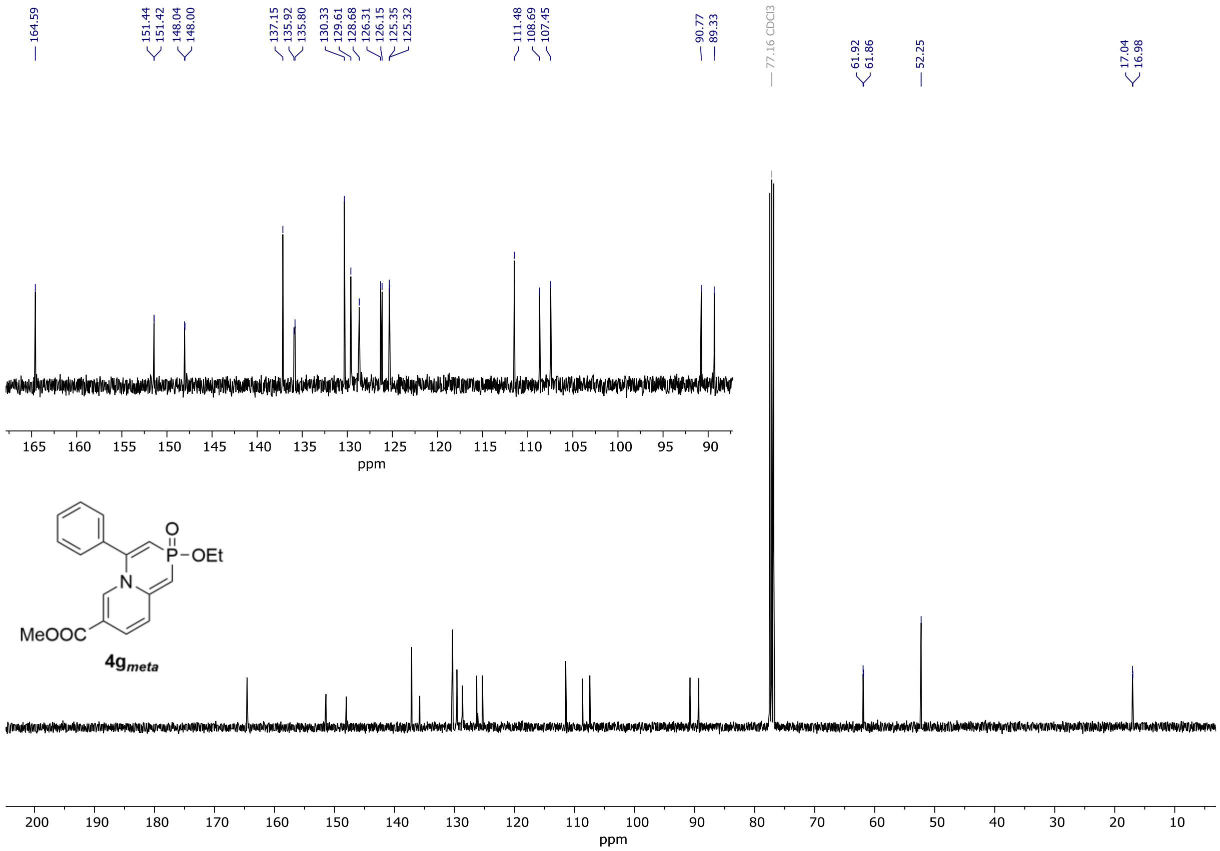

Figure S238 ^{13}C {^{1}H} NMR spectrum of $\mathbf{4g_{meta}}$ (101 MHz, $CDCl_3$).

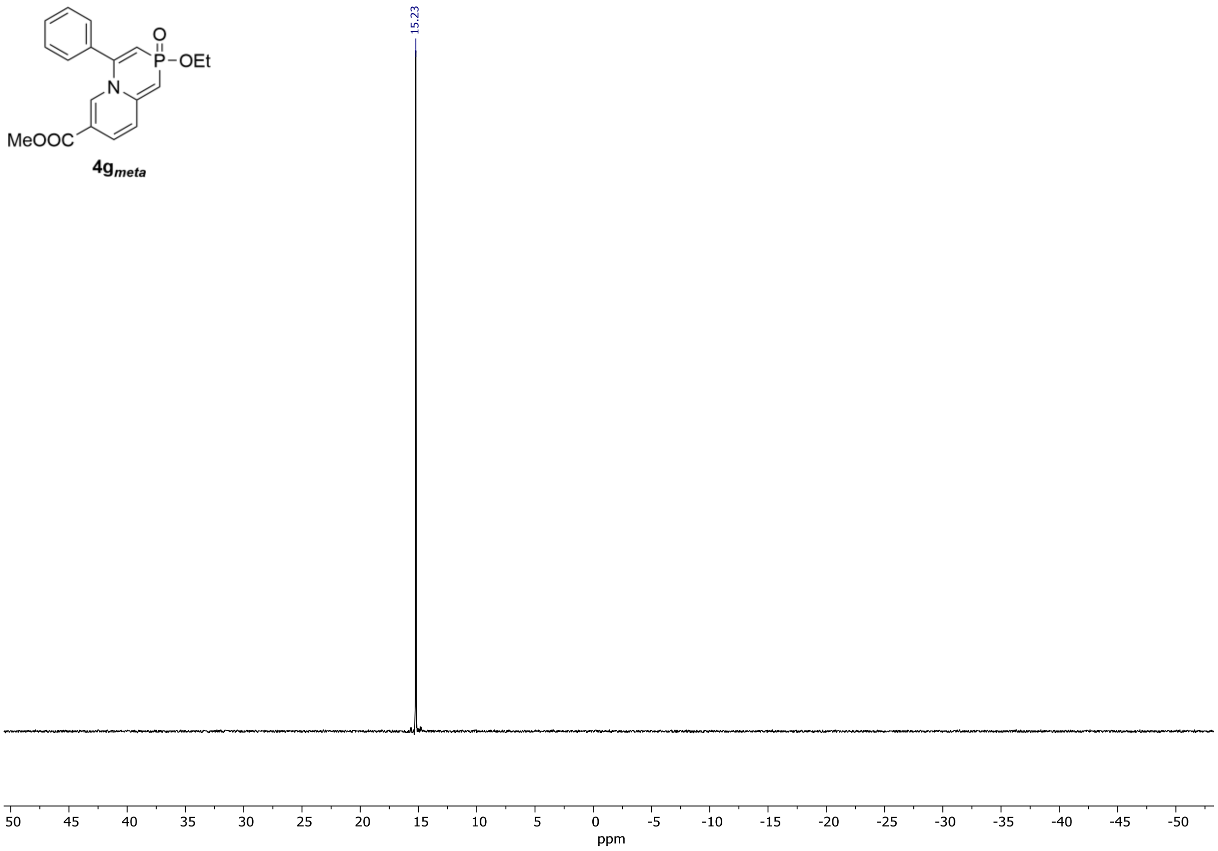

Figure S239 $^{31}P\ \{^1H\}$ *NMR spectrum of* ***4g$_{meta}$*** *(162 MHz, $CDCl_3$).*

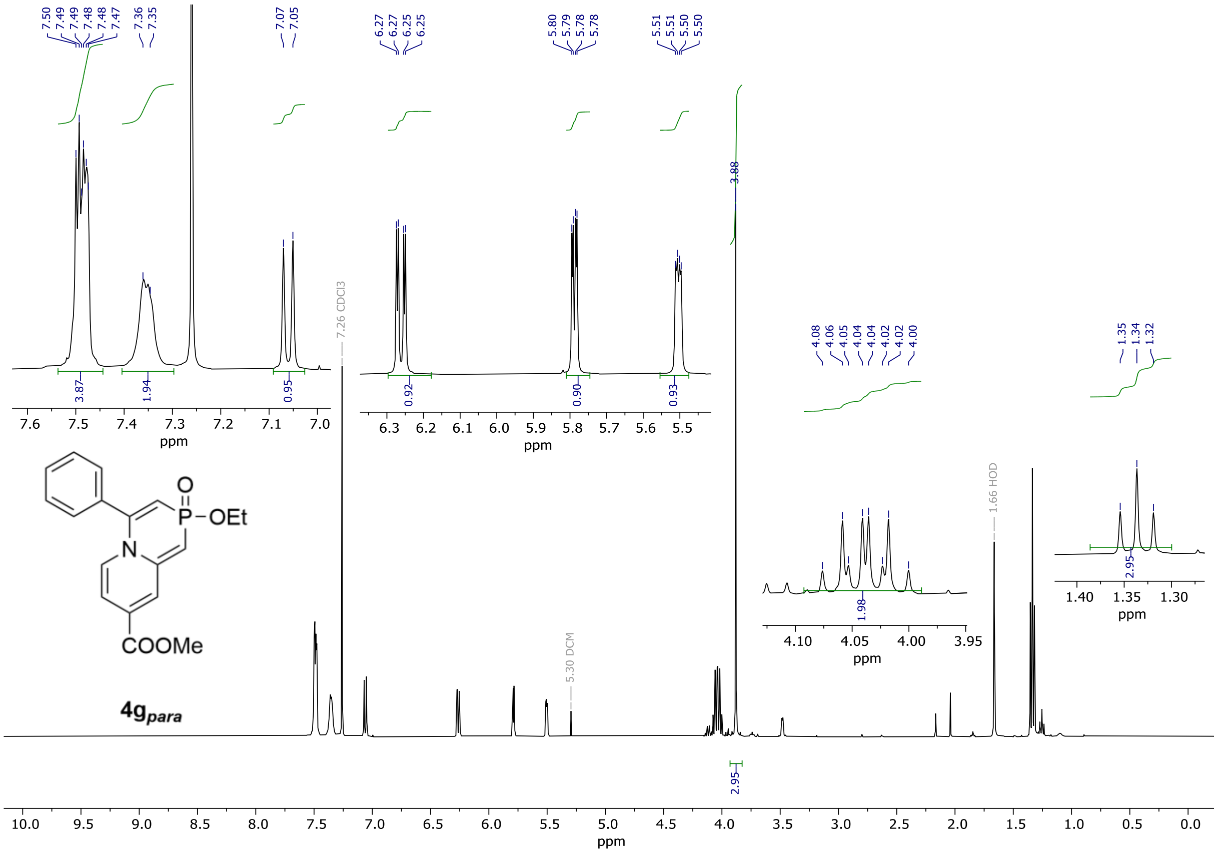

Figure S240 ^1H *NMR spectrum of* ***4g$_{para}$*** *(400 MHz, $CDCl_3$).*

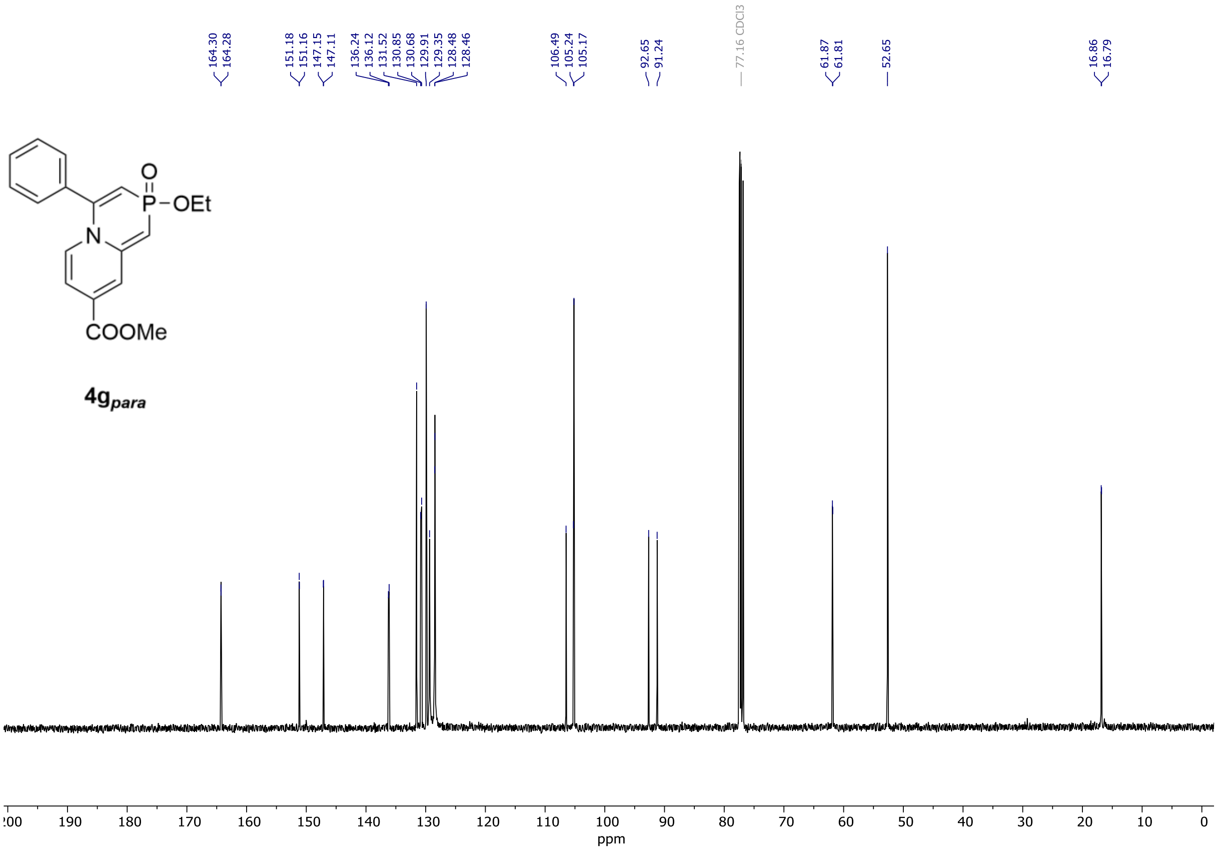

Figure S241 ^{13}C {^{1}H} *NMR spectrum of* $\mathbf{4g}_{\mathit{para}}$ *(101 MHz, CDCl*$_3$*).*

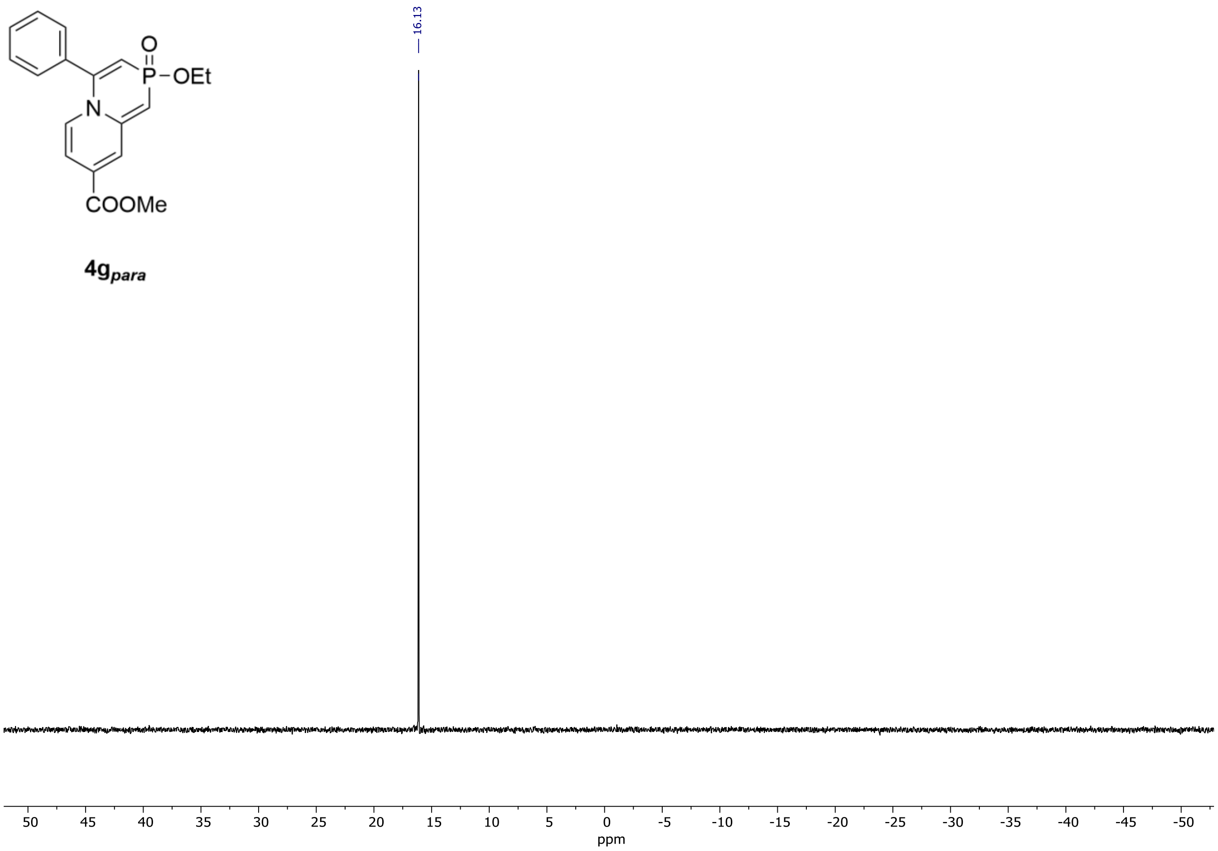

Figure S242 ^{31}P {^{1}H} *NMR spectrum of* $\mathbf{4g}_{\mathit{para}}$ *(162 MHz, CDCl*$_3$*).*

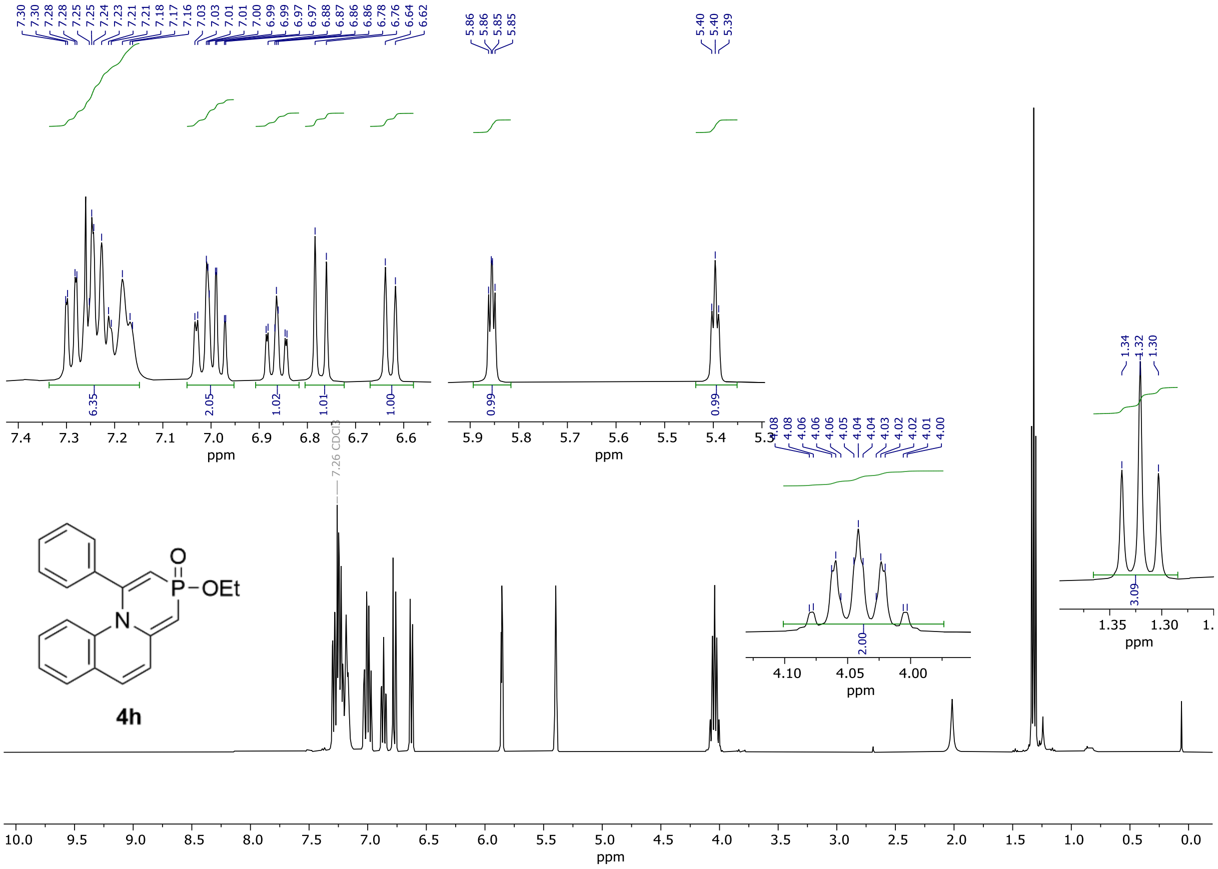

Figure S243 *¹H NMR spectrum of **4h** (400 MHz, CDCl₃).*

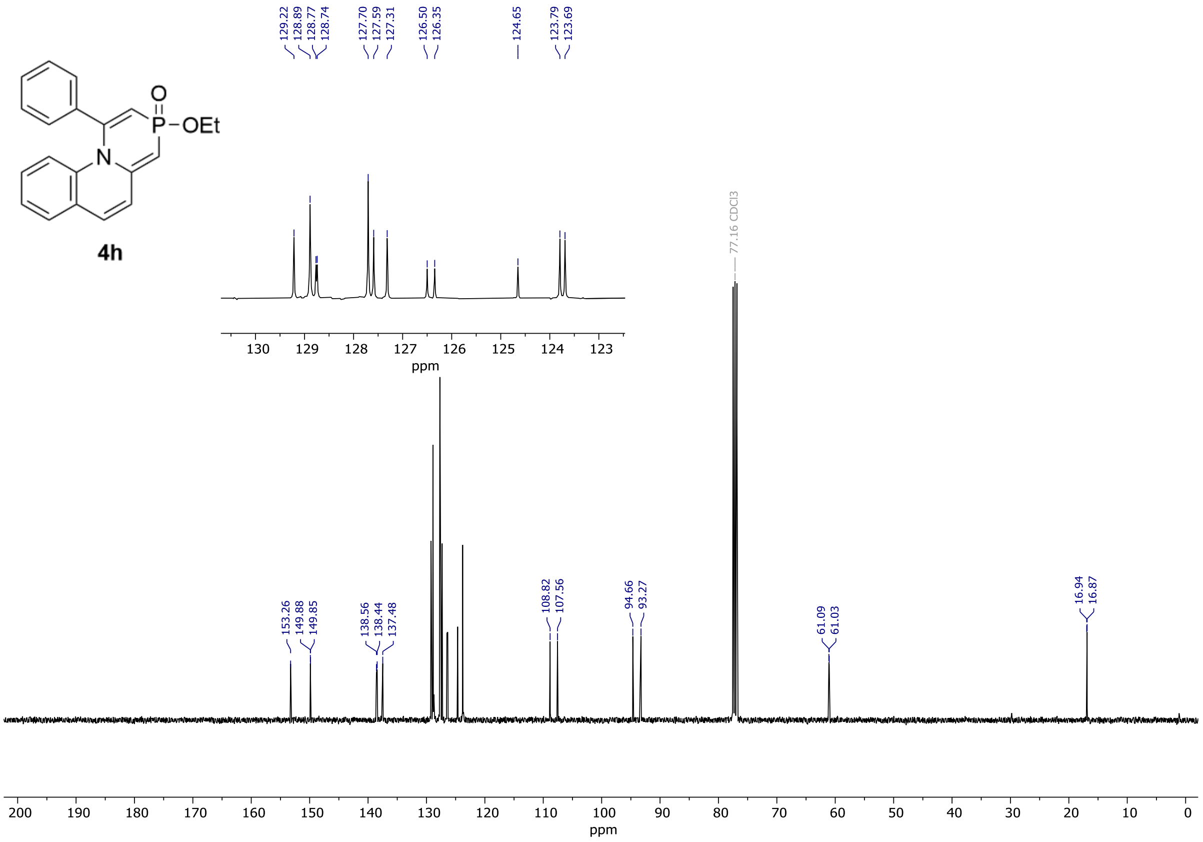

Figure S244 *¹³C {¹H} NMR spectrum of **4h** (101 MHz, CDCl₃).*

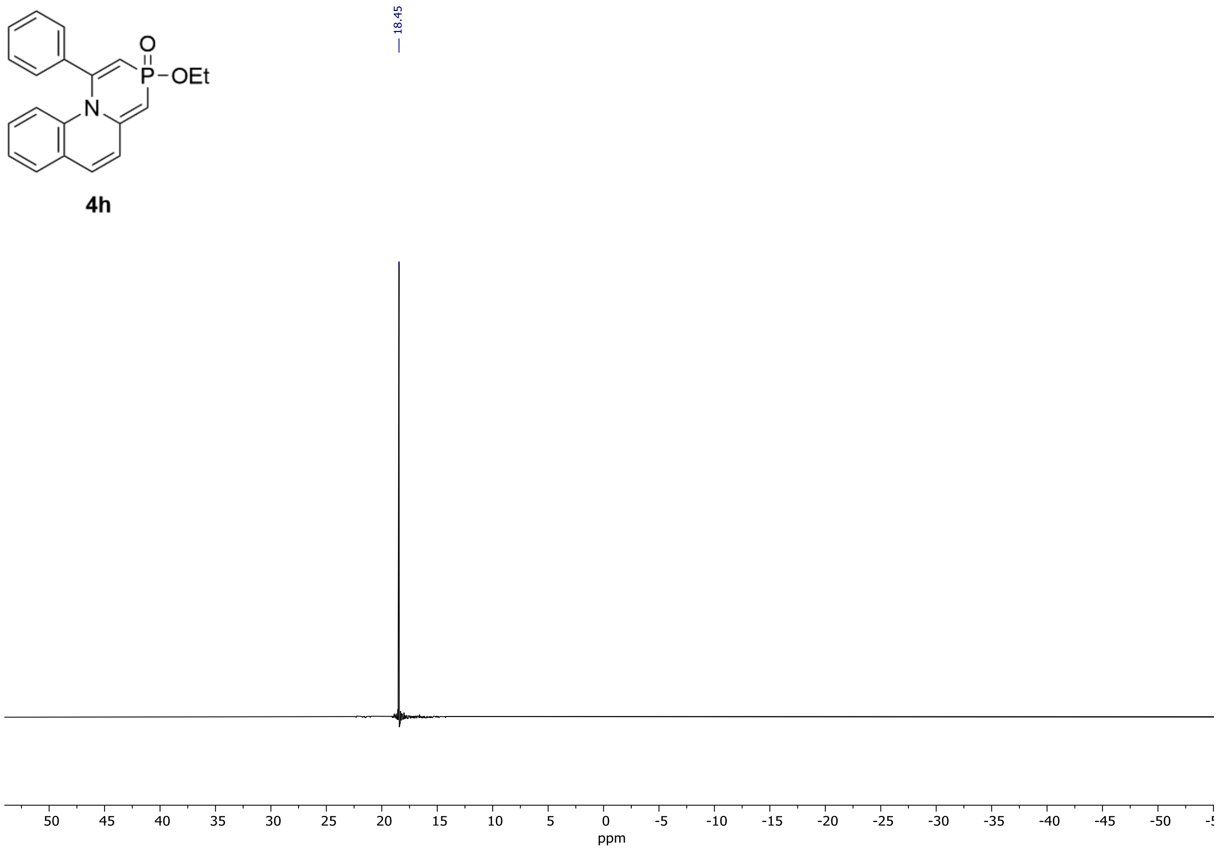

Figure S245 $^{31}P\ \{^{1}H\}$ NMR spectrum of **4h** (162 MHz, $CDCl_3$).

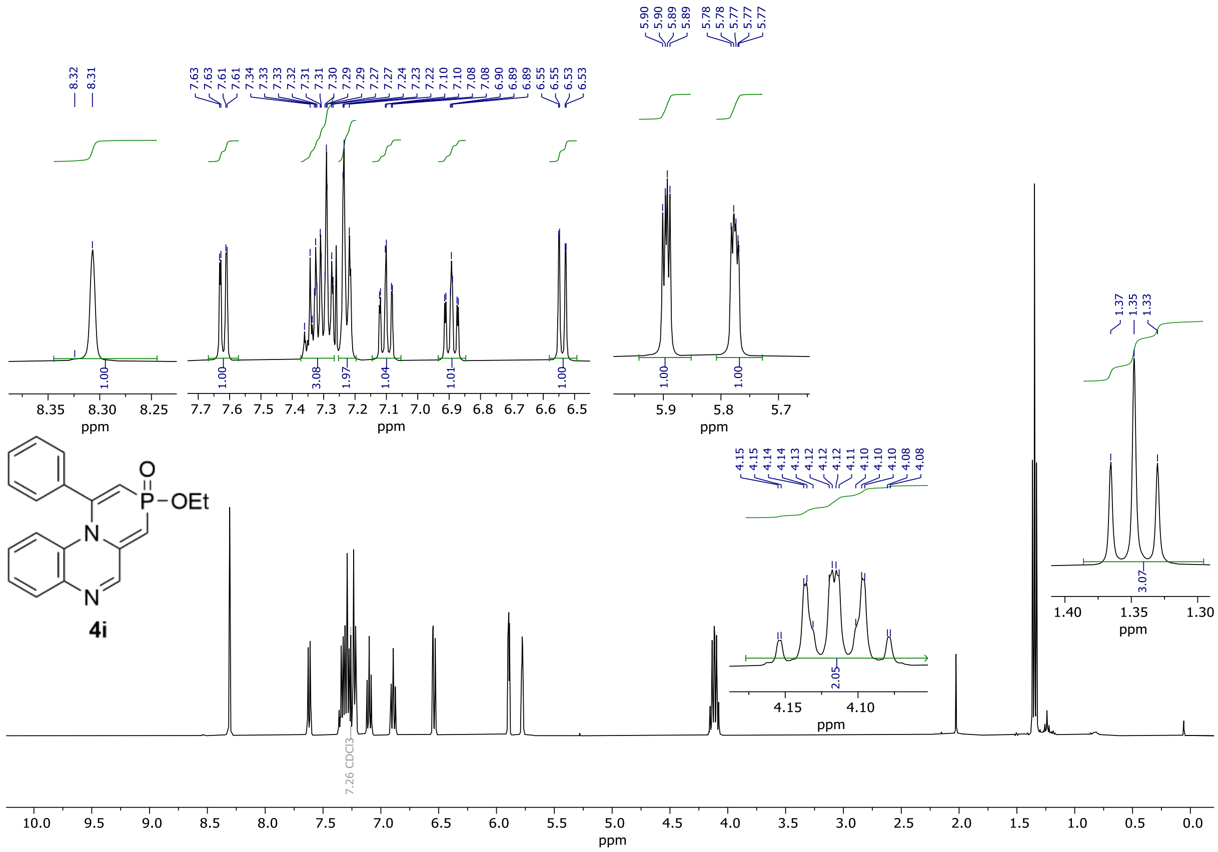

Figure S246 ^{1}H NMR spectrum of **4i** (400 MHz, $CDCl_3$).

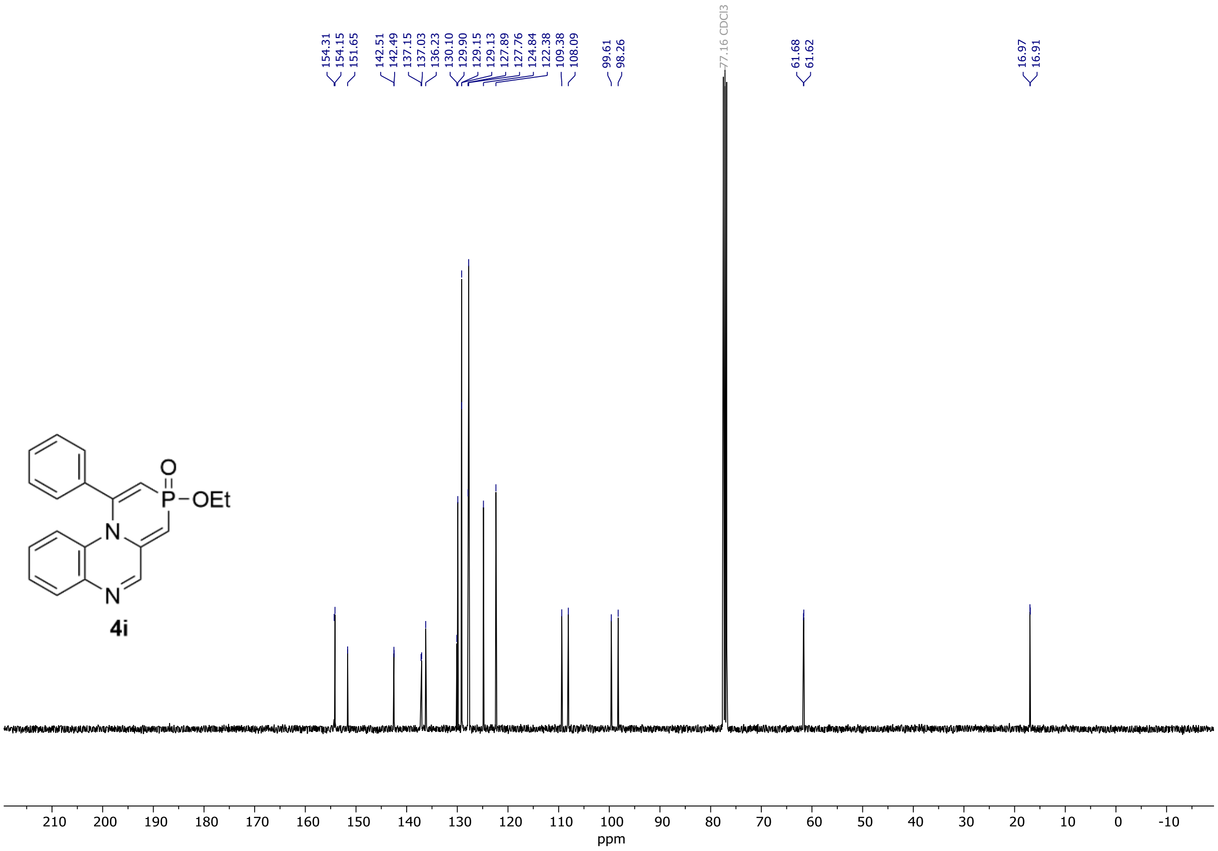

Figure S247 *^{13}C {^{1}H} NMR spectrum of **4i** (101 MHz, $CDCl_3$).*

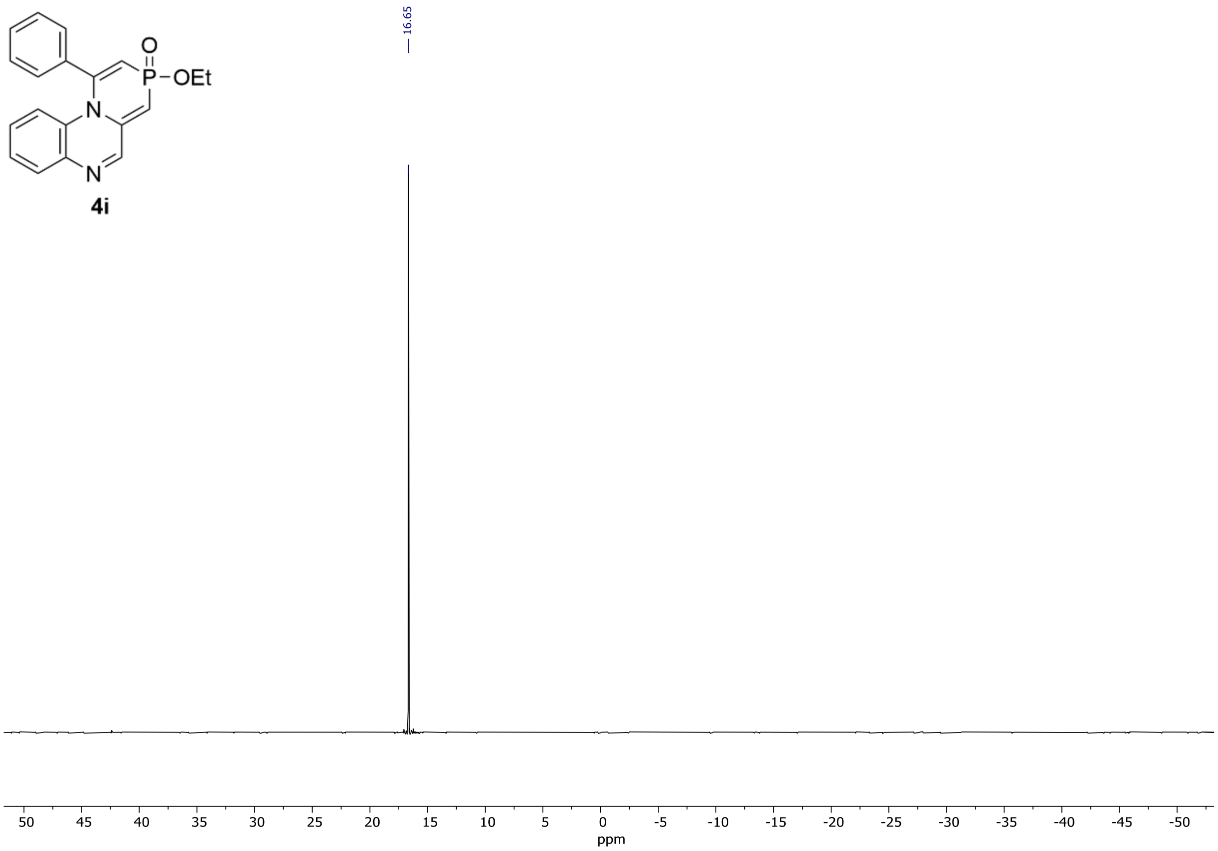

Figure S248 *^{31}P {^{1}H} NMR spectrum of **4i** (162 MHz, $CDCl_3$).*

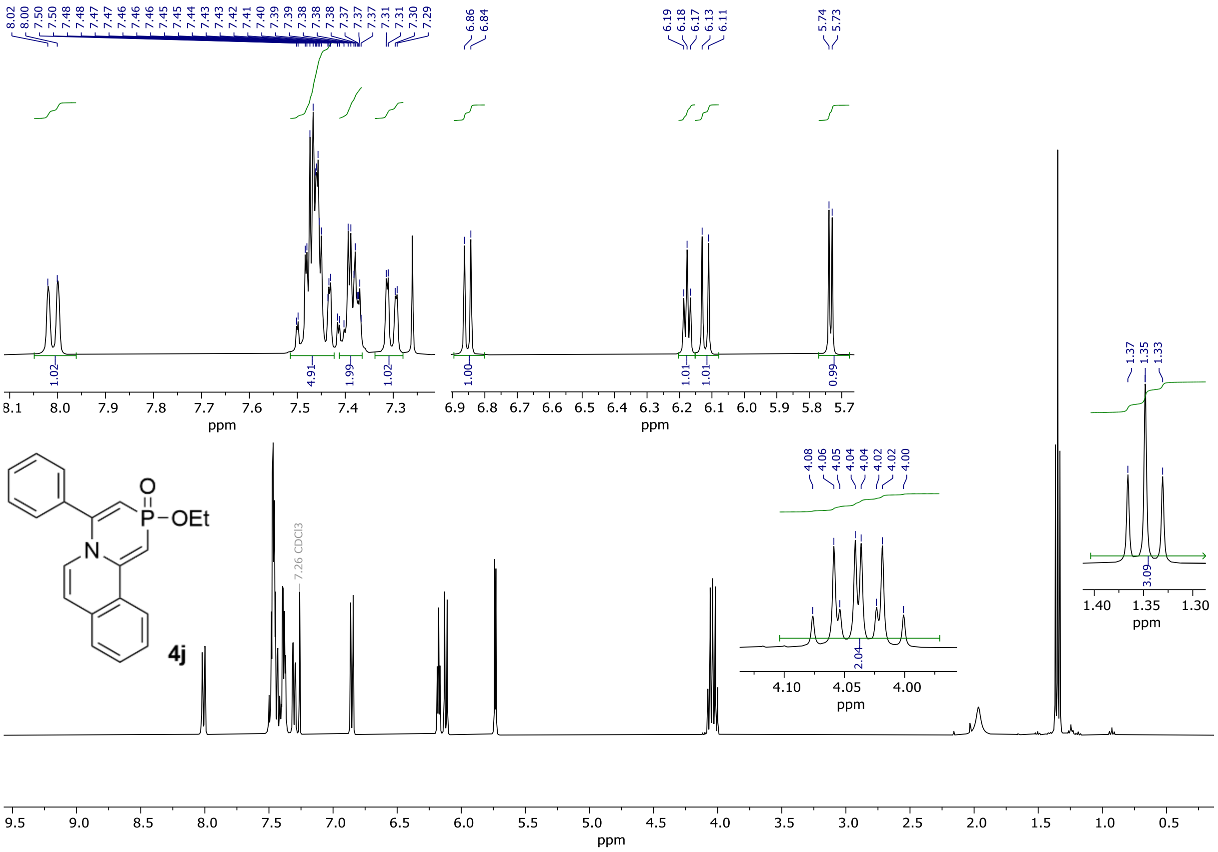

Figure S249 *^{1}H NMR spectrum of **4j** (400 MHz, CDCl$_3$).*

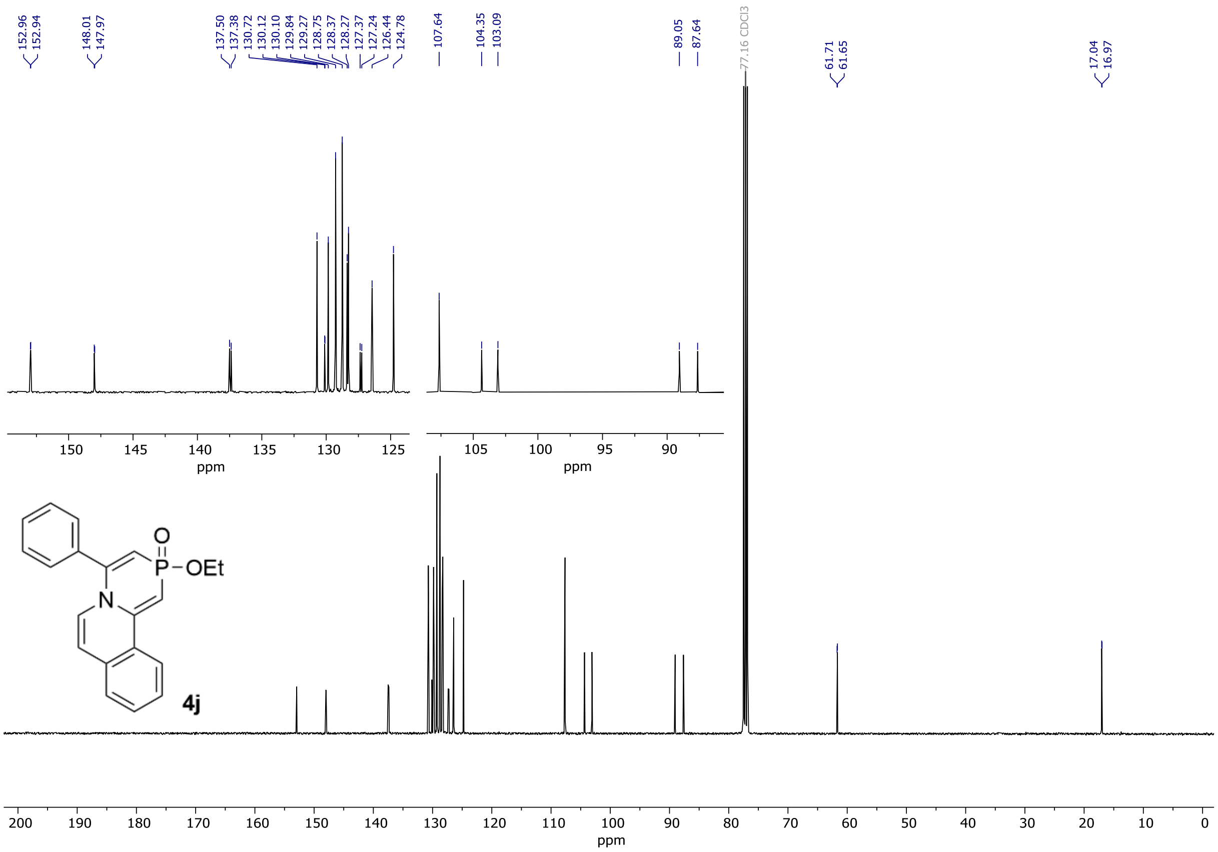

Figure S250 *^{13}C {^{1}H} NMR spectrum of **4j** (101 MHz, CDCl$_3$).*

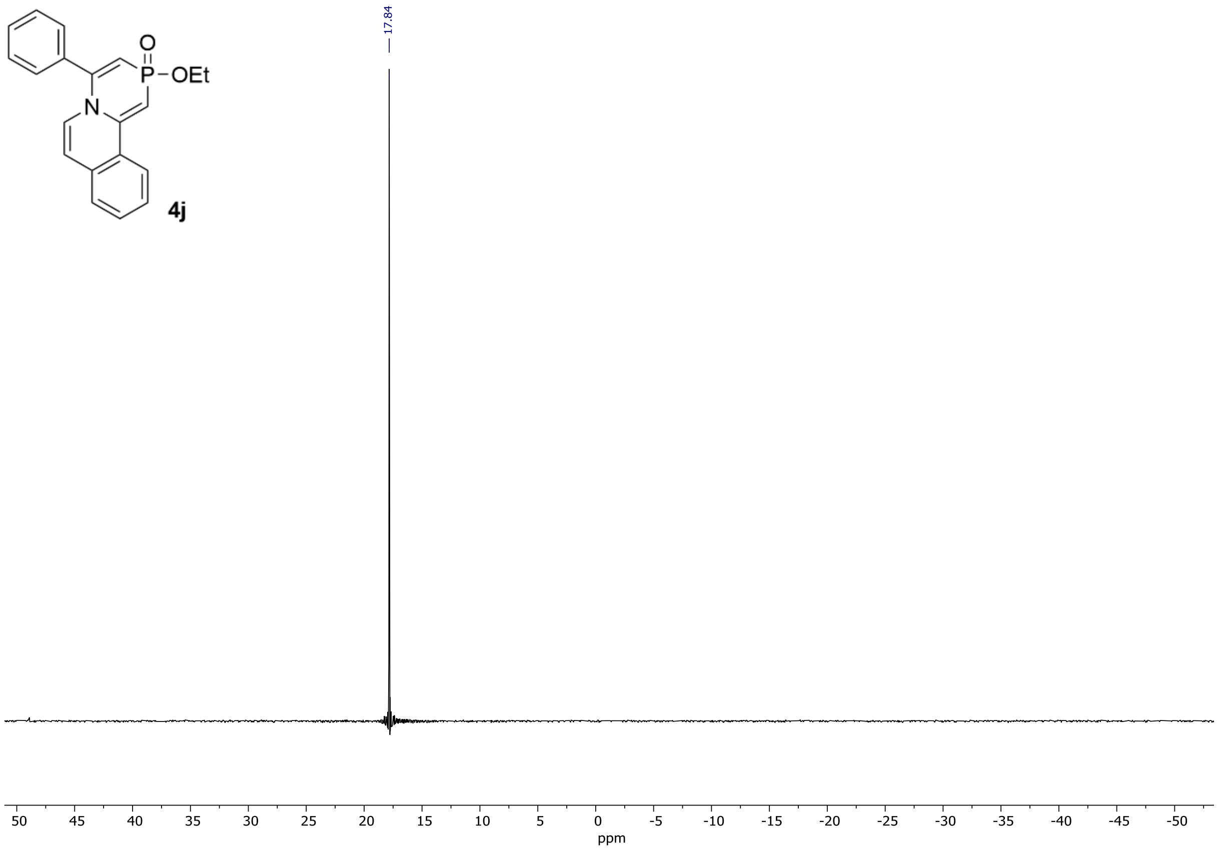

Figure S251 $^{31}P\ \{^1H\}$ *NMR spectrum of* ***4j*** *(162 MHz, $CDCl_3$).*

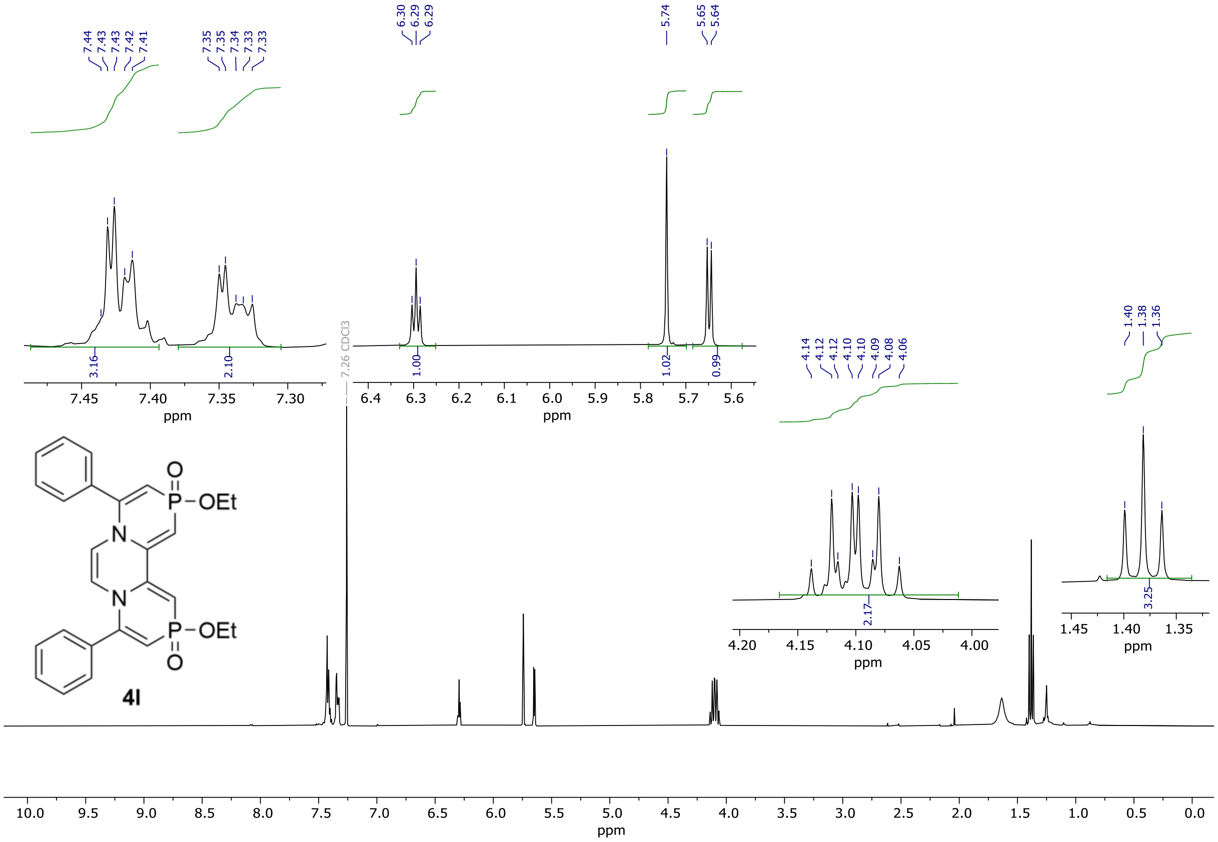

Figure S252 ^1H *NMR spectrum of* ***4l*** *(400 MHz, $CDCl_3$).*

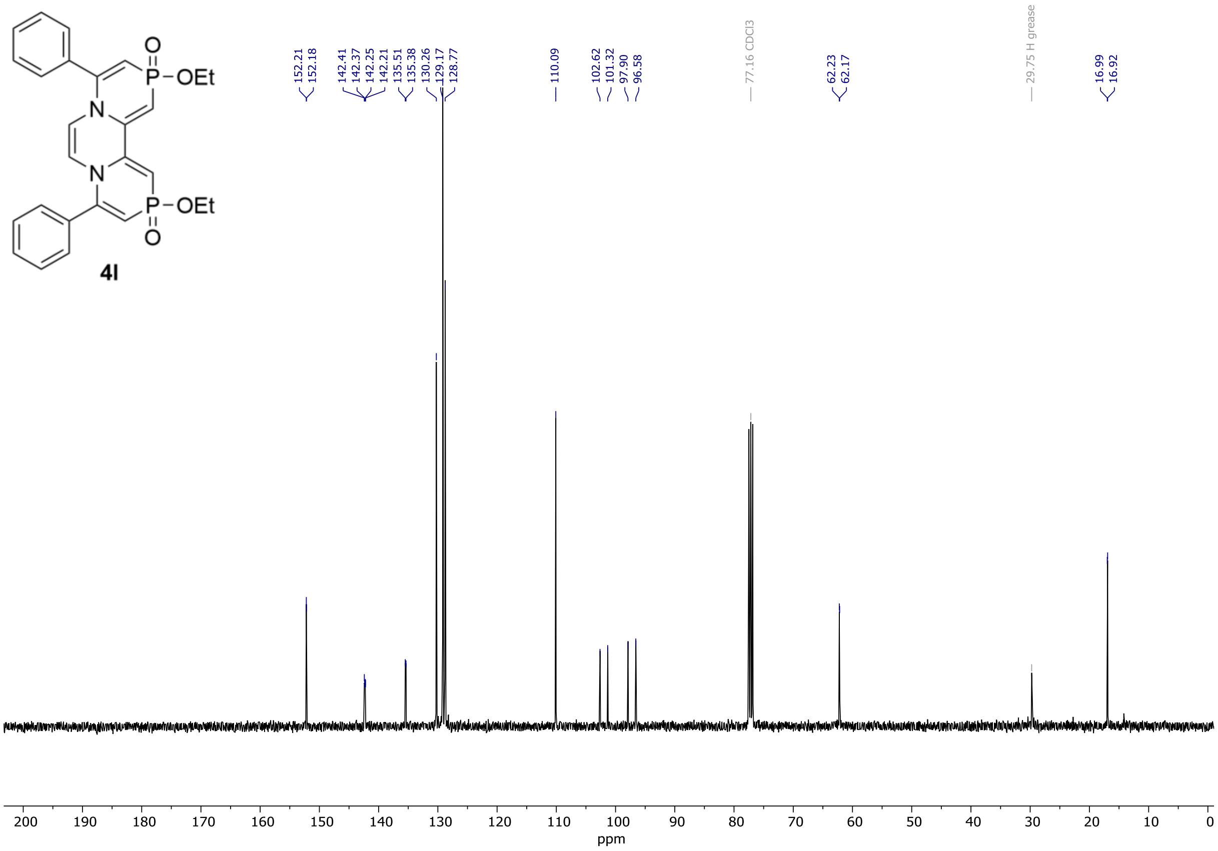

Figure S253 ^{13}C {^{1}H} *NMR spectrum of **4l** (101 MHz, $CDCl_3$).*

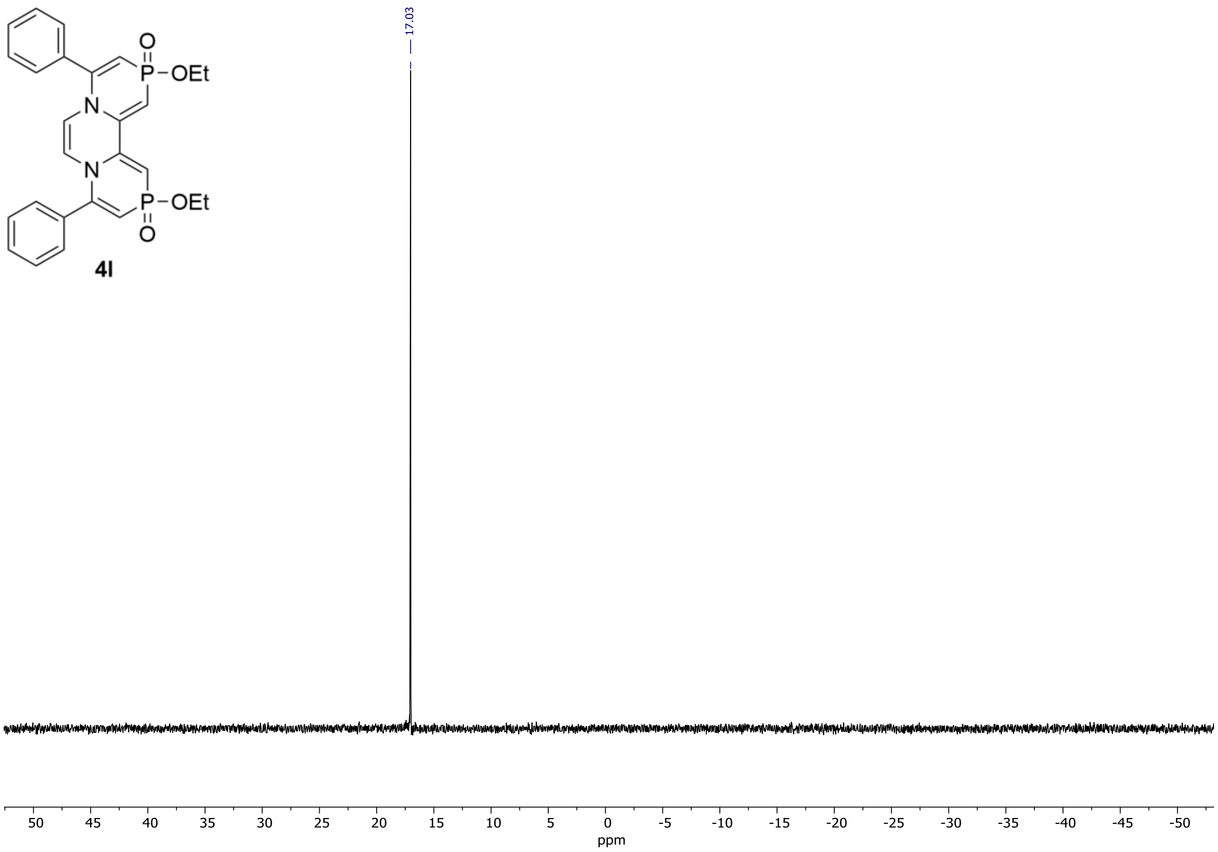

Figure S254 ^{31}P {^{1}H} *NMR spectrum of **4l** (162 MHz, $CDCl_3$).*

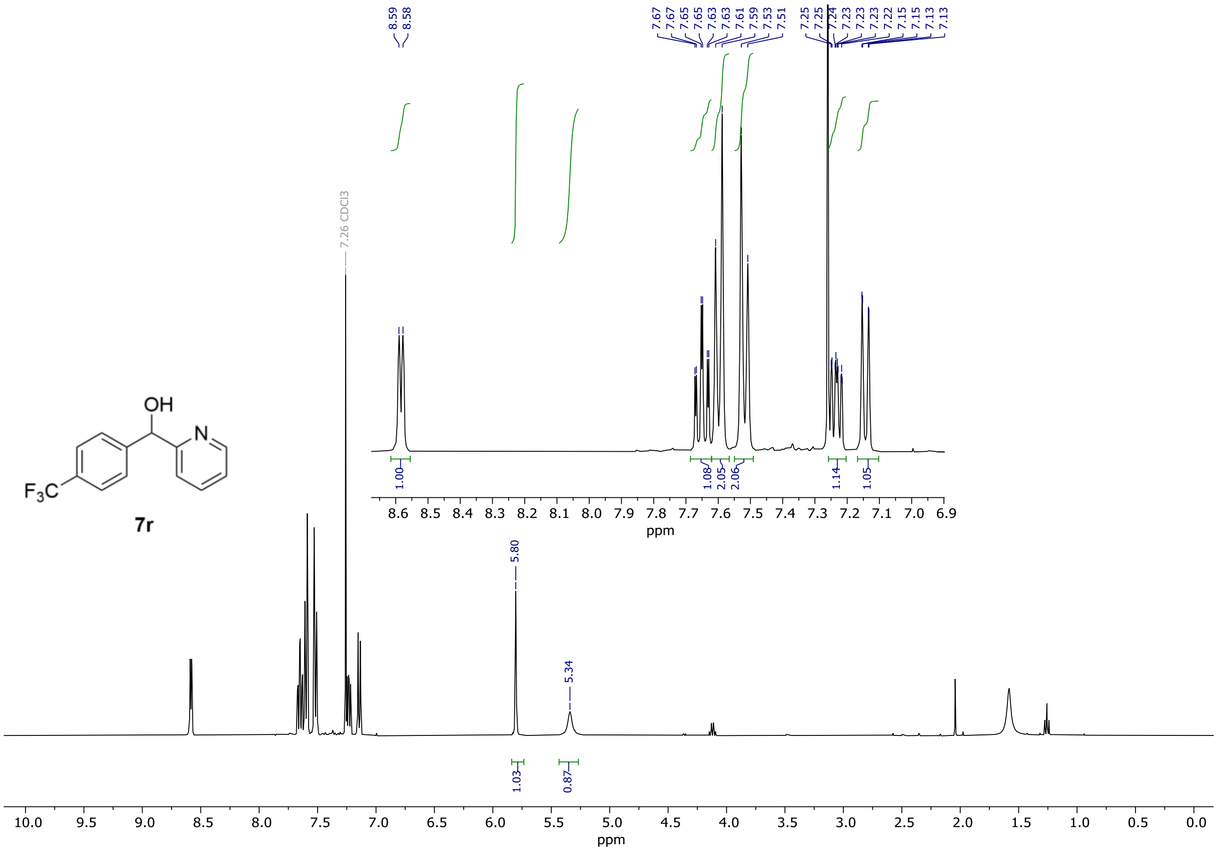

Figure S255 ^{1}H NMR spectrum of **7r** (400 MHz, $CDCl_3$).

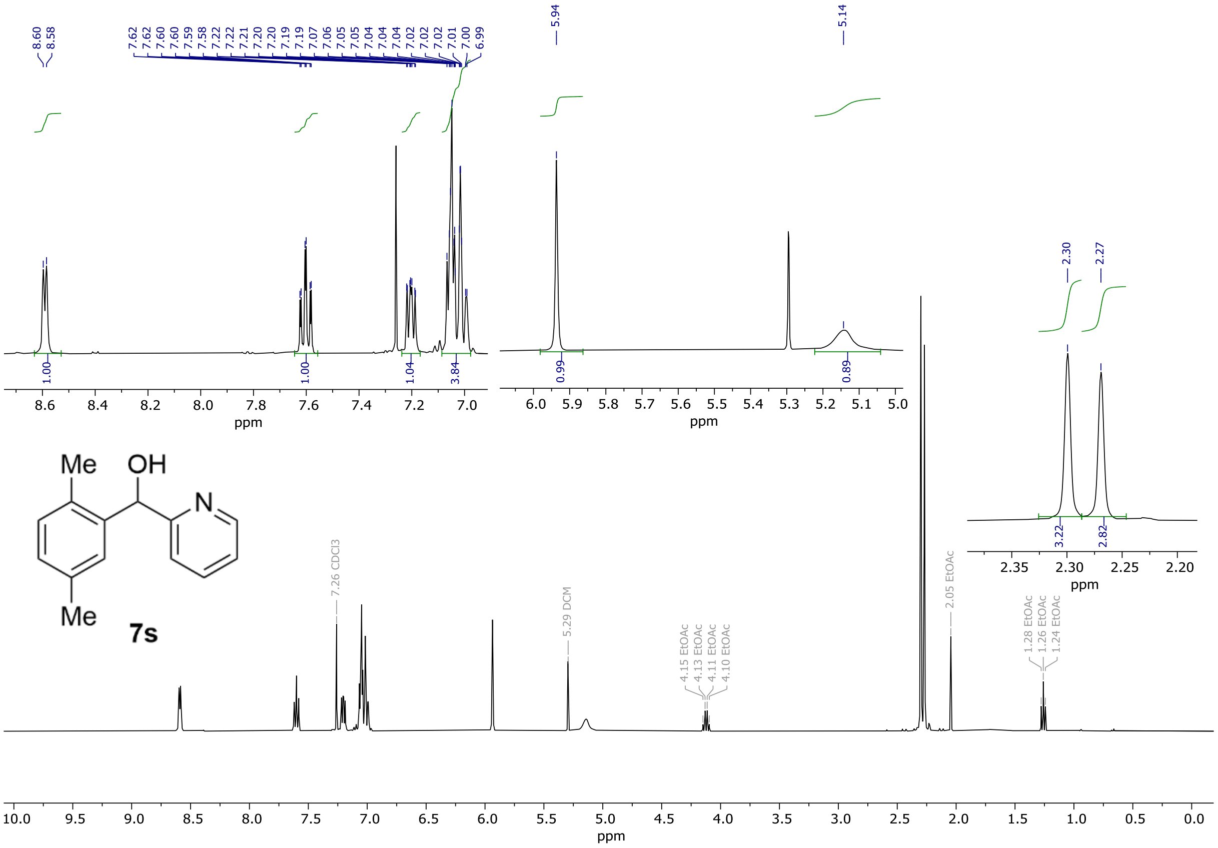

Figure S256 ^{1}H NMR spectrum of **7s** (400 MHz, $CDCl_3$).

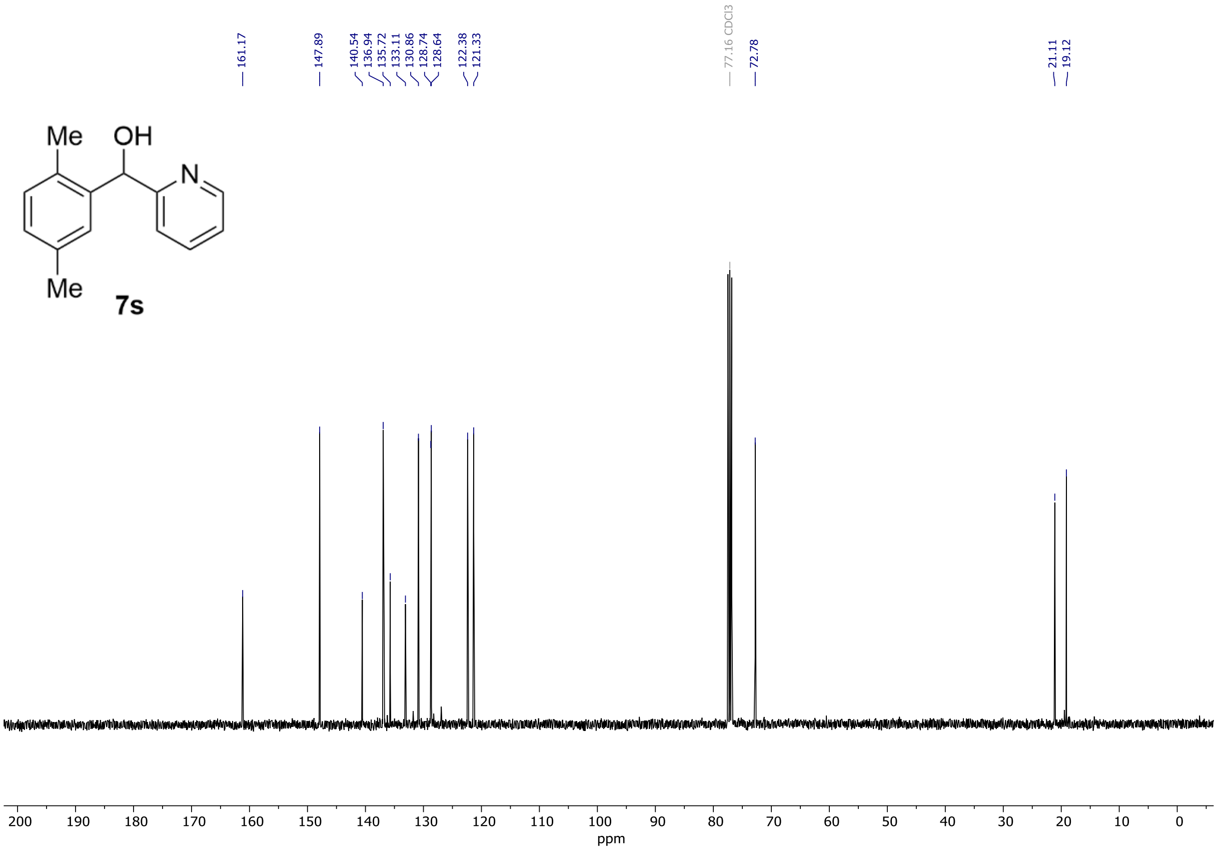

Figure S257 *^{13}C {^{1}H} NMR spectrum of **7s** (101 MHz, $CDCl_3$).*

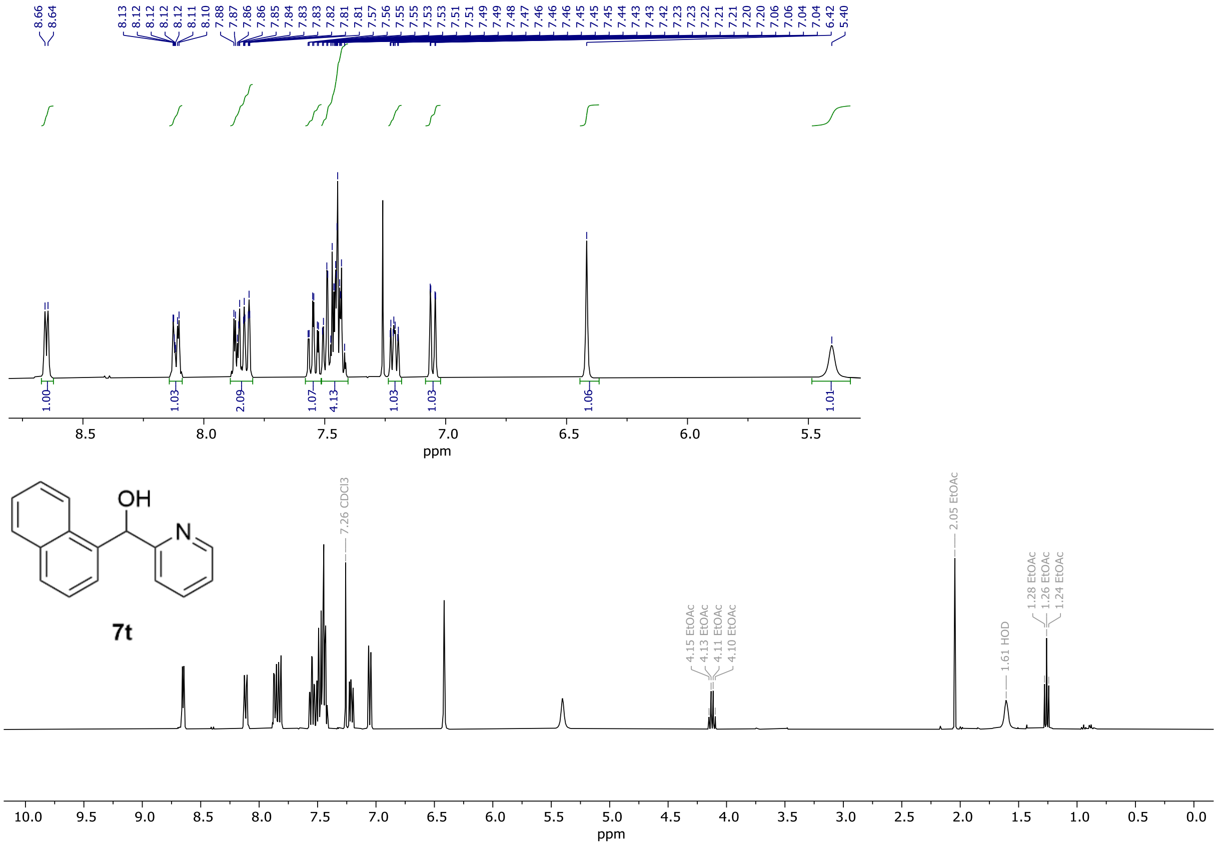

Figure 258 *^{1}H NMR spectrum of **7t** (400 MHz, $CDCl_3$).*

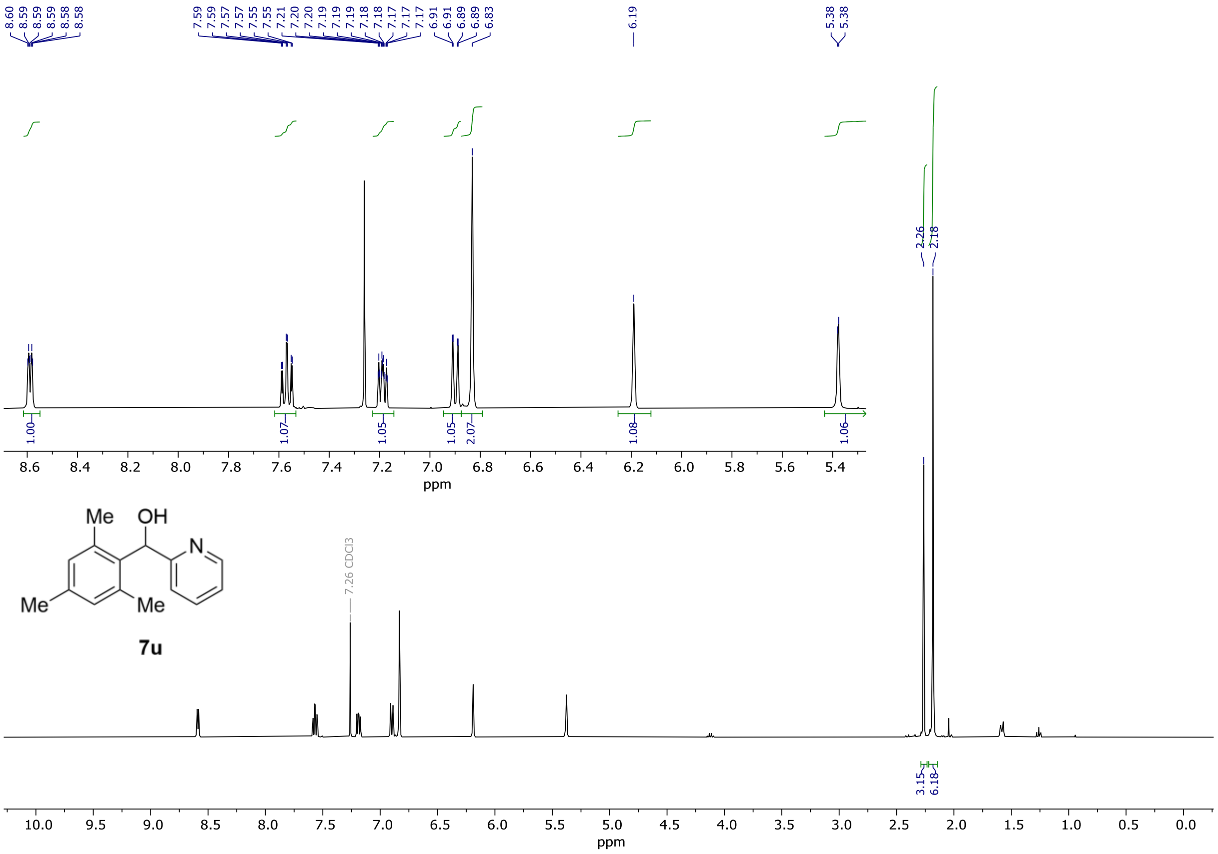

Figure S259 ^{1}H NMR spectrum of **7u** (400 MHz, $CDCl_3$).

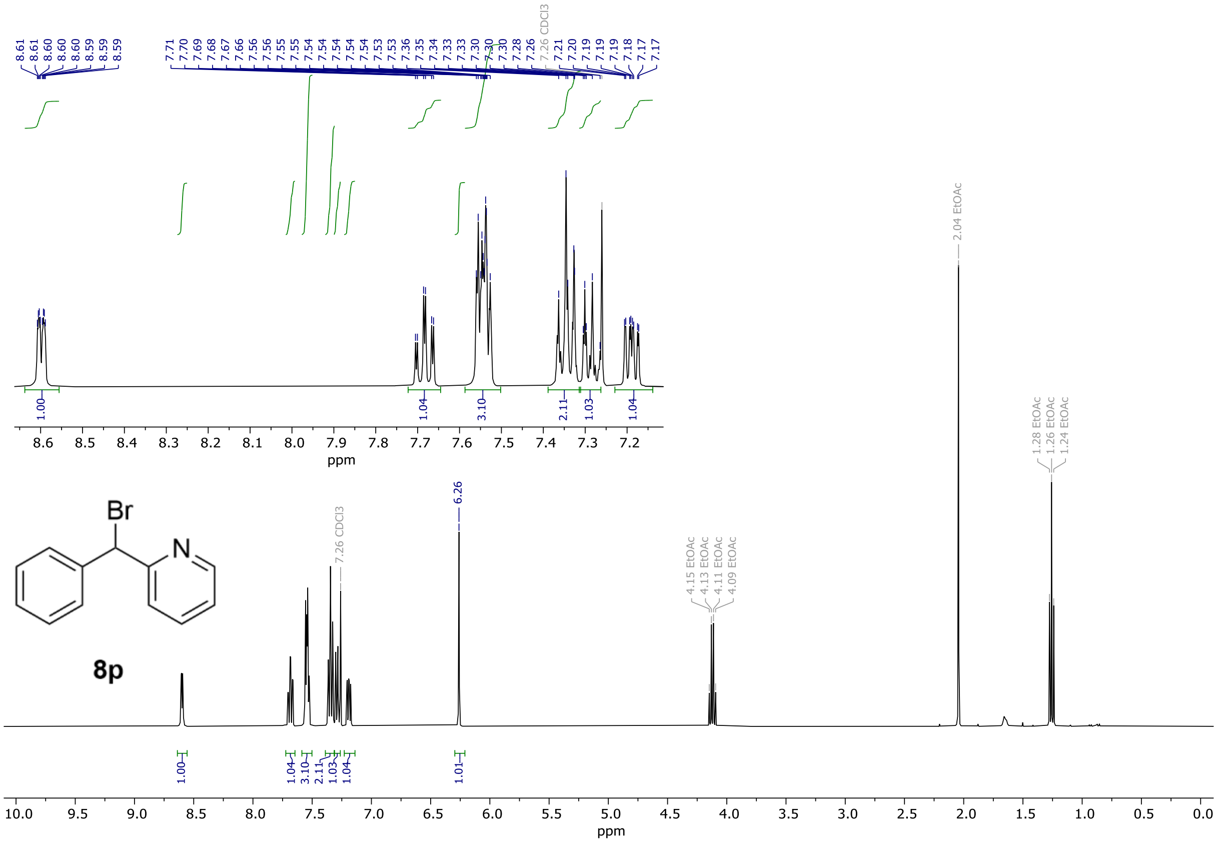

Figure S260 ^{1}H NMR spectrum of **8p** (400 MHz, $CDCl_3$).

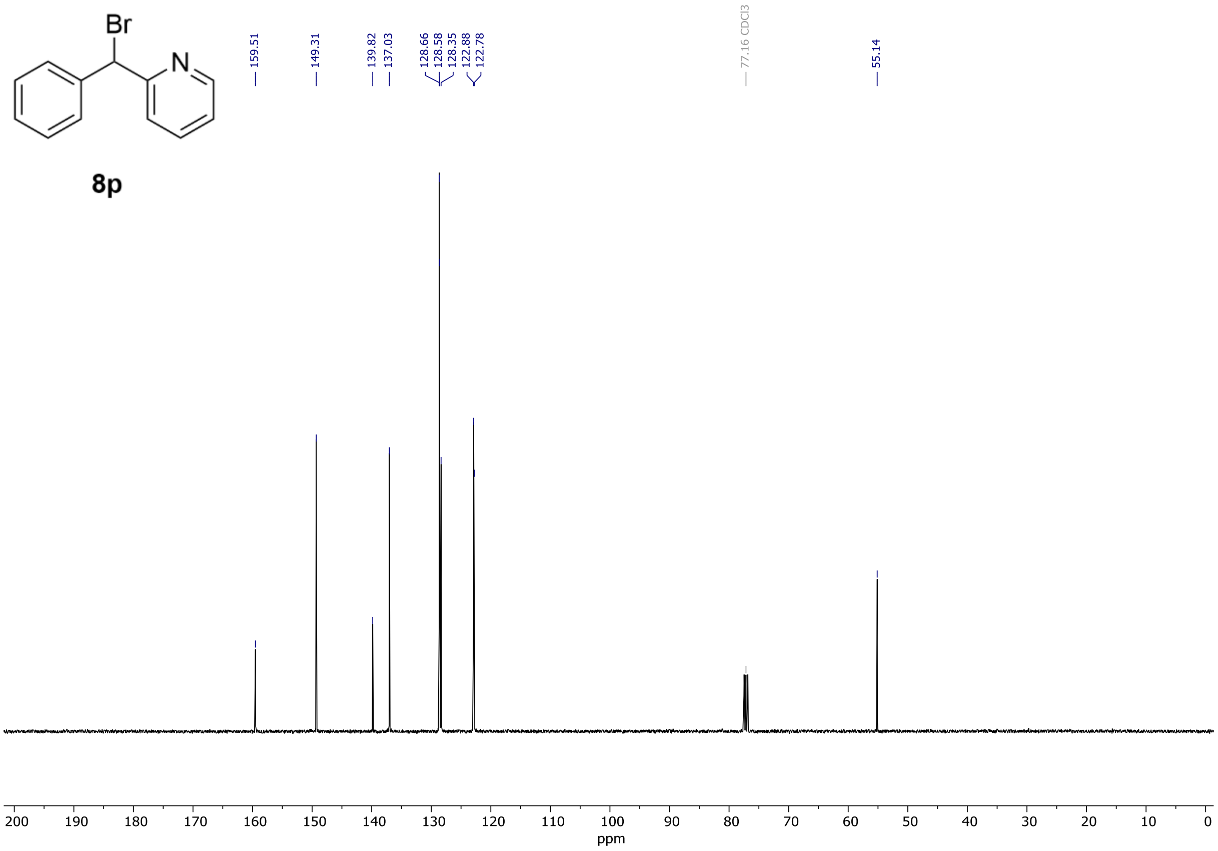

Figure S261 ^{13}C {^{1}H} NMR spectrum of **8p** (101 MHz, $CDCl_3$).

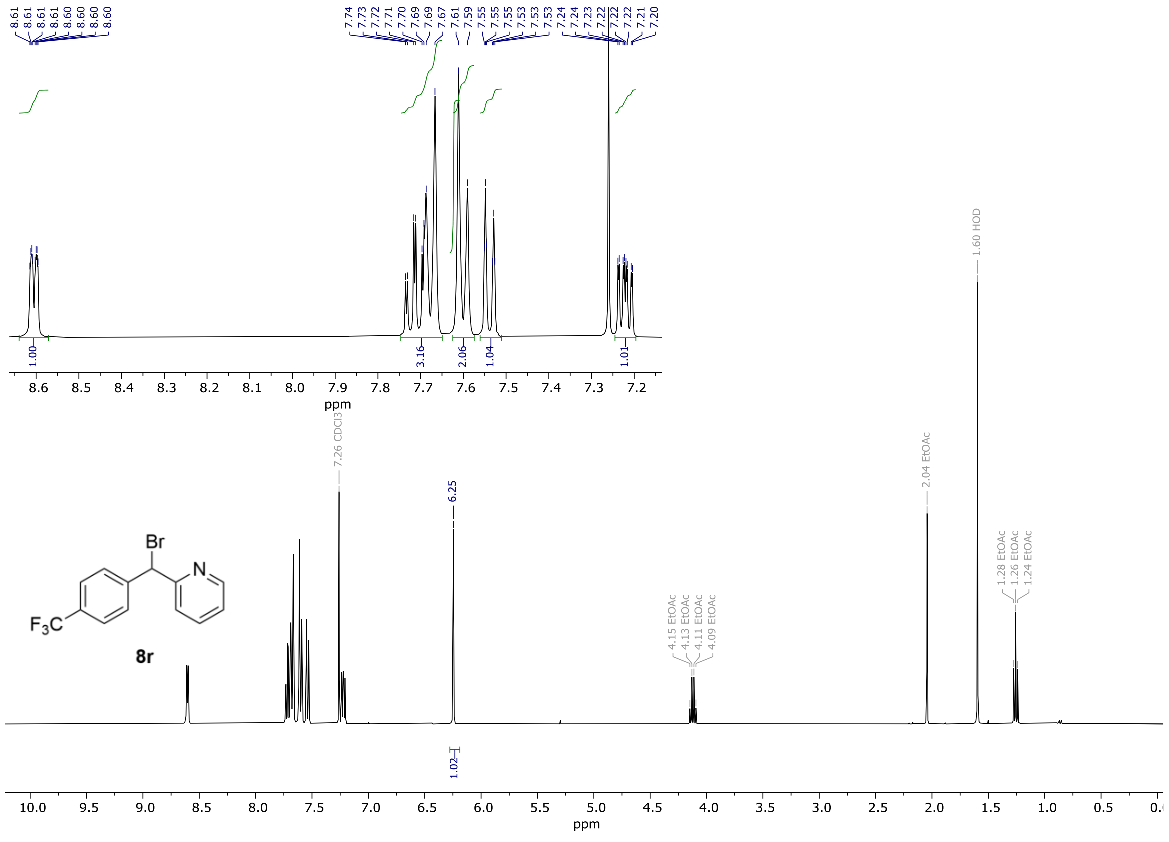

Figure S262 ^{1}H NMR spectrum of **8r** (400 MHz, $CDCl_3$).

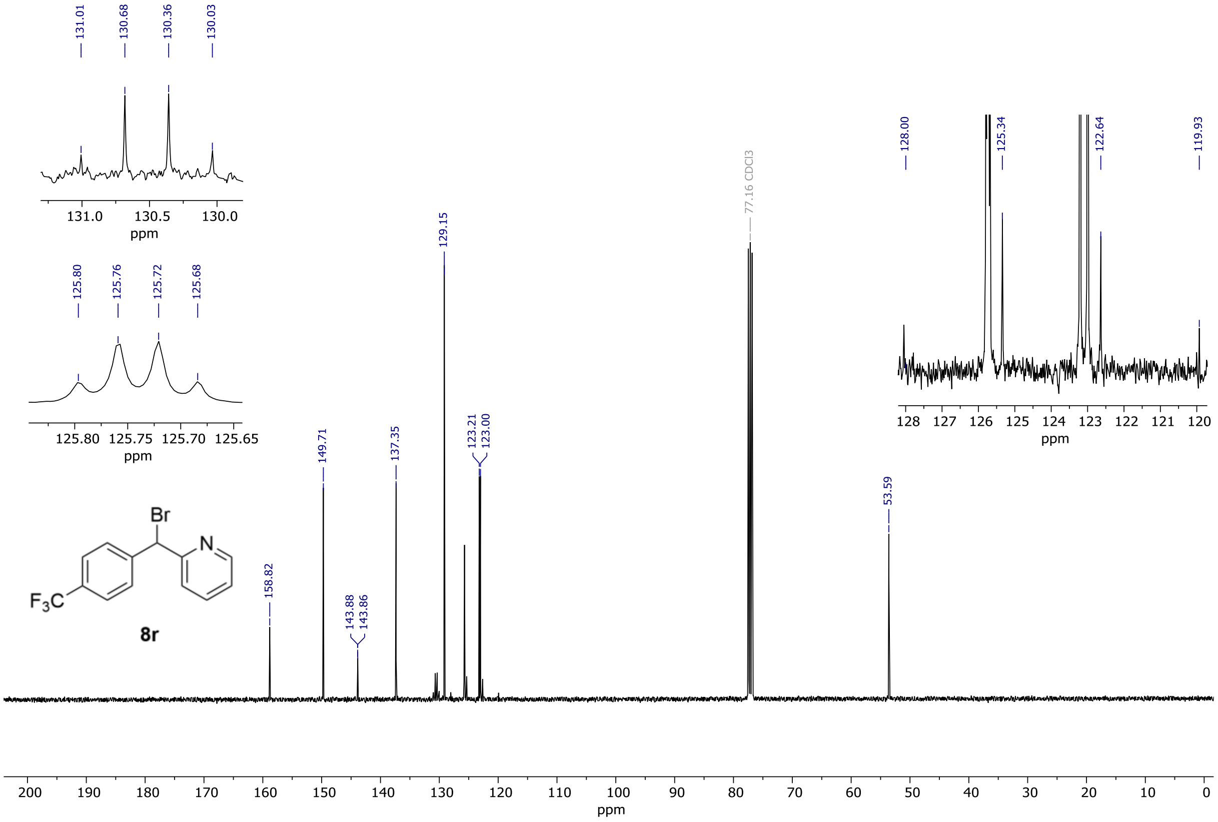

Figure S263 ^{13}C *{^1H} NMR spectrum of* ***8r*** *(101 MHz, $CDCl_3$).*

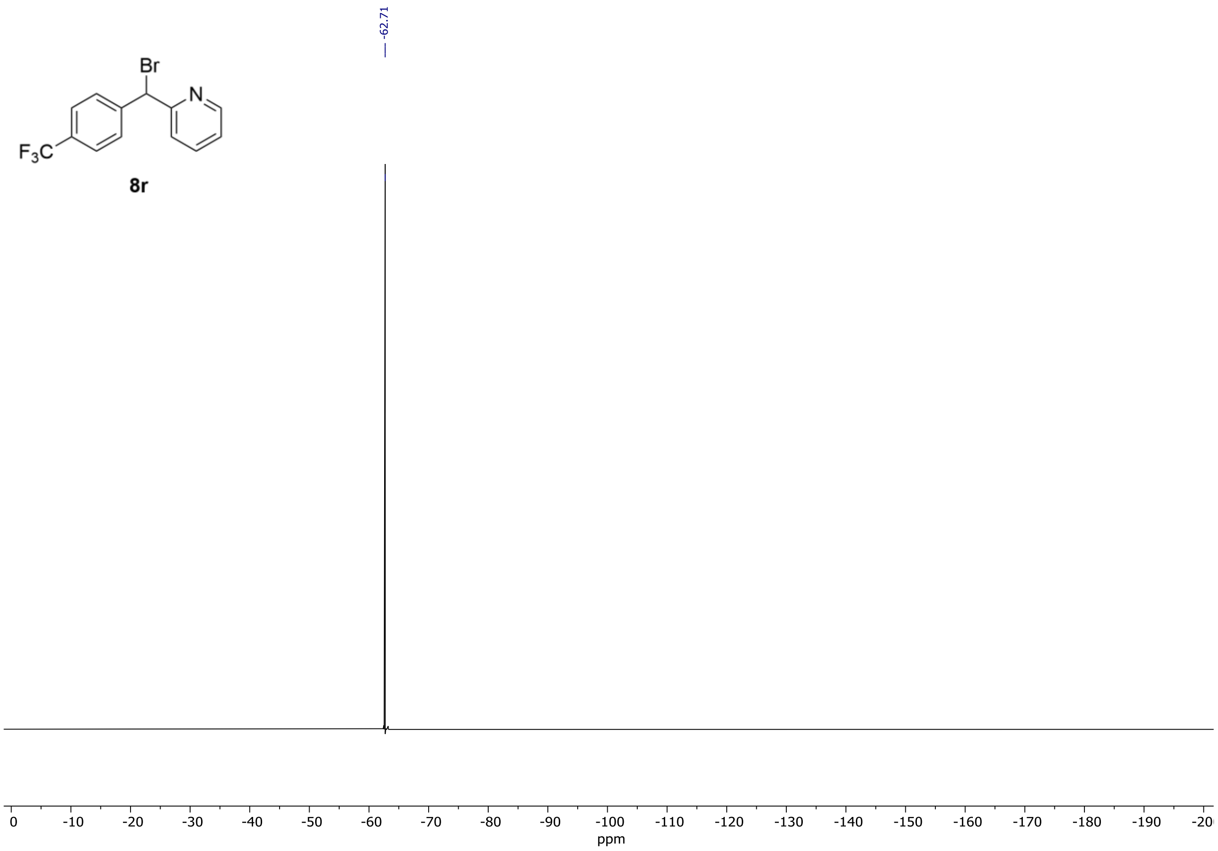

Figure S264 ^{19}F *{^1H} NMR spectrum of* ***8r*** *(376 MHz, $CDCl_3$).*

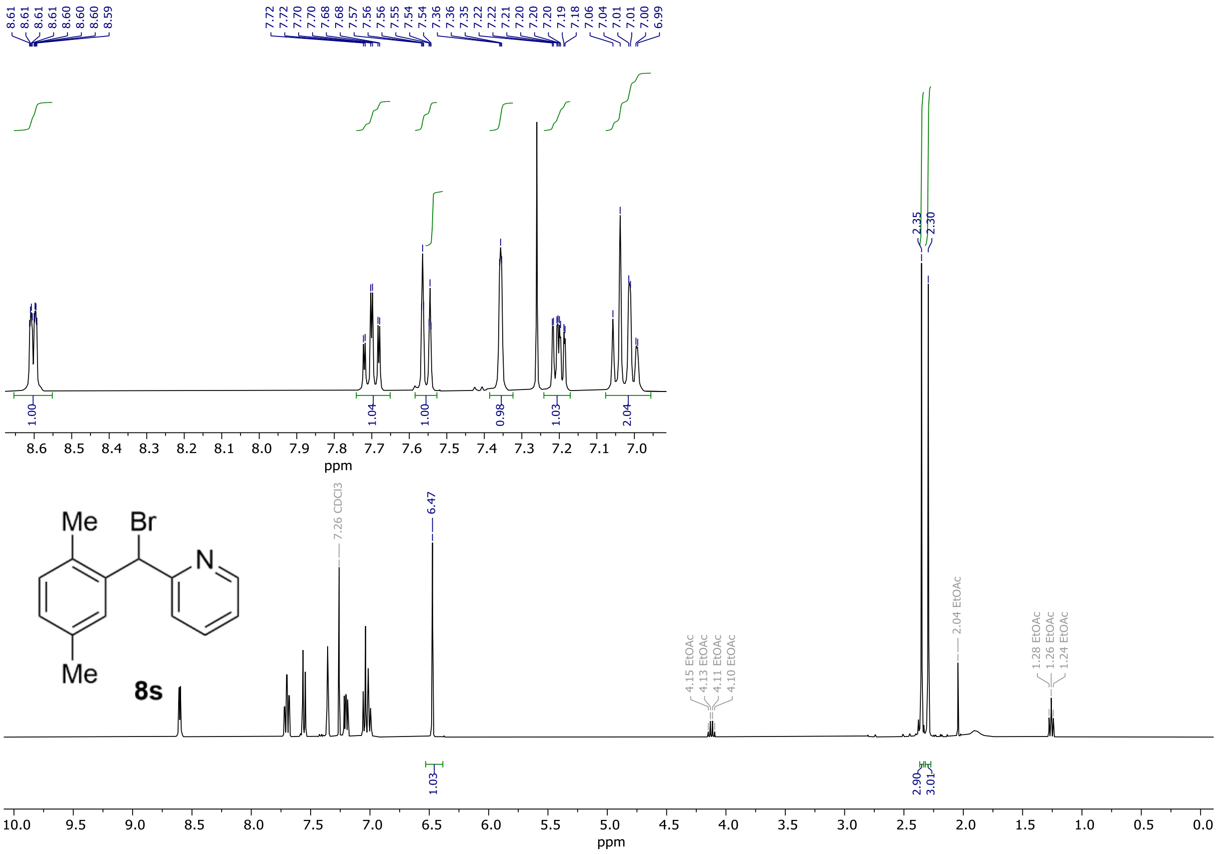

Figure S265 ^{1}H *NMR spectrum of* ***8s*** *(400 MHz, $CDCl_3$).*

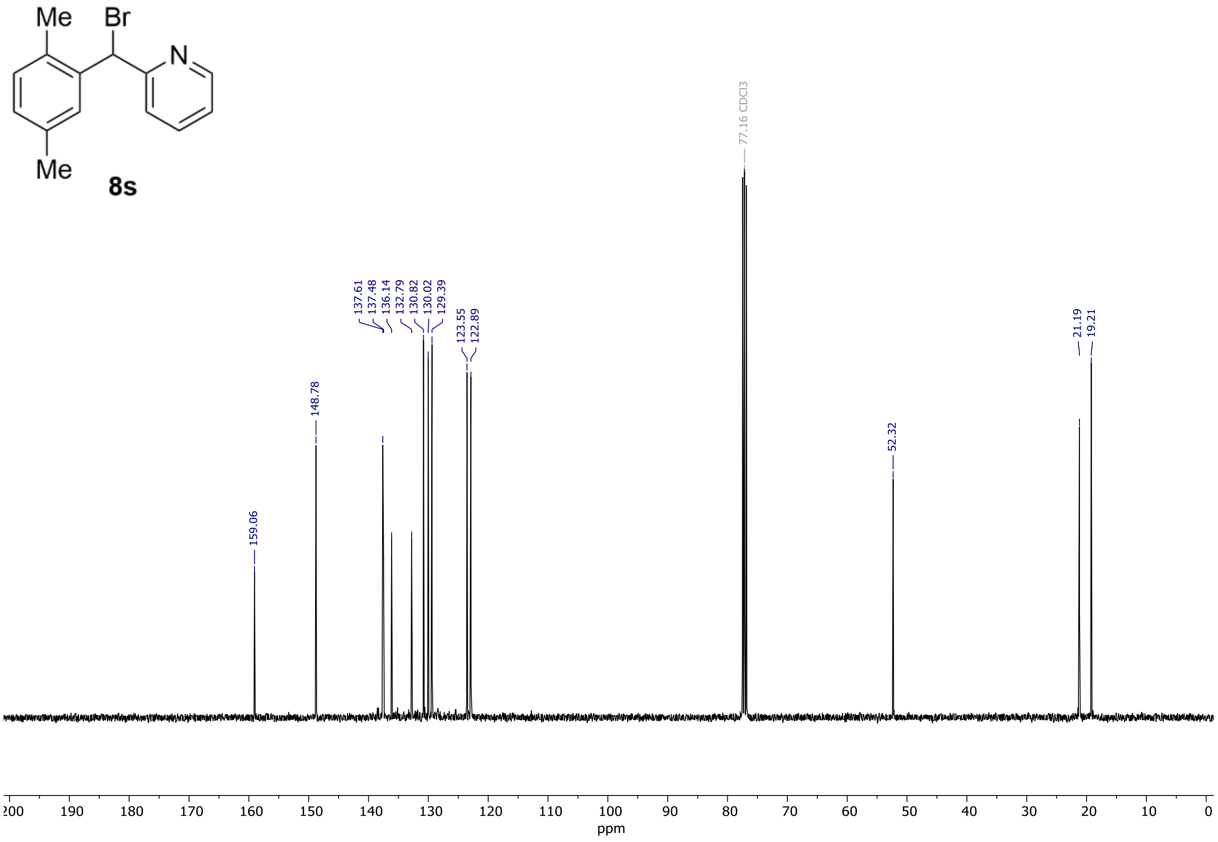

Figure S266 $^{13}C\{^{1}H\}$ *NMR spectrum of* ***8s*** *(101 MHz, $CDCl_3$).*

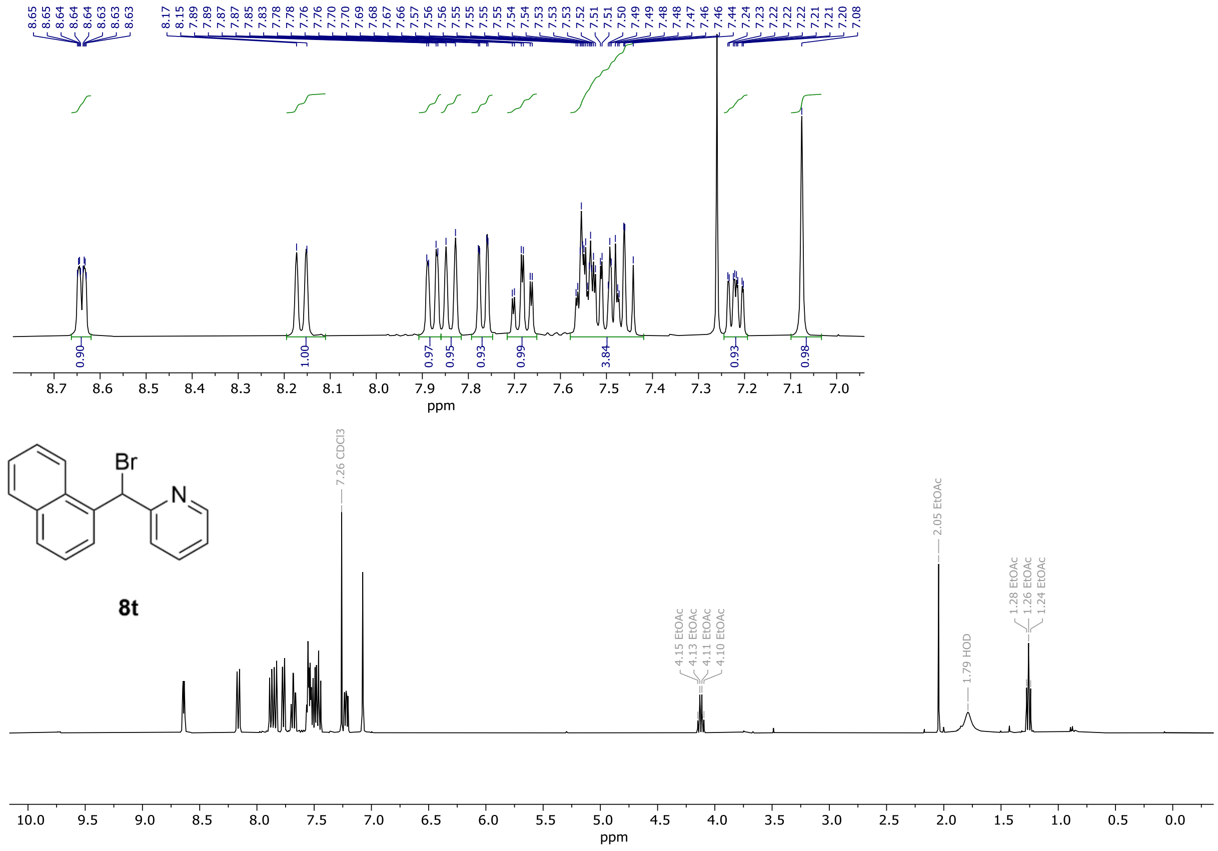

Figure S267 *^{1}H NMR spectrum of **8t** (400 MHz, $CDCl_3$).*

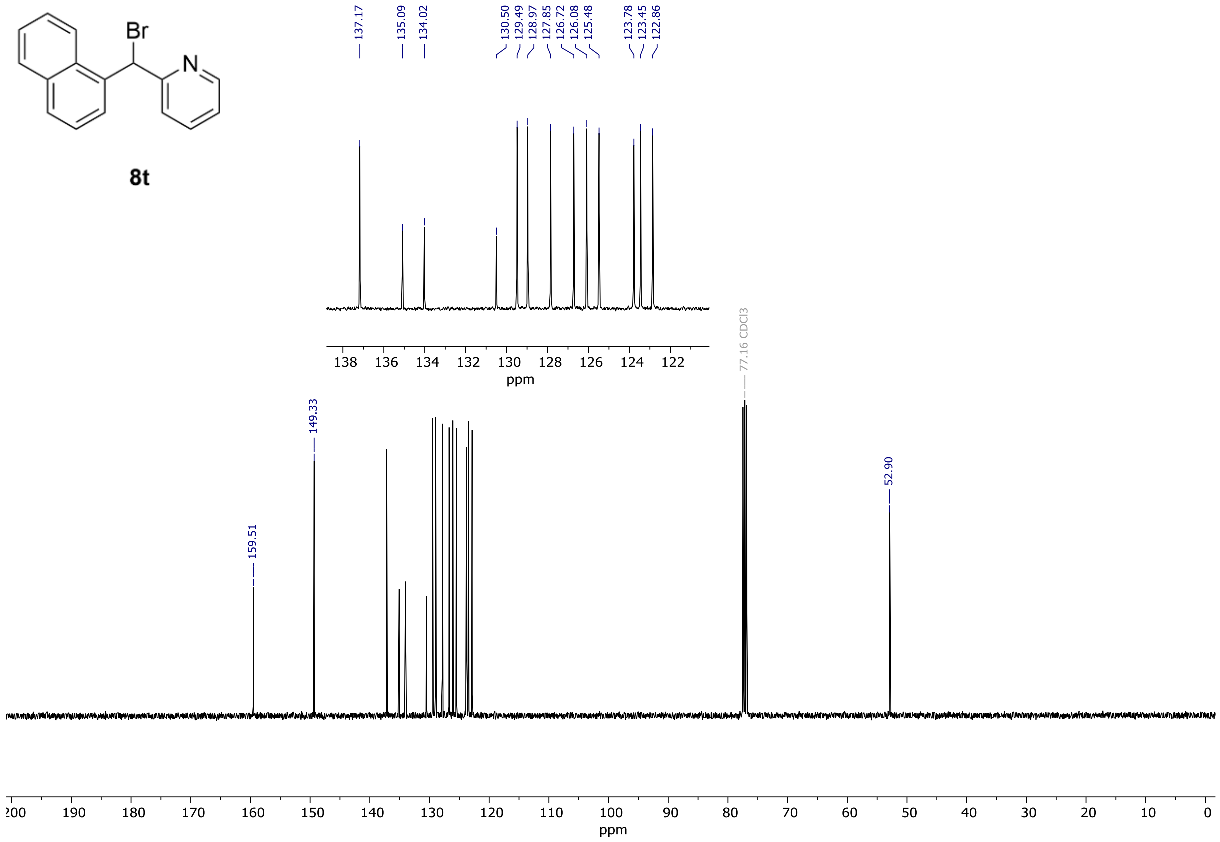

Figure S268 *^{13}C {^{1}H} NMR spectrum of **8t** (101 MHz, $CDCl_3$).*

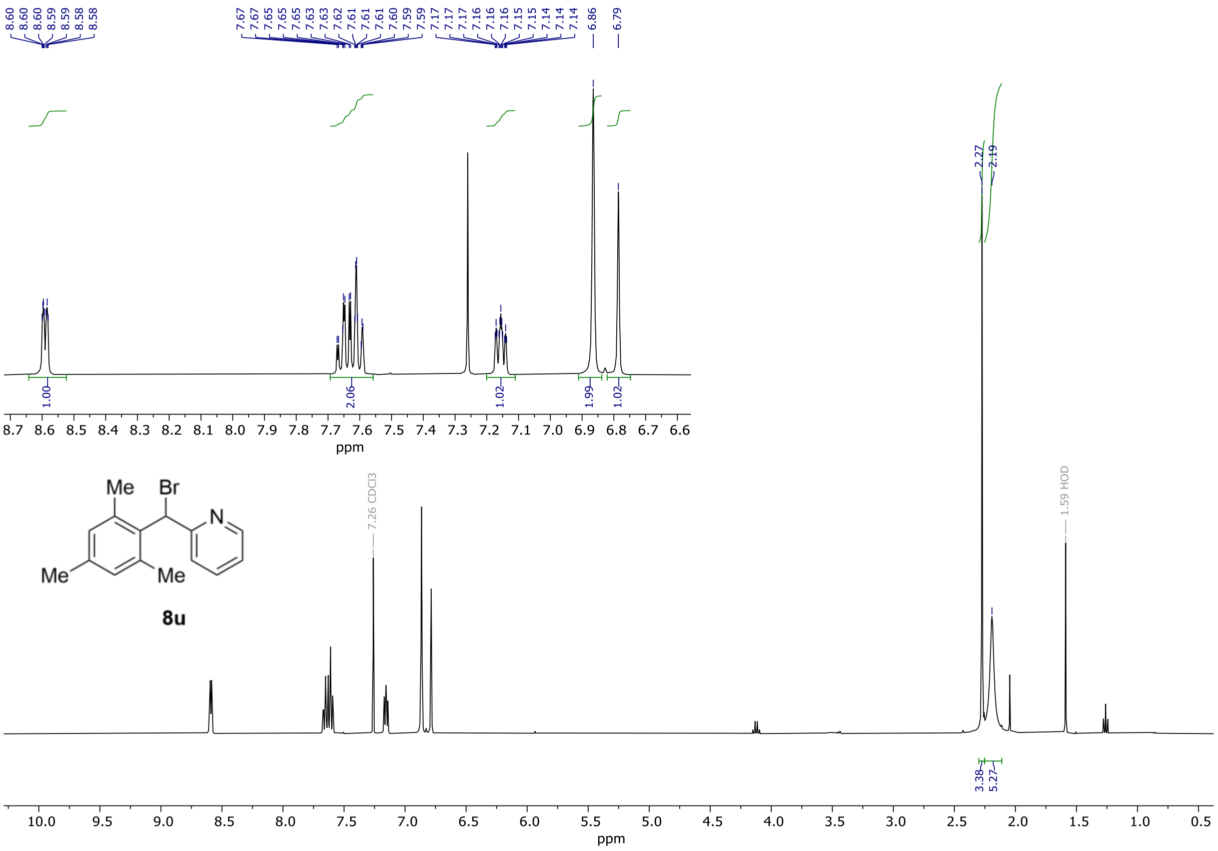

Figure S269 ^{1}*H NMR spectrum of* ***8u*** *(400 MHz, $CDCl_3$).*

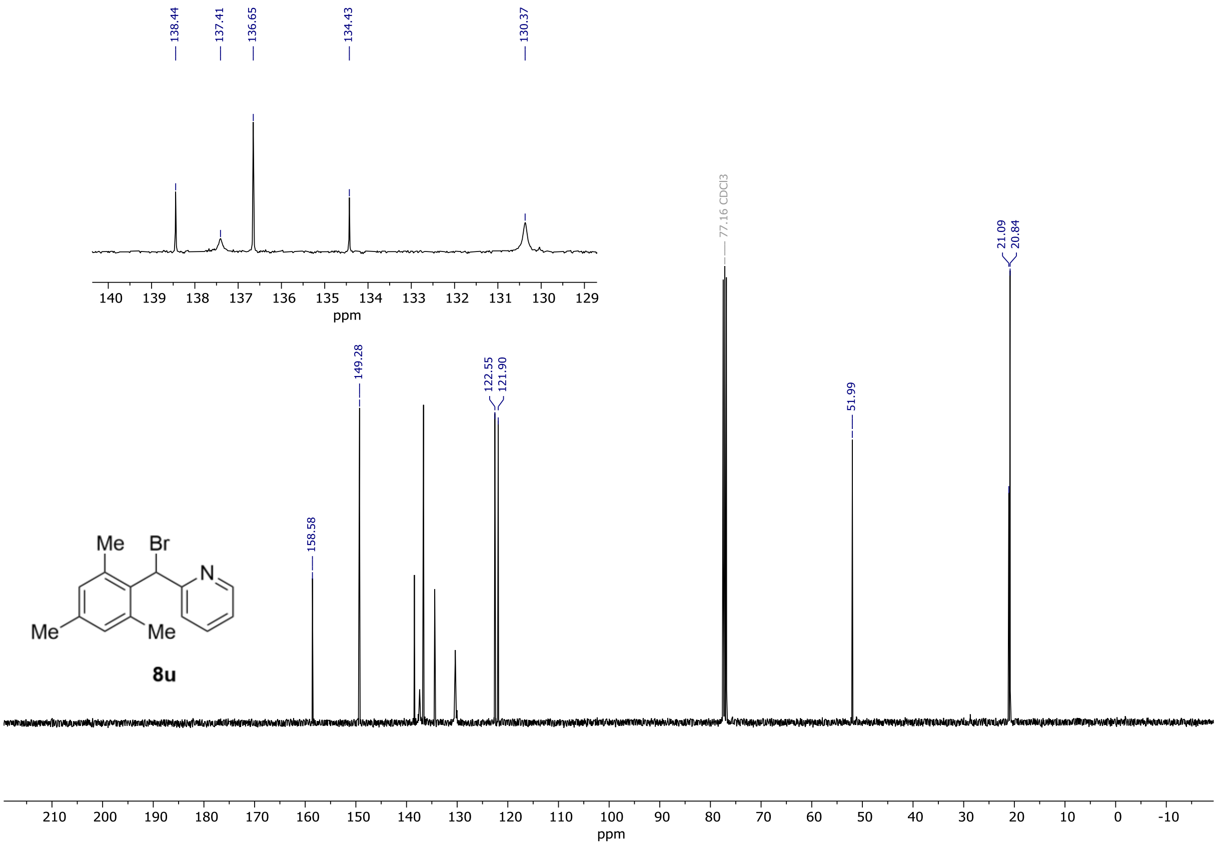

Figure S270 ^{13}*C {^{1}H} NMR spectrum of* ***8u*** *(101 MHz, $CDCl_3$).*

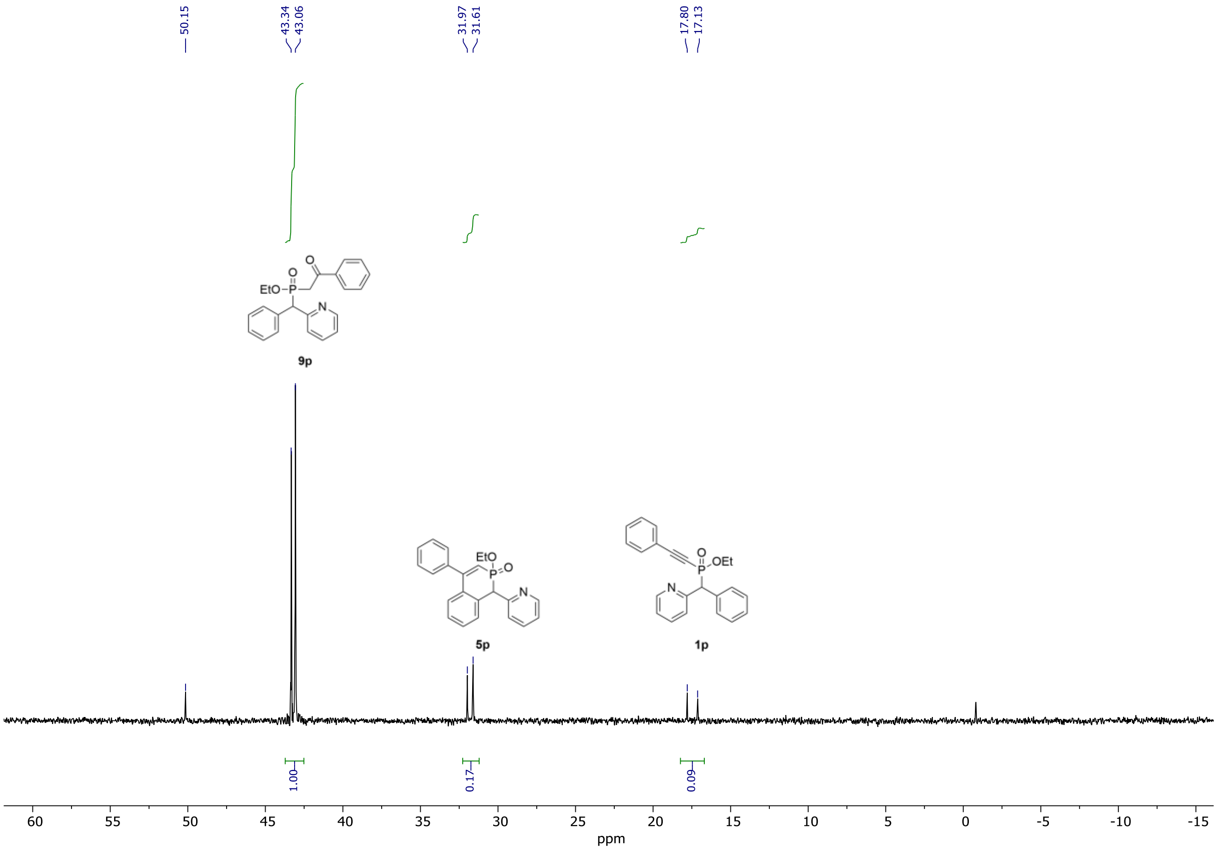

Figure S271 *^{31}P {^{1}H} NMR spectrum of reaction mixture of* ***1p*** *after 18 days in TFA at 80°C (162 MHz, $CDCl_3$).*

3. DFT Calculations

Optimization of the structures and calculation of HOMO/LUMO energies were performed with the Gaussian09 programme[1]. B3LYP[2,3] was used as the functional in combination with the 6-311++G(d,p) basis set. The vibrational analysis showed that all structures correspond to the local minima (zero imaginary frequencies) in the potential energy surface. NICS(0) values were obtained using nmr=giao keyword from shielding values of dummy atoms (Bq) at the centroids of individual rings. The Cartesian coordinates of all optimized structures are following.

*2-Ethoxy-4-phenylpyrido[1,2-a][1,4]azaphosphinine 2-oxide (**2a**)*

```
Total energy =                             -1166.14240369 a.u.
Number of imaginary frequencies =             0
Zero-point correction =                         0.286017(Hartree/Particle)
 Thermal correction to Energy =                   0.303953
 Thermal correction to Enthalpy =                 0.304897
 Thermal correction to Gibbs Free Energy =        0.237664
 Sum of electronic and zero-point Energies =         -1165.856387
 Sum of electronic and thermal Energies =            -1165.838451
 Sum of electronic and thermal Enthalpies =          -1165.837507
 Sum of electronic and thermal Free Energies =       -1165.904740

Charge =  0 Multiplicity = 1
 C                   0.34676   3.4019   -0.24708
 C                  -0.85847   4.01091  -0.3198
 C                  -2.04389   3.23131  -0.13771
 C                  -1.93257   1.89843   0.04638
 N                  -0.70636   1.23336   0.06864
 C                   0.49756   1.98529   0.00938
 C                   1.74327   1.44318   0.17189
 P                   2.0587   -0.21705   0.63557
 C                   0.4311   -0.89662   0.40399
 O                   2.69885  -0.5011    1.95719
 C                  -0.69073  -0.17582   0.18771
 C                  -1.98982  -0.90185   0.02496
 C                  -2.60499  -0.99511  -1.23072
 C                  -3.77385  -1.73409  -1.3885
 C                  -4.3452   -2.38477  -0.2943
 C                  -3.73846  -2.29768   0.9564
 C                  -2.56446  -1.56196   1.1158
 O                   2.96098  -0.80688  -0.59819
 C                   5.09796  -1.58783  -1.38099
 C                   3.97275  -1.8209   -0.39045
 H                   1.26548   3.96392  -0.35601
 H                  -0.92791   5.07716  -0.49887
 H                  -3.02598   3.68371  -0.14063
 H                  -2.79466   1.26849   0.18775
 H                   2.58818   2.11476   0.08054
 H                   0.32338  -1.9742    0.44751
 H                  -2.15891  -0.49704  -2.08467
 H                  -4.23487  -1.80837  -2.36691
 H                  -5.25539  -2.96027  -0.41879
 H                  -4.17502  -2.80381   1.80992
 H                  -2.08847  -1.49755   2.08766
 H                   5.85827  -2.36702  -1.27086
 H                   4.72543  -1.61194  -2.4078
 H                   5.57039  -0.61817  -1.20744
 H                   4.32338  -1.769     0.64223
```

```
H                     3.51029  -2.80094  -0.55144
```

*2-Ethoxy-4-(4-methoxyphenyl)pyrido[1,2-a][1,4]azaphosphinine 2-oxide (**2b**)*

```
Total energy =                              -1280.69965077 a.u.
Number of imaginary frequencies =              0
Zero-point correction =                            0.318307(Hartree/Particle)
 Thermal correction to Energy =                      0.338856
 Thermal correction to Enthalpy =                    0.339801
 Thermal correction to Gibbs Free Energy =           0.266783
 Sum of electronic and zero-point Energies =            -1280.381344
 Sum of electronic and thermal Energies =               -1280.360794
 Sum of electronic and thermal Enthalpies =             -1280.359850
 Sum of electronic and thermal Free Energies =          -1280.432868

Charge =  0 Multiplicity = 1
 C                    1.97977   3.30289  -0.1985
 C                    1.01101   4.24551  -0.24295
 C                   -0.35125   3.85132  -0.05629
 C                   -0.64147   2.54156   0.09781
 N                    0.32925   1.54028   0.08448
 C                    1.70225   1.90109   0.03238
 C                    2.73046   1.01064   0.1808
 P                    2.53344  -0.67574   0.61465
 C                    0.78249  -0.83895   0.35298
 O                    3.042    -1.15785   1.9367
 C                   -0.0777    0.18606   0.16196
 C                   -1.53147  -0.11408  -0.0069
 C                   -2.17686   0.06657  -1.24215
 C                   -3.50475  -0.2869   -1.40963
 C                   -4.23118  -0.83031  -0.33963
 C                   -3.60403  -1.01731   0.894
 C                   -2.26245  -0.66078   1.04709
 O                    3.23996  -1.48832  -0.62065
 C                    5.14959  -2.78323  -1.31455
 C                    3.92472  -2.74728  -0.41943
 H                    3.02361   3.56708  -0.31025
 H                    1.26182   5.28738  -0.40248
 H                   -1.1532    4.57626  -0.03269
 H                   -1.6505    2.19308   0.24059
 H                    3.73707   1.4015    0.09601
 H                    0.36355  -1.83837   0.3567
 H                   -1.62625   0.47705  -2.08157
 H                   -4.00257  -0.16074  -2.36343
 H                   -4.13792  -1.43522   1.73658
 H                   -1.78046  -0.81161   2.00639
 H                    5.65865  -3.74634  -1.21062
 H                    4.87117  -2.65129  -2.36278
 H                    5.85089  -1.99164  -1.04051
 H                    4.19165  -2.84411   0.63487
 H                    3.22976  -3.55299  -0.67955
 O                   -5.52924  -1.14198  -0.60367
 C                   -6.32206  -1.70412   0.43603
 H                   -6.41114  -1.01755   1.28453
 H                   -7.30601  -1.86999   0.00125
 H                   -5.91057  -2.65929   0.77858
```

*2-Ethoxy-4-(4-(trifluoromethyl)phenyl)pyrido[1,2-a][1,4]azaphosphinine 2-oxide (**2n**)*

```
Total energy =                              -1503.29029148 a.u.
Number of imaginary frequencies =              0
Zero-point correction =                            0.290327(Hartree/Particle)
```

```
 Thermal correction to Energy =                   0.311948
 Thermal correction to Enthalpy =                 0.312892
 Thermal correction to Gibbs Free Energy =        0.235815
 Sum of electronic and zero-point Energies =         -1502.999965
 Sum of electronic and thermal Energies =            -1502.978344
 Sum of electronic and thermal Enthalpies =          -1502.977400
 Sum of electronic and thermal Free Energies =       -1503.054477
```

```
Charge =  0 Multiplicity = 1
 C                    2.70686   3.22615  -0.41984
 C                    1.80468   4.23339  -0.40745
 C                    0.4478    3.94888  -0.05265
 C                    0.0861    2.67142   0.18992
 N                    0.97945   1.60154   0.11041
 C                    2.36266   1.86004  -0.08745
 C                    3.33543   0.90396   0.01199
 P                    3.06568  -0.73232   0.58225
 C                    1.29077  -0.79182   0.44913
 O                    3.62796  -1.15009   1.90304
 C                    0.49417   0.28479   0.27121
 C                   -0.98394   0.0731    0.17978
 C                   -1.67059   0.30654  -1.02038
 C                   -3.02778   0.03196  -1.12162
 C                   -3.71963  -0.47456  -0.01828
 C                   -3.04863  -0.71301   1.17836
 C                   -1.68581  -0.43949   1.27422
 O                    3.62491  -1.67349  -0.63254
 C                    5.35494  -3.15843  -1.40713
 C                    4.25188  -2.95642  -0.38562
 H                    3.7478    3.40957  -0.65363
 H                    2.10587   5.24674  -0.64403
 H                   -0.29242   4.73265   0.0305
 H                   -0.92188   2.40658   0.46272
 H                    4.35323   1.21143  -0.19459
 H                    0.80961  -1.76048   0.51737
 H                   -1.13643   0.69539  -1.87971
 H                   -3.54904   0.21034  -2.05411
 H                   -3.58509  -1.10265   2.03399
 H                   -1.16276  -0.62104   2.2056
 H                    5.81653  -4.14042  -1.26634
 H                    4.95996  -3.10559  -2.42434
 H                    6.12866  -2.39521  -1.29613
 H                    4.6361   -2.97449   0.63606
 H                    3.4838   -3.73092  -0.48288
 C                   -5.18171  -0.80657  -0.15273
 F                   -5.80621  -0.87269   1.0424
 F                   -5.84146   0.10928  -0.89942
 F                   -5.37028  -2.00534  -0.75963
```

*2-Ethoxy-1,4-diphenylpyrido[1,2-a][1,4]azaphosphinine 2-oxide (**2p**)*

```
Total energy =                                  -1397.24966279 a.u.
Number of imaginary frequencies =                   0
Zero-point correction =                              0.366750(Hartree/Particle)
 Thermal correction to Energy =                    0.389443
 Thermal correction to Enthalpy =                  0.390387
 Thermal correction to Gibbs Free Energy =         0.312838
 Sum of electronic and zero-point Energies =          -1396.882913
 Sum of electronic and thermal Energies =             -1396.860220
 Sum of electronic and thermal Enthalpies =           -1396.859276
 Sum of electronic and thermal Free Energies =        -1396.936825
```

```
Charge =  0 Multiplicity = 1
 O                  1.19911   1.66141  -2.06268
 C                 -4.04486   0.9413   -0.90973
 C                 -5.39567   1.16293  -0.64321
 C                 -5.94299   0.76529   0.57402
 C                 -5.13452   0.14371   1.52682
 C                 -3.78796  -0.08352   1.26093
 C                 -3.22928   0.31448   0.03812
 C                  3.50419  -0.55163  -1.01014
 C                  4.85989  -0.7816   -0.77921
 C                  5.30328  -1.17788   0.48102
 C                  4.38165  -1.33531   1.51634
 C                  3.02777  -1.10252   1.28919
 C                  2.56616  -0.71885   0.02033
 C                  0.5211   -2.80889   0.01638
 C                 -0.37714  -3.8183   -0.05942
 C                 -1.75242  -3.51609  -0.30418
 C                 -2.13004  -2.2237   -0.38834
 C                  0.16031  -1.41475  -0.14314
 N                 -1.24123  -1.15751  -0.23796
 C                 -1.76627   0.15221  -0.2243
 C                 -1.00761   1.25755  -0.38509
 P                  0.73237   1.23215  -0.70657
 C                  1.12293  -0.43137  -0.21666
 O                  1.30346   2.21632   0.46642
 C                  2.31333   4.2788    1.17585
 C                  2.44667   3.08116   0.25497
 H                 -3.61768   1.25053  -1.85681
 H                 -6.01754   1.64634  -1.38807
 H                 -6.99223   0.94063   0.78259
 H                 -5.55215  -0.15745   2.48088
 H                 -3.16215  -0.56073   2.00703
 H                  3.16189  -0.2352   -1.98824
 H                  5.56956  -0.65189  -1.58899
 H                  6.35799  -1.3569    0.65798
 H                  4.71839  -1.63026   2.5043
 H                  2.31683  -1.20529   2.10178
 H                  1.57145  -3.01913   0.15268
 H                 -0.05191  -4.84769   0.03338
 H                 -2.49201  -4.29389  -0.43596
 H                 -3.15124  -1.93913  -0.57936
 H                 -1.49117   2.22262  -0.29694
 H                  3.18169   4.93432   1.05974
 H                  1.4143    4.85273   0.93876
 H                  2.25711   3.96371   2.22046
 H                  3.3517    2.50941   0.48132
 H                  2.47849   3.37746  -0.79551
```

*2-Ethoxy-1-(4-methoxyphenyl)-4-phenylpyrido[1,2-a][1,4]azaphosphinine 2-oxide (**2q**)*

```
Total energy =                              -1511.80621956 a.u.
Number of imaginary frequencies =                0
Zero-point correction =                           0.398850(Hartree/Particle)
 Thermal correction to Energy =                    0.424302
 Thermal correction to Enthalpy =                  0.425246
 Thermal correction to Gibbs Free Energy =         0.339184
 Sum of electronic and zero-point Energies =          -1511.407369
 Sum of electronic and thermal Energies =             -1511.381918
 Sum of electronic and thermal Enthalpies =           -1511.380974
 Sum of electronic and thermal Free Energies =        -1511.467036
```

Charge = 0 Multiplicity = 1

O	0.49887	1.83164	-2.05099
C	-4.6767	0.68082	-0.91059
C	-6.04027	0.80039	-0.6434
C	-6.55329	0.37552	0.57959
C	-5.69769	-0.17081	1.53734
C	-4.33802	-0.2966	1.27056
C	-3.81377	0.12942	0.0421
C	2.96334	-0.21841	-1.04361
C	4.3396	-0.3428	-0.83948
C	4.82433	-0.71567	0.41547
C	3.9216	-0.95199	1.4603
C	2.55878	-0.82117	1.24498
C	2.04708	-0.46358	-0.01544
C	0.16664	-2.70111	-0.01078
C	-0.65263	-3.77613	-0.07581
C	-2.0508	-3.579	-0.29826
C	-2.52671	-2.31915	-0.37638
C	-0.30118	-1.33788	-0.15976
N	-1.72001	-1.18765	-0.23918
C	-2.34262	0.07792	-0.22207
C	-1.67146	1.23917	-0.38104
P	0.06503	1.34928	-0.70119
C	0.58438	-0.28479	-0.23577
O	0.55669	2.36186	0.48438
C	1.37357	4.52613	1.14521
C	1.64345	3.29977	0.29401
H	-4.27576	1.01252	-1.86153
H	-6.69879	1.22605	-1.392
H	-6.0893	-0.49255	2.49571
H	-3.6759	-0.71556	2.02028
H	2.59555	0.08052	-2.01799
H	5.01223	-0.14802	-1.66408
H	4.31229	-1.22551	2.4333
H	1.87243	-0.98749	2.0681
H	1.23203	-2.82995	0.10903
H	-0.24847	-4.77787	0.00915
H	-2.73155	-4.41057	-0.41837
H	-3.56947	-2.1137	-0.55153
H	-2.22834	2.16349	-0.28921
H	2.20149	5.23499	1.04788
H	0.45448	5.02433	0.82757
H	1.2742	4.25556	2.19908
H	2.57132	2.80621	0.59865
H	1.71461	3.55031	-0.76647
H	-7.61261	0.47161	0.78881
O	6.14583	-0.86978	0.72409
C	7.11138	-0.63418	-0.29169
H	8.08044	-0.81163	0.17206
H	6.98171	-1.32185	-1.1345
H	7.06683	0.39843	-0.65459

*2-Ethoxy-4-phenyl-1-(4-(trifluoromethyl)phenyl)pyrido[1,2-a][1,4]azaphosphinine 2-oxide (**2r**)*

Total energy = -1734.40009213 a.u.
Number of imaginary frequencies = 0
Zero-point correction = 0.370980(Hartree/Particle)
Thermal correction to Energy = 0.397462
Thermal correction to Enthalpy = 0.398406
Thermal correction to Gibbs Free Energy = 0.309771
Sum of electronic and zero-point Energies = -1734.029112

```
 Sum of electronic and thermal Energies =            -1734.002630
 Sum of electronic and thermal Enthalpies =          -1734.001686
 Sum of electronic and thermal Free Energies =       -1734.090321

Charge =  0 Multiplicity = 1
 O                  -0.16274   1.9608   -2.09703
 C                  -5.21753   0.53863  -0.75428
 C                  -6.57128   0.58101  -0.42201
 C                  -7.00499   0.09445   0.80868
 C                  -6.08021  -0.43624   1.70915
 C                  -4.72994  -0.48534   1.37752
 C                  -4.28574   0.00309   0.14083
 C                   2.44145   0.05903  -1.22402
 C                   3.82286  -0.00333  -1.06409
 C                   4.36716  -0.42135   0.14948
 C                   3.52465  -0.77227   1.20652
 C                   2.14679  -0.70547   1.04342
 C                   1.58     -0.30224  -0.1765
 C                  -0.1892   -2.63212  -0.18702
 C                  -0.96143  -3.74173  -0.25881
 C                  -2.37403  -3.60319  -0.41519
 C                  -2.90883  -2.36439  -0.42698
 C                  -0.72474  -1.28908  -0.26264
 N                  -2.14811  -1.20314  -0.28132
 C                  -2.82821   0.03185  -0.19146
 C                  -2.21861   1.22665  -0.34173
 P                  -0.507     1.43041  -0.74032
 C                   0.10561  -0.1898   -0.33822
 O                   0.00451   2.42734   0.4469
 C                   0.58334   4.68867   1.05013
 C                   0.97194   3.48269   0.21613
 H                  -4.87829   0.91822  -1.71135
 H                  -7.28424   0.99476  -1.12603
 H                  -6.41055  -0.80579   2.67328
 H                  -4.01387  -0.89252   2.0829
 H                   2.01925   0.39414  -2.16355
 H                   4.47416   0.27637  -1.88284
 H                   3.94505  -1.07878   2.15654
 H                   1.49634  -0.95274   1.87451
 H                   0.88498  -2.71944  -0.12054
 H                  -0.50811  -4.72539  -0.2302
 H                  -3.02077  -4.46095  -0.53812
 H                  -3.96618  -2.20055  -0.55115
 H                  -2.81211   2.12074  -0.19586
 H                   1.32827   5.48032   0.92545
 H                  -0.38867   5.07927   0.73967
 H                   0.53063   4.42982   2.11022
 H                   1.95372   3.10083   0.51111
 H                   0.99395   3.71692  -0.84993
 H                  -8.05687   0.13037   1.06817
 C                   5.85543  -0.54762   0.30916
 F                   6.29199  -1.8029    0.01814
 F                   6.25971  -0.29016   1.57562
 F                   6.53651   0.29128  -0.50327
```

*2-Ethoxy-1-(naphthalen-1-yl)-4-phenylpyrido[1,2-a][1,4]azaphosphinine 2-oxide (**2t**)*

```
Total energy =                            -1550.92088830 a.u.
Number of imaginary frequencies =             0
Zero-point correction =                         0.413202(Hartree/Particle)
 Thermal correction to Energy =                   0.438526
```

```
 Thermal correction to Enthalpy =                 0.439470
 Thermal correction to Gibbs Free Energy =        0.355979
 Sum of electronic and zero-point Energies =         -1550.507686
 Sum of electronic and thermal Energies =            -1550.482362
 Sum of electronic and thermal Enthalpies =          -1550.481418
 Sum of electronic and thermal Free Energies =       -1550.564909

Charge =  0 Multiplicity = 1
 O                    0.03832   2.18821  -2.43678
 C                   -4.708     0.67759  -0.50506
 C                   -6.01153   0.63903  -0.01129
 C                   -6.2905   -0.03354   1.17584
 C                   -5.26036  -0.67035   1.86948
 C                   -3.9602   -0.63853   1.37552
 C                   -3.67038   0.03845   0.1821
 C                    5.10751  -1.02144   1.3258
 C                    4.50118  -1.44717   2.48218
 C                    3.09295  -1.40511   2.5925
 C                    2.32426  -0.94955   1.54686
 C                    2.80892   0.41705  -1.91572
 C                    4.21755   0.39249  -2.00233
 C                    4.97076  -0.08297  -0.95866
 C                    4.34394  -0.54133   0.22842
 C                    2.91404  -0.50978   0.33026
 C                    2.14374  -0.03149  -0.78676
 C                    0.42934  -2.39693  -0.84828
 C                   -0.31877  -3.51775  -0.97908
 C                   -1.74293  -3.41235  -0.99602
 C                   -2.3075   -2.19983  -0.81681
 C                   -0.14359  -1.07352  -0.72517
 N                   -1.56518  -1.03766  -0.60341
 C                   -2.26554   0.15556  -0.31373
 C                   -1.70355   1.37826  -0.42051
 P                   -0.06458   1.6593   -1.04668
 C                    0.65359   0.05473  -0.74385
 O                    0.61037   2.74334  -0.03415
 C                    1.31528   3.84166   1.9698
 C                    0.6949    2.58205   1.39525
 H                   -4.4901    1.19968  -1.4297
 H                   -6.80681   1.1343   -0.5566
 H                   -7.30333  -0.06118   1.56119
 H                   -5.46955  -1.18698   2.79934
 H                   -3.16306  -1.13152   1.92108
 H                    6.18862  -1.04229   1.23357
 H                    5.09782  -1.80994   3.3116
 H                    2.61646  -1.73266   3.51002
 H                    1.24678  -0.92123   1.6464
 H                    2.22901   0.7994   -2.74716
 H                    4.69762   0.74993  -2.90634
 H                    6.05354  -0.11003  -1.02344
 H                    1.5071   -2.45138  -0.88321
 H                    0.15957  -4.48214  -1.10207
 H                   -2.37635  -4.27272  -1.16281
 H                   -3.37551  -2.05946  -0.8302
 H                   -2.30102   2.23679  -0.13685
 H                    1.39954   3.75341   3.05693
 H                    2.31314   3.99976   1.55573
 H                    0.70298   4.7156    1.73706
 H                   -0.30806   2.41726   1.80556
 H                    1.30637   1.70568   1.62694
```

*8-Ethoxy-6-phenylpyrazino[1,2-a][1,4]azaphosphinine 8-oxide (**4a**)*

```
Total energy =                              -1182.75808781 a.u.
Number of imaginary frequencies =              0
Zero-point correction =                            0.283742(Hartree/Particle)
 Thermal correction to Energy =                      0.302511
 Thermal correction to Enthalpy =                    0.303456
 Thermal correction to Gibbs Free Energy =           0.234175
 Sum of electronic and zero-point Energies =           -1182.474346
 Sum of electronic and thermal Energies =              -1182.455576
 Sum of electronic and thermal Enthalpies =            -1182.454632
 Sum of electronic and thermal Free Energies =         -1182.523913

Charge =  0 Multiplicity = 2
 C                  -2.08357   3.16934  -0.05273
 C                  -1.96133   1.85159   0.17034
 N                  -0.71711   1.17957   0.09347
 C                   0.46271   1.97922   0.0111
 C                   1.72838   1.45918   0.16656
 P                   2.0725   -0.19757   0.63216
 C                   0.46706  -0.92435   0.40642
 O                   2.73208  -0.45936   1.95026
 C                  -0.66957  -0.21476   0.2011
 C                  -1.9558   -0.95925   0.02931
 C                  -2.64889  -0.92257  -1.18846
 C                  -3.80473  -1.67724  -1.36479
 C                  -4.28894  -2.47253  -0.32531
 C                  -3.60722  -2.51257   0.88865
 C                  -2.44528  -1.76183   1.06504
 O                   2.9974   -0.75106  -0.60577
 C                   5.09549  -1.59087  -1.4233
 C                   3.98729  -1.78506  -0.4053
 H                  -1.04951   4.92336  -0.46277
 H                  -3.03529   3.67456   0.00591
 H                  -2.81558   1.24937   0.42345
 H                   2.56427   2.14098   0.06566
 H                   0.38548  -2.00396   0.42915
 H                  -2.27165  -0.31123  -2.00074
 H                  -4.32446  -1.65028  -2.31596
 H                  -5.18987  -3.05943  -0.46378
 H                  -3.97721  -3.12819   1.70077
 H                  -1.91276  -1.79338   2.00865
 H                   5.84245  -2.38354  -1.31759
 H                   4.70002  -1.6227   -2.44135
 H                   5.59046  -0.62825  -1.27501
 H                   4.36282  -1.72709   0.61852
 H                   3.5025   -2.75861  -0.54169
 N                  -0.96636   3.92718  -0.35026
 C                   0.28298   3.35728  -0.26206
 H                   1.13785   4.00446  -0.37333
```

*8-Ethoxy-6-phenylpyrimido[1,2-a][1,4]azaphosphinine 8-oxide (**4c**)*

```
Total energy =                              -1182.18498639 a.u.
Number of imaginary frequencies =              0
Zero-point correction =                            0.274092(Hartree/Particle)
 Thermal correction to Energy =                      0.291847
 Thermal correction to Enthalpy =                    0.292791
 Thermal correction to Gibbs Free Energy =           0.226172
 Sum of electronic and zero-point Energies =           -1181.910895
 Sum of electronic and thermal Energies =              -1181.893139
```

```
 Sum of electronic and thermal Enthalpies =           -1181.892195
 Sum of electronic and thermal Free Energies =        -1181.958815

Charge =  0 Multiplicity = 1
 C                 -0.71017   3.91022  -0.25995
 C                 -1.92116   3.25849   0.13303
 C                 -1.87177   1.91624   0.28683
 N                 -0.69736   1.20799   0.11325
 C                  0.51858   1.94994  -0.04714
 C                  1.75276   1.38828   0.06282
 P                  2.04047  -0.26933   0.56937
 C                  0.40938  -0.94442   0.31876
 O                  2.64528  -0.52936   1.91142
 C                 -0.71087  -0.20672   0.16735
 C                 -2.03131  -0.88454  -0.00008
 C                 -2.76218  -0.74967  -1.18924
 C                 -3.95862  -1.43863  -1.3625
 C                 -4.44466  -2.26588  -0.34922
 C                 -3.72513  -2.40449   0.8352
 C                 -2.52335  -1.71906   1.00935
 O                  2.94633  -0.89787  -0.6361
 C                  5.23017  -1.40856  -1.22861
 C                  4.03568  -1.82096  -0.38866
 H                 -0.71649   4.9705   -0.50372
 H                 -2.84557   3.79946   0.27554
 H                 -2.73842   1.32621   0.54101
 H                  2.5917    2.04441  -0.1281
 H                  0.30425  -2.02265   0.29306
 H                 -2.38319  -0.11653  -1.98412
 H                 -4.50801  -1.33634  -2.29144
 H                 -5.3772   -2.80147  -0.48545
 H                 -4.09706  -3.04535   1.62646
 H                 -1.96301  -1.82547   1.93119
 H                  6.04521  -2.1261   -1.09306
 H                  4.96974  -1.37832  -2.28917
 H                  5.58919  -0.42037  -0.93183
 H                  4.27124  -1.81753   0.67735
 H                  3.68766  -2.82026  -0.66871
 N                  0.42679   3.29858  -0.34751
```

*2-Ethoxy-7-fluoro-4-phenylpyrido[1,2-a][1,4]azaphosphinine 2-oxide (**4d**$_{meta}$)*

```
Total energy =                              -1265.40376088 a.u.
Number of imaginary frequencies =              0
Zero-point correction =                            0.277458(Hartree/Particle)
 Thermal correction to Energy =                      0.296381
 Thermal correction to Enthalpy =                    0.297326
 Thermal correction to Gibbs Free Energy =           0.227362
 Sum of electronic and zero-point Energies =          -1265.126303
 Sum of electronic and thermal Energies =             -1265.107380
 Sum of electronic and thermal Enthalpies =           -1265.106435
 Sum of electronic and thermal Free Energies =        -1265.176399

Charge =  0 Multiplicity = 1
 C                 -0.01504   3.23835  -0.2102
 C                 -1.29992   3.65668  -0.26773
 C                 -2.31662   2.6801   -0.08681
 C                 -2.02463   1.37888   0.08277
 N                 -0.70281   0.92362   0.08515
 C                  0.36166   1.86057   0.02758
```

```
 C                    1.68072   1.52632   0.1726
 P                    2.26235  -0.06716   0.62018
 C                    0.76695  -0.99716   0.37473
 O                    2.93627  -0.25096   1.94233
 C                   -0.46021  -0.46491   0.17915
 C                   -1.62982  -1.38402   0.01801
 C                   -2.24232  -1.55093  -1.23128
 C                   -3.28758  -2.45681  -1.38621
 C                   -3.73627  -3.20285  -0.2958
 C                   -3.13105  -3.04316   0.94844
 C                   -2.08042  -2.13958   1.10524
 O                    3.25154  -0.48856  -0.61395
 C                    5.5185   -0.81487  -1.35869
 C                    4.43847  -1.29263  -0.40651
 H                    0.7996    3.94186  -0.32111
 H                   -1.56593   4.69314  -0.43163
 H                    2.40566   2.32552   0.07904
 H                    0.83571  -2.0785    0.3962
 H                   -1.89057  -0.97861  -2.08273
 H                   -3.74767  -2.5854   -2.35933
 H                   -3.47335  -3.62148   1.799
 H                   -1.60605  -2.01649   2.07221
 H                    6.4127   -1.43577  -1.24839
 H                    5.18081  -0.87751  -2.39582
 H                    5.79051   0.22148  -1.14529
 H                    4.74952  -1.20625   0.63641
 H                    4.1766   -2.33704  -0.60645
 H                   -4.55133  -3.90697  -0.41811
 H                   -2.79556   0.64062   0.22286
 F                   -3.61034   3.07954  -0.07937
```

*2-Ethoxy-8-fluoro-4-phenylpyrido[1,2-a][1,4]azaphosphinine 2-oxide (**4d**$_{para}$)*

```
Total energy =                                  -1265.40984648 a.u.
Number of imaginary frequencies =                   0
Zero-point correction =                                0.277697(Hartree/Particle)
 Thermal correction to Energy =                          0.296545
 Thermal correction to Enthalpy =                        0.297489
 Thermal correction to Gibbs Free Energy =               0.228061
 Sum of electronic and zero-point Energies =                -1265.132150
 Sum of electronic and thermal Energies =                   -1265.113302
 Sum of electronic and thermal Enthalpies =                 -1265.112357
 Sum of electronic and thermal Free Energies =              -1265.181785

Charge =  0 Multiplicity = 1
 C                    0.07763   3.15748  -0.12374
 C                   -1.19059   3.60686  -0.15032
 C                   -2.29817   2.73143   0.01945
 C                   -2.03572   1.41336   0.15392
 N                   -0.75094   0.88241   0.13213
 C                    0.37255   1.75718   0.08371
 C                    1.66677   1.33773   0.21335
 P                    2.1521   -0.29636   0.62218
 C                    0.60028  -1.1331    0.37432
 O                    2.82136  -0.55445   1.93413
 C                   -0.59078  -0.52538   0.19545
 C                   -1.81306  -1.36958   0.01357
 C                   -2.43219  -1.47173  -1.23964
 C                   -3.52634  -2.31351  -1.41675
 C                   -4.01793  -3.05961  -0.3449
 C                   -3.40642  -2.96444   0.90287
```

```
 C                    -2.30702  -2.12515   1.08182
 O                     3.09958  -0.75424  -0.63086
 C                     5.35362  -1.19709  -1.35877
 C                     4.22144  -1.6542   -0.45826
 H                     0.90951   3.84031  -0.22606
 H                    -3.31165   3.10415   0.04481
 H                     2.44022   2.09193   0.13676
 H                     0.60542  -2.21688   0.37477
 H                    -2.04657  -0.90057  -2.07715
 H                    -3.99055  -2.39341  -2.39316
 H                    -3.78118  -3.544     1.7388
 H                    -1.82723  -2.05333   2.05129
 H                     6.19625  -1.89047  -1.27794
 H                     5.03315  -1.16371  -2.4027
 H                     5.6983   -0.20136  -1.07033
 H                     4.51666  -1.65816   0.59284
 H                     3.88781  -2.66067  -0.73207
 H                    -4.8703   -3.71457  -0.48471
 H                    -2.82662   0.69421   0.2856
 F                    -1.45225   4.91812  -0.32458
```

*2-Ethoxy-7-methoxy-4-phenylpyrido[1,2-a][1,4]azaphosphinine 2-oxide (**4f**$_{meta}$)*

```
Total energy =                               -1280.69116953 a.u.
Number of imaginary frequencies =                0
Zero-point correction =                              0.317688(Hartree/Particle)
 Thermal correction to Energy =                        0.338521
 Thermal correction to Enthalpy =                      0.339465
 Thermal correction to Gibbs Free Energy =             0.265170
 Sum of electronic and zero-point Energies =              -1280.373481
 Sum of electronic and thermal Energies =                 -1280.352649
 Sum of electronic and thermal Enthalpies =               -1280.351704
 Sum of electronic and thermal Free Energies =            -1280.426000

Charge =  0 Multiplicity = 1
 C                     0.41612  -2.98313  -0.4502
 C                     1.7576   -3.13004  -0.53058
 C                     2.59259  -1.99696  -0.26419
 C                     2.02226  -0.79881  -0.01032
 N                     0.63662  -0.60831  -0.00533
 C                    -0.22304  -1.72963  -0.11214
 C                    -1.57934  -1.67278   0.06553
 P                    -2.44978  -0.25498   0.6198
 C                    -1.17756   0.96634   0.39736
 O                    -3.10869  -0.28217   1.96273
 C                     0.12559   0.6983    0.15471
 C                     1.08066   1.83948   0.00209
 C                     1.65726   2.12866  -1.24217
 C                     2.48834   3.23449  -1.39259
 C                     2.75898   4.06206  -0.30215
 C                     2.1893    3.78132   0.93735
 C                     1.3518    2.67661   1.08918
 O                    -3.54045   0.03584  -0.56714
 C                    -5.86176  -0.21766  -1.17145
 C                    -4.87462   0.52438  -0.28935
 H                    -0.2456   -3.82078  -0.62962
 H                     2.20208  -4.08174  -0.79581
 H                    -2.13532  -2.59121  -0.0777
 H                    -1.46262   2.0099    0.46146
```

H	1.4452	1.49113	-2.09344
H	2.92066	3.45377	-2.36225
H	2.39378	4.42114	1.78829
H	0.90543	2.45838	2.05268
H	-6.87123	0.17358	-1.0118
H	-5.60837	-0.09776	-2.22746
H	-5.86509	-1.2844	-0.93523
H	-5.09372	0.38171	0.77068
H	-4.88716	1.59711	-0.50866
H	3.40774	4.92241	-0.42077
H	2.62688	0.07187	0.17869
O	3.96312	-2.08182	-0.33393
C	4.57579	-2.89797	0.66903
H	4.20409	-3.92749	0.6358
H	5.64364	-2.89544	0.45465
H	4.39842	-2.48206	1.66678

*2-Ethoxy-8-methoxy-4-phenylpyrido[1,2-a][1,4]azaphosphinine 2-oxide (**4f**$_{para}$)*

```
Total energy =                              -1280.69804066 a.u.
Number of imaginary frequencies =              0
Zero-point correction =                           0.317989(Hartree/Particle)
 Thermal correction to Energy =                     0.338781
 Thermal correction to Enthalpy =                   0.339725
 Thermal correction to Gibbs Free Energy =          0.265804
 Sum of electronic and zero-point Energies =           -1280.380052
 Sum of electronic and thermal Energies =              -1280.359260
 Sum of electronic and thermal Enthalpies =            -1280.358316
 Sum of electronic and thermal Free Energies =         -1280.432237
```

Charge = 0 Multiplicity = 1

C	1.15954	-2.64932	-0.06857
C	2.50997	-2.49259	-0.07768
C	3.05306	-1.17777	0.10327
C	2.20162	-0.13397	0.21693
N	0.82201	-0.25651	0.1682
C	0.2432	-1.5569	0.1177
C	-1.10389	-1.78534	0.22833
P	-2.29464	-0.56827	0.62237
C	-1.31438	0.89851	0.37118
O	-3.0228	-0.63353	1.92828
C	0.02464	0.91523	0.20884
C	0.71473	2.22912	0.01741
C	1.25771	2.57976	-1.22647
C	1.83235	3.8331	-1.41592
C	1.87682	4.75052	-0.36528
C	1.33885	4.40968	0.87334
C	0.75814	3.156	1.06393
O	-3.34493	-0.61181	-0.63642
C	-5.54646	-1.14757	-1.44458
C	-4.74834	-0.3041	-0.46795
H	0.73701	-3.6398	-0.16901
H	4.11414	-0.988	0.15757
H	-1.43539	-2.81367	0.15461
H	-1.82596	1.85386	0.35456
H	1.21584	1.87306	-2.0482
H	2.23778	4.09744	-2.38606
H	1.36713	5.11855	1.69308
H	0.33489	2.89098	2.02613
H	-6.61062	-0.90606	-1.36279
H	-5.22808	-0.96095	-2.47296

```
 H                    -5.41668  -2.21099  -1.23052
 H                    -5.03532  -0.50121   0.56693
 H                    -4.89056   0.76341  -0.6701
 H                     2.32376   5.72677  -0.51486
 H                     2.56837   0.8697    0.35355
 O                     3.27854  -3.59997  -0.24404
 C                     4.69787  -3.47289  -0.23856
 H                     5.08136  -4.47966  -0.39334
 H                     5.04577  -2.82666  -1.05058
 H                     5.06311  -3.09342   0.72093
```

*Methyl 2-ethoxy-4-phenylpyrido[1,2-a][1,4]azaphosphinine-7-carboxylate 2-oxide (**4g**$_{meta}$)*

```
Total energy =                              -1394.09090430 a.u.
Number of imaginary frequencies =               0
Zero-point correction =                            0.328491(Hartree/Particle)
 Thermal correction to Energy =                      0.351123
 Thermal correction to Enthalpy =                    0.352067
 Thermal correction to Gibbs Free Energy =           0.273409
 Sum of electronic and zero-point Energies =            -1393.762414
 Sum of electronic and thermal Energies =               -1393.739781
 Sum of electronic and thermal Enthalpies =             -1393.738837
 Sum of electronic and thermal Free Energies =          -1393.817496

Charge =  0 Multiplicity = 1
 C                     0.57062  -2.77093  -0.24659
 C                     1.9132   -2.63204  -0.26486
 C                     2.48153  -1.33349  -0.03181
 C                     1.64344  -0.27573   0.13163
 N                     0.26886  -0.3809    0.09336
 C                    -0.33516  -1.66964   0.00534
 C                    -1.67495  -1.88756   0.14198
 P                    -2.84053  -0.664     0.61815
 C                    -1.84748   0.79498   0.37771
 O                    -3.51147  -0.77692   1.94897
 C                    -0.5128    0.80315   0.18308
 C                     0.19063   2.11108   0.01034
 C                     0.74183   2.47177  -1.22678
 C                     1.32872   3.72177  -1.39677
 C                     1.37726   4.62399  -0.33377
 C                     0.83203   4.27213   0.8984
 C                     0.23836   3.02236   1.07019
 O                    -3.92519  -0.66243  -0.60522
 C                    -6.1529   -1.13685  -1.38055
 C                    -5.32255  -0.34658  -0.38744
 H                     0.10917  -3.73906  -0.39332
 H                     2.56321  -3.4789   -0.43662
 H                    -2.02336  -2.90743   0.03362
 H                    -2.34845   1.75587   0.39469
 H                     0.70035   1.77629  -2.05801
 H                     1.74499   3.99322  -2.36009
 H                     0.86829   4.96791   1.72882
 H                    -0.18678   2.74721   2.02866
 H                    -7.21159  -0.8882   -1.26035
 H                    -5.85862  -0.90627  -2.40705
 H                    -6.03039  -2.21015  -1.21808
 H                    -5.5854   -0.5873    0.64452
 H                    -5.45475   0.73021  -0.53788
 H                     1.83777   5.59613  -0.46734
 H                     2.04354   0.71138   0.29821
```

C	3.93503	-1.06659	0.03942
O	4.43644	0.01753	0.24301
O	4.65893	-2.19186	-0.15472
C	6.08885	-2.0347	-0.10431
H	6.49535	-3.02878	-0.27668
H	6.42313	-1.3424	-0.87806
H	6.39596	-1.65687	0.87164

Methyl 2-ethoxy-4-phenylpyrido[1,2-a][1,4]azaphosphinine-8-carboxylate 2-oxide ($\mathbf{4g}_{para}$*)*

Total energy = -1394.06947476 a.u.
Number of imaginary frequencies = 0
Zero-point correction = 0.327873(Hartree/Particle)
Thermal correction to Energy = 0.350675
Thermal correction to Enthalpy = 0.351620
Thermal correction to Gibbs Free Energy = 0.272440
Sum of electronic and zero-point Energies = -1393.741602
Sum of electronic and thermal Energies = -1393.718799
Sum of electronic and thermal Enthalpies = -1393.717855
Sum of electronic and thermal Free Energies = -1393.797035

Charge = 0 Multiplicity = 1

C	-2.22994	-0.95175	0.18724
C	-3.10866	0.08112	0.18876
C	-2.60395	1.41023	-0.01159
C	-1.2788	1.59601	-0.1704
N	-0.34945	0.55554	-0.13051
C	-0.80973	-0.78187	-0.02535
C	0.00187	-1.87926	-0.11508
P	1.70081	-1.82586	-0.55675
C	1.99036	-0.08143	-0.36737
O	2.12481	-2.42317	-1.86021
C	1.02974	0.85646	-0.21281
C	1.43687	2.29016	-0.07835
C	1.32041	2.95427	1.15037
C	1.76835	4.26488	1.28566
C	2.33392	4.92888	0.19647
C	2.45419	4.27441	-1.02704
C	2.01011	2.95953	-1.16421
O	2.46067	-2.53589	0.70396
C	3.56699	-4.54037	1.46079
C	3.65192	-3.34396	0.53206
H	-2.55752	-1.96978	0.35
H	-3.2743	2.2574	-0.02583
H	-0.46114	-2.8501	0.01322
H	3.01761	0.26369	-0.38115
H	0.88892	2.43915	2.00167
H	1.68263	4.76582	2.24318
H	2.89472	4.78384	-1.87645
H	2.10639	2.44726	-2.11474
H	4.47935	-5.13892	1.37971
H	3.45489	-4.22145	2.49962
H	2.71599	-5.17333	1.19873
H	3.72809	-3.64784	-0.51374
H	4.51553	-2.71824	0.77939
H	2.68176	5.9499	0.30403
H	-0.85908	2.57476	-0.33151
C	-4.56154	-0.07974	0.52894
O	-5.26282	-1.11674	0.01538
O	-5.10566	0.68581	1.28234
C	-4.87444	-1.81538	-1.18174

```
H                    -4.23583  -1.20452  -1.81963
H                    -4.3634   -2.74584  -0.92848
H                    -5.80273  -2.04592  -1.70288
```

*3-Ethoxy-1-phenyl-[1,4]azaphosphinino[1,2-a]quinoline 3-oxide (**4h**)*

```
Total energy =                              -1319.82173854 a.u.
Number of imaginary frequencies =               0
Zero-point correction =                            0.332314(Hartree/Particle)
 Thermal correction to Energy =                      0.352808
 Thermal correction to Enthalpy =                    0.353753
 Thermal correction to Gibbs Free Energy =           0.281424
 Sum of electronic and zero-point Energies =            -1319.489424
 Sum of electronic and thermal Energies =               -1319.468930
 Sum of electronic and thermal Enthalpies =             -1319.467986
 Sum of electronic and thermal Free Energies =          -1319.540314

Charge =  0 Multiplicity = 1
 C                    -1.22907   1.34805  -0.41343
 C                     0.00216   0.80038  -0.29023
 N                     0.21958  -0.59269  -0.40994
 C                    -0.64207  -1.38254  -1.21198
 C                    -1.92522  -1.02991  -1.48911
 P                    -2.69481   0.38484  -0.75801
 C                     1.27862  -1.23113   0.28944
 C                     1.791    -2.45009  -0.20801
 C                     1.12761  -3.0694   -1.33104
 C                    -0.04667  -2.58199  -1.77354
 O                    -3.74287   1.1086   -1.53576
 O                    -3.25067  -0.20358   0.66461
 C                    -4.23566   0.52673   1.43397
 C                    -4.84743  -0.42628   2.44226
 C                     1.20118   1.68561  -0.20734
 C                     2.31177   1.46239  -1.03228
 C                     3.38657   2.34539  -1.02456
 C                     3.37443   3.4611   -0.18791
 C                     2.27553   3.69041   0.63772
 C                     1.19678   2.80965   0.62757
 C                     1.80149  -0.70014   1.47334
 C                     2.86714  -1.32408   2.11099
 C                     3.42217  -2.49557   1.59121
 C                     2.87377  -3.05574   0.44758
 H                    -1.31159   2.42626  -0.47565
 H                    -2.48981  -1.62193  -2.19868
 H                     1.55135  -3.97166  -1.75802
 H                    -0.61283  -3.08592  -2.54705
 H                    -3.7396    1.36338   1.93943
 H                    -4.9885    0.93157   0.75364
 H                    -5.58691   0.10139   3.05187
 H                    -5.34577  -1.2549    1.93436
 H                    -4.08232  -0.83695   3.10516
 H                     2.32746   0.60225  -1.69113
 H                     4.23351   2.16465  -1.67678
 H                     4.21447   4.14621  -0.1806
 H                     2.2585    4.55296   1.29437
 H                     0.3486    2.98565   1.27954
 H                     1.37345   0.1946    1.90167
 H                     3.25817  -0.89634   3.02687
 H                     4.25578  -2.97513   2.09016
 H                     3.26489  -3.987     0.05175
```

*3-Ethoxy-1-phenyl-[1,4]azaphosphinino[1,2-a]quinoxaline 3-oxide (**4i**)*

```
Total energy =                          -1335.86316024 a.u.
Number of imaginary frequencies =            0
Zero-point correction =                        0.320541(Hartree/Particle)
 Thermal correction to Energy =                  0.340936
 Thermal correction to Enthalpy =                0.341880
 Thermal correction to Gibbs Free Energy =       0.269213
 Sum of electronic and zero-point Energies =        -1335.542619
 Sum of electronic and thermal Energies =           -1335.522224
 Sum of electronic and thermal Enthalpies =         -1335.521280
 Sum of electronic and thermal Free Energies =      -1335.593947

Charge =  0 Multiplicity = 1
 N                    -1.6197   -3.0024   -1.18784
 C                    -0.35102  -2.81039  -1.20127
 C                    -2.37676  -2.15624  -0.37677
 C                    -1.82162  -1.01357   0.23899
 N                    -0.47343  -0.68096  -0.06606
 C                     0.35342  -1.72954  -0.51592
 C                     0.05347   0.61269   0.12475
 C                     1.37231   0.82268   0.35468
 P                     2.558    -0.49089   0.53285
 C                     1.70637  -1.73498  -0.40664
 O                     3.00646  -0.86847   1.90597
 O                     3.78529   0.02386  -0.40903
 C                     5.17248  -0.2269   -0.0663
 C                     6.00675   0.9378   -0.56279
 C                    -0.84568   1.78     -0.10788
 C                    -0.7979    2.89092   0.74267
 C                    -1.57066   4.01899   0.47727
 C                    -2.39738   4.05339  -0.64396
 C                    -2.44711   2.95249  -1.4988
 C                    -1.68122   1.82238  -1.23197
 C                    -3.71282  -2.49534  -0.12947
 C                    -4.48429  -1.74442   0.74383
 C                    -3.91108  -0.65053   1.39649
 C                    -2.59099  -0.28862   1.15396
 H                     0.26881  -3.5028   -1.76774
 H                     1.73722   1.84193   0.35657
 H                     2.2568   -2.54377  -0.87308
 H                     5.2545   -0.35568   1.01465
 H                     5.47589  -1.16032  -0.55121
 H                     7.06377   0.75364  -0.34901
 H                     5.89106   1.0701   -1.64094
 H                     5.71152   1.86498  -0.06625
 H                    -0.16417   2.86212   1.62188
 H                    -1.52889   4.86864   1.14932
 H                    -2.99874   4.93119  -0.8511
 H                    -3.08164   2.97478  -2.3775
 H                    -1.72246   0.97391  -1.90493
 H                    -4.1058   -3.37306  -0.62816
 H                    -5.51481  -2.01777   0.93625
 H                    -4.49092  -0.07811   2.11113
 H                    -2.16172   0.54894   1.68386
```

*2-Ethoxy-4-phenyl-[1,4]azaphosphinino[2,1-a]isoquinoline 2-oxide (**4j**)*

```
Total energy =                             -1319.82654355 a.u.
Number of imaginary frequencies =              0
Zero-point correction =                           0.332736(Hartree/Particle)
 Thermal correction to Energy =                    0.353314
 Thermal correction to Enthalpy =                  0.354258
 Thermal correction to Gibbs Free Energy =         0.281369
 Sum of electronic and zero-point Energies =          -1319.493808
 Sum of electronic and thermal Energies =             -1319.473229
 Sum of electronic and thermal Enthalpies =           -1319.472285
 Sum of electronic and thermal Free Energies =        -1319.545175

Charge =  0 Multiplicity = 1
 C                  4.91233   0.69611  -0.62993
 C                  5.05195   1.82551   0.18413
 C                  3.94284   2.34588   0.82926
 C                  2.68045   1.74488   0.68549
 C                  2.54195   0.58294  -0.10637
 C                  3.67466   0.08562  -0.77059
 C                  1.50002   2.28894   1.30833
 C                  0.28991   1.78627   1.01774
 N                  0.10521   0.68615   0.16924
 C                  1.22894  -0.09441  -0.20547
 C                 -1.21279   0.32246  -0.18519
 C                 -1.56059  -0.90781  -0.62248
 P                 -0.41983  -2.25303  -0.79877
 C                  1.10771  -1.37725  -0.63684
 O                 -0.5448   -3.11923  -2.00953
 O                 -0.66319  -3.10139   0.58248
 C                 -0.37562  -4.51785   0.66126
 C                 -1.2337   -5.11753   1.75872
 C                 -2.25077   1.3948   -0.12338
 C                 -3.42777   1.19054   0.60514
 C                 -4.43228   2.15719   0.61065
 C                 -4.27313   3.33558  -0.1147
 C                 -3.10343   3.54581  -0.84614
 C                 -2.09695   2.58542  -0.84801
 H                  5.77006   0.29681  -1.1585
 H                  6.02062   2.29844   0.29841
 H                  4.03445   3.23181   1.4485
 H                  3.58707  -0.77562  -1.4199
 H                  1.57635   3.12932   1.98591
 H                 -0.6163    2.19898   1.43013
 H                 -2.58023  -1.06461  -0.95181
 H                  2.00076  -1.91195  -0.92932
 H                 -0.58034  -4.97702  -0.30837
 H                  0.68899  -4.64329   0.88789
 H                 -1.01461  -6.18459   1.86122
 H                 -1.03707  -4.63142   2.71718
 H                 -2.29449  -5.00411   1.52379
 H                 -3.54904   0.27513   1.17278
 H                 -5.33687   1.98848   1.18378
 H                 -5.0554    4.08603  -0.11296
 H                 -2.97836   4.45598  -1.42164
 H                 -1.19305   2.75047  -1.42361
```

*2,11-Diethoxy-4,9-diphenylpyrazino[1,2-a:4,3-a']bis([1,4]azaphosphinine) 2,11-dioxide (**4l**)*

```
Total energy =                             -2099.98632008 a.u.
Number of imaginary frequencies =              0
Zero-point correction =                           0.471728(Hartree/Particle)
 Thermal correction to Energy =                    0.503599
```

Thermal correction to Enthalpy = 0.504543
Thermal correction to Gibbs Free Energy = 0.403435
Sum of electronic and zero-point Energies = -2099.514592
Sum of electronic and thermal Energies = -2099.482722
Sum of electronic and thermal Enthalpies = -2099.481777
Sum of electronic and thermal Free Energies = -2099.582885

Charge = 0 Multiplicity = 1

C	0.75193	-0.7415	-0.02191
N	1.37113	0.46507	0.34333
C	0.62078	1.64387	0.17178
C	-0.66035	1.62966	-0.19743
N	-1.38767	0.43521	-0.35376
C	-0.7471	-0.75419	0.03298
C	1.43287	-1.85665	-0.36966
P	3.20149	-1.98488	-0.3656
C	3.62367	-0.44409	0.41244
C	2.73858	0.54777	0.66301
C	-2.75347	0.48615	-0.68575
C	-3.62481	-0.51162	-0.41314
P	-3.18446	-2.01604	0.41401
C	-1.41092	-1.87341	0.39963
O	-3.71986	-3.29517	-0.13772
O	-3.67984	-1.74677	1.94875
C	-4.0092	-2.84321	2.83908
C	-4.92257	-2.31162	3.92612
O	3.79472	-3.20526	0.25686
O	3.58875	-1.8495	-1.94819
C	4.72465	-2.54583	-2.52079
C	4.43694	-2.80157	-3.98742
C	-3.21798	1.69317	-1.43066
C	-4.30275	2.43857	-0.956
C	-4.78261	3.52706	-1.68257
C	-4.18707	3.8781	-2.89202
C	-3.10658	3.13789	-3.37317
C	-2.62108	2.05536	-2.64639
C	3.18818	1.78162	1.37296
C	2.60749	2.15503	2.59311
C	3.0799	3.26483	3.2868
C	4.13149	4.02112	2.76818
C	4.71117	3.65882	1.55439
C	4.2439	2.54366	0.86077
H	1.14341	2.57259	0.31926
H	-1.20046	2.54658	-0.35609
H	0.88017	-2.74849	-0.63184
H	4.63685	-0.3245	0.77648
H	-4.63853	-0.4229	-0.7834
H	-0.84183	-2.75517	0.66166
H	-4.48551	-3.63764	2.26092
H	-3.07944	-3.23388	3.26632
H	-5.1686	-3.11333	4.62859
H	-4.44033	-1.50328	4.48067
H	-5.85236	-1.93136	3.49704
H	5.61006	-1.91278	-2.39979
H	4.88597	-3.47446	-1.96992
H	5.29283	-3.30079	-4.45136
H	3.55981	-3.44215	-4.10291
H	4.25407	-1.86497	-4.51926
H	-4.76303	2.16545	-0.01346
H	-5.62079	4.09952	-1.30197
H	-4.56291	4.72221	-3.45888

```
 H                    -2.64607   3.39994  -4.31891
 H                    -1.7867    1.47763  -3.02775
 H                     1.79538   1.56541   3.00333
 H                     2.6318    3.53574   4.23601
 H                     4.49725   4.8863    3.30925
 H                     5.52689   4.24352   1.14464
 H                     4.69188   2.26258  -0.08538
```

*2-Ethoxy-4-phenyl-1H-isophosphinoline 2-oxide (**II**)*

```
Total energy =                                   -1150.12965717 a.u.
Number of imaginary frequencies =                   0
Zero-point correction =                                0.297849(Hartree/Particle)
 Thermal correction to Energy =                          0.315803
 Thermal correction to Enthalpy =                        0.316747
 Thermal correction to Gibbs Free Energy =               0.250312
 Sum of electronic and zero-point Energies =                -1149.831809
 Sum of electronic and thermal Energies =                   -1149.813854
 Sum of electronic and thermal Enthalpies =                 -1149.812910
 Sum of electronic and thermal Free Energies =              -1149.879345

Charge =  0 Multiplicity = 1
 C                    -11.48644    1.62417   -0.39209
 C                    -11.5957     0.3017    -0.22391
 C                    -10.49985   -0.46876   -0.29148
 C                    -9.26534     0.02436   -0.53739
 C                    -9.17357     1.36917   -0.6772
 C                    -10.27276    2.14424   -0.61281
 C                    -8.18472    -0.80153   -0.56762
 C                    -6.96952    -0.30389   -0.24709
 P                    -6.67383     1.42415    0.33008
 C                    -7.85464     2.04846   -0.95721
 O                    -5.28796     1.80334   -0.04463
 C                    -8.26816    -2.1129    -0.92298
 C                    -7.38881    -3.0506    -0.50516
 C                    -7.46986    -4.33859   -0.87667
 C                    -8.4389     -4.74305   -1.70773
 C                    -9.31372    -3.8371    -2.16258
 C                    -9.2185     -2.55462   -1.77707
 O                    -7.11046     1.65876    1.85269
 C                    -7.75428     2.91498    3.77497
 C                    -7.36491     2.96601    2.30043
 H                    -12.37599    2.27283   -0.33358
 H                    -12.58108   -0.14419   -0.00678
 H                    -10.66546   -1.53706   -0.07145
 H                    -10.19173    3.2362    -0.74421
 H                    -6.04906    -0.89985   -0.34048
 H                    -7.89802     3.15744   -0.87472
 H                    -7.49641     1.79164   -1.98007
 H                    -6.57976    -2.82132    0.20722
 H                    -6.74204    -5.07378   -0.49263
 H                    -8.50746    -5.79782   -2.02093
 H                    -10.09956   -4.14686   -2.87243
 H                    -9.93723    -1.8638    -2.24989
 H                    -7.96388     3.9366     4.16766
 H                    -8.66963     2.2977     3.92646
 H                    -6.93679     2.47461    4.39093
 H                    -6.44949     3.59022    2.17063
 H                    -8.20051     3.40864    1.70852
```

Frontier MOs of compounds 2a, 2b, 2n, 2p, 2q, 2r, 2t, 4a, 4c, 4d, 4f, 4g, 4h, 4i, 4j, 4l, and II

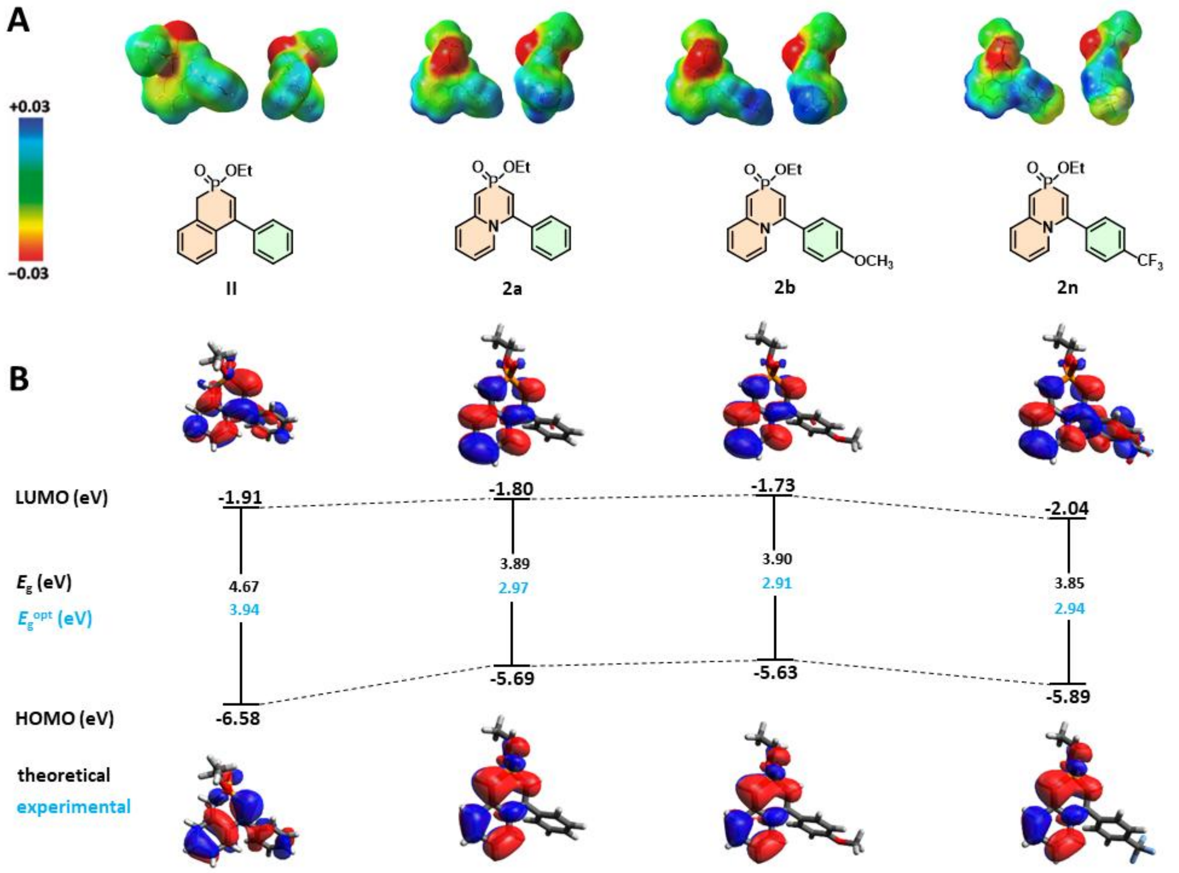

Figure S272 *(A) The electrostatic potentials mapped onto the electron density surface in the range from – 0.03 (red) to 0.03 (blue). (B) Calculated HOMO/LUMO orbitals and energy gaps (Eg) (DFT, B3LYP/6-311G++(d,p)) and experimental values of optical gap energies (E_g^{opt} = 1240/λ^{edge}) (in blue) for series compounds* ***II****,* ***2a****,* ***2b****, and* ***2n****.*

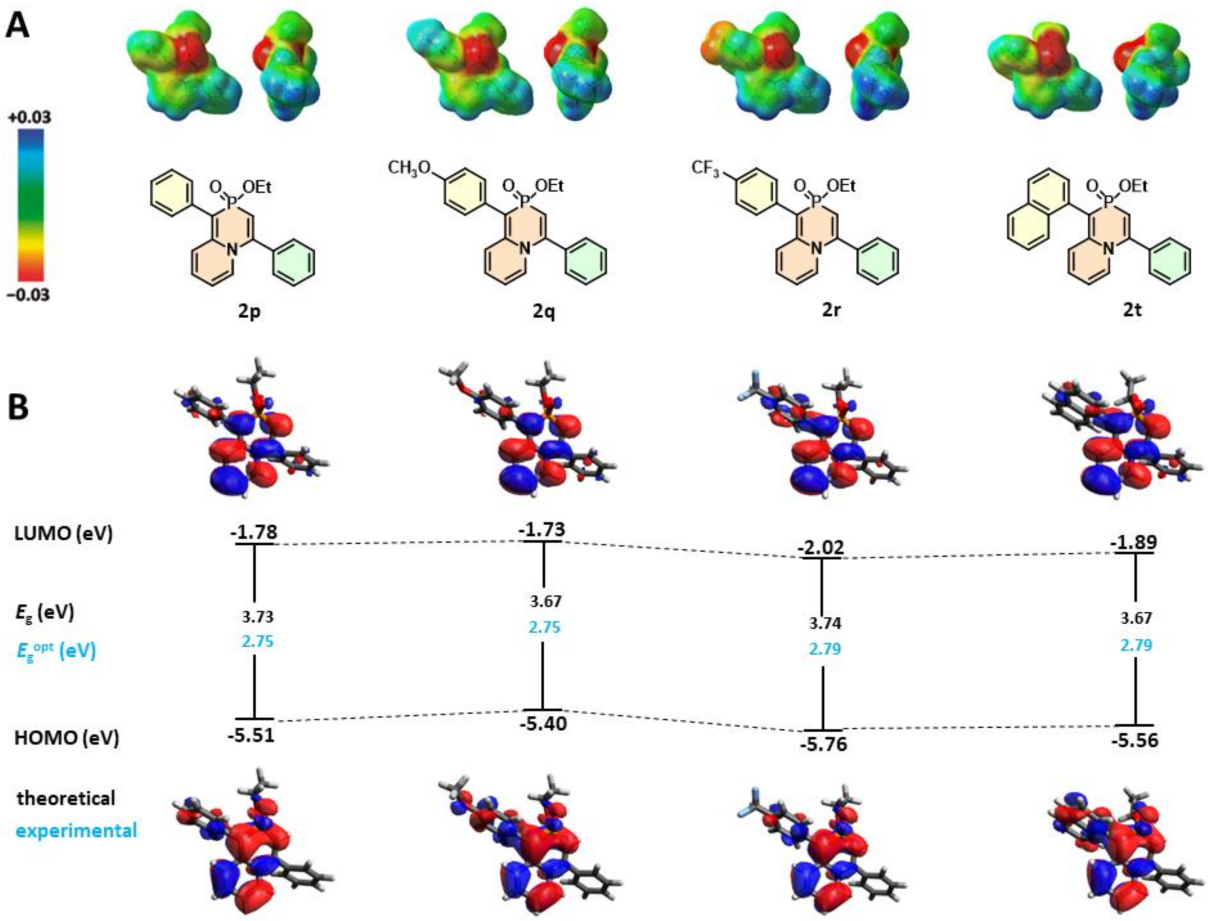

Figure S273 *(A) The electrostatic potentials mapped onto the electron density surface in the range from – 0.03 (red) to 0.03 (blue). (B) Calculated HOMO/LUMO orbitals and energy gaps (Eg) (DFT, B3LYP/6-311G++(d,p)) and experimental values of optical gap energies (E_g^{opt} = 1240/λ^{edge}) (in blue) for series compounds* ***2p****,* ***2q****,* ***2r****, and* ***2t****.*

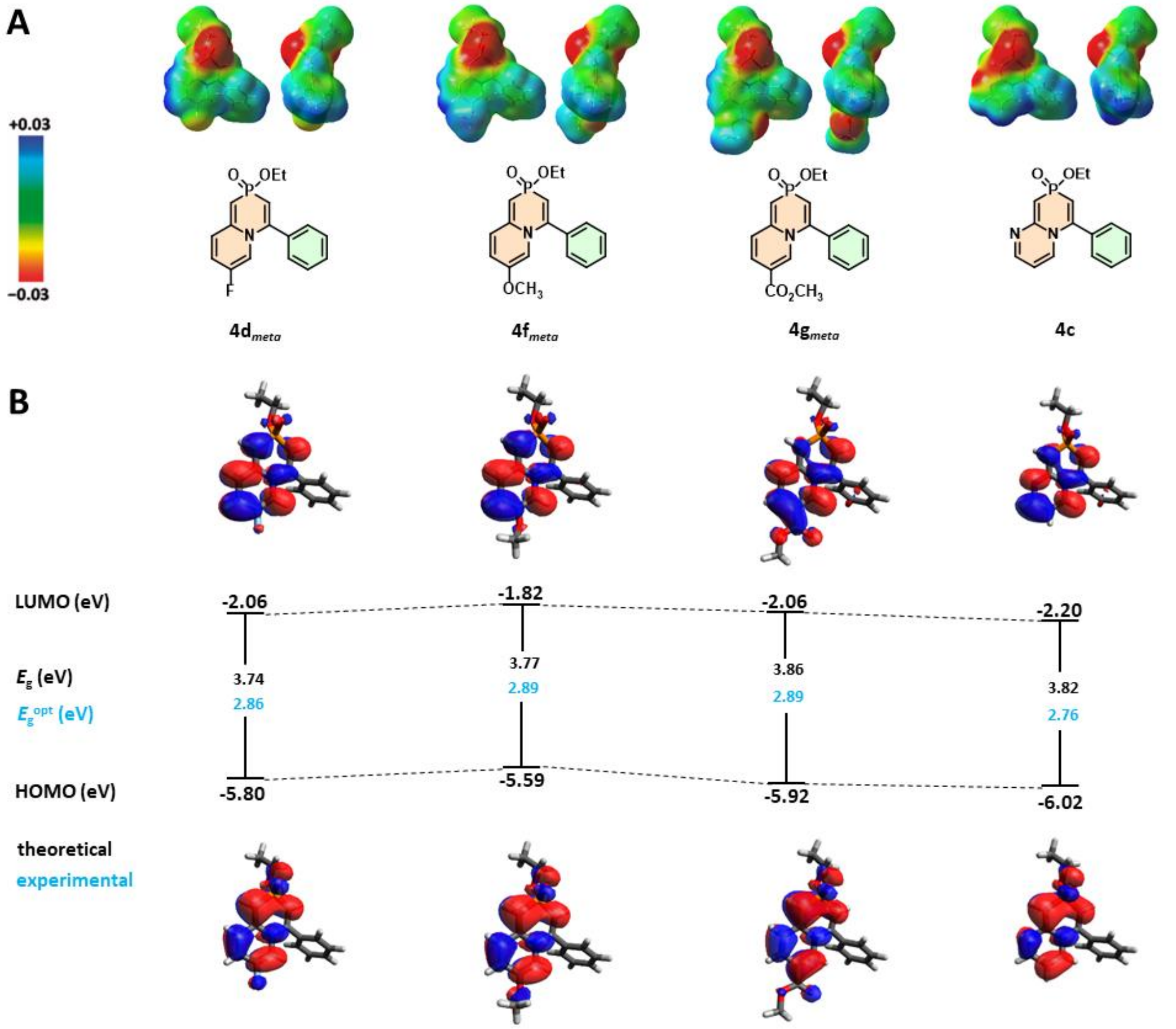

Figure S274 *(A) The electrostatic potentials mapped onto the electron density surface in the range from – 0.03 (red) to 0.03 (blue). (B) Calculated HOMO/LUMO orbitals and energy gaps (*Eg*) (DFT, B3LYP/6-311G++(d,p)) and experimental values of optical gap energies ($_g^{opt}$ = 1240/λ^{edge}) (in blue) for series compounds* ***4d***$_{\mathbf{meta}}$*,* ***4f***$_{\mathbf{meta}}$*,* ***4g***$_{\mathbf{meta}}$*, and* ***4c****.*

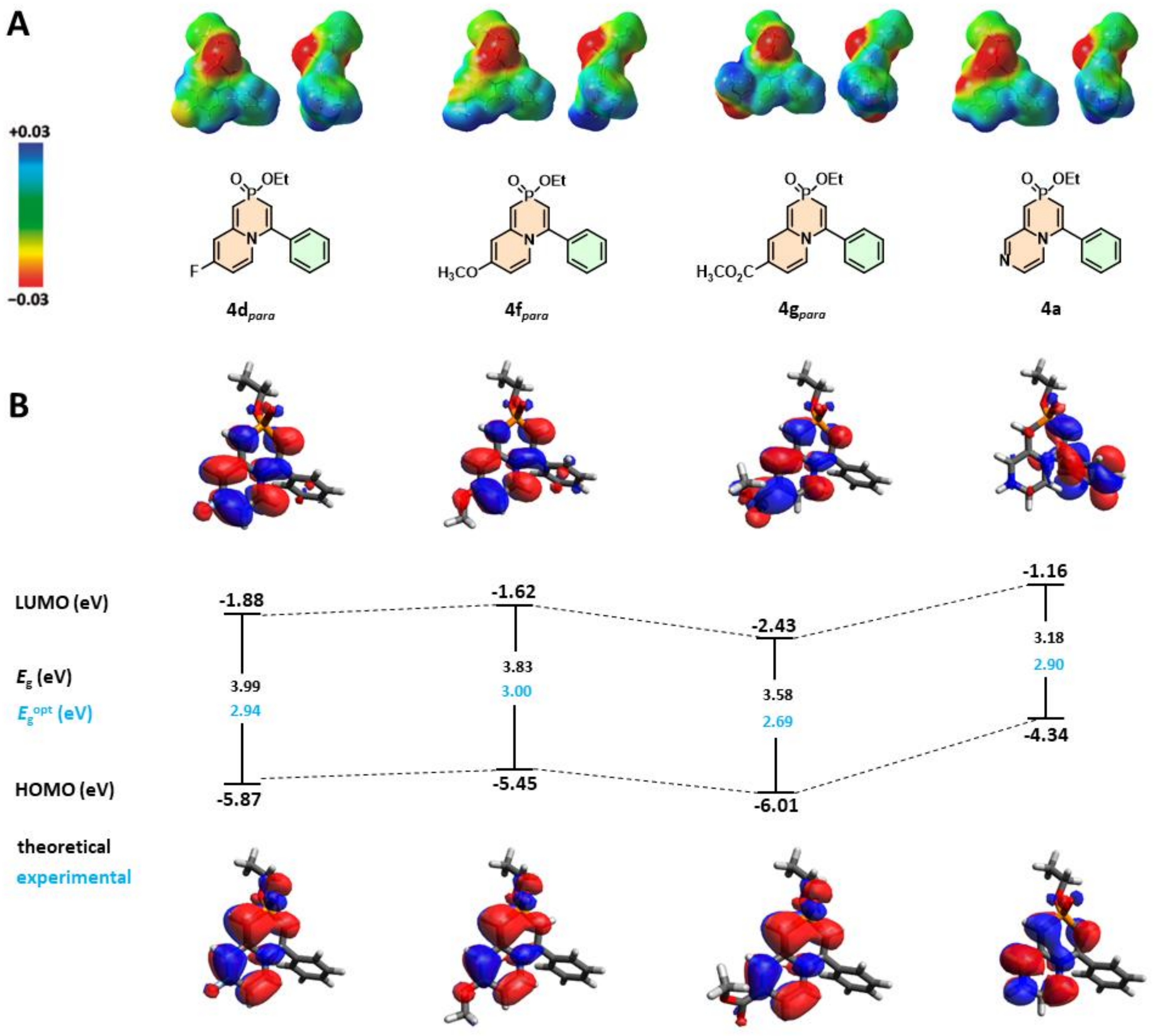

Figure S275 *(A) The electrostatic potentials mapped onto the electron density surface in the range from – 0.03 (red) to 0.03 (blue). (B) Calculated HOMO/LUMO orbitals and energy gaps (Eg) (DFT, B3LYP/6-311G++(d,p)) and experimental values of optical gap energies (E_g^{opt} = 1240/λ^{edge}) (in blue) for series compounds* ***4d$_{para}$, 4f$_{para}$, 4g$_{para}$,*** *and* ***4a****.*

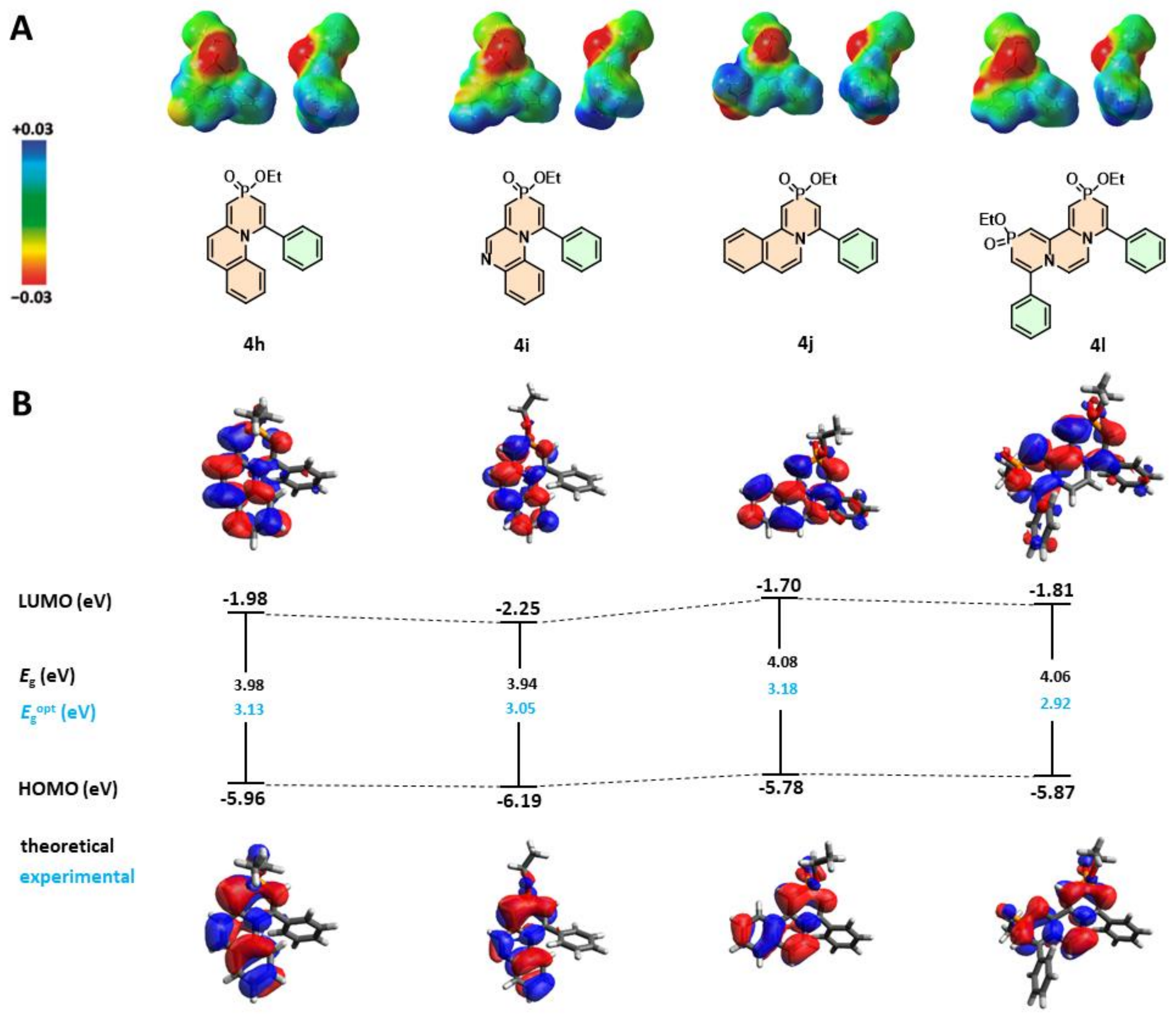

Figure S276 *(A) The electrostatic potentials mapped onto the electron density surface in the range from – 0.03 (red) to 0.03 (blue). (B) Calculated HOMO/LUMO orbitals and energy gaps (Eg) (DFT, B3LYP/6-311G++(d,p)) and experimental values of optical gap energies ($E_g^{opt} = 1240/\lambda^{edge}$) (in blue) for series compounds **4h**, **4i**, **4j**, and **4l**.*

*The vertical energy electronic transitions, corresponding wavelengths with oscillator strength (f), and their major orbital contributions for compounds **2a**, **2b**, **2n**, **2p**, **2q**, **2r**, **2t**, **4a**, **4c**, **4d**, **4f**, **4g**, **4h**, **4i**, **4j**, **4l**, and **II***

Table S1 Calculated vertical energy electronic transition, wavelength, oscillator strength, and major orbital contributions of **2a** by the B3LYP/6-311++G(d,p) method

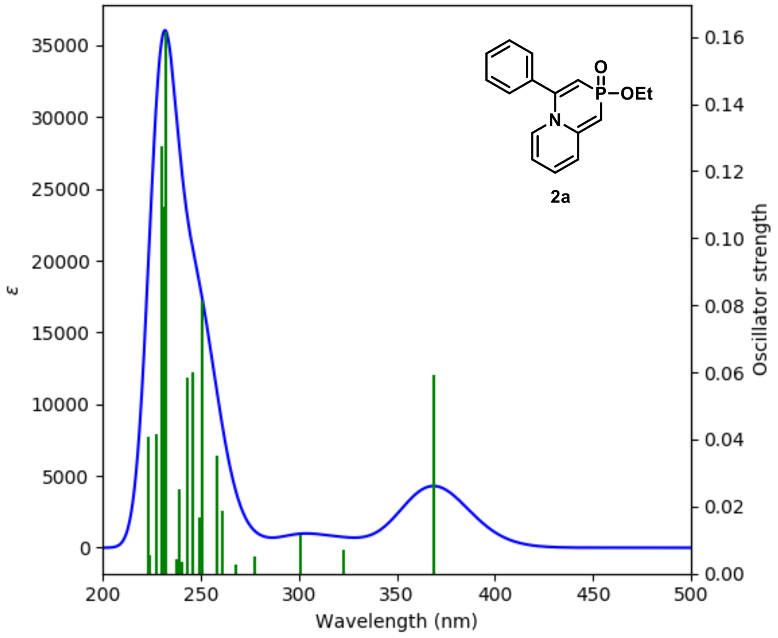

Energy (eV)	Wavelength (nm)	Osc. Strength	Major orbital contributions
5,56	223,14	0,0406	H-5->L+2 (14%), H-4->L+1 (11%), H-3->L+1 (17%), H-1->L+2 (16%)
5,54	223,79	0,0057	HOMO->L+10 (81%)
5,46	227,14	0,0417	H-2->L+1 (72%), H-1->L+2 (13%)
5,38	230,27	0,1274	H-4->LUMO (12%), HOMO->L+7 (13%), HOMO->L+8 (16%), HOMO->L+9 (39%)
5,36	231,21	0,1092	H-4->LUMO (11%), HOMO->L+8 (30%), HOMO->L+9 (35%)
**5,34**	**232,09**	**0,1616**	H-4->LUMO (24%), HOMO->L+8 (48%)
5,22	237,38	0,0044	H-5->LUMO (88%)
5,19	238,69	0,0252	H-4->LUMO (24%), H-3->LUMO (39%)
5,16	240,06	0,0033	HOMO->L+7 (74%), HOMO->L+9 (15%)
5,09	243,37	0,0586	H-1->L+1 (21%), H-1->L+2 (43%)
5,05	245,62	0,0602	H-3->LUMO (32%), H-1->L+1 (48%)
4,97	249,49	0,0167	HOMO->L+3 (12%), HOMO->L+5 (48%), HOMO->L+6 (21%)
**4,94**	**250,88**	**0,0814**	H-2->LUMO (26%), H-1->L+1 (11%), HOMO->L+4 (21%), HOMO->L+6 (10%)
4,80	258,12	0,0353	H-2->LUMO (47%), HOMO->L+5 (14%), HOMO->L+6 (23%)
4,75	260,89	0,0188	H-2->LUMO (13%), HOMO->L+4 (37%), HOMO->L+5 (11%), HOMO->L+6 (33%)
4,63	268,04	0,0027	HOMO->L+3 (82%), HOMO->L+5 (13%)
4,46	277,72	0,005	H-1->LUMO (78%), HOMO->L+4 (13%)
4,12	301,06	0,0116	HOMO->L+2 (97%)
3,84	323,11	0,0072	HOMO->L+1 (99%)
**3,36**	**368,78**	**0,0593**	**HOMO->LUMO (98%)**

Table S2 Calculated vertical energy electronic transition, wavelength, oscillator strength, and major orbital contributions of **2b** by the B3LYP/6-311++G(d,p) method

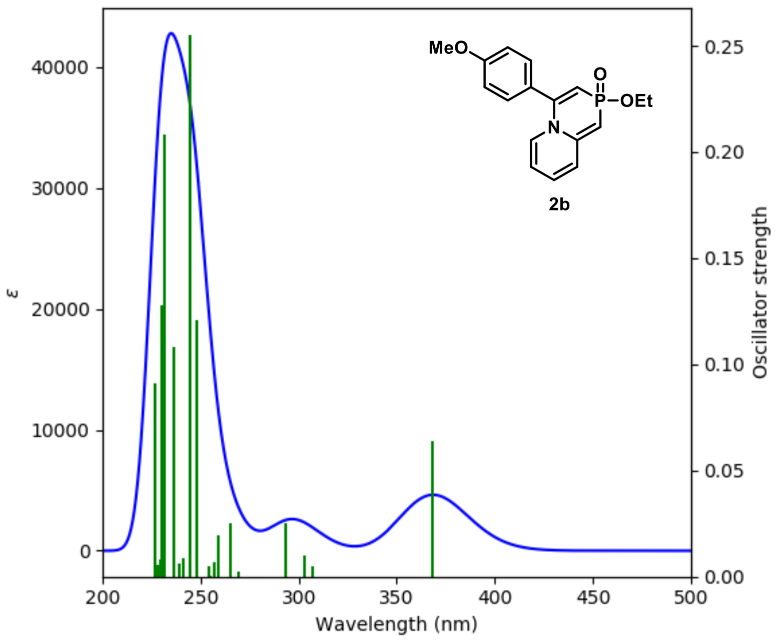

Energy (eV)	Wavelength (nm)	Osc. Strength	Major orbital contributions
5,47	226,51	0,0910	H-2->L+1 (59%)
5,43	228,24	0,0054	HOMO->L+9 (37%), HOMO->L+10 (48%)
5,40	229,56	0,0081	H-1->L+3 (93%)
5,38	230,45	0,1276	HOMO->L+8 (12%), HOMO->L+9 (30%), HOMO->L+10 (23%)
**5,35**	**231,77**	**0,2084**	H-5->LUMO (29%), HOMO->L+9 (19%)
5,25	236,23	0,1083	H-5->LUMO (42%), H-4->LUMO (34%)
5,19	238,77	0,0064	HOMO->L+7 (68%), HOMO->L+8 (21%)
5,14	241,04	0,0088	HOMO->L+7 (18%), HOMO->L+8 (52%), HOMO->L+10 (12%)
**5,07**	**244,73**	**0,2553**	H-4->LUMO (31%), H-1->L+2 (54%)
5,00	248,20	0,1207	H-4->LUMO (17%), H-2->LUMO (22%), H-1->L+2 (21%)
4,87	254,48	0,0052	H-3->LUMO (52%), HOMO->L+6 (32%)
4,86	255,04	0,0004	HOMO->L+3 (38%), HOMO->L+4 (13%), HOMO->L+5 (34%)
4,83	256,82	0,0070	H-3->LUMO (19%), H-2->LUMO (13%), HOMO->L+6 (44%)
4,79	259,02	0,0195	H-3->LUMO (10%), H-1->L+1 (75%)
4,68	264,93	0,0251	H-2->LUMO (45%), HOMO->L+4 (29%), HOMO->L+5 (16%)
4,60	269,41	0,0025	HOMO->L+3 (53%), HOMO->L+4 (22%), HOMO->L+5 (22%)
4,22	293,50	0,0250	H-1->LUMO (77%), HOMO->L+2 (16%)
4,09	302,78	0,0101	H-1->LUMO (17%), HOMO->L+1 (31%), HOMO->L+2 (50%)
4,04	307,01	0,0047	HOMO->L+1 (66%), HOMO->L+2 (32%)
**3,36**	**368,52**	**0,0638**	**HOMO->LUMO (98%)**

Table S3 Calculated vertical energy electronic transition, wavelength, oscillator strength, and major orbital contributions of **2n** by the B3LYP/6-311++G(d,p) method

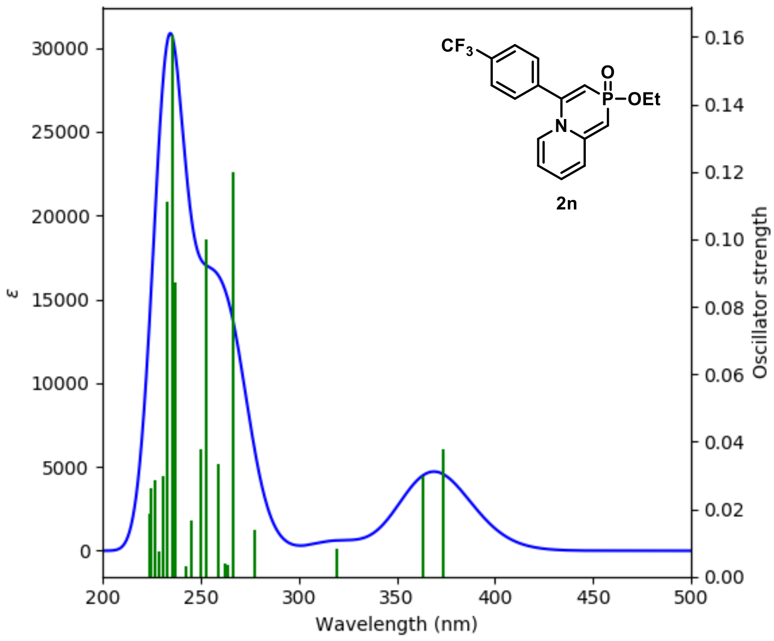

Energy (eV)	Wavelength (nm)	Osc. Strength	Major orbital contributions
5,53	224,06	0,0187	H-5->LUMO (10%), H-5->L+1 (16%), H-4->L+1 (10%), H-2->L+2 (26%), HOMO->L+9 (15%)
5,52	224,41	0,0264	H-4->LUMO (10%), H-4->L+1 (11%), H-2->L+2 (58%)
5,47	226,76	0,0284	H-5->LUMO (67%), H-4->LUMO (17%)
5,42	228,80	0,0076	HOMO->L+8 (88%)
5,37	230,78	0,0297	H-4->LUMO (44%), H-1->L+2 (17%)
5,33	232,46	0,1113	H-3->L+1 (66%)
**5,26**	**235,51**	**0,1607**	H-3->LUMO (26%), H-3->L+1 (11%), HOMO->L+7 (21%)
5,23	236,91	0,0871	H-3->LUMO (14%), HOMO->L+7 (56%)
5,12	242,33	0,0031	HOMO->L+6 (82%)
5,06	245,15	0,0165	H-2->L+1 (74%)
4,96	249,72	0,0376	H-3->LUMO (33%), H-2->LUMO (11%), H-1->L+2 (31%)
4,91	252,52	0,0999	H-2->LUMO (13%), H-2->L+1 (10%), H-1->L+2 (27%), HOMO->L+3 (12%)
4,79	258,71	0,0332	H-2->LUMO (64%), HOMO->L+5 (15%)
4,73	262,15	0,0039	HOMO->L+4 (36%), HOMO->L+5 (51%)
4,70	264,02	0,0035	HOMO->L+3 (37%), HOMO->L+4 (43%), HOMO->L+5 (10%)
**4,65**	**266,50**	**0,1197**	H-1->L+1 (88%)
4,47	277,18	0,0138	H-1->LUMO (70%), HOMO->L+3 (13%)
3,88	319,17	0,0081	HOMO->L+2 (98%)
3,41	363,47	0,0299	HOMO->L+1 (95%)
**3,32**	**373,35**	**0,0379**	**HOMO->LUMO (95%)**

Table S4 Calculated vertical energy electronic transition, wavelength, oscillator strength, and major orbital contributions of **2p** by the B3LYP/6-311++G(d,p) method

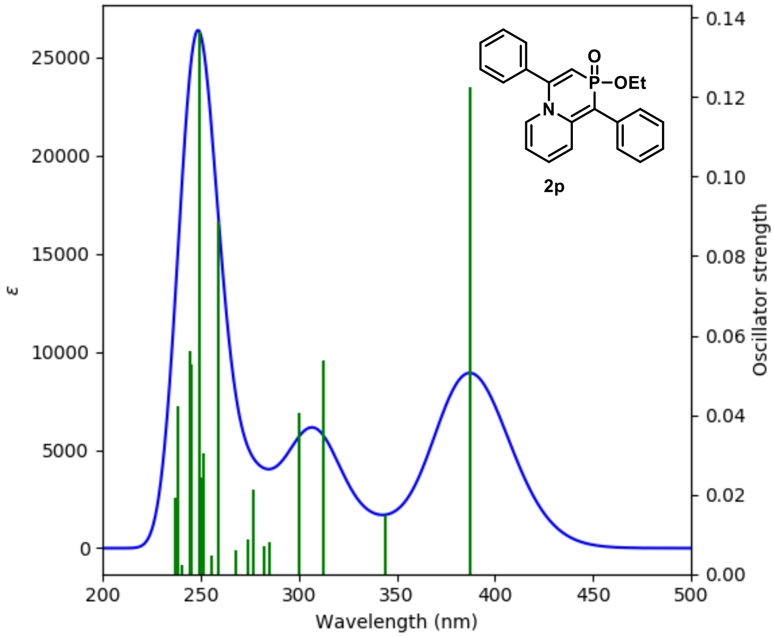

Energy (eV)	Wavelength (nm)	Osc. Strength	Major orbital contributions
5,23	237,12	0,0191	H-7->LUMO (12%), H-6->LUMO (60%), H-5->LUMO (16%)
5,20	238,28	0,0422	H-5->LUMO (54%)
5,15	240,68	0,0023	HOMO->L+10 (79%)
5,08	244,13	0,0560	H-5->LUMO (14%), H-4->LUMO (53%), H-2->L+1 (13%)
5,05	245,40	0,0529	H-5->L+1 (10%), H-2->L+1 (17%), H-2->L+2 (36%)
**4,98**	**249,02**	**0,1365**	H-2->L+1 (13%), H-1->L+1 (64%)
4,97	249,64	0,0244	H-4->LUMO (28%), H-2->L+1 (36%), H-1->L+1 (15%)
4,96	249,99	0,0097	HOMO->L+8 (50%), HOMO->L+9 (36%)
**4,93**	**251,66**	**0,0304**	HOMO->L+8 (29%), HOMO->L+9 (40%)
4,86	255,22	0,0048	HOMO->L+6 (76%), HOMO->L+7 (11%)
4,79	258,65	0,0885	HOMO->L+7 (52%)
4,63	267,83	0,0060	H-3->LUMO (87%)
4,53	273,79	0,0088	HOMO->L+4 (25%), HOMO->L+5 (57%)
4,48	276,51	0,0212	H-2->LUMO (52%), HOMO->L+4 (19%), HOMO->L+7 (11%)
4,39	282,15	0,0070	H-2->LUMO (26%), H-1->LUMO (10%), HOMO->L+4 (36%), HOMO->L+5 (24%)
4,35	285,20	0,0079	H-1->LUMO (81%), HOMO->L+4 (10%)
4,13	300,07	0,0404	HOMO->L+3 (92%)
**3,97**	**312,67**	**0,0539**	HOMO->L+2 (93%)
3,60	344,11	0,0147	HOMO->L+1 (98%)
**3,20**	**387,41**	**0,1226**	**HOMO->LUMO (98%)**

Table S5 Calculated vertical energy electronic transition, wavelength, oscillator strength, and major orbital contributions of **2q** by the B3LYP/6-311++G(d,p) method

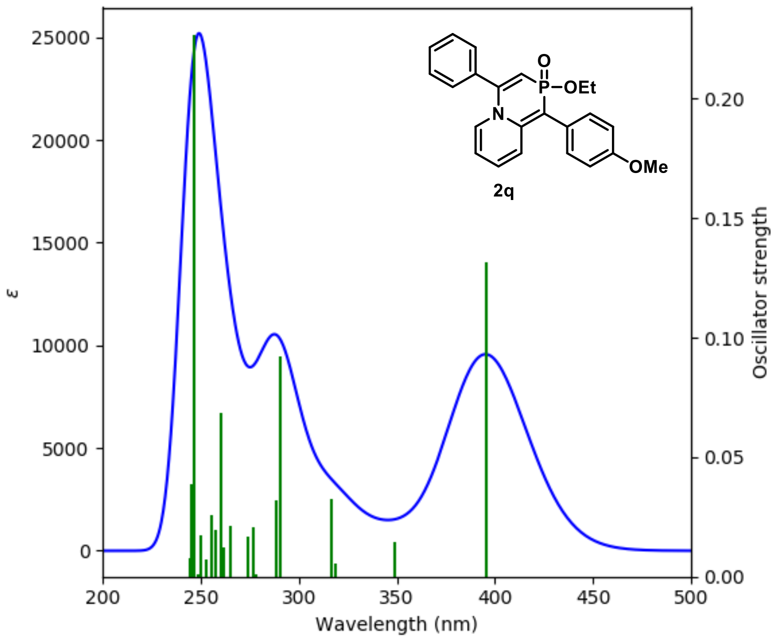

Energy (eV)	Wavelength (nm)	Osc. Strength	Major orbital contributions
5,07	244,64	0,0079	H-1->L+4 (50%)
5,05	245,48	0,0384	H-5->L+1 (14%), H-2->L+2 (41%), H-1->L+4 (12%)
**5,02**	**246,86**	**0,2268**	H-2->L+1 (44%), H-1->L+4 (10%)
4,99	248,37	0,0007	HOMO->L+9 (51%), HOMO->L+10 (29%)
4,96	249,88	0,0172	H-2->L+1 (26%), HOMO->L+9 (12%), HOMO->L+10 (12%), HOMO->L+11 (20%)
4,91	252,63	0,0071	H-4->LUMO (69%)
4,85	255,64	0,0255	H-1->L+2 (30%), HOMO->L+8 (40%)
4,82	257,40	0,0194	H-1->L+2 (50%), HOMO->L+7 (10%), HOMO->L+8 (21%)
4,76	260,45	0,0684	H-3->LUMO (24%), HOMO->L+7 (46%)
4,74	261,37	0,0123	HOMO->L+6 (84%)
**4,68**	**265,13**	**0,0210**	H-3->LUMO (63%), HOMO->L+7 (11%)
4,52	274,18	0,0169	H-1->L+1 (79%)
4,48	276,90	0,0209	H-2->LUMO (42%), H-1->L+1 (16%), HOMO->L+5 (20%), HOMO->L+7 (13%)
4,45	278,42	0,0008	H-2->LUMO (16%), HOMO->L+5 (64%)
4,30	288,34	0,0320	HOMO->L+3 (31%), HOMO->L+4 (59%)
**4,27**	**290,39**	**0,0924**	HOMO->L+3 (61%), HOMO->L+4 (34%)
3,92	316,32	0,0324	HOMO->L+2 (89%)
3,89	318,42	0,0055	H-1->LUMO (90%)
3,55	348,93	0,0144	HOMO->L+1 (98%)
**3,14**	**395,42**	**0,1316**	**HOMO->LUMO (98%)**

Table S6 Calculated vertical energy electronic transition, wavelength, oscillator strength, and major orbital contributions of **2r** by the B3LYP/6-311++G(d,p) method

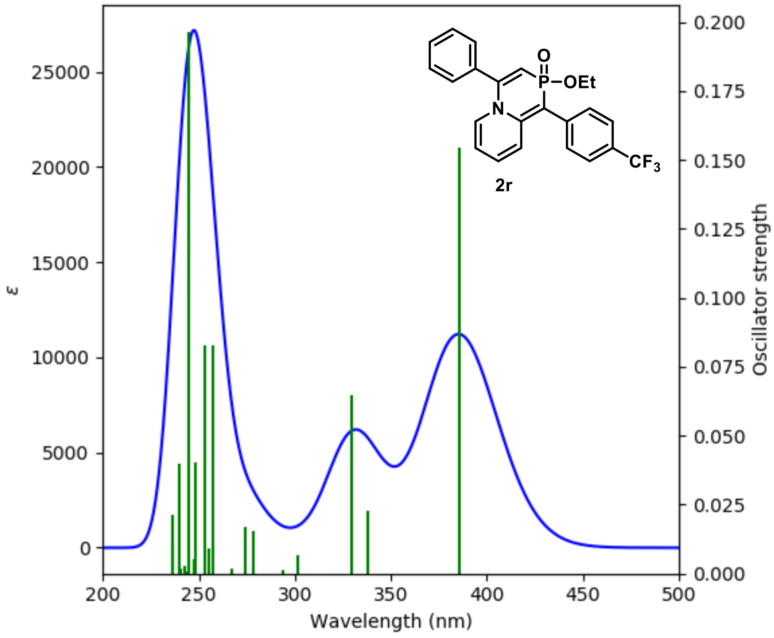

Energy (eV)	Wavelength (nm)	Osc. Strength	Major orbital contributions
5,25	235,93	0,0214	H-7->LUMO (10%), H-2->L+1 (10%), H-2->L+2 (10%), H-1->L+2 (27%), H-1->L+3 (13%)
5,24	236,42	0,0108	H-6->LUMO (29%), H-2->L+1 (44%)
5,17	239,58	0,0397	H-7->LUMO (20%), H-5->LUMO (41%)
5,16	240,48	0,0016	H-6->LUMO (22%), H-5->LUMO (23%), H-4->LUMO (15%), H-2->L+1 (24%)
5,11	242,44	0,0028	HOMO->L+9 (88%)
5,09	243,76	0,0009	H-6->LUMO (10%), H-4->LUMO (36%), H-1->L+2 (28%)
**5,06**	**244,79**	**0,1966**	H-1->L+1 (54%), H-1->L+3 (10%)
5,01	247,67	0,0053	HOMO->L+5 (10%), HOMO->L+7 (85%)
4,99	248,38	0,0403	H-4->LUMO (40%), H-1->L+1 (25%)
4,89	253,32	0,0826	HOMO->L+6 (15%), HOMO->L+8 (65%)
4,86	254,94	0,0093	H-3->LUMO (89%)
4,81	257,59	0,0826	HOMO->L+6 (42%), HOMO->L+8 (28%)
4,64	267,33	0,0019	HOMO->L+5 (87%), HOMO->L+7 (10%)
4,52	274,00	0,0170	H-2->LUMO (96%)
4,45	278,53	0,0157	H-1->LUMO (80%), HOMO->L+6 (15%)
4,22	293,58	0,0012	HOMO->L+4 (95%)
4,11	301,79	0,0068	HOMO->L+3 (92%)
**3,76**	**329,53**	**0,0646**	HOMO->L+1 (18%), HOMO->L+2 (77%)
3,67	337,87	0,0228	HOMO->L+1 (81%), HOMO->L+2 (17%)
**3,22**	**385,44**	**0,1543**	**HOMO->LUMO (98%)**

Table S7 Calculated vertical energy electronic transition, wavelength, oscillator strength, and major orbital contributions of **2t** by the B3LYP/6-311++G(d,p) method

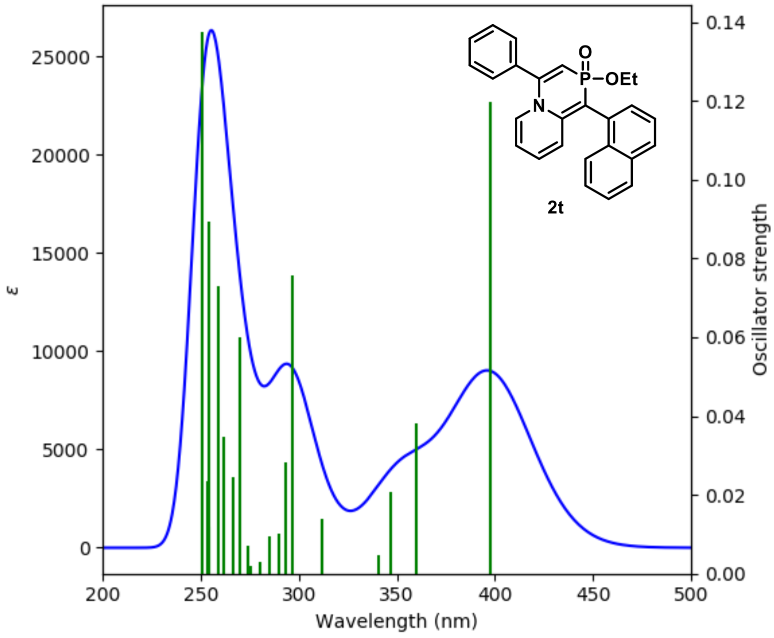

Energy (eV)	Wavelength (nm)	Osc. Strength	Major orbital contributions
**4,94**	**250,95**	**0,1376**	H-3->L+1 (14%), H-3->L+2 (47%), H-2->L+2 (26%)
4,90	253,28	0,0236	HOMO->L+7 (35%), HOMO->L+8 (11%), HOMO->L+9 (34%)
4,88	253,83	0,0895	H-3->L+1 (31%), HOMO->L+9 (28%)
4,88	254,13	0,0120	HOMO->L+7 (52%), HOMO->L+8 (10%), HOMO->L+9 (13%)
4,80	258,55	0,0728	H-4->LUMO (11%), HOMO->L+8 (37%)
4,74	261,67	0,0348	H-4->LUMO (64%), HOMO->L+8 (21%)
4,66	266,21	0,0246	H-4->LUMO (11%), H-1->L+3 (64%), HOMO->L+6 (13%)
4,59	269,82	0,0601	H-1->L+3 (26%), HOMO->L+6 (44%)
4,53	273,77	0,0071	H-2->L+1 (18%), H-1->L+4 (35%), HOMO->L+4 (13%), HOMO->L+5 (14%)
4,50	275,48	0,0019	HOMO->L+5 (74%)
4,42	280,49	0,0029	H-3->LUMO (57%), H-2->LUMO (24%)
4,35	284,85	0,0095	H-3->LUMO (16%), H-2->LUMO (60%)
4,27	290,12	0,0103	H-1->L+2 (89%)
4,23	293,13	0,0283	H-1->L+1 (30%), HOMO->L+4 (46%)
**4,18**	**296,66**	**0,0757**	H-1->L+1 (63%), HOMO->L+4 (22%)
3,98	311,82	0,0139	HOMO->L+3 (96%)
3,64	340,89	0,0047	H-1->LUMO (96%)
3,57	346,99	0,0208	HOMO->L+2 (94%)
3,45	359,64	0,0383	HOMO->L+1 (93%)
**3,12**	**397,92**	**0,1196**	**HOMO->LUMO (98%)**

Table S8 Calculated vertical energy electronic transition, wavelength, oscillator strength, and major orbital contributions of **4a** by the B3LYP/6-311++G(d,p) method

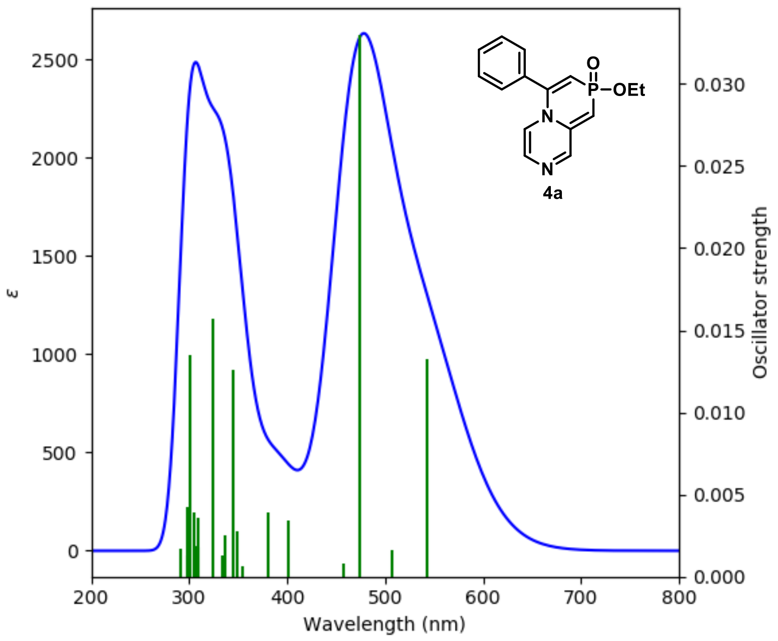

Energy (eV)	Wavelength (nm)	Osc. Strength	Major orbital contributions
4,26	291,30	0,0017	HOMO(A)->L+9(A) (26%), HOMO(A)->L+10(A) (41%), HOMO(A)->L+14(A) (11%)
4,16	297,84	0,0042	H-2(B)->LUMO(B) (74%)
4,16	298,23	0,0013	HOMO(A)->L+6(A) (11%), HOMO(A)->L+9(A) (34%), HOMO(A)->L+10(A) (20%), H-2(B)->LUMO(B) (10%)
**4,12**	**300,58**	**0,0135**	HOMO(B)->L+2(B) (62%)
4,06	305,25	0,0039	H-1(A)->LUMO(A) (15%), HOMO(B)->L+3(B) (66%)
4,04	306,74	0,0019	HOMO(A)->L+7(A) (14%), HOMO(A)->L+8(A) (54%), HOMO(A)->L+9(A) (10%)
4,02	308,58	0,0036	H-1(A)->LUMO(A) (23%), HOMO(A)->L+7(A) (18%), HOMO(B)->L+3(B) (19%)
4,01	308,89	0,0018	H-1(A)->LUMO(A) (11%), HOMO(A)->L+7(A) (39%), HOMO(A)->L+8(A) (13%), HOMO(B)->L+3(B) (10%)
**3,82**	**324,29**	**0,0157**	H-1(B)->LUMO(B) (70%)
3,71	333,95	0,0013	HOMO(A)->L+6(A) (72%)
3,69	336,15	0,0025	H-2(A)->LUMO(A) (11%), H-1(B)->L+1(B) (12%), HOMO(B)->L+1(B) (24%)
**3,60**	**344,37**	**0,0126**	HOMO(A)->L+5(A) (24%), HOMO(B)->L+1(B) (31%)
3,55	349,33	0,0028	HOMO(A)->L+4(A) (71%)
3,50	354,36	0,0006	HOMO(B)->L+2(B) (19%)
3,26	380,28	0,0039	HOMO(A)->L+5(A) (52%), HOMO(B)->L+1(B) (11%)
3,09	401,56	0,0034	HOMO(A)->L+1(A) (33%), HOMO(A)->L+3(A) (63%)
2,71	457,83	0,0008	HOMO(A)->L+2(A) (95%)
**2,61**	**474,56**	**0,0330**	HOMO(A)->L+1(A) (49%), HOMO(A)->L+3(A) (17%), HOMO(B)->LUMO(B) (22%)
**2,44**	**507,67**	**0,0016**	**HOMO(A)->LUMO(A) (86%)**
**2,28**	**542,89**	**0,0132**	HOMO(A)->L+1(A) (13%), HOMO(A)->L+3(A) (12%), HOMO(B)->LUMO(B) (71%)

Table S9 Calculated vertical energy electronic transition, wavelength, oscillator strength, and major orbital contributions of **4c** by the B3LYP/6-311++G(d,p) method

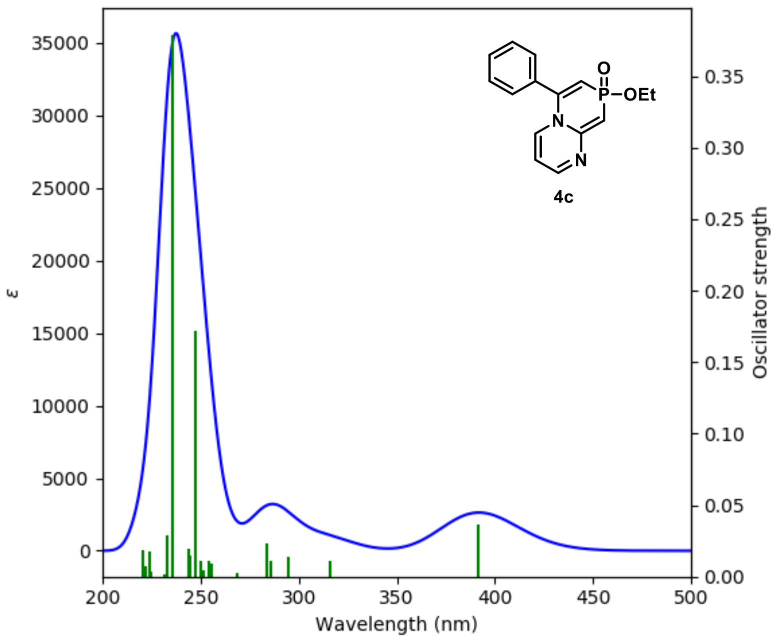

Energy (eV)	Wavelength (nm)	Osc. Strength	Major orbital contributions
5,62	220,42	0,0187	H-4->L+1 (52%), H-3->L+1 (32%)
5,59	221,82	0,0068	HOMO->L+7 (29%), HOMO->L+8 (62%)
5,53	224,00	0,0180	H-7->LUMO (17%), H-3->L+1 (22%), H-1->L+2 (12%), HOMO->L+8 (12%)
5,52	224,72	0,0035	H-7->LUMO (12%), HOMO->L+7 (49%), HOMO->L+8 (15%)
5,36	231,39	0,0015	H-2->L+1 (84%)
5,33	232,61	0,0291	HOMO->L+6 (78%)
**5,27**	**235,42**	**0,3792**	H-7->LUMO (13%), H-5->LUMO (13%), HOMO->L+4 (28%), HOMO->L+5 (15%)
5,08	243,83	0,0192	H-1->L+2 (34%), HOMO->L+5 (17%)
5,07	244,53	0,0144	H-1->L+2 (20%), HOMO->L+5 (46%)
**5,01**	**247,34**	**0,1720**	H-5->LUMO (27%), H-1->L+1 (58%)
4,96	250,03	0,0109	H-6->LUMO (26%), H-4->LUMO (51%)
4,93	251,29	0,0047	HOMO->L+3 (63%), HOMO->L+4 (23%)
4,88	254,23	0,0107	H-5->LUMO (24%), H-4->LUMO (20%), H-1->L+1 (17%), HOMO->L+3 (14%), HOMO->L+4 (14%)
4,85	255,50	0,0090	H-3->LUMO (85%)
4,62	268,53	0,0027	H-2->LUMO (88%)
**4,37**	**283,48**	**0,0232**	H-6->LUMO (27%), H-4->LUMO (11%), HOMO->L+2 (43%)
4,34	285,62	0,0107	H-6->LUMO (28%), H-5->LUMO (11%), HOMO->L+2 (43%)
4,21	294,56	0,0139	H-1->LUMO (81%), HOMO->L+2 (12%)
3,93	315,79	0,0113	HOMO->L+1 (95%)
**3,16**	**391,87**	**0,0363**	**HOMO->LUMO (98%)**

Table S10 Calculated vertical energy electronic transition, wavelength, oscillator strength, and major orbital contributions of **4d*meta*** by the B3LYP/6-311++G(d,p) method

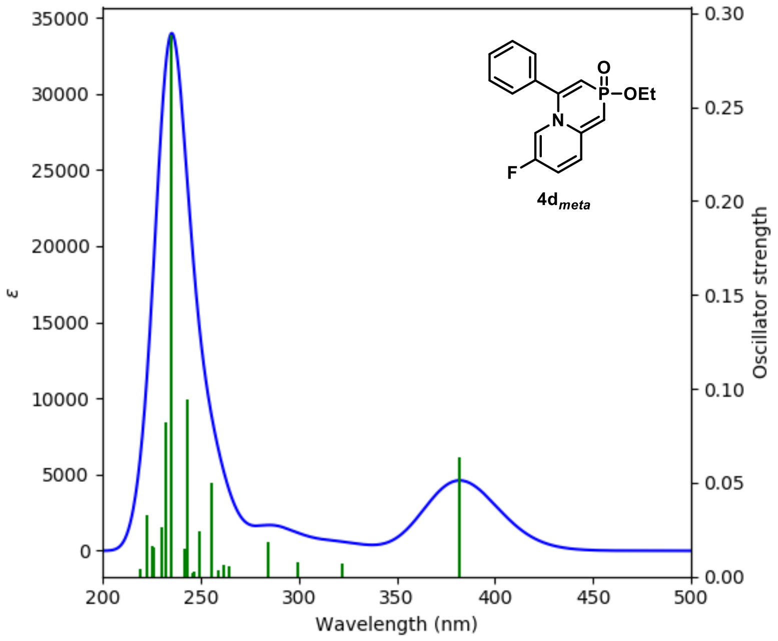

Energy (eV)	Wavelength (nm)	Osc. Strength	Major orbital contributions
5,66	219,18	0,0044	HOMO->L+10 (53%), HOMO->L+11 (31%)
5,58	222,22	0,0328	H-5->L+2 (14%), H-3->L+1 (23%), H-1->L+2 (22%)
5,50	225,24	0,0164	HOMO->L+8 (19%), HOMO->L+9 (58%)
5,49	225,85	0,0159	H-2->L+1 (66%), H-1->L+2 (10%)
5,39	229,82	0,0260	HOMO->L+8 (62%), HOMO->L+9 (14%)
5,34	232,22	0,0824	HOMO->L+7 (71%)
**5,28**	**235,01**	**0,2887**	H-4->LUMO (21%), H-1->L+1 (13%), HOMO->L+5 (17%), HOMO->L+7 (15%)
5,13	241,53	0,0145	H-3->L+1 (16%), H-2->L+1 (11%), H-1->L+2 (50%)
5,10	243,20	0,0947	H-4->LUMO (20%), H-1->L+1 (62%)
5,05	245,51	0,0022	H-5->LUMO (78%)
5,02	246,83	0,0025	HOMO->L+3 (16%), HOMO->L+6 (67%)
4,98	249,18	0,0245	H-3->LUMO (63%)
4,85	255,57	0,0502	H-4->LUMO (28%), H-3->LUMO (19%), H-2->LUMO (13%), HOMO->L+4 (15%), HOMO->L+5 (14%)
4,79	258,97	0,0034	HOMO->L+3 (31%), HOMO->L+4 (36%), HOMO->L+6 (21%)
4,74	261,71	0,0061	H-2->LUMO (26%), HOMO->L+3 (22%), HOMO->L+4 (10%), HOMO->L+5 (33%)
4,69	264,60	0,0056	H-2->LUMO (53%), HOMO->L+3 (22%), HOMO->L+4 (18%)
4,36	284,32	0,0186	H-1->LUMO (83%)
4,14	299,29	0,0080	HOMO->L+2 (93%)
3,85	321,89	0,0067	HOMO->L+1 (99%)
**3,25**	**382,04**	**0,0637**	**HOMO->LUMO (98%)**

Table S11 Calculated vertical energy electronic transition, wavelength, oscillator strength, and major orbital contributions of **4d***para* by the B3LYP/6-311++G(d,p) method

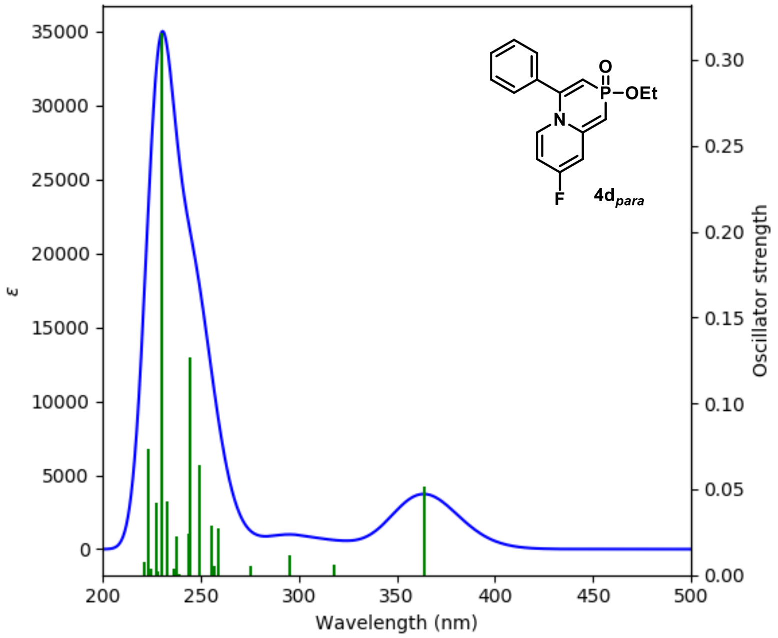

Energy (eV)	Wavelength (nm)	Osc. Strength	Major orbital contributions
5,61	220,98	0,0079	HOMO->L+8 (12%), HOMO->L+10 (48%), HOMO->L+11 (18%), HOMO->L+12 (13%)
5,55	223,19	0,0736	H-3->L+1 (17%), H-2->L+1 (12%), H-1->L+2 (12%)
5,53	224,27	0,0039	HOMO->L+6 (10%), HOMO->L+7 (11%), HOMO->L+9 (71%)
5,45	227,35	0,0420	H-2->L+1 (70%), H-1->L+2 (13%)
5,45	227,67	0,0019	HOMO->L+8 (77%)
**5,39**	**230,18**	**0,3159**	H-4->LUMO (31%), HOMO->L+7 (10%)
5,32	233,04	0,0427	HOMO->L+7 (67%)
5,25	236,15	0,0038	H-5->LUMO (95%)
5,22	237,57	0,0225	H-4->LUMO (23%), H-3->LUMO (54%)
5,19	238,71	0,0006	HOMO->L+4 (10%), HOMO->L+6 (67%), HOMO->L+9 (16%)
5,09	243,53	0,0243	H-3->L+1 (11%), H-1->L+2 (50%)
5,07	244,30	0,1264	H-4->LUMO (12%), H-3->LUMO (29%), H-1->L+1 (36%)
4,97	249,34	0,0642	H-2->LUMO (20%), H-1->L+1 (41%), HOMO->L+3 (12%)
4,85	255,39	0,0288	H-2->LUMO (48%), HOMO->L+5 (37%)
4,82	257,03	0,0051	HOMO->L+4 (85%), HOMO->L+6 (11%)
4,78	259,15	0,0271	H-2->LUMO (14%), HOMO->L+3 (44%), HOMO->L+5 (36%)
4,50	275,56	0,0056	H-1->LUMO (77%), HOMO->L+3 (14%)
4,20	295,14	0,0118	HOMO->L+2 (96%)
3,90	318,10	0,0062	HOMO->L+1 (99%)
**3,41**	**363,77**	**0,0514**	**HOMO->LUMO (98%)**

Table S12 Calculated vertical energy electronic transition, wavelength, oscillator strength, and major orbital contributions of **4f*meta*** by the B3LYP/6-311++G(d,p) method

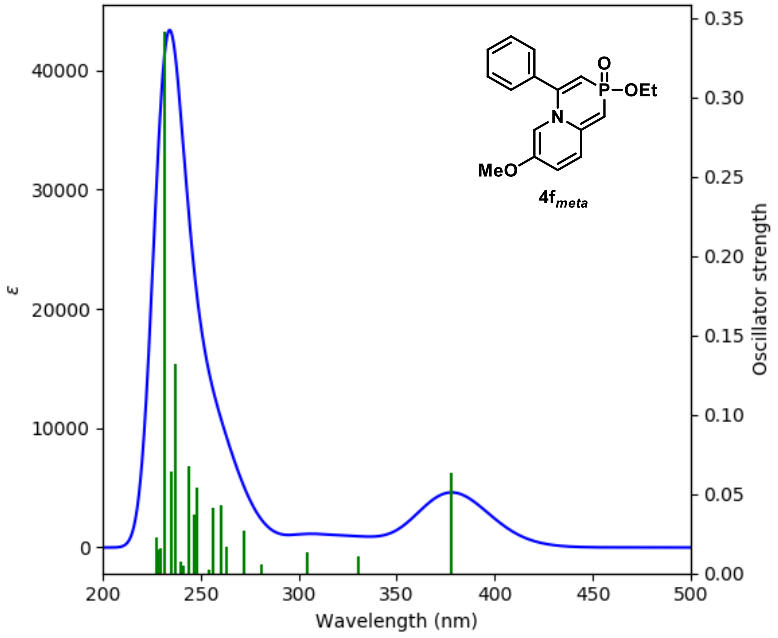

Energy (eV)	Wavelength (nm)	Osc. Strength	Major orbital contributions
5,45	227,34	0,0228	H-2->L+1 (76%)
5,44	228,00	0,0151	H-7->LUMO (13%), H-6->LUMO (70%)
5,40	229,62	0,0160	HOMO->L+9 (11%), HOMO->L+10 (67%), HOMO->L+11 (12%)
**5,35**	**231,79**	**0,3415**	H-3->LUMO (10%), HOMO->L+9 (30%)
5,28	235,00	0,0640	HOMO->L+8 (38%), HOMO->L+9 (34%)
5,23	237,10	0,1323	HOMO->L+8 (48%), HOMO->L+9 (12%)
5,17	240,00	0,0078	H-5->LUMO (82%)
5,14	241,44	0,0051	H-4->LUMO (51%), H-3->LUMO (15%), H-1->L+2 (16%)
5,08	243,93	0,0673	H-1->L+1 (26%), H-1->L+2 (34%)
5,03	246,70	0,0370	H-1->L+1 (25%), HOMO->L+7 (54%)
5,00	248,04	0,0538	H-3->LUMO (29%), H-1->L+1 (23%), HOMO->L+7 (29%)
4,87	254,44	0,0027	HOMO->L+4 (53%), HOMO->L+5 (31%)
4,84	256,20	0,0414	H-3->LUMO (16%), H-2->LUMO (56%), HOMO->L+4 (10%)
4,76	260,37	0,0432	H-3->LUMO (11%), H-2->LUMO (29%), HOMO->L+6 (46%)
4,71	263,31	0,0170	HOMO->L+4 (16%), HOMO->L+5 (49%), HOMO->L+6 (22%)
4,56	272,04	0,0265	HOMO->L+3 (83%)
4,41	281,09	0,0056	H-1->LUMO (77%)
4,07	304,57	0,0136	HOMO->L+2 (96%)
3,75	330,28	0,0105	HOMO->L+1 (99%)
**3,28**	**378,13**	**0,0636**	**HOMO->LUMO (98%)**

Table S13 Calculated vertical energy electronic transition, wavelength, oscillator strength, and major orbital contributions of **4f**$_{para}$ by the B3LYP/6-311++G(d,p) method

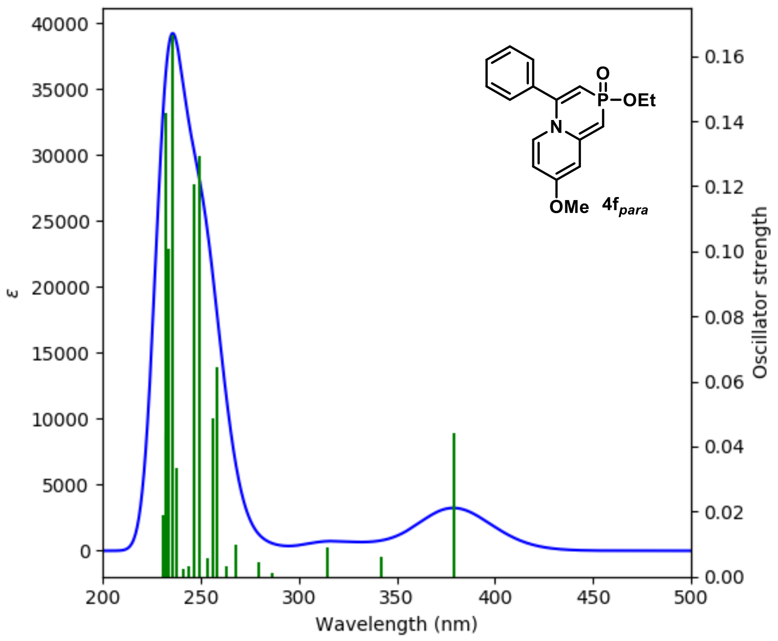

Energy (eV)	Wavelength (nm)	Osc. Strength	Major orbital contributions
5,37	230,99	0,0187	H-5->LUMO (66%), H-4->LUMO (16%)
5,34	232,00	0,1425	H-2->L+1 (14%), HOMO->L+11 (32%), HOMO->L+13 (11%)
5,34	232,24	0,0441	H-5->LUMO (19%), H-4->LUMO (23%), H-2->L+1 (47%)
5,31	233,61	0,1008	H-4->LUMO (13%), H-2->L+1 (12%), HOMO->L+11 (23%)
**5,26**	**235,71**	**0,1666**	H-4->LUMO (19%), H-3->LUMO (37%)
5,22	237,70	0,0332	HOMO->L+10 (68%)
5,15	240,71	0,0023	HOMO->L+7 (15%), HOMO->L+9 (79%)
5,09	243,56	0,0032	HOMO->L+8 (86%)
5,02	246,78	0,1204	H-3->LUMO (32%), H-2->LUMO (10%), H-1->L+2 (30%)
4,97	249,53	0,1292	H-1->L+2 (43%)
4,90	253,26	0,0055	HOMO->L+7 (68%), HOMO->L+9 (11%), HOMO->L+10 (10%)
4,83	256,47	0,0487	H-2->LUMO (42%), H-1->L+1 (52%)
4,80	258,16	0,0643	H-2->LUMO (32%), H-1->L+1 (36%), HOMO->L+5 (10%), HOMO->L+6 (11%)
4,72	262,86	0,0032	HOMO->L+4 (96%)
4,63	267,56	0,0099	HOMO->L+5 (44%), HOMO->L+6 (52%)
4,43	279,80	0,0044	H-1->LUMO (72%), HOMO->L+5 (14%), HOMO->L+6 (12%)
4,33	286,18	0,0010	HOMO->L+3 (96%)
3,94	314,30	0,0091	HOMO->L+2 (98%)
3,62	342,15	0,0061	HOMO->L+1 (99%)
**3,27**	**379,30**	**0,0443**	**HOMO->LUMO (98%)**

Table S14 Calculated vertical energy electronic transition, wavelength, oscillator strength, and major orbital contributions of **4g**_**meta**_ by the B3LYP/6-311++G(d,p) method

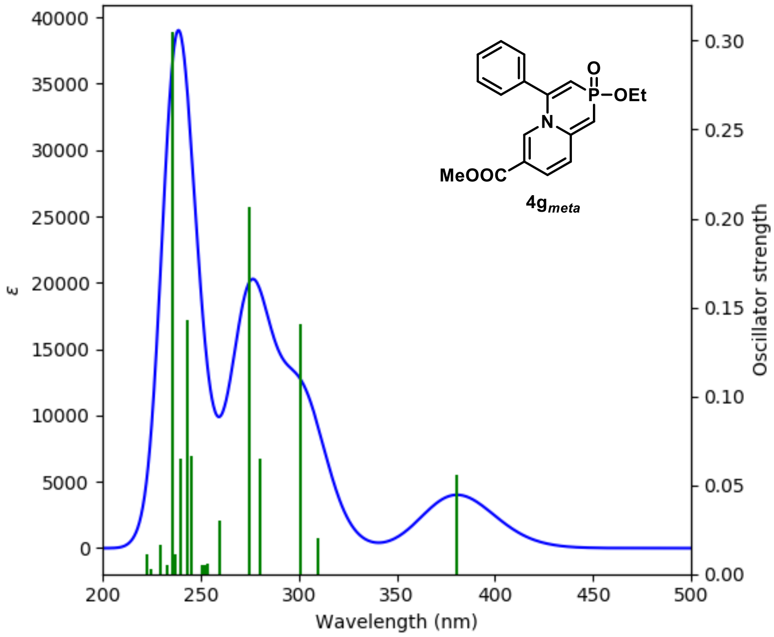

Energy (eV)	Wavelength (nm)	Osc. Strength	Major orbital contributions
5,57	222,50	0,0110	H-3->L+2 (32%), H-2->L+2 (51%)
5,52	224,44	0,0028	HOMO->L+8 (87%)
5,41	229,36	0,0165	H-3->L+1 (14%), H-2->L+1 (72%)
5,33	232,54	0,0048	HOMO->L+6 (14%), HOMO->L+7 (74%)
**5,26**	**235,73**	**0,3051**	H-5->LUMO (35%), H-1->L+2 (24%)
5,24	236,78	0,0114	HOMO->L+6 (71%)
5,18	239,39	0,0649	H-3->L+2 (15%), H-2->L+2 (20%), H-1->L+2 (11%), H-1->L+3 (34%)
5,10	242,89	0,1431	H-5->LUMO (29%), H-1->L+2 (51%)
5,05	245,40	0,0661	H-4->LUMO (84%)
4,95	250,64	0,0047	H-1->L+1 (62%)
4,94	250,84	0,0049	H-5->LUMO (12%), HOMO->L+4 (14%), HOMO->L+5 (57%)
4,92	251,78	0,0050	H-6->LUMO (19%), H-3->LUMO (64%)
4,90	253,07	0,0058	H-6->LUMO (58%), H-6->L+1 (10%), H-3->LUMO (25%)
4,89	253,74	0,0014	HOMO->L+4 (62%), HOMO->L+5 (20%)
4,78	259,56	0,0301	H-2->LUMO (86%)
**4,51**	**274,78**	**0,2067**	H-1->LUMO (25%), HOMO->L+3 (55%)
4,42	280,52	0,0647	H-1->LUMO (44%), HOMO->L+3 (42%)
**4,13**	**300,52**	**0,1405**	H-1->LUMO (22%), HOMO->L+1 (70%)
4,00	310,08	0,0199	HOMO->L+2 (95%)
**3,26**	**380,70**	**0,0555**	**HOMO->LUMO (99%)**

Table S15 Calculated vertical energy electronic transition, wavelength, oscillator strength, and major orbital contributions of **4g**$_{para}$ by the B3LYP/6-311++G(d,p) method

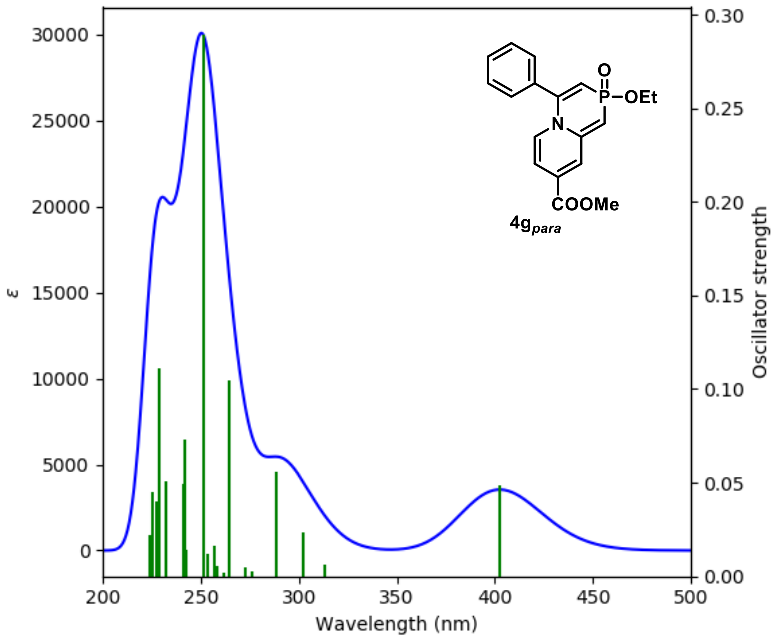

Energy (eV)	Wavelength (nm)	Osc. Strength	Major orbital contributions
5,54	223,62	0,02	H-3->L+1 (33%), H-2->L+1 (17%)
5,51	225,16	0,04	H-8->LUMO (82%)
5,46	227,01	0,04	H-2->L+1 (49%), H-1->L+2 (21%), H-1->L+3 (12%)
**5,43**	**228,37**	**0,11**	H-7->LUMO (41%), HOMO->L+7 (30%)
5,35	231,83	0,05	H-7->LUMO (30%), HOMO->L+7 (47%)
5,15	240,81	0,05	H-1->L+1 (26%), HOMO->L+6 (50%)
5,13	241,66	0,07	H-1->L+1 (49%), HOMO->L+6 (30%)
5,11	242,63	0,01	H-3->L+1 (18%), H-2->L+1 (14%), H-1->L+2 (53%)
**4,94**	**251,05**	**0,29**	H-6->LUMO (20%), H-4->LUMO (36%), H-1->L+1 (10%), HOMO->L+3 (19%)
4,89	253,63	0,01	HOMO->L+5 (77%), HOMO->L+6 (12%)
4,83	256,86	0,02	H-5->LUMO (33%), HOMO->L+4 (52%)
4,80	258,16	0,01	H-5->LUMO (44%), H-3->LUMO (20%), HOMO->L+4 (20%)
4,74	261,82	0,00	H-3->LUMO (64%), HOMO->L+4 (12%)
4,69	264,52	0,10	H-6->LUMO (22%), H-2->LUMO (10%), HOMO->L+3 (41%)
4,55	272,70	0,01	H-6->LUMO (18%), H-4->LUMO (26%), H-2->LUMO (21%), HOMO->L+3 (19%)
4,50	275,80	0,00	H-6->LUMO (16%), H-2->LUMO (57%)
**4,29**	**288,72**	**0,06**	H-1->LUMO (12%), HOMO->L+2 (78%)
4,10	302,28	0,02	H-1->LUMO (68%), HOMO->L+1 (12%), HOMO->L+2 (15%)
3,96	313,00	0,01	H-1->LUMO (12%), HOMO->L+1 (86%)
**3,08**	**402,52**	**0,05**	**HOMO->LUMO (98%)**

Table S16 Calculated vertical energy electronic transition, wavelength, oscillator strength, and major orbital contributions of **4h** by the B3LYP/6-311++G(d,p) method

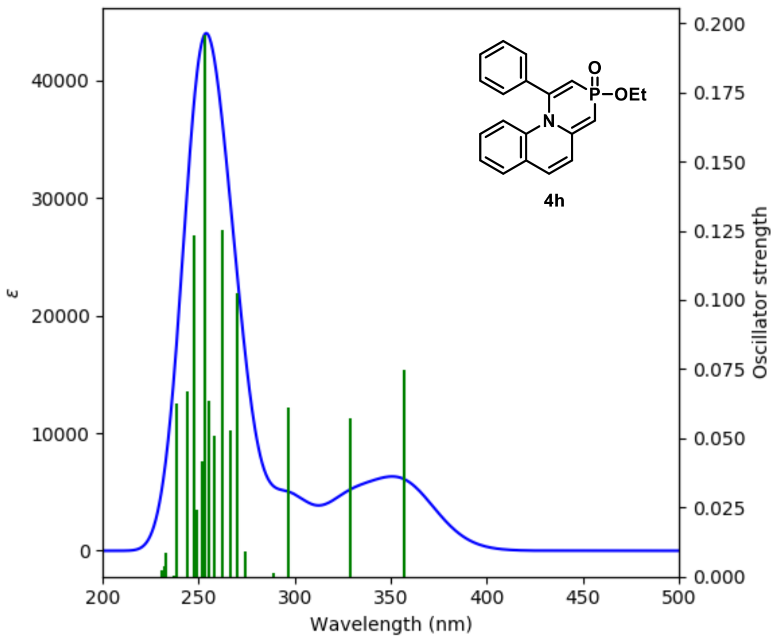

Energy (eV)	Wavelength (nm)	Osc. Strength	Major orbital contributions
5,37	231,07	0,0022	H-5->L+1 (49%)
5,34	232,14	0,0037	HOMO->L+7 (67%)
5,32	233,19	0,0085	H-2->L+2 (19%), H-1->L+3 (56%)
5,23	237,11	0,0002	HOMO->L+6 (75%)
5,19	238,71	0,0627	H-1->L+2 (57%), H-1->L+3 (14%)
5,08	244,20	0,0668	H-4->L+1 (29%), H-3->L+1 (36%)
5,01	247,45	0,1231	H-3->L+1 (12%), HOMO->L+5 (64%)
4,98	248,93	0,0240	H-5->LUMO (20%), H-4->LUMO (13%), H-3->L+1 (32%)
4,93	251,49	0,0419	H-5->LUMO (38%), H-4->LUMO (20%), H-1->L+2 (10%)
**4,90**	**253,18**	**0,1959**	H-5->LUMO (27%), H-2->L+1 (19%), HOMO->L+4 (23%)
4,86	255,36	0,0635	H-4->LUMO (52%), H-4->L+1 (11%)
4,81	257,78	0,0511	H-2->L+1 (50%), HOMO->L+4 (40%)
4,73	262,30	0,1253	H-3->LUMO (49%), HOMO->L+2 (10%)
4,66	266,32	0,0526	H-3->LUMO (23%), H-1->L+1 (51%)
4,59	270,21	0,1026	H-3->LUMO (20%), H-2->LUMO (28%), H-1->L+1 (18%), HOMO->L+2 (19%)
4,53	273,97	0,0092	HOMO->L+2 (23%), HOMO->L+3 (74%)
4,30	288,62	0,0011	H-2->LUMO (56%), HOMO->L+2 (23%)
**4,18**	**296,88**	**0,0611**	H-1->LUMO (70%), HOMO->L+2 (17%)
**3,77**	**328,55**	**0,0571**	HOMO->L+1 (90%)
**3,47**	**357,09**	**0,0746**	**HOMO->LUMO (94%)**

Table S17 Calculated vertical energy electronic transition, wavelength, oscillator strength, and major orbital contributions of **4i** by the B3LYP/6-311++G(d,p) method

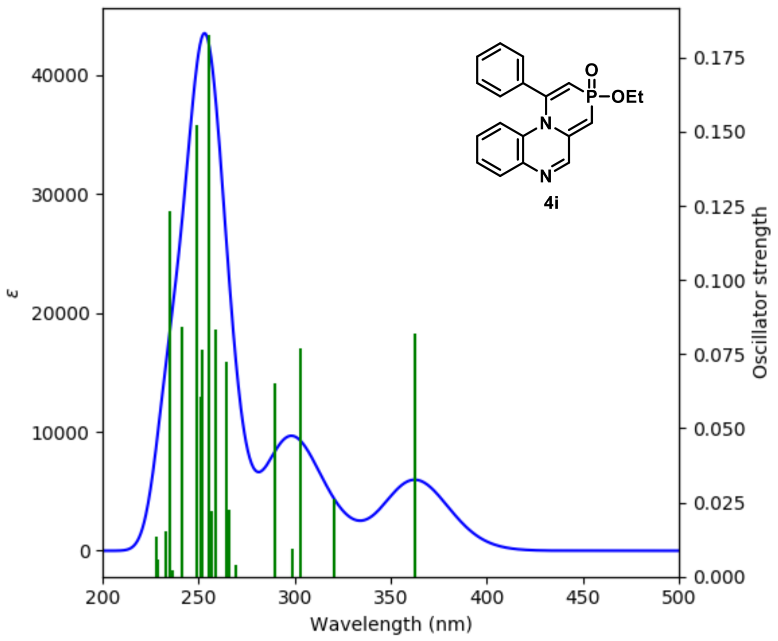

Energy (eV)	Wavelength (nm)	Osc. Strength	Major orbital contributions
5,44	227,90	0,0136	H-5->L+1 (25%), H-4->L+1 (11%), H-1->L+2 (22%), HOMO->L+6 (18%)
5,43	228,47	0,0059	H-5->L+1 (33%), H-2->L+2 (10%), H-1->L+3 (47%)
5,32	232,92	0,0154	H-8->LUMO (27%), H-7->LUMO (16%), H-1->L+3 (11%)
5,28	234,89	0,1232	H-7->LUMO (43%)
5,25	236,23	0,0021	H-7->LUMO (10%), HOMO->L+5 (71%)
5,14	241,40	0,0841	H-4->L+1 (17%), H-3->L+1 (68%)
4,99	248,66	0,1521	H-6->LUMO (19%), H-5->LUMO (11%), H-3->L+1 (12%), HOMO->L+4 (20%)
4,94	251,21	0,0606	H-4->L+1 (32%), H-1->L+2 (25%)
4,93	251,50	0,0767	H-6->LUMO (45%), HOMO->L+4 (21%)
**4,85**	**255,47**	**0,1828**	H-1->L+1 (30%), HOMO->L+3 (18%), HOMO->L+4 (25%)
4,83	256,55	0,0219	H-5->LUMO (22%), H-2->L+1 (42%)
4,79	258,69	0,0832	H-5->LUMO (31%), H-4->LUMO (12%), H-2->L+1 (31%), H-1->L+1 (17%)
4,69	264,29	0,0724	H-4->LUMO (23%), H-1->L+1 (19%), HOMO->L+3 (27%)
4,67	265,49	0,0225	H-4->LUMO (23%), H-3->LUMO (36%), H-1->L+1 (12%)
4,60	269,45	0,0040	HOMO->L+2 (83%), HOMO->L+3 (13%)
4,28	289,59	0,0651	H-4->LUMO (10%), H-3->LUMO (24%), H-2->LUMO (28%), HOMO->L+3 (13%)
4,15	298,40	0,0096	H-3->LUMO (15%), H-2->LUMO (52%), H-1->LUMO (15%)
**4,09**	**303,09**	**0,0769**	H-3->LUMO (13%), H-1->LUMO (53%), HOMO->L+1 (17%)
3,87	320,15	0,0262	H-1->LUMO (20%), HOMO->L+1 (75%)
**3,42**	**362,68**	**0,0820**	**HOMO->LUMO (96%)**

Table S18 Calculated vertical energy electronic transition, wavelength, oscillator strength, and major orbital contributions of **4j** by the B3LYP/6-311++G(d,p) method

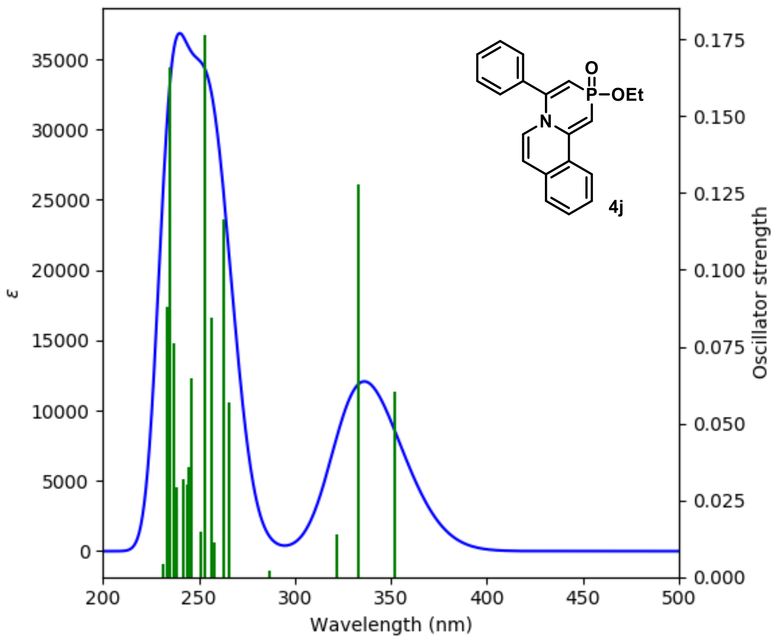

Energy (eV)	Wavelength (nm)	Osc. Strength	Major orbital contributions
5,36	231,44	0,0041	H-5->LUMO (10%), HOMO->L+7 (14%), HOMO->L+8 (61%)
5,30	233,72	0,0878	H-2->L+2 (39%), HOMO->L+9 (20%)
**5,27**	**235,07**	**0,1661**	H-4->LUMO (11%), H-2->L+2 (20%), HOMO->L+9 (27%)
5,24	236,69	0,0759	H-4->LUMO (45%), H-3->L+1 (15%)
5,20	238,42	0,0294	HOMO->L+7 (62%), HOMO->L+8 (12%)
5,19	238,69	0,0233	H-4->LUMO (17%), H-3->L+1 (64%)
5,12	242,12	0,0317	H-5->LUMO (22%), H-2->L+1 (17%), H-2->L+2 (22%)
5,08	244,11	0,0303	H-2->L+1 (31%), H-1->L+2 (21%), HOMO->L+5 (17%)
5,07	244,35	0,0358	H-2->L+1 (13%), HOMO->L+5 (53%), HOMO->L+6 (13%)
5,04	245,97	0,0648	HOMO->L+5 (15%), HOMO->L+6 (52%)
4,95	250,69	0,0147	H-3->LUMO (32%), H-1->L+2 (20%)
**4,90**	**252,96**	**0,1766**	H-3->LUMO (13%), H-1->L+1 (31%), H-1->L+2 (37%)
4,84	256,36	0,0846	H-3->LUMO (35%), H-2->LUMO (29%), H-1->L+1 (19%)
4,81	257,88	0,0112	HOMO->L+4 (88%)
4,71	263,19	0,1165	H-2->LUMO (10%), H-1->LUMO (63%)
4,66	265,99	0,0568	H-2->LUMO (42%), H-1->LUMO (10%), H-1->L+1 (28%)
4,32	286,67	0,0019	HOMO->L+3 (97%)
3,86	321,55	0,0137	HOMO->L+2 (98%)
**3,72**	**332,93**	**0,1280**	HOMO->L+1 (90%)
**3,52**	**352,25**	**0,0602**	**HOMO->LUMO (97%)**

Table S19 Calculated vertical energy electronic transition, wavelength, oscillator strength, and major orbital contributions of **4l** by the B3LYP/6-311++G(d,p) method

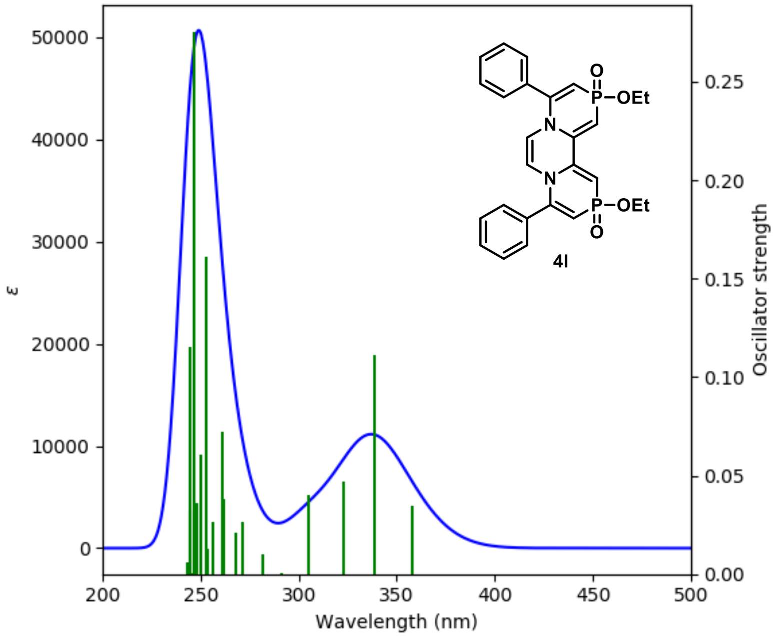

Energy (eV)	Wavelength (nm)	Osc. Strength	Major orbital contributions
5,10	243,01	0,0059	H-8->LUMO (11%), HOMO->L+9 (14%), HOMO->L+10 (16%), HOMO->L+11 (14%)
5,08	244,23	0,0316	HOMO->L+7 (79%)
5,07	244,50	0,1152	H-4->LUMO (14%), H-2->LUMO (12%), HOMO->L+7 (13%)
5,05	245,67	0,0008	H-1->L+3 (50%)
**5,02**	**246,73**	**0,2754**	H-4->LUMO (16%), H-2->LUMO (13%)
5,01	247,67	0,0357	H-5->LUMO (35%), H-2->L+1 (28%)
4,96	250,04	0,0603	H-5->LUMO (25%), H-3->LUMO (10%), H-2->L+1 (41%)
4,90	252,80	0,1614	H-4->LUMO (32%), H-3->LUMO (11%), H-2->LUMO (10%), H-1->L+2 (26%)
4,90	253,18	0,0129	H-4->LUMO (16%), H-3->LUMO (57%)
4,84	256,40	0,0263	HOMO->L+8 (80%)
4,75	261,27	0,0720	H-2->LUMO (26%), H-1->L+2 (47%), HOMO->L+5 (12%)
4,74	261,68	0,0381	HOMO->L+6 (81%)
4,63	267,95	0,0211	H-1->L+1 (93%)
4,57	271,43	0,0266	H-2->LUMO (23%), HOMO->L+5 (67%)
4,40	281,71	0,0100	H-1->LUMO (93%)
4,25	291,47	0,0004	HOMO->L+4 (99%)
4,07	304,73	0,0400	HOMO->L+3 (97%)
3,84	322,93	0,0468	HOMO->L+2 (98%)
**3,66**	**338,89**	**0,1115**	HOMO->L+1 (98%)
**3,46**	**358,17**	**0,0347**	**HOMO->LUMO (99%)**

Table S20 Calculated vertical energy electronic transition, wavelength, oscillator strength, and major orbital contributions of **II** by the B3LYP/6-311++G(d,p) method

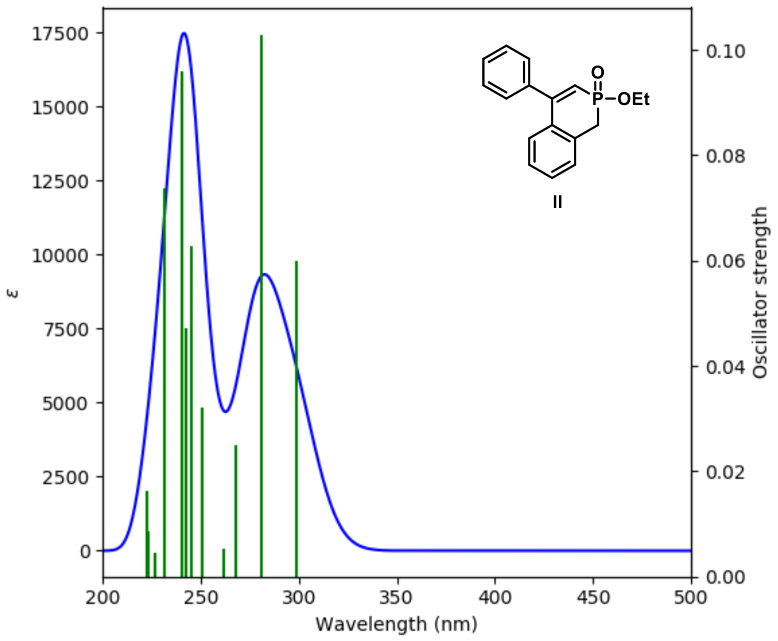

Energy (eV)	Wavelength (nm)	Osc. Strength	Major orbital contributions
5,57	222,46	0,0162	H-1->L+1 (41%), H-1->L+2 (24%)
5,56	222,87	0,0086	H-6->LUMO (94%)
5,48	226,43	0,0045	H-2->L+1 (17%), H-1->L+1 (21%), H-1->L+2 (16%), HOMO->L+3 (24%)
5,36	231,34	0,0736	H-3->L+2 (12%), H-1->L+1 (14%), HOMO->L+3 (43%)
**5,16**	**240,39**	**0,0959**	H-5->LUMO (67%), HOMO->L+2 (18%)
5,11	242,53	0,0472	H-5->LUMO (13%), H-4->LUMO (14%), HOMO->L+2 (46%)
5,06	245,00	0,0627	H-5->LUMO (16%), H-3->LUMO (20%), HOMO->L+1 (50%)
4,94	250,86	0,0322	H-4->LUMO (72%), HOMO->L+2 (15%)
4,73	261,91	0,0052	H-3->LUMO (57%), H-2->LUMO (11%), HOMO->L+1 (21%)
4,63	267,60	0,0249	H-2->LUMO (80%)
**4,41**	**280,89**	**0,1029**	H-1->LUMO (71%), HOMO->LUMO (17%)
**4,15**	**298,76**	**0,0600**	H-1->LUMO (19%), **HOMO->LUMO (77%)**

4. UV-Vis spectra of Selected Compound and Excitation/Emission spectra of **4l**

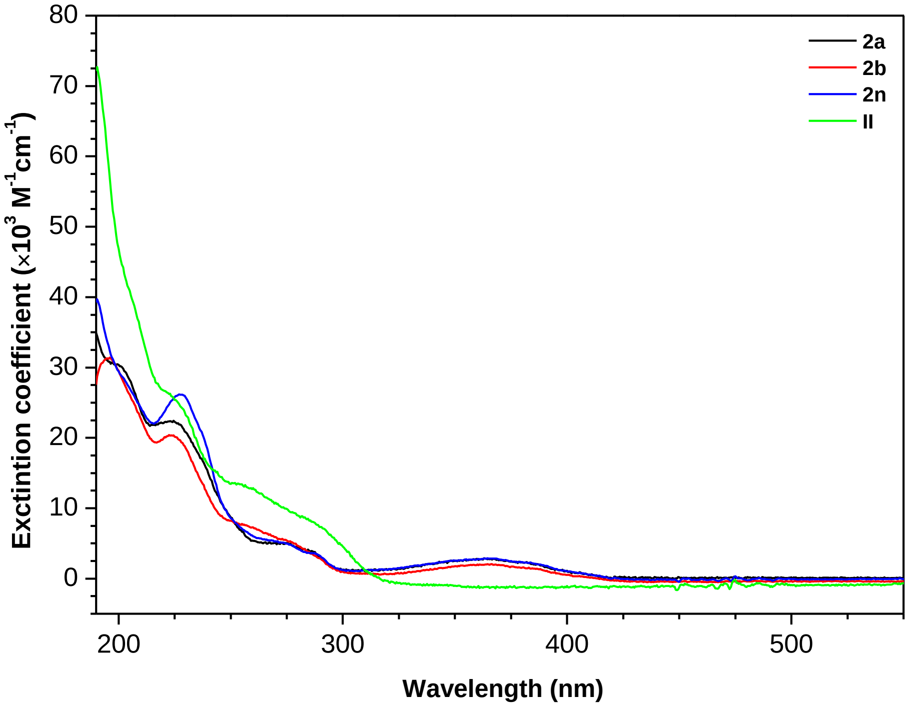

Figure S277 *UV-vis of* **2a**,**2b**,**2n**, *and* **II** *(for simplicity, the* **II** *absorption spectrum of the second stereoisomer is omitted).*

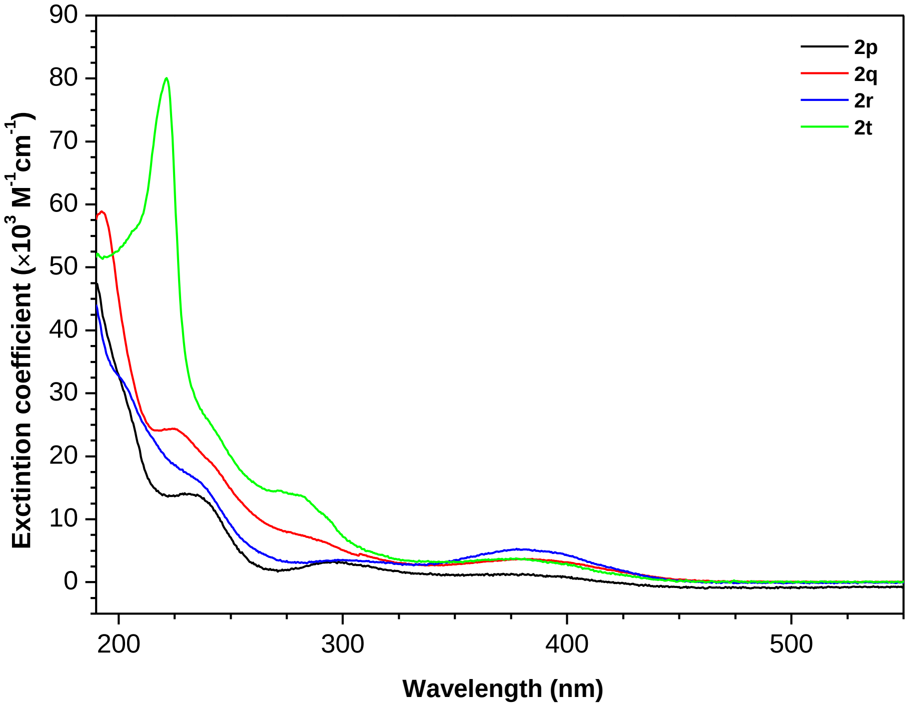

Figure S278 *UV-vis of* **2p**,**2q**,**2r**, *and* **2t** *(for simplicity, the* **2t** *absorption spectrum of the second stereoisomer is omitted).*

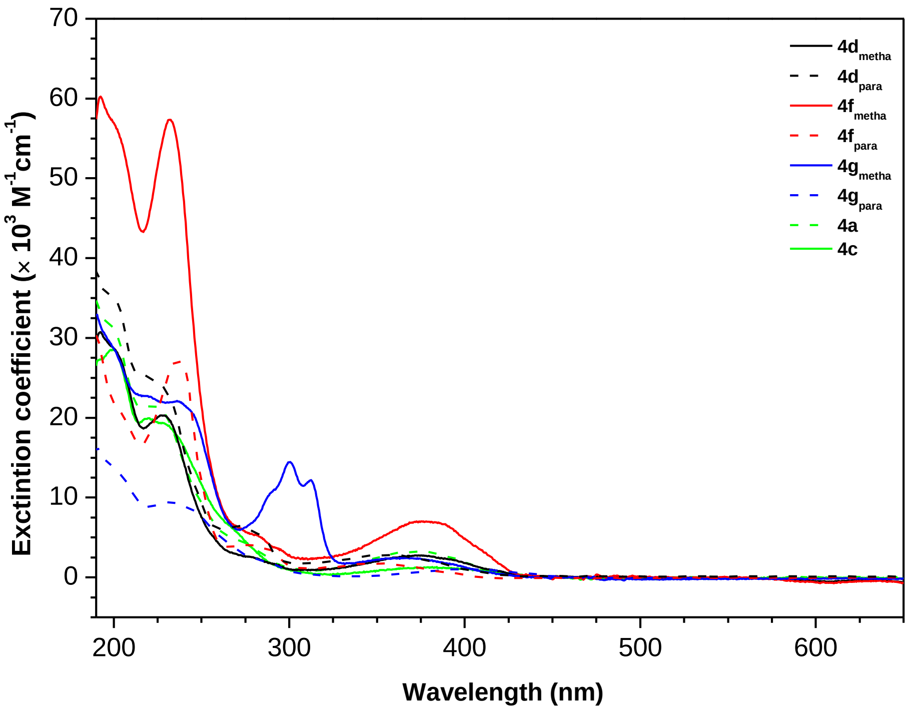

Figure S279 *UV-vis of* **4a**,**4c**, *and positional isomers of* **4d**,**4f**, and **4g**.

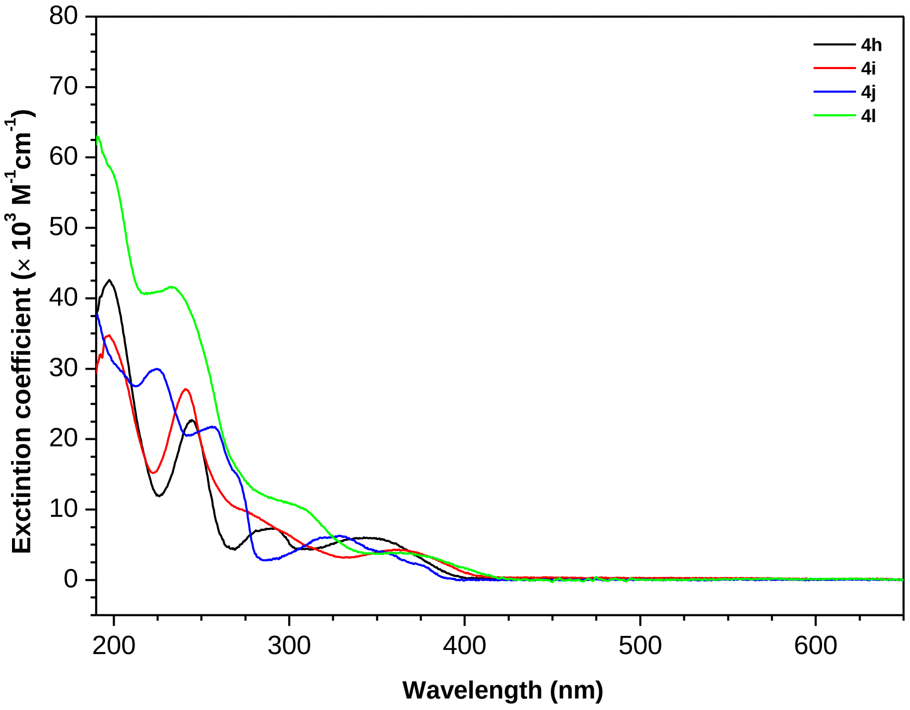

Figure S280 *UV-vis of* **4h**,**4i**,**4j**, and **4l**.

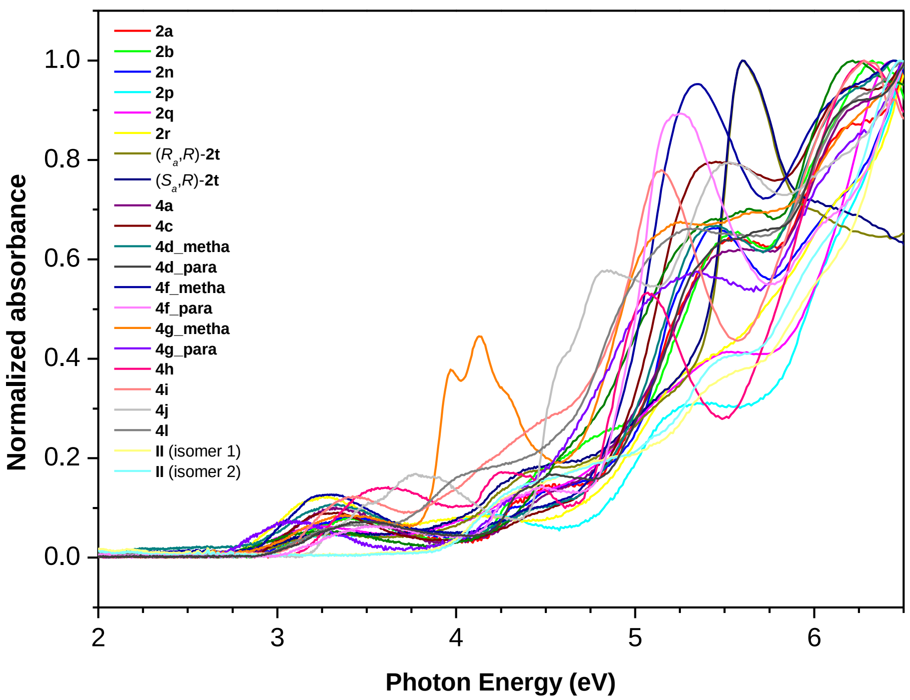

Figure S281 *UV-vis plotted as a function of photon energy used for ΔE^{opt} energy assignment (see **Table S21** below).*

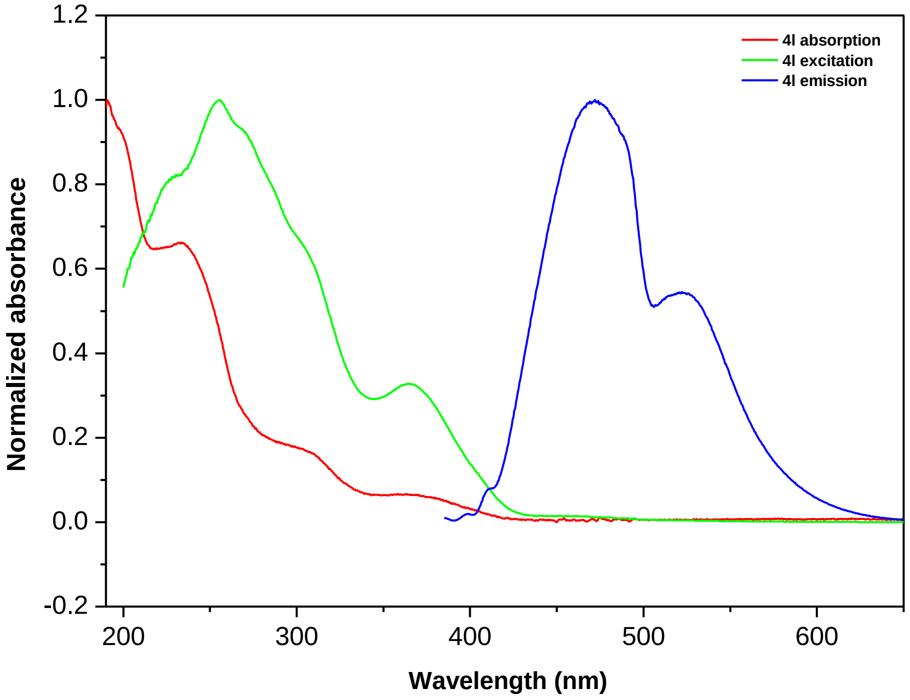

Figure S282 *Normalized absorption, excitation, and emission spectra of* **4l**

Table S21 *UV-vis Data of 1,4-azaphosphinines*

Entry	Compound	Conc. [× 10^{-5} M]	λ_{max}^{abs} ($\Delta\varepsilon$) [nm (M^{-1} cm^{-1})][a]	λ_{0-0}^{abs} ($\Delta\varepsilon$) [nm (M^{-1} cm^{-1})][b]	λ^{edge} [nm][c]	ΔE_{calc}^{opt} [eV][d]	ΔE_{plot}^{opt} [eV][e]
1	**2a**	2.38	199 (30 501), 223 (22 414), 272 sh (5 055), 285 sh (3 641)	363 (2 898)	418	2.97	2.95
2	**2b**	2.44	<u>196</u> (31 227), 223.5 (20 343), 257 sh (7 584), 274 sh (5 516)	366 (2 067)	426	2.91	2.90
3	**2n**	2.36	~202 sh (28 546), 228 (26 142), ~273 sh (5 120), 287 sh (3 576)	367 (2 860)	422	2.94	2.88
4	**2p**	1.48	231 (14 012), 295.5 (3 266)	375 (1 360)	451	2.75	2.78
5	**2q**	1.89	<u>192.5</u> (58 888), 224 (24 381), ~241 sh (19 071), ~286 sh (7 075),	382 (3 680)	451	2.75	2.73
6	**2r**	1.84	~202 sh (31 810), ~234 sh (16 521), 301 (3 514)	381.5 (5 226)	445	2.79	2.78
7	(*R_a*,*R*)-**2t**	1.24	<u>221.5</u> (80 005), ~241 sh (25 145), ~275 sh (14 268)	377.5 (3 767)	444	2.79	2.77
8	(*S_a*,*R*)-**2t**	1.10	<u>221.5</u> (68 444), ~241 sh (22 342), ~275 sh (12 445)	378 (3 127)	445	2.79	2.73
9	**4a**	2.38	~198 sh (31 715), 221.5 (21 490), ~275 sh (4 286)	375.5 (3 394)	427	2.90	2.86
10	**4c**	2.96	<u>199.5</u> (28 567), 220 (19 941), 227.5 (19 376), ~271 sh (5 480)	381.5 (1 295)	450	2.76	2.70
11	**4d**$_{metha}$	2.91	<u>192.5</u> (30 716), ~200 sh (28 711), 227.5 (20 299), ~280 sh (2 444)	375 (2 757)	436	2.84	2.86
12	**4d**$_{para}$	2.51	~198 sh (35 430), ~219 sh (25 227), 274.5 (6 486), ~284 sh (5 205)	368 (2 814)	422	2.94	2.93
13	**4f**$_{metha}$	2.28	<u>192.5</u> (50 882), ~201 sh (47 753), 231.5 (48 433), ~283 sh (4 322), ~294 sh (3 067)	378 (5 914)	433	2.86	2.87
14	**4f**$_{para}$	3.06	~205 sh (20 404), 236 (27 095), 276.5 (4 227), ~288 sh (3 626)	352.5 (1 819)	414	3.00	2.98
15	**4g**$_{metha}$	2.01	~200 sh (28 641), ~219.5 sh (22 730), 236 sh (22 112), ~288 sh (10 197), 300.5 (14 444), 312.5 (12 210)	367 (2 495)	429	2.89	2.84
16	**4g**$_{para}$	2.34	~202 sh (13 433), 232 (9 471)	405.5 (1 096)	461	2.69	2.68
17	**4h**	2.12	<u>197.5</u> (42 588), 244.5 (22 702), 291.5 (7 320)	344 (5 999)	396	3.13	3.13
18	**4i**	2.89	<u>197.5</u> (34 762), 241 (27 114)	365 (4 257)	407	3.05	3.03
19	**4j**	2.00	224.5 (29 972), 257.5 (21 723), ~271 sh (14 633), 328.5 (6 293), ~356 sh (3 734)	~376 sh (2 008)	390	3.18	3.18
20	**4l**	1.57	~200 sh (57 484), 233.5 (41 603), ~306 (10 374)	369.5 (3 727)	424	2.92	2.93
21	**II** (pr1)	1.06	~205 sh (40 897), ~225 sh (25 379), ~257 sh (13 010)	~286 sh (8 083)	316	3.92	3.88
22	**II** (pr2)	1.80	~205 sh (32 858), ~225 sh (20 524)	~286 sh (6 416)	315	3.94	3.91

[a]Absorption maxima in MeCN solution with corresponding values of extinction coefficient; the underlined value signifies the most intense absorption band within 190-650 nm wavelength region; **sh** = shoulder; ~ indicates an estimated value.
[b]Absorption 0–0 electronic transition wavelengths (for ^1L_b state).
[c]Optical absorption edges.
[d]Optical HOMO-LUMO gap calculated as ΔE_{calc}^{opt} = 1240/λ^{edge}.
[e]Optical HOMO-LUMO gap from plot of Absorption vs. Photon Energy (see Fig. S284 in the SI).

6. X-ray analysis

Diffraction data were collected on a Bruker D8 VENTURE Kappa Duo PHOTON 100 CMOS with the monochromated Mo/Cu-Kα radiation. The structures were solved by direct methods (SHELXT[4]) and refined by full-matrix least-squares on F^1 values (SHELXL[4]). All heavy atoms were refined anisotropically. The hydrogen atoms on carbon atoms were fixed into idealized positions (riding model) and assigned temperature factors Hiso(H) = 1.2 Ueq(pivot atom). ORTEP-3[5] was used for structure presentation.

The crystallographic data for the structures reported in this paper have been deposited with the Cambridge Crystallographic Data Centre as supplementary publication. Copies of the data can be obtained free of charge on application to CCDC, e-mail: deposit@ccdc.cam.ac.uk.

X-ray data of 1,4-azaphosphinine (S_a,*R*)-**2t**: $C_{26}H_{22}NO_2P$, M=411.42 g.mol^{-1}, triclinic system, space group *P-1*, a=9.4576(9), b=10.5065(9), c=11.7602(11) Å, α=88.187(3), β=73.477(3), γ=66.218(3)°, Z=2, V=1020.58(16) Å^3, Dc=1.34 g.cm^{-3}, μ(Mo Kα)=0.16 mm^{-1}, T=120 K, crystal dimensions of 0.17 x 0.29 x 0.65 mm, yellow prism. The independent part of the lattice cell is formed by one molecule. It was necessary to model a disorder of the ethoxy group. The occupancy factors of disordered atoms converged to the ratio 0.88:0.12. All heavy atoms were refined anisotropically (Fig. S283). The model converged to the final R=0.0393 and R_w=0.0998 using 4975 independent reflections for 281 refined parameters (θ_{max}=28.29°). CCDC registration number 2489827.

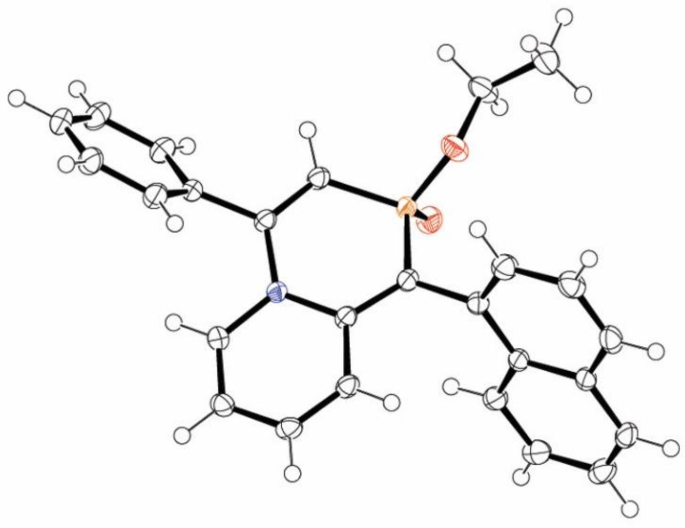

(S_a,*R*)-**2t**

Figure S283 *ORTEP projection of the crystal structure of (*S_a,*R*)-**2t**. *The disordered atoms were omitted for the clarity. Thermal ellipsoids are shown with 50% probability.*

X-ray data of 1,4-azaphosphinine (R_a,R)-**2t**: $C_{26}H_{22}NO_2P$, M=411.42 g.mol^{-1}, triclinic system, space group *P-1*, a=9.5272(3), b=10.7242(5), c=12.0045(4) Å, α=84.793(2), β=68.976(1), γ=63.692(1)°, Z=2, V=1020.87(7) Å^3, Dc=1.34 g.cm^{-3}, μ(Mo Kα)=0.16 mm^{-1}, T=120 K, crystal dimensions of 0.12 x 0.20 x 0.33 mm, light yellow prism. The independent part of the lattice cell is formed by one molecule. It was necessary to model a disorder of the groups situated at the phosphorus atom. The occupancy factors of disordered atoms converged to the ratio 0.59:0.41. All heavy atoms were refined anisotropically (Fig. S284). The model converged to the final R=0.0542 and R_w=0.1350 using 4803 independent reflections for 310 refined parameters (θ_{max}=28.28°). CCDC registration number 2489829.

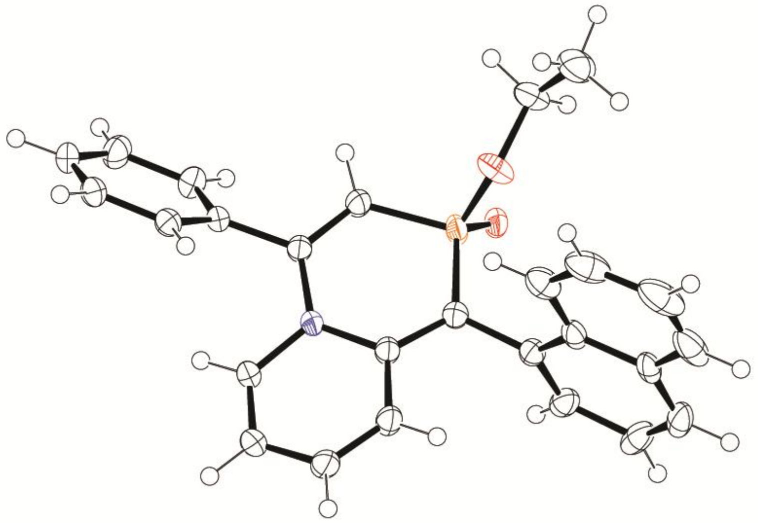

(R_a,R)-**2t**

Figure S284 *ORTEP projection of the crystal structure of* (R_a,R)-**2t**. *The disordered atoms were omitted for the clarity. Thermal ellipsoids are shown with 50% probability.*

X-ray data of 1,4-azaphosphinine **2r**: $C_{23}H_{19}F_3NO_2P$, M=429.36 g.mol^{-1}, triclinic system, space group *P-1*, a=8.1297(3), b=10.4315(3), c=12.5067(41) Å, α=78.517(1), β=76.353(1), γ=71.161(1)°, Z=2, V=966.73(6) Å^3, Dc=1.48 g.cm^{-3}, μ(Mo Kα)=0.19 mm^{-1}, T=120 K, crystal dimensions of 0.21 x 0.28 x 0.42 mm, colourless-yellow prism. The independent part of the lattice cell is formed by one molecule. All heavy atoms were refined anisotropically (Fig. S285). The model converged to the final R=0.0337 and R_w=0.0906 using 4635 independent reflections for 272 refined parameters (θ_{max}=28.27°). CCDC registration number 2489826.

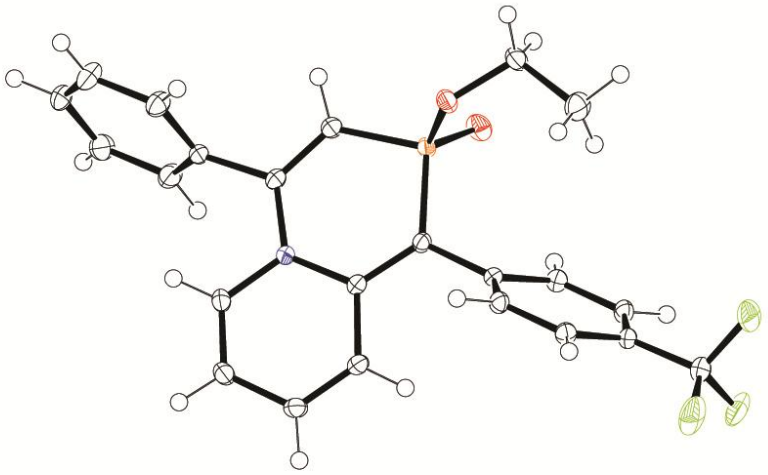

Figure S285 *ORTEP projection of the crystal structure of* **2r***. Thermal ellipsoids are shown with 50% probability.*

X-ray data of 1,4-azaphosphinine **4j**: $C_{20}H_{18}NO_2P$, M=429.36 g.mol^{-1}, triclinic system, space group *P-1*, a=9.6860(5), b=9.8475(6), c=9.9624(5) Å, α=82.794(2), β=74.992(2), γ=62.318(2)°, Z=2, V=812.76(8) Å^3, Dc=1.37 g.cm^{-3}, μ(Mo Kα)=0.18 mm^{-1}, T=120 K, crystal dimensions of 0.07 x 0.15 x 0.18 mm, colourless bar. The independent part of the lattice cell is formed by one molecule. All heavy atoms were refined anisotropically (Fig. S286). The model converged to the final R=0.0320 and R_w=0.0859 using 4007 independent reflections for 218 refined parameters (θ_{max}=28.69°). CCDC registration number 2489828.

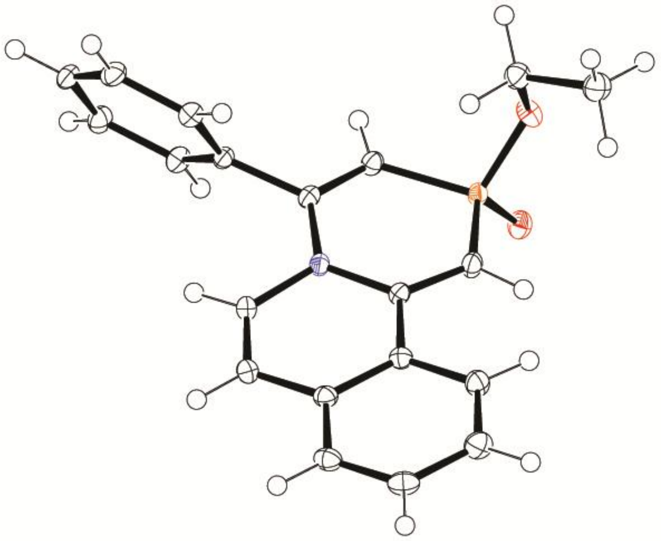

Figure S286 *ORTEP projection of the crystal structure of* **4j***. Thermal ellipsoids are shown with 50% probability.*

7. References

(1) M. J. Frisch, G. W. Trucks, H. B. Schlegel, G. E. Scuseria, M. A. Robb, J. R. Cheeseman, G. Scalmani, V. Barone, B. Mennucci, G. A. Petersson, H. Nakatsuji, M. Caricato, X. Li, H. P. Hratchian, A. F. Izmaylov, J. Bloino, G. Zheng, J. L. Sonnenberg, M. Hada, et al., Gaussian 09, Revision B.01, Gaussian Inc., Wallingford CT, 2009.

(2) A. D. Becke, *J. Chem. Phys.* **1993**, *98*, 5648–5652.

(3) J. P. Perdew, Y. Wang, *Phys. Rev.* B **1992**, *45*, 13244–13249.

(4) G. M. Sheldrick: SHELXL-2017/1, Program for Crystal Structure Solution and Refinement, University of Göttingen, Göttingen, Germany, **2017**.

(5) Farrugia L. J.: ORTEP-3 for Windows - a version of ORTEP-III with a Graphical User Interface (GUI). *J. Appl. Cryst.* **1997**, *30*, 565. https://doi.org/10.1107/S0021889897003117.
