# Supplementary figures and images for: Synthesis of Quinolizidine-Based 1,4-Azaphosphinines via Cyclization of Heteroarylmethyl(alkynyl)phosphinates
Source: J Org Chem. 2025 Dec 22;91(1):698–719. doi: 10.1021/acs.joc.5c02870 (PMC12797286; doi:10.1021/acs.joc.5c02870)

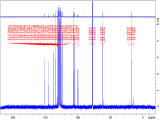

Supplement: Supplementary file 2 [file jo5c02870_si_002.zip › NMR spectra in FID/(Ra,R)-2t/13C/pdata/1/thumb.png]

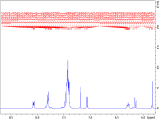

Supplement: Supplementary file 2 [file jo5c02870_si_002.zip › NMR spectra in FID/(Ra,R)-2t/1H/pdata/1/thumb.png]

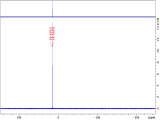

Supplement: Supplementary file 2 [file jo5c02870_si_002.zip › NMR spectra in FID/(Ra,R)-2t/31P/pdata/1/thumb.png]

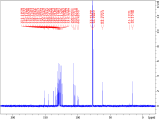

Supplement: Supplementary file 2 [file jo5c02870_si_002.zip › NMR spectra in FID/(Sa,R)-2t/13C/pdata/1/thumb.png]

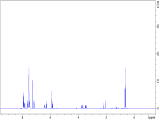

Supplement: Supplementary file 2 [file jo5c02870_si_002.zip › NMR spectra in FID/(Sa,R)-2t/1H/pdata/1/thumb.png]

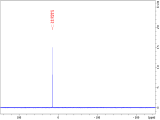

Supplement: Supplementary file 2 [file jo5c02870_si_002.zip › NMR spectra in FID/(Sa,R)-2t/31P/pdata/1/thumb.png]

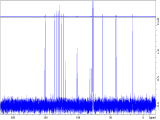

Supplement: Supplementary file 2 [file jo5c02870_si_002.zip › NMR spectra in FID/1a/13C/pdata/1/thumb.png]
